# Peer Review in Law Journals

**DOI:** 10.3389/frma.2021.787768

**Published:** 2021-12-08

**Authors:** Jadranka Stojanovski, Elías Sanz-Casado, Tommaso Agnoloni, Ginevra Peruginelli

**Affiliations:** ^1^ University of Zadar, Zadar, Croatia; ^2^ Centre for Scientific Information, Ruđer Bošković Institute, Zagreb, Croatia; ^3^ Laboratory on Metric Information Studies, University Carlos III of Madrid, Madrid, Spain; ^4^ Institute of Legal Informatics and Judicial Systems, National Research Council, Florence, Italy

**Keywords:** research assessment, peer review, law journal, content analysis, editorial policy, evaluation criteria, publishing ethics

## Abstract

The field of law has retained its distinctiveness regarding peer review to this day, and reviews are often conducted without following standardized rules and principles. External and independent evaluation of submissions has recently become adopted by European law journals, and peer review procedures are still poorly defined, investigated, and attuned to the legal science publishing landscape. The aim of our study was to gain a better insight into current editorial policies on peer review in law journals by exploring editorial documents (instructions, guidelines, policies) issued by 119 Croatian, Italian, and Spanish law journals. We relied on automatic content analysis of 135 publicly available documents collected from the journal websites to analyze the basic features of the peer review processes, manuscript evaluation criteria, and related ethical issues using WordStat8. Differences in covered topics between the countries were compared using the chi-square test. Our findings reveal that most law journals have adopted a traditional approach, in which the editorial board manages mostly anonymized peer review (104, 77%) engaging independent/external reviewers (65, 48%). Submissions are evaluated according to their originality and relevance (113, 84%), quality of writing and presentation (94, 70%), comprehensiveness of literature references (93, 69%), and adequacy of methods (57, 42%). The main ethical issues related to peer review addressed by these journals are reviewer’s competing interests (42, 31%), plagiarism (35, 26%), and biases (30, 22%). We observed statistically significant differences between countries in mentioning key concepts such as “Peer review ethics”, “Reviewer”, “Transparency of identities”, “Publication type”, and “Research misconduct”. Spanish journals favor reviewers’ “Independence” and “Competence” and “Anonymized” peer review process. Also, some manuscript types popular in one country are rarely mentioned in other countries. Even though peer review is equally conventional in all three countries, high transparency in Croatian law journals, respect for research integrity in Spanish ones, and diversity and inclusion in Italian are promising indicators of future development.

## 1 Introduction

Research of the peer review process, “the backbone of modern science” according to Bornmann (2015) or “a flawed process at the heart of science and journals” according to Smith (2006), reveals its power and imperfections. It often relies on the analysis of journal guidelines for authors and reviewers, which give an insight into journal mission, scope, criteria, and editorial practices. However, in legal science, such research is scarce due to the specificities of this field. There is no established culture of peer review in law journals and no uniform system of peer review that function across national borders. This is the reason why research of peer review in law journals is particularly challenging.

The description of law as a science includes a series of interpretations and offers many hypotheses about the meaning and scope of legal concepts, rules, and principles that may be confirmed or rejected through scholarly research. In the academic context legal science could be observed through different manifestations of law, namely “law as a practical discipline”, “law as humanities”, and “law as social science” (Siems and Mac Sithigh, 2012). All three categories are relevant within legal academia and can map how far institutions, individuals, and legal cultures belong to one or more of these categories (Conti and Peruginelli, 2021). Roughly speaking, legal science is a scholarly discipline in its own right with a methodology that, in its core characteristics, is quite comparable to the methods used in other disciplines (Rubin, 2010). Nevertheless, there is no agreement among legal theorists on the nature of legal science as a discipline (Hoecke, 2011, 17). In such a panorama, different definitions, standards, and practices are used for the assessment (Castermans and Amtenbrink, 2015), as they acknowledge differences in legal science disciplines (civil, administrative, labor, criminal, commercial), research (doctrinal, comparative, empirical), scope (national, regional, or international), choice of language (local or English), and strong links between practice and research (Conte, 2015; Van Gestel and Lienhard, 2019).

As a body of work produced by academics, legal scholarship is in a remarkable and singular position, because not only is it the way to communicate the science of law but is also an influential and authoritative source of law (Gutwirth, 2009, 70). It deals with different branches of law (i.e., civil, administrative, labor, criminal, commercial law), different schools of interpretation within these branches, and different communication tools. Moreover, there is a strong link between legal scholarship and law practice, given that both rely on similar instruments for analysis, practical argumentation and reasoning. Thus, legal scholarship represents both the science of law and one of the authoritative and influencing sources of the law with its “intrinsic connection with the national environment, parliaments, courts and administrative bodies that create national law and practice in national languages” (Hojnik 2021, 9). As a result, legal science has to pass two “exams”: a quality test within legal academia, which first evaluates its robustness as scientific research and then assesses its pertinence and relevance to legal practice (Gutwirth 2009).

Another peculiarity is related to authorship, as legal researchers usually work alone (Peruginelli and Faro, 2018, 108) and give a personal and original interpretation of research subject within a framework of pluralistic views. Furthermore, many scholarly law journals publish contributions by practitioners (judges, professionals, public servants). Each genre also has its specific perceived value in the legal community, whether it is new knowledge (Raimo 2015) or expertise in case law. Additionally, legal scholarship strongly influences society’s core values, such as justice, freedom, equality, human dignity, and solidarity. One of the objectives of legal scholars of positive law is to influence the world with their research. However, this kind of influence is difficult to measure, as case law and legislation in many jurisdictions do not contain doctrinal citations. All of these aspects are particularly relevant for law journals, as they influence the process of research assessment and should be taken into account in designing a model to measure the quality of legal scholarship.

In general, research evaluation consists of two parts: qualitative (peer review, prominence of authors, publication’s or publisher’s prestige) and quantitative (number of publications, number of citations). Quantitative “research evaluation” relies on many metric indicators, some of which have prevailed thanks to ease of use, especially in the STEM (science, technology, and medicine) areas. One such indicator–journal impact factor (JIF)—has become an indispensable measure the “quality” of research results published in journals. However, because there are many complex and specific issues to consider, none of the numerous discussions between legal academics has yielded an evaluation system that would be globally accepted within the legal community. Instead, two ways to evaluate research have prevailed, although unresolved problems are present in both. The first is the peer review process to which manuscripts are subjected prior to publication, and the second is the ranking of journals based on citations or peer review.

Still, there is no commonly recognized transnational assessment system that would make it possible to benchmark and rank law journals and other legal science outputs (van Gestel 2015; Stolker 2014; Hojnik 2021). Law journals are multidisciplinary and attract manuscripts from various fields, including anthropology, economics, history, political science, psychology, and sociology. Scholars from various disciplines might perceive the same journals differently, which calls for discipline-based journal ranking (Collins, 2018). Only a few resources allow their assessment through popular quantitative indicators of visibility and impact. One is the Washington and Lee University School of Law list of more than 1.500 law journals (1.000 from the US and 500 non-US) included in the Westlaw database and ranked by the number of citations. SCImago Journal & Country Rank (SJR)^1^ in turn, ranks 770 law journals according to citations in the Scopus database. There are also subscription-based services like InCites Journal Citation Reports (150 law journals) and HeinOnline Law Journal Library’s most cited 100 journals. All these, however, strongly favor “internationally leading” law journals published in English, while the majority of national law journals across Europe, constituting the core of legal publishing in each country, are poorly represented (Gutwirth 2009) and not included in multidisciplinary databases like Web of Science Core Collection and Scopus.

The field of law has retained its distinctiveness regarding peer review to this day, and reviews are often conducted without following standardized rules and principles. In addition, the number of journals has consistently increased, and more and more papers are published in journals with low level of critical assessment. In most European countries evaluation of legal scholarship relies on both editorial and independent/external peer review. This co-existence reflects the lack of a general agreement about quality indicators and the lack of a lingua franca that would expand the audience and scrutiny beyond national borders (Van Gestel and Lienhard, 2019, 428). In fact, the legal community is not always enthusiastic about external peer review, and some law journals prefer review undertaken by members of the editorial staff. Recently this practice has received severe criticism, especially where editing, selecting, and reviewing papers for publication falls on the shoulders of law students (Friedman, 2018). There is no question that student-edited law journals contribute to legal science, but usually they do not have the same weight as peer-reviewed journals (Collins, 2018). Moreover, some authors argue that even if student editors are to some extent competent to evaluate traditional legal or “doctrinal” scholarship, they are by no means competent to evaluate increasingly interdisciplinary papers (Friedman, 2018). Nevertheless, editors will continue to retain broad discretion over the review process in law journals (Zhang, 2018).

For specific branches of law and for topics limited to the national context (e.g., taxes, employment procedures) the legal scholarly community might be so small that anonymized peer review makes no sense because colleagues would still recognize each other’s work. For this reason, blind peer review is rarely used in some countries. While involving reviewers from abroad might provide a solution, foreign reviewers may find themselves at a disadvantage if they do not know the language or specific national topics (Maier 2019, 122). Furthermore, in most jurisdictions, editorial boards are rather vague about assessment criteria, nor do they know how to apply existing criteria to different types of legal research (theoretical, comparative, practical) to avoid bias (van Gestel, 2017).

Like in other disciplines, peer review in the law domain entails no institutional recognition or reward in terms of academic appointments and promotion. Various studies show that peer review is slow and inefficient, expensive, ineffective (in detecting errors, plagiarism, and fraudulent research), biased, opaque, easily abused, unreliable, and without incentive (Godlee, Gale, and Martyn, 1998; Wager and Jefferson, 2001; Bornmann, Nast, and Daniel, 2008; Birukou et al., 2011; Yarkoni, 2012; Lee et al., 2013; Nguyen et al., 2015; Sciullo, 2015; Walker and Rocha da Silva, 2015; Garrido-Gallego, 2018; Knöchelmann et al., 2019; Ross-Hellauer and Derrick, 2019). Many of these shortcomings fall into the domain of publishing ethics and can be overcome with greater transparency of the peer review process, including open peer review (Heeks, 2011; Pöschl, 2012; Hachani, 2015; Nosek et al., 2015; Schmidt et al., 2018; Wolfram et al., 2020).

Yet, its deficiencies and weaknesses do not diminish its value and relevance. Instead, they call for changes and more innovative approaches (Walker and Rocha da Silva, 2015). Peer review is still considered “an important gatekeeper and key component of scientific discourse” (Aleksic et al., 2015), “the best available practice to ensure the quality and correctness of the scientific literature” (Horbach and Halffman, 2018), and “the cornerstone of the scientific world” (Nguyen et al., 2015). Legal scholars agree on the benefits of peer review and consider it the least controversial method of evaluating the quality of scholarly publications (van Gestel and Vranken, 2011, 902). Recent years have shown a clear trend towards independent peer review as a relevant quality indicator, like in Swiss and Dutch law journals (Van Gestel et al., 2018). Still, how this should work in practice and what quality indicators should referees apply remains open (Van Gestel and Lienhard, 2019).

Publishing in peer-reviewed journals not only benefits researchers for their professional advancement but also improves the image of academic institutions and disciplines, as scholars generally believe that peer-reviewed journals produce quality science (Sciullo, 2015). However, whether the legal community retains editorial review (including student review) as the prevailing evaluation model, replaces it with peer review, or develops its own model based on the strengths of both, the transparency of manuscript evaluation will play a key role in improving the quality of legal scholarship. Moreover, such transparency will entail responsibility and accountability of those involved in scientific gatekeeping (Reinhart and Schendzielorz, 2021).

Transparency is also in the focus of our study. We wanted to see to which extent peer-reviewed Croatian, Italian, and Spanish law journals are transparent in their editorial policies by asking ourselves four questions: where is the their peer review process information located, how are the elements of the peer review process addressed, what evaluation criteria are reviewers asked to follow, and what ethical concerns do law journals mention in their documents on peer review?

## 2 Materials and Methods

We began research on various aspects of legal science assessment in 2018 as part of the COST Action ENRESSH–European Network for Research Evaluation in the Social Sciences and Humanities, which ended in February 2020 (Peruginelli, Sanz-Casado, Stojanovski 2020; Peruginelli et al., 2021). The idea for a specific study of peer review in law journals was conceived during an STSM visit to the Institute of Legal Informatics and Judicial Systems at CNR in Italy in November 2019, when a methodology for data collection and analysis was outlined.

### 2.1 Data Collection

Journals to analyze were selected in two steps. In the first step, we limited our research to law journals from Croatia, Italy, and Spain to cover small and large European countries in which English is not native language, yet publish much of their legal research in English. In the second step we selected journals with a sufficient level of transparency of editorial policy which declare themselves peer-reviewed in publicly available documents.

Croatian law journals were selected from the HRČAK Portal of the Croatian Scientific and Professional Journals^2^, while the Italian and Spanish journals were selected from the list of A-rated law journals by the Italian National Agency for the Evaluation of Universities and Research Institutes (ANVUR)^3^ and from the list of journals approved by the Spanish Foundation for Science and Technology (FECYT)^4^. Each country applies a set of criteria to evaluate journals in all SSH disciplines, including law (Peruginelli et al., 2021), and differences in the publishing landscape of the three countries (Peruginelli, Sanz-Casado, and Stojanovski 2020) provide more dimensions for studying peer review. The selection included 36 law journals for Croatia and Spain each. To get an Italian sample of comparable size we then randomly selected 52 law journals from list of 154 A-rated ones. As all Croatian and Spanish journals have publicly available documents addressing peer review, and five Italian journals did not satisfy this criterion, we excluded these from further analysis. Our final sample consisted of 119 law journals (36 Croatian, 47 Italian, and 36 Spanish).

Then we ran a pilot study collecting only instructions to reviewers (ItR) and peer review policies (PRPol) but found that ItRs mostly did not contain information on the peer review process, and only a few journals had publicly available PRPol. This is why we had to expand research to all the most recent versions of every publicly accessible document issued by the editorial board mentioning peer review for each journal. The documents were taken from journal or publisher websites, national journal databases, and where no document was found, from the content of a recent issue. Altogether we collected 135 documents in different formats (PDF, DOC, and HTML) with information on the journal title, publisher’s country, ISSN, format, language, document URL, and document title (Supplementary Table S1). The documents were collected in January 2021 and updated in June 2021.

The documents were then categorized by where the information on peer review is located, as follows: instructions to authors (ItA), instructions to reviewers (ItR), peer review policies (PRPol), review forms (RefForm), editorial policies (EdPol), publishing ethics (PubEth), about the journal webpage (AboutJ), other journal webpages (WP), and notes (Note). Were no English version of the document was available we collected information in the local language (Supplementary Table S1).

### 2.2 Content Analysis

The 135 collected documents addressing peer review were analyzed using automatic content analysis, in which each document (case) was the analytical unit. For text-mining, content analysis dictionary, descriptive statistical analysis, analysis of co-occurrences, and qualitative insight we used the QDA Miner5 and WordStat8. Additional variables “country” and “language” were defined in the QDA Miner and used to compare categories between the three countries and English and non-English documents. We did not convert the documents to lowercase, since WordStat is case-insensitive. In addition, we used a list of stopwords in English, Italian, Spanish, and Croatian to exclude the most common words from the analysis (Diaz Gene, 2020).

In WordStat we developed a multilevel and multilanguage categorization dictionary in four languages (English, Croatian, Italian, and Spanish) (Supplementary Table S3, Data sheet S1). Three main categories—“Peer review process”, “Peer review evaluation criteria”, and “Peer review ethics” –were defined according to our research questions. For each category, several subcategories were defined down to the fifth hierarchical level. Altogether, we defined 75 categories and subcategories to record the appearance of different peer review elements and related research integrity and ethical topics in our corpus. Within the subcategories we defined 441 keywords (words, word patterns, phrases, and coding rules) in four languages for coding and analysis. Thus, the peer review categorization dictionary was partly data-driven (content in the documents/cases) and partly concept-driven (framing categories and subcategories). A simplified version of the categorization dictionary is presented in Table 1 and the full version of categorization dictionary is available in XLSX and CSV format in the (Supplementary Materials).

**TABLE 1:** A simplified version of the peer review categorization dictionary.

Category	Subcategory	Examples of keywords
PR_process	Goverance	Editor-in-chief, editorial board, scientific/advisory board, steering committee
	ID_transp	Transparency of reviewer’s, author’s and editor’s identity: visible, anonymous (single, double, triple)
	Reviewer	Number of reviewers (one, two, three, or more), competencies, objectivity, autonomy, independence
PR_criteria	Publ_type	Article (scholarly, professional), review article, case note, essay
	Eval_criter	Design, methods, relevance, appropriateness, theory, writing style, literature
	PR_outcome	Acceptance (with or without revision), rejection
PR_ethics	Coi	Conflict of interest/competing interest
	Confident	Confidentiality
	EDI	Equity, diversity, inclusion, bias (gender, race, religion, nationality)
	Res_misconduct	Data fabrication and falsification, text recycling
	Text_similar	Plagiarism, self-plagiarism, text similarity-check

Within the categorization dictionary we defined several categories that contained general terms in four languages irrelevant for analysis, namely, “PR_gen” (peer review, evaluation), “eval_cri_gen” (criteria), “reviewer_gen” (reviewer, referee), “submiss_gen” (article, contribution, submission, manuscript) and “ethics_gen”(ethics, ethical). These general categories served to gain insight into the overall representation of the most common terms in the documents and create rules by which to record the presence of more specific categories and keywords. For example, for the “Title” we created the rule @TITLE (TITLE NEAR #SUBMISS_GEN to register “article title”, “title of the paper”, “submission title”, etc., Such general categories are excluded from the tables below, because they are very frequent and irrelevant, but are included in Supplementary Table S2. Additionally, to reduce table size we removed categories represented in 10% or less of the documents (Tables 3, 4, and 5) or represented in 50% or less of the documents (Tables 6 and 7).

The spreadsheet with a list of all journals and documents in the sample, a full version of peer review categorization dictionary, all documents in RTF format, and a spreadsheet with all results from WordStat8 are available in the Supplementary Figshare.

Descriptive statistics were used to summarize the data, and comparisons between the groups were analyzed with chi-square test. A *p*-value <0.05 was considered statistically significant. Representation of categories shows the frequency of the appearance, number of documents (cases), and percentages of documents containing the keyword. Besides considering the frequency of categories in the documents, we also assessed co-occurrences of categories, and their relation to each other within documents. Moreover, the keyword-in-context (KWIC) feature, displaying the original content and the keyword’s position, added the qualitative aspect to the analysis.

## 3 Results

### 3.1 Document Categorization

Information on peer review is scattered across various types of documents issued by the journal. Most information was found in the instructions for authors, editorial policies, and web pages describing the journal, usually under the link ‘About journal’ (Table 2). We also found scarce information on the journal homepage or even hidden in the footnote of the journal’s issue (recorded as a ‘Note').

**TABLE 2:** Documents included in content analysis by type.

	ItA	ItR	PRPol	RevForm	EdPol	PubEth	AboutJ	WP	Note	Total
Croatia	21	8	0	7	3	2	0	7	0	48
Italy	7	6	10	0	4	8	3	3	2	43
Spain	11	2	2	1	13	0	15	0	0	44
ToTAL	39	16	12	9	20	9	18	10	2	135

ItA: instructions to authors; ItR: instructions to reviewers; PRPol: peer review policy; RevForm: review form; EdPol: editorial policy; PubEth: publishing ethics; AboutJ: about journal; WP: WebPage.

Among the 135 documents, 69 were in English (29 Croatian, 13 Italian, and 27 Spanish documents), 30 in Italian, 17 in Spanish, and 19 in Croatian.

The top three categories are represented as follows: “PR evaluation” with 3,174 mentions (130, 96%), “PR process” with 1,290 mentions (129, 96%), and “PR ethics” with 478 mentions (73, 54%).

### 3.2 Peer Review Process

In order to find out what organizational and quality elements of the “PR process” are addressed in the documents, we analyzed three aspects: involvement of editorial and other boards and committees in the process, the transparency of identities during the peer review, and preferred characteristics of the reviewers. Accordingly, the “PR process” category in the dictionary includes three subcategories represented differently in the corpus of the collected documents (Table 3): “Governance” (68%), “ID transparency” (76%), and “Reviewer” (89%).

**TABLE 3:** Peer review process categories (found in more than 10% of the documents).

			Mentions	Documents	
Category (1st level)	Category (2nd level)	Category (3rd level)	Frequency	Number	%
PR_process	—	—	1,290	129	96
—	Goverance		621	108	80
—	—	ED_board	392	81	60
—	—	Editor-in-cheif	136	51	38
—	—	SCI/advisor_board	59	23	17
—	ID_transp		332	104	77
—	—	Anonym	331	104	77
—	Reviewer	—	337	98	73
—	—	Independ	116	65	48
—	—	Competence	98	51	38
—	—	Objectivity	58	31	23
—	—	Number	65	29	21

#### 3.2.1 Governance

The journal’s governance could be defined by journal owner (university, learned society), publisher, or journal. It usually involves different boards, committees, and councils with various duties and responsibilities. When it comes to the bodies that manage the journal, there is no uniform terminology in a particular language, nor are there uniform roles and responsibilities related to the peer review process. The most considerable differences in terminology were recorded in Italian journals, less in Spanish, while Croatian journals were uniform regarding the used terminology in both languages.

The main pillars of the journal’s governance are editor-in-chief, editorial board, and advisory board, responsible for the journal’s mission and composed of renowned experts in the fields covered by the journal. Their full names, affiliations, and contact information are usually given on the journal website. Members of the editorial board are primarily responsible for selecting submitted manuscripts for publication. Some journals may require additional assessment by members of the advisory board, which supports the editorial board in academic duties. The journal may also have a board of reviewers and a steering committee.

The editorial board comprises “renowned experts in the fields covered by the journal” (#123, Spain) or “university professors and researchers, magistrates and well-known lawyers” (#73, Italy). Additionally, members of the editorial board should have “research skills, proved and acknowledged by their active and regular participation in the assessment processes of leading national and foreign publications, experience in managing and editorial tasks in other scholarly journals; and full availability in the performance of their duties” (#84, Spain). The editorial board is responsible for “keeping the editorial line of the journal and for adequately running the manuscript selection process” (case 84, Spain), including the peer review process. “Journal’s ‘management approves a list of evaluators external to the bodies of the journal, made up of Italian and foreign scholars particularly qualified in the field of study, whose inclusion in the list can be proposed by each member of the management” (# 62, Italy).

In most law journals, the editor-in-chief and editorial board check whether the submission fits the journal’s scope and its overall quality. The manuscripts whose quality is low or outside the scope may be rejected without external review. Those that pass this first step are sent to external reviewers. When reviewer reports disagree or additional opinion is needed, the editorial board can send the manuscript to another reviewer. Once the editorial board has all reviews, it makes one of the following decisions and communicates them to the author(s): accept without revision, accept with revisions, or reject. The distinction between minor and major revisions was noted in only a few Croatian and Spanish journals.

If appointed, the advisory board usually monitors the development of the journal, suggests new topics and special editions, evaluates journal’s yearly performance, and submits comments, proposals, and complaints to the editor-in-chief, editorial board, or journal owner. Some law journals ask members of the advisory board to make the initial assessment of submitted manuscripts in terms of quality and fitting the journal scope: “All manuscripts are evaluated, first, both by the editors-in-chief and one or more members of the advisory board.” (#74, Spain). Members of the advisory board can also assist in checking and verifying substantially revised manuscripts in terms of journal requirements and suggestions given by external reviewers. Instead of “advisory board” some journals (primarily Italian) use the term “scientific board” to describe similar roles and responsibilities.

Under the “Governance” category, we identified three governing instances involved in the peer review process: the “Editorial board” (81, 60%), “Editor-in-chief” (51, 38%), and the “Advisory board” (23, 17%). The steering committee, board of reviewers, and other boards, councils and committees are referred to in less than 2% of the documents.

The ‘ID transparency’ category, which includes information about peer review being single-blind, double-blind, or open, was referred to 332 times across 104 documents (77%), and all of them spoke about anonymized peer review. “Double-anonymized” peer review was mentioned in 50 documents (37%) and “Single-anonymized” in 21 documents (16%). The single mention of open peer review (visible identities), not represented in Table 3, was wrongly interpreted by the journal and described as a single-blind peer review.

#### 3.2.2 The Reviewer

In our study, reviewer’s “Independency” was the most represented subcategory in the “Reviewer” category (65, 48%), followed by “Competencies” (51, 38%). A deeper look into the documents reveals that reviewers are sometimes recruited from advisory, scientific, reviewer, or even editorial boards. Some journals recruit “external academics not belonging to the editorial board” (#81, Spain) or “experts in the field external to both to the Editorial Board and the Advisory Board” (#122, Spain). When declared, “independence” most often means independence from a journal and its bodies and less often from an institution or publisher. Less represented are keywords describing reviewer’s “Objectivity” (31, 23%). Two reviewers per manuscript are the prevalent model (24, 18%), and “Three and more” are less common [(8, 6%) and (15, 11%) respectively]. Even though a reviewer is expected to keep manuscript information and ideas confidential in closed and blinded peer reviews (Stojanovski, 2015), references to confidentiality of the peer review process were found in less than 10% of the documents, and this subcategory was excluded from Table 3.

### 3.3 Peer Review Evaluation

Typically, manuscript evaluation relies on specific journal criteria and refers to peer review outcomes, represented accordingly by the categories “Evaluation criteria” and “Peer review outcome” (Table 4). The category “Publication type” was added because our preliminary study found that peer review in law journals strongly depends on it. Peer reviewers are often asked to categorize a manuscript by publication type. It turned out to be well-represented, with 481 mentions in 101 documents (75%).

#### 3.3.1 Manuscript Type

Law journals welcome a variety of “genres”, and review practices differ with manuscript types. In some law journals all submissions undergo peer review, and in others only manuscripts with scientific content are sent to external reviewers. Subject to peer review are usually scholarly articles, professional articles, preliminary communications, review articles, essays, case comments/notes, and legislative comments/notes. Peer review is not always mandatory with regional observations, expert reviews, forums, and conference papers.

We included twelve publication types in this category: article (scholarly and professional), book review, preliminary communication, note, essay, conference paper, review paper, debate, experience, conference review, critical discussion, and comment. The most popular category, “Article” (68, 50%), has two subcategories: “Scholarly article”, which is more common (63, 47%), and “Professional article” (35, 26%). Law journals use the attribute “scientific,” “scholarly,” and “academic” for journal articles interchangeably to denote “the paper which is characterized by originality of conclusions, or which presents previously unpublished original results of scientific research” (#5, Croatia). “Book review,” “Preliminary communication” and “Note” share a 20% representation, give or take, while “Essay” (19, 14%), “Conference paper” (18, 13%), “Review paper” (14, 10%), and “Debate” (13, 10%) are less common (Table 4).

**TABLE 4:** Peer review evaluation criteria categories (found in more than 10% of the documents).

			Mentions	Documents	
Category (1st level)	Category (2nd level)	Category (3rd level)	Frequency	Number	%
PR_evaluation	—	—	3,174	130	96
—	Publ_type		520	103	76
—	—	Article	244	68	50
—	—	Book_rev	36	27	20
—	—	Pre_comm	42	26	19
—	—	Note	53	26	19
—	—	Essay	41	19	14
—	—	Conf_paper	51	18	13
—	—	Rev_paper	16	14	10
—	—	Debate	18	13	10
—	Eval_criteria		2024	122	90
—	—	Relevance	709	113	84
—	—	Writing and present	521	94	70
—	—	Ref_liter	518	93	69
—	—	Method and stat	148	57	42
—	—	Theory	82	34	25
—	—	Design and concept	30	18	13
—	PR_outcome	—	630	110	81
—	—	Acceptance	517	108	80
—	—	Rejection	113	53	39

#### 3.3.2 Peer Review Evaluation Criteria

Journals are dedicated to publishing high-quality articles on legal scholarship that can be read by a large community of practitioners and the general public, and peer review serves to reach these goals. For the review process to ensure quality evaluation, journals should provide clear and complete evaluation criteria to authors, reviewers, and editors, in line with the journal’s scope and mission. To define categories for ‘PR evaluation’, we relied on the Bornmann, Nast, and Daniel study (2008) that identified 572 criteria and grouped them into nine main categories: 1) “relevance of contribution”, 2) “writing/presentation”, 3) “design/conception”, 4) “method/statistics”, 5) “discussion of results”, 6) “references to the literature and documentation”, 7) “theory”, 8) “author’s reputation/institutional affiliation”, and 9) “ethics”. We modified the “relevance” category by adding the “Originality”, “Appropriateness”, “Contribution to practice”, and “Significance” subcategories. To ‘Writing&Presentaton’ we added the “Clarity”, “Accuracy”, “Publication Guidelines”, and “Structure&Layout” subcategories. The last was further divided into “Discussion”, “Title”, “Abstract”, “Introduction”, and “Conclusion”. We also added the “Replicability” subcategory to “Design/Concept”. Being a category *per se* in our categorization dictionary, we did not include ‘Ethics’ in “PR evaluation”. The final number of categories in our “PR evaluation” was seven, as follows: “Relevance”, “Writing and Presentation”, “Design/Concept”, “Method/Statistics”, “Theory”, “Reference to literature”, and “Author reputation”.

The “Evaluation criteria” category was identified in 90% of the documents, including subcategories (Table 4). The most common third level category was “Relevance” (113, 84%) with four subcategories: “Originality” (106, 79%), “Significance” (37, 27%), “Appropriateness” (20, 15%), and less represented “contribution to practice” (6, 4%). For “Author reputation”, we found only nine mentions in seven documents, and this category is not included in the table.

Other well-represented categories were “Writing&Presentation” (95, 70%), “References and literature” (93, 69%), and “Methods and Statistics” (58, 43%). Other categories in the “Evaluation criteria” were found in less than 50% of the documents. For example, “Publishing guidelines” were mentioned by 14 documents (25%) only.

#### 3.3.3 Peer Review Process Outcome

Peer review outcome in the applied taxonomy contains two categories, “Acceptance” (108, 80%) and “Rejection” (53, 39%) (Table 5). As multiple revisions of manuscripts most often result in acceptance, the category “Revision” (76, 56%) is included in the parent category “Acceptance”. However, the manuscript may be accepted as publishable without additional revisions (save for copy-editing):

“The main criteria for acceptance are methodological rigour and consistency, text structure and framework; scientific originality and relevance; the validity of the reasoning supporting the central thesis; accuracy/completeness of sources and bibliography.” (#51, Italy).

We did not include the concepts of minor and major revisions in our corpus, even though some journals describe the process of manuscript revision exhaustively:

“If the manuscript has been accepted with modifications, the authors will have to resubmit a new version of the article, taking into account the demands and suggestions of the external evaluators. If they wish, the authors can also provide a letter to the Editorial Board to indicate the content of the modifications to the article. Articles with important corrections may be sent to the Advisory Board to verify the validity of the modifications made by the author. Depending on the degree of compliance with the requested modifications, the Advisory Board will decide whether or not to publish the article. This decision will be communicated to the author by the director of the journal.” (#124, Spain).

### 3.4 Ethical Concerns Related to the Peer Review

Legal scholarship has adopted peer review as a cornerstone of modern legal science. It relies on legal experts to deliver objective, constructive, and consistent evaluation intended to help editors make an informed decision and authors to improve their manuscript. Still, peer review can be slow, expensive, inconsistent, biased, unreliable, unable to detect errors, and abused (Smith 2006; Walker and Rocha de Silva, 2015). This, in conjunction with the “blindness” of the review process, in which the editor moderates the closed discussion between the author and their peer and makes a final decision (Teixeira da Silva and Jaime, 2019), raises numerous ethical concerns. We therefore wanted to identify the extent to which editors of law journals refer to ethical issues to ensure that manuscripts comply with ethical principles.

Studies show that peer review in SSH can be the source of all sorts of biases arising from differences between reviewer’s and author’s affiliation, nationality, language, gender, race, religion, interpretation, ideology, and level of conservatism (Shatz 2004; Lee et al., 2013). In single-blinded peer reviews, invited reviewers are often asked to declare potential conflicts of interest before accepting the invitation. Conflicts of interest “… may involve financial conflicts (having received any monetary compensation from any parties that may be involved in funding or developing the study), academic commitments (being a member of the group or institution involved in the study or being involved in a similar study that may lead to an impartial assessment of the submitted manuscript), and personal relationships.” (Wachholz 2019).

Our analysis included six ethical issues: conflict of interests, research misconduct, (non)adherence to ethical guidelines, equity/diversity/inclusion (EDI), plagiarism, and confidentiality. The World Association of Medical Editors (WAME) suggests that editors should address conflict of interest as follows:

“Conflict of interest exists when a participant in the publication process (author, peer reviewer, or editor) has a competing interest that could unduly influence (or be reasonably seen to do so) his or her responsibilities in the publication process (submission of manuscripts, peer review, editorial decisions and communication between authors, reviewers and editors).” (Ferris and Fletcher 2010).

Detecting research misconduct, like data falsification or fabrication, could be one of the most challenging tasks for reviewers. However, the burden of upholding the journal’s ethical standards rests on editors’ shoulders, and they often rely on guidelines such as those issued by the Committee of Publication Ethics (COPE) or International Committee of Medical Journal Editors (ICMJE).

Of the three top categories represented in law journals “Peer review ethics” ranked the lowest (74, 54%). Table 5 shows that its category “Conflict of interest” ranked the highest with 31%, followed by “Text similarity” (26%), “EDI” (22%), “Ethical guidelines” (21%), and “Research misconduct” (19%). Being below the 10% threshold, “Confidentiality” is not included in the table.

**TABLE 5:** Peer review ethics categories (found in more than 10% of the documents).

			Mentions	Documents	
Category (1st level)	Category (2nd level)	Category (3rd level)	Frequency	Number	%
PR ethics	—	—	478	73	54
—	Coi		132	43	32
—	Text_similar		85	35	26
—	Edi		111	30	22
—	Eth_guid		64	28	21
—	Res_misconduct		70	25	19

As for the top ranking category, some journals clearly state that it is not acceptable for a reviewer to participate in the same project, to mentor, or otherwise be involved with the manuscript under review.

“Reviewers are required not to accept for reading articles for which there is a conflict of interest due to previous collaborative or competitive relationships with the author and/or his/her institution.” (76, Italy).

By accepting to review, the reviewers implicitly declare no such conflict of interest.

### 3.5 Country and Language Differences

To compare possible differences between the three countries, we used the Crosstab feature of WordStat8, which tabulates case occurrences across the “country” variable. The difference in case occurrences was assessed with the χ2 (chi-squared) test, and the threshold of statistical significance was set at *p* < 0.05 (Table 6).

**TABLE 6:** Distribution of categories identified in at least 50% of the documents by country (Croatia 48, Italy 43, Spain 44).

Category (1st level)	Category (2nd level)	Category (3rd level)	Category (4th level)	Croatia	Italy	Spain	Chi2	P (2-tails)
					Number of documents			
PR process	—	—	—	44	42	43	2.653	0.265
—	Governance	—	38	19	35	16.692	<0.001^∗∗∗^
—	—	Edboard	38	9	34	40,166	<0.001^∗∗∗^
—	ID_transp	—	28	35	41	16.437	<0.001^∗∗∗^
—	—	Anonym	—	28	35	41	16.437	<0.001^∗∗∗^
—	—	—	Double anonym	14	10	26	13.954	0.001^∗∗^
—	Reviewer	—	27	33	37	9.545	0.008^∗∗^
—	—	Independ	11	19	35	29.884	<0.001^∗∗∗^
—	—	Competence	8	19	23	13.863	0.001^∗∗^
PR evaluation	—	—	45	41	44	2.673	0.263
—	Publ_type	—	40	24	27	8.904	0.012^∗^
—	—	Article	—	41	9	15	42.946	<0.001^∗∗∗^
—	—	Preliminary comm.	26	0	0	58,366	<0.001^∗∗∗^
—	Eval_criter	—	42	39	44	5.56	0.062
—	—	Writing and present	—	36	24	34	5.751	0.056
—	—	Relevance	—	39	32	42	7.384	0.025^∗^
—	—	—	Originality	38	28	40	8.596	0.014^∗^
—	—	Ref_liter	—	35	25	33	3.448	0.178
—	—	Method and stat	—	25	14	18	3.591	0.166
—	PR outcome	—	40	32	38	2.226	0.329
—	—	Acceptance	—	37	19	30	11.182	0.004^∗∗^
—	—	—	Revision	20	29	27	6.806	0.033^∗^
—	—	Rejection	—	13	11	29	19.462	<0.001^∗∗∗^
PR ethics	—	—	—	21	21	30	6.018	0.049^∗^

Note: ^∗∗∗^<0.001, ^∗∗^<0.001, ^∗^<0.05.

The three countries show similarities in the distribution of “PR process” and “PR evaluation” first level categories. However, distribution starts to differ significantly with the second, third, and fourth level categories. Croatian and Spanish journals refer to the editorial board more often than Italian (*p* = 0.049), Spanish and Italian journals rely more on double anonymized peer review than Croatian ones (*p* = 0.001), and Spanish journals refer to reviewer independence (*p* < 0.001) and competences (*p* = 0.001) significantly more often than the other two countries.

The “PR evaluation” category shows that publication types “Article” (*p* < 0.001) and “Preliminary communication” are significantly more relevant to Croatian than to Spanish or Italian journals (*p* < 0.001 and *p* < 0.001, respectively). As for “Evaluation criteria”, the manuscript’s originality is more relevant to Spanish and Croatian than to Italian journals (*p* = 0.014). In terms of “PR outcome”, acceptance dominates in Croatian and Spanish journals (*p* = 0.004) and rejection in Spanish journals (*p* < 0.001).

Ethics too seems to be closer to the heart of Spanish law journals, research misconduct in particular (*p* = 0.049).

Between the documents in English (69) and those in national languages (non-English, 66), statistically significant differences were recorded in the “Article” (*p* = 0.001), “Rejection” (*p* = 0.002), and “Relevance” (*p* = 0.004) subcategories (Table 7).

**TABLE 7:** Distribution of categories identified in at least 50% of the documents by document language (English 69, non-English 66).

Category (1st level)	Category (2nd level)	Category (3rd level)	English	Non-English	Chi2	P (2-tails)
PR process	Governance	Edboard	53	53	0.244	0.621
PR process	ID_transp	Anonym	49	55	2.894	0.089
PR evaluation	Publ_type r	Article	44	24	10.134	0.001*
PR evaluation	Eval_criter	Writing and present	53	42	2.808	0.094
PR evaluation	Eval_criter	Relevance	64	49	8.474	0.004*
PR evaluation	Eval_criter	Ref_liter	51	42	1.662	0.197
PR evaluation	PR outcom	Acceptance	58	50	1.453	0.228
PR evaluation	PR outcome	Rejection	36	17	9.871	0.002*

Note: *<0.05.

## 4 Discussion

Everyone agrees that journal editorial policies should be accessible, clear, and transparent to authors, reviewers, and readers alike (Stojanovski and Marušić 2017). In its Principles of Transparency and Best Practice in Scholarly Publishing, COPE says the following:

“Journal content must be clearly marked as whether peer reviewed or not. Peer review is defined as obtaining advice on individual manuscripts from reviewers experts in the field who are not part of the journal’s editorial staff. This process, as well as any policies related to the journal’s peer review procedures, shall be clearly described on the journal website, including the method of peer review used.” (COPE 2013).

For research journals it is essential to provide evidence of editorial policies that foster the quality of published content (Sathyanarayana Rao and Tharyan, 2011). This pursuit of quality is furthered by open and transparent review, as transparency strengthens the process itself and improves the reputation of editors and reviewers (Pulverer 2010). Therefore, today’s editorial managing and peer review procedures should be more clearly defined, standardized, more transparent, and easy to find.

Finding information about peer review in law journals of the three countries was not that easy. It was scattered across different documents with different titles in different sections of journal websites. While most Croatian journals provide information on peer review in instructions to authors, Spanish journals provide it with journal description, and Italian journals provide it in separate documents specifically dedicated to peer review policy. Some journals have an additional online version, hosted by a repository or a publishing platform, which often organizes information about review transparency and editorial policy differently. Information given in English often reflects the desire to reach international audience and improve journal’s visibility and impact, even if the articles are published in the local language only. Journals that provide this information in local language alone, as many Italian journals do, show their national orientation.

Law journals in all three countries perceive anonymous (primarily double-blind) peer review as the necessary guarantee of the journal’s highest scientific quality. Despite increasing demands for open peer review as one of the core principles of open science, the editors of the law journals in our sample all prefer the blinded option. Similar to the Klebel et al., 2020 study of journals from different disciplines, a large percentage of law journals in our sample refers to anonymized peer review, but the percentage of documents declaring double-blind peer review is even (significantly) higher than in the Klebel study.

Some journals in our sample require authors to anonymize their manuscripts before submission and remove all identifying information, including references to the institution or project the manuscript is associated with, personal information in the file title or file properties, and bibliographic references which may identify the authors. Some journals even ask reviewers to contact the editorial board if they learn author identity by chance. We also noticed that some journals misinterpret double-anonymized peer review as the one in which two reviewers do not know each other’s identity. One journal in our sample misinterprets ‘open peer review’ as a single-anonymized peer review, because author identity is not hidden.

As a rule, law journals in our sample expect that reviewers are competent experts in the subject matter of the manuscript they are invited to evaluate, but not all invite independent/external reviewers. External review is more often requested by Spanish and Italian than Croatian journals. Also, only certain types of manuscripts are subject to peer review. Some law journals list types of manuscripts that are not sent to external review, and these are usually book reviews, doctoral dissertations, and conference papers. Instead, such manuscripts are accepted or rejected by the editor-in-chief and/or the editorial board. Such submissions have an additional time constraint of 2 years after the book publication date, conference date, or dissertation defense date, which seem unjustifiably long (who is interested in a conference paper after 2 years?). Surprisingly, some journals in our sample do not require peer review for papers authored by a professor emeritus or a recognized expert in a relevant position such as constitutional judge. Some law journals also seem to depart from their proclaimed peer review policies in practice.

Editors of smaller and less prestigious journals seem to rely more heavily on reviewers to categorize submissions by type, but the final decision remains with the editorial board. Such practice is common in Croatian journals. Once the paper is published, its category will be listed in the heading and table of contents. Decisions on manuscript type are not relevant only for journals but also for authors and their academic careers and could significantly impact journal metrics. Different types of papers are evaluated differently by academic institutions in terms of career advancement, which may be the main reason why editors ask reviewers to assist them in this matter.

Ethical issues are undoubtedly under-represented in documents describing the peer review process and editorial policies issued by Italian, Spanish, and Croatian law journals. Although it is essential to disclose possible competing interests that might prevent a reviewer from providing a fair and unbiased peer review, only one-third of the documents mention conflict of interests, and all other ethical concerns are even more neglected, although it is crucial for reviewers to remain unbiased towards author nationality, religious or political beliefs, gender, ethnicity, or geographical origin. Some journals pursue balanced gender representation in their editorial boards and reviewer pools. Low representation of research misconduct (e.g. fabrication or falsification of data) in policy documents may suggest that this is not an issue in legal scholarship as opposed to some other disciplines like biomedicine. This assumption, however, is yet to be verified or dismissed. Even so, editors of law journals should do more to check manuscripts for similarity and to preserve confidentiality.

There are several internationally prominent publishing and ethical guidelines created to improve the quality of scholarly publishing such as the EASE Guidelines for Authors and Translators of Scientific Articles to be published in English^5^ issued by the European Association of Science Editors, the Vancouver rules for authorship^6^ issued by the International Committee of Medical Journal Editors (ICMJE), and the Core practices^7^ issued by the Committee on Publication Ethics. Other noteworthy documents are the White Paper on Promoting Integrity in Scientific Journal Publications^8^ issued by the Council of Science Editors and the Wager and Kleinert position statement promoting international standards for authors^9^. Yet, law journals seldom refer to such guidelines.

Thanks to statistical analysis we have observed some significant differences in trends between the three countries. For example, Italian journals refer to the editorial board in their peer review documents far less often than the journals from the other two countries. One reason may be that peer review in these documents is addressed separately, while Croatian and Spanish journals tend to put both review and editorial roles together in the same document. We also noticed that, although journals in all three countries prefer traditional anonymized peer review, Croatian journals refer to author, reviewer, or editor anonymization significantly less often. Spanish journals also seem to hold reviewer independence and competence significantly more dear than Croatian journals.

Interesting differences were also observed in the “publication type” category. Croatian journals mention original scientific papers in their documents much more often than the journals from the other two countries. The difference is even more significant in preliminary communications, which Italian and Spanish journals do not mention at all in their documents. This may be owed to the journal subsidy evaluation criteria issued by the Croatian Ministry of Science and Education, which favor original scientific papers, review papers, and preliminary communications.

Croatian and Spanish journals insist on the originality of submitted manuscripts much more than Italian journals. Such manuscripts must not be previously published and must provide an original contribution to the field of law. The high prevalence of this term may arise from cultural distinctions, as what is termed an “original scientific work” in Croatia is simply termed a “research article” or simply an “article” in English-speaking countries.

Mentions of manuscript acceptance are significantly less frequent in Italian law journals, while mentions of manuscript rejection are significantly more frequent in Spanish journals. Spanish journals also refer to research misconduct, including fabrication, falsification, and manipulation of data and scientific fraud significantly more often than Croatian and Italian journals.

Although we found statistically significant differences for three categories (“Article”, “Rejection”, and “Relevance”) between documents in English and local languages, this does not mean that internationally oriented journals (those which publish their editorial policies in English), have adopted more advanced and globally accepted standards and peer review policies than local journals. Finally, the most important question is how to improve peer review in law journals. Some progressive ideas and experiments have already been described in the cited literature. Peer review can also be improved through contributions of editors to discussions about publishing and ethical standards, standardization of the peer review process in particular. A step further would be to make peer review open (unblinded and/or public), to incentivize and reward reviewers, to train editors and reviewers, and to provide a platform for efficient communication between authors, reviewers, and editors.

## 5 Conclusion

In legal science, peer review is considered the guardian of publication quality, or at least “the worst way to assess research, except for all the others” (Carrol, 2018). Our study shows that peer review in law journals is complex, even in its traditional form. As we were building our categorization dictionary starting with the three main concepts of peer review (process, evaluation, and ethical issues), we came up with more than seventy categories and subcategories (down to the fifth hierarchical level) containing several hundred keywords.

Our general finding is that law journals in our sample prefer double-blind peer review managed by the editorial board and require a high level of expertise from reviewers. Traditional approach is also characteristic for all other elements and there is little room for innovative approaches. Criticizing anonymous peer review in great many scholarly journals from all disciplines, the editor of the British Medical Journal once argued that “a court with an unidentified judge makes us think immediately of totalitarian states and the world of Franz Kafka” (Smith 1999). While we are not inclined to liken reviewers to judges, we firmly believe that adopting transparency and openness, especially in terms of peer review, can significantly improve the journal quality and advance legal scholarship in general. Assessing someone’s work behind the curtain of anonymity could be considered deeply unethical.

We were not surprised by the great variety of genres (article types), as legal scholars publish both peer-reviewed scholarly papers and several shorter and less formal types of articles specific to the field of law that are valued by their colleagues, but in view of this variety we were somewhat surprised to find that most law journals value manuscripts by relevance (originality), writing and presentation, and exhaustive and accurate referencing.

In terms of publication ethics, most law journals address conflict of interests, while other aspects such as transparency, plagiarism, best practices, bias, and research integrity are less represented and deserve more attention in future research. Law journals have yet to tackle more complex ethical issues specific to different areas of law. The most advanced in that respect are the Spanish journals.

Future research should also address other issues raised by our findings, such as the lack of diversity in editorial procedures across countries and reluctance to adopt innovative approaches to peer review. In fact, our findings could serve as an incentive to law journals to make their editorial policies more transparent and comprehensive. Future research could also compare actual review practices with the declared ones through qualitative evaluation of various stakeholders in the editorial process, including editorial boards, managing editors, publishers, authors, and reviewers.

With the multilingual peer review taxonomy developed for this research, it is also possible to include journals/documents from other countries and languages and increase the corpus to obtain more reliable and statistically more relevant analysis.

## 6 Limitations

Our analysis is limited to publicly available documents and therefore does not provide a more comprehensive picture that would include some closed commercial law journals and their practices. Furthermore, it is possible that some journals included in this study have restricted access to more detailed information on peer review (such as instructions to reviewers) to reviewers who have accepted their invitation to review submissions. Some journals provide instructions to reviewers and peer review policy with evaluation criteria only through their editorial management systems, and these documents are not publicly available on journal websites.

Another limitation may be the design of our categorization dictionary. Is it intuitive and comprehensive enough? We could not find any validated instruments for our study besides the STM Standard Taxonomy on Peer Review, which is focused on four elements of the process (i.e. how transparent is the identity of those involved, with whom does the reviewer interact, what information about the review process is published, and whether post-publication commenting takes place) and on peer review evaluation criteria published by (Bornmann et al., 2008; Bornmann et al., 2011). Although our categorization dictionary has not been validated externally, all authors of this study find its content validity evident. Some words with ambiguous meanings were excluded from the taxonomy or were replaced with phrases or rules to reduce the noise. This may have limited the coverage and yielded lower return frequencies. Some frequencies could also be higher, like when we looked for “methods” as an evaluation criterion and got mismatched hits like “metodo doble ciego”, “metodo de citacion” or “metodo di selezione degli articoli” in addition to correct matches like “materia y metodo” and “metodo obiettivo ed analitico”. Despite these limitations of software-assisted coding, our results give a pretty good insight into the peer review procedures of the selected law journals. In addition, the multilingual categorization dictionary created for this study is a valuable resource that could serve further studies in this area.

Another limitation could be that we used the number of mentions, documents, and shares in the total sample as a proxy for keyword relevance, which could be an imperfect solution for our corpus consisting of a small number of relatively short documents and could have affected our statistical analysis. Our future research will therefore expand to law journals from other countries.

## Data Availability

The original contributions presented in the study are included in the article/Supplementary Material, further inquiries can be directed to the corresponding author.

## Footnotes

1 SCImago (n.d.). SJR—SCImago Journal and Country Rank (Portal). Retrieved August 13, 2021 from http://www.scimagojr.com.

2
https://hrcak.srce.hr/?lang=en.

3
https://www.anvur.it/en/.

4
https://calidadrevistas.fecyt.es/.

5
https://ease.org.uk/publications/author-guidelines-authors-and-translators/.

6
http://www.icmje.org/recommendations/browse/roles-and-responsibilities/defining-the-role-of-authors-and-contributors.html.

7
https://publicationethics.org/core-practices.

8
https://www.councilscienceeditors.org/resource-library/editorial-policies/white-paper-on-publication-ethics/.

9
http://publicationethics.org/files/International%20standards_authors_for%20website_11_Nov_2011.pdf.

## Supplementary Material

Supplementary Table S1 List of journals and collected documents with necessary metadata new version is uploaded.

Supplementary Table S2 Wordstat8 data at category and country level.

Supplementary Table S3 Categorization dictionary (Excel spreadsheet).

Supplementary Data sheet S1 Categorization dictionary (CSV).

Supplementary Data sheet S2 All collected documents on editorial policies related to the peer review process.
