# Supplementary material for: Peer Review in Law Journals
Source: Front Res Metr Anal. 2021 Dec 8;6:787768. doi: 10.3389/frma.2021.787768 (PMC8692876; doi:10.3389/frma.2021.787768)
Supplement: Supplementary file 3 [file DataSheet2.ZIP › DOCUMENT - 0008-7750_1.RTF]

About the Journal
Focus and Scope
Anales de la Cátedra Francisco Suárez Chair (ACFS) is a journal on legal and political philosophy, edited by the Department of Philosophy of Law of the University of Granada and by the Editorial Universidad de Granada.
ACFS has been published since 1961 and our intent is to be a means of communication in the field of legal and political philosophy, an instrument in which the conclusions of the philosophical reflection on the problems posed by the political organization of the coexistence of individuals, especially the problems raised by the use of law as an instrument for that organization.
The language of the magazine is Spanish and it is published annually. Each issue has a monographic section, an open section, and a bibliographical critique section.
Anales de la Cátedra Francisco Suárez Chair is indexed in
CARHUS PLUS
CIRC
ERIHPLUS
Latindex
Philosophers Index
Periodicals Index Online
EBSCO
REDIB
ANVUR
Anales de la Cátedra Francisco Suárez has obtained the FECYT Quality Seal in the VI Call for the Evaluation of Editorial and Scientific Quality of Spanish Scientific Journals.
 
Open Access Policy
This journal  provides immediate free access to its content under the principle that making research freely available to the public, which encourages greater global knowledge sharing.
Our magazine adheres to the recommendations for the implementation of article 37 Dissemination in Open Access of the Law of Science, Technology and Innovation:
Authors whose contributions are accepted for publication in this journal will retain the non-exclusive right to use their contributions for academic, research and educational purposes, including self-archiving or deposit in open-access repositories of any kind.
Preferably the use of the published version of the scientific contributions (post-print version of the editor) or, failing that, of the post-print version of the author already evaluated and accepted, which will be accessible in open as soon as it is, will be allowed possible, since the magazine does not impose any period of embargo.
Publication costs of the articles: this journal does not charge the authors any cost for the presentation or processing of the articles.
Ethical Standards and Best Practices
Anales de la Cátedra Francisco Suárez adheres to and abides by the guidelines of the EASE (European Association of Science Editors) and of the COPE (Committee on Publications Ethics), which guide good practices for the management, edition, review and publication of scientific results in journals of Different areas of knowledge.
Briefly, what is established there implies:
(a)   For authors. Submitting a paper to Anales de la Cátedra Francisco Suárez implies the author’s acceptance of this Journal’s rules for publication, revision, and evaluation. Authors pledge to submit only original manuscripts that had not been published earlier and that are not currently subject to any other journal’s review process. They also agree not to submit them for evaluation in another journal while they are in process in this one. Where portions of the content overlap with published or submitted content, authors will acknowledge and cite those sources, as well as, if necessary, they will obtain permissions. Additionally, if require, authors will provide the editor with a copy of any submitted manuscript that might contain overlapping or closely related content. Authors will also need: To declare any potential conflicts of interest (e.g. where the author has a competing interest (real or apparent) that could be considered or viewed as exerting an undue influence on his or her duties at any stage during the publication process); To notify promptly the journal editor or publisher if a significant error in their publication is identified; To cooperate with the editor and publisher to publish an erratum, addendum, corrigendum notice, or to retract the paper, where this is deemed necessary. On the other hand Anales de la Cátedra Francisco Suárez will inform authors of papers in a prompt and timely manner whether their work is accepted; will ensure that any copy-editing is respectful of authors’ style and ideas; and will give authors adequate time to see and correct the final proofs.
(b)   For Reviewers assume the burden of carrying out, within the confines of their skills and knowledge, a critical, honest, and constructive review regarding the scientific quality of the manuscripts entrusted to them. This is the reason why each reviewer will accept to assess a manuscript only if they consider themselves sufficiently competent and if there are no conflicts of interest. Peer reviewers ought to maintain the confidentiality of any information supplied by the editor or author and not to retain or copy the manuscript. They also assume the responsibility to alert the editor of any published or submitted content that is substantially similar to that under review. Finally, peer reviewers committed to blind review need to be aware of any potential conflicts of interest (financial, institutional, collaborative or other relationships between the reviewer and author) and to alert the editor to these, if necessary withdrawing their services for that manuscript.
(c)   For the Director, Editor, the members of the Editorial Board, and members of the Advisory Board: They pledge to be impartial and to maintain the confidentiality of the manuscripts submitted to Anales de la Cátedra Francisco Suárez, of their authors, and of the designated reviewers. All of these is done in order to let the principle of anonymity to preserve the integrity of the whole assessment process. To that effect, the selection of the most qualified reviewers and specialists will be guaranteed. The editorial team pledges to avoid any kind of conflict of interest and to strictly observe the assessment, edition, and publication deadlines that are consistent with the Journal’s periodicity.
Plagiarism control. Best Practices for the editorial team would include the following actions:
–      Encouraging reviewers to comment on the originality of the submitted papers and to be alert to possible repeated publications and plagiarism;
–      Adopting plagiarism detection systems (eg, software, search for similar titles) for submitted articles (either routinely or when doubts arise about a particular document);
–      Supporting authors whose copyrights have been violated or who have been victims of plagiarism;
Being prepared to work together with the editorial group to defend the rights of authors and prosecute offenders (for example, requesting retractions or removal of material from websites) regardless of whether the journal owns the copyright.
 
