# Supplementary material for: Peer Review in Law Journals
Source: Front Res Metr Anal. 2021 Dec 8;6:787768. doi: 10.3389/frma.2021.787768 (PMC8692876; doi:10.3389/frma.2021.787768)
Supplement: Supplementary file 3 [file DataSheet2.ZIP › DOCUMENT - 0008-7750_2.RTF]

Submissions
Login or Register to make a submission.
Submission Preparation Checklist
As part of the submission process, authors are required to check off their submission's compliance with all of the following items, and submissions may be returned to authors that do not adhere to these guidelines.
`.	The paper has not been previously published, nor has it been submitted to another journal.
`.	 The file sent is in OpenOffice, Microsoft Word, RTF, or WordPerfect format.
`.	Web sites have been added as references when available.
`.	The text formalities follow the rules especified in Authors' Guidelines.
`.	The text meets the bibliographic and style requirements indicated in the Norms for authors, which can be found in About the journal.
`.	If you are sending to a section of the jounal that is peer reviewed, you have to make sure that the instructions in the Normas para autores`.	.
Author Guidelines
Authors registration and paper subsmission
To submit an original you must be registered as an author on the website of the Magazine. Registration is done at https://revistaseug.ugr.es/index.php/acfs/login Exceptionally, originals can be sent by email to filode@ugr.es or apena@ugr.es
 
Extension
The extension should not exceed 10,000 words (approximately 20 DIN-A4 pages, written in space and a half in Times New Roman 12) for articles in the monographic and open sections. Bibliographic reviews must not exceed 3,500 words.
 
Title, summary, keywords, and authors' data
The first page will include, centered, the title in Spanish and English, name of the author, academic affiliation, country, professional address of ordinary mail and electronic mail. Articles should include a 100-150 word summary written in Spanish and English. They must also include between 5 and 10 keywords in Spanish and English.
The reviews will have a title, in Spanish and English, related to their content and must refer to the work reviewed as follows: AUTHOR, title, editorial, place, year, number of pages.
All authors must attach some brief curricular lines (maximum 150 words) that, in addition to recording their institutional affiliation, account for their main publications and reflect the thematic areas cultivated.
Latin characters (Roman script) and academically appropriate language should be used.
 
Notes
They will be placed at the bottom of the page, numbered by Arabic characters and in superscript format. They will only contain additional text and bibliographic citations. In no case will they include complete bibliographical references, which will be included at the end of the article.
 
Quotations
Quotation system of all originals, including those in the bibliographical criticism section, must conform to the guidelines of the American Psychological Association. More information at https://apastyle.apa.org/style-grammar-guidelines/citations/. To facilitate indexing, citations must be in Latin characters (Roman Script).
Examples:
Quotations from books and articles in the text will appear in the body of the text. They will be indicated in parentheses the author's last name in lowercase, followed by the year and page of the publication.
(Pitch, 1998, p. 31)
If two or more documents have the same author and year, they will be distinguished from each other with lowercase letters after the year and within the parentheses.
(Bobbio, 1990a)
Documents with two authors will be cited by their first surnames linked by “and”. (Nussbaum and Sunstein, 2004)
For documents with more than two authors the quotation will be abbreviated indicating only the last name of the first author followed by “et al.”.
(Koselleck et al., 1972)
When the last name of the cited author is part of the text of the work, the year and page (s) of the cited work must always be indicated in parentheses:
As Zambrano (1958, p. 12) states, ...
 
Bibliographic references
Bibliographic references will be included at the end of the articles. Only the works, sources or documents cited in the text will be referred to. References will be sorted alphabetically by the author's first name following the instructions of the American Psychological Association. More information at https://apastyle.apa.org/style-grammar-guidelines/references/
References to cities will be made in the language of the text of the body of the article, that is, in Spanish if there is a common term of common use (eg Milán but not Milano, London but not London, Gerona but not Girona). The names of publishers, universities and other institutions must appear in their original form (New York University, London School of Economics, Universitat de Girona, and so). To facilitate indexing, citations and references must be in Latin characters (Roman Script).
If two or more works by an author published in the same year are cited, they will be distinguished by means of a letter:
Bobbio, N. (1990a). Saggi his Gramsci. Milán: Feltrinelli.
Bobbio, N. (1990b). L’età dei diritti. Turín: Einaudi.
If a reissued work is cited and the date of the first edition is considered relevant, it will be indicated in square brackets:
Díaz, E. (2010) [1966]: Rule of law and democratic society. Madrid: Taurus.
Below are examples for the most used types of documents:
Books
—One author
Pitch, T. (1998). A disciple per due. Milán: il Saggiatore.
—Two or more authors
Nussbaum, M. and Sunstein, C. (2004). Animal Rights. Oxford: Oxford University Press. Koselleck, R., Brunner, O., Conze, W. (1972). Geschichtliche Grundbegriff. Stuttgart: Klett-Cotta.
Book Chapters
Resta, E. (2000). The unconfessable community and fraternal law. In Silveira Gorski (comp.). Community identities and democracy (pp. 211-234). Madrid: Trotta.
Articles from academic journals
Puigpelat, F. (1986). On the science of law and rational discourse. Yearbook of Philosophy of Law, 3, 229-248. http: //dx.doi. org / 10.7203 / CEFD.34.8927
Reports
Amnesty International (2017). First, do no harm: ensuring the rights of children with variations of sex characteristics in Denmark and Germany. Londres.
Thesis
Escudero Alday, R. (1999). Sistema jurídico y justicia: sobre la relevancia moral de la estructura jurídica [doctoral thesis]. Universidad Carlos III de Madrid. Available at: http: //hdl.handle. net / 10016/11184
Rubio Castro, A. (1984). La filosofía jurídica académica en España (1900-1936) [unpublished doctoral thesis]. Universidad de Granada.
 
Anonymization of originals and evaluation reports
To ensure the integrity of the review process of the originals and the anonymity of authors and reviewers, it is necessary to avoid that the characteristics or properties of the texts sent allow knowing the identity of their authors. Hence:
- Authors and reviewers will omit any reference to their identity in their texts.
- Authors should substitute their names and the names of their publications in the citations and references, using the formula "Author, year" instead of their own name or the name of their publications.
- In Microsoft Office documents, the identity of the author must be deleted from the document properties (see File in Word), proceeding as follows: in File, in the main Office menu, File> Save As> Tools (or Options on Mac) > Security> Delete personal information from file when saving> Save.
- In PDFs, authors must also delete their names from the document properties, in the file menu in the Adobe Acrobat main menu.
 
Admission
The Editorial Board will decide on the admission of the originals when the reception period ends and will notify each author (a) if the submitted paper has been accepted and goes to evaluation and on the admission modality, (b) if it is pending or (c) if it is not admitted and the reason why it is not. However, articles that obey a personal invitation to publish from the Director or Editor may be admitted before that date.
Among others, the following will be criteria of inadmissibility of submissions: (a) that the content of the paper cannot be presumed of interest to an academic or scholarly audience, (b) that the text does not affect the field of knowledge cultivated by the journal, (c) significantly exceeding or not reaching the indicated length (d) that the text has serious formal defects and (e) that the text is poorly written or in an academically inappropriate language.
 
Evaluation
All the originals of the monographic and open sections will be informed by two evaluators who will not know the identity of the author (peer review, double blind).
Evaluators will be selected by the Editor, who will preferably choose experts not belonging to the Editorial Team. At least 50% of the reviewers will be external to the Editorial Team of the Journal.
The evaluators will respond to the evaluation form that will be provided and will assess (a) if the original can be published without modifications, (b) if its publication is conditioned to the introduction of revisions or corrections in the text or (c) if it is not publishable and the reasons why it is not. In case of discrepancy between the reviewers, that is, if there is a negative and a positive report (conditioned or not), a third evaluator will be asked. Unless expressly indicated by the evaluators, their identity will not be known by the author.
Once the evaluation is known, the author must inform the editor if the text will be revised in the sense expressed in the evaluation reports or, if not, if its publication is waived. In the first case, the author must review the text within the period indicated by the editor and send a final definitive corrected version, without any annotation or comment and, together with it, a separate document that explains how the referees’ observations have been satisfied in the text. The editor will assess whether the reported observations have been properly satisfied, with the assistance of the evaluators if necessary or if it had been expressly requested by them.
 
Copyright Notice
Authors are the owners of the rights to their works. ACFS requests that publication notice on ACFS is disclosed if they appear later in another place.
