# Supplementary material for: Peer Review in Law Journals
Source: Front Res Metr Anal. 2021 Dec 8;6:787768. doi: 10.3389/frma.2021.787768 (PMC8692876; doi:10.3389/frma.2021.787768)
Supplement: Supplementary file 3 [file DataSheet2.ZIP › DOCUMENT - 0012-3447.RTF]

I contributi pubblicati nella sezione dottrina e le note a sentenza sono stati sottoposti con esito positivo alla revisione anonima da parte di un professore ordinario di diritto tributario
