# Supplementary material for: Peer Review in Law Journals
Source: Front Res Metr Anal. 2021 Dec 8;6:787768. doi: 10.3389/frma.2021.787768 (PMC8692876; doi:10.3389/frma.2021.787768)
Supplement: Supplementary file 3 [file DataSheet2.ZIP › DOCUMENT - 0012-348X.RTF]

PROCEDURA DI REFERAGGIO  PER GLI ARTICOLI E LE NOTE
Questa Rivista ha adottato la procedura di revisione degli articoli e delle note nel 2011, applicando il programma di revisione che segue.
Dopo aver proceduto ad un esame preliminare di ogni scritto pervenuto e alla sua classificazione in una delle rubriche della Rivista (Dottrina, Note a sentenza e Cronache e note) i Direttori della Rivista, nel caso di un suo esito positivo:
a)  Se lo scritto è destinato alla pubblicazione nella rubrica “Dottrina” lo trasmettono alla valutazione, con il sistema del "doppio cieco", a due revisori scelti tra noti ed autorevoli esperti appartenenti ad Università italiane o straniere, con conoscenza dell'argomento, assicurando l'anonimato sia degli Autori che degli esperti. I due revisori esprimono la loro valutazione sulla base dei seguenti criteri:
- chiarezza della esposizione;
- correttezza dell’impostazione metodologica;
- contributo apportato allo studio dell’argomento;
- adeguatezza della bibliografia essenziale e dell’analisi della giurisprudenza.
b)  Se si tratta di "Nota a sentenza", i Direttori della Rivista valutano, congiuntamente ad un revisore scelto con le modalità in precedenza indicate se la nota possieda o meno i requisiti per la sua pubblicazione, utilizzando i seguenti criteri:
- individuazione nella nota del punto e dei punti della decisione che l’autore intende analizzare;
- eventuale inquadramento di ciascuno di tali punti nell’ambito di uno specifico istituto;
- se la decisione è (o avrebbe dovuto essere) basata su una convenzione internazionale, ricerca e sintesi dei precedenti estesa anche alla giurisprudenza di altri paesi contraenti;
- concisa esposizione delle (eventuali) opinioni della dottrina.
c)  Se lo scritto appare pubblicabile nella rubrica “Cronache e note” i Direttori della Rivista valutano, congiuntamente ad un revisore scelto con le modalità in precedenza indicate, se lo scritto possieda o meno i requisiti per la sua pubblicazione, utilizzando in linea di massima i seguenti criteri:
- opportunità di scelta dell’argomento, che deve presentare un interesse nel momento in cui avviene la sua pubblicazione, come appare dalla parola “cronache”;
- adeguatezza della sua trattazione e chiarezza della esposizione.
Sulla base della valutazione, compiuta seguendo i criteri sopra esposti, i Direttori della Rivista possono accettare o meno lo scritto per la sua pubblicazione.
Giorgio Berlingieri - Stefano Zunarelli
