# Supplementary material for: Peer Review in Law Journals
Source: Front Res Metr Anal. 2021 Dec 8;6:787768. doi: 10.3389/frma.2021.787768 (PMC8692876; doi:10.3389/frma.2021.787768)
Supplement: Supplementary file 3 [file DataSheet2.ZIP › DOCUMENT - 0013-9491.RTF]

Ephemerides Iuris Canonici è un luogo di approfondimento scientifico e dibattito critico sui punti di maggior interesse per la comunità canonistica internazionale, nello stile che da sempre qualifica il periodico. Ogni suo numero sviluppa un tema di carattere monografico; inoltre raccoglie contributi di rilievo nell’ambito del diritto canonico ed ecclesiastico. Tutti gli articoli pubblicati nella rivista sono preventivamente sottoposti alla valutazione di referee esterni, secondo la procedura di peer review.
