# Supplementary material for: Peer Review in Law Journals
Source: Front Res Metr Anal. 2021 Dec 8;6:787768. doi: 10.3389/frma.2021.787768 (PMC8692876; doi:10.3389/frma.2021.787768)
Supplement: Supplementary file 3 [file DataSheet2.ZIP › DOCUMENT - 0015-783X.RTF]

LA STORIA DEL DIRITTO ITALIANO DAL 1876
Dalla sua nascita a Roma il Foro Italiano è il più prestigioso strumento di studio e di lavoro per ogni professionista del settore giuridico. Nel corso della sua vita, ogni singolo numero ha scandito le evoluzioni del diritto e del costume italiano, offrendo sicurezza e credibilità alla pratica quotidiana di giuristi, magistrati, avvocati.
LA CONTINUITÀ E LA TRADIZIONE
La prima direzione della rivista annoverava, tra i suoi tre membri, Enrico Scialoja. Per gli oltre 140 anni a seguire, vi sarà sempre uno Scialoja alla guida della rivista, un passaggio di testimone che trova corrispondenza nei suoi fedeli abbonati: di generazione in generazione, i ‘fascicoli gialli’ del Foro sono la staffetta costante negli studi più prestigiosi, nelle aule delle università e di tutti i tribunali d’Italia.
UN’OPERA CORALE
Una pluralità di voci composta da un’agguerrita redazione e da uno stuolo di grandi giuristi che hanno contribuito e contribuiscono in maniera determinante alla realizzazione di un’impronta moderna, capace di coniugare rigore e tempestività, indipendenza e trasparenza. Un’opera corale, che dialoga costantemente con il mondo dell’accademia e con l’universo professionale.
INSIEME, NEL FUTURO
Nel 2017 l’incontro tra Il Foro Italiano e La Tribuna: due eccellenze nel settore dell’editoria giuridico-legale uniscono le forze con l’obiettivo di creare un polo di matrice tutta italiana che possa confrontarsi ad armi pari con i colossi internazionali del settore, porre le basi per un’espansione all’estero, perseguire un progetto di modernizzazione e innovazione al servizio di ogni professionista: formazione e informazione tempestive, affidabili, autorevoli.
L’ATTUALE COMPAGINE SOCIALE
La sua compagine sociale è attualmente così composta: Marco Annecchino — Antonella Capone — Daniela Carbone — Francesco Di Ciommo — Massimo Fabiani — Onofrio Fanelli — Maurizio Ferrari — Giovanni Fiandaca — Alessandro Iacoboni — IP2tech Srl — La Tribuna Srl — Giorgio Lener — Raffaele Lener — Francesco Macario — Livia Magrone Furlotti — Maurizio Paganelli — Roberto Pardolesi — Domenico Piombo — Michele Scialoja — Massimiliano Silvetti — Vincenzo Sinisi — Antonio Tizzano.
COMITATO DI REFERAGGIO
Il Foro italiano seleziona i contributi (note, articoli ecc.) destinati alla pubblicazione. Quelli che superano il vaglio dei responsabili d’area e sono caratterizzati da una dimensione consistente (eccedente i 20.000 caratteri) vengono sottoposti a referaggio anonimo sui due versanti (‘doppio cieco’). La procedura di affidamento e compilazione dei referaggi è automatizzata e viene attuata nella parte riservata di questo sito, cui il valutatore indipendente accede con apposite credenziali.

Il comitato di riferaggio è composto da autorevoli esperti delle materie coperte. Di seguito si riporta la composizione del comitato di referaggio come consolidata al 16 aprile 2020: 
Niccolò Abriani — Roberto Adam — Enrico Altieri — Chiara Amalfitano — Cristina Amato — Giuliano Amato — Ferruccio Auletta — Gaetano Azzariti — Laura Baccaglini — Stefano Bastianon — Stefano Battini — Giuseppe Bellantuono — Elisabetta Bergamini — Mirzia Bianca — Stefano Bielli — Emanuele Boscolo — Giuseppe Bronzini — Ilaria Amelia Caggiano — Daniela Calafiore — Alfredo Calderale — Ermanno Calzolaio — Albina Candian — Giovanni Canzio — Remo Caponi — Paolo Carbone — Sergio M. Carbone — Francesco Caringella — Antonio Carratta — Giuseppe Carriero — Roberto Caso — Marina Castellaneta — Rossella Catena — Stefano A. Cerrato — Mario Cicala — Fabio Cintioli — Giuseppe Colangelo — Giovanni Comandè — Massimo Condinanzi — Guido Corso — Cristina Costantini — Giorgio Costantino — Pasquale Costanzo — Antonio Cucinotta — Valentina V. Cuocci — Piero Curzio — Giovanni D'Amico — Marilisa D'Amico — Angelo Danilo De Santis — Camilla Di Iasi — Annalisa Di Paolantonio — Angela Di Stasi — Gianluca Faella — Susanna Fortunato — Fabrizio Fracchia — Pietro Franzina — Massimo Franzoni — Beatrice Gambineri — Lucilla Gatt — Gianvito Giannelli — Gina Gioia — Stefano Giubboni — Giuseppe Grisi — Attilio Guarneri — Antonio Iannarelli — Gianpaolo Impagnatiello — Silvia Izzo — Umberto Izzo — Gioacchino La Rocca — Enrica Laterza — Ferdinando Lignola — Elisabetta Loffredo — Alessandro Lolli — Daniela Longo — Massimo Luciani — Raffaello Lupi — Francesco Macario — Elena Malfatti — Anna Maria Mancaleoni — Antonio Manna — Roberto Martino — Carlo Marzuoli — Roberto Mastroianni — Giuseppe Melis — Grazia Miccoli — Olga Mignolo — Massimo Miola — Antonello Miranda — P.G. Monateri — Arnaldo Morace Pinelli — Pasquale Nappi — Bruno Nascimbene — Luca Nivarra — Massimo Occhiena — Alessio Pacces — Lorenzo F. Pace — Stefano Pagliantini — Ilaria Pagni — Paolo Pardolesi — Roberto Pardolesi — Giovanni Pascuzzi — Paolo Passaglia — Francesco P. Patti — Salvatore Patti — Rita Perez — Barbara Pezzini — Antonietta Picardi — Valentina Piccone — Giorgio Pino — Fabrizio Piraino — Giorgio Giuseppe Poli — Barbara Poliseno — Oreste Pollicino — Giulio Ponzanelli — Gaetano Presti — Roberto Pucella — Andrea Pugiotto — Margherita Ramajoli — Gian Michele Roberti — Marco Rossetti — Lucia S. Rossi — Antonio Ruggeri — Giovanni Sala — Cesare Salvi — Rita Sanlorenzo — Bruno Sassani — Nicola Scannicchio — Enrico Scoditti — Claudio Scognamiglio — Irene Scordamaglia — Pietro Selicato — Mario Serio — Marco Sica — Guido Smorto — Alessandro Somma — Domenico Sorace — Antonino Spadaro — Sara Spuntarelli — Stefania Stefanelli — Giovanni Tarli Barbieri — Marina Tavassi — Giuseppe Tesauro — Giacomo Travaglino — Bruno Tridico — Debora Tripiccione — Onofrio Troiano — Alessandrina Tudino — Tommaso Ubertazzi — Daniele Vattermoli — Francesco Vella — Michele Vellano — Sergio Vinciguerra — Umberto Violante. 
