# Supplementary material for: Peer Review in Law Journals
Source: Front Res Metr Anal. 2021 Dec 8;6:787768. doi: 10.3389/frma.2021.787768 (PMC8692876; doi:10.3389/frma.2021.787768)
Supplement: Supplementary file 3 [file DataSheet2.ZIP › DOCUMENT - 0021-325X_1.RTF]

Quiénes somos
`.	» Contacto
`.	» Equipo editorial
`.	» Consejo editorial
`.	» Consejo asesor
Información específica
Enfoque y alcance 
Política de secciones 
Estudios 
Comentarios 
Nuevas voces del DGDC 
Crónicas 
Política de acceso abierto 
Política antiplagio 
Política de preservación 
Tasas de publicación 
Frecuencia de publicación 
Prepublicación de artículos 
Proceso de evaluación por pares 
1. Los artículos se someterán a doble revisión anónima por expertos ajenos al Consejo Editorial.

2. Se garantiza la confidencialidad de los autores y de los revisores.

3. Periódicamente se dará a conocer la lista de revisores de Ius Canonicum, sin indicación de los trabajos revisados.
Instrucciones a los evaluadores 
El sistema de doble evaluación anónima e independiente por expertos (proceso de doble-ciego) es un referente central de las revistas de mayor calidad científica. El Consejo de Redacción tomará muy en cuenta sus observaciones antes de tomar una decisión sobre el escrito que le hemos enviado. Si considera que hay alguna razón que le impida una valoración anónima, independiente y equitativa del artículo, no dude en señalárnoslo.

Garantizamos la confidencialidad del autor y los evaluadores. El escrito que sometemos a su valoración es un documento privado: le rogamos que no lo muestre a terceros, ni exponga a otros su contenido a menos que se publique. Su evaluación será tratada igualmente de manera reservada: solo trasmitiremos al autor las observaciones que la revista considere más importantes para mejorar su manuscrito, sin revelarle la identidad del evaluador. En caso de que parte de sus comentarios vayan dirigidos solo al Consejo de Redacción y no al autor evaluado, indíquelo así.

Para que el proceso editorial sea ágil y no hacer esperar excesivamente a los autores, le rogamos que responda al cuestionario en un plazo lo más breve posible.
Informe de evaluación 
En el siguiente enlace encontrará el formulario que se les envía a los evaluadores para que elaboren el informe de revisión.
Listado de evaluadores anteriores 
Presencia en bases de datos, directorios de prestigio y repositorios 
Exención de responsabilidad 
Declaración de buenas prácticas 
Datos editoriales y tirada 
Pedidos y suscripciones 
Precios 
Modos de pago: 
 
Envío de manuscritos
`.	» Normas editoriales
`.	» Nota de copyright
`.	» Política de privacidad
