# Supplementary material for: Peer Review in Law Journals
Source: Front Res Metr Anal. 2021 Dec 8;6:787768. doi: 10.3389/frma.2021.787768 (PMC8692876; doi:10.3389/frma.2021.787768)
Supplement: Supplementary file 3 [file DataSheet2.ZIP › DOCUMENT - 0034-7639_1.RTF]

Temas de interés
La Revista de Administración Pública (RAP) publica trabajos de investigación originales sobre Derecho Administrativo y Administración Pública.
 
Envío de originales
Los trabajos deberán ser originales e inéditos; en caso de ser publicados una vez enviados a esta Secretaría, los autores deberán notificarlo y proceder a la retirada de los mismos. Se enviarán en español, escritos en Microsoft Word o en formato compatible, y se harán llegar por correo electrónico a la dirección evaluaciones@cepc.es, consignando en el asunto del mensaje que se trata de un artículo destinado a la RAP. El Consejo de Redacción solo tomará en consideración aquellos trabajos que se hayan enviado directamente a la revista, a través del medio indicado. Será obligatorio enviar dos versiones: una anonimizada para faciltar la evaluación anónima del texto, y otra sin anonimizar.
 
Formato
Los originales deberán ir escritos a espacio y medio, en letra Times New Roman tamaño 12. La extensión total no debiera superar las 12 000 palabras, incluidas las notas a pie de página y los apéndices en su caso. La primera página incluirá el título, nombre del autor o autores, filiación académica, direcciones de correo ordinario y electrónico y teléfonos de contacto. En una segunda página se presentarán dos resúmenes, en español e inglés, de unas 120 palabras cada uno y un máximo de cinco palabras clave, también en los dos idiomas.
Las recensiones no podrán superar las cinco páginas.
 
Proceso de publicación
La RAP decidirá, a través de su Consejo de Redacción, la publicación de los trabajos sobre la base de dos informes de evaluación emitidos por sendos especialistas ajenos a la organización editorial de la revista, aplicándose el método de doble ciego. Los autores de artículos aceptados para publicación podrán ser requeridos para la corrección de pruebas de imprenta, que habrán de ser devueltas en el plazo máximo de cinco días. No se permitirá la introducción de cambios sustanciales en las pruebas, quedando estos limitados a la corrección de errores con respecto a la versión aceptada.
 
Normas de citación
Citas y referencias bibliográficas
Aparecerán en el área de notas, que se situará a pie de página, y siempre en su forma completa si es la primera vez que aparece la referencia, mientras que se hará de forma abreviada si ya ha aparecido con anterioridad. No se elaborará bibliografía final.
Notas
Se situarán a pie de página, numeradas mediante caracteres arábigos y en formato superíndice. Contendrán texto adicional y las citas y referencias bibliográficas en la forma indicada en el apartado anterior.
Normas comunes de citación en el área de notas
— Inicial del nombre abreviado + apellido (s) + (año).
— Si son dos autores, separados por «y»:
   C. Alchourrón y E. Bulygin (1981)
— Si son más de dos autores, separados por coma y el último por «y»:
   A. López, M. Alonso y E. Carmona (2010)
— Usar «véase» en vez de «vid.»
 
Tipología documental
Artículos de revista
Véase M. García Pérez y A. Salazar López (2004), «El deslinde de las costas», Anuario de la Facultad de Derecho de la Universidad de La Coruña, 8, págs. 391-422.
 
F. Sospedra Navas (2004), «Los conflictos entre el dominio marítimo terrestre y la propiedad privada», Revista del Poder Judicial, 76, págs. 247-282.
 
V. Magro Servet (2002), «Casuística sobre el concepto penal de domicilio en la diligencia de entrada y registro», La Ley: Revista jurídica española de doctrina, jurisprudencia y bibliografía, 2, págs. 174-177.
 
Monografías
F. Meco Tébar (1998), El deslinde de las costas: efectos jurídico civiles, Valencia: Tirant lo Blanch.
 
E. Desdentado Daroca (2007), La expropiación de enclaves privados en el litoral, Madrid: Civitas.
 
Capítulos de monografías
M.ª T. Carballeira Rivera (2012), «Protección de bienes culturales y límites a la propiedad en la jurisprudencia del TEDH», en E. García de Enterría y R. Alonso García (coords.), Administración y justicia. Un análisis jurisprudencial. Liber amicorum Tomás-Ramón Fernández, vol. II (págs. 2757-2774), Madrid: Civitas-Thomson Reuters.
 
L. Zea (2007), «América Latina: largo viaje hacia sí misma», en D. Pantoja (comp.), Antología del pensamiento latinoamericano sobre la educación, la cultura y las universidades (págs. 125-138), México: UDUAL.
 
Citas a obras ya citadas anteriormente
Cuando una obra se haya citado en forma completa con anterioridad, se hará de forma abreviada:
 
Véase Menéndez Rexach (2014: 40).
Para González-Varas Ibáñez (1995: 27), el deslinde de costas es un tema de gran actualidad…
 
Citas de páginas concretas
Cuando se estime necesario citar la página concreta de la referencia, se hará inmediatamente después de la referencia completa, y entre paréntesis:
 
F. Sospedra Navas (2004), «Los conflictos entre el dominio marítimo terrestre y la propiedad privada», Revista del Poder Judicial, 76, págs. 247-282 (pág. 252).
F. Meco Tébar (1998), El deslinde de las costas: efectos jurídico civiles, Valencia: Tirant lo Blanch (pág. 82).
 
Si la página concreta se cita en una referencia abreviada, se expresará tras el año y dos puntos:
 
Véase Menéndez Rexach (2014: 40).
 
Inserción de citas en textos más amplios
Si aparte de la referencia bibliográfica, en la misma frase se añade algo más o se incrusta alguna cita literal, separar la referencia bibliográfica con coma de lo que sigue a continuación:
 
a. Inserción en referencias que se citan por primera vez
Como afirma T. R. Fernández Rodríguez (2015), «La discrecionalidad técnica: un viejo fantasma que se desvanece», Revista de Administración Pública, 196, págs. 211-227, es un contrasentido hablar de «discrecionalidad técnica»: una operación técnica, por esencia, no puede llevar consigo la relativa libertad de actuación que la discrecionalidad comporta.
 
b. Inserción en referencias ya citadas (forma abreviada)
Como afirma Fernández Rodríguez (2015), es un contrasentido hablar de «discrecionalidad técnica»: una operación técnica, por esencia, no puede llevar consigo la relativa libertad de actuación que la discrecionalidad comporta.
 
Derechos de autor
En el momento en que una obra es aceptada para su publicación, se entiende que el autor cede a la RAP en exclusiva los derechos de reproducción, distribución y venta de su manuscrito para su explotación en todos los países del mundo en formato de revista de papel, así como en cualquier otro soporte magnético, óptico y digital.
Los autores cederán también a la RAP los derechos de comunicación pública para su difusión y explotación a través de intranets, internet y cualesquiera portales y dispositivos inalámbricos que decida el editor, mediante la puesta a disposición de los usuarios para consulta online de su contenido y su extracto, para su impresión en papel y/o para su descarga y archivo, todo ello en los términos y condiciones que consten en la web donde se halle alojada la obra.
 
Plagio y fraude científico
A efectos de lo estipulado en la Ley de Propiedad Intelectual respecto a las acciones y procedimientos que puedan emprenderse contra quien infrinja los derechos de propiedad intelectual, la publicación de un trabajo que atente contra dichos derechos será responsabilidad de los autores, que serán los que asuman los conflictos que pudieran tener lugar por razones de derechos de autor. Los conflictos más importantes pueden darse por la comisión de plagios y fraudes científicos. Se entiende por plagio:
     a. Presentar el trabajo ajeno como propio.
     b. Adoptar palabras o ideas de otros autores sin el debido reconocimiento.
     c. No emplear las comillas u otro formato distintivo en una cita literal.
     d. Dar información incorrecta sobre la verdadera fuente de una cita.
     e. El parafraseo de una fuente sin mencionar la fuente.
     f. El parafraseo abusivo, incluso si se menciona la fuente.
 
Las prácticas constitutivas de fraude científico son las siguientes:
     a. Fabricación, falsificación u omisión de datos y plagio.
     b. Publicación duplicada.
     c. Conflictos de autoría.
 
