# Supplementary material for: Peer Review in Law Journals
Source: Front Res Metr Anal. 2021 Dec 8;6:787768. doi: 10.3389/frma.2021.787768 (PMC8692876; doi:10.3389/frma.2021.787768)
Supplement: Supplementary file 3 [file DataSheet2.ZIP › DOCUMENT - 0034-9380_1.RTF]

CONTRIBUTIONS
0.	General.
The Journal is open for publication to Spanish and foreign specialists in the areas of public international law, private international law or international relations. Publication is conditional upon the adequate elaboration, scientific interest and quality of the paper submitted, which is to be assessed by peer-reviewers.
All contributions must be original and still unpublished; papers already printed elsewhere will not be accepted.
The Journal publishes preferably texts in Spanish. For further details see General Guidelines.
Click Style Guide for editorial instructions.
The authors will not be asked for any payment for the publication of their works.  There is no financial compensations to the authors, evaluators or editorial and management team.
COPYRIGHT: The authors assigns the right to publish his/her work in the REDI through the publishers  (Marcial Pons)
REUSE POLICY: Articles issued in the Revista Española de Derecho Internacional may be republished in other journals or in collective books, provided a note is added referring to the previous publication in the Revista Española de Derecho Internacional, indicating the issue, year and pages. On the pre-print and post-print policy, see infra under 8.
0.	Estudios 
Papers intended to be published as “Estudios” shall be sent by email to the senior manager editor, or to the editor-in-chief, Prof. Dr. Jorge Cardona Lloréns (jorge.cardona@uv.es). Reception of the originals will be acknowledged within thirty days. Preference will be given to studies matching one or several editorial lines of the Journal, adopted and made public for each issue in advance.
Acceptance for publication is conditional upon a positive peer-review assessment vis-à vis the innovative character, methodological approach, scientific proposal and contribution of a given paper. The evaluation is entrusted to external academics not belonging to the editorial board of the Journal; the anonymity is ensured both regarding the author and the reviewers. The publication may be conditioned to the amendments, if any, suggested by the reviewers with respect to the original version. Authors may be asked as well to ensure the editorial rules are met.
The decision regarding the publication or refusal of a paper submitted to the REDI shall be reasoned; it will be communicated six months after submission at the latest. The authors of accepted papers may be required to proof-read the first printed proofs, and to return them within a week. At this stage no substantial changes will be allowed.
For the details of the evaluation and editorial process, see the General Guidelines and the Best practice code.
The outcome of the peer-review assessment may be shared with the other journals of the AEPDIRI (Revista Electrónica de Estudios Internacionales and Spanish Yearbook of International Law)
 
0.	Foro
The "Foro" section is a non-fixed one devoted to contributions on topical issues identified as such during the semester prior to the publication of each issue, in the form of a debate between two or more specialists. As a rule participation to the Foro is made upon invitation; proposals to the Editorial Board may nevertheless be taken into consideration.
Coordinators:
`.	Derecho Internacional Público: Prof. Dr. Montserrat Abad  (montserrat.abad@uc3m.es )
`.	Derecho internacional privado, Prof Dr. Miquel Gardeñes (Miquel.Gardenes@uab.cat)
`.	​Relaciones internacionales, Prof. Dr. Rafael Grasa (rafael.grasa@uab.cat)
 
0.	Práctica española
This non-fixed section will host doctrinal contributions of an average extension commenting on relevant national practice (judicial, administrative or diplomatic decisions), in the fields of International Relations, Public International Law or Private International Law.
Coordinators:
?	Public International Law: Prof. Dr. Joaquin Alcaide (jalcaide@us.es)
?	Private international law: Prof. Dr. Cristina González (cgonzalezb@ub.edu)
?	International Relations: Prof. Dr. Inmaculada Marrero (marrero@ugr.es)
 
0.	Bibliografía
As a rule, all book reviews will be commissioned by the Section coordinators. Books sent to the REDI (to the Section coordinators, the editor-in-chief or the senior manager editor) and meant for this section will be included in a list published at the end of each issue.
Coordinators:
`.	Public International Law: Prof. Dr. Jaume Ferrer (jaume.ferrer@ua.es);
`.	Private International Law: Prof. Dr. Andrés Rodríguez (arodben@upo.es)
`.	International relations: Prof. Dr. Inmaculada Marrero (marrero@ugr.es)
 
6. Deadlines
In order to guarantee the periodicity of the Journal all manuscripts and contributions must be submitted before November 1st, for the first annual issue, and May 15th, for the second.
7. Compliance
Any breach of these rules will entail the rejection of the original submitted.
8. Pre-print/Post-print
A manuscript (pre-print) sent for publication in REDI shall not be published in any way prior to its evaluation and acceptance by the Editorial Board. The publication in institutional repositories or personal websites, either in Word format or as a pdf file, is allowed once the manuscript has been positively assessed, with the amendments suggested by the reviewers or by the editorial board, if any (post-print). A note shall be included stating the fact that the contribution has been accepted and is pending publication in the REDI, with reference to the specific issue. Once the manuscript has been released the post-print text shall be immediately replaced with the final text in the pdf format provided by the publishers. During the embargo established by the publisher, the publication may only appear at the author's personal website or in academic institutional repositories. After the embargo period (the last two issues of REDI), the author has full freedom to make it public by any means.
0.	Information
Further information can be obtained at revista-redi.es, or addressing:
?	The senior manager editor,
?	The editor-in-chief, Prof. Dr. Jorge Cardona LLoréns (e-mail: jorge.cardona@uv.es).
