# Supplementary material for: Peer Review in Law Journals
Source: Front Res Metr Anal. 2021 Dec 8;6:787768. doi: 10.3389/frma.2021.787768 (PMC8692876; doi:10.3389/frma.2021.787768)
Supplement: Supplementary file 3 [file DataSheet2.ZIP › DOCUMENT - 0034-9380_2.RTF]

﻿GENERAL GUIDELINES OF THE  

SPANISH JOURNAL OF INTERNATIONAL LAW (adopted by the Governing Board on March 12, 2018 on the motion of REDI’s Editorial Board ) 1. GENERAL GUIDELINES OF THE JOURNAL. CONTEXT AND OBJECTIVES 1.1.  The  Revista  Española  de  Derecho  Internacional  (Spanish  Journal  of  International  Law; hereinafter, the Journal or REDI) is a semi-annual scientific journal published by the Spanish  
Association of  International  Law and  International Relations Professors. The journal is made available  both  in  hard  copy  and  digital  format,  and  it  is  widely  accessible  at  www.revistaredi.es. The Association intends to place and maintain REDI at the highest level in prestigious academic indexes.  
 1.2. The Journal is made up of the following bodies and positions: a) the Editor-in-Chief; b) the  
Editorial Board; c) the Academic Secretary and d) the Advisory Board.  
 1.3. All REDI’s as as authors, and involved the publishing process must comply with REDI’s  Best Practice Code  posted on its website.  
 1.4.  The Journal will publish papers on public international law, private international law and international in with Association’s nature .  Regarding  the published  works,  over  the  year,  the  Journal  will  seek  to  achieve  a  balance  between  the abovementioned  matters  in  the  Studies  Section,  and  it  will  encourage  the  publication  of contributions from the various areas of study in the remaining Sections.  
 1.5.  Both Spanish and foreign scholars are welcome to publish in the Journal. REDI publishes scholarly  works  written,  preferably,  in  Spanish.  However,  the  Editorial  Board  may exceptionally accept English or French contributions from foreign authors.  
 1.6. REDI’s only  key publication criteria for any works requested or received are their interest, their scientific scope and their quality.  
 1.7. The Editor-in-Chief and the Editorial Board must seek compliance with these Guidelines.  
In  accordance  with  these  Guidelines,  the    Governing  Board  will  submit  to  the  
Association’s  General  Meeting  any  proposals  regarding  the  appointment  and,  where appropriate, reappointment of any members of the Journal’s governing bodies . REDI’s Editorin-Chief submit reports the  General  Meeting.  Upon  receipt  of these  reports,  the  General  Meeting  will  submit  any  relevant  comments  or  remarks  on  the  
Journal’s development .
 2. REDI’s EDITOR -IN-CHIEF 2.1. The Editor -in-Chief  will  be  appointed  by  majority  vote  by  the  
Governing Board from among the members of the Editorial Board.  
  
As for any subsequent governing or leading positions, the Editorial Board and the Association’s  
Governing  Board  will  seek  a  well-balanced  representation  of  the  various  areas  of  study comprising the Association.  
 2.2. The Editor-in-Chief position, as well as any other leading or governing position, is for a 4-year term, renewable once for another 4-year period under the procedure provided in paragraph 2.3. Regardless of any additional functions provided by these Guidelines, the Editor-in-Chief, alongside any other leading or governing positions, will be responsible for the following duties:  
 a) Representing the Journal; b) Calling and chairing the Editorial Board meetings; c) Proposing to the Editorial Board the appointment of the Journal’s Academic Secretary ; d) Submitting proposals to the Editorial Board and advancing its activities, whilst coordinating the works of the various Journal Sections, seeking the  timely publication of these works and ensuring a high scientific quality and academic value of any published works; e)  Periodically to Association’s Board the Advisory  
Board on the Journal’s development; if deemed convenient, the Editor -in-Chief may also report to the Association’s  General Meeting.  
 3. REDI’s EDITORIAL BOARD 3.1.  The  Editorial  Board  will  be  made  up  of  renowned  experts  in  the  fields  covered  by  the  
Journal, and they will be appointed in accordance with the rules of the Spanish Association of  
International  Law  and  International  Relations  Professors.  Their  full  names,  along  with  the institutions where they work and their contact information will appear on the Journal’s website .  
  
The Editorial Board will be made up of 9 members:  
 a) 4 experts in public international law; b) 3 experts in private international law; c) 2 experts in international relations.  
 3.2. The Editorial Board members will be appointed by the Association’s  General Meeting.  
  
For this purpose, the Governing Board may submit proposals to the General Meeting. It will also submit, sufficiently in advance, any proposals made by the Association members.  
 3.3. The Editorial Board will be responsible for the following duties:  
 a) Proposing  to  the  Governing  Board  the  appointment  of  the  Editor-in-Chief  and,  if appropriate, of any other leading or governing positions; b)  Establishing the Journal’s and its Sections’ editorial line , promoting the publication of works on scientifically interesting or current topics; c) Scheduling, at the initiative of the Editor-in-Chief, the Journal’s issues,  seeking the timely publication of these works and ensuring a high scientific quality of any published works.  The  Editorial  Board  will  also  lay  down  the  formal  criteria  to  be  met  by  the relevant works from each Section.  
 d) Proposing the Gove rning  Board  any  measures  that  may  have  a financial impact on the Journal or that may be related to its printing or distribution.  
 3.4.  Following  a  proposal  submitted  by  the  Editor-in-Chief,  the  Editorial  Board  will  appoint the  Section  Coordinators,  seeking  to  achieve  a  balance  between  the  various  areas  of  study addressed by the works.  
 3.5. The Editorial Board will meet as convened by the Editor-in-Chief and at least twice per calendar year.  
 4. REDI’s ACADEMIC SECRETARY 4.1. Following a proposal submitted by the Editor-in-Chief, the Association’s  Governing Board will appoint the Academic Secretary.  
 4.2.  The Academic Secretary position is for a 4-year term, renewable once for another 4-year period under the procedure provided in paragraph 4.1.  
 4.3. The Academic Secretary will be responsible for the following duties:  
 a) Implementing any agreements and decisions made by the Editorial Board regarding the scheduling  and publishing of the works,  as well as undertaking any specific tasks entrusted by the Editor-in-Chief, including the relationship with authors, reviewers and editors; b) Delivering  or  sending  the  original  issues  to  print  for  publication,  along  with  the magnetic media agreed with the printing services. The Academic Secretary will give the relevant  instructions  to  the  authors the general  on  the submission of manuscripts; c) Seeking  compliance  with  any  technical  publishing  requirements  and  with  the  
Journal’s publication schedule agreed with the publishing company ; d)  Ensuring  a  timely  proofreading  process,  as  well  as  the  delivery  of  offprints  to  the authors, duly verifying the Journal’s distribution dates by the publishing company ; e)  Drafting and preserving any minutes of Editorial Board meetings following approval by the Editorial Board.  
 5. REDI’s  ADVISORY BOARD 5.1. The  Advisory  Board  will  be  made  up  of  12  Professors  (Catedráticos)  appointed  by  the  
Association’s  General  Meeting  for  a  6-year  term  following  a  proposal  submitted  by  the  
Association’s Governing Board .  
  
The appointment of Advisory Board members will be made having regard to the candidates’ scientific academic as as their contribution the purpose,  seeking  to  achieve  a  balance  between  public  international  law,  private  international law and international relations.  
 5.2. The  Chairman  of  the  Advisory  Board  will  be  elected  by  a  majority  vote  of  all  Advisory  
Board members.  
 5.3. The Advisory Board will be responsible for the following duties:  
 a)  Monitoring  the  editorial  line  approved  by  the  Editorial  Board,  assessing  the  
Journal’s yearly performance ; b)  Submitting any comments and proposals to the Editor-in-Chief, the Editorial Board and, where appropriate, the Association’s Governing Board ; c)  Requesting  a  report  from  the  Editorial  Board  on  any  discrepancies  or  complaints from  the  Chairman  of  the  Advisory  Board  addressed  by  the  Association  members regarding the works submitted for publication; d) Any other advisory duties related to the Journal that may be assigned to the Advisory  
Board by the Editor-in-Chief.  
 5.4. The Editorial and Advisory Boards may hold joint meetings on the motion of the Advisory  
Board or the Editor-in-Chief.  
 6. EDITORIAL PROCESS 6.1. Manuscript selection process. Selection of reviewers 6.1.1. Any considered “Studies” be  to  a  blind  review  process.  
Accordingly, the Editor-in-Chief, through the Academic Secretary, will request two experts on the  field  to  issue  two  separate  reports  in  order  to  make  a  publication  decision.  In  case  of disagreement between the two peer reviewers, the Editor-in-Chief will request a third report.  
  
The Editorial  Board is responsible for  all publication decisions, which  must be based on the quality  of  manuscripts,  their  relevance  for  the  Journal  and  its  readers,  as  well  as  on  any recommendations issued by the peer reviewers and on copyright rules. The Academic Secretary will notify the authors of the decision made by the Editorial Board.  
 6.1.2.  The purpose of the peer review process is to assist the Editor-in-Chief and the Editorial  
Board  in  their  publication  decisions  for  each  issue.  It  is  also  aimed  at  helping  authors  to improve any submitted manuscripts. Peer reviewers will be experts in the field external both to the  Editorial  Board  and  the  Advisory  Board.  The  Editor-in-Chief  will  not  request  any  peer reviews to any reviewers whose impartiality may be compromised due to a personal, working or  any  other  kind  of  relationship  with  the  author.  REDI’s Secretary take necessary  measures  to  ensure  that  the  digital  medium  has  no  trace  of  the or reviewers’  identity.
 6.1.3. The  Editor-in-Chief  may  at  a  preliminary  stage  (with  no  peer  review)  reject  any manuscripts that fall outside of the scientific scope of the Journal or that clearly fail to meet the quality  standards  to  be  subject  to  peer  review.  Rejections  at  this  preliminary  stage  must  be notified to the Editorial Board. Only those manuscripts meeting these preliminary requirements will be subject to a blind peer review process.  
 6.1.4. The reviewers must not know the author’s identity. If t hey somehow know or become aware of the author’s identity, they must refrain from reviewing his/her manuscript.  When in doubt about the author’s identity, peer reviewers must discuss it with the Academic Secretary or the Editor-in-Chief.  
 6.1.5.  Any persons requested to carry out a peer review may refuse to review a manuscript if they  feel  unfit  to  do  so or  if  they  do  not  consider  themselves  able  to  perform  the  review  on time,  promptly  notifying  the  Academic  Secretary  so  that  they  may  be  replaced  as  soon  as possible.  
 6.2. Duties of reviewers 6.2.1. Following  his/her  assessment,  the  peer  reviewer  must  recommend  whether  or  not  to publish the manuscript, choosing one of the options listed below.  
 a) The manuscript can be published as submitted.  
 b)  It is advisable to publish the manuscript (in its original wording or suggesting certain changes).  
 c)  It is not advisable to publish the manuscript, since it requires major changes.  
 d) The manuscript must not be published.  
 6.2.2.  Peer reviewers’  recommendations will generally include specific comments addressed to the authors. The content of these comments should be made available to the authors.  
 6.2.3.  Peer  reviewers’   recommendations  will  not  be  binding  on  the  Editor-in-Chief  or  the  
Editorial Board, who will decide by a majority vote whether or not to publish the manuscript.  
 6.2.4. Reviews  should  be  conducted  objectively.  Reviewers  must  express  their  views  clearly with  appropriate  supporting  arguments  in  a  constructive  manner,  so  as  to  allow  authors  to improve their manuscripts based on the reviews. Personal criticism of the author and offensive remarks of any kind shall be unacceptable.  
 6.3. Editorial review  
  
The Editor-in-Chief and the Academic Secretary will agree on a team of editors (experts in the fields covered by the Journal) that will be appointed in order to assist the Journal. The final version  of  the  manuscripts  accepted  for  publication  will  be  subject  to  an  editorial  review process that will be carried out by an editor from REDI’s team of editors . The editor may return the manuscript to the author: 1) if deemed convenient because the author has failed to comply with  the  basic  style  requirements;  2)  if  deemed  necessary  because  there  are  incomplete paragraphs or footnotes, or 3) if deemed necessary because the manuscript lacks information that can only be provided by the author.
 7. AUTHORS 7.1. Authors  must  submit  their  manuscripts  in  compliance  with  the  submission  requirements (digital  format,  length,  etc.)  and style published REDI’s Any manuscripts that fail to fulfil these requirements will not be subject to peer review.  
 7.2.  Authors  must  ensure  that  they  only  submit  entirely  original  manuscripts.  Their  work should  include  all  the  specifics  about  their  research  and  conclusions.  Additionally,  the manuscript must include sufficient details and references so as to allow for a third-party review.  
If authors have used the works of others, they must ensure that these works are appropriately cited or quoted. Any publications that have been material for the preparation of the submitted manuscript should be appropriately referenced. Making inaccurate or false claims voluntarily shall  be  unacceptable,  and  the  manuscript  will  be  immediately  removed  from  the  editorial process.  
 7.3. This will publish manuscripts essentially with author’s already  published  research,  either  in  other  journals  or  as  part  of  collective  or  monographic works.  Notwithstanding  the  foregoing,  the  Editorial  Board  may  exceptionally  accept  a manuscript for publication providing data or conclusions similar or identical to those included in an already published work if, in the Editorial Board’s view, the circumstances so require, yet expressly  stating  this  situation  and  referencing  the  first  publication.  Under  similar circumstances, the Editorial Board may accept a subsequent edition of a manuscript that had already been published in the Journal, yet including the said specific references.  
 7.4. If the Editor-in-Chief became aware of any errors through third parties, he/she must urge the author to correct the work as soon as possible.  In case of plagiarism, the Editor-in-Chief will notify the author of the need to get the publication offline requesting, where appropriate, the author’s assistance .  
 7.5.  Authors  must  be  subject  to  the  peer  review  process,  and  they  should  cooperate  if  so requested. If, as a result of the peer review, the manuscript must be modified, authors should take reviewers’  nts  into  account,  in  order  to  make  any  required  changes, subsequently re-submitting the manuscript to the Journal within the specified deadline. If the author decides not to take into account the reviewer’s recommendations, he/she must  submit a written report, giving reasons, to the Editor-in-Chief or the Academic Secretary. Having regard to the reviewers’ recommendations, the Editorial Board will assess whether or not  additional modifications are required.  
 7.6. No manuscripts submitted for publication in the REDI will be published in any form (pre-print) without the prior assessment and acceptance by the Editorial Board. Upon acceptance of a  manuscript  for  publication,  authors  may  only  publish  it,  including  the  modifications suggested by the reviewers or the Editorial Board (post-print), on their personal website or in an academic repository expressly stating that it has been accepted for publication by the REDI and specifying the repository volume. Once the manuscript has been published in the REDI, the author must promptly replace the text with the finally published pdf document provided by the editor. During the temporary ‘freezing period’ provided by the editor, the publication may only appear on the author’s personal website or in academic repositories.  Following this ‘freezing period’ (REDI’s last two issues), the author may freely disseminate the work by any means .
 7.7. Authors  will  receive  a  digital  copy  of  their  work  and,  if  they  are  not  members  of  the  
Association, they will also receive a hard copy of the issue where their text is published.  
  
FINAL PROVISION  
  
These Guidelines have been prepared by REDI’s Editorial Boar d and they will be applicable immediately following their approval by the Association’s Governing Board, without prejudice to their subsequent validation by the General Meeting.  
  
Any  amendment  to  these  Guidelines  must  be  made  in  accordance  with  the  abovementioned procedure.
