# Supplementary material for: Peer Review in Law Journals
Source: Front Res Metr Anal. 2021 Dec 8;6:787768. doi: 10.3389/frma.2021.787768 (PMC8692876; doi:10.3389/frma.2021.787768)
Supplement: Supplementary file 3 [file DataSheet2.ZIP › DOCUMENT - 0035-5895.RTF]

CRITERI DI REFERAGGIO
Tutti gli scritti sono sottoposti ad un preliminare verifica da parte del Direttore al fine di accertare la loro coerenza con le materie di interesse della Rivista. Gli scritti, che compaiono nelle rubriche «articoli», «varietà» e «note a sentenza», sono poi assoggettati a referaggio, con il sistema del «doppio cieco» («double blind peer review process»); gli scritti, che compaiono nelle rubriche «attualità» e «osservazioni a sentenza», sono assoggettati a referaggio, con il sistema del «referaggio anonimo» («blind peer review process»). È rispettato l’anonimato sia dell’autore che dei revisori previa verifica di coerenza con i temi di interesse della Rivista da parte del Direttore.
La valutazione è affidata a due esperti del tema trattato, designati dal Direttore, preferibilmente che rivestano, o abbiano rivestito, la posizione di professore universitario di prima fascia nelle università italiane o posizione equivalente nelle università straniere.
I revisori formuleranno il proprio giudizio, tenendo conto dei seguenti parametri: ampiezza dell’articolazione e analisi critica; completezza delle fonti e dell’informazione bibliografica.
Sulla base di tali parametri, i revisori potranno formulare i seguenti giudizi:
a) pubblicabile senza modifiche;
b) pubblicabile previo apporto di modifiche;
c) da rivedere in maniera sostanziale;
d) da rigettare.
La revisione avverrà in maniera che ciascun autore riceva l’esito della revisione possibilmente entro 20 giorni dall’invio del proprio elaborato redatto nel rispetto delle regole redazionali. Nel caso di giudizio discordante fra i due revisori, la decisione finale sarà assunta dal Direttore, salvo casi particolari in cui il Direttore medesimo provvederà a nominare tempestivamente un terzo revisore a cui rimettere la valutazione dell’elaborato.
Il Direttore, su sua responsabilità, può decidere di non assoggettare a revisione scritti pubblicati su invito o comunque di autori di particolare prestigio.
