# Supplementary material for: Peer Review in Law Journals
Source: Front Res Metr Anal. 2021 Dec 8;6:787768. doi: 10.3389/frma.2021.787768 (PMC8692876; doi:10.3389/frma.2021.787768)
Supplement: Supplementary file 3 [file DataSheet2.ZIP › DOCUMENT - 0035-6093.RTF]

Codice etico 
Il codice etico della “Rivista di diritto civile” si conforma alle norme contenute nel Regolamento per la classificazione delle riviste nelle aree non bibliometriche (Approvato con delibera del Consiglio Direttivo ANVUR n. 42 del 20/02/2019), alle norme etiche di comportamento e agli standard indicati dal “Committee on Publication Ethics” (COPE) nel Code of Conduct and Best Pratice Guidelines for Journal Editors(http://publicationethics.org/). L’Editore garantisce l’utilizzo delle seguenti buone pratiche dal punto di vista etico nel processo di pubblicazione:  La Direzione è responsabile della decisione di pubblicare o meno i contributi proposti alla Rivista sulla base della Procedura di revisione riportata in ciascun fascicolo. La Direzione decide in ordine alla pubblicazione o meno dei contributi proposti alla Rivista, esclusivamente in base al valore scientifico, alla rilevanza e all’originalità del contenuto del contributo senza distinzione di, genere, orientamento sessuale, credo religioso, origine etnica, cittadinanza, nonché di orientamento scientifico o accademico o politico degli Autori.La Direzione e il Comitato Editoriale assicurano che il materiale inviato rimarrà strettamente confidenziale durante la procedura di revisione. La Direzione individua il Comitato di Revisione sulla base della competenza e della fiducia, onde conseguire una valutazione adeguata del contributo ai fini della pubblicazione. Qualora la Direzione o il Comitato Editoriale dovessero ricevere segnalazioni in merito ad errori o imprecisioni, conflitto di interessi o plagio in un contributo pubblicato, ne daranno tempestiva comunicazione all’Autore e all’Editore ed intraprenderanno le azioni necessarie per chiarire la questione. La revisione paritaria deve essere svolta in modo corretto e oggettivo. I Revisori sono invitati a motivare in modo adeguato le proprie valutazioni.
