# Supplementary material for: Peer Review in Law Journals
Source: Front Res Metr Anal. 2021 Dec 8;6:787768. doi: 10.3389/frma.2021.787768 (PMC8692876; doi:10.3389/frma.2021.787768)
Supplement: Supplementary file 3 [file DataSheet2.ZIP › DOCUMENT - 0035-6131.RTF]

RIVISTA DI DIRITTO FINANZIARIO E SCIENZA DELLE FINANZE

Regolamento di autodisciplina dell'attività di revisione


Articolo 1

Procedura di revisione

0.	Ai sensi dell'art. 5, comma 2, del Regolamento approvato dall'ANVUR nella seduta del 3 maggio 2017, tutti i contributi scientifici pubblicati nella Rivista sono sottoposti a revisione, fatta eccezione per i contributi non rilevanti per le finalità che presiedono alla classificazione delle riviste.

0.	I contributi sono sottoposti: a) alla valutazione preliminare della Direzione, che ne verifica la coerenza con i criteri di qualità della Rivista; b) se approvati, alla valutazione del Comitato scientifico dei revisori.


Articolo 2

Comitato scientifico dei revisori

0.	Il Comitato scientifico dei revisori è formato da professori ordinari di ruolo e fuori ruolo, nonché da professori abilitati alla prima fascia, italiani e stranieri, afferenti per la maggior parte ai settori scientifico disciplinari IUS/12, SECS- P0/3 e SECS-P0/2 e indicati in un elenco periodicamente aggiornato dalla Direzione e pubblicato nella Rivista nei modi graficamente più opportuni.

0.	Per consentire l'interdisciplinarità della valutazione, possono far parte del Comitato scientifico dei revisori anche i professori di altri settori disciplinari in una percentuale non superiore al 25% dei componenti.

0.	L'attività dei revisori è svolta su base volontaria e non è retribuita.


Articolo 3

Valutazione del Comitato scientifico dei revisori

0.	Dopo la valutazione preliminare, la Direzione trasmette al Comitato scientifico dei revisori i contributi approvati, previa rimozione di ogni elemento idoneo a identificare gli autori.

0.	Il Comitato scientifico dei revisori designa un revisore, o più revisori, qualora il carattere interdisciplinare del contributo lo richieda, con una procedura a doppio cieco, tenendo conto delle specifiche competenze in relazione all'argomento del contributo e garantendo l'indipendenza del giudizio.

0.	I revisori designati esprimono il proprio giudizio sulla forma espositiva, sull'originalità e sul contenuto dei contributi, con particolare riguardo alla coerenza delle argomentazioni e alla conoscenza della letteratura scientifica sul tema trattato.


0.	L'esito del giudizio può essere positivo, negativo o positivo con riserva di modifiche o integrazioni da parte degli autori.

0.	In casi eccezionali la Direzione può assumere direttamente la responsabilità della pubblicazione, segnalando la circostanza e le relative motivazioni in una nota nella prima pagina del contributo.


Articolo 4

Esito della valutazione


0.	A seguito del giudizio dei revisori designati, i contributi sono sottoposti alla valutazione finale della Direzione.

0.	In caso di giudizio positivo con riserva, la Direzione sospende la pubblicazione in attesa delle modifiche o delle integrazioni da parte degli autori.
