# Supplementary material for: Peer Review in Law Journals
Source: Front Res Metr Anal. 2021 Dec 8;6:787768. doi: 10.3389/frma.2021.787768 (PMC8692876; doi:10.3389/frma.2021.787768)
Supplement: Supplementary file 3 [file DataSheet2.ZIP › DOCUMENT - 0035-6158.RTF]

Codice etico di pubblicazione

La Rivista di diritto internazionale è una rivista scientifica, edita da Giuffrè sin dal 1953 ed ora da Giuffrè Francis Lefebvre, con peer-review di tipo double-blind.

Tutte le parti coinvolte – direzione, valutatori e autori – conoscono e condividono i requisiti etici richiesti.

Direzione

La direzione collegiale discute e decide le linee editoriali e i temi scientifici da privilegiare. Il Direttore responsabile coordina le strategie e le politiche editoriali della Rivista. Le decisioni di accettare o rifiutare un contributo per la pubblicazione devono essere basate sui seguenti criteri: insussistenza di un significativo conflitto di interessi, rilevanza scientifica, originalità, chiarezza e pertinenza dello scritto rispetto all'ambito scientifico coperto dalla Rivista, dimensioni del contributo in rapporto alla programmazione dei singoli fascicoli. I manoscritti devono essere valutati esclusivamente per il loro contenuto scientifico, senza alcuna discriminazione di genere, orientamento sessuale, religione, origine etnica, razza, cittadinanza o orientamento politico degli autori. La Direzione valuta gli articoli ricevuti supportata dal parere di referees.

La Direzione si preoccupa di garantire che la procedura di revisione avvenga in modo tempestivo, monitorandone il regolare svolgimento al fine di aumentare l'efficienza e prevenire ritardi. La Direzione, pur nel rispetto dell'anonimato dell'autore e del valutatore, assicura – per quanto possibile – che i referees non ricevano manoscritti in relazione ai quali abbiano o possano avere conflitto d'interessi derivante da rapporto di concorrenza, collaborazione o altro collegamento con gli autori, aziende o enti che abbiano relazione con l'oggetto del manoscritto. La Direzione è tenuta a garantire che il materiale inviato alla Rivista rimanga riservato mentre è sotto revisione.

La Direzione è tenuta a pubblicare correzioni, chiarimenti, ritrattazioni e scuse nel caso in cui riceva segnalazioni rilevanti relative a errori, imprecisioni, plagio, conflitto d'interesse. In tal caso è tenuta a darne tempestiva comunicazione all'Autore del manoscritto, intraprendendo ogni azione necessaria e, se il caso, pubblicando una ritrattazione o Erratum.

Referees esterni

Ai fini della pubblicazione di un contributo, la Direzione della Rivista si avvale – di norma – del supporto di referees, selezionati secondo i criteri ANVUR. Il parere dei referees assiste la Direzione nell'adozione di decisioni sulla pubblicazione degli articoli ricevuti e può indicare alla Direzione gli adeguamenti necessari al loro miglioramento.

L'individuazione dei referees deve avvenire in aderenza allo scopo e al contenuto del manoscritto per ottenere le migliori valutazioni possibili. La revisione deve essere condotta in maniera obiettiva e imparziale. I referees devono esprimere la propria valutazione in modo chiaro ed esaustivo, con il supporto di argomentazioni limpide e


documentate. Nell'ipotesi in cui i revisori identifichino una somiglianza sostanziale o una sovrapposizione fra il manoscritto oggetto d'esame e ogni altro documento in qualsiasi modo pubblicato, del quale hanno conoscenza personale, essi sono tenuti a richiamare l'attenzione della Direzione. La procedura di peer review deve essere svolta in tempi ragionevoli. Non è consentito utilizzare le informazioni ottenute durante il processo di peer review per il proprio o altrui vantaggio. I referees hanno l'obbligo, inoltre, di non divulgare il materiale ricevuto in valutazione, da considerarsi strettamente confidenziale, a non discuterne i testi con altre persone senza autorizzazione della Direzione.

Regole di autodisciplina
0.	La valutazione dei contributi è affidata di norma a due referees.

0.	Il contributo è inviato ai valutatori senza indicazione dell'identità dell'autore.

0.	L'identità dei valutatori di ciascun contributo è anonima.

0.	In caso di giudizio contrastante la Direzione si assume la responsabilità della decisione.

0.	Qualora sia formulato un giudizio positivo condizionato a revisione o modifica del contributo, la Direzione ne promuove la pubblicazione solo a seguito dell'adeguamento, assumendosi la responsabilità della verifica.

Autori

Gli autori si fanno garanti dell'originalità del loro lavoro e assicurano che, qualora abbiano utilizzato testi altrui, ciò sia adeguatamente segnalato, secondo l'uso corrente nella disciplina; inoltre si impegnano a dare la corretta indicazione delle pubblicazioni o fonti similari citate nel loro contributo.

Nel proporre uno scritto alla Rivista, gli autori si impegnano a non presentare il medesimo scritto, ovvero una parte sostanziale di esso, ad altre riviste fino al termine del processo di revisione.

Inviando un contributo, l'Autore conviene che, una volta che il lavoro sia accettato per la pubblicazione, Egli perde tutti i diritti di sfruttamento economico, senza limiti di spazio e con tutte le modalità e tecnologie attualmente esistenti e/o in futuro sviluppate. I diritti saranno trasferiti alla proprietà della Rivista. La paternità letteraria del testo pubblicato è limitata a coloro che hanno dato un contributo significativo per l'ideazione, la progettazione, l'esecuzione o l'interpretazione dello studio. Tutti coloro che hanno dato un contributo significativo devono essere elencati come co-autori. Ove sia uno solo degli autori a proporre l'articolo per la pubblicazione, egli deve assicurarsi che tutti i coautori siano stati opportunamente indicati, che abbiano letto e approvato la versione finale dell'articolo e che siano d'accordo su un'eventuale pubblicazione.

Qualora un autore riscontri errori significativi o inesattezze nel manoscritto pubblicato ha il dovere di comunicarlo tempestivamente alla Direzione.

Eventuali fonti di sostegno finanziario al progetto di pubblicazione devono essere indicate.


Tutti gli autori devono dichiarare esplicitamente ogni conflitto di interesse significativo che potrebbe influenzare i risultati della ricerca o l'interpretazione dei dati.

Agli autori viene richiesto di omettere dal proprio scritto, prima che sia sottoposto ai referees, qualsiasi riferimento che possa farli individuare come tali.
