# Supplementary material for: Peer Review in Law Journals
Source: Front Res Metr Anal. 2021 Dec 8;6:787768. doi: 10.3389/frma.2021.787768 (PMC8692876; doi:10.3389/frma.2021.787768)
Supplement: Supplementary file 3 [file DataSheet2.ZIP › DOCUMENT - 0048-7694_1.RTF]

REVISTA DE ESTUDIOS POLÍTICOS (REP)
Enfoque y alcance
La Revista de Estudios Políticos (REP) publica trabajos de investigación, originales e inéditos, sobre Teoría de la Constitución, Teoría del Estado, Ciencia Política, Historia Política e Historia del Pensamiento Político. Su perspectiva amplia y multidisciplinar, y la elevada calidad de sus estudios la convierten en referencia fundamental en su ámbito académico.
Proceso de evaluación por pares
La Revista de Estudios Políticos acusará recibo de todos los originales en el plazo de treinta días desde su recepción. El Consejo de Redacción decidirá la publicación de los trabajos sobre la base de dos informes de evaluación, emitidos por sendos especialistas ajenos a la organización editorial de la revista, aplicándose  el método doble ciego. La publicación podrá quedar condicionada a la introducción de cambios con respecto a la versión original motivada por el resultado de las evaluaciones. En este caso, el plazo máximo para incorporar las modificaciones y enviar de nuevo el original a la Revista, será de un mes. La decisión sobre la publicación no excederá de seis meses. Los autores de artículos aceptados para publicación podrán ser requeridos para la corrección de pruebas de imprenta, que habrán de ser devueltas en el plazo de 48 horas. No se permitirá la introducción de cambios sustanciales en las pruebas, quedando éstos limitados a la corrección de errores con respecto a la versión aceptada.
Frecuencia de publicación
La Revista de Estudios Políticos (REP) se viene publicando ininterrumpidamente desde 1941, y actualmente tiene periodicidad trimestral, publicándose cuatro veces al año, en marzo, junio, septiembre y diciembre por el Centro de Estudios Políticos y Constitucionales (CEPC).
Política de acceso abierto
Esta revista provee acceso libre inmediato a su contenido bajo el principio de que hacer disponible gratuitamente la investigación al público, lo cual fomenta un mayor intercambio de conocimiento global. La publicación no tiene ningún coste para los autores.
