# Supplementary material for: Peer Review in Law Journals
Source: Front Res Metr Anal. 2021 Dec 8;6:787768. doi: 10.3389/frma.2021.787768 (PMC8692876; doi:10.3389/frma.2021.787768)
Supplement: Supplementary file 3 [file DataSheet2.ZIP › DOCUMENT - 0210-0444_1.RTF]

CÓDIGO DE BUENAS PRÁCTICAS


La Revista Crítica de Derecho Inmobiliario, fundada en 1925 y editada por el Colegio de Registradores de la Propiedad, Mercantiles y de Bienes Muebles, ha pretendido desde su origen contribuir a la investigación en el ámbito del derecho privado. Para ello ha sido siempre fundamental asegurar la calidad de los artículos y trabajos publicados, así como el cumplimiento de unos estándares de ética editorial que permitan configurarla como una referencia esencial para la comunidad científica.

Los principios que en la actualidad inspiran a la Revista están basados en la Guía Cope de Buenas Prácticas para Editores de Publicaciones (https://publicationethics.org/resources/guidelines). El objetivo último es que, durante todo el proceso, desde que un autor solicita la publicación de su trabajo hasta que el lector accede a su contenido, se mantenga un comportamiento ético irreprochable. De igual forma resulta fundamental la observancia de los principios de transparencia que en la actualidad exigen las distintas agencias de calificación editorial.

A) Normas sobre propiedad de la Revista y sus órganos de gobierno:

En la página web de la Revista aparece con total claridad que el editor titular de la misma es el Colegio de Registradores de la Propiedad, Mercantiles y de Bienes Muebles. De igual forma se incorpora un anexo con las Normas Reguladoras de Organización de la Revista (enlace), donde se definen las competencias de los diferentes órganos de gobierno.

B)  Normas que garantizan el respeto al principio de igualdad:

La Revista garantiza que en el proceso de selección de los textos a publicar solo se examinará el contenido intelectual de los trabajos, sin que en ningún caso puedan tenerse en cuenta el sexo, la raza, la nacionalidad, las creencias religiosas o el pensamiento político de los autores.

De igual forma, se mantendrá un estricto respeto al principio de igualdad género y de no discriminación por razón de sexo, raza, creencias religiosas o pensamiento político para la formación de los órganos de gobierno de la Revista, procurando avanzar hacia una progresiva paridad entre hombres y mujeres en los mismos. 

C) Normas de transparencia en el proceso de evaluación de trabajos:

El proceso de selección de trabajos para su publicación en la Revista está claramente definido en un protocolo que aparece publicado en la página web (enlace) para su conocimiento por autores y suscriptores. Dicho protocolo está basado en los siguientes principios:

?	Es un procedimiento totalmente electrónico en el que todas las comunicaciones con el autor se realizarán a través de la web.
?	La Revista dispone de un Comité Editorial integrado por  revisores externos que serán los encargados de valorar los trabajos cuya publicación decidirá la Comisión Ejecutiva.
?	El proceso garantizará en todo momento la confidencialidad, sin que el contenido de los trabajos sometidos a evaluación pueda ser revelado a nadie ajeno a dicho proceso de revisión.
?	La evaluación de los trabajos debe estar basada en la imparcialidad de los revisores y de la Comisión Ejecutiva. Los evaluadores realizarán su labor sin conocer los datos de autoría del trabajo. No obstante lo cual, si por cualquier cauce llegaran a conocer la identidad del autor, deben abstenerse de realizar dicha evaluación. Los miembros de la Comisión Ejecutiva deberán abstenerse de participar en la votación sobre la admisión a publicación de un trabajo con cuyo autor exista cualquier conflicto de intereses. Se entenderá que existe conflicto de intereses cuando concurra relación de parentesco hasta el cuarto grado por consanguinidad o afinidad, relación conyugal o asimilada, amistad o enemistad manifiesta, subordinación o vinculación contractual.
?	También deberá abstenerse de realizar la evaluación aquel revisor que considere que el trabajo a analizar no pertenece al campo de su propia especialización, o cuando prevea que no podrá cumplir los plazos previstos en el protocolo.
?	El sistema de revisión consiste en un procedimiento de evaluación por pares doble ciego. La revisión por pares se define como la obtención de asesoramiento sobre manuscritos individuales por parte de revisores expertos en el tema que no forman parte del personal editorial de la revista. Al ser doble ciego, ni el autor podrá conocer la identidad de los evaluadores, ni estos la de aquel.
?	La evaluación de los trabajos se llevará a cabo cumpliendo los plazos que se determinan en el protocolo que aparece publicado en la página web
?	No se admitirán a evaluación los trabajos que no cumplan las normas de publicación que aparecen recogidas en la propia web.
?	Los evaluadores han de actuar de forma puramente objetiva, siguiendo los criterios de evaluación que recoge el protocolo publicado en la web. Deben exponer con claridad su juicio sobre el trabajo, apoyándose en argumentos objetivos y no en meras opiniones personales.

D) Normas que garantizan la originalidad de los trabajos publicados:

?	La primera de las exigencias que el protocolo publicado en la web de la Revista establece para la admisión de trabajos que pretendan su publicación es su originalidad. 

?	La Secretaría de la Revista, los evaluadores externos y la Comisión Ejecutiva realizarán un examen exhaustivo de cada trabajo para confirmar que se trata de una publicación original y que no contiene plagio. Para ello utilizarán alguno de los programas informáticos diseñados para tal fin (falta especificar el nombre de dicho programa. Estoy pendiente de que me indique Manolo cuál va a ser finalmente el que compremos).

?	Los autores deben presentar trabajos que no hayan aparecido en todo o en parte en ninguna otra revista o publicación. Tampoco aquellos textos que estén bajo consideración o hayan sido aceptados para su publicación en cualquier otro lugar, aun cuando esta no se haya producido de manera efectiva. Además, no deben publicar ni solicitar la publicación del trabajo presentado en ninguna otra revista.  

?	En el formulario de presentación de trabajos que está habilitado en la web aparecerá una cláusula específica por la que la cual el autor asegurará que el manuscrito no ha sido publicado ni se ha solicitado su publicación en ningún otro lugar, así como el compromiso de que no va a presentarlo en ninguna otra publicación hasta que se decida sobre su aceptación en la Revista. (REVISAR PÁGINA WEB).

?	Se considera autor de una publicación a todo aquel que haya realizado una contribución significativa a la concepción, estructura o planteamiento del artículo o trabajo. En caso de ser varios los autores, habrán de asegurar al presentar el trabajo que todos ellos han prestado su conformidad a la versión definitiva del manuscrito presentado a publicación, así como al hecho de ser incluidos como coautores.

?	Se considera plagio, además de apropiación en todo o en parte de un trabajo ajeno, la atribución de idea ajena sin cita de su autor; no emplear las comillas en una cita literal; parafrasear sin citar la fuente; parafrasear abusivamente aun citando la fuente; no informar correctamente sobre la verdadera fuente. Junto al plagio, constituye fraude científico la fabricación, falsificación u omisión de datos, la publicación reiterada o redundante y los conflictos de autoría. 

?	Si se detecta plagio o cualquier otra conducta incorrecta, la Comisión Ejecutiva seguirá las orientaciones de la Guía COPE para estos casos. 

?	También se seguirán las indicaciones de la Guía COPE en caso de que la Revista conozca cualquier alegación de mala conducta relacionada con un artículo publicado en la misma.

E) Derechos de autor:

El autor, de conformidad con las Condiciones Generales del Contrato de Colaboración, cederá, por medio del contrato que suscribe con la Revista al presentar un trabajo a publicación (enlace), todos los derechos de propiedad intelectual, industrial, de imagen o de cualquier otra índole sobre los trabajos que finalmente se publiquen en la Revista. 

En dichas Condiciones Generales y en el contrato suscrito se especifican los derechos económicos que, en su caso, devengará el colaborador.

F)  Acceso a los contenidos de la Revista:

La Revista es accesible a todos los suscriptores, según la modalidad de contrato que suscriban. Las diferentes modalidades de contrato de suscripción y sus respectivos precios aparecen publicadas en la web, junto con el texto de las Condiciones Generales del Contrato de Suscripción (enlace).

G) Fuentes de ingresos de la Revista:

Las tarifas pagadas por los suscriptores serán la principal fuente de ingresos. La Revista no contiene publicidad.

H) Servicio de atención al público:

La Revista, a través de su Secretaría, atenderá y resolverá las quejas, reclamaciones o sugerencias presentadas por los autores, suscriptores o público en general sobre cualquier aspecto relacionado con el cumplimiento del presente Código de Buenas Prácticas. 
