# Supplementary material for: Peer Review in Law Journals
Source: Front Res Metr Anal. 2021 Dec 8;6:787768. doi: 10.3389/frma.2021.787768 (PMC8692876; doi:10.3389/frma.2021.787768)
Supplement: Supplementary file 3 [file DataSheet2.ZIP › DOCUMENT - 0210-1173_1.RTF]

AIMS AND SCOPE

Public Economics is one of the main focus areas on both theoretical and applied economic research. Topics traditionally covered by «Public Finance» have expanded as new issues arise relating to the behaviour of the public sector and its effects on the other economic agents from a very wide perspective. Thus, new areas of study related to the economic analysis of Government intervention have been consolidated. This is the case of the economic analysis of public management, environmental economics, public choice, transport economics, regulation economics, health economics, institutional economics and the «new economic history», among other areas. There have been important changes in relation to the methodology implemented in the analysis such as the generalised use of mathematical formulation and statistical and quantitative techniques for the empirical test of theoretical hypotheses. The availability of new and better-designed databases (particularly microeconomic databases) has also played an important role in this methodological development.

In view of the aforementioned, the Spanish Institute for Fiscal Studies (Instituto de Estu-dios Fiscales), as a benchmark for research on Public Economics, implemented a process of reflection on the need for a periodical publication of a professional and academic nature.This process was concluded with a new phase for the journal Hacienda Pública Española/Review of Public Economics, a meeting point for many Spanish economists for over three decades. The modernisation of this publication has been driven by the need for the diffusion of results to conform to the usual standards required by prestigious journals, both national and foreign.

With an emphasis on the excellence of the research on Economics, Hacienda Pública Es-pañola/Review of Public Economics adopts an editorial guideline based on four major pillars:

First, the acceptance of an anonymous assessment performed by expert and independent referees as the unique criterion for selecting papers to be published by the Journal.

The editorial management is the second pillar. Hacienda Pública Española/Review of Public Economics is managed by a Council of Management consisting of an Editorial Board and an Advisory Committee. The executive management is carried out by the Editorial Board, composed of a maximum of three Executive Editors and five Associate Editors. The Editorial Board shall be responsible for keeping the editorial line of the Journal and for adequately running the manuscript selection process. The Advisory Committee is in charge of supporting and advising the Editorial Board in academic issues. In addition, the Journal has an Academic Secretariat. All members in these bodies are chosen in accordance with the following qualities: research skills, proved and acknowledged by their active and regular participation in the assessment processes of leading national and foreign publications; experience in managing and editorial tasks in other scholarly journals; and full availability in the performance of their duties. All these positions shall be subject to periodic renewal process.

The third pillar is related to the structure of contents. Without compromising the open and inclusive view when selecting those manuscripts submitted, the Journal has introduced two new sections. Under the heading Survey Reviews, the Journal publishes original collaborations containing reviews of the literature regarding issues of interest in the Public Econom-


ics field. In addition, the Journal has a section called Policy Watch which includes original studies aimed at showing the current contributions of Public Economics in relation to contemporary and relevant issues faced by public decision-makers in the real world. For these two sections, the Editorial Board will receive originals from authors and in addition will be able to commission papers on specific subjects. However, in both cases the publication of originals is subject to the same assessment process as any other contribution.

From June 2018, the Journal will be accepting Comments to previously published articles. Contributions to this section should be limited to a maximum of 2.000 words (12 pages). The adequacy of Comments submitted for publication will be evaluated by the Executive­ Editors. If deemed adequate, the authors of the commented article will be given the opportunity to react in a Reply. Both Comment and Reply will be published together.

Lastly, the Journal faces all these challenges with a more up-to-date format in keeping with the usual standards in publications of this type.

Following these principles Hacienda Pública Española /Review of Public Economics is willing to receive manuscripts in Spanish or –preferably– English dealing with some of the many areas included under the heading «Public Economics». Scientific rigour and search for academic excellence will be the only criteria when selecting papers as the Journal is aware that the appreciation of the readers will be earned in this way.

Editorial board
