# Supplementary material for: Peer Review in Law Journals
Source: Front Res Metr Anal. 2021 Dec 8;6:787768. doi: 10.3389/frma.2021.787768 (PMC8692876; doi:10.3389/frma.2021.787768)
Supplement: Supplementary file 3 [file DataSheet2.ZIP › DOCUMENT - 0210-7716_1.RTF]

About the Journal
Focus and Scope
Is an annual publication that, as the title indicates, includes articles covering the areas of history, institutions and the edition of manuscripts, with particular reference to the period previous to the disintegration of the Ancien Régime.
Peer review process
The journal's Editorial Board, once it has checked that the article complies with the rules regarding style and content indicated in the guidelines for authors, will send the article to two anonymous expert reviewers who are not part of the Editorial Board, according to the double-blind model.
The evaluation will affect the interest of the article, its contribution to the knowledge of the subject matter, the novelties provided, the correct relations established, the critical judgment developed, the bibliographical references handled, its correct writing, etc., indicating recommendations, if any, for its possible improvement.
Based on the reviewers' recommendations, the journal's director will communicate the reasoned result of the evaluation to the authors by e-mail, at the address they used to send the article. The director will communicate to the main author the result of the review (publication without changes; publication with minor corrections; publication with major corrections; not recommended for publication), as well as the observations and comments of the reviewers.
If the manuscript has been accepted with modifications, the authors will have to resubmit a new version of the article, taking into account the demands and suggestions of the external evaluators. If they wish, the authors can also provide a letter to the Editorial Board in which they will indicate the content of the modifications to the article. Articles with important corrections may be sent to the Advisory Board to verify the validity of the modifications made by the author.
Depending on the degree of compliance with the requested modifications, the Advisory Board will decide whether or not to publish the article. This decision will be communicated to the author by the director of the journal
Open access policy
This journal provides open access to its content, based on the principle that providing the public with free access to research supports greater global knowledge sharing.
Publication Costs
This journal does not charge authors for the presentation and processing of articles.
Journal History
The first volume of Historia. Instituciones. Documentos. was published in 1974. As is so often the case, it began as an adventure undertaken with enthusiasm by three different departments, under the guidance of three eminent professors – Prof. José Martínez Gijón, Prof. Luis Núñez Contreras and Prof. Julio Valdeón Baruque – and with the collaboration of an enthusiastic group of young researchers who were at the time initiating their academic careers.
The journal began as, and has continued to be a venue for the publication of local, national and international research. Historia. Instituciones. Documentos. is an annual publication that, as the title indicates, includes articles covering the areas of history, institutions and the edition of manuscripts, with particular reference to the period previous to the disintegration of the Ancien Régime.
