# Supplementary material for: Peer Review in Law Journals
Source: Front Res Metr Anal. 2021 Dec 8;6:787768. doi: 10.3389/frma.2021.787768 (PMC8692876; doi:10.3389/frma.2021.787768)
Supplement: Supplementary file 3 [file DataSheet2.ZIP › DOCUMENT - 0210-7716_2.RTF]

Article Submission Guidelines
1. Submission of manuscripts, peer reviews and publication.
 
- Historia. Instituciones. Documentos. is an anual journal. Manuscripts submitted for publication must deal with issues relating to the Ancien Régime and must be original and unpublished. Authors will guarantee that they have not been submitted elsewhere for evaluation.
- Manuscripts submitted will not exceed 80,000 characters, including spaces and notes.
- Manuscripts may be submitted throughout the year and should be sent by email to the Editor or the Secretary of the journal. They will be accompanied by a covering letter indicating the name of the author, or authors, an email address, institutional affiliation, postal address, telephone number and, if so deemed pertinent, a personal postal address.
- The Editors and Editing Board of the journal reserve the right to reject a manuscript for evaluation if, for editorial reasons, incorrect format or inadequate standard, they deem it to be unsuitable. Authors will be informed of any such decision within a maximum period of two months.
- Articles accepted for consideration will be subjected to a process of peer reviewing. Each paper will be submitted to a minimum of two double-blind evaluations by external experts in the field who are not members of the Editing Board. Authors will be notified of the acceptance or rejection of their articles within a maximum period of six months.
- Once articles have been accepted for publication, authors must undertake to revise them and introduce any modifications that may have been suggested by the reviewers. They must also format their manuscripts in accordance with the publication guidelines of the journal. Failure to do so may imply the return of the manuscript to the author until such time as the pertinent corrections are made.
- Authors will correct the first galley proofs but will not introduce significant changes in the text. Corrections will be confined to misspellings, typographical errors, or to the updating of necessary data, without affecting the format of the text.
- Authors will receive one offprint and one copy of the journal in PDF format.
 
2. Format of manuscripts.
            - The title of the manuscript will appear both in Spanish and English.
            - The author’s name will appear under the title, at the right-hand side of the page. Name and surname(s) will appear on the first line in small capitals. The name of the institution of affiliation will appear on the second line in Roman type. The author’s email address will appear on the third line in Roman type.
            - Texts will be preceded by an abstract in Spanish of 80 to 150 words, together with a translation in English or French. Manuscripts in English or French will be accompanied by the corresponding Spanish translation of the abstract.
            - Texts will also include keywords in Spanish together with their translation in English or French. Manuscripts in English or French will be accompanied by the corresponding Spanish translation. Between 5 and 7 keywords are recommended. They will be written in lower case, separated by semicolons and with a final full stop.
            - Articles will be followed by a detailed bibliography of the works consulted by the authors.
            - The various sections of the article will be headed in small capitals. Subsections will be written in bold. Both sections and subsections will be numbered in Arabic numerals. Roman numerals and letters will not be used.
            - Text citations within the article will be written in italics and, if they exceed two lines in length, will be written in smaller type, centred and with double indents. In the footnotes, text citations will appear between quotation marks and in Roman type.
 
Documentary appendices
- Documentary appendices must comply with the journal’s guidelines for transcriptions and edition of documents. (See below)
 
 
Illustrations, maps and tables
            - Illustrations, maps and tables will be submitted by separate file, in .tiff or .jpg format. They will be peer reviewed. The Editorial Board reserves the right to accept or reject them.
            - All illustrations, maps and tables must include a caption or identifying title and must be numbered in Arabic numerals.
            - Authors are responsible for all necessary permissions relating to the above. The Journal will be exempted from all liability.
 
Abbreviations
            - All abbreviations must be included in the first note following the title, with the heading ‘Abbreviations used’. The abbreviations used in the text and footnotes will be presented in alphabetical order and will be separated by semicolons. The abbreviation will be followed by the symbol = plus an explanation of what it represents.
            - All full stops will be eliminated (e.g., AHN, not A.H.N.).
            - Archive sources will be written in Roman type.
            - The abbreviations for ‘folio(s)’ will be f. / ff.
            - Citations from original folios will include ‘recto’ or ‘verso’, using the abbreviation ‘r’, ‘v’ or ‘r-v’ after the folio number, without spaces or punctuation marks. In the case of there being more than one page, a hyphen will be used (e.g., f. 17r, f. 17v, f. 17r-v, ff. 17v-18r).
            - The abbreviations for ‘page(s)’ will be p. / pp. and when necessary ‘and following’ or ‘et seq’.
 
Cross-references
            -Cross-references can be indicated using ‘see’, vid., cf., id. ibid., ‘above’ and ‘below’.
 
Footnotes
            - In the text, reference numbers to footnotes, in superscript, will be placed before punctuation marks.
 
Bibliographical citations in footnotes
            - The bibliographical citations in footnotes will indicate, in Roman type, the author’s surname(s), the year of publication and, if relevant, the page number(s). If more than one work by the same author is cited, the year of publication of each work will be separated by a comma, unless page numbers are included, in which case, semicolons will be used. However, works by different authors will always be separated by semicolons. When citing several works by the same author, published in the same year, lower case letters will be used.
            - When a work has up to three authors, their surnames will be separated by commas. When there are more than three authors, only the surname (s) of the first author will appear followed by a comma and et al, in italics.
            -  Citations in the footnotes will refer exclusively to page numbers in books or articles. Full information regarding the source will be reserved for the final detailed bibliography at the end of the article.
            - For publications that comprise more then one volume, the number of the volume will be indicated in Roman numerals (e.g., vol. II). If, in addition, a tome number has to be added, it will be written in Arabic numerals preceded by a hyphen (e.g., vol. II-1).
            - When the name of the editor, coordinator, director etc. is included, it will come first, similarly to that of the author.
 
Examples of citations in footnotes
Valdeón Baruque 1969.
Valdeón Baruque 1969, pp. 125-150.
Valdeón Baruque 1969, 1980.
Valdeón Baruque 1970a, 1970b.
Valdeón Baruque 1969; 1980, p. 45.
Valdeón Baruque 1969, pp. 75-80; 1980.
Valdeón Baruque 1969, p. 51; Díaz y Díaz 1989, p. 50.
 
                                            
 
Bibliography
- All bibliographical references must appear in a final section at the end of the manuscript, entitled ‘Bibliography’ which, like other sections and in accordance with the guidelines, will be numbered in Arabic numerals.
- This bibliography will include only references cited in the footnotes. It will be presented in the alphabetical order of the authors’ surnames. When several works by the same are being cited, they will appear in chronological order, beginning with the earliest and ending with the most recent. In the case of several works by the same author being published in the same year, these will come in alphabetical order of title, and will be followed, after the year, by a letter in lower case. Each entry will include in full the surname(s) and name of the author and will not use idem or dashes. Should the article have a DOI, this will be included.
- The surname(s) of the author(s) will be written in Roman script, followed by a comma, their name in full, also in Roman script, the year of publication of the work in brackets, followed by a comma. If there is more than one author, their names will be separated by semicolons. Unlike the footnotes, the detailed bibliography will include the full names and surnames of all the authors and will not use et al.
- Monographs will indicate the author’s name, the year of publication in brackets, followed by a comma, the title in italics, followed by a comma and the place of publication.
- Book chapters will indicate the author’s name, the date of publication in brackets, followed by a comma, the title between quotation marks, followed by a comma. Then will follow a complete bibliographical reference that will include the author’s name, title of the book, place of publication, volume number in Roman script if relevant, and the corresponding pages.
- When reference is made to an article, the author’s name will be written in Roman script, followed by the year of publication in brackets, the title between quotation marks, a comma, the name of the journal in italics, the volume number in Arabic numerals, followed by a comma, and the page numbers of the article. Fascicules will come immediately after the volume number, preceded by a slash, without spaces.
- When ‘grey’ literature is being cited, i.e., Ph.D. theses, reports, memoranda, etc., the same procedure as for general bibliography will be followed. For Ph.D. theses, the author’s name will be given with the year of defence in brackets, followed by a comma, the title of the thesis, a comma, the academic institution where the thesis was defended, a comma, and finally Ph.D., or doctoral thesis, in brackets.
 
Examples of bibliographical reference
Valdeón Baruque, Julio (1969), Los judíos de Castilla y la revolución Trastámara, Valladolid.
Díaz y Díaz, Manuel Cecilio (1989), “El testamento monástico de San Rosendo”, Historia. Instituciones. Documentos, 16, pp. 47-102.
 
Referencing of electronic documents
- When citing electronic documents that have a DOI (Digital Object Identifier), this identifier will be used in preference to a URL address, without indicating the date of consultation.
- When citing electronic documents that do not have a DOI, the URL address will be used, and the date of consultation will be indicated in square brackets [consultation: dd/mm/yyyy].
 
Guidelines for the Transcription and Edition of documents
- The Editing Board of the journal has established guidelines for the transcription and edition of historical documents, following the criteria established, and accepted internationally, in 1984 by the International Diplomatics Commission.
- The original spelling must always be respected. In the case of misspellings or obvious errors, these will be indicated in a footnote, with the word ‘sic’.
- All abbreviations will be written in full.
- Double consonants will be eliminated only if they occur at the beginning of a word but not if they come within the word itself.
- If the text is written in Castilian and the sigmatic ‘s’ (ç) is used, it should be transcribed as an ‘s’ or ‘z’ depending on whether the present-day sound in Spanish is a voiceless alveolar fricative (s) or a voiceless interdental fricative (z).
- Modern punctuation uses should be followed, and the same criteria should be used for accents.
- Interpolations must be marked with < > brackets and must come exactly where they appear in the original document.
- Gaps in the text will be marked with square brackets [ ]. If reconstruction of the text is possible, the syllables or words will be placed between the square brackets. If reconstruction is not possible, three dots will be written between the brackets [ … ].
- To facilitate the locating of words or phrases within the document, it is necessary to add numbers to the text. If the document is written on parchment or paper in horizontal format, line changes are usually marked by a simple slash ( / ) and a corresponding superscript line number (e.g., /6). On the other hand, if the document is written on parchment or paper in folio format and exceeds one page, it will not be necessary to mark or number the lines. In order to indicate the change of folio, it will be sufficient to use a double slash ( // ) together with the folio number, indicating whether it is ‘recto’ or ‘verso’. (e.g., //1v or //9r.)
-Any other incident should be referred to in a footnote.
 
Correspondence, Subscriptions, and Exchanges
- Scientific correspondence should be addressed to the Secretary of the Journal.  Email: hidsecretaria@us.es
-  Orders may be placed with Editorial Universidad de Sevilla, C/ Porvenir, 27. 41013 Sevilla. Email: eus6@us.es
