# Supplementary material for: Peer Review in Law Journals
Source: Front Res Metr Anal. 2021 Dec 8;6:787768. doi: 10.3389/frma.2021.787768 (PMC8692876; doi:10.3389/frma.2021.787768)
Supplement: Supplementary file 3 [file DataSheet2.ZIP › DOCUMENT - 0210-8364_1.RTF]

Focus and Scope
Sociología del Trabajo is an independent and plural academic journal that aims to disseminate research and reflections on the reality of work, together with the critical analysis of research on it.
Peer Review Evaluation Process
The acceptances of articles is based upon a system of external peer reviews.
The original works received are, in the first place, read by the Editorial Board in order to evaluate if they fulfill both the minimum formal requirements indicated and the adaptation to the guidelines and objectives of the journal.
When this evaluation is passed satisfactorily, articles will be sent to be evaluated by, at least, two external reviewers. With their evaluation the Editorial Board will procede to sent back to the author the comments and suggestions expressed by the reviewers, as well as the list of of modifications or rewrittings if such are needed, required for the work's acceptance for publication. In the case of requiring major changes, articles will be evaluated again by two external reviewers and by one member from the Editorial Board before their eventual publication. This process is double blind peer review. The journal will notify authors by email the reception of the original manuscript and their return, the results of the evaluation, the need of modifications, as well as the acceptance for publication. The journal will notify authors of the acceptance for publication in a period inferior to three months (since the results of the evaluation are notified), as well as a preliminary date for the issue's publication.
Publication Frequency
Biannual (from 2018 onwards; quarterly up to 2017)
Open Access Policy
This is an open access journal which means that all content is freely available without charge to the user or his/her institution. Users are allowed to read, download, copy, distribute, print, search, or link to the full texts of the articles, or use them for any other lawful purpose, without asking prior permission from the publisher or the author. This is in accordance with the BOAI definition of open access.
Sociología del Trabajo is an open access journal that does not charge authors for article processing (submission, review or editing) or publication.
Interoperability protocols
Sociología del Trabajo provides an interface OAI-PMH (Open Archives Initiative – Protocol for Metadata Harvesting) that enables interoperability between different platforms and repositories through the exchange of metadata.
Protocol: OAI-PMH Version 2.0
Metadata formats: Dublin Core Metadata; MARC; MARC21; RFC1807
URL for harvesters: https://revistas.ucm.es/index.php/STRA/oai
Anti-plagiarism policy
Sociología del Trabajo guarantees the originality of all the submitted manuscripts through the use of anti-plagiarism software provided by Ediciones Complutense. This policy ensures the appropriate originality standards as well as the detection of coincidences and similarities between texts sent for publication and those published previously in other sources. In the event of plagiarism, the manuscript will be rejected.
