# Supplementary material for: Peer Review in Law Journals
Source: Front Res Metr Anal. 2021 Dec 8;6:787768. doi: 10.3389/frma.2021.787768 (PMC8692876; doi:10.3389/frma.2021.787768)
Supplement: Supplementary file 3 [file DataSheet2.ZIP › DOCUMENT - 0211-5743_1.RTF]

About the journal
The aim of the Revista Española de Derecho Constitucional (REDC) is to disseminate previously-unpublished academic work in the fields of Constitutional Law, State Theory and Constitutional History.
Submissions
The original papers to the REDC should be sent by email to evaluaciones@cepc.es. Papers must be previously unpublished in any language. The author will be responsible for informing the REDC of the situation regarding copyright. For the purposes of the stipulations laid down in the Ley de Propiedad Intelectual concerning the actions and proceedings that may be brought against those who infringe intellectual property rights, the author will be considered liable for the publication of a paper that infringes said rights.
The works must be anonymized, avoiding any references that may allow the identification of authorship. Bibliographical references to works by the author should be omitted at this stage of presentation of the manuscript. If published, these references will be added in the final version.
Submission of a manuscript to the REDC implies having read and accepted the journal's editorial guidelines and instructions for authors. While the manuscript is in the process of being assessed by the REDC, the author will not submit it to other journals for assessment.
Languages of original articles
REDC accepts articles written in Spanish and English, and publishes them in the original language. Articles submitted in English must have a level of quality equivalent to having been written or revised by a native English speaker.
Submissions format
Submitted texts must use the Times New Roman font, size 12, with a line spacing of 1.5. The only exception is footnotes, which should be in size 10 with single line spacing. They will have a maximum length of 12,000 words, including the title, abstracts, keywords, tables, bibliography and footnotes. The first page will include the title, name of the author or authors, academic affiliation, ordinary and electronic mail addresses and contact telephone number, as well as the indication of who is the recipient of all communications with the journal.
Title
The title of “studies” and “research notes” should appear first in Spanish and then in English.  It should reflect the content of the work. As far as possible, the use of abbreviations and acronyms should be avoided.
Abstract
Articles should be prefaced by a brief, one version in Spanish and another in English, both with the same content. For the articles, the extension of the Spanish version must be between 120 and 150 words. Information that does not appear in the text of the article can not be included in the same. Unwanted words or expressions, periphrasis, and excessively long phrases should also be avoided.
Keywords
The abstract should be followed by a list of between 5 and 10 keywords in Spanish and the corresponding translation into English. In order to favor their normalization, it is recommended that they will be extracted from thesaurus and standardized vocabularies.
Peer Review Process
REDC will acknowledge receipt of all originals. The Editorial Board will decide on the publication of articles on the basis of two double-blind peer review reports −except for justified exceptions− provided by specialists from outside the editorial organization of the journal. The journal undertakes to complete the evaluation process within a maximum period of six months. However, if the author has not received a statement from the journal in a year, the publication shall be deemed rejected.
The Editorial Board approves the publication of those articles receiving the best peer reviews. Publication may be contingent on the introduction of changes recommended by the evaluations. The authors of articles accepted for publication may be requested to correct article proofs, which should be returned within 48 hours. At this stage substantial changes in the text will not be allowed, only the correction of errors in the accepted version.
Citation formats
References cited
Citations will preferably appear in the body of the text and not in footnotes. References will be abbreviated according to the Harvard author-year system: (author, year: page(s)-).
(Rodríguez Bereijo, 2012: 72)
Where various articles of an author, or group of authors, from the same year are cited, a letter (a, b, c, etc.) must be added after the year:
 (Mercader Uguina, 2003a)
References with two authors will be cited using primary surnames and the conjunction ‘and’:
(Telles and Ortiz, 2011)
Where work with three or more authors is cited, it is sufficient to cite the first author followed by et al.:
 (Amador et al.,  1989)
Where a cited author’s name forms part of the text of the footnote, the year and page number(s) must always be included in parentheses:
According to Sesma Sánchez (2005)…
Notes
Notes shall be placed at the foot of the page (footnotes), numbered using Arabic characters in superscript format. Footnotes should only contain brief clarifications that are not of sufficient importance to be placed within the main body of the text. Bibliographical citations could be included in footnotes. In no case will this be the complete bibliographical reference, which will be included in the final article Bibliography.
Bibliography
A Bibliography will be included at the end of all articles. The Bibliography will only contain references cited in the text. Additional references may not be included. Items will be listed alphabetically by the author’s first surname, following the American Psychological Association (APA) style. If two or more works of an author from the same year are included, they shall be distinguished by means of a letter following the year.
Maravall, J. A. (1966a). Antiguos y modernos: la idea de progreso en el desarrollo inicial de una sociedad. Madrid: Sociedad de Estudios y Publicaciones.
Maravall, J. A. (1966b). De la Ilustración al Romanticismo: el pensamiento político de Cadalso. Paris: Centre de Recherches de L'Institut d'Etudes Hispaniques.
Where a reissued work is cited and the date of the first edition is considered relevant, the original year shall be indicated in brackets:
Maravall, J. A. (1981) [1963]. Las comunidades de Castilla: una primera revolución moderna. Madrid: Alianza Editorial.
Below are bibliographic examples for the most common document types:
Scientific journal articles
García de Enterría, E. (1989). Un paso importante para el desarrollo de nuestra justicia constitucional: la doctrina prospectiva en la declaración de ineficacia de las leyes inconstitucionales. Revista Española de Derecho Administrativo, 61, 5-18.
−Digital/online journals
Pifarré, M. J. (2013). Internet y redes sociales: un nuevo contexto para el delito. IDP. Revista de Internet, Derecho y Política, 16, 40-43. Disponible en: http://www.redalyc.org/articulo.oa?id=7882 8864004.
- Digital/online journals with DOI
Díaz-Noci, J. (2010). Medios de comunicación en internet: algunas tendencias. El Profesional de la Información, 19 (6), 561-567. Retrieved from: http://dx.doi.org/10.3145/epi.2010.nov.01.
Books
-Single author
García Ruiz, J. L. (1994). El Consejo Económico y Social. Génesis constituyente y parlamentaria. Madrid: Consejo Económico y Social.
-Two or more authors
Anduiza Perea, E., Crespo, I. y Méndez Lago, M. (1999). Metodología de la Ciencia Política. Madrid: Centro de Investigaciones Sociológicas.
-Digital/online book
Rodríguez de Santiago, J. M. (2015). Responsabilidad del Estado legislador por leyes inconstitucionales o contrarias al Derecho Europeo. Retrieved from: http://almacendederecho.org/responsabilidad-del-estado-por-leyes-inconstitucionales-o-contrarias-al-derecho-europeo/.
Book chapters
Zea, L. (2007). América Latina: largo viaje hacia sí misma. In D. Pantoja (ed.). Antología del pensamiento latinoamericano sobre la educación, la cultura y las universidades (pp. 125–138). México: UDUAL.
Reports
-Institutional author
Instituto para la Diversificación y Ahorro de la Energía. (2004). Eficiencia energética y energías renovables (Informes IDEA. Boletín IDEA; 6). Madrid: IDEA.
-Personal author
Caruso, J., Nicol, A. y Archambault, E. (2013). Open Access Strategies in the European Research Area. Montreal: Science-Metrix. Retrieved from:  http://www.science-metrix.com/pdf/SM_EC_OA_Policies.pdf.
 Conferences
- Proceedings
Cairo, H. and Finkel, L. (eds.). (2013). Actas del XI Congreso Español de Sociología: crisis y cambio. Propuestas desde la Sociología. Madrid: Federación Española de Sociología.
- Papers published in Conference Proceedings
Codina Bonilla, L. (2000). Parámetros e indicadores de calidad para la evaluación de recursos digitales. In Actas de las VII Jornadas Españolas de Documentación (Bilbao, 19-21 de octubre de 2000): la gestión del conocimiento: retos y soluciones de los profesionales de la información (pp. 135-144). Bilbao: Universidad del País Vasco.
- Papers and communications online
Durán Heras, M. A. (2014). Mujeres y hombres ante la situación de dependencia. Paper presented at the seminar: Políticas públicas de atención a personas mayores dependientes: hacia un sistema integral de cuidados. Retrieved from: http://www.imserso.es/InterPresent2/groups/imserso/documents/binario/mujereshombres19_21mayo.pdf.
 
Thesis
- Published
Llamas Cascón, A. (1991). Los valores jurídicos como ordenamiento material [PhD thesis]. Universidad Carlos III de Madrid. Retrieved from: http://hdl.handle.net/10016/15829.
- Unpublished
De las Heras, B. (2011). Imagen de la mujer en el Fondo Fotográfico de las Guerra Civil Española de la Biblioteca Nacional de España. Madrid, 1936-1939 [Unpublished PhD thesis]. Universidad Carlos III de Madrid.
News and articles of newspaper
Bassets, M. (2015). El Tribunal Supremo respalda la reforma sanitaria de Obama. El País, 25-6-2015. Retrieved from: http://internacional.elpais.com/internacional/2015/06/25/actualidad/1435242145_474489.html.
-Without author
Drogas genéricas. (2010). El Tiempo, 25-9-2015, p. 15.
Blogs
Escolar, I. (2015). El falso mito de que los emprendedores de Internet son la solución al paro.  Escolar.Net [blog], 25-6-2015. Retrieved from: http://www.eldiario.es/escolar/falso-empresas-Internet-solucion-paro_6_402519746.html.
Electronic forums and mailing lists
Pastor, J. A. (2014). Aspectos prácticos para proyectos de datos abiertos en las administraciones públicas. IWETEL [lista de distribución], 31-1-2014. Retrieved from en: http://listserv.rediris.es/cgi-bin/wa?A2=IWETEL;b361930a.1401e.
DOIS
If the bibliographical references contain doi (digital object identifier), it will be included as follows:
Murray, S. (2006). Private Polls and Presidential Policymaking. Reagan as a Facilitator of Change. Public Opinion Quarterly, 70 (4), 477-498. Available in: http://dx.doi.org/10.1093/poq/nfl022.
Copyright Notice
Submission of a manuscript to the REDC implies having read and accepted the journal's editorial guidelines and instructions for authors. When a work is accepted for publication, it is understood that the author grants the REDC exclusive rights of reproduction, distribution and, where appropriate, sale of his manuscript for exploitation in all countries of the world in printed version, as well as any other magnetic, optical and digital media.
Authors shall transfer the publishing rights of their manuscript to REDC so that it may be disseminated and capitalised on Intranets, the Internet and any web portals and wireless devices that the publisher may decide, by placing it at the disposal of users so that the latter may consult it online and extract content from it, print it and/or download and save it. These activities must comply with the terms and conditions outlined on the website hosting the work. However, the REDC authorises authors of papers published in the journal to include a copy of these papers, once published, on their personal websites and/or other open access digital repositories. Copies must include a specific mention of REDC, citing the year and issue of the journal in which the article was published, and adding a link to the REDC website(s).
A year after its publication, the works of the REDC will be under the Creative Commons Attribution-Noncommercial-NoDerivative 4.0 International license (CC BY-NC-ND 4.0), which allows third parties to share the work as long as its author and its first publication is indicated, without the right to commercial exploitation and the elaboration of derivative works.
Plagiarism and scientific fraud
 
 The publication of work that infringes on intellectual property rights is the sole responsibility of the authors, including any conflicts that may occur regarding infringement of copyright. This includes, most importantly, conflicts related to the commission of plagiarism and/or scientific fraud.
Plagiarism is understood to include:
a. Presenting the work of others as your own.
b. Adopting words or ideas from other authors without due recognition.
c. Not using quotation marks or another distinctive format to distinguish literal quotations.
d. Giving incorrect information about the true source of a citation.
e. The paraphrasing of a source without mentioning the source.
f. Excessive paraphrasing, even if the source is mentioned.
 
Practices constituting scientific fraud are as follows:
a. Fabrication, falsification or omission of data and plagiarism.
b. Duplicate publication.
c. Conflicts of authorship.
