# Supplementary material for: Peer Review in Law Journals
Source: Front Res Metr Anal. 2021 Dec 8;6:787768. doi: 10.3389/frma.2021.787768 (PMC8692876; doi:10.3389/frma.2021.787768)
Supplement: Supplementary file 3 [file DataSheet2.ZIP › DOCUMENT - 0211-979X_1.RTF]

Sobre la revista
Enfoque y alcance
La Revista de Derecho Político publica trabajos de investigación originales sobre Derecho Constitucional, Derecho Público, Teoría del Estado e Historia del constitucionalismo.


The Revista de Derecho Político has been published every four months by the Department of Constitutional Law at the National University of Education at Distance (UNED; Spain) since 1978. With 100 issues published, the Journal specializes in Constitutional Law matters, along with issues related to Public Law, EU law, constitutional history and theory of the state. In addition, the Revista de Derecho Político is one of the four Spanish journal dealing with Legal Science which passed the evaluation process for academic journals undertaken by the Spanish Foundation for Science and Technology (FECYT). Besides, it is included in Scopus and in the Catálogo de Latindex where deserved the highest rating: 33/33 quality factors, and is also included in Index Copernicus.
Proceso de evaluación por pares
?	Se acusará recibo de todos los trabajos recibidos. El Consejo de Redacción decidirá, en los siguientes cuatro meses a dicho acuse de recibo, la publicación o no de los trabajos, basándose en informes de al menos dos evaluadores externos y anónimos, que seguirán el método de "doble ciego". Para la elaboración de los informes, los evaluadores cuentan con un formulario elaborado por el Consejo de Redacción de la Revista. La decisión sobre la publicación se le comunicará al autor y, en caso afirmativo, se le indicará el número de la Revista de Derecho Político en el que se incluirá el trabajo. La publicación podrá quedar condicionada a la introducción de cambios con respecto a la versión original, en especial a la adaptación a las normas de publicación.
?	Los artículos que sean devueltos por no cumplir con las normas de publicación podrán ser reenviados de nuevo una vez hechas las oportunas modificaciones.
?	Es condición para la publicación que el autor o autores ceda(n) a la Revista de Derecho Político, en exclusiva, los derechos de reproducción. Si se producen peticiones de terceros para reproducir o traducir artículos o partes de los mismos, la decisión corresponderá al Consejo de Redacción.
Frecuencia de publicación
La periodicidad es de tres números al año, uno al cuatrimestre.

Política de acceso abierto
Desde el año 2014, esta revista provee acceso libre a su contenido, lo cual fomenta un mayor intercambio de conocimiento global.
La Revista de Derecho Político no cobra tasas por el envío de trabajos, ni tampoco cuotas por la publicación de sus artículos.
La Revista de Derecho Político es gratuita desde el momento de la publicación de cada número y sus contenidos se distribuyen con la licencia Creatve Commons, que permite al usuario el acceso libre y en abierto,criterios que cumplen con la definición de open access de la Declaración de Budapest  en favor del acceso abierto.
Publications Ethics and malpractice Statement (Declaración sobre ética y buenas prácticas)
Revista de Derecho Político
Publications Ethics and malpractice Statement
A: Publication and authorship
1.  The submitted papers must be original and unpublished. The journal will use anti-plagiarism tools to ensure this.
2. All submitted papers are subject to strict peer-review process by at least two national and international reviewers that are experts in the area of the particular paper.
3. The factors that are taken into account in review are relevance, soundness, significance, originality, readability and language.
4.   The possible decisions include acceptance, acceptance with revisions, or rejection.
5.   If authors are encouraged to revise and resubmit a submission, there is no guarantee that the revised submission will be accepted.
6.   Rejected articles will not be re-reviewed.
7.   The paper acceptance is constrained by such legal requirements as shall then be in force regarding libel, copyright infringement and plagiarism.
8.   No research can be included in more than one publication.
 
B: Authors' responsibilities
1.   Authors must certify that their manuscripts are their original work.
2.   Authors must certify that the manuscript has not previously been published elsewhere.
3.   Authors must participate in the peer review process.
4.   Authors are obliged to provide retractions or corrections of mistakes.
5.   All Authors must have significantly contributed to the research.
6.   Authors must state that all data in the paper are real and authentic.
7.   Authors must notify the Editors of any conflicts of interest.
8.   Authors must identify all sources used in the creation of their manuscript.
9.   Authors must report any errors they discover in their published paper to the Editors.
 
C: Reviewers' responsibilities
1.   Reviewers should keep all information regarding papers confidential and treat them as privileged information.
2.   Reviews should be conducted objectively, with no personal criticism of the author
3.   Reviewers should express their views clearly with supporting arguments
4.   Reviewers should identify relevant published work that has not been cited by the authors.
5.   Reviewers should also call to the Editor in Chief's attention any substantial similarity or overlap between the manuscript under consideration and any other published paper of which they have personal knowledge.
6.   Reviewers should not review manuscripts in which they have conflicts of interest resulting from competitive, collaborative, or other relationships or connections with any of the authors, companies, or institutions connected to the papers.
 
D: Editors' responsibilities
1.   Editors have complete responsibility and authority to reject/accept an article.
2.   Editors are responsible for the contents and overall quality of the publication.
3.   Editors should guarantee the quality of the papers and the integrity of the academic record.
4.   Editors should publish errata pages or make corrections when needed.
5.   Editors should have a clear picture of a research's funding sources.
6.   Editors should base their decisions solely one the papers' importance, originality, clarity and relevance to publication's scope.
7.   Editors should not reverse their decisions nor overturn the ones of previous editors without serious reason.
8.   Editors should preserve the anonymity of reviewers.
9.   Editors should ensure that all research material they publish conforms to internationally accepted ethical guidelines.
10. Edittors should only accept a paper when reasonably certain.
11. Editors should act if they suspect misconduct, whether a paper is published or unpublished, and make all reasonable attempts to persist in obtaining a resolution to the problem.
12. Editors should not reject papers based on suspicions, they should have proof of misconduct.
13. Editors should not allow any conflicts of interest between staff, authors, reviewers and board members.
 
Declaración de ética y buenas prácticas
A: Publicación y autoría
1. Los trabajos presentados deben ser originales e inéditos. La revista utilizará herramientas antiplagio para garantizar este aspecto.
2. Todos los trabajos presentados están sujetos a un proceso de estricta revisión por pares, por al menos dos revisores nacionales e internacionales, expertos en el área específica de la contribución. 
3. Los factores que se tendrán en cuenta en la revisión son: pertinencia, solidez, relevancia, originalidad, legibilidad y manejo del lenguaje.
4. Las decisiones posibles serán: aceptación, aceptación con revisiones o rechazo.
5. Si a los autores se les solicita una revisión y volver a enviar el trabajo, no hay ninguna garantía de que se aceptará la presentación revisada.
6. Los artículos rechazados no volverán a ser objeto de revisión.
7. La aceptación del artículo está limitada por el respeto a los requisitos legales vigentes en materia de difamación, derechos de autor y plagio.
8. Ninguna investigación puede incluirse en más de una publicación.
 
B: Responsabilidades de los autores
1. Los autores deben certificar que sus manuscritos son originales y propios. 
2. Los autores deben certificar que el manuscrito no ha sido publicado previamente en otra parte.
3. Los autores se comprometen a participar en el proceso de revisión por pares.
4. Los autores están obligados a incorporar rectificaciones o correcciones de errores.
5. Todos los autores deben haber contribuido significativamente a la investigación.
6. Los autores deberán indicar que todos los datos que figuran en el documento son reales y auténticos.
7. Los autores deben notificar a los editores de cualquier conflicto de intereses.
8. Los autores deben identificar todas las fuentes utilizadas en la creación de su manuscrito.
9. Los autores deben informar a los editores sobre cualquier error que descubran en el trabajo publicado.
 
C: Responsabilidades del revisor
1. Los revisores deben mantener la confidencialidad sobre toda la información que contengan los trabajos y tratarlos como información privilegiada.
2. Las revisiones deben realizarse objetivamente, absteniéndose de críticas personales al autor.
3. Los revisores deben expresar sus puntos de vista de modo claro y suficientemente argumentado.
4. Los revisores deben identificar los trabajos publicados relevantes que no hayan sido citado por los autores.
5. Los revisores también deberían llamar a atención del Director o Secretario de la Revista sobre cualquier similitud sustancial o coincidencia entre el manuscrito sometido a consideración y cualquier otro artículo publicado del que tengan conocimiento personal.
6. Los revisores no deben revisar los manuscritos sobre los que tengan conflictos de intereses, resultantes de posibles relaciones competitivas, colaboración, u otras relaciones o contactos con alguno de los autores, empresas o instituciones conectadas a los manuscritos sometidos a su consideración.
 
D: Responsabilidades de los editores
1. Los editores tienen toda la responsabilidad y la autoridad para rechazar/aceptar un artículo.
2. Los editores son responsables del contenido y la calidad general de la publicación.
3. Los editores deben garantizar la calidad de los trabajos y la integridad del proceso académico.
4. Los editores deben publicar páginas de erratas o hacer correcciones cuando sea necesario.
5. Los editores deben tener un panorama claro de las fuentes de financiación de la investigación.
6. Los editores deben basar sus decisiones sobre los trabajos únicamente en la importancia, originalidad, claridad y relevancia para el alcance de la publicación.
7. Los editores no deben revertir sus decisiones ni revocar las tomadas por los anteriores editores sin un motivo serio y fundamentado.
8. Los editores deben preservar el anonimato de los revisores.
9. Los editores deben velar por que todo el material de investigación publicado cumple con las pautas éticas aceptadas internacionalmente.
10. Los editores sólo debe aceptar un trabajo cuando exista certeza razonable.
11. Los editores deben actuar si sospechan de mala conducta, relativa a si un trabajo es publicado o no y deben realizar todos los intentos razonables para persistir en la obtención de una solución al problema.
12. Los editores no deben rechazar trabajos basados en la simple sospecha; deberían tener pruebas de mala conducta.
13. Los editores no deben permitir cualquier conflicto de intereses entre el personal, autores, revisores y miembros de la Consejo de Redacción.
 
 
Interoperabilidad
La Revista de Derecho Político tiene protocolos de interoperabilidad que permiten que sea recolectada por otros sistemas de distribución.
Para hacerlo, debe seguirse la siguiente ruta:
 http://revistas.uned.es/index.php/derechopolitico/oai
Sponsors
Departamento de Derecho Constitucional, Facultad de Derecho de la UNED
0.	Departamento de Derecho Constitucional, UNED
Historial de la revista
La Revista de Derecho Político se publica por parte del Departamento de Derecho Constitucional de la Universidad Nacional de Educación a Distancia con una periodicidad cuatrimestral desde 1978. Inicialmente se llamó "Boletín informativo del Departamento de Derecho Político", posteriormente "Revista del Departamento de Derecho Político", hasta que adoptó su denominación actual en 1981. Desde 1978, esta revista, que conmemoró en 2003 su veinticinco aniversario, ha dedicado más de un tercio de su producción a la Constitución española.
En los primeros números (1978-1981) se hizo especial hincapié en temas de historia constitucional, el proceso constituyente e, incluso, en la publicación de repertorios bibliográficos o materiales para su estudio. Junto a números de temática variada, se han publicado también periódicamente números monográficos. En 2003 se editó un número doble, conmemorativo del XXV aniversario de la Constitución Española, en el que participaron un elevado número de los mejores especialistas españoles en Derecho Constitucional. Este número monográfico se presentó en un acto público celebrado en el Círculo de Bellas Artes de Madrid, acto que fue presidido por el entonces Presidente del Tribunal Constitucional, el Excmo. Sr. D. Manuel Jiménez de Parga.Los últimos números monográficos han sido dedicados al Tratado Constitucional Europeo (números 64 Y 65), a una completa selección de los ejercicios de la última habilitación nacional de catedráticos de Derecho Constitucional, celebrada en la Universidad de Murcia en junio de 2007 (número doble 71-72), a un Homenaje a Manuel García Pelayo (número doble 75-76, presentado en unacto homenaje en la Facultad de Derecho de la UNEDen el que intervinieron el Ministro de Justicia, la Presidenta del Tribunal Constitucional y el Presidente del Consejo de Estado), y al bicentenario de la Constitución de Cádiz de 1812 (números 82, 83 y 84).
La revista cumplió 40 años en 2018, igual que la Constitución Española. Con este motivo, los números 100 y 101 fueron monográficos para conmemorar este aniversario.
Junto a las secciones de Estudios y Notas, contiene también unas secciones fijas dedicadas a la Crónica Constitucional (de periodicidad anual), al Derecho Constitucional Europeo, y al Derecho Político Iberoamericano.
La Revista de Derecho Político es una de las cuatro revistas españolas del área de Ciencias Jurídicas que superaron el primer proceso de evaluación de revistas científicas que ha llevado a cabo la Fundación Española de Ciencia y Tecnología (FECYT).
 
