# Supplementary material for: Peer Review in Law Journals
Source: Front Res Metr Anal. 2021 Dec 8;6:787768. doi: 10.3389/frma.2021.787768 (PMC8692876; doi:10.3389/frma.2021.787768)
Supplement: Supplementary file 3 [file DataSheet2.ZIP › DOCUMENT - 0212-5056_1.RTF]

Editorial Policies
?	Focus and Scope
?	Section Policies
?	Experts Review Process
?	Publication Frequency
?	Open Access Policy
?	Journal Digital Archiving Policy
?	Code of ethics
?	Monographic section proposal guidelines
Focus and Scope
La Revista de Llengua i Dret s’ha consolidat com a revista acadèmica de referència en la seva triple especialització: el llenguatge administratiu i jurídic, el dret lingüístic i la política i la planificació lingüístiques, és a dir, com “la revista de la llengua del dret i del dret a la llengua”.
Des dels inicis ha tingut una clara vocació internacional, a través de l’interès per incorporar-hi veus externes que n’enriqueixen el debat i amb la voluntat de difondre internacionalment els estudis que s’hi publiquen, per això s’ha treballat de forma acurada perquè compleixi els criteris de qualitat editorial exigibles a qualsevol publicació científica i augmenti, així, la presència en repositoris i bases de dades de revistes científiques nacionals i internacionals i, per tant, la valoració en les agències qualificadores. És un procés constantment obert que ha donat fruits, com  ha estat, entre d’altres:
`.	La incorporació de la Revista en bases de dades com Dialnet, Raco, Dulcinea o DOAJ, entre moltes d'altres. Consulteu la llista completa.
`.	La incorporació de la Revista a l’Emerging Sources Citation Index (ESCI).
`.	La incorporació de la Revista a la base de dades Scopus d’Elsevier, posicionada en el segon quartil a la categoria de lingüística.
`.	La renovació del segell de qualitat de la Fundació Espanyola per a la Ciència i la Tecnologia (FECYT) (vegeu nota de premsa).
`.	La renovació de la categoria A, la la màxima qualificació, del Carhus+ de l'AGAUR.
 
Section Policies
Essays on administrative and legal language
Editors
0.	Clara Castelló
0.	Cristina Gelpí
0.	Esther Monzó Nebot
0.	Gemma Pauné Xuriguera
0.	Eva Pons Parera
0.	Elvira Riera
0.	Montserrat Serra
0.	Roser Térmens
 Open Submissions	 Indexed	 Peer Reviewed	
Essays on forensic linguistics
Editors
?	Clara Castelló
?	Cristina Gelpí
?	Esther Monzó Nebot
?	Eva Pons Parera
?	Elvira Riera
?	Montserrat Serra
?	Roser Térmens
 Open Submissions	 Indexed	 Peer Reviewed	
Essays on linguistic law
Editors
0.	Clara Castelló
0.	Antoni Milian
0.	Gemma Pauné Xuriguera
0.	Eva Pons Parera
0.	Agustí Pou Pujolràs
0.	Elvira Riera
0.	Montserrat Serra
0.	Joan Ramon Solé i Durany
0.	Roser Térmens
0.	Jaume Vernet
 Open Submissions	 Indexed	 Peer Reviewed	
Essays on language policy and sociolinguistics
Editors
0.	Albert Bastardas
0.	Clara Castelló
0.	Eva Codó
0.	Carles de Rosselló
0.	Gemma Pauné Xuriguera
0.	Eva Pons Parera
0.	Joan Pujolar Cos
0.	Elvira Riera
0.	Montserrat Serra
0.	Roser Térmens
0.	Francesc Xavier Vila
 Open Submissions	 Indexed	 Peer Reviewed	
Legislation, jurisprudence and documentation
Editors
0.	Clara Castelló
0.	Gemma Pauné Xuriguera
0.	Eva Pons Parera
0.	Agustí Pou Pujolràs
0.	Elvira Riera
0.	Montserrat Serra
0.	Joan Ramon Solé i Durany
0.	Roser Térmens
 Open Submissions	 Indexed	 Peer Reviewed	
Notices and information
Editors
0.	Clara Castelló
0.	Gemma Pauné Xuriguera
0.	Eva Pons Parera
0.	Elvira Riera
0.	Montserrat Serra
0.	Roser Térmens
 Open Submissions	 Indexed	 Peer Reviewed	
Book reviews and the bibliographical news
Editors
`.	Gemma Pauné Xuriguera
`.	Eva Pons Parera
`.	Elvira Riera
`.	Montserrat Serra
`.	Joan Ramon Solé i Durany
`.	Roser Térmens
 Open Submissions	 Indexed	 Peer Reviewed	
 
Experts Review Process
The Revista de Llengua I Dret, Journal of Language and Law (RLD) publishes previously unpublished and original articles only. In accordance with widespread international practice, before publication is agreed, all submitted articles are reviewed anonymously by external referees who are experts in the subject matter and alien to the Escola d’Administració Pública de Catalunya and the journal’s Editorial Board. Authors will receive the results of this review and, when appropriate, will be asked to make the changes suggested by the reviewers.

Guaranteeing a blind review
Authors are requested to submit their articles without any personal details as described in the following section.

Ensuring a blind review
To ensure the integrity of the blind peer-review for submission to this journal, every effort should be made to prevent the identity of the author and reviewers from being known to each other. Authors are requested to delete any personal details from the articles (name and surname, email address, academic position, postal address, biographical note). This information is provided separately when submitting an article. Authors should check to see if the following steps have been taken with regard to the text and the file properties:
 
1. The authors of the document have deleted their names from the text, with "Author" and year used in the references and footnotes, instead of the authors' name, article title, etc.
2. With Microsoft Office documents, author identification should also be removed from the properties for the file (see under File in Word), by clicking on the following, beginning with File on the main menu of the Microsoft application: File > Save As > Tools (or Options with a Mac) > Security > Remove personal information from file properties on save > Save.
Personal information will be added to the article in the final stages of the editing process.

Phases in the blind review process
Phase 1: the author submits the manuscript without any personal details
Phase 2: peer reviewers expert in the subject matter review the article.
Phase 3: the reviewers send the result of their review to the Editorial Board so they can make their decision.
Phase 4: authors are notified the result of the review process.
Accept submission: the article is accepted and no changes are required.
Decline submission: the article is rejected when the result of the review process is negative.
Revisions required: the author is requested to make the changes suggested by the reviewers before accepting it for publication.
 
Publication Frequency
The Revista de Llengua i Dret, Journal of Language and Law published biannually, is released in the months of June and December. As from issue number 58, the journal is published in its electronic version only.
 
Open Access Policy
This journal provides immediate open access to its content on the principle that making research freely available to the public supports a greater global exchange of knowledge.
The author assigns the non-exclusive intellectual property rights to the editors, for worldwide use and for the duration of protection of the work, the known forms and uses (digital, data bases…).
All articles in Revista de Llengua i Dret. Journal of Language and Law are published under the Creative Commons Attribution – Non Commercial – No Derivative Works license from 2009. Submission to this journal implies accepting this publishing modality.
 
Journal Digital Archiving Policy
The journal's digital archiving is provided by IBM Global Services.
Daily security backups are performed (one full copy per week, the rest being differential backups)
All the backups are kept in a safe place for 90 days, should recovery be needed.
 
Code of ethics
Code of ethics for the scientific journals of the Public Administration School of Catalonia
The Public Administration School of Catalonia (EAPC) is committed to guaranteeing the ethics and quality of the articles published in its scientific journals, the Catalan Journal of Public Law and the Journal of Language and Law, based on the Code of conduct and best practice guidelines for journal editors of the Committee on Publication Ethics (COPE).               
The EAPC and the editorial committees are responsible for the proper publication of content. They strive to respond to the needs of readers and authors and constantly improve publications. They have processes to assure the quality of the published materials in accordance with the specificity of the different sections. They guarantee freedom of expression, maintain the integrity of academic content and prevent third-party or corporate interests from compromising their ethical and intellectual standards. Likewise, they undertake to publish corrections, clarifications, retractions and apologies in a clear manner when necessary. The School’s journals comply with the regulations on personal data protection, confidentiality and intellectual property. 
Relations with authors
The admission or non-admission of a submission is based on the importance, originality and clarity of the article or contribution, as well as on the validity of the study and its relevance in accordance with the objectives and the mission of the journals. 
The EAPC and the editorial committees publish author guidelines describing everything that is required of them. These guidelines are updated regularly and are linked to this code of ethics. 
The School’s scientific journals undergo a double-blind review process of the studies they publish. The EAPC and the editorial committees publish a description of this process and are willing to justify any deviation from the described process. They are always ready to receive and evaluate possible diverging opinions on behalf of authors and are committed to responding in all cases. 
The journals are open to all those in related academic or professional fields who are interested in publishing an article, although these interested parties must bear in mind the criteria for submitting originals and the established system for selecting articles. In any case, the interpretations expressed by the authors are not necessarily subscribed by the EAPC nor by the editorial committees. 
If new members are appointed to the editorial committee, the authors are guaranteed that the decisions made by previous members will be respected unless serious problems have been identified with the contents of a specific article. 
With respect to preventing possible bad practices, the authors must guarantee that their submitted works are original and unpublished and should avoid both plagiarism and self-plagiarism. In addition, authors must confirm the veracity of the presented data, that is to say, that the empirical data has not been altered to confirm the hypothesis. All authors are responsible for guaranteeing and protecting the confidentiality of the personal data obtained during the research process. Also, if their research has been funded, they must declare the source. 
In the case of shared authorship, the person who appears as the interlocutor of the journal must ensure that the text presented is the result of a consensus of all the authors and that it has not been submitted or previously published elsewhere. 
The editorial committees can directly reject the works received, without resorting to an external review process, if they consider them to be inappropriate because they do not meet the required level of quality, they do not match the scientific or editorial objectives of the journals or they present evidence of bad practices. They also reserve the right to retract articles once they are published if they subsequently demonstrate a lack of reliability either due to involuntary errors or bad practices. If only a part of the article contains an error it will be rectified by means of an editorial note or erratum. In the event of dispute, the journals will ask the authors for the relevant explanations and evidence to clarify the case and will take a final decision. 
Relations with reviewers
The EAPC and the editorial committees strive to ensure that the review of journal articles is fair, impartial and timely. They provide guidance to reviewers on everything that is expected of them, including the commitment to handle aubmitted material with the appropriate confidentiality and to declare any other ethical issues. They provide instructions in a guide for reviewers that is updated regularly and linked to this code of ethics. 
The School and the editorial committees ask the reviewers to disclose possible conflicts of interest before accepting the review of articles. Likewise, a system of work has been established that guarantees a double blind review so that the identity of the reviewers remains anonymous as does the identity of the author whose work they are reviewing. 
The reviewers must consider the study they are reviewing as a confidential document until it is published, both during and after the review process. Under no circumstances shall they disseminate or use information, details, arguments or interpretations contained in the text subject to review for their own benefit or that of other individuals, nor for the purpose of favouring or harming third parties. 
Relations with the editorial committee members
The relationship between the EAPC, publisher of the journals, and the editorial committees of its journals, is based on the principle of editorial independence. The editorial committees determine which articles should be published based on their quality and suitability for the journals, without interference from the publisher. The EAPC formally appoints the members of the editorial committees, provides them with instructions on their functions and tasks, and asks them to adhere to this code of ethics. In addition, the EAPC provides new members of the editorial committee with the necessary guidelines on everything related to their functions and keeps them informed about new policies or lines of work. 
The editorial committees must be impartial when it comes to handling the submitted works and must respect the intellectual independence of the authors, whose right to reply if they have been reviewed negatively is respected. 
The editorial committees undertake to ensure that the selected reviewers are apt and able to judge the works without competitive interests with the authors. 
Members of the editorial committees are obliged to maintain the confidentiality of texts received and their content until they have been published. Also, no member of the editorial committees may use data, arguments or interpretations contained in unpublished works for their own research, unless it is expressly authorized in writing by the authors. Members of the editorial committees should also refrain from handling an original when it raises a conflict of interest, for example when there is a manifest relationship of kinship, friendship or enmity with the authors. 
Other commitments
The School and the editorial committees are open to receiving comments and reasoned complaints about the works published in their journals, and commit to responding promptly and to forwarding them to the authors so that they can also respond. 
The School’s scientific journals publish prominent studies that are the result of unpublished research and that are subject to a double-blind review process. In any case, when they include several sections, they will clearly indicate which are subject to blind review and which are not. 
The journals avoid publishing in-house works and generally publish only works by authors external to editorial boards and the EAPC. Under exceptional circumstances, the editorial committees may admit studies authored by members of the editorial boards, provided they do not represent more than 20% of the studies published in the same issue. In these cases, the School ensures that necessary technical conditions are in place so that the process of double-blind review of the studies can be carried out rigorously in accordance with the criteria established in this code of ethics. In addition, the journals may admit other types of collaborations by members of editorial boards or staff members linked to the EAPC, such as editorials, commentaries, news and reviews. 
Consulted sources:
- Code of conduct and best practice guidelines for journal editors of the Committee on Publication Ethics (COPE)
 - Guide to best practices for periodical and single-issue publications of the Spanish National Research Council (CSIC) 
 
Monographic section proposal guidelines
The Revista de Llengua i Dret, Journal of Language and Law (RLD), is open to proposals for publishing monographic sections that fall within its lines of study: administrative and legal language, language law, language policy and sociolinguistics. Monographic sections are to be published in the June or December issues of the RLD and they include an introductory article by the coordinators together with all the selected articles. 
Submission and evaluation of proposals
Those people who wish to coordinate a monographic section must send a proposal to the journal editor at the following address rld.eapc@gencat.cat.
This should include at least the following:
-       A suggested title for the monographic section.
-       Details of the monographic section coordinators (names, surnames, email address, ORCID number, position, university and a summary of their main academic merits).
-       Description of and purpose of the monographic section. This must answer at least the following questions: what are the aims of the section? How does it fit in with the journal? What is its interest? What new input does it provide? Does the proposal stem from a scientific event? (Specify it.)
-       Extensive summary of the introductory article by the editors.
-       Between 7 and 10 proposals for articles, which must contain the title, abstract and the authors’ names.
-       The planned work schedule.
We recommend following the structure of this form [link].
The Editorial Board, notwithstanding the possibility of asking for an external expert opinion, will assess the suitability of the proposal and accept or reject it, or suggest amendments. The editor will send a reply within a maximum of three months.
If they deem it necessary, those persons behind the proposal may request, before drafting it, support for the call for articles through the Revista de Llengua i Dret blog. The promoters are responsible for drawing the call, which must include the planned schedule for drafting, assessing and editing the articles, and for publicising it through their academic networks. We recommend that the call be in Catalan, Spanish and English, as these are the most common languages articles are published in.
Publication of the call in the blog means the journal’s editor regards the initiative in a positive light but does not guarantee approval of a future monographic section proposal. This proposal must be drawn up and submitted in accordance with the above guidelines. The Editorial Board will then assess it.
Editing the monographic section
The editorial process starts once the RLD Editorial Board has accepted the monographic section proposal.
The section coordinators are responsible for keeping in touch with the authors and ensuring they adhere to the schedule. Likewise, they must ensure the articles are unpublished, of suitable quality, comply with the code of ethics and follow the journal’s formal criteria.
The main formal criteria of the journal are as follows:
0.	Originals should be submitted in an editable electronic file format (e.g., DOC, DOCX, RTF, ODT).
0.	Originals must be anonymised when submitted.
0.	The maximum length for articles is 10,000 words, including footnotes and references.
0.	Each article must include the title, the abstract, the keywords, the body of the text and the corresponding bibliographical references.
0.	Authors should provide a biographical summary in the form on the website, as well as their ORCID number if they have one.
0.	Bibliographical references must follow the APA style stipulated for the RLD (see the criteria, available in Catalan, Spanish and English).
For further information on the journal criteria, consult our website. Should the articles not comply with RLD criteria, the documents will be returned to the authors so they can incorporate the necessary amendments.
Each author must send their article through the ournal platform. Once the submission has been received, the double-blind review process starts. All articles have to go through this process except for the introductory article, which will only have to undergo it in those cases where the Editorial Board considers it advisable. This process will be handled by the monographic section coordinators in collaboration with the Editorial Board and editorial team of the journal. Different collaboration models may be followed, depending on the type of monographic section in question.
A minimum of five articles will have to pass the blind review process for publication of the monographic section to go ahead. Should the final result not meet the expected quality, the Revista de Llengua i Dret Editorial Board may decide not to publish the monographic section. In that case, articles that pass the assessment may still be published independently.
The Editorial Board may make the process described in this guide more flexible should they consider it necessary to do so.
If you have any queries, please write to rld.eapc@gencat.cat.
