# Supplementary material for: Peer Review in Law Journals
Source: Front Res Metr Anal. 2021 Dec 8;6:787768. doi: 10.3389/frma.2021.787768 (PMC8692876; doi:10.3389/frma.2021.787768)
Supplement: Supplementary file 3 [file DataSheet2.ZIP › DOCUMENT - 0213-7585_1.RTF]

JOURNAL INFO
SHORT HISTORY OF THE REVISTA DE ESTUDIOS REGIONALES

History and contents.

It was in 1977 and by a quickly accepted initiative of Málaga's Economics Faculty that a group of university lecturers belonging to the campuses of Málaga, Seville, Granada and Córdoba met in different occasions along the year with the intention of bringing out a journal. It was agreed that this journal should have the following characteristics: it should focus on regional studies; empirical or theoretical analysis would be admitted; with a multidisciplinary approach, but circumscribed to the field of social sciences. After a year of preparations, the first issue of the Revista de Estudios Regionales (RER) was finally published in the first semester of 1978, with a Board of Directors and an Adviser Council constituted by lecturers from the four Universities mentioned above. They constitute the block of the "Founders", whose specificity was dutifully included in the RER statutes.

The journal came out, as it was pointed out in its "Presentation" to the first issue, "open to all". And it has certainly remained this way since then. Its ultimate purpose was "the need to fill a publishing gap on regional studies", thus offering "a channel for the scientific analysis of these topics", since these are issues "that, at the present moment, are of special concern for the Spanish public". So it was clearly established from the start the journal's main purpose and main object: covering the regional field in the domain of the social sciences with scientific rigor, aiming to deepen the knowledge and to bring about methodological renewal. These were the principles and aims that guided the contents of the RER, the same that guide them now.

Along its 25 years of uninterrupted existence, the principles, aims, contents and structure of the journal have been maintained and enriched. At present, the RER is the oldest journal of broad-ranging territorial issues in Spain. The Universities of Andalusia have been joining to the Editorial Board and to the Councils of the journal as they were being created (in the journal cover it is stated that the Universities of Andalusia are in charge of its publishing, and their respective emblems appear in the back page). In fact, in the very "Preamble" of its 1983 Statute the point is made that the RER "was born with an unmistakable will of rallying the Andalusian Universities in a common effort to know and improve their territory, thus meeting their commitment of participation placed upon them by society. The initiative is regarded as a starting point for the cooperation of Andalusian Universities to be extended to other fields in the future. Later on, by Agreement of all the Rectors it was settled the Statute that is in force at present. Finally, it might be of interest to remark that the journal publishes three issues annually since its start (in its first stage, two ordinary issues and a special issue containing the papers of Workshop of Regional Studies) and has had three layouts: the first one until issue 15/16 (1978-1986); the second from issues 17 to 43 (1987-1995); and the third, and current, from issue 44.

1. Scientific areas, collaborators and implantation.

The RER has been a journal of social sciences in its wider sense. It is, consequently, a multidisciplinary publication. The scientific areas with a predominant presence along the issues have been: Economics, Geography, Political Science, History, Sociology, Law, and Anthropology. The post of Editor, appointed by the Editorial Council (former Adviser Council) following the proposal of the Board of Directors, has been hold by Juan Ramón Cuadrado (University of Málaga, at the time), issues 1 to 10 (1978-1982); Javier Lasarte (University of Seville), issues 11 and 12 (1983); Adolfo Rodero (University of Córdoba), issues 13 to 16 (1984-1986); Manuel Martín Rodríguez (University of Granada), issues 17 to 25 (1987-1989); Manuel Delgado Cabeza (University of Seville), issues 26 to 52 (1990-1998); Juan Antonio Lacomba (University of Málaga), issue 53 (1999) to the present. The catalogue of authors is quite extended, including outstanding Spanish, European, and Latin American researchers. One only needs to observe the indexes of the issues in order to check this. This is a testimony of the popularity of the RER and of its scientific acknowledgement and interest throughout Spain, Europe and Latin America.
Being open to all researches in the field of regional science, a fair amount of authors submit their papers to the RER to be peer-reviewed and also considered by its Councils in their regular (four-monthly) meetings. It is only occasionally, or as a result of one of the Workshops held by the journal, that commissioned reports by specialists get to be published.

2. Organization and operation.

The organizational structure of the RER includes three Councils: the Board of Directors composed of the Rectors of the Andalusian Universities; the Editorial Council composed of three lecturers from each of these Universities, appointed by the corresponding Rector by a period of three years; and the External Adviser Council, integrated by specialists in the different areas covered by the journal, elected by the Editorial Council in view of their merits. The Universities of Andalusia constitute the key element that sustains and allows the growing of the journal.

The journals Operation takes the following steps: reception of original papers; distribution of these papers among the members of the Editorial Council or, in some occasions, among those of the External Adviser Council; designation of reviewers; final assessment of the scientific value of papers by the Council based on reviewers reports. Following this assessment three possible outcomes are considered: rejected papers, which are sent back to its authors explaining the decision; papers which hold some interest but which may profit from some changes and are consequently sent back to the authors with the indications of reviewers; and, finally, accepted papers, which will be published in the Articles or Notes section, according to the decision of reviewers. When a paper gets published in the RER includes an abstract both in Spanish and English, Keywords, also in Spanish and English, as well as the reception and acceptation date.

3. Final assessment.

The RER has published a total of 64 issues, plus 6 special monographs as result of the Workshop of Andalusian Studies that the journal holds with the sponsorship of the Bilbao Bank, with different numberings (from I to VI), which were devoted to the following subjects: I (1979), "Knowing and transforming Andalusia"; II (1980), "The Autonomous communities"; III (1981), "Cultural roots of Andalusia"; IV (1982), "Andalusia and the Common Market European"; V (1984), "The economic planning in Andalusia"; VI (1986), "Tourism in Andalusia". From 1987 on, the monograph issues (resulting as well from the Workshops, held with different sponsorships) took on the ordinary numbering, getting published as special volumes. Since then, these volumes have been: issue 19 (1987), "Regional Economic Incentives in Spain and in the EEC"; issue 22 (1988), "Regional Studies"; issue 28 (1990), "Symposium on the current situation and perspectives of the Andalusian economy"; issue 31 (1991), "International Seminar on Rural Labor Market in the South of Europe"; issue 36 (1993), "Workshop on higher education: financing and employment. A regional approach"; issue 43 (1995), "International Seminar on the territorial impacts of the processes of restructuring"; issue 44 (1996), "The Autonomous communities in the Spanish State: an assessment"; issue 54 (1999), "Workshop on inequality: a regional perspective"; issue 64 (2002), "Andalusia at the beginning of the third millennium". The interest of these subjects, plus the relevance both of the participants and of their papers are a testimony of the efforts of the RER and of its level of acknowledgement by outstanding specialists in the field of social sciences. Many of these monographs, some of them out of print, are considered landmark publications in their particular subject of study.

The RER issues contain an average of 250 pages. Its catalogue of authors goes over 300, being almost all of them lecturers from Andalusian and Spanish Universities, as well as from European and Latin American Universities. This confirm its reputation, esteem and acknowledgement in very important centers of study of regional and territorial questions. The Universities of Andalusia, in their sponsoring of the RER, have made possible a substantial development in this field of study, especially in its economic aspect, but also in its historical, geographical, political, sociological, anthropological, and other aspects. When doing the survey of the development of this line of research, one assesses the decisive role played by the RER since its start in 1978.
