# Supplementary material for: Peer Review in Law Journals
Source: Front Res Metr Anal. 2021 Dec 8;6:787768. doi: 10.3389/frma.2021.787768 (PMC8692876; doi:10.3389/frma.2021.787768)
Supplement: Supplementary file 3 [file DataSheet2.ZIP › DOCUMENT - 0214-8676_1.RTF]

Editorial Policies
?	Focus and Scope
?	Section Policies
?	Peer Review Process
?	Publication Frequency
?	Open Access Policy
?	Archiving
?	Ethics for publications
?	Anti-plagiarism policy
?	Editorial practices regarding gender equality
Focus and Scope
Doxa. Cuadernos de Filosofía del Derecho is an international journal which has been published by the University of Alicante since 1984. It was launched at a time of renewed interest in the philosophy of law in Spain, and is aimed at serving as a vehicle for communication and exchange among philosophers of law in Latin America and Latin Europe.
The journal welcomes two types of submission: "articles" and "notes", both of which must be unpublished research reports. The journal reserves the right to decide how to classify the submissions received.
 
Section Policies
Articles
 Open Submissions	 Indexed	 Peer Reviewed	
Notes
 Open Submissions	 Indexed	 Peer Reviewed	
Interview
 Open Submissions	 Indexed	 Peer Reviewed	
 
Peer Review Process
The journal editorial team will acknowledge receipt of submissions, which will be sent for confidential peer review to at least two anonymous reviewers who meet the minimum relevant requirements. In the light of the peer review, the editorial team shall take one of the following decisions, which shall be communicated to the author(s):
0.	Acceptance for publication.
0.	Acceptance for publication conditional on implementation of the required changes.
0.	Rejection.
The journal Doxa. Cuadernos de Filosofía del Derecho does not have either article submission charges or article processing charges (APCs).
 
Publication Frequency
Doxa. Cuadernos de Filosofía del Derecho has been published annually since 1984 (except for 1992 and 1993, when it was published biannually).
 
Open Access Policy
This is an open-access journal, i.e. all content is available free of charge for users or their institutions. Users may read, download, copy, distribute, print, search for or add links to the full texts of the articles, or use them for any other legal purpose, without prior consent from the publisher or author, in accordance with the Budapest Open Access Initiative.
All contents of the journal are immediately available upon publication. No previous registration is required.

 
Archiving
ARCHIVING AND DIGITAL PRESERVATION
This journal uses the LOCKSS system (https://www.lockss.org/) to create a distributed archiving system among participating libraries and permits those libraries to create permanent archives of the journal for purposes of preservation and restoration. The LOOKSS publisher manifest may be found at: https://doxa.ua.es/gateway/lockss
In addition to the usual multiple version backup copy procedures, the journal contents are replicated in the institutional repository of the University of Alicante (based on Dspace, https://duraspace.org/dspace/). The journal website in such repository is: http://rua.ua.es/dspace/handle/10045/9949?locale=en
INTEROPERABILITY PROTOCOLS
The journal implements the OAI-PMH (Open Archives Initiative Protocol for Metadata Harvesting, https://www.openarchives.org/pmh/), https://www.openarchives.org/pmh/), protocol, a tool for metadata harvesting from repositories. This feature may be verified through the following request: https://doxa.ua.es/oai
 
Ethics for publications
ETHICAL STANDARDS
DOXA. Cuadernos de Filosofía del Derecho has been contributing to foster and to consolidate legal philosophical debate in Spanish. Since 1984, this Journal has been yearly released in the University of Alicante. This means 41 issues, more than 1000 works (including articles, notes, interviews). Through publication of high quality academic work, DOXA has created a legal philosophical community for the Latin World, both American and European. DOXA. Cuadernos de Filosofía del Derecho is today a standing journal that gives voice to all different legal philosophy schools of thought able to contribute to enrich the quality of ius-philosophical inquiry. Thus, authors and readers from the Academy and beyond see in this Journal a reference of high quality standards as well as a serious publication where to read and publish contributions to the eternal legal philosophical topics or to current legal philosophical topics of debate.
DOXA. Cuadernos de Filosofía del Derecho adheres to the guidelines established by EASE (European Association of Science Editors) with respect to the management, edition, revision, and publication of scientific results in journals concerning different areas of expertise.
ETHICAL COMMITMENTS
1. Ethical guidelines
`.	The authors pledge to submit only original manuscripts that had not been published earlier and that are not currently subject to any other journal’s review process. Where portions of the content overlap with published or submitted content, authors will acknowledge and cite those sources, as well as, if necessary, they will obtain permissions. Additionally, if require, authors will provide the editor with a copy of any submitted manuscript that might contain overlapping or closely related content. Author will also need: To declare any potential conflicts of interest (e.g. where the author has a competing interest (real or apparent) that could be considered or viewed as exerting an undue influence on his or her duties at any stage during the publication process); To notify promptly the journal editor or publisher if a significant error in their publication is identified; To cooperate with the editor and publisher to publish an erratum, addendum, corrigendum notice, or to retract the paper, where this is deemed necessary.
Likewise, submitting a manuscript implies the author’s acceptance of this Journal’s rules for publication, revision, and evaluation. On the other hand DOXA. Cuadernos de Filosofía del Derecho will inform authors of submitted papers in a prompt and timely manner whether their work is accepted; will ensure that any copy-editing is respectful of authors’ style and ideas; and will give authors adequate time to see and correct the final proofs.
Following best examples of high standard codes of good practices, this Journal rules (which will also be reproduced in each issue) are the following:
`.	Anonym peer reviewers assume the burden of carrying out, within the confines of their skills and knowledge, a critical, honest, and constructive review regarding the scientific quality of the manuscripts entrusted to them. This is the reason why each reviewer will accept to assess a manuscript only if they consider themselves sufficiently competent and if there are no conflicts of interest. Peer reviewers ought to maintain the confidentiality of any information supplied by the editor or author and not to retain or copy the manuscript. They also assume the responsibility to alert the editor of any published or submitted content that is substantially similar to that under review. Finally, peer reviewers committed to blind review need to be aware of any potential conflicts of interest (financial, institutional, collaborative or other relationships between the reviewer and author) and to alert the editor to these, if necessary withdrawing their services for that manuscript.
`.	The Directors, the members of the Redaction Board, of the Editorial Board, and of the Advisory Board pledge to be impartial and to maintain the confidentiality of the manuscripts submitted to DOXA. Cuadernos de Filosofía del Derecho, of their authors, and of the designated reviewers. All of these is done in order to let the principle of anonymity to preserve the integrity of the whole assessment process. To that effect, the selection of the most qualified reviewers and specialists will be guaranteed. The editorial team pledges to avoid any kind of conflict of interest and to strictly observe the assessment, edition, and publication deadlines that are consistent with the Journal’s periodicity.
2. Copyright
Submitting a manuscript to DOXA.Cuadernos de Filosofía del Derecho implies the acceptance of the following terms:
`.	The authors pledge to observe the above-mentioned ethical guidelines.
`.	The authors grant DOXA the right to the first publication of their manuscripts, and the license to publish them in all formats and means available to the Journal, either in print or in electronic versions.
`.	DOXA will promptly forward any requests to reprint, abridge, or translate an author’s article to the author, so that he or she can decide whether to agree or not. In the case that an author decides to subsequently publish the content of her/his manuscript elsewhere, either completely or partially, she/he pledges to specify in the subsequent publication that a version of her/his work firstly appeared in DOXA.
3. Procedures for dealing with unethical behaviour
0.	Identification of unethical behaviour
o	Misconduct and unethical behavior may be identified and brought to the attention of the editor and publisher at any time, by anyone.
o	Misconduct and unethical behaviour may include, but need not be limited to, examples as outlined above.
o	Whoever informs the editor or publisher of such conduct should provide sufficient information and evidence in order for an investigation to be initiated. All allegations should be taken seriously and treated in the same way, until a successful decision or conclusion is reached.
0.	Investigation
o	The editor, who should seek advice from the publisher, if appropriate, should take an initial decision.
o	Evidence should be gathered, while avoiding spreading any allegations beyond those who need to know.
0.	Minor breaches
o	Minor misconduct might be dealt with without the need to consult more widely. In any event, the author should be given the opportunity to respond to any allegations.
0.	Serious breaches
o	Serious misconduct might require that the [public or private] employers of the accused be notified. The editor, in consultation with the publisher or editorial team as appropriate, should make the decision whether or not to involve the employers, either by examining the available evidence themselves or by further consultation with a limited number of experts.
0.	Outcomes (in increasing order of severity; may be applied separately or in conjunction)
o	Informing or educating the author or reviewer where there appears to be a misunderstanding or misapplication of acceptable standards.
o	A more strongly worded letter to the author or reviewer covering the misconduct and as a warning to future behaviour.
o	Publication of a formal notice detailing the misconduct.
o	Publication of an editorial detailing the misconduct.
o	A formal letter to the head of the author's or reviewer's department or funding agency.
o	Formal retraction or withdrawal of a publication from the journal, in conjunction with informing the head of the author or reviewer's department, Abstracting & Indexing services and the readership of the publication.
o	Imposition of a formal embargo on contributions from an individual for a defined period.
o	Reporting the case and outcome to a professional organisation or higher authority for further investigation and action.
 
Anti-plagiarism policy
Doxa. Cuadernos de Filosofía del Derecho has an anti-plagiarism policy in place to guarantee that all manuscripts are original. Anti-plagiarism software Turnitin is used to detect coincidences and similarities between texts submitted for publication and those previously published in other sources. The Editorial Board will run anti-plagiarism checks on submitted manuscripts before proceeding with formal review and external assessment. If suspected cases of plagiarism are detected, the manuscript will not be published and authors will be informed.
 
Editorial practices regarding gender equality
Doxa. Cuadernos de Filosofía del Derecho is committed to implementing gender policies that lead to real equality between women and men in society. This commitment is reflected in a number of key actions.
1 EDITORIAL PARTICIPATION
The journal will take measures to ensure balanced gender representation, both on the journal's different boards and among manuscript reviewers.
2 USE OF INCLUSIVE LANGUAGE
Doxa. Cuadernos de Filosofía del Derecho recommends using inclusive language in scientific articles, in order to take into account the presence and situation of women in society and in accordance with the principle of gender equality. In this sense, authors are encouraged to use gender-neutral rather than masculine terms to refer to classes or groups of individuals, whether male or female. It is also advisable to add explanatory phrases, avoid references to the subject or, if none of the above is possible, make reference to both women and men in the text.
For further details and recommendations, see the University of Alicante's inclusive language guide (PDF) (available in Spanish and Catalan).
3 SEX AND GENDER IN RESEARCH
When writing research works, authors must avoid gender stereotypes and biases whereby men are regarded as the universal point of reference, biological differences are exacerbated or socially constructed differences are seen as natural. They must also consider the sex variable in research of any kind concerning persons, animals, tissues or cells, which involves:
0.	Reflecting and making well-founded decisions on sample composition by sex and providing information about the sex of the subjects analysed.
0.	Analysing existing differences within each sex and presenting results disaggregated by sex.
For more information, see the practical guide for the inclusion of the gender perspective in research contents (PDF) (available in Spanish).
