# Supplementary material for: Peer Review in Law Journals
Source: Front Res Metr Anal. 2021 Dec 8;6:787768. doi: 10.3389/frma.2021.787768 (PMC8692876; doi:10.3389/frma.2021.787768)
Supplement: Supplementary file 3 [file DataSheet2.ZIP › DOCUMENT - 0350-154X.RTF]

﻿  

SMJERNICE ZA AUTORE  
       
Znanstveni    otvorenog  pristupa  
Revija za sociologiju prvenstveno objavljuje izvorne znanstvene (istraživačke) radove i  pregledne  radove  od interesa sociologiju  i  srodne  discipline,  s  posebnim naglaskom na radove koji doprinose boljem razumijevanju tema relevantnih za kontekst  
Hrvatske,  regije  zapadnog  Balkana,  te općenito Centralne i Istočne Europe.    
 Časopis prihvaća   na  razmatranje  i prethodna priopćenja i stručne radove , pri čemu se od autora traži da pri prijavi naznače tu  kategorizaciju  za  radove  koji zadovoljavaju sljedeće kriterije:   
-  Prethodno (ili bilješka)   –   Znanstveni  rad  koji neobjavljene  rezultate  izvornih istraživanja u preliminarom obliku.   
-  Stručni rad  –  Rad usmjeren razmatranju kako se već poznati rezultati znanstvenih istraživanja mogu primijeniti u praksi.   
  
Poželjno je da opseg znanstvenih i stručnih radova sažetak, literaturu i grafičke priloge) ne prelazi 8000 riječi. se za nakon dviju pozitivnih anonimnih recenzija.  
 Časopis također objavljuje  prikaze knjiga i konferencija, i eseje kritičke rasprave  /  edukativne  ili  popularizacijske tekstove u rubrici Forum. Prilozi za rubriku  
Forum uključuju, ali nisu ograničeni na:   
-  Priloge se na od interesa  za ili sociološku akademsku zajednicu;  
-  Priloge pružaju u ili slabije istraživačke i/ili analitičke tehnike;   
-  Priloge  u  kojima  se  raspravljaju raznovrsni teorijski pristupi ili modeli;  
-  Priloge pružaju raznih softvera  za  analizu  ili  upravljanje (kvalitativnim  ili  kvantitativnim) podacima;   
-  Tekstove društvenim popularizirajuće ili edukativne naravi.   

GUIDELINES FOR AUTHORS  
  
The  peer-reviewed,  open  access  journal  
Croatian  Sociological  Review  primarily publishes  original  research  articles  and review  articles  of  general  interest  for sociology  and  related  disciplines,  with  an emphasis on articles that contribute to a better understanding of topics relevant for the context of  Croatia,  the  Western  Balkans  and,  more generally, Central and Eastern Europe.   
  
The  journal  might  also  consider  preliminary communications  and  technical  papers.  
Authors are required to indicate whether their paper  fits  the  criteria  for  these  categories during the submission process:  
-  Preliminary  communication  (or  research note)  –   Scientific  article  presenting preliminary scientific results.  
-  Technical  (professional)  paper  –   Paper considering how available scientific results can be used in practice.  
  
The preferred length of scientific and technical articles  (including  the  abstract,  notes, references,  and  graphs  and  tables)  is  below 8000  words.  Articles  are  accepted  for publication  after  two  positive  blind  peer reviews.  
  
The  journal  also  publishes  book  and conference  reviews,  as  well  as  shorter essays  /  critical  discussions  /  educational  or popularization  texts  in  the  Forum  section.  
Forum contributions include, but are not limited to:  
-  Critical  discussions  or  debates  about matters  of  interest  to  society  or  the academic and sociological community;  
-  Introductory  texts  about  advanced  or unfamiliar  research  methods  and/or analytical techniques;  
-  Discussions  of  diverse  theoretical approaches or models;  
-  Notes  about  various  data  analysis  or (qualitative  or  quantitative)  date management software;  
-  Social  sciences'  popularization  or educational texts.

  

Opseg prikaza ne bi smio prelaziti 1200 riječi.  
Prilozi za rubriku Forum trebaju biti između 1000 i 5000 riječi. Ovi prilozi prihvaćaju se za objavljivanje  nakon  pozitivne  recenzije uredništva.    
  
Prijava rada   
Radovi  se  zaprimaju  isključivo   preko elektroničkog za časopisa na mrežnim stranicama  Revije za sociologiju:  
https://hrcak.srce.hr/ojs/index.php/rzs/about/ submissions  
       
Autori zadržavaju autorska prava za članke objavljene u časopisu, no svojim pristankom na daju pr avo  prvog objavljivanja  u  tiskanom  te  elektroničkom obliku.  Revija  za  sociologiju  autorima  ne naplaćuje obrađivanje objavljivanje priloga  
  
Uredništvo  zaprima  samo  one  radove  koji prethodno  nisu  objavljivani  niti  su  u recenzijskom  postupku  pri  nekom  drugom časopisu.    
  
Radovi  se  zaprimaju  i  objavljuju  na hrvatskom  ili  engleskom  jeziku,  a  po posebnoj uredništva na jezicima.   
  
Radovi trebaju biti pripremljeni u OpenOffice,  
Microsoft Word ili RTF formatu.   
  
Autori  mogu  inicijalno  predati  svoj  rad dosljedno bilo autor -datum sustav referenci. slučaju prihvaćanja rada za objavu, od autora će se tražiti rad  ede  u  skladu  sa  sustavom navođenja kojeg  Revija  za sociologiju.  
***  
      
Pri  pripremi  rada,  autore  se  poziva  da obvezno  konzultiraju  i  Izjavu izdavačkoj etici,  a  pogotovo  dio  o definiciji  autorstva  i zahvalama.  Ta  je  izjava  dostupna  online:  
https://hrcak.srce.hr/upute/etika_hr_Revija_ za_sociologiju.pdf  

The length of reviews should not exceed 1200 words. Forum contributions should be between 1000 and 5000 words. These contributions are accepted  for  publication  after  a  positive editorial review.  
  
Submission  
Articles  should  be  submitted  exclusively through  the  Open  Journal  System  of  the  
Croatian Sociological Review:  
https://hrcak.srce.hr/ojs/index.php/rzs/about/s ubmissions  

The  authors  reserve  copyright  for  articles published in the journal but, by giving consent for publication, they delegate the right of first publication in print and in electronic format to the journal. The Croatian Sociological Review charges  no  article  submission,  processing  or publication fees to authors  

The  editorial  board  accepts  for  consideration only  those  articles  that  have  not  been published  previously  or  that  are  not  under consideration in another journal.   
  
Articles  are  considered  and  published  in  
Croatian  or  English.  Under  certain circumstances,  the  editors  can  decide  to consider  and  publish  articles  in  other languages.  
  
Manuscripts  should  be  submitted  in  
OpenOffice, Microsoft Word or RTF format.  
  
Authors  may  initially  submit  their  manuscript using  any  consistent  author-date  referencing style.  After  the  acceptance,  authors  will  be required to edit their article in accordance with the  style  used  by  the  Croatian  Sociological  
Review.  
***  
When preparing their manuscripts, the authors are requested to also consider the Statement of  Publication  Ethics,  and  to  particularly examine  the  guidelines  on  the  authorship definition  and  the  acknowledgements.  This  
Statement  is  available  online:  
https://hrcak.srce.hr/upute/etika_en_Revija_z a_sociologiju.pdf

  

Revija  za  sociologiju  ne  zahtjeva  uvodno pismo kao obvezan dio prijave, no svakako ohrabruje prijave koje uključuju  takav uvodni tekst u kojem se ukratko predstavlja doprinos rada i definira njegova prikladnost za objavu u  Reviji.  Takvo pismo je uključiti dio  obrasca  "Komentari uredniku"  koje  je dio prijave rada.  
  
Osim  opcionalnog  uvodnog  pisma,  autori  u obrascu  "Komentari  uredniku"  također mogu naznačiti i neke druge informacije koje smatraju  potrebnim  naglasiti  uredniku,  a dodatno traži da  obvezno  naznače sljedeće  informacije:  
1)  Iskaz o sukobu interesa, ako postoji.  
2)  Iskaz  o  postojanju  etičn e  suglasnosti.  
Ako  te  informacije  nisu  navedene  u samom tekstu, potrebno je pri prijavi rada pružiti informacije  je li istraživanje koje su autori  proveli  dobilo  suglasnost povjerenstva za prosudbu etičnosti  (ako se o za  koje  je  takva suglasnost  potrebna).  Ako  potrebna etična nedostaje, trebaju pružiti objašnjenje o tome.    
3)  Informacije pruženoj potpori, ako je ima.   
4)  Zahvale, ako postoje.  
5)  Bilo  koje  relevantne  informacije  koje mogu otkriti identitet autora, a koje su bile uklonjene  iz  rukopisa  u  procesu anonimizacije.   
  
Anonimizirani rukopis  
Autori su obvezni svoj rukopis anonimizirati prije  elektroničke rada.  
Anonimizacija  nije  potrebna  za  priloge namijenjene rubrikama Prikazi i Forum.   
  
Iz anonimiziranog rukopisa treba ukloniti sve informacije koje bi mogle uputiti na identitet autora. Ovo također uključuje i informacije o instituciji  ili  projektu  unutar  kojeg  je  rad izrađen.    
  
Također potrebno  ukloniti  sve informacije  o  autoru  iz  naslova,  kao  i  iz sistemskih koje sama  

The  Croatian  Sociological  Review  does  not require,  although  it  encourages  authors  to submit a cover letter. In such an introductory text  the  authors  are  welcome  to  concisely specify a contribution of their manuscript and its fit for the journal. Such a cover letter can be included  into  the  section  "Comments  to  the  
Editor"  which  is  an  integral  part  of  the electronic submission process.  
  
In addition to an optional cover letter, authors are  asked  to  indicate  in  the  section “ Comments  to  the  Editor” information that requires the editor's attention, and they are additionally specifically requested to provide the following information:   
1)  Disclosure  statement.  Declaration  of conflicting interests, if any.  
2)  Ethics  approval  statement.  If  such  an information  is  not  part  of  the  manuscript, the submission of the manuscript needs to be  accompanied  by  the  information whether an ethics committee approved the research  presented  in  the  text  (if applicable).  In  case  of  unavailable  ethics committee approval for research requiring one,  authors  are  asked  to  provide  an explanation.  
3)  Acknowledgments of funding, if any.  
4)  Other acknowledgements, if any.  
5)  Any  relevant  identifying  information  that were removed from the manuscript during anonymization.  
  
  
Anonymized manuscript    
Authors  are  required  to  anonymize  their manuscript  prior  to  electronic  submission.  
Anonymization  is  not  necessary  for  the submissions  to  the  sections  of  book  and conference reviews and Forum.  
   
All identifying information should be removed from  the  manuscript.  This  also  includes references  to  the  institution  or  the  project  to which the manuscript is associated.  
  
Personal  information  about  the  author noted in file title and file properties should also  be  removed  (e.g.  in  Microsoft  Word:
 datoteka (npr.  

  

Microsoft  

  

Word:  

  

Inspect 

  

Document – 

  

Remove  

  

Document  

  

Properties and Personal Information). 

U slučaju upućivanja čitatelja na autorove ranije radove u tijelu teksta, nije potrebna izmjena ako se u tom upućivanju ne otkriva da se radi o autoru/ima rukopisa. 

  

Drugim riječima, ako iz načina na koji se koristi referenca u tekstu ništa ne upućuje da je autor reference ujedno i autor teksta, tada se ta referenca unosi kao i obično. 

  

No, u slučaju da upućivanje otkriva da se radi o istom/im autoru/ima, a nije moguće privremeno tu referencu izostaviti, tada je potrebno referencu u tekstu oblikovati na sljedeći način: (AUTOR), te je privremeno ukloniti iz 

  

Popisa literature. 

Prva stranica anonimiziranog rukopisa treba sadržavati (na jeziku na kojem je rukopis napisan): 
 1) 

  

Naslov rukopisa. 
 2) 

  

Sažetak, opsega do 250 riječi, koji treba sadržavati opći prikaz teme, metodološki pristup, glavne rezultate i zaključak. 
 3) 

  

Do pet ključnih riječi. 

Druga strana anonimiziranog rukopisa treba započeti s tijelom teksta, sli jedeći dolje navedene upute. 
 1) 

  

Rad treba biti pisan jednostrukim proredom (font Times New Roman, Arial ili bilo koji drugi standardni font, veličina 12).  

  

Poželjno je, iako ne nužno, da naslovi i podnaslovi budu numerirani. 
 2) 

  

Grafički prilozi (tablice, grafikoni, slike i sl ično) moraju biti izrađeni u crno 

-bijeloj tehnici na jedan od standardnih načina računalne izrade (poželjna razlučivost slika jest 300 dpi), a po pravilu se prilažu unutar teksta rada na mjestima gdje trebaju biti prikazani. 

  

U slučaju preuzimanja priloga iz drugog izvora autori su sami dužni osigurati dopuštenje za korištenje grafičkog priloga. 

  

U procesu elektroničke prijave rada, autori će također biti u mogućnosti – i preporuča se da to učine 

  

-grafičke priloge dodatno prilo žiti i u izvornom obliku (primjerice, 

  

  

  

  

  

  

  

  

  

  

  

  

  

  

  

  

  

  

  

  

  

  

  

  

  

  

  

Inspect  

  

Document – 

  

Remove  

  

Document 

  

Properties and Personal Information). 

If the authors are referring to their previous work in their manuscript, they should keep the references in the text intact if these references do not suggest the authors' identity. 

  

In other words, if the references to previous work are used in such a way that the reader cannot guess that the author of the reference and the author of the text are the same person, then the reference should be inserted in an usual way. 

  

However, if the references contain identifying information, and it is not possible to temporarily remove them from the text, then these references should be presented in the text as follows:  
 (AUTHOR), and temporarily removed from the Bibliography. 

The first page of the anonymized manuscript must contain (in the language of the manuscript): 
 1) 

  

Title of the manuscript 2) 

  

Abstract of a maximum 250 words which should contain a general presentation of the subject, the methods implemented, the main results and the conclusion(s). 
 3) 

  

Up to five key words. 

The second page of the anonymized manuscript should start with the body of the text, following the instructions below. 
 1) 

  

The text should be single spaced using 

Times  

  

New  

  

Roman,  

  

Arial or another common 12-point font.  

  

It is desirable, but not necessary, that the headings and subheadings are numbered. 
 2) 

  

Illustrations (tables, graphs, figures, etc.) must be in black and white in one of the standard computer software programs (recommended figure resolution is 300 dpi), and they should be placed within the body of the text.  

  

If such material is taken from other sources, the author is required to secure copyright permission for use of illustrations.  

  

During the electronic submission of the article, authors will have an opportunity 

  

-and they are encouraged to do so 

  

-to also submit all illustrations in their original format (e.g. jpg, pdf, eps and jpg, pdf, eps i sl., te .xls tablica na kojoj se temelji grafikon...).  
3)  Navođenje sintagmi, podataka i sl. označuje se u tekstu, a ne u bilješkama.   
4)  Bilješke (fusnote), koje služe za dodatna objašnjenja,  entare  ili  digresije, ispisuju se na dnu pojedine stranice, a ne na teksta. ako moguće, bilješke (fusnote) trebalo bi izbjegavati.   
  
Nakon rada, autora se tražiti da rukopis urede u skladu sa sustavom navođenja referenci opisanim  niže .  
  
[Molim, mrežnu časopisa za dostupnost bibliografskih obrazaca.]  
       
Navođenje u tekstu.    
U tekstu se izvor stavlja u zagrade i sadržava prezime godinu i, slučaju citiranja, redni broj stranice:  
(Bourdieu, 1979) (Berger i Luckmann, 1966: 119).   
(Bourdieu, 1979; Berger i Luckmann, 1966:  
119).   
  
  
Ako navodi radova autora objavljenih u jednoj godini, valja ih razlikovati slovom (a, b, c itd.) iza godine izdanja:  
(Bourdieu, 1979a, 1979b)  
  
Ako rad ima do tri autora, treba navesti sva tri autora u prvom spominjanju u tekstu:   
(Beck, Giddens i Lash, 1994)  
  
U  naknadnim  spominjanjima rada  tri  autora  kao  i  u  prvom  i  u  svim naknadnim spominjanjima zajedničkog rada četvorice ili više autora kori sti se oblik “i dr.”:   
(Beck i dr., 1994)  
   
Oznake “ibid.”, “op. cit.” i slično ne koriste se.   
  
  
Popis literature.   
U literature se radovi navedeni  u  tekstu  i  to  abecednim  redom similar, .xls table which is the source of the graphs…).   
3)  Other phrases, data similar pieces should be referenced in the text, not in the footnotes.  
4)  For additional clarification and comments, footnotes  are  to  be  used  instead  of endnotes. However, if possible, the use of footnotes should be avoided.  
  
Manuscripts  accepted  for  publication  will  be required to  be  edited  in  the referencing  style described below:   
  
[Please,  check  the  journal's  website  for  the availability of the bibliographic templates.]  
  
In-text citations.  
The  source  in  the  text  is  placed  in  the parenthesis that contains the last name of the author(s), year of publication, and, in cases of direct citations, the page number:  
(Bourdieu, 1979) (Berger and Luckmann, 1966: 119).   
(Bourdieu, 1979; Berger and Luckmann, 1966:  
119).   
  
References to more than one work by the same author(s)  in  the  same  year  should  be distinguished by letters (a, b, c, etc.) following the year of publication:  
(Bourdieu, 1979a, 1979b)  
  
In works with up to three authors, all last names should be written in the first mention in the text:   
(Beck, Giddens and Lash, 1994)  
  
Subsequent  references  to  works  with  three authors,  as  well  as  the  first  and  subsequent references to works with four or more authors should use the “et al.” form:   
(Beck et al., 1994)  
  
Notations as “op. and such similar forms should not be used.  
  
Bibliography.  
The reference list should contain all the works referred  to  in  the  text  listed  in  alphabetical order  according  to  the  last  names  of  the prema autora kronološkim slijedom za radove istog autora.   
  
Ako navodi radova autora objavljenih u jednoj godini, valja ih razlikovati slovom  (a,  b,  c  itd.)  iza  godine  izdanja.  Za zajednički rad više autora u popisu literature navode se svi autori s punim prezimenom i inicijalima imena.  
  
Iza bibliografskih jedinica koje ga posjeduju treba DOI obliku mrežne poveznice  (https://doi.org/10.xxxx/xxxxx) ispred koje se ne nalazi riječ “doi” ili “DOI”.   
  
Za izvor je punu mrežnu (http://) u naznačiti pristupanja. novinske članke u mrežnom obliku potrebno je navesti i datum objave.   
  
Primjeri navođenja literature:    
       
Knjiga  
Bourdieu P, Passeron J-C i Chamboredon J- 
C  (1973).  Le  métier  de  sociologue.  Paris:  
Mouton i Bordas  
       
Prilog u knjizi/zborniku  
Lalić D i Bulat N (1992). Rat i mladi: prema novoj generacijskoj kulturi. U: Čaldarović O,  
Mesić M i Štulhofer A (ur.).  Sociologija i rat.  
Zagreb: Hrvatsko sociološko društvo, 83 – 91 Članak u časopisu   
Konecki KT (2011). Visual Grounded  
Theory: A Methodological Outline and  
Examples from Empirical Work, Revija za sociologiju, 41 (2): 131– 160.  
https://doi.org/10.5613/rzs.41.2.1  
       
Elektronička objava teksta prije tiska    
Konecki KT (2011). Visual Grounded Theory:  
A  Methodological  Outline  and  Examples from  Empirical  Work,  Revija  za  sociologiju.  
Elektronička teksta tiska, siječnja. https://doi.org/10.5613/rzs.41.2.1 authors  and  in  chronological  order  for  works from the same author(s).   
  
References to more than one work by the same author(s)  in  the  same  year  should  be distinguished by letters (a, b, c, etc.) following the  year  of  publication.  Co-authored  works should  be  referenced  with  the  full  last  name and initials of all authors.  
  
The  DOI  should  be  added  after  those bibliographic units that have one in the form of full URL link (https://doi.org/10.xxxx/xxxxx) that is not preceded by the word “doi” or “DOI”.    
  
Works  that  are  available  online  should  also include  full  URL  link  (http://)  and  the  date  of access  in  brackets.  Newspaper  articles available online should also include the date of publication.   
  
Examples:  
  
Books  
Bourdieu P, Passeron J-C and Chamboredon  
J-C  (1973).  Le  métier  de  sociologue.  Paris:  
Mouton and Bordas  
  
Chapters in books / edited collections  
Lalić D and Bulat N (1992). Rat i mladi: prema novoj  generacijskoj In: O,  
Mesić M and Štulhofer A (eds).  Sociologija i rat.  
Zagreb: Hrvatsko sociološko društvo, 83 – 91  
  
Journal articles  
Konecki KT (2011). Visual Grounded Theory:  
A Methodological Outline and Examples from  
Empirical Work, Croatian Sociological Review, 41 (2): 131– 160.  
https://doi.org/0.5613/rzs.41.2.1  
  
Advanced online publication  
Konecki KT (2011). Visual Grounded Theory:  
A Methodological Outline and Examples from  
Empirical Work, Croatian Sociological Review.  
Advanced  online  publication,  15  January.  
https://doi.org/10.5613/rzs.41.2.1  
  
  

  

Mrežni izvor   
COPE Council (2017). Ethical guidelines for peer reviewers.  
https://publicationethics.org/files/Ethical_Gui delines_For_Peer_Reviewers_2.pdf (15.  
svibnja 2019.)  
  
Novinski članak   
Kalogjera I (2010). Prođite kroz test iz sociologije. Jutarnji list, 15. travnja.  
https://www.jutarnji.hr/vijesti/hrvatska/prodit e-kroz-test-iz-sociologije-pitanja-odgovori-ikomentari-strucnjaka/2260493 (10. lipnja 2019.)  
  
Korporativni izvor  
UNDP-Hrvatska (2006). Neumreženi: lica socijalne isključenosti u Hrvatskoj . Zagreb:  
Program Ujedinjenih naroda za razvoj -  
Hrvatska http://www.undp.hr/upload/file/130/65076/FI 
LENAME/WEB_hrvatska_verzija.pdf (1.  
rujna 2010.)  

Website  
COPE Council (2017). Ethical guidelines for peer reviewers.   
https://publicationethics.org/files/Ethical_Guid elines_For_Peer_Reviewers_2.pdf (15 May 2019)  
       
Newspaper article  
Kalogjera I (2010). Prođite kroz test iz sociologije. Jutarnji list, 15 April.  
https://www.jutarnji.hr/vijesti/hrvatska/prodite-kroz-test-iz-sociologije-pitanja-odgovori-ikomentari-strucnjaka/2260493 (10 June 2019)  
  
Corporate source  
UNDP-Hrvatska (2006). Neumreženi: lica socijalne isključenosti u Hrvatskoj . Zagreb:  
Program Ujedinjenih naroda za razvoj -  
Hrvatska http://www.undp.hr/upload/file/130/65076/FIL 
ENAME/WEB_hrvatska_verzija.pdf (1  
September 2010)  
  
  
  
