# Supplementary material for: Peer Review in Law Journals
Source: Front Res Metr Anal. 2021 Dec 8;6:787768. doi: 10.3389/frma.2021.787768 (PMC8692876; doi:10.3389/frma.2021.787768)
Supplement: Supplementary file 3 [file DataSheet2.ZIP › DOCUMENT - 0350-2058.RTF]

Information for Contributors


Collected Papers of Zagreb Law Faculty (Zbornik Pravnog fakulteta u Zagrebu) is a journal which publishes papers primarily from the fields of legal and social sciences. The Editorial Board accepts for consideration exclusively previously unpublished papers. Authors retain the copyright on the papers published in the journal, but grant the right of the first publication to the journal. The paper accepted for publication or already published in Collected Papers may be published by the author(s) in other publications only with the permission of the Editorial Board, and in such a case only with proper notice of its publication in Collected Papers.   

Manuscripts should be submitted on a diskette or CD-Rom in Microsoft Word or RTF format and two double-spaced printed copies. The title of the paper, the author’s name, surname and academic or professional status should be indicated, as well as the name and address of the author’s place of work or home address and e-mail address.

Manuscripts are anonymously reviewed twice and categorised as follows:
(1) Original scientific paper – the paper which is characterised by originality of conclusions, or which presents previously unpublished original results of scientific research;
(2) Review article - the article that contains detailed and comprehensive critical review of a certain problem area, but with no significant originality of results;
(3) Preliminary communication – the paper which presents primary findings of research in progress, which due to current interest require immediate publication, but without the level of deep and thorough study required for the scientific paper, and
(4) Professional paper – the paper which contains information and experience relevant for a certain profession, but without scientific characteristics.

Manuscripts as a rule should not exceed thirty-two double-spaced pages. They should include   an introductory synopsis of not more than ten lines, a list of up to five key words and a summary of not more than one double-spaced page. Book reviews, commentaries and evaluative reviews are not subject to review and should generally be no longer than eight double-spaced pages.

The Editorial Board reserves the right to edit the paper according to the general rules of editing publications and the standard of the Croatian language. Manuscripts will not be returned. Notes should supply full data on references since they are not published separately. References should include the name of the author, the title, the publisher, place and date of publication and the number of the page(s) to which they refer. 
