# Supplementary material for: Peer Review in Law Journals
Source: Front Res Metr Anal. 2021 Dec 8;6:787768. doi: 10.3389/frma.2021.787768 (PMC8692876; doi:10.3389/frma.2021.787768)
Supplement: Supplementary file 3 [file DataSheet2.ZIP › DOCUMENT - 0350-6886.RTF]

GUIDELINES FOR REVIEWERS TO FOLLOW

The task of a reviewer is to professionally evaluate and scrutinise in detail a submitted paper including in the review the following elements: general structure and organisation of the paper, particular aspects, the reviewer's conclusion and recommendation.

?	General structure – entails an evaluation of the scope of the written material at hand (number of pages, tables, figures, illustrations, etc.), structural organisation (e.g. division into chapters), and a decision whether the structural units are well rounded and complete or lacking in information and content.

?	Particular aspects – entails an evaluation of the following: title, foreword, table of contents, introduction, individual chapters or sections of the paper, other enclosed materials and information (e.g. tables, drawings, photographs, charts, etc.) if any, references to literature, notes.

?	In addition to the above, the reviewer should supply his/her qualified opinion on the paper, together with suggestions to the author regarding improvements in areas such as the topicality and relevance of the subject under discussion, language and style (e.g. appropriate use of scientific/professional terminology), implementation of research methods, applicability of the author's findings in theory and practice, validity of the author's conclusion, adequacy of the summary, and literature used.

?	Conclusion and recommendation – contains an overall evaluation of the paper and a recommendation whether, and with what minor or major changes, it should be published, as well as proposing the category in which the paper is to be classed.


Although the conscientious reviewing of papers takes time, it is also a basis for ensuring high quality of the journal contents.

Thank  you  for  agreeing  to  review  the  papers  accepted  for  publishing  in  the  journal
"Sigurnost".
