# Supplementary material for: Peer Review in Law Journals
Source: Front Res Metr Anal. 2021 Dec 8;6:787768. doi: 10.3389/frma.2021.787768 (PMC8692876; doi:10.3389/frma.2021.787768)
Supplement: Supplementary file 3 [file DataSheet2.ZIP › DOCUMENT - 0350-6886_1.RTF]

NOTES TO AUTHORS

The journal Sigurnost exists for the publication of papers that undergo review and are classified in the following categories: original scientific papers, preliminary communications, reviews, professional papers, and conference papers. Sigurnost has also published the papers that do not undergo review such as: considerations and comments, technical notes, accounts, and experiences.

The original scientific papers should contain the unpublished results of original research. Scientific information should be presented so that the experiment can be easily repeated obtaining results of equal accuracy or within the limits of the experimental error as quoted by the author himself and the accuracy of the analyses checked, methods and observations from which deduced and on which results are founded.
Preliminary communications should contain new scientific conclusions whose character suggests quick publishing. They should not make possible repetition of experiments and examination of the presented results.

Reviews are expected to contain complete surveys of a definite field or problem based on the already published material collected, analyzed, and discussed in the review.

Conference papers will, as a rule, be published only if not incorporated in the proceedings. The essentially modified and enlarged papers make an exception to the rule.
Professional papers should present useful contributions from the field of an applied science whose problems do not relate to an original research.
Considerations, comments, technical notes, accounts, and experiences should consist of short summaries of the activities and problems in the field of safety.

Every manuscript submitted for possible publication in the Sigurnost journal is first briefly reviewed by the editorial board. Notification of successful submission is sent. After initial screening the paper is passed on to at least two reviewers (one from abroad). Authors are invited to suggest up two possible reviewers in the field discussed in the article and the category of their papers. Authors are solely responsible for the contents of their papers. The review process can take up to eight weeks. Based on the comments of the reviewers, the editor will take a decision about the paper: accepting the paper, reconsidering the paper after changes or rejecting the paper.

The text has to be typed on the computer, with space between the lines and a larger space between individual paragraphs.

Papers, including illustrations, should neither be below 6 (six) nor exceed 16 (sixteen) pages (a maximum of 30 lines to each page). Editor can accept longer papers if required by the quality, actuality and contents.

0.	The paper must not have been previously published. The paper is to be sent via e-mail to: sigurnost@zirs.hr, with the text prepared as a Microsoft Word document. After submission to ZIRS, the paper shall not be offered for publication to others. Only exceptionally the Editor may accept an already published paper. We kindly request that papers written in English be edited and proofread prior to mailing.

0.	Full official address of the author and of the joint authors is to be written. The title of the paper should be clear and reasonably short.

0.	All communications should be written in the third person, phrases and passive forms avoided. Standard scientific and technical terms should be used.
0.	A short summary of the paper should also be provided. It should contain a general account, methods and observations, principal new conclusions and results. About 250 words is an optimum. Key words (the most important concepts from the text) should be quoted at the end of the summary.

0.	Illustrations, whether they are line drawings or photographic prints, should be titled and numbered consecutively through the paper using Arabic numerals. Any illustrations in electronic form, irrespective of whether they are incorporated in the text or not, need to be saved separately from the text under the extension jpg, tif or pdf. If at all possible, please enclose quality prints on paper of the illustrations.

0.	If any abbreviations are used their full name should be written in brackets when first used. Standard abbreviations of mathematical and other values should be used.
0.	References to the literature should be quoted by abc order of the authors' names and listed at the and.

Examples of reference quotations: for books:
Keros, P.: Temelji anatomije čovjeka, Medicinska naklada, Zagreb, 1977.

for proceedings:

Škanata, D., Čaldarović, O.: O percepciji rizika kod različitih sistema za proizvodnju električne energije. U: Zbornik poz-vanih radova, HND – Hrvatsko nuklearno društvo, Zagreb, 75–91, 1994.
for periodicals:

Vidaček, S., Radošević–Vidaček, B.: Biološki ritmovi i nesreće, Sigurnost, 38, 1996., 2, 107–115.
for internet sources:

Showa Glove Co., accessible at: http//www.bestglove. com, accessed: 15.4.2011.
Address of the Editor:
Zavod za istraživanje i razvoj sigurnosti d.o.o. SIGURNOST

Ul. grada Vukovara 68, p.p. 912, 10001 Zagreb Phone No.: (385–1) 615–60–90

Fax No.: (385–1) 611–98–12 sigurnost@zirs.hr
