# Supplementary material for: Peer Review in Law Journals
Source: Front Res Metr Anal. 2021 Dec 8;6:787768. doi: 10.3389/frma.2021.787768 (PMC8692876; doi:10.3389/frma.2021.787768)
Supplement: Supplementary file 3 [file DataSheet2.ZIP › DOCUMENT - 0352-342X.RTF]

UDRUGA PRAVNIK

Pravnik
časopis za pravna i društvena pitanja

Trg maršala Tita 3 Zagreb
tel. 01 45 97 533 fax. 01 45 97 521 casopis@pravnik.hr

OBRAZAC ZA RECENZIJU

NAZIV RADA: _____________________________________________________________________


AUTOR RADA: _____________________________________________________________________


RECENZENT: ______________________________________________________________________


PODACI O RADU:

___________________________________________________________________________________

___________________________________________________________________________________

___________________________________________________________________________________

___________________________________________________________________________________

___________________________________________________________________________________

MIŠLJENJE RECENZENTA:

___________________________________________________________________________________

___________________________________________________________________________________

___________________________________________________________________________________

___________________________________________________________________________________

___________________________________________________________________________________

___________________________________________________________________________________

___________________________________________________________________________________

___________________________________________________________________________________

___________________________________________________________________________________

___________________________________________________________________________________

___________________________________________________________________________________

___________________________________________________________________________________

___________________________________________________________________________________

DATUM:	POTPIS RECENZENTA:
