# Supplementary material for: Peer Review in Law Journals
Source: Front Res Metr Anal. 2021 Dec 8;6:787768. doi: 10.3389/frma.2021.787768 (PMC8692876; doi:10.3389/frma.2021.787768)
Supplement: Supplementary file 3 [file DataSheet2.ZIP › DOCUMENT - 0352-342X_1.RTF]

Pravila za objavu radova u časopisu Pravnik


KOJI SU PRIMARNI UVJETI ZA OBJAVU RADA?

• mora se raditi o stručnom ili znanstvenom radu pravne, politološke, sociološke, ekonomske

ili neke druge društvene tematike

	Uredništvu se uz rad mora dostaviti i recenzija/preporuka sveučilišnog nastavnika

	rad ne smije biti objavljivan u drugim stručno–znanstvenim publikacijama


ZAŠTO PRIJAVITI RAD ZA OBJAVU?

Posebno se potiče studente da prijavljuju svoje seminarske (ili druge) stručne i znanstvene radove za objavu u časopisu Pravnik. Dosad objavljivani radovi spadali su u red najboljih studentskih radova, često nagrađivanih Rektorovom ili Dekanovom nagradom. Pozitivnom selekcijom radova i objavljivanjem najboljih postiže se višestruka svrha.

Svake godine nagrađujemo najbolji studentski rad objavljen u časopisu („Nagrada Pravnik"). Studenti s objavljenim radom imaju prednost pri javljanju za različite stipendije

(primjerice, objavljen rad u časopisu Pravnik donosi dodatnih 0.2 boda na natječaju za stipendiju Zaklade Zlatko Crnić). Također, objavljeni rad koristi pri prijavi za određene projekte, postdiplomske studije i posebice studiranje u inozemstvu; časopis se redovito dostavlja profesorima, stručnoj javnosti, institucijama (sudovima, knjižnicama), dok ostali studenti koji čitaju Pravnik imaju dodatni izvor literature i inspiracije za pisanje vlastitih radova.


KAKO DO RECENZIJE?

Recenziju/preporuku može napisati vaš mentor (najčešće je to voditelj vaše seminarske grupe, mentor kod diplomskog), ali i bilo koji drugi sveučilišni nastavnik koji se bavi područjem kojim se bavi rad. Recenzija se uvijek slobodno i bez ustručavanja može zatražiti, štoviše, mnogi voditelji seminara će i sami na početku podsjetiti studente na mogućnost objave u Pravniku.

Recenzija se u pravilu piše na obrascu, čiji je download dostupan na web stranici: www.pravnik.hr. Recenziju/preporuku možete dobiti i ako nije napisana na navedenom


obrascu. Obrazac je smišljen kao pomoć recenzentima, a ne kao uvjet za uzimanje rada u obzir. Forma recenzije nije presudna.


KADA I KAKO DOSTAVITI RAD?

Nakon što ste dobili recenziju/preporuku, trebate dostaviti rad i recenziju našem uredništvu. Rad i recenziju potrebno je poslati na e-mail Udruge Pravnik: casopis@pravnik.hr, s tim da rad mora obvezano biti u MS Word obliku. Navedeni dokumenti za pojedini broj časopisa trebaju biti dostavljeni u roku koji objavi Udruga Pravnik (internet stranica, Facebook stranica itd.).


HOĆE LI MOJ RAD BITI SIGURNO OBJAVLJEN?

Nakon što ste predali rad, ocijenit će ga naše Uredništvo. Bude li primjedbi na sadržaj i formu (dužina rada, način citiranja, fusnote i sl.), moguće je da vam rad bude vraćen na izmjene ili dopune. Kada uredništvo ocijeni rad, obavijestit će vas je li rad prihvaćen za

objavljivanje u časopisu. Zbog ograničenog broja radova koje jedan broj časopisa može sadržavati, uredništvo pridržava pravo odlučiti koji će radovi biti objavljeni. Ako uredništvo ocjeni da se određeni rad ne može objaviti u predstojećem broju, moguće je da taj isti rad bude razmotren za objavu u idućem broju časopisa.

Predajom rada za objavu autor pristaje da se njegov rad objavi i u elektroničkom obliku unutar elektroničke baze podataka EBSCO čiji član je i časopis Pravnik kao i našem portalu takve vrste – „Hrčak".


KOJE FORMALNOSTI TREBA RAD ZADOVOLJAVATI?

Maksimalno tri osobe mogu biti autori jednoga rada.

Preporučuje se da rad ne bude duži od 50 kartica teksta (jedna kartica iznosi 1800 znakova, prored od 1.5 cm te najčešće font 12 u Microsoft Wordu).

Potrebno je uz rad predati i sažetak (summary) na hrvatskom i jednom od svjetskih jezika dužine do 300 riječi, s ključnim riječima rada (do 5) na oba jezika. Naslov rada također mora biti preveden na isti jezik kao i sažetak.

Popis literature nije obvezno pisati jer se oni ne objavljuju, iako je poželjno jer olakšava

posao uredništvu.

Traži se dosljednost stila citiranja kroz cijeli rad, na sljedeći način navode se:

0.	Knjige - autor, naslov, mjesto izdanja, godina izdanja, stranica (npr. P. KLARIĆ, M. VEDRIŠ, Građansko pravo, Zagreb, 2008., str. 635.).

0.	Članci – autor, naslov članka, naslov časopisa, broj i godina izdanja, godište, stranica (npr.

B. VUKAS, Pomorski zakonik Republike Hrvatske i međunarodno pravo mora, Zbornik

Pravnog fakulteta u Zagrebu, 1-2/2008, 58, str. 199-200.).

3. Propisi – naziv, službeno glasilo, vrijeme (npr. Zakon o sustavu državne uprave, NN

190/03, 199/03, 79/07.). Dalje u tekstu poželjno je koristiti kraticu za propis te to naznačiti (u našem primjeru: nadalje - ZSDU).

0.	Izvori s Interneta – naziv izvora, puna adresa stranice i datum zadnjeg pregleda u zagradi (npr. definicija „zdravlja" Svjetske zdravstvene organizacije (WHO definition of health), www.who.int/about/definition/en/print.html, (29. 01. 2009.))

0.	Ponovljeni citati – npr. KURTOVIĆ, op. cit. (bilj. 5), str. 76.

Za ostale citate (ponovljeni citati, sudska praksa itd.) upućujemo na „Upute za pisanje studentskih radova" prof. dr. sc. Alana Uzelca i analognu primjenu stila navedenog u primjerima. Također autorima skrećemo pozornost na 87. broj časopisa Pravnik u kojem je po prvi put prakticiran ovakav jedinstveni stil citiranja.


ZAŠTO OBJAVITI RAD NA STRANOM JEZIKU?

Povodom pristupanja Pravnika u svjetsku elektroničku bazu podataka EBSCO, uredništvo potiče objavljivanje radova na nekom od svjetskih jezika, jer objava takvih radova ima izniman značaj pri prijavi za studiranjem ili usavršavanjem u inozemstvu.


Imate li još kakvih pitanja ili vas nešto zanima, slobodno nam se obratite na e-mail: udruga@pravnik.hr
