# Supplementary material for: Peer Review in Law Journals
Source: Front Res Metr Anal. 2021 Dec 8;6:787768. doi: 10.3389/frma.2021.787768 (PMC8692876; doi:10.3389/frma.2021.787768)
Supplement: Supplementary file 3 [file DataSheet2.ZIP › DOCUMENT - 0352-5317.RTF]

Prikazi knjiga: Ivo Grabovac, Slobodan Kaštela, Međunarodni i nacionalni izvori hrvatskoga prometnog prava, Odabrana poglavlja..., PPP god. 52 (2013), 167, str. 236-238


INSTRUCTIONS FOR AUTHORS

Poredbeno pomorsko pravo = Comparative Maritime Law (CML), ISSN 1331-9914

(Print); ISSN 1848-8927 (Online), http://doi.org/10.21857/mjrl3uwg79 is a peer-reviewed open access law journal published annually by the Croatian Academy of Sciences and Arts – Adriatic Institute (Zagreb, Croatia).

The journal is the key periodical in the field of maritime law and the law of the sea in Croatia, but also in the region. It has an international editorial board and all the articles are peer reviewed by the leading local and foreign experts and academics.

The CML publishes materials related to the fields of the international law of the sea and maritime law, including shipping and transport law, marine and transport insurance law, maritime labour law, protection of marine environment, maritime safety and security and similar. The Editorial board shall accept only unpublished materials. The articles accepted for the publication or already published in the CML, may be published in other publications only subject to the approval of the Editorial board, and in that case with a clear reference to the original publication in the CML.

The academic articles are subject to the blind peer review procedure. Each article shall be reviewed by two peer reviewers proposed by the Editorial board and nominated by the Academy's Department of Social Sciences. Depending on the reviewers' evaluations, the articles shall be categorized as:

0)	original scientific paper – the paper which is characterised by originality of conclusions, or which presents previously unpublished original results of scientific research;

0)	preliminary communication – the paper which presents primary findings of research in progress, which due to current interest require immediate publication, but without the level of deep and thorough study required for the scientific paper;

0)	review article – the article which contains detailed and comprehensive critical review of a certain problem area, but with no significant originality of results, and

0)	professional paper – the paper which contains information and experience relevant for a certain profession, but without scientific characteristics.


362


Prikazi knjiga: Ivo Grabovac, Slobodan Kaštela, Međunarodni i nacionalni izvori hrvatskoga prometnog prava, Odabrana poglavlja..., PPP god. 52 (2013), 167, str. 236-238


The reviews shall be anonymous and the reviewers shall communicate their comments, suggestions and corrections to the authors exclusively via the Editorial board.

Other materials, such as professional translations, book reviews, case law commentaries or digests, reports etc. shall not be subject to the peer review but only to the editing process.

Upon the publication, the Editorial board shall retain the right to the printed and electronic version of the published material.

Each manuscript shall contain the following elements:

-	title (in the original language and in English),

-	author's details (name and surname, academic title, professional position, personal e-mail address, name and address of the home institution),

-	abstract (in the original language and in English, app. 250 words),

-	key words (in the original language and in English),

-	body text,

-	bibliography/list of references (according to the customary academic rules of citation).

The materials for publication are to be submitted to:

Jadranski zavod Hrvatske akademije znanosti i umjetnosti

/ Adriatic Institute, Croatian Academy of Sciences and Arts / Augusta Šenoe 4, 10000 Zagreb, Croatia
tel: +385 1 49 20 733 tel/fax: + 385 1 48 12 703 e-mail: jz@hazu.hr


363
