# Supplementary material for: Peer Review in Law Journals
Source: Front Res Metr Anal. 2021 Dec 8;6:787768. doi: 10.3389/frma.2021.787768 (PMC8692876; doi:10.3389/frma.2021.787768)
Supplement: Supplementary file 3 [file DataSheet2.ZIP › DOCUMENT - 0353-359X (2).RTF]

Publication Ethics
 
Etička načela za časopis Ekonomski Vjesnik / Econviews – Review of Contemporary Business, Entrepreneurship and Economic Issues temelje se na smjernicama za najbolju praksu za urednike časopisa Udruge za etiku objavljivanja (COPE – Committee on Publication Ethics).  Uredništvo izjavljuje da će postupati sukladno prihvaćenim međunarodnim načelima.
Usvojena etička načela obvezujuća su za Uredništvo, recenzente i autore.
 
Uredništvo
Glavni urednik donosi konačnu odluku o tome hoće li rad biti prihvaćen i objavljen. Kriterij za izbor je kvaliteta i značaj radova bez obzira na rasu, spol, nacionalnu pripadnost, vjerska ili politička uvjerenja autora. Uredništvo ne smije otkriti bilo kakve informacije o podnesenim radovima niti ih smije koristiti u druge svrhe.
 
Autori radova
Svi autori navedeni u jednom radu moraju dati značajan intelektualni doprinos istraživanju, odnosno pisanju rada. Predaja rukopisa podrazumijeva da su svi suautori, ako postoje, kao i nadležna tijela u ustanovi u kojoj se provodi istraživanje, prešutno ili izrijekom, suglasni s njegovim objavljivanjem. Izdavač ne snosi odgovornost za bilo kakav zahtjev za naknadu. Podneseni radovi ne bi smjeli biti ranije objavljeni niti predani na razmatranje za objavljivanje nekamo drugdje.
 
Recenzenti
Radovi podliježu tzv. dvostrukoj slijepoj recenziji (eng. double-blind review) kojom se ne otkrivaju identiteti autora i recenzenta. Recenzent je dužan odbiti recenziju ukoliko smatra da nije kvalificiran recenzirati dostavljen rad ili nije u mogućnosti u roku od 6 tjedana izraditi recenziju. Recenzent je dužan upoznati urednika s bilo kakvim potencijalnim sukobom interesa ili nepravilnostima vezanima za rad. Svaki rad koji se prima na recenziju treba tretirati kao povjerljiv dokument.
 
EKONOMSKI VJESNIK / ECONVIEWS : Review of contemporary business, entrepreneurship and economic issues pridržava se standarda uredničkog rada i etičkog postupnika za urednike koji su objavljeni na stranicama Ministarstva znanosti, obrazovanja i sporta Republike Hrvatske.
 
| 06.10.2014.


Akreditacije
         
Mapa portala
 
·	Naslovna·	 
·	Misija i vizija Fakulteta·	 
·	Katedre·	 
·	Nastavnici prema abecedi·	 
·	Raspored sati·	 
·	Ured za studente i studije – Referada·	 
·	Gdje smo?·	 
·	Kontaktirajte nas·	 
EFOS popularno
 
·	stipendije.info·	 
·	coursera.org·	 
·	P2PU·	 
·	TED Talks·	 
·	Bloomberg Institute Khan Academy·	 
Radno vrijeme
 
·	Radno vrijeme referade 
·	Radnim danom od 9 do 12 sati 
·	Utorkom (samo za izvanredne studente) 
·	od 16 do 18.00 sati 
·	Radno vrijeme knjižnice 
·	Ponedjeljak - Četvrtak: 08:00-20:00 
·	Petak: 08:00-15:00 
·	Radno vrijeme skriptarnice 
·	Ponedjeljak–petak: 9:00h–12:00h 
·	Utorak: 9:00h–12:00h i 16:00h-18:00h 
Poveznice
             
Sva prava pridržana 2007-2021; Sveučilište J.J. Strossmayera u Osijeku, Ekonomski fakultet u Osijeku, Trg Lj. Gaja 7, Osijek, 031/224-400
OIB: 52778515544 | Matični broj: 03021645 | IBAN Ekonomskog fakulteta u Osijeku: HR43 2500 0091 1020 1337 6
