# Supplementary material for: Peer Review in Law Journals
Source: Front Res Metr Anal. 2021 Dec 8;6:787768. doi: 10.3389/frma.2021.787768 (PMC8692876; doi:10.3389/frma.2021.787768)
Supplement: Supplementary file 3 [file DataSheet2.ZIP › DOCUMENT - 0353-359X.RTF]

UNIVERSITY OF JOSIP JURAJ STROSSMAYER OF OSIJEK

FACULTY OF ECONOMICS IN OSIJEK

EKONOMSKI VJESNIK / ECONVIEWS

Review of Contemporary Business, Entrepreneurship and Economic Issues


REVIEW FORM


Notes for the Reviewers:

Submissions undergo a double blind review process, in which identities of author(s) and reviewer(s) are not disclosed. Reviewers should decline a review request if they lack sufficient expertise, or if they are unable to complete the review within six weeks. Reviewers must inform the editor of any possible conflict of interest or irregularities connected to the paper under review. Any paper received for review must be treated as a confidential document.


REVIEWER


Name and surname:

Affiliation:

Home address:

Scientific area, field, branch:

Academic title:

Contact:


TITLE  OF  THE

PAPER UNDER

REVIEW


1


The title is appropriate for the content of the paper.	YES	NO		
	(mark the appropriate box with "+")				
STATEMENT OF					
REASONS					
(only if negative)					
The paper contains all the essential elements.	YES	NO		
	(mark the appropriate box with "+")				
STATEMENT OF					
REASONS					
(only if negative)					
The introduction clearly states the topic and the approach to the issue.	YES	NO		
	(mark the appropriate box with "+")				
STATEMENT OF					
REASONS					
(only if negative)					
The author builds upon previous research in the chosen field.	YES	NO		
	(mark the appropriate box with "+")				
STATEMENT OF					
REASONS					
(only if negative)					
	Appropriate methodology has been used.	YES	NO		
	(mark the appropriate box with "+")				
STATEMENT OF					
REASONS					
(only if negative)					
	The conclusion contains clearly stated:	YES	NO		
	(mark the appropriate box with "+")				
					
	scientific claims				
	open questions				
	suggestions for further research.				
STATEMENT OF					
REASONS					
(only if negative)					
References used in the paper are appropriate to the topic.	YES	NO		
	(mark the appropriate box with "+")				
STATEMENT OF					
REASONS					
(only if negative)					
	The paper can be qualified as:	YES	NO		
	(mark the appropriate box with "+")				
					
	topical/current				
	contributing to the research field				
corresponding with the scope of the journal				
STATEMENT OF					
REASONS					
(only if negative)					


2


SUGGESTED CATEGORIZATION
(mark the appropriate box with "+")

ORIGINAL SCIENTIFIC PAPER

PRELIMINARY COMMUNICATION

REVIEW PAPER

PROFESSIONAL PAPER


BRIEF STATEMENT

OF REASONS


FINAL RECOMMENDATION (ACCEPTABILITY FOR PUBLICATION)
(mark the appropriate box with "+")

Accept the paper.

Accept the paper provided it is revised according to reviewer's suggestions (minor revisions).

Suggest significant revisions to the author(s) and submit the paper for re-review.

Reject the paper.


REMARKS TO THE AUTHOR(S)


COMMENT TO THE EDITOR (not visible to authors)


3


SPECIFIC DATES:

Paper received by the reviewer:

Review completed:

Paper re-read:

Paper rejected:


Reviewer's signature:


4
