# Supplementary material for: Peer Review in Law Journals
Source: Front Res Metr Anal. 2021 Dec 8;6:787768. doi: 10.3389/frma.2021.787768 (PMC8692876; doi:10.3389/frma.2021.787768)
Supplement: Supplementary file 3 [file DataSheet2.ZIP › DOCUMENT - 0390-6744.RTF]

RIVISTA DI STORIA DEL DIRITTO ITALIANO

Codice Etico


Il Codice etico della «Rivista di Storia del diritto italiano» risponde alle vigenti linee-guida adottate dalla Committee on Publication Ethics (COPE) circa le pubblicazioni scientifiche. In particolare esso prevede che tutte le parti coinvolte – Direzione, autori e revisori – conoscano e condividano i seguenti principi etici.


-	Doveri della Direzione e dei redattori

Decisioni sulla pubblicazione

La Direzione è responsabile della decisione di pubblicare o meno gli articoli proposti alla rivista e si giova della consultazione con i referee per tale decisione.

Correttezza

La decisione è presa in base al valore scientifico, alla rilevanza ed all'originalità del contenuto, nonché al pieno rispetto delle regole editoriali della rivista, senza discriminazioni di razza, genere, religione, origine etnica, cittadinanza, orientamento sessuale, orientamento politico degli autori.

Riservatezza, conflitto di interessi e divieto di utilizzazione o divulgazione

La Direzione e le persone con essa redattrici della rivista si impegnano a non rivelare informazioni sugli articoli proposti per la stampa a persone diverse dall'autore, dai referee e dall'editore o stampatore ed a non utilizzare i dati ivi esistenti per ricerche proprie, senza l'espresso consenso scritto dell'autore.

La Direzione affida in modo anonimo a due referenti di fiducia competenti l'articolo presentato per averne una valutazione adeguata al fine della pubblicazione sulla rivista.


-	Doveri degli autori degli articoli inviati alla rivista

Originalità scientifica

Gli autori sono tenuti a comporre un lavoro scientificamente originale in ogni sua parte ed a citare adeguatamente i testi utilizzati seguendo le regole editoriali indicate per la pubblicazione sulla rivista.

Non devono essere proposti contributi editi o in corso di pubblicazione in altri periodici o volumi, salvo espresso consenso della Direzione. Se l'articolo sarà pubblicato in seguito in altri periodici o volumi dovrà esserne consenziente la «Rivista di storia del diritto italiano»

e dovrà essere fatto espresso riferimento alla pubblicazione sulla «Rivista di storia del diritto italiano».

Integrazione dell'opera

La Direzione potrà chiedere agli autori le correzioni e integrazioni ritenute opportune, anche a seguito della valutazione anonima dei referee. In determinati casi, la Direzione potrà portare a conoscenza dell'autore il predetto giudizio anonimo. Gli autori possono esprimere in proposito le loro osservazioni o obiezioni, su cui deciderà poi la Direzione.

La Direzione si riserva di procedere a modificazioni meramente editoriali dopo la prima correzione delle bozze, per l'omogeneità di pubblicazione della rivista.

Paternità dell'opera

La paternità dell'opera deve risultare con chiarezza: devono apparire come coautori tutti coloro che abbiano dato un contributo significativo alla realizzazione dell'articolo. Deve pure essere esplicitamente riconosciuto il contributo dato da altre persone in modo significativo ad alcune fase della ricerca. Nel caso di contributi a più mani, deve risultare correttamente con chiarezza la quota-parte di ogni coautore.

Conflitti di interesse

Gli autori non devono avere conflitti d'interesse che potrebbero aver condizionato i risultati conseguiti o le interpretazioni proposte. Gli autori devono inoltre indicare gli eventuali finanziatori della ricerca o del progetto da cui deriva l'articolo.

Rimedio ad inesattezze dell'articolo

Se un autore individua in un suo articolo un errore o un'inesattezza rilevante, deve informare con tempestività la rivista e fornire tutte le indicazioni necessarie per segnalare in calce o in appendice dell'articolo le doverose correzioni. Egli deve inoltre provvedere qualora la segnalazione gli giunga dalla Direzione della rivista, salvo accordo con essa.


-	Doveri dei referee

Contributo alla decisione editoriale

La peer-review è un procedimento che aiuta la Direzione della rivista a valutare gli articoli proposti e consente anche all'autore di migliorare il proprio contributo.


Rispetto dei tempi

Il referee che non si senta adeguato all'incarico richiesto o che presume di non poter rispondere nei tempi indicati è tenuto a comunicarlo con la massima urgenza alla Direzione della rivista.

Correttezza ed oggettività

La peer-review dev'essere svolta in modo corretto ed oggettivo. I referenti sono invitati a motivare le proprie valutazioni sull'articolo loro inviato in modo adeguato. Ogni giudizio personale sull'autore è inopportuno.


Eventuale indicazione di testi

Il referente è invitato ad indicare con precisione gli estremi bibliografici di opere basilari trascurate dall'autore nell'articolo. Egli deve inoltre segnalare eventuali somiglianze o sovrapposizioni del testo ricevuto in lettura con altre opere a lui note.

Conflitto d'interessi e divulgazione

Informazioni riservate o indicazioni ottenute durante il procedimento di peer-review devono essere ritenute fiduciarie e confidenziali: non possono essere usate a proprio interesse. I referee sono tenuti a non accettare in lettura articoli per i quali emerga un conflitto di interessi dovuto a precedenti specifici rapporti di collaborazione o concorrenza con l'autore (di per sé ignoto, ma comunque individuato per deduzione) e /o con la relativa istituzione di appartenenza.
