# Supplementary material for: Peer Review in Law Journals
Source: Front Res Metr Anal. 2021 Dec 8;6:787768. doi: 10.3389/frma.2021.787768 (PMC8692876; doi:10.3389/frma.2021.787768)
Supplement: Supplementary file 3 [file DataSheet2.ZIP › DOCUMENT - 0390-9522.RTF]

﻿NICCOLÒ SALANITRO 
La  SEDE  della  Rivista  è  presso  Giuseppe  Santoni,  Corso  Rinascimento  11, 00186, Roma, tel. 06/68301139 - fax 06/68214752 
Direzione 
Mario  Campobasso  ( mario_campobasso@hotmail.com )  -  Aldo  A.  Dolmetta (aadolmetta@gmail.com) - Giuseppe Santoni (giussant@me.com) - Ruggero Vigo (vigo@unict.it). 
Comitato Scientifico 
Peter  Kindler  (München)  -  Federico  Martorano  -  Giuseppe  B.  Portale  -  Juan  
Sanchez Calero Guilarte (Madrid) 
Responsabile di Redazione 
Antonella Sciarrone Alibrandi (antonella.sciarrone@unicatt.it) 
Comitato di Redazione 
Francesco Barachini - Federico Briolini - Antonio Cetra - Marco Cian - Paoloefisio  
Corrias  -  Concetto  Costa  -  Federico  Ferro-Luzzi  -  Sabino  Fortunato  -  Enrico  
Gabrielli - Gianvito Giannelli - Enrico Ginevra - Bruno Inzitari - Raffaele Lener  
-  Daniele  Maffeis  -  Francesco  S.  Martorano  -  Pietro  Masi  -  Aurelio  Mirone  -  
Umberto Morera - Federico Mucciarelli - Gustavo Olivieri - Andrea Perrone - Luca  
Pisani - Luca G. Radicati di Brozolo - Matteo Rescigno - Massimo Rubino de Ritis  
- Luigi Salamone - Renato Santagata - Vittorio Santoro - Giuliana Scognamiglio  
- Sergio Seminara - Andrea Tucci. 
Redazione di Bari-Foggia: Vincenzo Vito Chionna - Mariella Cuccovillo. 
Redazione di  Bologna:  Giovanni B. Barillà - Marco Lamandini - Caterina Pasquariello. 
Redazione di Cagliari-Sassari: Alessandra Camedda - Federico Cappai - Corrado  
Chessa - Monica Cossu - Federico Onnis Cugia - Elisabetta Piras. 
Redazione di Catania: Enrico Macrí - Renato Mangano -  Ugo Salanitro. 
Redazione di  Milano: PierDanilo Beltrami - Francesca Benatti - Francesco Bordiga -  
Vincenzo De Stasio - Claudio Frigeni - Mariasofia Houben - Ugo Malvagna - Gianni  
Mignone - Paolo Mondini - Gianluca Mucciarone - Luca Purpura - Marco Speranzin   
- Piergiuseppe Spolaore - Silvia Vanoni. 
Redazione  di  Napoli:  Antonio  Blandini  -  Nicola  de  Luca  -  Maria  Consiglia  di  
Martino - Emanuela Fusco - Paolo Ghionni Crivelli Visconti - Gian Paolo La Sala -  
Antonio Maria Marzocco - Camillo Patriarca - Lucia Picardi. 
Redazione  di  Padova-Venezia-Verona:  Peter  Agstner  -  Maurizio  Bianchini  -  
Carmelita Camardi - Lorenzo De Angelis - Giuliana Martina - Giovanni Meruzzi -  
Andrea Minto - Linda Miotto - Federica Pasquariello - Carlotta Rinaldo - Claudia  
Sandei - Alberto Urbani. 
Redazione di Pisa: Amal Abu Awwad - Ilaria Kutufà - Gabriele Nuzzo - Vincenzo  
Pinto 
Redazione  di  Roma:  Francesco  Accettella  -  Elisa  Brodi  -  Grazia  Monia  Buta  
-  Nicoletta  Ciocca  -  Manuel  Franchi  -  Paola  Lucantoni  -  Manuela  Natale  -  
Maddalena Rabitti - Manuela Tola. 
Comitato  dei  Referees:  Antonella  Antonucci  -  Gino  Cavalli  -  Mario  Cera  -  
Marcello Clarich - Renzo Costi - Ombretta Di Giovine - Guido Ferrarini - Andrea  
Guaccero - Fabrizio Guerrera - Massimo Miola - Gianmaria Palmieri - Roberto  
Pennisi  -  Paolo  Piscitello  -  Mario  Porzio  -  Gaetano  Presti  -  Filippo  Ranieri (Saarbrücken) - Guido Rossi - Roberto Sacchi - Piero Schlesinger - Maurizio Sciuto -   
Mario Stella Richter - Giuseppe Terranova - Francesco Vella. 
La direzione è responsabile della pubblicazione degli articoli e delle note a sentenza. I contributi sono pubblicati previa valutazione di un  referee scelto tra  i  componenti  del  comitato  dei  referees  o,  all’occorrenza,  fra  professori ordinari  della  materia.  I  contributi  saranno  sottoposti  a  tale  valutazione senza indicare l’identità dell’Autore. Il  referee compila una scheda di valutazione conservata dalla direzione. Se il valutatore, nel suo giudizio, suggerisce integrazioni  o  modifiche,  il  contributo  sarà  pubblicato  previo  controllo dell’adeguamento richiesto. 
L’AMMINISTRAZIONE   è  presso  la  Casa  Editrice  Dott.  A.  GIUFFRÈ  
EDITORE S.p.A. via Busto Arsizio, 40 - 20151 Milano tel.  02/38.089.200  -  fax  02/38089432  -  Internet:  http://www.giuffre.it e-mail: vendite@giuffre.it 
PUBBLICITÀ: Dott. A.  Giuffrè  Editore  S.p.A.  -  Servizio  Pubblicità  via  Busto  
Arsizio,  40  -  20151  Milano  -  tel.  02/38.089.335  -  fax  02/38089426  e-mail:  
periodici@giuffre.it 

BANCA BORSA 
TITOLI DI CREDITO 

CONDIZIONI DI ABBONAMENTO PER IL 2017 
Unione europea  .................................................................. E 150,00 
Paesi extra Unione europea .............................................. E 225,00 
Prezzo di un singolo numero  ............................................ E  25,00 (Extra U.E. E 38,00) 
Abbonamento sostenitore  .................................................. E 310,00 
Le annate arretrate a fascicoli, dal 2011 fino al 2016, sono disponibili fino ad esaurimento scorte. 

In  caso  di  sottoscrizione  contemporanea  a  due  o  più  riviste  cartacee  tra quelle qui di seguito indicate  sconto 10% sulla quota di abbonamento: 
  Unione europea  Paesi extra 
    Unione europea 
Banca Borsa e Titoli di Credito  E 150,00  E 225,00 
Diritto del Commercio Internazionale  E 155,00  E 232,00 
Giurisprudenza Commerciale  E 190,00  E 285,00 
Rivista dei Dottori Commercialisti  E 140,00  E 210,00 
Rivista delle Società  E 150,00  E 225,00 
Gli sconti non sono cumulabili. 

RIVISTA ON-LINE ALL’INTERNO   
DI “BIBLIOTECA RIVISTE” DAL 1975 
U.E.  abbonato   E 44,00*  non abbonato   E 123,00* 
*IVA esclusa 
La  rivista  on-line  riproduce,  in  pdf,  i  contenuti  di  ogni  fascicolo dall’anno indicato fino all’ultimo numero in pubblicazione. 
La  sottoscrizione  dell’abbonamento  “abbonato”  alla  rivista  cartacea  garantisce  un  accesso  al  contenuto  dal  1  gennaio  2017,  o  da qualunque data successiva di sottoscrizione, fino al 31 dicembre 2017. 
La  sottoscrizione  dell’abbonamento  “non  abbonato”  alla  rivista cartacea garantisce un accesso di 365 giorni dalla data di sottoscrizione. 
Il  sistema  on-line  Biblioteca  Riviste  permette  la  consultazione  dei fascicoli attraverso ricerche: 
UÊvÕÊÌiÝÌ 
UÊ«iÀÊiÃÌÀiÊ`Ê«ÕLLV>âiÊ­ÕiÀÊiÊ>Êv>ÃVV® 
UÊ«iÀÊ`>Ì> 

L’abbonamento alla rivista cartacea decorre dal 1° gennaio di ogni anno e dà diritto a tutti i numeri relativi all’annata, compresi quelli già pubblicati. 
Il pagamento può effettuarsi direttamente all’Editore: 
ÊU Ê VÊÛiÀÃ>iÌÊÃÕÊ c.c.p. 721209, indicando chiaramente gli estremi dell’abbonamento; ÊU Ê a ricevimento fattura (riservata ad enti e società); ÊU Ê >VµÕÃÌÊiÊÌÀ>ÌiÊÃÌÊºÃ«°}ÕvvÀi°Ì» ; ÊU Ê ««ÕÀiÊÌÀ>ÌiÊ}Ê Agenti Giuffrè a ciò autorizzati (cfr. pagine gialle). 
Il rinnovo dell’abbonamento deve essere effettuato entro il 31 marzo di ciascun anno. 
I  fascicoli  non  pervenuti  all’abbonato  devono  essere  reclamati  al  ricevimento del fascicolo successivo. Decorso tale termine si spediscono, se disponibili, contro rimessa dell’importo. 
Le comunicazioni in merito a mutamenti di indirizzo vanno indirizzate all’Editore. 
Per ogni effetto l’abbonato elegge domicilio presso la “Dott. A. Giuffrè Editore  
SpA” - Via Busto Arsizio, 40 - 20151 Milano. 
Ai collaboratori sono inviati gratuitamente 25 estratti degli articoli e delle note a sentenze. 

I contributi pubblicati in questa rivista potranno essere riprodotti dall’Editore su altre, proprie pubblicazioni, in qualunque forma. 

Registrazione presso il Tribunale di Milano al n. 754 del 28 ottobre 1948 
R.O.C. n. 6569 (già RNS n. 23 vol. 1 foglio 177 del 2/7/1982) 
Direttore responsabile: Dott. Matteo Bianchi 

  Rivista associata 
