# Supplementary material for: Peer Review in Law Journals
Source: Front Res Metr Anal. 2021 Dec 8;6:787768. doi: 10.3389/frma.2021.787768 (PMC8692876; doi:10.3389/frma.2021.787768)
Supplement: Supplementary file 3 [file DataSheet2.ZIP › DOCUMENT - 0391-187X.RTF]

RESPONSABILITÀ CIVILE E PREVIDENZA

Rivista bimestrale di dottrina, giurisprudenza e legislazione diretta da Giovanni Iudica e Ugo Carnevali

direttore responsabile Giovanni Iudica


Codice etico di pubblicazione

Responsabilità civile e previdenza è una rivista scientifica, edita da Giuffrè sin dal 1935 ed ora da Giuffrè Francis Lefebvre, con peer-review di tipo double-blind, dotata di codice etico secondo le indicazioni elaborate da COPE (Committee on Publication Ethics).

Tutte le parti coinvolte – direzione, comitato scientifico, comitato editoriale, comitato di valutazione (referee), autori – conoscono e condividono i requisiti etici richiesti.

Direzione

La direzione decide le linee e le ricerche scientifiche da privilegiare, valuta gli articoli ricevuti dal comitato editoriale e decide sui successivi passaggi relativi alla pubblicazione, supportata dall'opinione del comitato scientifico e dal parere dei referee.

La direzione valuta i contributi considerando unicamente il loro contenuto, senza alcuna discriminazione di genere, orientamento sessuale, religione, origine etnica, razza, cittadinanza o orientamento politico degli autori.
La decisione ultima circa la pubblicazione dei contributi spetta al direttore responsabile

Comitato scientifico

Il comitato scientifico supporta la direzione e il comitato editoriale nella valutazione delle linee scientifiche e della qualità complessiva dei contributi pubblicati.

Comitato editoriale

Il comitato editoriale concorre in maniera continuativa ad alimentare la Rivista di contributi scientifici, esercita l'attività di editing, si impegna alla riservatezza dei contributi ricevuti e a non divulgare alcuna informazione a nessun altro soggetto diverso dagli autori, revisori potenziali o effettivi, direzione, comitato scientifico, eventuali altri membri del comitato editoriale ed editore.

Il comitato editoriale si impegna a non divulgare l'identità degli autori ai referee e viceversa.

Comitato di valutazione

Il parere dei revisori esterni aiuta il comitato editoriale e la direzione ad assumere decisioni sulla pubblicazione degli articoli ricevuti e può suggerire all'autore gli adeguamenti necessari al loro miglioramento.

Il referee che ritenga non essere adeguato alla valutazione di un contributo sottoposto alla sua attenzione, o che sappia di non poterla svolgere nei tempi indicati, è tenuto ad informare tempestivamente il comitato editoriale.

Il referee si impegna a valutare obiettivamente il materiale ricevuto, motivando i giudizi espressi in maniera chiara ed esplicita.

Il referee si impegna, inoltre, a non divulgare il materiale ricevuto in valutazione, da considerarsi riservato, a non discuterne i testi con altre persone senza autorizzazione della direzione/comitato editoriale e a rifiutare la proposta di revisione di articoli sui quali possano sorgere conflitti di interesse.

Regole di autodisciplina

0.	La valutazione dei contributi è affidata a due referee, membri del Comitato per la valutazione;
0.	Il contributo è inviato ai valutatori senza indicazione dell'identità dell'autore;

0.	L'identità dei valutatori di ciascun contributo è anonima;

0.	In caso di giudizio contrastante la Direzione si assume la responsabilità della decisione;

0.	Qualora sia formulato un giudizio positivo condizionato a revisione o modifica del contributo la

Direzione ne promuove la pubblicazione solo a seguito dell'adeguamento, assumendosi la responsabilità della verifica.

Autori

Gli autori si fanno garanti dell'originalità del loro lavoro e, qualora abbiano utilizzato testi altrui, che questi siano stati opportunamente citati nello scritto; inoltre si impegnano a dare la corretta indicazione delle pubblicazioni citate nel loto contributo.

Gli autori non proporranno lo stesso articolo contemporaneamente a più di una rivista e si impegnano a non sottoporre alla Rivista lavori che hanno già ricevuto parere non favorevole.

Tutti coloro che hanno fornito un apporto significativo alla realizzazione dell'articolo devono essere indicati come coautori, riconoscendo esplicitamente il contributo di eventuali altre persone che hanno partecipato in maniera rilevante ad alcune fasi della ricerca.

L'autore che propone l'articolo per la pubblicazione deve assicurarsi che tutti i coautori siano stati opportunamente indicati, che abbiamo letto e approvato la versione finale dell'articolo e che siano d'accordo su un'eventuale pubblicazione.

Tutti gli autori devono dichiarare esplicitamente ogni conflitto di interesse significativo che potrebbe influenzare i risultati della ricerca o l'interpretazione dei dati. Gli autori devono indicare inoltre eventuali soggetti finanziatori della ricerca e/o del progetto.

Agli autori viene richiesto omettere dal proprio scritto, prima di essere sottoposto ai referee, qualsiasi riferimento che possa farli individuare come tali.
