# Supplementary material for: Peer Review in Law Journals
Source: Front Res Metr Anal. 2021 Dec 8;6:787768. doi: 10.3389/frma.2021.787768 (PMC8692876; doi:10.3389/frma.2021.787768)
Supplement: Supplementary file 3 [file DataSheet2.ZIP › DOCUMENT - 0391-5239.RTF]

Procedura di Revisione

I lavori che aspirano alla pubblicazione vengono inviati, per posta elettronica ed in formato Word, al seguente indirizzo: a uno degli indirizzi email dei Coordinatori di redazione o al Direttore Responsabile i quali dopo una prima delibazione del singolo lavoro, lo avvia al referaggio o revisione scientifica tra pari (peer review), dopo aver espunto dal testo ogni elemento idoneo a consentire l’identificazione dell’autore o dell’autrice.
Per contributi della Parte Prima della Rivista, nonché per i contributi della Parte Seconda, (salve le eccezioni che verranno illustrate infra), la revisione scientifica viene affidata, con il metodo del “doppio cieco”, ad uno studioso (referee) esterno alla Direzione scientifica ed alla Redazione della Rivista, e scelto sulla base della riconosciuta esperienza e competenza rispetto ai temi trattati nel lavoro stesso, nonché sulla base dell’assenza di conflitti d’interessi, da un lato, e di legami particolari (di parentela o affinità, di stretta amicizia, di colleganza nel medesimo Dipartimento o nella medesima Scuola accademica) con l’autore o con l’autrice, dall’altro.
I contributi destinati alle rubriche “Problemi della pratica” e “Osservatorio straniero”, possono essere pubblicati anche solo previo controllo di qualità effettuato dai direttori della Rivista.
Al referee viene richiesto di esprimere, entro il termine massimo di 15 giorni, la propria valutazione positiva ovvero negativa sul punto se il lavoro sottoposto al suo esame sia maturo per la  pubblicazione nella Rivista, formulando e motivando un breve giudizio scritto, che viene inviato alla Direzione scientifica, la quale ne cura l’inoltro, in maniera anonima, all’autore/autrice dell’articolo.
Nel caso in cui la valutazione del referee risulti negativa, e comunque in ogni caso in cui si reputi opportuno un riscontro della prima valutazione, la Direzione scientifica della Rivista può decidere collegialmente di interpellare, con le medesime modalità del primo, un secondo referee, oppure, la Direzione della Rivista ha la facoltà, qualora ritenga comunque utile la pubblicazione, di pubblicare il lavoro nelle rubriche non oggetto di referaggio, con specifica indicazione.
Le schede contenenti le valutazioni espresse dai referees vengono archiviate e conservate a cura della Direzione della Rivista.
In linea con le previsioni del Regolamento dell’ANVUR, non sono di regola sottoposti a revisione scientifica (peer review): (a) i lavori pubblicati, a firma della Direzione o di uno o più condirettori, nella rubrica “Editoriale”, trattandosi di lavori specificamente rivolti alla rappresentazione di una determinata linea editoriale della Rivista ovvero al lancio di una determinata proposta culturale, il cui merito non è perciò assoggettabile ad una valutazione esterna; (b) gli articoli scaturiti dalla partecipazione a seminari o convegni.
In casi eccezionali, la Direzione può assumere direttamente la responsabilità della pubblicazione di singoli contributi senza sottoporli a previa revisione scientifica, segnalando la circostanza e le relative motivazioni in una nota nella prima pagina del contributo.
