# Supplementary material for: Peer Review in Law Journals
Source: Front Res Metr Anal. 2021 Dec 8;6:787768. doi: 10.3389/frma.2021.787768 (PMC8692876; doi:10.3389/frma.2021.787768)
Supplement: Supplementary file 3 [file DataSheet2.ZIP › DOCUMENT - 0391-5646.RTF]

Archivio giuridico Filippo Serafini

Informazioni specifiche sulla Rivista: contenuti, periodicità, policy di accesso aperto (Open Access), procedura di revisione double-blind peer review, dichiarazione

di pubblicazione etica e di negligenza editoriale, elenco di Revisori, indicizzazione della Rivista

1 – Contenuti. Archivio giuridico Filippo Serafini è una Ri-vista interdisciplinare riconosciuta di Classe A dall'Agenzia di Valutazione del sistema Universitario e della Ricerca (AN-VUR) per l'Area 12 (Scienze Giuridiche). Pubblica contribu-ti considerati scientifici secondo le caratteristiche di ciascuna disciplina per l'originalità, l'ampiezza della trattazione, la cor-rettezza metodologica e dell'analisi critica, la ricchezza delle fonti e dell'informazione bibliografica nonché per la capacità di entrare in dialogo approfondito col dibattito nazionale e/o internazionale (ove rilevante per la disciplina).

2 – Periodicità. Secondo quanto programmato dalla Dire-zione, ogni anno sono pubblicati quattro fascicoli della Rivista nei mesi di marzo, giugno, settembre e dicembre.

3 – Sezioni. La Rivista si articola in quattro sezioni: Miscellanea, nella quale sono pubblicati contributi scientifici diver-si per contenuti e settore scientifico-disciplinare di riferimen-to che abbiano superato con esito positivo la procedura di revisione double-blind peer review; Fatti e giudizi, nella quale sono ospitati brevi interventi non sottoposti a valutazione circa que-stioni emergenti e resoconti di convegni o congressi; Rassegne e note, nella quale sono ospitate rassegne bibliografiche o giuri-sprudenziali e note giurisprudenziali; e Recensioni, nella quale sono recensite le principali novità editoriali in materia giuridi-ca (opere monografiche, volumi collettanei, ecc.).

4 – Policy di accesso aperto (Open Access). I contributi pub-blicati nella Rivista a partire dall'anno 2017, dopo un periodo

Archivio giuridico Filippo Serafini

di embargo di tre anni sono consultabili in modalità Open Access al fine di promuovere la pubblicazione e la divulgazione dei risultati della ricerca scientifica svolta da cultori del dirit-to italiani e stranieri.

5 – Procedura di revisione double-blind peer review.

`.	La Rivista giuridica interdisciplinare Archivio giuridico

Filippo Serafini, conformandosi al Regolamento per la classi-ficazione delle Riviste nelle aree non bibliometriche dell'Agen-zia Nazionale di Valutazione del sistema Universitario e del-la Ricerca (ANVUR) e alle guidelines elaborate dal Committee on Publication Ethics (COPE), adotta la procedura di revisione tra pari (peer review) con sistema doppiamente cieco (double blind): l'Autore non conosce l'identità dei Revisori e questi ultimi non conoscono quella dell'Autore.
`.	Le procedure di revisione sono formalizzate in modo da garantire integrità e etica della pubblicazione scientifica, tra-sparenza, autonomia dei Revisori e, in generale, assenza di conflitti di interesse. Ciascuna parte coinvolta nei processi di revisione dei contributi e di pubblicazione della Rivista (Auto-ri, Redattori, Revisori, Editore) è tenuta ad assicurare buone prassi di pubblicazione scientifica.

`.	La proposta di pubblicazione è trasmessa alla Direzio-ne della Rivista da uno dei componenti del Comitato diretti-vo o direttamente dall'Autore tramite: a) invio del contributo in lingua italiana o straniera; b) invio dell'abstract del contri-buto in lingua italiana ed inglese, con traduzione del titolo in lingua inglese e indicazione di parole chiave in lingua italiana ed inglese (dal minimo di 3 al massimo di 5). Abstract e parole chiave possono essere eventualmente redatte, oltre che in inglese, in un'altra delle principali lingue veicolari del dibat-tito scientifico.

`.	La Direzione effettua una selezione preliminare in ordine alle proposte da sottoporre alla valutazione, potendo de-cidere di non pubblicare contributi palesemente privi dei necessari requisiti di scientificità, originalità, pertinenza, senza discriminazioni basate su razza, origine etnica, cittadinanza,

Informazioni specifiche

orientamento religioso, politico o scientifico degli Autori. Per-tanto, ancora prima di procedere alla valutazione del contri-buto, la Direzione ne accerta la pertinenza all'ambito dei set-tori scientifico-disciplinari di riferimento, o valuta (in caso di pertinenza del contributo ad altri settori scientifici) se il tema trattato possa comunque essere di interesse per gli studiosi dei settori di riferimento della Rivista.

`.	La Redazione, in caso di valutazione positiva previamen-te espressa dalla Direzione della Rivista, invia il contributo dell'Autore in forma anonima a due Revisori, ad esso almeno pari, indicando il termine di consegna della scheda di valuta-zione debitamente compilata e firmata. I Revisori sono indivi-duati dalla Direzione tra studiosi, in ruolo e fuori ruolo, ita-liani e stranieri, esperti dei settori scientifico-disciplinari del-la Rivista, che siano disponibili ad esaminare in tempi brevi il contributo sottoposto alla loro valutazione, e che accettino espressamente i criteri e le modalità previste per l'espletamen-to del loro compito. I Revisori restano in carica fino a rinuncia o revoca dell'incarico. Non possono essere affidate revisioni di singoli articoli ai componenti della Direzione, del Comitato di-rettivo e della Redazione della Rivista. In casi eccezionali, la revisione del contributo può essere affidata ad uno dei compo-nenti del Comitato scientifico. La Direzione della Rivista, pur nel rispetto dell'anonimato dell'Autore e dei Revisori, assicura che a questi ultimi non siano sottoposti manoscritti rispetto ai quali abbiano o possano avere conflitti di interesse.

`.	Sono sottoposti a revisione i contributi pubblicati in cia-scun fascicolo della Rivista nella sezione Miscellanea. I con-tributi valutati riportano nella prima pagina in nota l'anno-tazione "Contributo sottoposto a valutazione". La Direzione, o il Comitato scientifico a maggioranza, può assumere in casi eccezionali direttamente la responsabilità della pubblicazio-ne, segnalando la circostanza e le relative motivazioni in una nota nella prima pagina del contributo. In particolare posso-no non essere sottoposti a valutazione i contributi: a) di Au-tori italiani e stranieri di riconosciuto prestigio accademico, o che ricoprano cariche di rilievo politico-istituzionale in organi

Archivio giuridico Filippo Serafini

nazionali, europei ed internazionali anche confessionali (essi riporteranno di regola in calce l'annotazione "Contributo ac-

cettato dalla Direzione per il comprovato prestigio scientifico dell'Autore"); b) già editi in altre Riviste o in lavori colletta-nei, di cui si chieda la pubblicazione con il permesso dell'Auto-re e dell'Editore della pubblicazione (con in calce l'annotazio-
ne "Contributo accettato dalla Direzione e pubblicato per cor-tesia dell'Autore e dell'Editore", l'indicazione degli estremi del-la Rivista o opera collettanea nelle quali il contributo è stato pubblicato e l'eventuale precisazione "già sottoposto a valuta-zione"); c) le relazioni a Congressi, Convegni, Tavole rotonde organizzate dalle associazioni scientifiche di riferimento della

Rivista, di rilevanza nazionale ed internazionale, per le qua-li non sia di fatto possibile osservare la regola dell'anonimato dell'Autore (con in calce l'annotazione "Il contributo, accetta-

to dalla Direzione per impossibilità di garantire l'anonimato

nella procedura di revisione tra pari, costituisce la Relazione tenuta al Congresso…"). Non sono sottoposti a revisione i con-tributi non rilevanti per le finalità che presiedono alla classifi-cazione delle Riviste, quali, ad esempio, le schede bibliografi-che, le rassegne storiografiche, le recensioni di volumi, gli in-terventi in forum e/o le discussioni scientifiche, gli editoria-li, le introduzioni o postfazioni di tipo meramente informati-vo, nonché tutto il materiale la cui paternità non è ascritta ad uno o più Autori.

g. Gli esiti della valutazione possono essere: a) "non pubbli-cabile"; b) "non pubblicabile se non rivisto, indicando motiva-tamente in cosa"; c) "pubblicabile dopo modifiche/integrazio-

ni, da specificare nel dettaglio"; d) "pubblicabile" (salvo even-tualmente il lavoro di editing per il rispetto dei criteri redazio-nali adottati dalla Rivista). L'esito della valutazione e i giudizi espressi dai Revisori sono comunicati all'Autore a cura della Redazione, nel rispetto dell'anonimato dei Revisori. Nell'ipo-tesi di cui alla lett. d), la Redazione comunica all'Autore l'esito della valutazione. Nelle ipotesi di cui alle lett. b) e c), i Revisori hanno il compito di identificare la presenza di riferimenti bibliografici rilevanti per il contributo sottoposto a valutazio-

Informazioni specifiche

ne, non citati ovvero non adeguatamente considerati dall'Au-tore. Qualora l'esito della valutazione sia "non pubblicabile se non rivisto, indicando motivatamente in cosa" (b) o "pubblica-bile dopo modifiche/integrazioni, da specificare nel dettaglio"

(c), i Revisori si rendono disponibili a valutare una seconda volta il manoscritto già esaminato, al fine di accertare se la nuova versione dello stesso manoscritto presentata dall'Auto-re possa considerarsi "pubblicabile".

`.	Sulla scorta dei dati della scheda e del giudizio sinteti-co espresso dai Revisori, la Direzione della Rivista, una vol-ta accertata l'osservanza dei criteri di valutazione e l'adempi-mento dei doveri dei Revisori, decide se pubblicare lo scritto, se chiederne la revisione o respingerlo in aderenza al giudizio espresso dai Revisori. In casi assolutamente eccezionali, la va-lutazione di "non pubblicabilità" dei Revisori potrà non essere vincolante, sempre che la Direzione e almeno due componenti del Comitato scientifico la ritengano non adeguatamente mo-tivata e decidano, pertanto, di sottoporre il contributo all'esa-me di un ulteriore Revisore.

`.	Qualora i giudizi espressi dai Revisori siano tra loro di-scordanti, la Direzione invia il contributo ad un terzo Revisore. Nel caso di giudizio negativo conforme da parte del ter-zo Revisore, il contributo non può essere pubblicato. Nel caso di giudizio positivo espresso dai primi due Revisori o, in caso di discordanza, di giudizio positivo conforme espresso dal ter-zo Revisore, la decisione finale sulla pubblicazione spetta alla

Direzione.

l. In caso di pubblicazione del contributo, la casa editrice fornirà, ai rispettivi Autori, 'estratto' degli articoli in forma-to elettronico pdf. Possono altresì essere forniti fascicoli carta-cei degli 'estratti', a pagamento. Chi fosse interessato è prega-to di richiedere preventivo di spesa a: info@mucchieditore.it.

Archivio giuridico Filippo Serafini

6 – Dichiarazione di pubblicazione etica e di negligenza editoriale.
a. Responsabilità editoriali della Direzione della Rivista. La Direzione è responsabile della decisione di pubblicare i contributi sottoposti dalla Rivista, secondo le politiche edito-riali della Rivista stessa e nel rispetto delle disposizioni di leg-ge vigenti.

Nell'assumere le proprie decisioni, la Direzione della Rivi-sta si avvale del supporto di almeno due Revisori esterni.

Se la Direzione ovvero uno o più componenti del Comitato direttivo, del Comitato scientifico e della Redazione della Ri-vista identifica o riceve una segnalazione di un problema ri-levante in merito a errori, imprecisioni, conflitto di interesse, controversie sulla paternità dell'opera (authorship disputes), ovvero a casi di cattiva condotta (misconduct) come riprodu-zione di testi altrui o riproposizione di testi propri già pubbli-cati (text recycling) o pubblicazione ridondante (redundant/ duplicate publication), che coinvolgano uno o più Autori, ne dà tempestiva comunicazione alla Direzione, all'Autore e all'E-ditore, in modo che si intraprenda ogni azione necessaria per chiarire la questione, espletando indagini e consentendo alla persona interessata di difendersi. La Direzione può, a secon-da dei casi, decidere di rifiutare il manoscritto (rejection) ov-vero, se il contributo è già stato edito, di pubblicare una cor-rezione (correction) o una ritrattazione (retraction), definendo la questione secondo le guidelines elaborate dal Commitee on Publication Ethics. Le misure adottate possono essere prece-dute dalla pubblicazione di un''espressione di preoccupazione' (expression of concern), qualora la Direzione reputi necessario avvisare i lettori in merito ad indagini ovvero ad azioni in cor-so, il cui esito potrebbe influenzare l'attendibilità dei contenu-ti pubblicati nella Rivista.

La Direzione garantisce l'indipendenza della procedura di revisione anche nelle ipotesi in cui siano pubblicati contributi di membri della Direzione, del Comitato direttivo, del Comita-to scientifico e della Redazione della Rivista.

Informazioni specifiche

b. Doveri dei Revisori. La valutazione dei Revisori non può tenere conto delle convinzioni personali, degli indirizzi teori-ci o delle appartenenze di scuola dell'Autore, ma verifica sol-tanto: a) l'originalità dell'impianto metodologico e dei risulta-ti; b) la correttezza degli stessi; c) l'ampiezza della conoscen-za critica della dottrina e della giurisprudenza; d) la coerenza interna formale (tra titolo, sommario ed abstract) e sostanzia-le (rispetto alla posizione teorica dell'Autore); e) la chiarezza dell'esposizione.

Gli esperti scelti come Revisori si impegnano: ad osserva-re scrupolosamente i criteri sopra indicati; b) a trattare il te-sto da valutare come confidenziale fino a che non sia pubblica-to, e a distruggere tutte le copie elettroniche e a stampa degli articoli ancora in bozza e le loro stesse relazioni una volta ri-cevuta la conferma della Redazione che esse sono state ricevu-te; c) a non rivelare ad altri quali scritti hanno giudicato; d) a non diffondere tali scritti nemmeno parzialmente e a non uti-lizzare informazioni e idee acquisite mediante la revisione per finalità scientifiche ovvero personali; e) ad assegnare per ognu-no dei cinque parametri prefissati un punteggio variabile dal minimo di 1 ad un massimo di 5, utilizzando l'apposita scheda trasmessa dalla Redazione, ad uno esclusivo e riservato della medesima. Un giudizio favorevole alla pubblicazione presup-pone che la qualità complessiva del contributo risulti "buona" (vale a dire un punteggio complessivo non inferiore a 15); f) a formulare un sintetico giudizio sul contributo, che sia espresso in modo chiaro e motivato con obiettività, prudenza e rispetto al fine, se necessario, di aiutare l'Autore a migliorare la quali-tà ed il valore scientifico del suo lavoro. Ogni dichiarazione, os-servazione o argomentazione riportata deve essere preferibil-mente accompagnata da una corrispondente citazione; g) a co-municare alla Direzione o alla Redazione della Rivista gli ele-menti emersi dopo la valutazione del contributo, nel caso in cui essi influiscano sull'esito del giudizio previamente espresso.

Il Revisore selezionato, che ritenga di non avere la compe-tenza necessaria per valutare il manoscritto assegnato ovvero che non sia in grado di adempiere al suo incarico nei tempi ri-chiesti, deve darne tempestiva comunicazione alla Direzione

Archivio giuridico Filippo Serafini

della Rivista, rinunciando a partecipare al processo di revisione o inoltrando una richiesta di proroga dei termini di esple-tamento dell'incarico.

Nel caso in cui i Revisori individuino la paternità del sag-gio e versino in una posizione di conflitto di interesse deri-vante da precedenti rapporti di concorrenza, di collaborazio-ne o altro tipo di collegamento con gli Autori e gli enti di ap-partenenza o comunque di afferenza, sono tenuti a rinunciare all'incarico, dandone tempestiva comunicazione alla Direzio-ne della Rivista.

I Revisori sono tenuti a comunicare alla Direzione della Ri-vista se sussista una somiglianza sostanziale o una sovrappo-sizione significativa tra il manoscritto da valutare e qualunque altro documento pubblicato di cui hanno conoscenza personale.

c. Doveri degli Autori. Con l'invio del contributo scienti-fico alla Direzione - Geraldina Boni (geraldina.boni@unibo. it) - o alla Redazione della Rivista - Daniela Bianchini Je-surum (daniela.bianchini@tiscali.it), Maria Teresa Capoz-za (m.capozza1@lumsa.it), Matteo Carnì (m.carni@lumsa.it), Francesco Galluzzo (francesco.galluzzo@unicatt.it), Manuel Ganarin (manuel.ganarin2@unibo.it), Alessia Legnani Anni-chini (alessia.legnani@unibo.it), Alessandro Perego (alessan-dro.perego@unipd.it) -, l'Autore esprime il consenso a sotto-porre il testo alla valutazione di docenti e esperti del settore scientifico disciplinare di riferimento o di settori affini, ester-ni agli organi della Rivista e scelti dalla Direzione nel rispetto della regola della revisione tra pari.

Gli Autori sono pregati di inviare i loro contributi via e-mail (scritti in formato .doc). Ogni lavoro dovrà essere corre-dato di: Nome, Cognome, Qualifica accademica, Indirizzo postale, Indirizzo e-mail, Numero di telefono (è gradito anche un numero di cellulare). Ogni articolo dovrà essere corredato di un titolo in lingua inglese e un riassunto in lingua italiana e inglese di non più di 200 parole specificando: scopo, metodolo-gia, risultati e conclusioni; e di almeno tre parole chiave in lingua italiana e inglese. Gli articoli, salvo casi eccezionali non potranno superare le 32 pagine (intendendosi già impagina-

Informazioni specifiche

te nel formato della rivista, ovvero circa 16 cartelle in forma-to A4 corrispondenti a 88.000 battute spazi e note inclusi). Gli Autori ed Editori di pubblicazioni giuridiche sono pregati di mandare un esemplare di ogni volume alla Redazione dell'Ar-chivio giuridico Filippo Serafini. Sarà gradito un foglio di ac-compagnamento con i dati bibliografici, classificazione, som-mario, etc. La Direzione della Rivista si riserva di recensire opere che, a suo insindacabile giudizio, risulteranno di mag-giore interesse.

L'Autore assicura che la sua opera sia pienamente origina-le e, qualora siano utilizzati il lavoro e/o parole di altri Auto-ri, che esse siano adeguatamente parafrasate o letteralmente riprodotte nel testo con precisi riferimenti in nota. L'Autore ha l'obbligo di citare le pubblicazioni rilevanti ai fini della re-dazione del contributo proposto. I manoscritti basati su ricer-ca originale devono essere corredati di un accurato resoconto delle indagini svolte, nonché di una adeguata argomentazione del risultato scientifico perseguito.

I manoscritti trasmessi alla Direzione della Rivista non de-vono essere stati pubblicati come materiale protetto da copyright in altre Riviste. I manoscritti in corso di valutazione non devono essere sottoposti ad altre Riviste ai fini della pubblica-zione. In caso contrario la Direzione può decidere di non accet-tare altri manoscritti presentati dal medesimo Autore per un periodo di tempo corrispondente a due anni, decorrente dalla data nella quale è comunicata all'Autore la sanzione commi-nata in conseguenza dell'infrazione commessa.

L'Autore (o gli Autori), una volta inviato il manoscritto, concorda che, in caso di pubblicazione, i diritti di sfruttamen-to economico, senza limiti di spazio e con le modalità e le tec-nologie attualmente esistenti e/o in futuro sviluppate, siano trasferiti alla Rivista e all'Editore.

Nel manoscritto l'Autore è tenuto a segnalare l'esistenza di conflitti economici o conflitti di interesse di diversa natu-ra che possano influenzare i risultati o l'interpretazione dello stesso manoscritto. Le fonti di supporto economico devono es-sere espressamente indicate.

Archivio giuridico Filippo Serafini

La paternità letteraria del manoscritto è limitata a colo-ro che: a) danno un contributo sostanziale all'ideazione, alla progettazione, all'analisi e all'interpretazione dello studio; b) redigono l'articolo o lo revisionano criticamente in relazione a importanti contenuti intellettuali; c) approvano la versione finale del contributo da pubblicare. Tutti coloro che hanno dato un contributo significativo devono essere elencati come co-Autori, secondo l'ordine stabilito da questi ultimi, indican-do l'apporto specifico di ciascuno allo studio e alla pubblicazio-ne. Uno o più co-Autori, se designati quali responsabili dell'in-tegrità del lavoro nel suo complesso, devono garantire che i nominativi di tutti i co-Autori siano inclusi nel manoscritto, che ciascuno di essi abbia preso visione ed approvato la versione definitiva dello stesso e concordi in merito alla sua pre-sentazione per la pubblicazione. Altre persone che hanno con-tribuito allo scritto, pur non essendo Autori, è opportuno siano menzionate tra i ringraziamenti.

Qualora un Autore riscontri inesattezze ovvero errori si-gnificativi, anche se commessi in buona fede, nel contributo pubblicato, ha il dovere di comunicarlo tempestivamente alla

Direzione della Rivista e all'Editore, cooperando al fine di cor-reggere o di ritrattare il contributo stesso.
d. Doveri di riservatezza. I Revisori e i componenti della

Direzione, del Comitato direttivo, del Comitato scientifico e della Redazione della Rivista si impegnano al rispetto scrupo-loso della riservatezza sul contenuto della scheda e del giudi-zio espresso, da osservare anche dopo la chiusura del processo di revisione e l'eventuale pubblicazione dello scritto.

I componenti della Direzione, del Comitato direttivo, del

Comitato scientifico e della Redazione della Rivista sono tenu-ti a non divulgare alcuna informazione sui manoscritti inviati ad alcun soggetto diverso da Autori, Editore, Revisori poten-ziali e incaricati della valutazione e membri degli organi della Rivista, a seconda dei casi.

Il materiale inedito contenuto nei manoscritti sottopo-sti alla Rivista e non ancora pubblicato deve essere trattato come un documento riservato. Esso non può essere usato dai

Informazioni specifiche

membri della Direzione, del Comitato direttivo, del Comitato scientifico e della Redazione della Rivista nonché dai Revisori potenziali e incaricati della valutazione per proprie ricerche, senza il consenso dell'Autore.

La documentazione relativa ai contributi sottoposti a va-lutazione ed in seguito pubblicati, agli scritti respinti a segui-to di decisione assunta dalla Direzione e a quelli respinti una volta concluso il processo di revisione è conservata negli archi-vi a cura della Redazione della Rivista, garantendo la riserva-tezza dei rispettivi contenuti.

7 – Elenco dei Revisori. I Revisori scelti dalla Direzione sono pubblicati ogni anno nel fascicolo n. 4 della Rivista e nel sito web dell'Editore.

8 – Indicizzazione della Rivista. La Rivista Archivio giuri-dico Filippo Serafini è indicizzata nelle seguenti banche dati: Articoli italiani di periodici accademici (AIDA), Catalogo ita-liano dei periodici (ACNP), DoGi Dottrina Giuridica, ESSPER Associazione periodici italiani di economia, scienze sociali e storia, Google Scholar, IBZ online International bibliography of periodical literature in the humanities and social sciences,

Journal Seek, Dialnet.
