# Supplementary material for: Peer Review in Law Journals
Source: Front Res Metr Anal. 2021 Dec 8;6:787768. doi: 10.3389/frma.2021.787768 (PMC8692876; doi:10.3389/frma.2021.787768)
Supplement: Supplementary file 3 [file DataSheet2.ZIP › DOCUMENT - 0392-1867.RTF]

Quaderni Fiorentini per la storia del pensiero giuridico moderno

CODICE ETICO

per il Direttore e il Consiglio di redazione, e per i revisori e gli autori degli articoli proposti per la stampa sui «Quaderni fiorentini per la storia del pensiero giuridico moderno»

I «Quaderni fiorentini per la storia del pensiero giuridico moderno» seguono i principi etici adottati dal COPE Committee on Publication Ethics.

DOVERI DEL DIRETTORE E DEL CONSIGLIO DI REDAZIONE

Il Direttore, coadiuvato dal Consiglio di redazione, decide quali articoli pubblicare sulla base di una revisione anonima fatta da esperti revisori (double-blind peer review).

Il Direttore e il Consiglio di redazione decidono esclusivamente in base al valore scientifico, alla rilevanza e all'originalità del contenuto dell'articolo senza discriminazioni di razza, genere, religione, origine etnica, cittadinanza, orientamento sessuale e orientamento politico degli autori.

Il Direttore e il Consiglio di redazione non possono dare informazioni sugli articoli proposti per la stampa a persone diverse dall'autore, dai revisori e dall'editore o stampatore, e s'impegnano a non usare per ricerche proprie i contenuti degli articoli non pubblicati, senza l'espresso consenso scritto dell'autore.

La Rivista si impegna a concludere il processo di valutazione entro tre mesi dal momento in cui il contributo è stato proposto. Il Direttore comunica senza ritardo le decisioni prese all'autore del contributo.


DOVERI DEI REVISORI

La revisione degli esperti contribuisce alla corretta valutazione degli articoli da parte del Direttore e del Consiglio di redazione e serve anche all'autore per migliorare il proprio contributo.

Il revisore che non si senta adeguato all'incarico richiesto o che non sia in grado di poter rispondere nei tempi indicati è tenuto a comunicarlo con la massima urgenza.

La revisione dev'essere svolta in modo corretto ed oggettivo. I revisori devono motivare le proprie valutazioni sull'articolo loro inviato in modo adeguato, indicando con precisione gli estremi bibliografici di opere importanti trascurate dall'autore dell'articolo e segnalando eventuali somiglianze o sovrapposizioni del testo esaminato con altre opere a lui note.

Le informazioni ottenute durante il procedimento di revisione devono essere ritenute confidenziali e non possono essere usate a vantaggio del revisore. I revisori non devono accettare in lettura articoli per i quali emerga un conflitto di interesse dovuto a precedenti specifici rapporti di collaborazione o concorrenza con l'autore o con la relativa istituzione di appartenenza.

DOVERI DEGLI AUTORI DEGLI ARTICOLI INVIATI AI «QUADERNI FIORENTINI»

Gli autori devono comporre un lavoro scientificamente originale in ogni sua parte e citare adeguatamente le fonti utilizzate per consentire ai lettori di comprendere il metodo e di ripercorrere la strada di ricerca seguita. Gli autori devono seguire le regole editoriali dei «Quaderni fiorentini».

Salvo espresso consenso del Direttore, non devono essere proposti contributi editi o in corso di pubblicazione altrove. Un articolo pubblicato sui «Quaderni fiorentini» potrà essere in seguito pubblicato altrove solo con il consenso del Direttore e a condizione che sia fatto espresso riferimento alla pubblicazione sui «Quaderni fiorentini».

La paternità dell'opera deve risultare con chiarezza: devono apparire come coautori tutti coloro che abbiano dato un contributo significativo alla realizzazione dell'articolo. Deve pure essere esplicitamente riconosciuto il contributo dato da altre persone in modo significativo ad alcune fase della ricerca. Nel caso di contributi a più mani deve risultare correttamente con chiarezza la parte di ciascun autore.
