# Supplementary material for: Peer Review in Law Journals
Source: Front Res Metr Anal. 2021 Dec 8;6:787768. doi: 10.3389/frma.2021.787768 (PMC8692876; doi:10.3389/frma.2021.787768)
Supplement: Supplementary file 3 [file DataSheet2.ZIP › DOCUMENT - 0392-7229.RTF]

About the Journal

Rivista Giuridica del Lavoro e della Previdenza Sociale website
Rivista Giuridica del Lavoro e della Previdenza Sociale – RGL was founded by Aurelio Becca and Ugo Natoli and is one of the oldest Italian labour law journals. The journal is a publication based on rigorous scientific criteria and was conceived as a peer reviewed publication. It is distributed “primarily” within the Italian scientific community, as witnessed by its variety of readers (university professors, lawyers, operators in the law field, judges, trade unionists, employers, experts in the industrial relations field, labour law consultants, public and private bodies) and by the number of subscribers (around 800). The journal gives ample space to the critical analysis of those innovations that occur in the areas of EU social policy law and International labour law. Special attention is also paid to EU Court of Justice and Italian Supreme Court’s case law
(on employment relations and social security).
ISSN: 0392-7229
Frequency: Quarterly
Language: Italian
First Year of Publication: 1949
