# Supplementary material for: Peer Review in Law Journals
Source: Front Res Metr Anal. 2021 Dec 8;6:787768. doi: 10.3389/frma.2021.787768 (PMC8692876; doi:10.3389/frma.2021.787768)
Supplement: Supplementary file 3 [file DataSheet2.ZIP › DOCUMENT - 0485-2435.RTF]

VALUTAZIONE SCIENTIFICA DEI CONTRIBUTI PUBBLICATI NELLE PARTI II E III E DELLE NOTE A SENTENZA


Lo scritto, dotato di un breve abstract in lingua inglese e italiana, viene pubblica-to in seguito ad una fase di valutazione preliminare in cui la Redazione valuta l'attinenza del tema trattato rispetto a quelli oggetto della Rivista, oltre alla presenza dei requisiti minimi di accettabilità.

In caso di esito positivo dell'esame preliminare, l'Autore riceve la comunicazione che lo scritto sarà valutato da referees scelti dall'elenco pubblicato nella Rivista ratione materiae. In casi eccezionali, la valutazione viene affidata a referees non presenti nell'elenco, ma dotati di idonea maturità scientifica e specifica esperien-za nella materia oggetto dello scritto da valutare.

Ciascun contributo viene sottoposto alla valutazione di due referees, italiani o stranieri, esperti della materia trattata, di cui almeno un professore ordinario, che comunicheranno esclusivamente con la Redazione della Rivista.

Su indicazione del Direttore della Rivista il contributo viene inviato dalla Reda-zione in forma anonima ai due referees, i quali sono vincolati (alla pari della Redazione e del Direttore della Rivista) a tenere segreto il loro operato. All'Au-tore non sono rivelati i nomi dei revisori neanche in caso di giudizio positivo (valutazione c.d. double blind peer review).

La valutazione dello scritto avviene sulla base dei seguenti criteri:

— rigorosità dell'impostazione metodologica;

— chiarezza espositiva;

— familiarità con la letteratura più rilevante sulla materia;

— adeguatezza e completezza della ricerca bibliografica;

— originalità della ricerca su cui si basa il lavoro;

— chiarezza delle tesi sostenute;

— apporto dello scritto rispetto al livello di conoscenza odierno delle tematiche trattate.

L'esito della valutazione può consistere nella:

— pubblicazione dell'articolo senza modifiche;

— pubblicazione con suggerimento di alcune modifiche migliorative, che sono sommariamente indicate dal revisore (l'adeguatezza delle modifiche apportate è valutata dal Direttore della Rivista senza necessità di ulteriore referaggio);

— pubblicazione con correzioni sostanziali in mancanza delle quali non è possi-bile pubblicare lo scritto;

— non pubblicazione.

In caso di valutazione divergente dei due referees, la decisione finale è presa dal Direttore della Rivista, sentito il Comitato scientifico.

VI	VALUTAZIONE SCIENTIFICA

In casi eccezionali un contributo potrà essere pubblicato senza previa valutazione dei referees, nel rispetto delle prescrizioni ANVUR. In tali casi, la circostanza sarà debitamente evidenziata.
