# Supplementary material for: Peer Review in Law Journals
Source: Front Res Metr Anal. 2021 Dec 8;6:787768. doi: 10.3389/frma.2021.787768 (PMC8692876; doi:10.3389/frma.2021.787768)
Supplement: Supplementary file 3 [file DataSheet2.ZIP › DOCUMENT - 0518-0872_1.RTF]

AFD Anuario de Filosofía del Derecho

ISSN: 0518-0872


Instrucciones para autores y autoras


I. Extensión y soporte material

Los originales de los artículos de las secciones «Monográfico», «Teoría y Filosofía del derecho» y «Debates» tendrán como extensión mínima 20 páginas y máxima 25 páginas DIN A4, a espacio y medio. Como criterio orientativo, se recomienda no exceder de los 50.000 caracteres, en general. El tipo de letra será «times 12» para el texto principal y en su caso «times 10» para las notas al pie. Tanto el texto principal como las notas al pie tendrán interlineado simple.

Todos los artículos deberán ir acompañados de un resumen y abstract de máximo de diez líneas en castellano y en inglés, así como de cinco o seis palabras clave o key words en castellano y en inglés. Asimismo, deberán incluir al comienzo el título en castellano y en inglés así como un sumario con los epígrafes en los que se estructura el texto tanto en castellano como en inglés.

Los originales de la sección «Crítica bibliográfica» no deberán superar las ocho páginas ni tener una extensión inferior a seis páginas DIN A4, a espacio y medio. Aproximadamente, no han de superar los 18.000 caracteres ni tener una extensión inferior a 15.000 caracteres.

Los artículos, recensiones y noticias han de enviarse por correo electrónico exclusivamente a la dirección del Anuario de Filosofía del Derecho: Anuario.Fil.Derecho@uv.es

En el documento es imprescindible incluir los datos del autor o autora así como el título del trabajo, entre los que deberá figurar tanto una dirección electrónica como una dirección postal de contacto al efecto de posibles envíos o comunicaciones. Deberá a su vez incluirse la relación del autor o autora con la institución pública o privada en la que desarrolla su cargo académico o actividad profesional (por ejemplo, en el caso de ser docente universitario, la universidad en la que ejerce su labor habitualmente).

1

II. Sistema de citas

Las notas a pie de página se confeccionarán según el criterio general que se incluye en el apartado III y se entenderá que constituyen las referencias bibliográficas del artículo, sin que sea necesario añadir una bibliografía final, evitando así reiteraciones. Una obra ya citada se mencionará con el nombre del autor o autora seguida de op. cit. y la página. Si el autor o autora tiene varias obras citadas en el artículo, se abreviará el título, seguido de cit. y la página. En caso de repetir de forma inmediata la misma referencia a autor, autora o documento, se indicará con ibidem y el número de página. Las abreviaciones de las locuciones de ídem, ibídem, infra, supra, apud o pássim, vide, cuando así lo exija la investigación serán preferentemente (id., ib., infr., supr., ap., páss. vid.). Las palabras extranjeras deben ir en cursiva. En las notas al pie y texto, la palabra página se abreviará p. o pp., según proceda; siguientes se expresará como ss.; para especificar conferir se utilizará su abreviación (cfr.), si no se trata de cita literal; la abreviatura de artículo será art. y solo se empleará cuando vaya entre paréntesis, en los demás casos deberá ir con todas las letras. Las siglas, abreviaturas y acrónimos irán en mayúsculas sin espacio (pegadas) y sin puntos, salvo la primera vez que se mencionen que deberá constar, entre paréntesis el significado de éstas.

Por último, en la primera cita de textos legales figurará el rango normativo, el número, fecha, nombre del fundamento legal y, entre paréntesis, el Boletín oficial en el que se publicó con referencia de número y fecha. Las citas iniciales de jurisprudencia y otras resoluciones contendrán: las siglas de la resolución y tribunal u órgano, sala y fecha, referencia de repertorio en que se publicó o consultó el original y nombre y apellidos del magistrado ponente.

III. Notas y referencias bibliográficas

En cualquier caso, al citar la obra completa, deberá ser presentada de la siguiente forma: Apellido/s, inicial del nombre, título, lugar de edición, nombre del editor, año de aparición, número de páginas (eventualmente colección). Se distinguirá entre libro, artículo de revista, capítulo del libro, etc. Ejemplos:

1.  Libro/monografía:

González Vicén, F.; El Positivismo en la Filosofía del Derecho contemporánea, Madrid, Instituto de Estudios Políticos, 1950, 96 pp.

2

2.  Artículo de revista:


González Vicén, F.; «La Filosofía del Derecho como concepto histórico», Anuario de Filosofía del Derecho, XIV, 1969, pp. 15-65.

3.  Trabajo o capítulo del libro:

González Vicén, F.; «Estudio preliminar a la traducción de Sobre la utilidad del estudio de la Jurisprudencia» de John Austin, en Estudios de Filosofía del Derecho, Santa Cruz de Tenerife, Facultad de Derecho, Universidad de La Laguna, 1979, pp. 17-33.

IV. Proceso de evaluación, admisión y revisión de manuscritos

Los trabajos enviados a la Redacción del Anuario de Filosofía del Derecho deben ser inéditos y serán evaluados mediante el sistema de «doble referee» manteniendo el anonimato en la revisión de los trabajos. Por ello, los autores o autoras deben eliminar los metadatos del mismo en el texto, para evitar que los evaluadores puedan identificar su autoría. El autor o autora que remita un trabajo de investigación ya publicado pero que haya sido objeto de una revisión y actualización sustantiva deberá acreditar fehacientemente la calidad de inédito de su trabajo conforme a las directrices de la Cátedra Unesco para que en última instancia el Consejo de Redacción o Asesor pueda considerar su posible evaluación. El evaluador o la evaluadora externa será decidido por el Consejo Asesor o el Consejo de Redacción del Anuario de Filosofía del Derecho, en su caso. La deliberación «por pares» de los trabajos presentados se realizará entre los meses de abril y junio y la aceptación o rechazo de los originales, así como las posibles sugerencias de modificación de los referees a los autores o autoras, serán comunicadas entre los meses de septiembre y octubre.

Tras el proceso de evaluación, los autores o autoras de los trabajos recibirán una comunicación motivada de la decisión editorial que incluya las razones para la aceptación, revisión o rechazo del artículo, así como, los correspondientes informes emitidos por los expertos o expertas independientes.

Los evaluadores o evaluadoras externos justificarán el dictamen emitido atendiendo a los siguientes criterios orientativos de evaluación de la Revista:

0.	Idoneidad de la temática propuesta.

0.	Originalidad o aportaciones novedosas.

0.	Bibliografía y aspectos metodológicos.


3

4.  Correcciones, recomendaciones y sugerencias de modificación.


Para ser publicados los trabajos, deberán obtener dos referees positivos de evaluadores o evaluadoras externos. Quienes evalúen podrán hacer constar los aspectos a corregir o sugerencias de modificación, así como comentarios adicionales que consideren oportunos para mejorar la calidad del trabajo. En el caso de que uno de los informes sea negativo o positivo con modificaciones, se estará a lo que decida el Consejo Asesor o el Consejo de Redacción, pudiendo ser enviado el trabajo a una tercera evaluación externa, que será vinculante. En cualquier caso, si la evaluación es positiva pero se indican recomendaciones de modificación sustantivas, el Consejo de Redacción y en su caso al Consejo Asesor, concederá un período de tiempo suficiente para que los autores o autoras obligatoriamente le remitan la versión definitiva del texto con un informe razonado y detallado de la revisión realizada de acuerdo a los referees. En tal supuesto, la decisión sobre la publicación definitiva del trabajo será sometida, de nuevo, al Consejo de Redacción, y en su caso, al Consejo Asesor de la revista.

Serán criterios excluyentes para la no admisión de los trabajos:


0.	Excederse en la extensión establecida según el tipo de colaboración.

0.	No utilizar el sistemas de citas propuesto en la manera indicada.

0.	No enviar el trabajo en el soporte requerido o sin las formalidades oportunas señaladas.

0.	Remitir el trabajo fuera de los plazos señalados en la convocatoria anual.

0.	Haber publicado un artículo en la misma sección del número inmediatamente anterior.

0.	Cualquier otra circunstancia que el Consejo de Redacción considere no adecuada a las instrucciones de la Revista.

Los autores o autoras de los trabajos aceptados recibirán a través de correo electrónico, una vez decidida la admisión definitiva del trabajo y hechas las oportunas modificaciones señaladas por los referees, una certificación escrita de la publicación de su trabajo en el número correspondiente.

Los autores o autoras de los trabajos rechazados recibirán por correo electrónico una comunicación escrita de la no publicación de su trabajo en el número correspondiente, junto a una copia del contenido de los referees negativos.

4

V. Compromiso ético para la publicación de artículos

El Anuario de Filosofía del Derecho se adhiere a las directrices del EASE (European Association of Science Editors) y del COPE (Committee on Publication Ethics) que pauta unas buenas prácticas para la gestión, edición, revisión y publicación de resultados científicos en revistas de diferentes áreas de conocimiento. Brevemente de acuerdo a lo allí establecido:

`.	Los autores y autoras se comprometen a remitir trabajos inéditos, que no hayan sido publicados anteriormente y que no se encuentren sometidos a evaluación por otras revistas mientras no se complete el proceso de evaluación por parte de esta revista. El envío de trabajos para su evaluación requiere omitir cualquier dato que pueda ser identificado por los revisores o revisoras respecto a su autoría y de igual modo implica aceptar las normas de publicación, revisión y evaluación de la revista que se detallan a tal fin en cada número de la revista

`.	Los revisores y revisoras de los trabajos asumen el compromiso de realizar una revisión crítica, honesta, constructiva sobre la calidad científica del texto dentro el ámbito de sus conocimientos y habilidades. Por ello, solo revisarán un trabajo si se sienten competentes con la temática a revisar y si no existen conflictos de interés.

`.	El Consejo de Redacción y los editores y editoras se comprometen a respetar la imparcialidad y mantener la confidencialidad de los trabajos enviados, sus autores y autoras y revisores y revisoras, de forma que el anonimato preserve la integridad de todo el proceso de evaluación. A tal fin garantizarán la selección de las personas más cualificadas y especialistas en la materia para emitir una apreciación crítica y experta del trabajo. Asímismo, en la medida de lo posible, evitarán todo tipo de conflictos de intereses así como cumplir estrictamente con los tiempos de evaluación, edición y publicación que exige la periodicidad de la revista.


5
