# Supplementary material for: Peer Review in Law Journals
Source: Front Res Metr Anal. 2021 Dec 8;6:787768. doi: 10.3389/frma.2021.787768 (PMC8692876; doi:10.3389/frma.2021.787768)
Supplement: Supplementary file 3 [file DataSheet2.ZIP › DOCUMENT - 0554-6397 (2).RTF]

Journal of Maritime & Transportation Sciences Instructions for authors

__________________________________________________________________________________


Instructions for authors


Journal of Maritime & Transportation Sciences (JMTS) is a periodical which publishes research results in the domain of maritime affairs, transportation and traffic sciences. It primarily publishes scientific papers. However, other paper categories are allowed, such as professional papers, reviews, news, forum sections and conference reports.

1 Categories of scientific papers

Original scientific paper represents the unpublished, replicable research, both fundamental or applicable, where new scientific contributions are reported.

Preliminary communication is a paper containing new scientific elements, such as evidences, results and conclusions, although without sufficient details needed for replication or validation.

Review article is a comprehensive scientific overview of (exclusively) the latest state made in a respective research area, contributing with a new and unique approach of collection, analyses, synthesis and presentation of results.

2 Other contributions

Professional paper contains useful information obtained during research on a specific topic. The contribution is reflected in the theoretical and/or practical applicability of published results.

Expert revision/review refers to evaluation of other's scientific work, technological progress in a certain area or, in general, any actual topic significant to scientific and professional community in areas covered by JMTS. Examples of review topics are doctoral thesis, scientific books, textbooks, projects, patents, program tools and applications, technological products etc.

Forum is a written form of an expert's personal opinion regarding a certain topic, often (but not exclusively) from a historical aspect. This section is subject to discussion among competent authors.

Categorization of scientific papers is made by the Editorial board based on positive reviews and reviewers' proposals. Once the paper is published, its category will be listed in the heading section and in the volume content.

3 The structure of the manuscript

The topic of the submitted manuscript should fit into the JMTS scope and covered areas. The submitted manuscript must, as much as possible, contain elements shown in the following table, and has to conform to the JMTS style.


__________________________________________________________________________________

1

Journal of Maritime & Transportation Sciences Instructions for authors

__________________________________________________________________________________

	Title of the paper;	
	Name of Author(s) (including e-mail and postal	
Heading section1	address);	
	Affiliation;	
	Abstract; and	
	Keywords.	
	Introduction;	
	Background;	
Research (main)	Methodology and resources;	
section	Research results;	
	Discussion; and	
	Conclusion	
	Supplements/ Annexes(if any);	
Back section	Acknowledgments(if any); and	
	References.	


The text should be as concise as possible, but not at the cost of clarity. The paper title has to be short, informative and comprehensive, justifying all paper elements. Within the abstract, a short paper overview is presented containing all necessary information – research topic, its significance, author's main contributions and derived conclusions. Keywords represent main terms on which the paper resides. The abstract is written in one paragraph up to approximately 150 words.

The central part of the paper presents the author/s' contribution. It is strongly recommended that it contains elements needed for clear and concise presentation of course of study, research, results and conclusions:

	Within the introductory chapter it is necessary to place the research and the topic in broad context and to emphasize the importance of the conducted research. This is the place where the main objective of the paper is stated, as well as derived conclusions and findings. It is desirable to indicate planned activities representing the continuation of the research;

	The background chapter refers to previous research in the area of the presented topic, including own contributions. Emphasis should be on the latest research and include the corresponding literature review. This chapter, either separately or as a part of the previous chapter, has to clearly and in detail introduce the reader to the main section;

	Methodology and resources have to be described distinctly, concisely and systematically in a way that the conducted research can be repeated. For usual scientific methods a short description is sufficient, while when employing new methods, particular emphasis should be given regarding their explanation;

	In the chapter on the results of the research it is necessary to provide a precise description of the results, and their objective interpretation and findings;


1 Author details are entered above the paper title. For additional explanation, refer to published issues.

__________________________________________________________________________________

2

Journal of Maritime & Transportation Sciences Instructions for authors

__________________________________________________________________________________

?	Authors are encouraged to discuss their findings from the aspect of previous research and proposed hypothesis. Conclusions, findings and their eventual impact (implications) have to be discussed in as wide a context as possible, with a clear definition of their limitations and possible future development possibilities;

?	The conclusion chapter summarizes the workflow of the paper, research results and findings. The reader should be familiar with any future planned activities and the possible continuation of the research.

The above elements do not have to be presented separately, but they can be placed in combination with other chapters (e.g. research results and discussion, introduction chapter and background etc.).

The back section contains acknowledgments (in case they exist), annexes which (if included) contain accompanying research documentation, and a mandatory list of references, listed alphabetically with numbers using Harvard style: bibliographic units, internet links, databases, software and programming tools etc. Throughout the text source numbers are used in square brackets and quoted before punctuation.

4 Referencing

0[]	(book, printed) Norris, A. (2001) ECDIS and Positioning. London, The Nautical Institute.

0[]	(e-book, electronic book) Sanz Subirana J., Juan Zornoza, J. M. & Hernandez-Pajares, M. (2013) GNSS Data Processing Book. Paris, European Space Agency. Available from: http://gage.upc.edu/gnss_book [Accessed 18th June 2017].

0[]	(book chapter) Hakkinen, J. M. & Posti, A. I. (2013) Overview of Maritime Accidents Involving Chemicals Worldwide and in the Baltic Sea. In: Weintrit, A. & Neumann, T. (eds.) Marine Navigation and Safety of Sea Transportation: Maritime Transport & Shipping. Boca Raton, CRC Press, pp. 15–26.

0[]	(scientific article, printed) Zhou, Y., Wang, W., Song, X. & Peng, Y. (2017) Container Shipping Network Optimisation Based on Steering Vector Search Pattern. Journal of Navigation. 70 (2), 395–410.

0[]	(scientific article, electronic) Lebedevas, S., Dailydka, S., Jastremskas, V. & Rapalis, P. (2017) Research of energy efficiency and reduction of environmental pollution in freight

rail transportation. Transport. 32 (3), 291–301. Available from: http://www.tandfonline.com/doi/pdf/10.3846/16484142.2016.1230888 [Accessed 20th October 2017].

0[]	(article in the publishing process) Vilke, S., Brčić, D. & Kos, S. (2017) Northern and

Southern European traffic flow land segment analysis as a part of the redirection justification. To be published in The International Journal on Marine Navigation and Safety of Sea Transportation.

0[]	(conference paper, published) Štern, A. & Bešter, J. (2012) Seamless Connectivity in a Networked Vehicle. In: Rijavec, R. & Anžek, M. (eds.) ISEP 2012: Linking people with ITS: Proceedings of the International Symposium on Electronics in Transport, ISEP 2012,


__________________________________________________________________________________

3

Journal of Maritime & Transportation Sciences Instructions for authors

__________________________________________________________________________________

26–27 March 2012, Ljubljana, Slovenia. Ljubljana: Electrotechnical Association of Slovenia. pp. 132–137.

0[]	(standard) British Standards Institution (2005) BS EN 1993-1-2:2005. Eurocode 3. Design of steel structures. General rules. Structural fire design. London, BSI.

0[]	(report) Thomas, M. (2011) Global Navigation Space Systems: Reliance and vulnerabilities. The Royal Academy of Engineering. Report Number: 63.

0[]	(doctoral thesis) Kotcharat, P. (2016) A forecasting model for container throughput: empirical research for Laem Chabang Port, Thailand. Ph. D. Malmo World Maritime University.

0[]	(magazine article) Williams, A. I., Shaw, G. & Ward, N. (2015) ACCSEAS: The Innovative North Sea e-Navigation Demonstration. Coordinates. 11 (7) pp. 11–16.

0[]	(map, chart) British Geological Survey. (1998) South London. 270, 1:50 000. London, British Geological Survey.

0[]	(website) Marine Electronics & Communications. (2017) Thenamaris improves fleet communications and data flows. Available from: http://www.marinemec.com/technology/fleet-management.htm [Accessed 16th October 2017].

0[]	(official documents) International Maritime Organization. (2012) MSC.347(91): Recommendation for the protection of the AIS VHF data link. Resolution. London: IMO.

0[]	(online database) National Geodetic Survey. (2017) Continuously Operating Reference Station (CORS) Navigation messages in RINEX format. [Online]. Available at: https://www.ngs.noaa.gov/CORS/standard1.shtml [Accessed 20th October 2017].

0[]	(software) RTKLIB. (2013) Tokyo. Takasu, T.2

5 Checklist

It is strongly recommended that prior to the final manuscript submission author/s consider the following checkpoints:
?	Originality of the paper, its significance and its contribution;

?	Style of writing, intelligibility and clarity;

?	Correct English language, grammar and appropriate vocabulary;

?	Explanatory but concise paper title and abstract;

?	Proper selection of keywords;

?	Consistent and thorough literature review;

?	Proper description of methodology, data and other resources used in the research;

?	Careful and comprehensive interpretation of results and their critical discussion;

?	Approach to presented topic, with relevant and exhaustive literature, emphasizing recent research in the area;
?	Adequate number and descriptive features of tabular and graphic presentations;

?	The structure and contents of the conclusion chapter;

?	General layout of the entire text.

2 For any unclassified reference type, authors are advised do contact editorial board.

__________________________________________________________________________________

4

Journal of Maritime & Transportation Sciences Instructions for authors

__________________________________________________________________________________

6 Remaining guidelines

Acceptance of the paper for publishing is based on the review process from at least two reviewers. During this process the authenticity of the manuscript is determined as well and its originality is validated. The submission of the paper implies that it was not sent elsewhere for publishing consideration.

Manuscripts have to be submitted in Microsoft Word format, using Times New Roman font, size 12. Authors are kindly requested not to format the manuscript, both graphically and textually, except the general guidelines provided here together with the following:
?	The text should be single spaced, without spaces between rows and paragraphs;

?	Numbering of chapter and subchapter titles should be entered manually;

?	Abbreviations, if used, should be explained when introduced;

?	Footnotes are used as little as possible, preferably not at all;

?	The SI measuring units system is allowed for use.

Tabular and graphical presentations should contain ordinal number, title and source (in case they are retrieved or adapted). The JMTS is printed in black and white, while online is published in color. Authors should consider this feature and customize all figures for the sake of clarity and understanding. For the same reason, within the presentations it is necessary to adjust the size of the text. Desirable image formats are TIF, JPG and PNG, with at least 300 dpi resolution.

Mathematical expressions should be numbered and written using appropriate MW Equation Editor.

The final size of the paper and its elements should not exceed 30 000 characters, being approximately 16 typewritten pages.

The JMTS official language is English. The paper title, abstract and keywords should be written both in English and in the corresponding author's native language. It is recommended that non-English authors prepare their manuscript and have it checked by a language expert or native English speaker.

The Editorial board reserves the right to adapt the manuscript to propositions of the Journal, as well as to comply with English and Croatian language standards.

Manuscript submissions are sent via email to the Association for Promotion and Development of Maritime Industries (udruga.pomorstvo@gmail.com). The same address should be used for any additional questions regarding submission, publishing of manuscripts or any other questionable detail.


__________________________________________________________________________________

5
