# Supplementary material for: Peer Review in Law Journals
Source: Front Res Metr Anal. 2021 Dec 8;6:787768. doi: 10.3389/frma.2021.787768 (PMC8692876; doi:10.3389/frma.2021.787768)
Supplement: Supplementary file 3 [file DataSheet2.ZIP › DOCUMENT - 0554-6397.RTF]

 
		

ARTICLE REVIEW

Reviewer
Article 
author
Article title

1.Article offers a new, original and important observations of this scientific area
YesNo
2.

Article reflects research method with appropriate and correct bibliographic units of literature of other authors and papers 
YesNo
3.
The terminology used in the article is in accordance with the standards and it is used consistently 
YesNo
4.The conclusion is methodically and logically derived from the content
YesNo
5.
Summary presents the essential article concepts, problems and results 
YesNo


		
			  6.       Article rating:           a) recommended publishing           b) require further work           c) not for publication7. In the article methodology is presented and applied:a) expressly and properlyb)implicitly and satisfactory c) unclear and poorly8. Article goal is achieved:a) completely
b) partially9. Suggestions for corrections and article improvements:


Proposal for article category:
`)	Professional paper
`)	Conference paper
`)	Review article
`)	Preliminary communication
`)	Original scientific paper


In


date

Reviewer signature


	
