# Supplementary material for: Peer Review in Law Journals
Source: Front Res Metr Anal. 2021 Dec 8;6:787768. doi: 10.3389/frma.2021.787768 (PMC8692876; doi:10.3389/frma.2021.787768)
Supplement: Supplementary file 3 [file DataSheet2.ZIP › DOCUMENT - 0584-9063.RTF]

INSTRUCTIONS FOR CONTRIBUTORS


Zbornik radova Pravnog fakulteta u Splitu (Collected Papers of the Faculty of Law in Split) is a journal for legal and general social issues that fosters thematic and disciplinary openness. It publishes works in different social and humanistic disciplines (sociology, political science, history, economics, etc.), and also considers works that are interdisciplinary. The journal is published four times a year.

The Editorial board only accepts manuscripts that are written in Croatian or English, which have not been previously published. Manuscripts should be submitted in written or in electronic form online via the Open Journal System: http://www.pravst.unist.hr/zbornik.php

By submitting your manuscript to this journal, you accept that your manuscript may be screened for plagiarism against previously published works. To find out more about Similarity Check visit http://www.crossref.org/similaritycheck.html.

The authors are not charged by the journal for the cost of receiving, reviewing and publishing papers. The journal is funded by the Ministry of Science and Education of the Republic of Croatia, by subscription to the print edition and funds from the publisher.

The journal supports an open approach, which means that all of its contents are freely available without charging users or their institutions. Users are allowed to read, upload, copy, distribute, print out, search, and share links to the full text of the articles or use them in any other legal way without requesting explicit permission from the author or publisher. Or. "All content is made freely available for non-commercial purposes, users are allowed to copy and redistribute the material, transform, and build upon the material as long as they cite the source.''

Review procedure

All the papers that pass the first editorial control are subject to the so-called double-blind review process which does not reveal the identity of the authors or reviewers. Therefore, the authors are asked to remove from the text and list of references, as well as from the properties of the electronic document, all the information that can reveal their identity (name, project information). After receiving the reviews, the authors, in addition to the new version of the manuscript, submit to the Editorial board a cover letter stating which reviewers' proposals were accepted and in what way, and which were not (with explanation and argumentation). Authors can write an appeal letter regarding the review process or the decision on the manuscript to the Editor in chief. In case of repeated appeal, the Editor decides on the basis of a discussion at a meeting of the Editorial board.

The Editorial board reserves the right to revise the manuscript to the journal's standards in terms of editing and language.

Copyright notice


Copyright for articles published in this journal is retained by the authors, with first publication rights granted to the journal (this applies to both print and electronic issue). Papers published in the journal are licensed under the Creative Commons license BY-NC-ND, which means that their content can be distributed with attribution for non-commercial purposes without modification.

Ethics

Research should be in accordance with the applicable ethical rules and codes of conduct of research, wherein the description of the research procedure should contain all information relevant to the evaluation of the ethics of conducting the research. The Editorial board may request confirmation from the authors that the research was approved by the ethics committee of the relevant institution.

In accordance with the instructions for authors of the European Association of Science Editors (EASE) 2016 (http://ease.org.uk/publications/author-guidelines), the journal recommends that the list of authors include a list of all people who have significantly contributed to the planning of the study, data collection and interpretation of the results and have also written or edited the manuscript and approved its final version accepting responsibility for all aspects of the paper. Any person who meets the first criterion should be allowed to participate in the writing of the manuscript and approval of its final version (ICMJE 2015).

Reviewers are required to treat each received manuscript as confidential. Authors and reviewers are required to reveal potential conflicts of interest related to the article which is sent or reviewed, or, in case of conflict of interest, to exclude themselves from the review process. The same is true for editors and editorial board members, who should be excluded from the editorial process if they are in conflict of interest (cooperation or other links with the authors).

In case of suspicion of unethical scientific conduct (plagiarism, self-plagiarism, manipulation of data, changes in authorship etc.) the Editorial board will act in accordance with the guidelines of the international association - "Committee on Publication Ethics" (COPE), the contents of which can be seen here: http: // public. mzos.hr/Default.aspx?art=7966&sec= 2142 (http://publicationethics.org/ resources/flowcharts).

Contributors are requested to consider the following:

Collected Papers of Faculty Law in Split is a journal which publishes papers primarily from the fields of legal and social sciences. The Editorial Board accepts for consideration exclusively previously unpublished papers. Authors retain the copyright on the papers published in the journal, but grant the right of the first publication to the journal. The paper accepted for publication or already published in Collected Papers may be published by the author(s) in other publications only with the permission of the Editorial Board, and in such a case only with proper notice of its publication in Collected Papers.

Manuscripts should be submitted on a diskette or CD-Rom in Microsoft Word or RTF format and two double-spaced printed copies. The title of the paper, the author's name, surname


and academic or professional status should be indicated, as well as the name and address of the author's place of work or home address and e-mail address.

Manuscripts are anonymously reviewed twice and categorised as follows:

0()	Original scientific paper – the paper which is characterised by originality of conclusions, or which presents previously unpublished original results of scientific research;

0()	Review article - the article that contains detailed and comprehensive critical review of a certain problem area, but with no significant originality of results;

0()	Preliminary communication – the paper which presents primary findings of research in progress, which due to current interest require immediate publication, but without the level of deep and thorough study required for the scientific paper, and

0()	Professional paper – the paper which contains information and experience relevant for a certain profession, but without scientific characteristics.

Manuscripts as a rule should not exceed thirty-two double-spaced pages. They should include an introductory synopsis of not more than ten lines, a list of up to five key words and a summary of not more than one double-spaced page. Book reviews, commentaries and evaluative reviews are not subject to review and should generally be no longer than eight double-spaced pages.

The Editorial Board reserves the right to edit the paper according to the general rules of editing publications and the standard of the Croatian language. Manuscripts will not be returned. Notes should supply full data on references since they are not published separately. References should include the name of the author, the title, the publisher, place and date of publication and the number of the page(s) to which they refer.
