# Supplementary material for: Peer Review in Law Journals
Source: Front Res Metr Anal. 2021 Dec 8;6:787768. doi: 10.3389/frma.2021.787768 (PMC8692876; doi:10.3389/frma.2021.787768)
Supplement: Supplementary file 3 [file DataSheet2.ZIP › DOCUMENT - 1120-9607.RTF]

Dichiarazione sull’etica e sulle pratiche scorrette nella pubblicazione dei lavori scientifici

La presente dichiarazione è basata sulle COPE’s Best Practice Guidelines for Journal Editors.

Decisione di pubblicazione e doveri dell’editore 
La pubblicazione dei risultati della ricerca scientifica è un processo complesso che impone a tutti i soggetti coinvolti (editore, membri dei board, revisori, autori) rigore e accuratezza.
L’editore seleziona le riviste che pubblica sulla base della propria politica editoriale e dichiara eventuali fonti di finanziamento; promuove presso le direzioni l'adozione delle migliori prassi internazionali e ne verifica l’applicazione; richiede pubblicazioni originali - redatte nel rispetto del diritto d’autore e non sottoposte contemporaneamente a valutazione presso altre riviste - che seguano alti standard nella cura editoriale dei testi.
Inoltre, l’editore promuove e controlla l’utilizzo della peer review come metodo di selezione degli articoli, favorisce l’indipendenza della ricerca, condanna la violazione del copyright e il plagio, richiede e promuove contributi originali basati su dati rigorosi, impegnandosi a pubblicare le correzioni di eventuali errori negli articoli pubblicati.
Infine, l’editore riflette con l'Associazione italiana editori sull'evoluzione dell'editoria accademica, dialoga con ministero dell’Università e della Ricerca, Anvur, Cun e Crui, si confronta con istituzioni, ricercatori e bibliotecari sui temi legati alla diffusione e valorizzazione della ricerca, anche nelle forme dell'Open access.
Doveri degli organi editoriali 
La direzione della rivista garantisce la correttezza dei sistemi utilizzati per valutare, accettare o respingere gli articoli sottoposti dagli autori e in particolare vigila sul processo di peer review e sull’anonimato dei revisori rispetto allo specifico articolo in esame; evita inoltre ogni conflitto di interesse, discriminazione per genere, orientamento sessuale o religioso, convinzioni politiche, provenienza geografica. 
La direzione della rivista avverte l’editore in caso riscontri negli articoli in esame episodi di plagio e violazioni del diritto d’autore. 
Inoltre, se vengono accertati gravi errori, situazioni di conflitto di interessi o casi di plagio in un articolo pubblicato, informa l’editore e, se appropriato, appronta un erratum o invita a ritirare l’articolo.
Il direttore responsabile esercita sul contenuto del periodico da lui diretto il controllo necessario a impedire che nella pubblicazione siano commessi reati. 
Il comitato di redazione è guidato dalle politiche editoriali della rivista e tenuto al rispetto delle disposizioni di legge vigenti in materia di diffamazione, violazione del copyright e plagio.
Il comitato di redazione è tenuto a non divulgare alcuna informazione sui manoscritti inviati se non agli autori stessi o a chi sia stato previamente autorizzato.
Il materiale inedito contenuto nei manoscritti sottoposti alla rivista non può essere usato dai membri del comitato di redazione per proprie ricerche senza il consenso scritto dell’autore.
Doveri dei revisori
Il revisore assiste la direzione della rivista nel processo di valutazione degli articoli rispettando i tempi previsti.
La revisione deve essere condotta obiettivamente e con argomentazioni chiare e documentate.
Il revisore non deve accettare manoscritti nei quali abbia conflitti di interesse derivanti da rapporti di concorrenza, collaborazione, o altro tipo di collegamento con autori, aziende o enti connessi in qualche modo con l’oggetto del manoscritto.
Il revisore ha il compito di identificare la presenza di materiale bibliografico rilevante per il lavoro da valutare ma non citato, e indicare agli autori eventuali miglioramenti utili alla pubblicazione dei contenuti. 
Gli articoli presi in esame per la valutazione devono essere trattati come documenti riservati. Essi non devono essere mostrati o discussi con chiunque non sia previamente autorizzato dal comitato di redazione.
Doveri degli autori 
L’autore garantisce che l’articolo sottoposto a valutazione sia originale, inedito e non sottoposto contemporaneamente ad altre riviste, e deve accettare le modalità di selezione degli articoli e in particolare il processo di peer review.
Qualora l’articolo sia accettato, l’autore riconosce all’editore il diritto alla pubblicazione, autorizzando tutte le modalità di utilizzo previste nella manleva, da firmare prima della pubblicazione.
L’autore si impegna a dichiarare, contestualmente all’invio del contributo per la valutazione, ogni possibile conflitto di interesse che possa aver influenzato il contenuto del manoscritto. Tutte le fonti di sostegno finanziario al progetto devono essere indicate.
L’autore ha l’obbligo di citare correttamente le fonti dei contenuti riportati e ottenere le autorizzazioni necessarie alla pubblicazione di immagini, tabelle o altri contenuti già pubblicati secondo quanto stabilisce la legge sul diritto d’autore. Dichiarazioni fraudolente o volontariamente inesatte costituiscono un comportamento non etico e sono inaccettabili.
L’autore garantisce la veridicità dei dati presentati nell’elaborato e l’obiettività delle proprie interpretazioni. I dati relativi devono essere riportati con esattezza e in modo dettagliato per permettere ad altri di replicare l’indagine.
Devono essere inclusi nel manoscritto, e figurare come autori, tutti quelli che hanno effettivamente partecipato alla stesura del testo, visto e approvato la versione definitiva dello stesso e sono d’accordo sulla pubblicazione.
Se vi sono altri soggetti che hanno fornito contributi sostanziali relativi a parti rilevanti dell’articolo, devono essere riconosciuti ed elencati come contributori.
Qualora l’autore riscontri errori significativi o inesattezze nel manoscritto pubblicato ha il dovere di comunicarlo tempestivamente al comitato di redazione e fornire gli opportuni errata corrige.
