# Supplementary material for: Peer Review in Law Journals
Source: Front Res Metr Anal. 2021 Dec 8;6:787768. doi: 10.3389/frma.2021.787768 (PMC8692876; doi:10.3389/frma.2021.787768)
Supplement: Supplementary file 3 [file DataSheet2.ZIP › DOCUMENT - 1126-7917.RTF]

Istituzioni del Federalismo istituzionidelfederalismo@regione.emilia-romagna.it

Editorial Board:

Gianluca Gardini (editor-in-chief), administrative law, University of Ferrara Brunetta Baldi, political science, Alma Mater Studiorum-University of Bologna Francesco Bilancia, constitutional law, G. D'Annunzio University, Chieti-Pescara

Stefano Civitarese Matteucci, administrative law, G. D'Annunzio University, Chieti-Pescara Justin Frosini, comparative public law, Bocconi University, Milan

Alfredo Galán Galán, administrative law, University of Barcelona
Giancarlo Gasperoni, sociology, Alma Mater Studiorum-University of Bologna Peter Leyland, public law, London Metropolitan University
Marco Magri, administrative law, University of Ferrara
Andrea Morrone, constitutional law, Alma Mater Studiorum-University of Bologna Alessandra Pioggia, administrative law, University of Perugia

Giuseppe Piperata, administrative law, IUAV University, Venice
Claudia Tubertini, administrative law, Alma Mater Studiorum-University of Bologna


Editorial Guidelines

"Istituzioni del Federalismo" (IdF) publishes scientific contributions on the topic of territorial autonomy, reflecting different disciplinary approaches, with a preferential focus on legal and political issues. IdF only accepts original, unpublished texts. On the basis of exceptional, specific agreements with the author, the journal will accept works already published or submitted for publication elsewhere, expressly mentioning this circumstance in a footnote. IdF accepts texts written in Italian, English, Spanish, or French, and such texts will be published in their original language. Once accepted, the work is covered by copyright of the journal and may not be reproduced without permission.

All contributions submitted for publication are subject to an anonymous, double-blind peer review process; the editorial board is responsible for the final decision concerning publication. The main criteria for acceptance are: methodological rigor and consistency, text structure and framework; scientific originality and relevance; validity of the reasoning supporting the central thesis; accuracy / completeness of sources and bibliography.

IdF is divided into several sections: "Essays and articles", "Notes and comments", "Regional observatory", "Reviews".

Essays and articles. The main part of IdF is dedicated to publishing essays and articles: their length may vary between 20,000 and 70,000 characters, including footnotes and bibliography; all submissions must be accompanied by a brief abstract.

Notes and comments. Contributions published in this section are shorter comments to legal sources, case notes, etc., and usually do not exceed 40,000 characters in length.

Regional observatory. This section is devoted to reviews of law, regional legislation, analysis of regional good practices, news from the European Union, and other information.
Reviews. This section hosts reviews of published monographs and bibliographical reports.

Please note that bibliographical references should be cited, in complete format, in footnotes (and not at the end of the article or in the main text).
Citations should be made strictly in accordance with the following information:

Monographs: author (first name initial and surname in capital letters), title (in italics), place of publication, publisher, year of publication.
Journal article: author and title (such as for books), journal name (in italics), issue number, initial and final page numbers.
Chapter in edited book: author and title (as for monographs), editor (first name initial and surname in capital letters), book title (in italics), place of publication, publisher, initial and final page numbers.
