# Supplementary material for: Peer Review in Law Journals
Source: Front Res Metr Anal. 2021 Dec 8;6:787768. doi: 10.3389/frma.2021.787768 (PMC8692876; doi:10.3389/frma.2021.787768)
Supplement: Supplementary file 3 [file DataSheet2.ZIP › DOCUMENT - 1128-7772_2.RTF]

Peer Review Policy

The Journal only publishes original research articles, review articles, and book reviews. To be accepted for publication, submitted papers must not have been previously published elsewhere, entirely or in part, nor should they be currently under review for publica-

tion by another journal. The Chief Editors of the Journal will decide whether submissions contain well-conducted research with conclusions supported by the sources discussed in the paper. All papers must present the results of original research and make an incremental or novel addition to the existing literature.

The Journal aims to maintain the highest standards of peer review while processing submissions in a timely and efficient manner.
	The Journal adopts a double-blind peer reviewing policy, in which the identity of both reviewer and author is concealed from both parties. All text of the submitted papers, including footnotes and acknowledgements, must therefore be worded appropriately in order to ensure anonymity. In addition, the author's name, institutional affiliation and other contact information are removed before the manuscript is sent to the reviewers.
	All publication decisions are made by the Chief Editors on the basis of the reviews provided.

	Members of the Editorial Board assist and advise the Chief Editors in evaluating specific submissions.

Peer review of new submissions:

Articles submitted directly to the Journal will be fully peer reviewed by at least two appropriately qualified experts in the field selected by one of the Chief Editors. The Chief Editors will then decide whether to accept, reject or request revisions based on the reviews and comments received. Authors will be informed about the decision by e-mail.

Further reviews of refereed papers:

Refereed papers will be assessed by the Chief Editors of the Journal, who will decide, on the basis of the reviews, whether to accept or reject them, or request revisions. The Editors may also decide to seek additional reviews if deemed necessary. Authors will be advised when this is the case.
