# Supplementary material for: Peer Review in Law Journals
Source: Front Res Metr Anal. 2021 Dec 8;6:787768. doi: 10.3389/frma.2021.787768 (PMC8692876; doi:10.3389/frma.2021.787768)
Supplement: Supplementary file 3 [file DataSheet2.ZIP › DOCUMENT - 1131-8635_1.RTF]

Cuadernos de Relaciones Laborales
Submission Preparation Checklist
As part of the submission process, authors are required to check off their submission's compliance with all of the following items, and submissions may be returned to authors that do not adhere to these guidelines.
0.	The article has never been published before, nor has it been submitted to another journal (or an explanation has been provided in Comments to the Editor).
0.	The format of the file sent is OpenOffice, Microsoft Word or RTF.
0.	Web addresses have been included for references whenever possible.
0.	DOI references have been included when available.
0.	The text meets the reference and style requirements described in the Author Guidelines, which may be found in About the Journal.
0.	If you are submitting something for a peer-reviewed section of the journal, please make sure you have followed the instructions in Ensuring a Blind Review.
Author Guidelines
1. The journal Cuadernos de Relaciones Laborales, (known also by the abbreviated title CRL) ISSN1131-8635, edited by the Escuela de Relaciones Laborales and published by the Publications Service of Universidad Complutense de Madrid, is a peer reviewed journal which comes out twice yearly and was founded in 1992. It includes articles, technical notes and accounts and reviews, accepts original work written mainly in Spanish and, on an exceptional basis  sometimes, in English or in French.
2. Presentation of Originals
The originals sent to the journal to be published should comply with the following rules:
?	Articles must be submitted via e-mail in Word, font size 11, Times New Roman font and should be at least 7,000 and not exceed 10,000 words. Technical notes must span a maximum of 4,000 words and must include notes or comments on research, events, or publications related to the journal’s theme. Book reviews should be no longer than 2,000 words and should be dedicated to analysing books related to the journal’s themes.
?	All contributions must meet the following format requirements: size of page 17 x 24 cm, top and bottom margins 2.6 cm., left-hand margin 2.1 cm., and right-hand margin 1.8 cm.; font Times New Roman 11 and line spacing exactly to 12; the first line of every paragraph should be indented by 0.5 cm. The heading titles must be written bold and capital letters, the first level sub-headings must be in roman and bold type, and the second level sub-headings in cursive. Quotations longer than 4 lines should be indented from the text by 1 cm and appear in Times New Roman 10.  Line spacing should be exactly to 11 and inverted commas should be used. Footnotes must be in Times New Roman 10 and line spacing exactly to 11.
?	The article’s cover page should include the following:
?	The title of the article in Spanish and in English.
?	Author(s): First and last name(s) (the latter in block capital letters), university or institution that the author(s) belong(s) to (full name), department, and e-mail address.
?	Abstract: a text in Spanish of between 80 and 150 words.
?	Standardised article reference: (Example: FERNÁNDEZ GARRIDO, J. 2002. “El entorno de la violencia psicológica en el trabajo”. Cuadernos de Relaciones Laborales. Vol. 20 Num. 2: 101-132).
?	Key words: each article should have between three and five key words.
?	English translation of the abstract, standardised article reference and key words.
?	Summary (only in Spanish): numbered headings in the article, including conclusions and bibliographic references.
?	The article should include bibliographic references, appearing in alphabetical order, as per the following example:
Journal article
Aragón Medina, J. (2002). La estrategia europea de empleo. Cuadernos de Relaciones Laborales. Vol. 20 Núm. 1, 15-56.
Books and Chapters from Books
Alonso Benito, L.E. (1999). Trabajo y ciudadanía. Madrid: Trotta.
Bilbao Sentis, A. (1999) Modelo socioeconómico y organización de las relaciones laborales. En C. A. Castillo Mendoza, (Coor.), Economía, organización y trabajo (pp 229-253). Madrid: Pirámide.
The DOI number of the articles or books, when applicable, should be included in the list of references.
e.g: Surname, Name: Title of the Article, Journal, volume, year, DOI: xxxx; http://dx.doi.org/xxxx
`.	References to the bibliography should be made inside the text itself, between brackets, citing the author’s surname, followed by the year and edition and, when applicable, a colon followed by the page number(s) mentioned. The bibliographical references must be mentioned in the text.
`.	When quotations are no longer than two lines, they must appear in the text itself in quotation marks. When they are longer than two lines, they must appear without quotation marks in a separate, indented paragraph.
`.	Because the texts must be handled with layout programs before they are sent to press, it is preferable for format codes to be kept to a minimum. For the same reason, any notes must be presented as end notes and not as footnotes.
`.	Because the journal is not published in colour, authors should take special care and have special regard for this when including tables and graphs.
Any article not meeting these format, presentation, and content requirements should be returned to the author.
3. Publishing Process
Each issue of the journal normally includes a series of articles focusing on a single topic as well as other articles addressing different topics. In addition, the journal always includes specific sections, notes, and reviews.
Originals should be sent to the editorial board secretary, at the following e-mail address: revista.crl@ucm.es
The articles shall be anonymously evaluated by several members of the Scientific Advisory Board in two stages before their publication. The first assessment determines whether the article is in line with journal’s editorial policy. The second assessment involves a peer review by various members the Advisory Council. The Editorial Council shall then issue a definitive decision using a double blind system.
No changes in the articles shall be accepted once the first proofs have been corrected.
As long as the magazines continues to be published both in print and digitally, the author(s) shall receive off prints of their article(s) in addition to two copies of the magazine.
Each issue of the journal is issued in both print and electronic versions, the latter available at: http://revistas.ucm.es/index.php/CRLA
Acceptance of a work for publication results in transfer of copyrights, in any medium or support, to the publisher of the journal.
Copyright Notice
In order to support the global exchange of knowledge, the journal Cuadernos de Relaciones Laborales is allowing unrestricted access to its content as from its publication in this electronic edition, and as such it is an open-access journal. The originals published in this journal are the property of the Complutense University of Madrid and any reproduction thereof in full or in part must cite the source. All content is distributed under a Creative Commons Attribution 4.0 use and distribution licence (CC BY 4.0). This circumstance must be expressly stated in these terms where necessary. You can view the summary and the complete legal text of the licence.
 
 
Privacy Statement
Basic information on treatment data protection: Publications Service
Controller	Vicerrectorado Cultura y Deporte y Extensión Universitaria, +info	
Purpose	Dissemination and sale of UCM’s publishing/ billing and shipping of publications +info	
Legitimación	Mission in public interest; execution of a contract +info	
Recipients	No data will be transferred to third parties, except legal obligation +info	
Rights	Access, rectify and erasure data, as well as other rights, explained in the additional information +info	
Additional information	You can find more detailed information on our website:
https://www.ucm.es/file/info-adic-servicio-publicaciones-eng	
