# Supplementary material for: Peer Review in Law Journals
Source: Front Res Metr Anal. 2021 Dec 8;6:787768. doi: 10.3389/frma.2021.787768 (PMC8692876; doi:10.3389/frma.2021.787768)
Supplement: Supplementary file 3 [file DataSheet2.ZIP › DOCUMENT - 1133-0740_1.RTF]

ABOUT THIS JOURNAL
   	
SCOPE
The Anuario de Psicología Jurídica - Annual Review of Legal Psychology is a multi-discipline scientific and professional journal, which accepts original, unpublished articles in Spanish and English. These articles must be related with topics which fall within the area of legal psychology, and both the legal and the psychological perspectives. Articles must be empirical contributions about basic or applied research within legal psychology. Activities described in the articles published must follow the professional code of ethics and responsibilities.
Anuario de Psicología Jurídica - Annual Review of Legal Psychology is published mainly in Spanish although original contributions in English are also accepted.Potential authors should also be aware that although the Annual Review of Legal Psychology makes use of an double-blind external revision process (peer review), the director of the journal may make a final decision to reject a manuscript, without sending it for revision, in cases the content of the paper does not fit the field of the journal, or it has not been prepared in accordance with the rules given to potential contributors. Authors should not submit manuscripts simultaneously to multiple journals.
All opinions and comments expressed by the authors of articles are their own responsibility, and do not necessarily reflect those of the journal or its publishers. 
Authors are not charged any fees or charges for manuscript processing and/or publishing in the journal.
 
INTERNATIONAL STANDARD SERIAL NUMBER AND SPANISH LEGAL DEPOSIT OF PRINTER
ISSN: 1133-0740
ISSN ELECTRÓNICO: 2174-0542
DEPÓSITO LEGAL: M.39.689-1991
JOURNAL METRICS
0.	Web of Science (Clarivate) Impact Factor 2020: 1.545 (Q2)
           Journal Citation Reports 2020 - Law: Ranking 64/151
           Journal Citation Reports 2020 - Psychology Multidisciplinary: Ranking 92/140
0.	2020 Scopus Cite Score: 2.0
Social Sciences (Law): Ranking 138 de 722 (Q1)
Medicine (Pathology and Forensic Medicine): Ranking 107 de 191 (Q3)
Psychology (Applied Psychology): Ranking 133 de 227 (Q3)
0.	2020 Scimago Journal Rank: 0.440 (Q1)
INDEXED
Anuario de Psicología Jurídica is indexed in the following indexes and bibliographics:
`.	Web of Science - Social Sciences Citation Index`.	 (Clarivate)
`.	    Journal Citation Report 2020 Law: Ranking 64/151 (Q2)
`.	Scopus (Base de datos bibliográfica internacional Elsevier)    
`.	
`.	Scopus CiteScore: 2.0
`.	      Law 2020: Ranking 138/722 (Q1)
`.	Academic Search Complete (EBSCO)
`.	Academic Search Ultimate (EBSCO)
`.	Business Continuity & Disaster Recovery Reference Center (EBSCO)
`.	CARHUS Plus+ 2019
`.	Crossref
`.	Dialnet (plataforma de recursos y servicios documentales de la Universidad e la Rioja)
`.	DICE & RESH (CINDOC, CSIC)
`.	DOAJ (Directory of Open Access Journals)
`.	ERIH PLUS (European Reference Index for the Humanities and Social Sciences)
`.	Google Académico (Google)
`.	ISOC (CINDOC, CSIC)
`.	Latindex (América Latina, el Caribe, España y Portugal)
`.	MIAR (University of Barcelona, Spain)
`.	PSICODOC (Colegio Oficial de la Psicología de Madrid)
`.	Psyke (Universidad Complutense de Madrid)
`.	Redalyc (Red de Revistas Científicas de América Latina y El Caribe, España y Portugal)
`.	Rebuin (Red de Bibliotecas Universitarias)
`.	ScienceDirect
`.	Sello de calidad FECYT 2020
The electronic version of Anuario de Psicología Jurídica is available through the following distributors:
0.	Directory of Open Access Journals0.	 (DOAJ) 
0.	E-libro
0.	Grupo Océano
DOI
Anuario de Psicología Jurídica makes use of DOI, the international code allowing reliable and consistent access to content at any moment.
EDITORIAL PROCESS
Anuario de Psicología Jurídica confirms the receipt of all articles submitted by the authors and keeps them informed by e-mail and on the platform of the admission/dismissal and acceptance/rejection process, as well as the editing process, in the event of acceptance. 
In case the manuscript presents format deficiencies or is not included in the thematic focus of the publication, the Editorial Board will dismiss the manuscript, without a re-submission option. No subsequent correspondence with authors of dismissed manuscripts will be maintained. However, if the manuscript’s deficiencies are superficial, it will be returned to the author for correction before the assessment process begins.
Manuscripts will be reviewed scientifically, anonymously, by two external experts (on average) in the field. In view of these external evaluations, a decision will be made as to whether to accept or reject the articles for publication, as well as the possible introduction of stylistic changes and/or the need to trim texts that exceed the maximum length permitted, always respecting the original content.
Papers that are evaluated positively, requiring modifications (both minor and major), will be returned. All the authors will receive anonymous scientific evaluation reports so the relevant improvements or responses can be made.
The authors of accepted articles will receive the printing proofs for correction by e-mail in PDF format before final publication. The proofread, corrected versions must be returned within three days of their receipt. Only very minor corrections can be made regarding the content of the previously evaluated original manuscript.
Once the manuscript has been definitely accepted, the final version for publication must be returned with all proposed changes in English or Spanish (according to the original version). Before an issue is published, the papers will be given their own DOI in the « Articles in Press» section in the website of the journal.
 In general, once the external scientific reports have been read, the criteria that justify the editors’ decision to accept or reject submissions by Editorial Board are as follows:
?	Current and new.
?	Relevance and significance: advancement of scientific knowledge
?	Originality.
?	Reliability and scientific validity: verified methodological quality.
?	Organisation (logical coherence and formal presentation).
?	External support and public/private funding.
?	Co-authorship and degree of internationalization of the proposal and equipment.
?	Presentation: good written style.
FREQUENCY OF PUBLICATION
Anuario de Psicología Jurídica is published one time a year
FORMAT
The dimensions of the journal are 21.5cm x 27.9cm and it is printed on acid-free permanent paper conforming to ISO 9706:1994.
The print run of Anuario de Psicología Jurídica is 500 copies.


Anuario de Psicología Jurídica is available online at: https://journals.copmadrid.org/apj/
COPYRIGHT
Works that are published in this journal are subject to the following terms:
1) Colegio Oficial de la Psicología de Madrid, as Publisher,  retains the proprietary rights (copyright) of published works, and favors and allows the reuse of the same under the license CC BY-NC-ND 4.0. ©Colegio Oficial de la Psicología de Madrid.
2) The works are published in the online edition of the journal under a license Creative Commons Atribution-NonCommercial-NoDerivates 4.0 Internacional.com: The articles may be copied, used, disseminated, provided that:
`.	Atribution: Cite the authorship and the original source of the publication (journal, publisher, URL and DOI of the work).
`.	NonCommercial:Are not used for commercial purposes.
`.	NoDerivates: If you remix, transform, or build upon the material, you may not distribute the modified material.
`.	Mention the existence and specifications of this license for use.
OPEN ACCESS POLICY
Anuario de Psicología Jurídica [Annual Review of Legal Psychology] is an open access journal.
- Conditions of pre-print self-archiving: Prior to final publication, it is advisable for authors to file their preprint version on their personal and institutional websites, scientific social networks, repositories, bibliographic managers... The preprint must include the following statement: "This is the electronic version of an article accepted for publication in Anuario de Psicología Jurídica [year], now available online on the official website through its DOI: https://doi.org/10.5093/apj...”. Once published, the authors must specify: "This is the electronic version of an article published in Anuario de Psicología Jurídica [year]. The final version is available at the official website, on the date indicated in the preprint, through its DOI".
- Conditions of post-print self-archiving: Authors are allowed to reuse published works, i.e. the post-print (final PDF version of the publisher) can be archived for non-commercial purposes, and authors are strongly recommended to deposit it in:
?	Social networks (Facebook, Twitter, LinkedIn…).
?	Institutional repository of your University and public repositories (Mendeley, Cosis…).
?	Scientific social networks (ResearchGate, Academia.edu, Kudos...).
?	Personal or institutional website, blog, etc.
?	Google Scholar, ORCID, ResearchID, ScopusID...
PERMISSION TO REPRODUCE ARTICLES
Annual Review of Legal Psychology is licensed under a Creative Commons Attribution-NonCommercial-NoDerivatives 4.0 International License
The journal authorizes authors to use published material, at any time and with no ment to seek permission, quoting the journal in which it was published (APA Standards), also is desirable including the link to the specific article webpage.

Likewise, the reproduction of articles without commercial uses is permitted, duly citing the magazine (APA Standards). As a courtesy, we would appreciate if you would inform Colegio Oficial de la Psicología de Madrid of such uses indicating the entity or person who will use the content, as well as its purpose.
For any other questions or additional information you can contact us at: revistas_copm@cop.es
ARCHIVE
This journal uses different national and international repositories that host publications, inter alia, Clarivate Analytics, Scopus, Ebsco, Scielo, Recyt, Redalyc or Dialnet.
PLAGIARISM POLICY
Anuario de Psicología Jurídica uses iThenticate® to detect plagiarism.
ETHICAL STANDARDS
Anuario de Psicología Jurídica subscribes to Ethical Standards of the American Psychological Association, and the legal framework of the country where the research was developed.
SUBSCRIPTIONS
Subcriptions are for a one-year period, from April to December. Prices for the year 2021 are:

Colegio Oficial de Psicólogos de Madrid Members: 33€

Member of other Colegios: 38,50€

Non-member individual: 49€

International subions: 90.50€

Payment is by direct debit, credit card or bank transfer to bank ac: Banco Sabadel IBAN/BIC ES05 0081 0655 6700 0133 7537 / BSAB ESBB

To subscribe, send the completed subscription form with the receipt of payment or bank transfer to:
Departamento de Suscripciones. Colegio Oficial de la Psicología de Madrid.
Cuesta de San Vicente 4, 4ª pl. 28008 Madrid, España
E-mail: suscripciones@cop.es
CONTACT
If you require further information or assistance you can submit a query to María León to publicacionescopm@cop.es or Ana Isabel García: revistas_copm@cop.es 
Colegio Oficial de la Psicología de Madrid (España))
