# Supplementary material for: Peer Review in Law Journals
Source: Front Res Metr Anal. 2021 Dec 8;6:787768. doi: 10.3389/frma.2021.787768 (PMC8692876; doi:10.3389/frma.2021.787768)
Supplement: Supplementary file 3 [file DataSheet2.ZIP › DOCUMENT - 1133-0937_1.RTF]

Editorial Policies
`.	Focus and Scope
`.	Section Policies
`.	Peer Review Process
`.	Open Access Policy
`.	Plagiarism control
`.	Derechos y Libertades indexing
`.	Ethics statement and good practice
Focus and Scope
Derechos y Libertades is the biannual journal published by the Instituto de derechos humanos Gregorio Peces-Barba de la Universidad Carlos III de Madrid. It forms part of the publications of the Institute, together with the collections Cuadernos Bartolomé de las Casas, Traducciones and Debates.
 The purpose of Derechos y Libertades is to constitute a forum for discussion and analysis in relation to the theoretical and practical problems of human rights, from the various perspectives through which they can be analysed, among which the philosophical-legal stand out. In this sense, the journal aims to be a means by which contemporary discussion in the field of Philosophy of Law and Political Philosophy is reflected.
Derechos y Libertades is presented at the same time as a means of expression and publication of the main activities and research carried out within the Gregorio Peces-Barba Human Rights Institute.
 
Section Policies
Nota del Director
 Open Submissions	 Indexed	 Peer Reviewed	
Artículos
 Open Submissions	 Indexed	 Peer Reviewed	
Recensiones
 Open Submissions	 Indexed	 Peer Reviewed	
Noticias
 Open Submissions	 Indexed	 Peer Reviewed	
 
Peer Review Process
All articles are reviewed by at least two reviewers in a double blind system. In case of doubt between two contradictory evaluations, a third report is requested.
The evaluators must complete an evaluation format found on the Journal's website. The evaluators have one month to make the report.
The receipt of an original, to be included in the Articles section, does not presuppose acceptance for publication. The originals are, first, read by the Editorial Board to see if they meet both the formal requirements indicated, as well as minimum scientific content and adaptation to the editorial lines and objectives of the Journal.
 The referees will be anonymous and may recommend to the author the introduction of certain modifications. In the situation that two reports received on an original are contradictory, a third external reviewer will be consulted. Authors whose articles have obtained a favorable opinion but with a suggestion of corrections will send it back to the Journal, once the improvements have been incorporated. In the case of draft modifications, the article will again be subjected to an external evaluation. In case of non-acceptance, the decision will be communicated to the author of the work together with the content of the respective reports.
 
Open Access Policy
Derechos y Libertades provides unrestricted access to all of its content from the time of publication in this electronic edition. The publication has no cost for the authors.
 
Plagiarism control
Before the evaluation, all the works are submitted to an antiplagium control using the Feedback studio application.
 
 
Derechos y Libertades indexing
The Journal Derechos y Libertades is indexed in the Emerging Sources Citation Index de la Web of Science.  It is also included in ERIH, European Index for the Humanities and Social Sciences.
The Journal passed the Call for Evaluation of Editorial and Scientific Quality of the Spanish Scientific Journals and obtained the Seal of Quality FECYT until 2020.
Derechos y Libertades has obtained the category A in the edition 2014 of the CARHUS database. It is indexed in the category B1 of Qualis -Brasil- and the category A in Anvur -Italy-
The Journal has been indexed in Philosopher´s Index, International Political Science Abstracts, Worldwide Political Science Abstracts and the International Bibliography of Social Science (IBSS) databases. It has been included in Dulcinea database as well.
 
 
Ethics statement and good practice
Publication and authorship
1. All submitted papers are subject to strict peer-review process by at least two national and international reviewers that are experts in the area of the particular paper.
2. The factors that are taken into account in review are relevance, soundness, significance, originality, readability and language.
3. The possible decisions include acceptance, acceptance with revisions, or rejection.
4. If authors are encouraged to revise and resubmit a submission, there is no guarantee that the revised submission will be accepted.
5. Rejected articles will not be re-reviewed.
6. The paper acceptance is constrained by such legal requirements as shall then be in force regarding libel, copyright infringement and plagiarism.
7. No research can be included in more than one publication.
8. Authors must certify that their manuscripts are their original work.
 
Author’s responsibilities

9. Authors must certify that the manuscript has not previously been published elsewhere.
10. Authors must participate in the peer review process.
11. Authors are obliged to provide retractions or corrections of mistakes.
12. All Authors must have significantly contributed to the research.
13. Authors must state that all data in the paper are real and authentic.
14. Authors must notify the Editors of any conflicts of interest.
15. Authors must identify all sources used in the creation of their manuscript.
16. Authors must report any errors they discover in their published paper to the Editors.

Reviewers’ responsibilities

17. Reviewers should keep all information regarding papers confidential and treat them as privileged information.
18. Reviews should be conducted objectively, with no personal criticism of the author
19. Reviewers should express their views clearly with supporting arguments
20. Reviewers should identify relevant published work that has not been cited by the authors.
21. Reviewers should also call to the Editor in Chief's attention any substantial similarity or overlap between the manuscript under consideration and any other published paper of which they have personal knowledge.
22. Reviewers should not review manuscripts in which they have conflicts of interest resulting from competitive, collaborative, or other relationships or connections with any of the authors, companies, or institutions connected to the papers.
 
Editor’s responsibilities
23. Editors have complete responsibility and authority to reject/accept an article.
24. Editors are responsible for the contents and overall quality of the publication.
25. Editors should guarantee the quality of the papers and the integrity of the academic record.
26. Editors should publish errata pages or make corrections when needed.
27. Editors should have a clear picture of a research's funding sources.
28. Editors should base their decisions solely one the papers' importance, originality, clarity and relevance to publication's scope.
29. Editors should not reverse their decisions nor overturn the ones of previous editors without serious reason.
30. Editors should preserve the anonymity of reviewers.
31. Editors should ensure that all research material they publish conforms to internationally accepted ethical guidelines.
32. Editors should only accept a paper when reasonably certain.
33. Editors should act if they suspect misconduct, whether a paper is published or unpublished, and make all reasonable attempts to persist in obtaining a resolution to the problem.
34. Editors should not reject papers based on suspicions, they should have proof of misconduct.
35. Editors should not allow any conflicts of interest between staff, authors, reviewers and board members.
