# Supplementary material for: Peer Review in Law Journals
Source: Front Res Metr Anal. 2021 Dec 8;6:787768. doi: 10.3389/frma.2021.787768 (PMC8692876; doi:10.3389/frma.2021.787768)
Supplement: Supplementary file 3 [file DataSheet2.ZIP › DOCUMENT - 1134-6035_1.RTF]

About the Journal
Focus
The journal Gestión y Análisis de Políticas Públicas (GAPP) aims at to promote theoretical and empirical analysis on public management and administration and on public policy in any sector. For this purpose, GAPP publishes both academic papers and contributions from public administration professionals that serve to improve those public policies. GAPP accepts for review and possible publication scientific studies, experiences and research cases, as well as book reviews.
As of January 2021, GAPP publishes three issues per year, two ordinary, dated July 1 and November 1, respectively, and an extraordinary monograph, published on March 1, all edited only in digital format.
Texts submitted to GAPP must be originals in Spanish or English. Texts must not have been published previously and must follow the guidelines accepted by the scientific community because this is the only mechanism that guarantees the indexing of the journal and its quality.
Articles received are subject to a rigorous double-blind peer review process according to the protocol of the Open Journal System.
GAPP believes in the free dissemination of scientific knowledge and, therefore, follows open access policies over its content.
GAPP is edited by the Instituto Nacional de Administración Pública (INAP), under the authority of Ministerio de Política Territorial y Función Pública, and is financed exclusively from the budget of the publishing organization.
Peer review process
Ethical principles
Editorial activity statistics since 01-01-2021
Indexing
Open access and copyright policy/a
Funding and publication charges (APCs)
Anti-Plagiarism Policy
Preservation and Archiving Policy
Interoperability protocols
