# Supplementary material for: Peer Review in Law Journals
Source: Front Res Metr Anal. 2021 Dec 8;6:787768. doi: 10.3389/frma.2021.787768 (PMC8692876; doi:10.3389/frma.2021.787768)
Supplement: Supplementary file 3 [file DataSheet2.ZIP › DOCUMENT - 1134-6035_2.RTF]

Reviewers guidelines
COMPETING INTEREST
GAPP uses the "double blind" peer review evaluation system, so that neither authors nor reviewers know the identity of the other party.
In the event that the author is identified even though, their names have been removed from the manuscript, the reviewers must declare any conflict of interest and reject the publisher's invitation to evaluate a manuscript.
A conflict of interest may occur as a result of:
`.	Animosity towards the authors;
`.	Academic or family closeness:
()	They belong to the same university, department, research group, professional network, research project, etc.
()	Have published articles with the author.
`.	Any other type of connection or conflict / professional proximity.
On the other hand, if you suspect that an article is a substantial copy of another work, the evaluator must inform the editors. Likewise, publishers will be informed if there is a suspicion that the results of the article are false or fraudulent.
EVALUATION OF ARTICLES
Reviewers will consider the following criteria in order to accept or reject the evaluation of a manuscript:
`.	They will have knowledge and experience in the topic discussed. They should only perform their function if they consider themselves competent in the matter they are proposed to evaluate.
`.	They undertake to respect the deadlines established for review (4 weeks from the receipt of the manuscript). In case of refusal, they will communicate to Secretary as soon as possible.
`.	They will assume a commitment of confidentiality so that they may not, throughout the process, disclose the content of the article to a third party. The only authorized interlocutors for any issue related to the evaluation process are the director and editors of the journal.
Indications for review
Once the review has been accepted, the reviewers shall carry out an objective, technical and constructive review of the article or book (subject to evaluation).
The task of the reviewers in the evaluation process includes:
0.	Checking that the article meets the guidelines of the journal’s review policy.
0.	Filling out the peer review form created by the journal’s editor, adding comments to the author for the manuscript review: necessary suggestions, improvements or reforms to enable the article to be published.
0.	Adding confidential comments to editors: any other consideration in relation to the quality and acceptability of the manuscript, or any comment that you do not wish to pass on to the author.
0.	Submitting the evaluation through the journal’s website to the editors issuing the corresponding recommendation.
0.	Here you can see the peer review form.
