# Supplementary material for: Peer Review in Law Journals
Source: Front Res Metr Anal. 2021 Dec 8;6:787768. doi: 10.3389/frma.2021.787768 (PMC8692876; doi:10.3389/frma.2021.787768)
Supplement: Supplementary file 3 [file DataSheet2.ZIP › DOCUMENT - 1134-6035_3.RTF]

About the Journal
Peer review process
The journal guarantees that the maximum review time for an article is two months.
1. Authors must consult the guidelines for submissions before sending a publication proposal. The submission will be accompanied by a brief note highlighting the contribution that the text has made to the discipline and guaranteeing the originality of the article, as well as the fact that it is not under review in another journal.
2. If the text in case meets all the formal requirements, the author will be informed and the evaluation process will begin.
3. Articles received are subject to an initial review by the GAPP Editorial Board, which will assess the quality and thematic adequacy of the work and may be directly rejected without undergoing an external review. For this first review, the Editorial Board may require the assistance of members of the Advisory Board, if deemed necessary.
4. If the article overcomes this first filter, a "double blind" review process begins, in which neither the authors nor the reviewers know the identity of the other party. Articles will be sent to two external reviewers, specialists in the field or line of research, together with the article review form. In the case that the evaluations differ, or for any other reason, the Editorial Board may send the text to a third reviewer
5. In view of the reports of the reviewers, the Editorial Board may adopt one of the following decisions that will be communicated to the author(s):
`.	Publishable as it is (or with minor revisions).
`.	Publishable after revision. In this case, publication will depend on the realization by the author(s) of all the changes required by the editors. The deadline for making these changes will be two months. The author must attach a brief report explaining the changes introduced and how they comply with the requirements of the Editorial Board. Among the proposed changes may be the adaptation of the article to another section.
`.	Not publishable, but with the possibility of rewriting and resending the article. In this case, the resubmission of a new version will not imply any guarantee of publication; rather, the evaluation process will start again from the beginning.
`.	Not publishable.
6. GAPP will publish annually the list of all the people who have conducted anonymous evaluations. 
7. If it is led to discover any of them, GAPP may make public any of the following scientific malpractices: plagiarism, falsification or invention of data, individual appropriation of collective authorship and duplicate publication.
