# Supplementary material for: Peer Review in Law Journals
Source: Front Res Metr Anal. 2021 Dec 8;6:787768. doi: 10.3389/frma.2021.787768 (PMC8692876; doi:10.3389/frma.2021.787768)
Supplement: Supplementary file 3 [file DataSheet2.ZIP › DOCUMENT - 1138-4026_1.RTF]

Política editorial y modalidades de publicación
La Revista de Derecho Comunitario Europeo (RDCE) publica desde 1974 trabajos originales de investigación sobre derecho europeo en tres modalidades: estudios, notas y comentarios de jurisprudencia.
Todos los trabajos deben ser inéditos, por lo que no se publicarán aquellos publicados con anterioridad en revistas, libros o actas de congresos, con independencia del formato utilizado. Los autores asumen el compromiso de no haber publicado con anterioridad el mismo trabajo y se comprometen a no someterlo a consideración para publicación en otros medios durante el proceso de revisión. La RDCE podrá, en todo momento del proceso de edición, someter el manuscrito enviado a uno o varios de los programas antiplagio existentes a fin de detectar su carácter original así como respetuoso de la autoría intelectual ajena.
La dirección de la RDCE podrá encargar en determinados casos una reseña o comentario. Solo se publicarán las recensiones encargadas por la RDCE.
 
Envío de originales
Los artículos se enviarán en formato electrónico (*.doc o *.docx) a través del Repositorio Español de Ciencia y Tecnología (RECYT), al que se accede a través de la url: https://recyt.fecyt.es/index.php/RDCE/index. La plataforma RECYT es un repositorio de revistas académicas de calidad mantenido por la Fundación Española para la Ciencia y la Tecnología. Una vez que se da de alta como autor, puede enviar el artículo a la revista a través de la plataforma, saber en qué estado de tramitación se encuentra y actualizar sus datos de contacto y su perfil biográfico.
 
Una vez que tiene el artículo listo, se ha asegurado de que cumple con las normas de formato y citas de la revista [véase más abajo], si no está registrado con anterioridad en RECYT, puede darse de alta en la plataforma para enviar el artículo del siguiente modo:
 
1. Acceda a https://recyt.fecyt.es/index.php/RDCE/user/register, rellene el perfil de usuario y pulse «registrar».
2. Con la claves entre en la revista (http://recyt.fecyt.es/index.php/RDCE/login). Vaya a la sección «mis revistas» (disponible en la barra de navegación lateral).
3. Pinche con el ratón en «Autor» (en azul) que es el rol del usuario en la revista.
4. Pinche en HAGA CLIC AQUÍ PARA COMENZAR EL PROCESO DE ENVÍO.
5. Siga las instrucciones. 
 
En caso de que el autor experimente dificultades con la plataforma, puede comunicarlo mediante un correo electrónico a nombre del director ejecutivo de la RDCE a public@cepc.es. Se acusará recibo de todos los originales en el plazo de treinta días desde su recepción.
 
Idiomas de los originales
La RDCE acepta trabajos en español y, en caso de autores no hispanohablantes, en inglés. Los originales se publicarán en el idioma en el que fueron escritos. Si se envía una versión en inglés, su calidad lingüística debe estar garantizada, por lo que el texto debe haber sido escrito o revisado por una persona nativa de lengua inglesa. Excepcionalmente, la Revista también publicará manuscritos en francés, aplicándose las mismas reglas de garantía de la calidad lingüística.
 
Formato
Los originales habrán de presentarse en formato Word (.doc o .docx) en Times New Roman tamaño 12, a doble espacio, y con las notas a pie de página en Times New Roman tamaño 10 e interlineado sencillo.
 
En cuanto a su extensión (incluyendo notas a pie de página, pero no los resúmenes y palabras clave en castellano, francés e inglés), se ajustará a los siguientes límites: los estudios no sobrepasarán las 16 000 palabras (aproximadamente 40 páginas); las notas las 10 000 palabras (unas 25 páginas), y los comentarios de jurisprudencia las 6000 palabras (aproximadamente unas 15 páginas, en su caso refiriendo las citas literales de la resolución judicial comentada en las notas a pie de página). El Comité de Redacción podrá considerar, atendidas la entidad del objeto tratado o la valía de la aportación científica, originales que excedan en no más del 15 % de la extensión máxima.
 
Todos los trabajos deben ir precedidos de título, resumen y palabras clave en castellano, inglés (title, abstract, keywords) y francés (titre, résumé, mots clés). La extensión del resumen debe estar comprendida entre 150 y 250 palabras. Los autores son responsables de la corrección lingüística de los manuscritos, incluyendo resúmenes, palabras clave y títulos. También deben ir acompañados de un sumario con los números referidos en el siguiente párrafo.
 
En función de su estructura interna, el artículo debe dividirse en apartados con sus correspondientes títulos numerados. Los apartados principales se identificarán con números romanos (I., II., III., etc.). Los subapartados irán en números arábigos  (1., 2., 3., etc.), pudiéndose también utilizar aquí otros niveles inferiores, indicados igualmente con números arábigos, aunque no es recomendable utilizar más de dos subdivisiones (1.1., 1.2., 1.3., etc.).
 
Al final del trabajo, como se indica a continuación, se incluirá la bibliografía exclusivamente de las fuentes citadas y ordenada alfabéticamente sin ninguna subdivisión.
 
Para garantizar la confidencialidad de las evaluaciones, el texto no debe contener el nombre del autor o autores, ni ninguna referencia que permita ser fácilmente identificado (tales como proyectos, seminarios, reconocimientos, etc.) Adviértase que los procesadores de texto contienen una sección  de metadatos (en Archivo/Propiedades) donde debe borrarse toda información relativa a la autoría u organización. En cualquier caso, el envío de un manuscrito para su evaluación conlleva el consentimiento del autor para su tratamiento digital y la eliminación de estos metadatos. Una vez que el artículo sea aceptado para su publicación, estas referencias serán incluidas más tarde durante su edición.
 
Normas de citación
En consonancia con los actuales estándares internacionales de la edición académica, y con el fin de favorecer la visibilidad, impacto e intercambio digital de contenidos, la RDCE utiliza, desde el primer número de 2018, unas nuevas normas de citación. Ya que se trata de un cambio profundo en el sistema de citación, se ruega a los autores que presten especial atención a las siguientes indicaciones generales.
 
Citas bibliográficas
Aparecerán preferentemente en el cuerpo del texto. No se usarán, por tanto, notas a pie cuyo único contenido sea la indicación de la fuente (cada una de las citas bibliográficas debe corresponderse con una referencia en la bibliografía final). Se empleará el sistema autor-año de Harvard (autor, año: página):
 
— Un autor único se citará, en los casos en que proceda, con ambos apellidos:
 
(Rodríguez Iglesias, 2012: 72), (Louis, 2015)
 
 — Dos autores se citarán por sus primeros apellidos unidos por «y»:
 
 (Mangas y Liñán, 2014: 87-120)
 
 — Tres o más autores se citarán por el primer autor seguido de et al.:
 (Martín Rodríguez et al., 2006), (Kilpatrick et al., 2014: 23-24)
 
 — Si se citan varios trabajos de un autor o grupo de autores de un mismo año, debe añadirse a, b, c… después del año:
 
 (Del Valle Gálvez, 2003a)
 
 — Cuando el apellido del autor citado forme parte del texto, debe indicarse siempre entre paréntesis el año de la obra citada, eventualmente al final de la citación textual:
 
Como afirma Gosalbo Bono, «Esta ratio decidendi del fallo también podría servir de base para impugnar otros acuerdos comerciales» (2016: 74).
 
Notas a pie
Se situarán a pie de página, numeradas mediante caracteres arábigos y en formato superíndice. Únicamente contendrán texto adicional y si incluyen referencias bibliográficas, se hará de forma abreviada, según se acaba de indicar en estas instrucciones, ya que las referencias completas se incluirán al final del artículo.
 
Citas de jurisprudencia
Cuando la referencia a una sentencia o jurisprudencia se realice en el texto principal, utilizará la cursiva para referirse a los nombres de las sentencias, incluidos aquellos usados comúnmente:
 
Tal y como se deriva de lo sostenido por el TJUE en la sentencia Melloni,...
La jurisprudencia AETR obliga a...
El TEDH en el asunto Soering,
 
Las resoluciones judiciales citadas se acompañarán de su cita completa en nota a pie. La cita de jurisprudencia international, europea y nacional se realizará, en general, siguiendo los criterios de cita del país o sistema de emisión del fallo y, siempre que esto sea posible, indicando el identificador europeo de jurisprudencia (ECLI). En caso de que se utilice el ECLI, no serán necesarias referencias adicionales a colecciones jurisprudenciales o publicaciones oficiales.
 
En caso de duda, la citación oficial podrá sustituirse por el siguiente modelo: Resolución del Órgano judicial de fecha, nombre o número del asunto y ECLI.
 
A continuación, tiene ejemplos de los principales Tribunales europeos:
Tribunal de Justicia de la Unión Europea [método de citación oficial]
Sentencia del Tribunal de Justicia de 15 de mayo de 1986, Johnston, 222/84, EU:C:1986:206.
Sentencia del Tribunal General de 30 de septiembre de 2010, Kadi/Comisión, T-85/09, EU:T:2010:418, apartado 78
Conclusiones del abogado general Jacobs, Unión  de  Pequeños  Agricultores/Consejo, C-50/00 P, EU:C:2002:197, punto 199.
 
Recuérdese que en español, a diferencia del inglés o del francés, los nombres de los asuntos siempre son abreviados y cortos, tal como figuran en el encabezado del texto español de las sentencias. Por ejemplo, el nombre del asunto es «Johnston» en español, a diferencia del inglés «Johnston vs. Chief Constable of the Royal Ulster Constabulary».
Obsérvese igualmente que en la citación oficial, los nombres de los asuntos no van en cursiva
Tribunal Europeo de Derechos Humanos  [método de citación oficial]
Campbell vs. Ireland [GC], no. 45678/98, § 24, ECHR 1999-II.
Campbell vs. Ireland, no. 45678/98, § 24, ECHR 1999-II.
Campbell vs. Ireland (dec.), no. 45678/98, ECHR 1999-II.
 
Pero también, se puede utilizar y recomendamos la cita mediante el ECLI según la siguiente fórmula
Sentencia del TEDH de 17 de enero de 2012, Zontul vs. Grèce, CE:ECHR:2012:0117JUD001229407.
 
Citas de legislación y otros documentos oficiales
Los actos jurídicos y otros documentos oficiales se citarán en nota a pie, haciendo referencia a su publicación oficial, en caso de existir:
 
Directiva 2008/115/CE del Parlamento Europeo y del Consejo, de 16 de diciembre de 2008, relativa a normas y procedimientos comunes en los Estados miembros para el retorno de los nacionales de terceros países en situación irregular (DO L 348, de 24 de diciembre de 2008, p. 98)
Convención de Viena sobre el Derecho de los Tratados, de 23 de mayo de 1969 (U.N.T.S., vol. 1155, p. 331).Texto refundido de la Ley del Estatuto de los Trabajadores, aprobado por el Real Decreto Legislativo 1/1995, de 24 de marzo (BOE 75, de 29 de marzo de 1995, p. 9654)
Ley sobre la orden europea de detención y entrega (Europäisches Haftbefehlsgesetz), de 20 de julio de 2006 (BGBl. 2006 I, p. 1721).
Libro Verde de la Comisión Europea, «Un mercado integrado de los servicios de entrega para impulsar el comercio electrónico en la UE», COM (2012) 698 final, 29-11-2012. Pero también (Comisión Europea, 2012: 4).
 
Bibliografía
Se incluirá al final del trabajo. Únicamente contendrá referencias citadas en el texto, sin posibilidad de incluir referencias adicionales. Se seguirá el sistema APA (American Psychological Association). La bibliografía se ordenará alfabéticamente sin subdivisiones. A continuación adjuntamos ejemplos para las tipologías más usadas.
 
Artículos de revistas científicas
Besselink, L. F. M. (2014). The parameters of constitutional conflict after Melloni. European Law Review, 39 (4), 531-552.
Pons Rafols, F. X. (2014). Un paso importante para el desarrollo de nuestra justicia constitucional: la doctrina prospectiva en la declaración de ineficacia de las leyes inconstitucionales. Revista de Derecho Comunitario Europeo, 47, 131-156.
 
 — Dos o más autores
Arroyo Gil, A. y Giménez Sánchez, I. (2013). La incorporación constitucional de la cláusula de estabilidad presupuestaria en perspectiva comparada: Alemania, Italia y Francia. Revista Española de Derecho Constitucional, 98, 149-188.
 
— Revistas electrónicas
Olesti Rayo, A. (2015). La financiación del presupuesto de la Unión Europea y la necesidad de revisar el sistema de recursos propios. Revista General de Derecho Europeo, 37. Disponible en: http://www.iustel.com/v2/revistas/detalle_revista.asp?id=13&z=1.
 
— Revistas electrónicas con DOI
Andrés Sáenz de Santa María, P. (2016). La Unión Europea y el Derecho de  los Tratados: Una relación compleja. Revista Española de Derecho Internacional, 68 (2), 51-102. Disponible en: https://doi.org/10.17103/redi.68.2.2016.1.02.
 
Monografías
— Un autor
López Escudero, M. (2004). El euro en el sistema monetario internacional. Madrid: Tecnos.
Peers, S. (2011). EU Justice and Home Affairs Law (3.ª ed.). Oxford: Oxford University Press.
 
 — Libro en línea
Martín y Pérez de Nanclares, J. (dir.). (2014). España y la práctica del Derecho Internacional. Madrid: Escuela Diplomática. Disponible en: http://www.exteriores.gob.es/Portal/es/Ministerio/EscuelaDiplomatica/Documents/Coleccion_ED_20_web.pdf.
 
Capítulos de monografías
Meyer-Ladewig, J. (2012). The Rule of Law in the Case Law of the Strasbourg Court. En H. J. Blanke y S. Mangiameli (eds.). The European Union after Lisbon (pp. 233-249). Heidelberg: Springer.

Informes
— Autor institucional
Europol (2011a). EU Organised Crime Threat Assessment. La Haya: Europol Public Information.
 
— Autor personal
Levi, M., Innes, M., Reuter, P. y Gundur, R. (2013). The Economic, Financial and Social Impacts of Organised Crime in the EU. Bruselas: Parlamento Europeo, PE 493.018. Disponible en: http://www.europarl.europa.eu/RegData/etudes/etudes/join/2013/493018/IPOL-JOIN_ET(2013)493018_EN.pdf.

Blogs
Sarmiento, D. (2016). Awakenings. Despite our Differences [blog], 27-1-2016. Disponible en: https://despiteourdifferencesblog.wordpress.com/2016/01/27/awakenings/.

Tesis
— Publicadas
Ruiz Díaz, L. J. (2015). La lucha contra el crimen organizado en la Unión Europea. Aspectos internos y dinámicas externas del discurso securitario [tesis doctoral]. Univesidad de Granada. Disponible en: http://0-hera.ugr.es.adrastea.ugr.es/tesisugr/25575715.pdf.
 
— Inéditas
Liñán Nogueras, D. J. (1978). La integración como factor de modificación del concepto de soberanía [tesis doctoral inédita]. Universidad de Granada. 

Artículos y noticias de periódicos
Vidal-Folch, X. (2017). Momento para Europa. El País, 25-9-2017. Disponible en: https://elpais.com/elpais/2017/09/24/opinion/1506249361_065083.html.
 
Con respecto a las fuentes electrónicas utilizadas, una declaración general en la primera nota, indicando que todas las fuentes han sido recuperadas en una fecha idéntica puede utilizarse para evitar especificar la fecha concreta del último acceso respecto de cada fuente citada. Esta opción es vivamente recomendada por la RDCE.

DOIS
En el caso de que los trabajos en formato electrónico contengan DOI (digital object identifier), será obligado recogerlo en la referencia bibliográfica. Se hará del siguiente modo:
 
Murray, S. (2006). Private Polls and Presidential Policymaking. Reagan as a Facilitator of Change. Public Opinion Quarterly, 70 (4), 477-498. Disponible en: http://dx.doi.org/10.1093/poq/nfl022.
 
Proceso de publicación
El Comité de Redacción revisará, en primer lugar, la calidad de los trabajos (estudios y notas). Si los considera adecuados, decidirá su publicación sobre la base de dos informes de evaluación, emitidos por sendos especialistas ajenos a la organización editorial de la RDCE, aplicándose el método doble ciego. Se valorarán la creatividad, rigor, metodología, propuestas y aportaciones de los trabajos. El proceso interno de evaluación garantizará el anonimato. La publicación podrá quedar condicionada a la introducción de cambios con respecto a la versión original, y sujeta a modificaciones conforme a estas Instrucciones para los autores .
La comunicación de la decisión sobre la publicación —condicionada o no a la introducción de las modificaciones pedidas— o la no publicación de los originales enviados, será motivada y no excederá de seis meses. Los autores de trabajos aceptados para publicación podrán ser requeridos para la corrección de pruebas de imprenta, que habrán de ser devueltas en el plazo de 48 horas. No se permitirá la introducción de cambios sustanciales en las pruebas, quedando estos limitados a la corrección de errores con respecto a la versión aceptada.
 
Copyright
En el momento en que una obra es aceptada para su publicación, se entiende que el autor cede a la RDCE en exclusiva los derechos de reproducción, distribución y, en su caso, venta de su manuscrito para su explotación en todos los países del mundo en formato de revista de papel, así como en cualquier otro soporte magnético, óptico y digital.
Los autores cederán también a la RDCE los derechos de comunicación pública para su difusión y explotación a través de intranets, internet y cualesquiera portales y dispositivos inalámbricos que decida el editor, mediante la puesta a disposición de los usuarios para consulta online de su contenido y su extracto, para su impresión en papel y/o para su descarga y archivo, todo ello en los términos y condiciones que consten en la web donde se halle alojada la obra. A su vez, la RDCE autoriza a los autores de los trabajos publicados en la revista a que ofrezcan en sus webs personales o en cualquier repositorio de acceso abierto una copia de esos trabajos una vez publicados. Junto con esa copia ha de incluirse una mención específica de la RDCE, citando el año y el número de la revista en que fue publicado el artículo o nota de investigación y añadiendo, además, el enlace a la web de la RDCE.
Transcurrido un año desde su publicación, los trabajos pasarán a estar sujetos a la Licencia de reconocimiento de Creative Commons Reconocimiento-No comercial-Sin obra derivada 4.0 Internacional, que permite a terceros compartir la obra siempre que se indique su autor y su primera publicación en esta revista.
 
Plagio y fraude científico
La publicación de un trabajo que atente contra los derechos de propiedad intelectual será responsabilidad de los autores, que serán los que asuman los conflictos que pudieran tener lugar por razones de derechos de autor. Los conflictos más importantes pueden darse por la comisión de plagios y fraudes científicos. Se entiende por plagio:
     a. Presentar el trabajo ajeno como propio.
     b. Adoptar palabras o ideas de otros autores sin el debido reconocimiento.
     c. No emplear las comillas u otro formato distintivo en una cita literal.
     d. Dar información incorrecta sobre la verdadera fuente de una cita.
     e. El parafraseo de una f uente sin mencionar la fuente.
     f. El parafraseo abusivo, incluso si se menciona la fuente.
 
 Las prácticas constitutivas de fraude científico son las siguientes:
     a. Fabricación, falsificación u omisión de datos y plagio.
     b. Publicación duplicada.
     c. Conflictos de autoría.
 
Advertencia
Cualquier incumplimiento de las presentes Instrucciones, así como de la Guía de estilo, constituirá motivo para el rechazo del original remitido.
