# Supplementary material for: Peer Review in Law Journals
Source: Front Res Metr Anal. 2021 Dec 8;6:787768. doi: 10.3389/frma.2021.787768 (PMC8692876; doi:10.3389/frma.2021.787768)
Supplement: Supplementary file 3 [file DataSheet2.ZIP › DOCUMENT - 1138-9877_1.RTF]

Home > About the Journal > Editorial Policies
`.	Focus and Scope
`.	Section Policies
`.	Peer Review Process
`.	Publication Frequency
`.	Open Access Policy
`.	Archiving
`.	Ethical standards and declaration of good practices
`.	CEFD Indexation
`.	System Against Plagiarism
`.	Editor
Focus and Scope
Cuadernos Electrónicos de Filosofía del Derecho (CEFD) is a semiannual electronic journal published from the Human Rights Institute, University of Valencia.
From its first issue published in 1998, the journal includes original scientific contributions mainly related to the area of philosophy of law and political philosophy. In this sense, the purpose of CEFD is the dissemination of knowledge, reflection and scientific debate from different perspectives of the iusphilosophic analysis. CEFD is published online and following the characteristic of open source which offers the possibility of a greater diffusion of its contents, which are accessible to all the scientific and professional community of any legal discipline interested.
This journal provides free and immediate access to its content under the maxim that establishes that by making the results of research available to the public free of charge, a greater knowledge exchange is supported at a global level. CEFD is a magazine that does not charge authors for publication, nor for the management of originals. Since the publication of the number 18 Cuadernos Electrónicos de Filosofía del Derecho follow a rigorous process of external peer review system of the submitted works, in order to guarantee a high level of scientific quality in each edition
CEFD is indexed at: DIALNET, IN-RECJ, DICE, RESH, CARHUS PLUS (Law: A; Philosophy: B), ESCI, EBSCO, CSIC-CCHS, ERIH PLUS, Dulciena, Sherpa / Romeo, MIAR, REDIB, Journal Scholar Metrics, Ulrich's, WorldCat, ISOC and DOAJ. Likewise, since 2016 it has the FECYT quality seal. More information.
Pieces of research can be remitted to its Editorial Board, according to the terms and formal criteria indicated in the edition rules section, only using the OJS (Open Journal System) application.
 
Section Policies
ARTICLES
 Open Submissions	 Indexed	 Peer Reviewed	
BOOK REVIEWS
 Open Submissions	 Indexed	 Peer Reviewed	
CONFERENCE PROCEEDINGS. INTERNATIONAL CONGRESS ABOUT THE 70TH ANNIVERSARY OF THE UNIVERSAL DECLARATION OF HUMAN RIGHTS
 Open Submissions	 Indexed	 Peer Reviewed	
DEBATES
 Open Submissions	 Indexed	 Peer Reviewed	
WEIMAR MOMENTS
Editors
`.	Andrés Gascón-Cuenca
 Open Submissions	 Indexed	 Peer Reviewed	
 
Peer Review Process
Only applications whose author is linked with a University or Higher Centre of Research as a Porfessor, PhD. candidate or Postdoctoral researcher with an official scholarship will be processed. 
Papers in the “Articles" section will be reviewed with an strict anonymously process by two experts who will evaluate if the work meets the established criteria of quality of CEFD. More information is provided in the section "Ethical standards and declaration of good practices".
Papers of the sections “Debates” and "Recensiones" can be communications and texts previously presented in congresses, conferences and conferences. They are not subject to the evaluation of arbitrators.
 
Publication Frequency
CEFD is a biannual publication. The contents will be renewed in June and December each year.
 
Open Access Policy
CEFD is an open access publication, which means that its contents are made available to the public completely free of charge. Users can read, download, print, distribute, or link the distributed texts without having to ask permission from the authors or the magazine. These guidelines follow the BOAI definitions regarding the interpretation of open access
 
Archiving
This review uses the LOCKSS system to create a distributed file among the participating libraries, allowing these libraries to create permanent archives of the journal for preservation and restoration purposes. More information here.
 
Ethical standards and declaration of good practices
Cuadernos Electrónicos de Filosofía del Derecho is an academic review that publishes scientific works and assumes a firm commitment with the fulfillment of the ethical standards and good practices. The Steering Committee and the Editorial Board are responsible for determining and maintaining the following standards in the process of selecting and accepting contributions submitted, as well as assuming the responsibilities arising from the publication process. Likewise, they are committed to ensuring the ethical and scientific and academic quality of the journal, and that all parties involved accept and respect the principles that follow.
 
Obligations of authors
Authors of articles must respect the following obligations, as well as comply with the instructions for the authors that can be found on this website and that are included in each number.
Authors must submit manuscripts that are not subject to consideration or that have been previously sent to other publications, and submit them respecting the indications included in the submitting process to assure an anonymous review process. The proposed research must have been developed in accordance with ethical standards, that must be included in the text of the publication. All authors must mention the funding sources that have allowed them to carry out the research and achieve the relevant results, as well as the possible influence of these on them.
The submitted articles must be completely original works and, in the case that the authors have used the work or fragments of others authors, they must be cited in an appropriate manner, according to the aforementioned indications. Any form of plagiarism (whether direct or indirect) is a practice contrary to these good practices and will not be accepted under any circumstances. It is not appropriate for an author to submit more than one paper describing the same research, unless it is a remission of an article rejected for publication in another journal. In case of knowing a fundamental error or a relevant inaccuracy in the works already published, the authors should communicate them to the journal immediately.
 
Ethical Obligations of Editors
Editors are responsible for complying with the following obligations. They must ensure the scientific and academic quality of Cuadernos Electrónicos de Filosofía del Derecho.
Editors must respect the intellectual independence of the authors and consider all the originals submitted for publication, evaluating each of the contributions objectively. They are also responsible for guaranteeing both authors and reviewers that manuscripts will be submitted to a rigorous anonymous review process. The final decision about the acceptance or rejection of an original will fall on the Editorial Board always basing their decision on the content the opinion of the experts.
It is the obligation of editors to look for reviewers, who will be chosen because of their prestige, knowledge and good judgment. Experts will value the scientific quality of the works submitted to the journal. However, papers may be rejected for publication without external review if, in the opinion of the editors, the work is inappropriate for publication in the journal. This decision should be based on the inadequacy of the work to the content and line of the journal, the absence of current or sufficient interest, formal incorrectness, or any other reason related to the scope of the journal. Articles, therefore, will be valued solely on the basis of their content, regardless of commercial interests and without discrimination of an ethnic, racial, ideological, religious, gender or any other kind towards the author.
Editors must communicate the result of the evaluation reports to authors without revealing any type of information about the identity of the reviewers, but they will inform about the criteria used by them when evaluating the work in view of its publication.
The members of the Editorial Board, reviews and any other person in contact with the original texts  submitted for consideration, must not disclose any information about them to anyone other than those to whom academic or scientific advice is requested. Confidentiality should be protected in all cases during the review process (from the reviewers to the author, and vice versa). The editors will abstain from participating in the evaluation and any other editorial process that includes manuscripts in case of having a conflict of interest because of a possible competitive, collaborative or other relationship with any of the authors of the submitted manuscript.
Unpublished information, arguments or interpretations contained in an original submitted to the journal may not be used in research developed by the editors.
 
Obligations of reviewers
Reviewers have the duty of objectively assess the quality of the texts, as well as their originality with special emphasis on the maintenance of scientific quality and scientific standards. Reviewers help editors in making decisions and can assist authors in improving their work.
Reviewers will advise the editor of any substantive similarity between the manuscript under review and any similar article or manuscript sent or published in any other journal.
Reviewers must act in an appropriate manner, submitting their assessment report within the established deadline. When a proposed reviewer considers that he or she is not the right person to evaluate the submitted research or that will not be able to meet the deadlines for the review, he or she should notify the editor and apologize as soon as possible.
Reviewers must consider the originals received for review as a confidential document. It should never be shown or discussed with third parties, except in exceptional cases where people who can advise scientifically or academically may be consulted. In these cases, the identities of the people consulted must be revealed to the editor.
 
Corrections and retraction
In case of recognizing any information published in the journal Theory & Law as false, erroneous, deceitful or fraudulent, the editors should inform the authors of these circumstances and wait for a response from them before making an editorial decision. In case of not being satisfied with the answer or if there is not any, the editors may decide to retract what was published after agreement of the Editorial Board.
The journal Cuadernos Electrónicos de Filosofía del Derecho will answer any complaint and complaint about the contents published through the address cuadernos@uv.es. The editors agree to follow up and proceed with the review and, if necessary, retraction if these are necessary.
When a retraction or correction of what is published in the journal is appropriate, the editors will be guided by the principles contained in the Guidelines for Retracting Articles of the Committee on Publication Ethics (COPE).
 
