# Supplementary material for: Peer Review in Law Journals
Source: Front Res Metr Anal. 2021 Dec 8;6:787768. doi: 10.3389/frma.2021.787768 (PMC8692876; doi:10.3389/frma.2021.787768)
Supplement: Supplementary file 3 [file DataSheet2.ZIP › DOCUMENT - 1139-5583_1.RTF]

FORMULARIO DE EVALUACIÓN

Teoría y Realidad Constitucional somete a evaluación los originales que le son remitidos para publicación. Gracias a la colaboración científica de quienes acceden a remitirnos los informes solicitados, la Revista cuenta con un criterio fundamental para continuar ofreciendo a sus lectores trabajos de calidad e interés.

Adjuntamos aquí para conocimiento general el esquema mínimo a incluir en tales informes.


TÍTULO DEL TRABAJO:

1. El trabajo evaluado

a) se incardina plenamente, por contenido y/o calidad, en la línea de publicaciones de Teoría y Realidad Constitucional

b) se aparta considerablemente, por contenido y/o calidad, de la línea de la línea de publicaciones de
Teoría y Realidad Constitucional
c) por su contenido y calidad podría enriquecer la línea de publicaciones de la Revista

2. El trabajo

`)	tiene un notable interés teórico o histórico para el ámbito del Derecho constitucional

`)	tiene notable interés práctico para el Derecho constitucional español
`)	tiene notable interés práctico para el Derecho constitucional comparado
`)	carece de interés relevante en la materia

3.

¿En qué medida el trabajo	ninguna   Poca  bastante  mucha	
-	recoge el status quaestionis sobre la materia abordada?

-	aborda cuestiones novedosas?

-	desarrolla argumentos innovadores?

-	propone tesis originales?

4.

¿En qué medida la exposición del texto	ninguna   Poca  bastante  mucha	
-	resulta atractiva y estimulante?

-	resulta rigurosa y bien documentada?

-	se atiene a las usuales convenciones científicas?

-	posee propiedad, precisión, claridad y buena sistemática?

0.	Otras consideraciones:

0.	En síntesis, a su juicio el Consejo Editorial de Teoría y Realidad Constitucional debería

`)	aceptar el trabajo para su publicación en la sección ….

`)	rechazar la publicación del trabajo
`)	dirigirse al autor sugiriéndole que, para proceder a una nueva evaluación de su trabajo, debería reelaborarlo siguiendo los siguientes criterios:
