# Supplementary material for: Peer Review in Law Journals
Source: Front Res Metr Anal. 2021 Dec 8;6:787768. doi: 10.3389/frma.2021.787768 (PMC8692876; doi:10.3389/frma.2021.787768)
Supplement: Supplementary file 3 [file DataSheet2.ZIP › DOCUMENT - 1139-5583_2.RTF]

NORMAS DE PUBLICACIÓN
La Revista Teoría y Realidad Constitucional, plataforma abierta a las inquietudes de los profesores de nuestra especialidad, publica con vocación de excelencia trabajos de investigación originales que ofrezcan una visión dinámica,  dialéctica y de óptimo nivel sobre el constitucionalismo de nuestra época. En estas notas ofrecemos unas sucintas normas de publicación para nuestros colaboradores.
 
— Normas de citas y referencias UNE-ISO 690:2013.
— Los trabajos, inéditos, habrán de ser enviados en lengua española, no debiendo estar pendientes de publicación en ninguna otra revista de ámbito nacional. La revista acusará recibo de los originales enviados por los autores.
— La Revista se reserva la iniciativa, especialmente en números monográficos, de invitar a los más prestigiosos especialistas en la materia, sin perjuicio de las restantes normas de publicación.
— Deberán remitirse por correo electrónico a la dirección TRC@adm.uned.es, indicándose en el archivo el nombre del autor o autores, filiación académica y dirección postal.
— La extensión máxima de los trabajos, a espacio y medio y letra times 12, será la siguiente: Respuestas a la encuesta: 15 págs. Estudios: 25 págs. Notas: 15 págs. Panorámica de otros sistemas constitucionales: 15 págs. Comentarios de jurisprudencia: 15 págs. Dictámenes del Consejo de Estado: 10 págs. Recensiones: 5 págs. No obstante lo anterior, se valorarán las circunstancias de interés del tema al efecto de determinar la extensión máxima.
— En la primera página habrá de incorporarse tras el título del trabajo, el nombre del autor o autores y el correspondiente sumario.
— Al final del trabajo se incorporará el título en inglés del trabajo así como un resumen del texto y palabras clave, en lengua española e inglesa en el orden siguiente: Title, Abstract, Resumen, Key words, Palabras clave.
— Los apartados del trabajo irán numerados del siguiente modo: I, II, III... (mayúscula). 1, 2, 3... (cursiva minúscula) a, b, c… (minúscula)
— La revista publica únicamente las recensiones previamente solicitadas por el Consejo de Redacción.
—Los artículos recibidos serán revisados por evaluadores externos con el sistema de “doble ciego”; no obstante, el Consejo de Redacción podrá rechazar un artículo sin necesidad de proceder a su evaluación en los casos en que el mismo no se adapte a las normas formales o de contenido, o que no se ajuste al perfil temático de la publicación. En el proceso de evaluación se mantendrá el anonimato tanto del autor como de los evaluadores externos, aunque periódicamente se publicará la lista de dichos evaluadores. Una vez finalizado el proceso evaluador se comunicará a los autores la aceptación o rechazo del trabajo; en el caso de aceptación se indicará en la publicación la fecha de remisión y la fecha de aceptación.
—La revista, a través de la Universidad, se sirve del programa "turnitin" para detectar posibles plagios. Todas las coincidencias que puedan encontrarse en los diferentes estudios serán analizadas con el detalle por el equipo de la revista, clasificándose con las referencias  correcta y cruzada. También se verificará el auto-plagio o la redundancia. La decisión final sobre la publicación o no del trabajo, en el caso de haber superado el proceso evaluador, se realizará a la vista del conjunto de todos los informes, y de forma particular a la vista de este informe de plagio. Si una vez publicado la revista tuviera noticia de un plagio no detectado procederá a la publicación de dichos datos.
—Derechos de autor: 1. Los autores/as conservarán sus derechos de autor y ceden a la revista el derecho de primera publicación de su obra. 2. Los autores/as podrán adoptar, previa solicitud a la revista, otros acuerdos de licencia no exclusiva de distribución de la versión de la obra publicada (p. ej.: depositarla en un repositorio institucional o publicarla en un volumen monográfico) siempre que se indique la publicación inicial en esta revista.
—MÉTODO DE CITA: SISTEMA DE NOTAS A PIE DE PÁGINA CONTINUAS.
- Cita: Se insertan los números en cada nota de forma consecutiva. Si hay diversas citas de un mismo recurso, se usará un número de nota para cada mención que da lugar a la cita.
- Referencia: Se presentan las notas en su orden numérico. Si una nota se refiere a un recurso ya mencionado, bien se repite la referencia completa o bien se remite a la nota anterior en la que se menciona el mismo recurso.
Ej. en el texto:
Cada sociedad, cada comunidad política, se forma su propio concepto de Constitución... [1].
En sentido jurídico la Constitución aparece como norma jurídica o, mejor, como un conjunto de normas jurídicas orienado a regular las relaciones políticas [2].
Ej. en el listado de referencias:
1. WOLKMANN, U., Elementos para una teoría de la Constitución alemana, Madrid: Marcial Pons, 2019, p. 31.
2. WOLKMANN, U., cit., p. 33 
REDACCIÓN DE REFERENCIAS EN NOTAS A PIE
Libros con un autor
NOMBRE DEL CREADOR. Título del libro (cursiva). Edición (si no es la primera edición). Lugar: Editor, Fecha de publicación
STOLLEIS, M., La textura histórica de las formas políticas, Madrid: Marcial Pons, 2011.
Libros con dos o tres autores
NOMBRE DEL (DE LOS) CREADOR (ES). Título del libro (cursiva).  Edición (si no es la primera edición). Lugar: Editor, Fecha de publicación. Para las obras con más de tres autores, se recomienda dar todos los nombres. Si se elige no mencionar a todos los autores, se dará el nombre del primer autor seguido de "y otros" o "et al."
PETERS, A., AZNAR, M.J., GUTIÉRREZ, I., La constitucionalización de la Comunidad internacional Valencia: Tirant, 2010.
Libro electrónico
RODRIGUEZ PIÑEIRO, M., CASAS BAAMONDE, M.E.,  Comentarios a la Constitución, Madrid: BOE, 2018. [consulta: 26 febrero 2019]. Disponible en: https://www.boe.es/biblioteca_juridica/publicacion.php?id=PUB-PB-2018-94&tipo=L&modo=2
Capítulo de libro
NOMBRE DEL (DE LOS) CREADOR (ES). Título de la contribución. En: NOMBRE DEL (DE LOS) CREADOR (ES) Título (cursiva). Edición (si no es la primera edición). Lugar: Editor, Fecha de publicación, Rango de páginas. Identificador normalizado (ISBN...) (si está disponible)
GUTIÉRREZ GUTIÉRREZ, I., “De la Constitución del Estado al Derecho constitucional para la comunidad internacional”, PETTERS, A., AZNAR, M.J., GUTIÉRREZ, I., La constitucionalización de la Comunidad internacional Valencia: Tirant, 2010, pp. 15 a 91.
Artículo de revista
NOMBRE DEL (DE LOS) CREADOR (ES). Título de la contribución En:  Título de la publicación seriada fuente (cursiva). Edición. Lugar: Editor, Fecha de publicación, Numeración, Rango de páginas
LUCAS VERDÚ, P., “¿Una polémica obsoleta o una cuestión recurrente? Derecho constitucional versus Derecho político”, Teoría y Realidad Constitucional nº 3,  1999, pp. 55-59.
Sitio web completo
Portal de la Constitución [consulta: 26 febrero 2018]. Disponible en: https://app.congreso.es/consti/

 
PROCESO DE EVALUACIÓN (2019) (Nota: A finales de diciembre de 2020 se publicará la relación de evaluadores correspondientes a ese año)
A lo largo de 2019, junto a los informes de los Consejos asesores y evaluadores, nacional e internacional, y a la labor previa de desbrozo del Consejo de Redacción, la revista ha tenido la oportunidad de contar con la colaboración de buen número de profesores de diferentes universidades a los que les agradecemos muy sinceramente su contribución.
Ana Aba Catoira (Universidad de A Coruña); Miguel Agudo Zamora (Universidad de Córdoba); Abraham Barrero Ortega (Universidad de Sevilla); Raúl Canosa Usera (Universidad Complutense); Luis Delgado del Rincón (Universidad de Burgos); Guillermo Escobar Roca (Universidad de Alcalá de Henares); José Julio Fernández Rodríguez (Universidad de Santiago de Compostela); Francisco Fernández Segado (Universidad Complutense); Antonio Fortes Martín (Universidad Carlos III); Enric Fossas Espadaler (Universidad Autónoma de Barcelona); Luis Alberto Gálvez Muñoz (Universidad de Murcia); Esperanza Gómez Corona (Universidad de Sevilla); Itziar Gómez Fernández (Universidad Carlos III); Luis Gordillo Pérez (Universidad de Deusto); Mercedes Iglesias Bárez (Universidad de Salamanca); Luis Jimena Quesada (Universidad de Valencia); Alberto Lopez Basaguren (Universidad del País Vasco); Javier Matia Portilla (Universidad de Valladolid); Pablo Lucas Murillo de la Cueva (Tribunal Supremo); Augusto Martín de la Vega (Universidad de Salamanca); David Ortega Gutiérrez (Universidad Rey Juan Carlos); Ana Ovejero Puente (Universidad Europea); Emilio Pajares Montolío (Universidad Carlos III); Nicolás Pérez Sola (Universidad de Jaén); Antonio Porras Nadales (Universidad de Sevilla); Miguel Revenga Sánchez (Universidad de Cádiz); María Rosa Ripollés Serrano (Universidad Complutense); Silvia Romboli (ESADE); Agustín Ruiz Robledo (Universidad de Granada); Manuel Sánchez de Diego (Universidad Complutense)¸ Susana Sánchez Ferro (Universidad Autónoma de Madrid); Soledad Santana Herrera (Universidad de Las Palmas de Gran Canaria) José Manuel Vera Santos (Universidad Rey Juan Carlos); Eduardo Vírgala Foruria (Universidad del País Vasco)
