# Supplementary material for: Peer Review in Law Journals
Source: Front Res Metr Anal. 2021 Dec 8;6:787768. doi: 10.3389/frma.2021.787768 (PMC8692876; doi:10.3389/frma.2021.787768)
Supplement: Supplementary file 3 [file DataSheet2.ZIP › DOCUMENT - 1330-0229.RTF]

INSTRUCTIONS TO AUTHORS


General information

The journal "Policija i sigurnost" (Police and Security) publishes reviewed scientific and professional papers from the field of police science and law enforcement, criminal investigation, substantive criminal law, criminal procedure law and correctional law, international public and criminal law, national security, criminology, penology, victimology, forensic medicine, forensic psychology and psychiatry and related fields (the history of the police, forensic anthropology, criminal investigation statistics etc.). The journal also publishes papers in the columns: From practice to practice, Police procedures and Court practice, Views and Opinions, Reviews and Comments and Student's papers. Before scientific and professional articles are published they are subjected to double-blind peer review process. Articles are published in Croatian and English.

The Editorial Board accepts manuscripts which have not been published and/or submitted elsewhere. The manuscript written in Microsoft Word format should be sent to the e-mail address: policijaisigurnost@mup.hr or on CD/DVD or USB stick to the following address:

Policijska akademija

Služba za razvoj policijskog obrazovanja i nakladničko-knjižničnu djelatnost

Avenija Gojka Šuška 1, 10 040 Zagreb

Manuscript preparation

A manuscript should have up to 36 000 characters with spaces. The Editorial Board reserves the right to edit the manuscript according to general rules of journal editing.

The full name of the author (or multiple authors, separated by a coma) should be printed at the left-top corner of the first page, and their research-teaching and/or teaching title, the name of the institution and their position should be written in the footnote. The title of the paper should be written in capital bald letters in the centre, and it should be concise and informative.

Furthermore, the manuscript to be reviewed, should contain an abstract in Croatian and English, not exceeding 250 words, which summarizes the contents of the article, a general review of the topic, the methodology of work, the hypothesis, etc. The abstract is followed by keywords, not more than six.

A manuscript, should contain an introduction, a body (divided into headings and subheadings) and a conclusion. The titles of the introduction, headings and conclusion should be written in capital letters, and the subheadings titles should be written in small letters. If there are pictures, graphs and other graphic illustrations within the text, the number and the caption should be written below (legend up to two lines). Footnotes are used for additional comments or explanations.


114

Instructions to authors

Polic. sigur. (Zagreb), godina 28. (2019), broj 1, str. 114 ‒ 116

Citing references

Information on citing references or quoting directly from the text is written at the end of the text, or within the text – in parentheses. The citing reference should include the author's surname, the year of publication, and if the part of the text is quoted directly from the text, also the page numbers should be mentioned. [For example, for citing a reference (Dujmović, 2003) or quoting (Modly, 1996:53).]

At the end of the manuscript there should be a list of references numbered and listed in alphabetical order of authors' names. If more than one item by a specific author is cited, they should be listed chronologically (the latest first).

Types of references are as follows:

a) to a book

The author's surname, initials. (year of publication). Title. Place of publication: Publisher.

e.g. Cormack, D. (2000). The Research Process in Nursing. Oxford: Blackwell Publishing Ltd.

b) to an article in a journal

The author's surname, initials. (Year of publication). Title of article. Title of journal, Volume number and (part number), Page numbers of contribution.

e.g. Turnbull, F. (2007). Acupuncture for Blood Pressure Lowering. Circulation, 115(24), 3048-3049.

c) to a book chapter or conference proceedings

The author's surname, initials. (Year of publication). Title of article. In: The editor's surname, initials. (editors). Title of the book or of the proceedings. Place of publication: Publisher, pages.

e.g. Josipović, I. (1996). Pravni i politički aspekti nastanka Međunarodnog kaznenog suda za bivšu Jugoslaviju. In: Šimonović, I., Vukas, B., Vukmir, B. (editors). Hrvatska i Ujedinjeni narodi. Zagreb: Organizator, 183.-196.

d) to unpublished theses

Rozenblit, J. W. (1985). A conceptual basis for model-based system design. PhD thesis. Detroit: Wayne State University.

e) to web sites

With all the above mentioned information it is necessary to write a full address of the web page and the access date, e.g. ‒ accessed 12 July 2007).

Abstract

An abstract at the end of the manuscript should be written in the 3rd person singular and translated into English. The abstract is followed by keywords also translated into English. Abstracts are not required for those articles which are not sent to peer review. Only title of the article should be translated into English.

115

Instructions to authors

Polic. sigur. (Zagreb), godina 28. (2019), broj 1, str. 114 ‒ 116

Information about the author

Manuscripts (non-returnable) and letters (which, among others, containing: the author's address, telephone number and/or e-mail address, personal identification number, giro account number and the bank's name) should be sent to the Editorial Board to the following address:

Policijska akademija, Služba za razvoj policijskog obrazovanja i nakladničko-knjižničnu djelatnost, for the journal Policija i sigurnost, Avenija Gojka Šuška 1, 10 040 Zagreb, (policijaisigurnost@mup.hr).

The Editorial Board


116
