# Supplementary material for: Peer Review in Law Journals
Source: Front Res Metr Anal. 2021 Dec 8;6:787768. doi: 10.3389/frma.2021.787768 (PMC8692876; doi:10.3389/frma.2021.787768)
Supplement: Supplementary file 3 [file DataSheet2.ZIP › DOCUMENT - 1330-0229_1.RTF]

Časopis Policija i sigurnost

ISSN 1848-428X (online) ISSN 1330-0229 (tisak)

UPUTE ZA RECENZENTE


Prije preuzimanja recenzentskog zaduženja, urednik će razmotriti nalazi li se članak unutar područja rada recenzenta te provjeriti postoji li sukob interesa. U pravilu nije prihvatljivo ako je recenzent u protekle tri godine bio sudionik istoga projekta, mentor ili na drugi način uključen u izradu rada koji se recenzira. Recenzenti su dužni upoznati urednika s bilo kakvim potencijalnim sukobom interesa u odnosu na autore ili sadržaj njihova članka koji im je upućen na recenziju. Recenzent je dužan obavijestiti urednika ako je rad za recenziranje sličan radu kojeg recenzent priprema.

Odgovornost je recenzenta da kritički ali konstruktivno procijeni članak i napiše detaljne primjedbe i savjete o istraživanju i samome članku - kako bi autorima pomogao poboljšati prikaz njihova rada. Ostale odgovornosti recenzenta uključuju postupanje s člankom kao s povjerljivim spisom te obavljanje recenzije na vrijeme. Recenzije bi trebalo dovršiti u roku tri tjedna.

Procjena članka uključuje procjenu originalnosti i važnosti istraživanja, ustroj studije, metodologiju, prikaz rezultata, snagu zaključivanja, te opću kvalitetu članka. Recenzent ne smije nikome pokazati članak bez izričite dozvole urednika.

Recenzenti trebaju biti pristojni u svojim primjedbama u članku upućenom autorima. U slučaju neprimjerenih komentara, urednici mogu takve komentare odbiti prenijeti autorima ili čak ne pokazati autorima sve recenzentove primjedbe. Recenzenti ne smiju za svoje istraživanje rabiti podatke opisane u članku koji su dobili na recenziju.

Recenzija je „dvostruko slijepa", tj. recenzent neće znati ime autora niti će autor znati ime recenzenta. Recenzenti ne bi smjeli neposredno komunicirati s autorima ili im se otkriti, osim svojim potpisom na primjedbe u okviru recenzije ili ako je drugačije dogovoreno s urednikom.

Recenzija treba biti utemeljena na pomnoj analizi članka i dobroj informiranosti recenzenta o temi i pripadajućoj joj relevantnoj literaturi. U recenziji treba uputiti i na eventualne nedostatke u tekstu članka; a poželjne su i sugestije o dopunama ili izmjenama koje bi pridonijele višoj valorizaciji članka.

Recenzija se dostavlja u Obrascu za recenziju kojeg Uredništvo dostavlja recenzentu. U Obrascu za recenziju popunjavaju se sljedeće rubrike:

0.	Vrijeme izrade recenzije

0.	Podaci o recenzentu

0.	Podaci o recenziranom rukopisu članka

0.	Mišljenje o rukopisu članka

0.	Preporuka recenzenta

0.	Zaključak i ocjena/klasifikacija rukopisa članka


Časopis Policija i sigurnost - Upute za recenzente

str. 1/2

Recenzenti sugeriraju kategoriju članka prema uputama Ministarstva znanosti i obrazovanja i članak klasificiraju prema ponuđenim kategorijama:

?	Izvorni znanstveni članak (Original scientific paper) jest originalno znanstveno djelo u kojem su izneseni novi rezultati fundamentalnih ili primijenjenih istraživanja. Članak sastavljen tako da se može na temelju danih informacija: a) reproducirati metodološki i računski postupak i dobiti rezultate s jednakom točnošću ili unutar granice stupnja slobode, kako to navodi autor; ili b) ponoviti autorova opažanja i prosuditi njegove analize; ili c) provjeriti točnost analiza i dedukcija na kojima se temelje autorovi nalazi;

?	Prethodno priopćenje (Preliminary communication) Taj znanstveni članak obvezno sadrži jednu ili više znanstvenih informacija, ali bez dovoljno pojedinosti koje bi omogućile čitatelju provjeru iznesenih znanstvenih spoznaja;

?	Izlaganje sa znanstvenog skupa (Conference paper) Može biti objavljeno samo kao cjeloviti članak koji je prethodno referiran na znanstvenome skupu, a u obliku cjelovitog

članka nije objavljeno u zborniku skupa;

?	Pregledni rad (Review article) sadrži posebni problem o kojem je već publiciran znanstveni rad, ali mu se pristupa na nov način;

?	Stručni članak (Professional paper) Sadrži korisne priloge iz struke i za struku. Sažet i kritičan pregled odabrane teme, s usmjerenjima i kontroverzama u njoj. Mora biti razumljiv i nespecijalistima predmetnoga područja. Od znanstvenog se članka razlikuje ponajprije u tome što ne donosi originalne rezultate autora istraživanja nego rabi već objavljene rezultate koje usustavljuje i objašnjava.


Ako recenzent smatra da prispjeli rad ne zadovoljava kriterije određene kategorije članka (izvorni znanstveni članak; prethodno priopćenje; pregledni znanstveni članak; izlaganje sa znanstvenog skupa; stručni članak) - recenzirani rad može predložiti za objavu u ostalim rubrikama časopisa Policija i sigurnost (Pogledi i mišljenja; Iz prakse za praksu; Policijsko postupanje i sudska praksa; Prikazi i osvrti ili dr.).

Nakon pročitanoga rada, recenzent je dužan dati svoj sud o tome treba li rad objaviti, predložiti kategorizaciju ukoliko je recenzija pozitivna - te iznijeti sud o tome treba li se u radu išta popraviti ili doraditi. Ocjena se treba kretati unutar sljedećih smjernica:

?	DA - („Prihvaća se") - bezuvjetno odobrenje za objavu rada

?	DA, POD UVJETOM DA - („Prihvaća se uz doradu") odobrenje predviđa izvjesne modifikacije/poboljšanja koja se trebaju obaviti na radu

?	NE, OSIM U SLUČAJU - („Ne prihvaća se") nužna temeljita revizija i rekonstrukcija rada

?	NE - („Ne prihvaća se") ne postoji ni minimum elemenata koji se mogu iskoristiti.


Časopis Policija i sigurnost - Upute za recenzente

str. 2/2
