# Supplementary material for: Peer Review in Law Journals
Source: Front Res Metr Anal. 2021 Dec 8;6:787768. doi: 10.3389/frma.2021.787768 (PMC8692876; doi:10.3389/frma.2021.787768)
Supplement: Supplementary file 3 [file DataSheet2.ZIP › DOCUMENT - 1330-2604.RTF]

GUIDELINES FOR AUTHORS


FOCUS AND SCOPE

Criminology and Social Integration is a journal dedicated to the interdisciplinary study of crime, behavioural problems, victimization, and social responses to deviant behaviours and crime. The journal's aim is to contribute to the scientifically based knowledge on the different forms of crime, behavioural problems, victimization, and effective social and state responses.

The journal publishes original (i.e. not previously published) scientific and professional papers in Croatian and English, as well as reviews of relevant conferences and books.

The journal is published twice a year in online format only. The whole content of the journal is in open access and available at https://hrcak.srce.hr/ksi. The journal is published by the Faculty of Education and Rehabilitation Sciences of the University of Zagreb, and its publication is co-financed by the Ministry of Science and Education of the Republic of Croatia. Authors are not charged for the costs of reviews and publication. Original scientific papers and preliminary reports written in Croatian, after acceptance for publication, are translated into English at the expense of the journal (free of charge for authors).


JOURNAL INDEXING

Criminology and Social Integration is indexed in the following bibliographic databases:

	PSYCH-INFO–AMERICAN PSYCHOLOGICAL ASSOCIATION,

	SOCIOLOGICAL ABSTRACTS,

	CRIMINAL JUSTICE ABSTRACTS (podbaza EBSCO),

	PROQUEST

	DOAJ (DIRECTORY OF OPEN ACCESS JOURNALS)

	SOCIOLOGY DATABASE

	CRIMINAL JUSTICE DATABASE

	SOCIAL SCIENCE DATABASE

	INTERNATIONAL BIBLIOGRAPHY OF THE SOCIAL SCIENCES

	HEINONLINE

	SOCINDEX


1


CATEGORIZATION OF PAPERS

Papers published in Criminology and Social Integration may be categorized as scientific (original scientific paper, preliminary report, review papers) or professional papers.

Original scientific paper

An original scientific paper contains original theoretical discussion and/or research results which have not previously been published. The research results must be reproducible and replicable. Key elements of an original scientific paper are a clearly defined and consistently described methodology as well as a critical approach regarding the research findings and limitations.

Preliminary report

This category of contribution contains results of ongoing research which are timely and topical, and published before the research process is concluded. The paper in this category puts forward new scientific data and has a clear methodological approach; the results, however, are not considered final and are not reproducible.

Review

A review contains a broad review of the current situation and trends in a particular area of study, theory or practice, but also methodological and theoretical approaches, and analysis of the current professional practice. This category of article contains both an overview of the relevant literature and the author's critical assessment of the current state of the art.

Professional paper

A professional paper primarily deals with the author's practical experiences and related issues; the article focuses on improving the practice in line with the relevant theoretical approaches and the state of the scholarly literature.


REVIEW PROCESS

Upon receipt of the paper, the editor conducts the first review step, consisting of ensuring that the manuscript is appropriate for publication (i.e. it fits thematically within the scope of the journal and satisfies the core structural criteria). The next step is a double-blind peer review process, whereby the authors and reviewers are not known to each other. We thus recommend that the authors remove any information from their manuscripts that may reveal their identity. All manuscripts are reviewed by two reviewers, and in case it is necessary, the editors may require a third review. While the authors may recommend reviewers, this decision is ultimately made by the editor. Upon receipt of reviews, the editors send the combined comments and/or recommendations to the authors, who revise their manuscript and resubmit it to the editors with an accompanying letter which explains the manner in which the reviewers' comments and recommendations were implemented, or the reasons why they

2

were not implemented. If the authors wish to appeal the review process, their complaint is to be sent to the editor. A repeated complaint is discussed by the editorial board. The editors may revise any article so that it fits the journal's standards related to formatting and language.


COPYRIGHT NOTICE

By agreeing to have their paper published in the journal, the author(s) grant to the journal the right to first publication. Once an article is accepted for publication in the journal, the copyright is owned by the publisher. The author(s) agree to have their paper used in line with the Creative Commons BY-NC-ND license, which means that their content can be redistributed with attribution for non-commercial purposes without modification and they must cite the source in an appropriate manner.

The use of measurement instruments and computer programs must be in line with valid regulations. The author(s) should attach a statement on the legal use of measurement instruments and computer programs.


ETHICAL ISSUES AND AUTHORSHIP

When it comes to original scientific contributions and prior reports, the sections that discuss methodology and the manner in which the research was conducted ought to note the ethical standards that were applied in the study (e.g. the Code of Ethics in Research Involving Children) and note the approval received by ethics commissions or similar bodies that provide approval for the study to be conducted.

In line with the guidelines by the European Association of Science Editors (EASE) 2016 (http://ease.org.uk/publications/author-guidelines), this journal recommends that the list of authors include all persons who have significantly contributed to the planning of the study, data collection and analysis, interpretation of results, writing, editing, approving its final version by assuming responsibility for all aspects of the work.

All those who have significantly contributed to the manuscript but do not satisfy the authorship criteria ought to be listed in the acknowledgments (for technical assistance, assistance in writing, general support, financial and material support). All persons listed in that part of the text must consent to being listed.

The journal does not support gift authorship, i.e. the inclusion among authors of those persons who did not take part in the research or writing, nor does it support ghost authorship, the exclusion from the list of authors of those who took part in research and writing of the article.

Alongside the paper, the author(s) must include a statement confirming that the paper has not been published nor submitted for publication in another journal, book, or similar publication. The author(s) must list any potential conflict of interest related to the paper they are submitting.

3

Ethical conduct of reviewers implies confidentiality regarding the papers they are reviewing. Reviewers are expected to report any potential conflict of interest and to exclude themselves from the review process if such a conflict of interest exists. The same is expected of editors, who must remove themselves from the editorial process if they are in a situation of conflict of interest.

In case of suspected unethical actions, the editors will proceed in line with the guidelines issued by the Committee on Publication Ethics. These guidelines are available in English at https://publicationethics.org/files/Full%20set%20of%20English%20flowcharts_9Nov2016.p df.


FORMATTING INSTRUCTIONS

The journal publishes articles in Croatian and English. The author(s) must ensure that the language of the article is appropriate for publication.

Articles are submitted via email to ksi@erf.hr.

Scientific and professional manuscripts, with all attachments, should not exceed 8000 words. The submission format is MS Word (.doc, .docx), with 1.5 spacing, Calibri size 11, margins of 2.5cm, and justified alignment. All pages must be numbered. Headers must not be used, and footnotes may only be used exceptionally.


The following structure is recommended:

	Full title of the paper (both English and Croatian language)

	Names, affiliations (department, institutions, city, state) and contact information for all authors. A corresponding author should also be noted. The editors will remove all of this information from the version of the document that is sent out for review.

	Abstract and keywords (for papers in English language, please write both into English and Croatian language). We recommend that the abstract does not exceed 200 words and that up to five keywords are included. The abstract of an original scientific article or a preliminary report should indicate research problem, aim and purpose of the study, methods, key results, and conclusions. For other types of papers, the abstract must point to the purpose of the article, methodology, key findings and conclusions. We remind the authors that the readers' willingness to read the entire article depends on the quality of the abstract.

	Introduction

	Aim and purpose of the study

	Research methodology (for empirical work)

	Results

	Discussion

4

	Research limitations (for empirical work)

	Conclusion

	Bibliography


Headings and subheadings must not be numbered. The subheadings ought to be in bold type, as well as any parts of the text that the authors wish to particularly accentuate. No parts of the text should be underlined.

Foreign-language expressions, names of documents and institutions should be italicised.


Tables, graphs, and images must be numbered and must have a title (which should be short and clear). Tables and images must be mentioned in the text and should be embedded in the text itself, near the text discussing them (i.e. they should not be added at the end of the paper nor in separate documents). Table title should be above the table, while the titles for graphs and images should be below. These titles should all be in bold type and italicised. Images must be saved in the original format, with the size and complexity adjusted to the journal format, so that all elements can be clearly visible and coloured. Decimals must be separated by full points, and zeros must be added where necessary.


The bibliography should provide complete information concerning the referenced works. Bibliographical items are listed in alphabetical order, starting with the first author's surname, and chronologically for works by the same author, starting with the earliest work. If multiple works by the same author have been published in the same year, each of these ought to be distinguished by letters (a, b, c, etc.) that follow the publication year. When referenced items have DOI numbers, these should be listed at the end of the bibliographic reference. For other work that is available online, the online source (web address) and date when the source was accessed should to be listed after the key information (title, author, etc.). If any works are directly quoted, the reference must include the page number (author last name, year, page). The journal uses the APA in-text referencing style (http://www.apastyle.org/), upon which the bibliography is based as well (more instructions can be found at the bottom of this document).


Instructions for writing reviews of books, doctoral dissertations, and conferences

The journal publishes reviews of books, conferences, and doctoral dissertations, with the limitation that they need to have been published (or defended) in the two years prior to submission. The reviews should not exceed the length of 2000 words. The introduction to the review ought to contain the information listed in the table below.


5


		BOOK REVIEW	DOCTORAL	CONFERENCE REVIEW		
			DISSERTATION REVIEW			
						
						
	First row	Book title	Dissertation title	Name of the conference		
						
	Second row	Author name and surname	Author name and	Organizer of the		
			surname	conference		
						
						
			Year in which the			
		Publisher, place of	dissertation was	The conference location		
	Third row	publication, publication	defended,			
				and dates		
		year, number of pages	faculty/department,			
						
			university, country			
						

Review author's name and surname must be listed at the end of the review.


REFERENCING

The journal uses the APA referencing style (http://www.apastyle.org/) for in-text references and the list of references. Each in-text reference must appear in the list of references, and each item from the list of references must be referenced/paraphrased in the text. The authors must ensure that the reference is identical in both locations.


APA style IN – TEXT citations

Sentences and texts from the papers of other authors can be (1) quoted indirectly or paraphrased and

(2) directly cited or quoted. Indirect quotation or paraphrase the retelling, summarizing and interpreting other people's texts, articles or books. It is also mandatory to state the reference, but it is necessary to be aware that paraphrasing faithfully displays the original work: the meaning or message of another text. Directly quoting or citing is literally transcribing or copying text from other sources. The directly quoted text is important to emphasize by putting the text under quotation marks („") and is needed to be written in italics.


Reference should be written in the text rather than in the notes (footnotes). References in the text can be cited in two ways:

1)  Author (year of publication)

Example: …was developed and validated by Brown and Clasen (1985).


2)  (Author, year of publication)

6

Example: The Peer Pressure Inventory (PPI) is one of the most well-validated measures of peer pressure (Brown and Clasen, 1985).


When the quoted paper has one or two authors, citing references throughout the text includes the surname of the cited author and the year of publishing the publication.

Example:
?	Chaffin (2008) or (Chaffin, 2008)

?	Minner i Muns (2005) or (Minner i Muns, 2005)


When the quoted paper has three, four, five or six authors, for the first cite all the author's surnames and year should be listed. Further in-text cites can be shorted to the first author's name followed by

„et al." and the year of publication.

Example:

	FIRST CITATION IN TEXT	SUBSEQUENT CITATIONS	
			
Work by three	Alarid, Sims and Ruiz (2011)	Alarid et al. (2011)	
authors	(Alarid, Sims and Ruiz, 2011)	(Alarid et al., 2011)	
			
Work by four	Rettenberger, Matthes, Boer and Eher (2010)	Rettenberger et al. (2010)	
authors	(Rettenberger, Matthes, Boer and Eher, 2010)	(Rettenberger et al., 2010)	
			
Work by five	Wanklyn, Ward, Cormier, Day and Newman (2012)	Wanklyn et al. (2012)	
authors	(Wanklyn, Ward, Cormier, Day and Newman, 2012)	(Wanklyn et al., 2012)	
			


?	When the quoted paper has more than six authors, and the paper is not published within an organization, association or corporation, then only the first author's surname should be stated

followed by „et al." and the year of publication.


If the reference cited is the work of a group of authors within a governmental organization, corporation or association, the full name of the institution that published the paper should be written when citing the reference in the text.

Example:
?	University of Pittsburgh (2005)

?	(University of Pittsburgh, 2005)


If quoted paper presents the work of a group of authors within institutions that are publicly

7

recognizable by their abbreviation, the full name and abbreviation and the year of publication should be written at the first in-text citing. Further in-text cites can be shorted to the abbreviation and the year of publishing.

Example:
➢ Council of Europe [COE] (2017)
First citation in text
?	(Council of Europe [COE], 2017)

?	COE (2017)
Subsequent citations
➢ (COE, 2017)


In the case of direct citation, the page number of the quoted text should be written after the authors surname and the year of publication.

Example: According to Orton and Weick (1990, p. 203–204), loose coupling is a „situation in which elements are responsive, but retain evidence of separateness and identity . . . [the] elements affect each other suddenly (rather than continuously), occasionally (rather than constantly), negligibly (rather than significantly), indirectly (rather than directly), and eventually (rather than immediately) ".


If some information in the text is supported by a number of different references by different authors, they are listed in alphabetical order as follows:

Example: In some studies (Kupchik and Ward, 2010; Orton and Weick, 1990), authors argues about…


If some information in the text is supported by a number of different references by the same author, they are listed as follows:

Example: A number of studies (University of Maribor, 2010, 2011, 2013) emphasize the importance of…


Two or more papers from the same author published in the same year are indicated in small letters a, b, c, etc. after the reference year.

Example: A number of studies (Chaffin, 2010a, 2010b; Lock, 2015) emphasize the importance of…


The use of "secondary references" is quoted in a way that the original author and year of publication is quoted first, and then "as cited in" is added and the reference (author's surname and year) in which this information is found.

Example: Meta-analytic study (Andrews and Dowden, 2006; as cited in Lock, 2015) consistently…

If the text uses a reference to a legal act that has numerous amendments, it is sufficient to write the

8

number of the Official Gazette indicating when the law was adopted, and the last number indicating the last amendment.

Example: Criminal Procedure Act (OG 152/08, 126/19)


APA STYLE – REFERENCE LIST

A list of all references used in the text is provided at the end of the paper. The list contains the full bibliographic information about the references that the author used in text in short form.

References are listed in alphabetical order by last name of the first author. The works of the same author are cited in the chronological order of their publication, and the works of the same author and the same year of publication are listed in the order of appearance in the text.

BOOKS

*	Author's surname, Initial(s) of the name. (Year of publishing). Title: subtitle (information about the edition of the book). Place of publishing: Publisher.

*	If the book is available online, at the end of the quote, should be written „Retrieved from the: link to the page from which the book was downloaded (date of access to the web page)".

*	If the book has its own DOI number, at the end of the quote should be written „doi: (doi of the book)".

Examples:
?	William Edwin, T. (2010). Juvenile delinquency: a sociological approach. Boston: Allyn & Bacon.


?	Taylor, R.W. & Fritsch, E.J. (2011). Juvenile Justice: Policies, Programsand Practices (Third Edition). New York: The McGraw – Hill Companies, Inc.


CHAPTER IN THE BOOK

*	Author's surname, Initial(s) of the name. (Year of publishing). Title: Subtitle. IN: editor initial(s), surname (Edt.) Book title: subtitle (chapter page range). Place of publishing: Publisher.

*	If the book is available online, at the end of the quote, should be written „Retrieved from the: link to the page from which the book was downloaded (date of access to the web page)".

*	If the book has its own DOI number, at the end of the quote should be written „doi: (doi of the book)".


Examples:

?	Fisher, H. E. (2014). The Tyranny of Love: Love Addiction – An Anthropologist's View. U K. P. Rosenberg and L. C. Feder (edt.), Behavioral Addictions: Criteria, Evidence and Treatment (237 –

9

266). Oxford: Elsevier.


PAPER FROM PROCEEDINGS

*	Author's surname, Initial(s) of the name. (Year of publishing). Title: Subtitle. IN: editor initial(s),

surname (Edt.) Title: subtitle: Vol.xx. (chapter page range). Place of publishing: Publisher.

Example:
?	Šegvić, S. (2011). Schengen Regime for Administering EU external borders. U A. Bačić (edt.),

Collected papers of the Law Faculty of the University of Split: Vol.48 (str.11 – 33). Split, Law Faculty of the University of Split.


JOURNAL ARTICLES

*	Author's surname, Initial(s) of the name. (Year of publication). Article title: subtitle. Journal Title, Volume number (issue number), article page range.

*	If the article has its own DOI number, at the end of the quote should be written „doi: (doi of

the article)".

Examples:
?	Brogan, L. (2015). Applying the Risk – Needs – Responsivity (RNR) Model to Juvenile Justice.

Criminal Justice Review, 40(3), 277 – 302.

?	Andrews, D.A., Bonta, J., Wormith, J.S. (2011). Does Adding the Good Lives Model Contribute to Effective Crime Prevention?. Criminal Justice and Behavior, 38(7), 735 – 755.

?	Edelstein, A. (2016). Rethinking Conceptual Definitions of the Criminal Career and Serial Criminality. Trauma, Violence & Abuse, 17(1), 62 – 71. doi: 10.1177/1524838014566694.?	


PUBLISHED PAPER FROM THE CONFERENCES

*	Author's surname, Initial(s) of the name. (Year of publishing). Title: Subtitle. IN: editor initial(s), surname (Edt.) Title: subtitle: Vol.xx. Title of the conference (chapter page range). Place of publishing: Publisher.


GRADUATE AND MASTER THESIS, DOCTORAL THESIS

*	Author's surname, Initial(s) of the name. (Year of publishing). Title: Subtitle (Type of the paper). Institution, Place.

10


LEGAL ACTS

*	The name of the legal act. The title of the publication in which the legal act was published, number.


NEWSPAPER ARTICLES

*	Author(s) surname, initial(s) of the name. (Year, Month of publishing). Title of article: subtitle. Title of Newspaper, column/section, page range. Retrieved from URL*


11
