# Supplementary material for: Peer Review in Law Journals
Source: Front Res Metr Anal. 2021 Dec 8;6:787768. doi: 10.3389/frma.2021.787768 (PMC8692876; doi:10.3389/frma.2021.787768)
Supplement: Supplementary file 3 [file DataSheet2.ZIP › DOCUMENT - 1330-2604_1.RTF]

Journal Criminology and Social Integration 
Title of the manuscript:
Report form

Please fill in the table below and answer all the questions. Please give your considered opinion on the manuscipt that you are asked to review in the commentary below the table. In doing so, please pay attention to the quality, originality, significance and relevance of the research topic. In addition, please take into account if the topic is new and under-reserached as well as whether the topic corresponds to the profile of our journal/reader.
At the end of the review please give your personal recommendation including the type/category of the paper, your opinion on acceptability of the manuscript for publishing in our journal, and recommended revision. If you recommend revision, please indicate whether you want to see the revised version or whether you leave it to the editors to decide on its acceptability or whether you reject the manuscript with no possibility for re-submission.
Please answer the questions below: 
Adequacy of the reserach topic	Yes 	No	
Is the manuscript's topic relevant to our journal? 			
Is the research original?			
Does the reserach make a new and/or useful contribution important for the development of its field of study?			
Are the reserach goals celarly stated? 			
Are the reserach questions adequate to the topic? 			

Adequacy of the research methods	Yes	No	
Do(es) the author(s) use adequate research methodology? 			
Is the research process adequately descibed? 			
Are the research ethics respected? 			

Adequacy of the results description	Yes	No	
Are the results adequatly presented and concluded? 			
Is the relevant discussion of the results conducted? 			
Are the conclusions of the research stressed?			
Do(es) the author(s) reflect on the reserach and its results?			
Is it clear how the results may be used in practice? 			


Adequacy of the structure of the paper and writting  style 	Yes 	No	
Does the paper have the structure which is appropriate for its type/category?			
Is the language of the text understandable and readable? 			
Is the summary informative in relation to the type of paper? 			
Is the list of key words appropriate and satisfactory with respect to the topic?			

Reviewer’ comment(s).


Recommended manuscript category:
`)	original scientific paper   ⎕
`)	preliminary report                 ⎕
`)	review paper                       ⎕
`)	professional paper            ⎕
`)	other ________________          ⎕

Reviewer’s final recommendation:
Accept without revision ⎕
Accept with revision  ⎕  (If yes, please describe the type and extent (minor, major) of the revision. If you recommend revision, please indicate whether you want to see the revised version of the manuscript.)
Reject ⎕
