# Supplementary material for: Peer Review in Law Journals
Source: Front Res Metr Anal. 2021 Dec 8;6:787768. doi: 10.3389/frma.2021.787768 (PMC8692876; doi:10.3389/frma.2021.787768)
Supplement: Supplementary file 3 [file DataSheet2.ZIP › DOCUMENT - 1330-2965.RTF]

 
 
·	Naslovna·	 
·	O časopisu·	 
·	Prijava·	 
·	Registracija·	 
·	Pretraživanje·	 
·	Trenutni broj·	 
·	Arhiva·	 
·	Obavijesti·	 
Open Journal Systems 
Pomoć 
Korisnik 
Korisničko ime		
Lozinka		
 Zapamti me	
	

Jezik / Language 

Sadržaj časopisa Pretraživanje 
	
	
	


Pregled 
·	Po broju časopisa·	 
·	Po autoru·	 
·	Po naslovu·	 
Veličina znakova   
Informacije 
·	Za čitatelje·	 
·	Za autore·	 
·	Za knjižničare·	 
Naslovna > O časopisu > Uređivačka politika 
Uređivačka politika
 
·	» Fokus i područje djelovanja·	 
·	» Rubrike·	 
·	» Recenzijski postupak·	 
·	» Učestalost izdavanja·	 
·	» Otvoreni pristup·	 
·	» Indeksiranje i referiranje·	 
·	» Cijena i pretplata·	 
·	» Troškovi zaprimanja i objavljivanja priloga·	 
·	» Uvjeti za korištenje sadržaja časopisa·	 
·	» Etički kodeks uredništva·	 
Fokus i područje djelovanja
 
Revija za socijalnu politiku je časopis koji tematizira široki raspon pitanja socijalne politike. Stoga se u njemu objavljuju tekstovi o mirovinskim, zdravstvenim, obiteljskim, stambenim, obrazovnim politikama, pitanjima rada, nezaposlenosti, siromaštva, socijalne pomoći te o svim drugim socijalnim problemima i aktualnim društvenim procesima. Uz izvorne radove u časopisu se redovito objavljuju i prevedeni tekstovi, različiti dokumenti, statistički podaci, informacije, prikazi, recenzije i osvrti.
 
Rubrike
 
Članci
 
Uredništvo prima prethodno neobjavljene priloge na hrvatskom ili engleskom jeziku. Rad zajedno s popisom literature i svim prilozima ne bi smio biti opsežniji od 50 000 znakova. Radu se prilaže sažetak na hrvatskom i engleskom jeziku, opsega do tisuću znakova te kratak popis ključnih riječi.
Otvoreno za prijave	Katalogizirano	Recenzijski postupak	
Dokument
 
Revija za socijalnu politiku redovno objavljuje selekciju 'hrvatskih i međunarodnih dokumenata relevantnih za područje socijalne politike.
Otvoreno za prijave	Katalogizirano	Recenzijski postupak	
Prijevod
 
Revija za socijalnu politiku redovito prevodi na hrvatski jezik posebno značajne ili zanimljive radove renomiranih inozemnih autora izvorno objavljenih na stranom jeziku.
Otvoreno za prijave	Katalogizirano	Recenzijski postupak	
Regulativa
 
Časopis redovito pruža pregled izmjena u zakonskoj regulativi relevantnih za područje socijalne politike u Hrvatskoj.
Otvoreno za prijave	Katalogizirano	Recenzijski postupak	
Knjige i časopisi
 
Časopis prihvaća prikaze hrvatskih i stranih djela ne starijih od tri godine. Poželjan opseg informacija, prikaza i recenzija iznosi od pet do devet tisuća znakova. Prikazuju se domaća i strana djela ne starija od tri godine. Na početku teksta se navode svi podaci prikazanog djela ili časopisa, dok se autor potpisuje na kraju rada.
Otvoreno za prijave	Katalogizirano	Recenzijski postupak	
Informacije i osvrti
 
Poželjan opseg informacija i osvrta na događanja iznosi od pet do devet tisuća znakova. Recenziraju se domaća i strana djela ne starija od tri godine. Na početku teksta se navode svi podaci prikazanog skupa, rasprave ili dokumenta dok se autor potpisuje na kraju rada.
Otvoreno za prijave	Katalogizirano	Recenzijski postupak	
Dokumentacija
 
Svaki broj Revije za socijalnu politiku donosi pregled pokazatelja i kretanja za određeno područje socijalne politike.
Otvoreno za prijave	Katalogizirano	Recenzijski postupak	
 
Recenzijski postupak
 
Svi radovi poslani Reviji za socijalnu politiku prolaze kroz recenzijski postupak u kojemu dva anonimna recenzenta procjenjuju rad u anonimiziranom obliku. Uredništvo će autore u primjerenom roku izvijestiti o rezultatima recenzijskog i uredničkog postupka. Uredništvo pridržava pravo prilagodbe rada uredničkim propozicijama i standardima hrvatskoga književnog jezika.

Prije preuzimanja recenzentskog zaduženja, recenzent treba razmotriti nalazi li se članak unutar njegovog područja ekspertize te provjerite postoji li sukob interesa. U pravilu nije prihvatljivo da recenzent bude sudionik istog projekta, mentor, ili na drugi način uključen u izradu rada koji se recenzira. Prihvaćanjem recenzije recenzent potvrđuje da ne postoji nikakav sukob interesa između recenzentske i ostalih profesionalnih ili osobnih uloga.

Okvirna struktura recenzije:
• Obujam i struktura rada 
Recenzent treba ukratko opisati rad na jasan i pregledan način. Svakako se treba osvrnuti na logičku strukturu iznesene građe te stilsku i sadržajnu dorađenost članka.
• Opće napomene
Recenzent treba procijeniti metodološku i teorijsko-konceptualnu utemeljenost članka. Također, ovdje se valja osvrnuti na utemeljenost i uvjerljivost iznesene argumentacije (na primjer povezanost nalaza i njihovih tumačenja), naglašavajući jake i problematične točke.
• Posebne napomene
Slično općim napomenama, recenzent može pružiti i posebne napomene koje se tiču pojedinih dijelova članka, posebno po pitanju mogućih pogrešaka.
• Ocjena rada i zaključak
Pri procjeni kvalitete rada, recenzent treba imati na umu njegovu izvornost i znanstveni doprinos za razumijevanje teme koju oslovljava, ali i relevantnost za područje socijalne politike.
• Preporuke za objavljivanje
Zaključno, recenzent treba predložiti objavljivanje, doradu, odnosno neprihvaćanje članka prema sljedećem popisu (odluka se evidentira i preko web-sučelja):
1. Tekst se može objaviti bez izmjena 
2. Tekst se može objaviti uz izmjene predložene u recenziji 
3. Tekst vratiti na recenziju nakon izvršenih izmjena predloženih u recenziji 
4. Tekst predložiti za objavljivanje u nekom drugom časopisu
5. Tekst nije za objavljivanje

Ukoliko je recenzija pozitivna, članak valja kategorizirati prema sljedećim kriterijima: 
1. Izvorni znanstveni članak 
• sadrži neobjavljene rezultate izvornih istraživanja u potpunom obliku. U ovu se skupinu članaka ubrajaju i sustavni pregledi i meta-analize.
2. Prethodno priopćenje 
• sadrži neobjavljene rezultate izvornih istraživanja u preliminarnom obliku.
3. Pregledni članak
• sadrži izvoran, sažet i kritički prikaz jednog područja ili njegova dijela, u kojemu autor i sam aktivno sudjeluje. Mora biti naglašena uloga autorova izvornog doprinosa u tom području u odnosu na već objavljene radove, kao i pregled tih radova.
4. Stručni članak 
• sadrži korisne priloge iz struke i za struku, ali ne mora predstavljati izvorna istraživanja. 

Poželjni rok za izradu recenzije je dva tjedna. Duljina teksta recenzije te opsežnost komentara i kritika prepuštena je procjeni recenzenta. Recenzenti mogu priložiti i komentiranu verziju izvornog teksta, koja može biti stavljena na uvid autorima.

Recenzijski postupak je dvostrano anoniman – identitet autora ostaje nepoznat do eventualnog objavljivanja teksta, dok se identitet recenzenata pojedinog teksta otkriva samo na recenzentov zahtjev. Na kraju svakog godišta časopis objavljuje popis svih recenzenata dotične godine.
 
Učestalost izdavanja
 
Časopis izlazi tri puta godišnje: u ožujku, srpnju i studenom.
 
Otvoreni pristup
 
Ovo je otvoreno dostupni časopis, što znači da su svi sadržaji časopisa dostupni korisnicima na javnom internetu za čitanje, preuzimanje, umnožavanje, distribuiranje, tiskanje, pretraživanje ili korištenje u bilo koju drugu zakonitu svrhu, bez ikakvih financijskih, pravnih ili tehničkih prepreka, sve dok se sadržaji citiraju na ispravan način.
 
Indeksiranje i referiranje
 
Prilozi objavljeni u časopisu indeksiraju se u:

 Sociological Abstracts, Social Services Abstracts, Worldwide Political Science Abstracts (Cambridge Scientific Abstracts, San Diego, USA),  Social Care Online (Social Care Institute for Excellence, London, UK),  International Bibliography of the Social Sciences (ProQuest, Cambridge, UK), FRANCIS (INIST-CNRS, Vandoeuvre-les-Nancy, France), SCOPUS, GEOBASE (Elsevier, Amsterdam, Netherlands), SocINDEX (EBSCO, Ipswich, USA), ERIH PLUS (Norwegian Social Science Data Services - NSD), Directory of Open Access Journals (Lund University, Sweden), Social Science Citation Index, Social Scisearch, Journal Citation Reports/Social Sciences Edition  (Thomson Scientific, Philadelphia, USA)
 
Cijena i pretplata
 
Pristup on-line izdanju časopisa besplatno je, ali moguće je pretplatiti se na tiskano izdanje Revije za socijalnu politiku ili kupiti pojedine tiskane primjerke.

Cijena broja za pojedince iznosi 30 kuna, a za ustanove 50 kuna. Godišnja Pretplata na časopis iznosi 80 kuna, a za ustanove 120 kuna. Popust na kupovinu starih godišta časopisa iznosi 30%

Za detaljnije informacije o pretplati pošaljite e-poštu na kontakt-adresu časopisa ili nazovite tajništvo na 01-4895-829
 
Troškovi zaprimanja i objavljivanja priloga
 
Časopis ne naplaćuje nikakve troškove autorima prilikom zaprimanja ili objavljivanja članaka ili drugih priloga.
 
Uvjeti za korištenje sadržaja časopisa
 
Svi sadržaji časopisa na javnom internetu dostupni su besplatno u nekomercijalne svrhe. Korisnici smiju umnožavati i distribuirati materijale, preoblikovati ih i prerađivati sve dok se sadržaji citiraju na ispravan način.
 
Etički kodeks uredništva
 
Etički kodeks uredništva časopisa Revije za socijalnu politiku temelji se na dokumentima, preporukama i smjernicama Odbora za izdavačku etiku (COPE-a - Committee on Publication Ethics). 
Uredništvo će štititi slobodu znanstvenog i stručnog izražavanja te voditi računa o znanstvenim, stručnim i profesionalnim potrebama čitatelja, autora i šire javnosti. Uredništvo će kontinuirano djelovati u smjeru podizanja razine kvalitete časopisa, što uključuje i jasne procedure u ocjeni radova koji se žele objavljivati. Kako je provjerljivost ključna odrednica znanstvene spoznaje, uredništvo će voditi računa o tome da autori u sklopu objavljenih sadržaja članovima znanstvene zajednice osiguraju dostupne metodološke i druge informacije korištene pri analizi, a koje su nužne za proces provjere objavljenog sadržaja. U tom pogledu uredništvo podržava i prihvaća politiku objavljivanja ispravaka, pojašnjenja ili povlačenja spornih radova objavljenih u časopisu. 
U svim slučajevima eventualnog neetičnoga ili neprofesionalnog djelovanja, uredništvo će žurno reagirati, prateći upute i etički postupnik COPE-a. Uredništvo će sve optužbe uzeti u razmatranje, tražiti odgovore i pojašnjenja od autora/ice ili drugih relevantnih dionika. Ako se dokaže neprofesionalno i neetično djelovanje, uredništvo će kroz časopis i/ili preko mrežne stranice časopisa ukazati na takvo djelovanje, kako se ono ne bi ponovilo. 
Autori su obavezni informirati čitatelje i javnost o financijskim izvorima te o drugim relevantnim aspektima istraživanja u sklopu kojih su nastali radovi objavljeni u časopisu, kako bi se osigurala objektivnost rezultata i interpretacija i otklonili mogući sukobi interesa. Uredništvo će posebnu pozornost posvetiti postojanju takvih informacija te njihovoj prezentaciji unutar objavljenog sadržaja. Uredništvo će i od recenzenata zatražiti da iskažu potencijalni sukob interesa koji bi mogao utjecati na njihovu ocjenu znanstvenoga rada kojeg recenziraju (u pravilu nije prihvatljivo da recenzent bude sudionik istog projekta, mentor ili na drugi način uključen u izradu rada koji se recenzira). K tome, uredništvo je razvilo i postupke za upravljanje vlastitim sukobom interesa. Tako se glavni urednik izuzima iz donošenja odluka u slučajevima kada sam ili njegovi suradnici objavljuju radove u časopisu, već odluku donosi izvršni urednik ili raniji glavni urednik ako je član uredništva. 
Također, uredništvo se obvezuje na poštivanje svih zakonskih, podzakonskih i drugih propisa vezanih uz zaštitu podataka prikupljenih tijekom istraživačkog procesa, na kojima se temelji znanstveni rad. Uredništvo će voditi računa da su autori/ice u istraživačkom procesu slijedili upute o etičkom postupanju te osigurali pristanak nadležnih institucija. Ukoliko se ocijeni potrebitim, uredništvo može zatražiti od autora da potvrde jesu li dobili pristanak nadležnih institucija ili da pojasne kako su razriješili moguće etičke dileme. 
Uredništvo će posebno voditi računa da radovi koji su uspješno prošli recenzentski postupak budu objavljeni u primjerenom vremenu, u pravilu u jednom od tri naredna broja časopisa. To podrazumijeva i činjenicu da će u slučaju promjene uredništva, novo uredništvo poštovati odluke ranijeg uredništva vezano za redoslijed objavljivanja članaka koji su uspješno prošli recenzentski postupak. Informacije o načinu provođenja recenzentskog postupka javno su dostupne na mrežnoj stranici časopisa. Recenzentski postupak je dvostrano slijep (tekstovi su anonimizirani, a identiteti recenzenata nisu poznati autorima).


Revija za socijalnu politiku (Online). ISSN: 1845-6014 
