# Supplementary material for: Peer Review in Law Journals
Source: Front Res Metr Anal. 2021 Dec 8;6:787768. doi: 10.3389/frma.2021.787768 (PMC8692876; doi:10.3389/frma.2021.787768)
Supplement: Supplementary file 3 [file DataSheet2.ZIP › DOCUMENT - 1330-349X.RTF]

Instructions for authors

llected Papers of the Faculty of Law University of Rijeka publishes scientific and professional papers. The main aim of the Journal is to improve scientific communication in the area of law and contribute to the development of legal science in Croatia, European Union and academic comunity at large. Jorunal publishes papers in the area of law and related social sciences if they thematically contribute to the area of law. Papers in the journal are published in Croatian, English, Italian and German. Papers in other languages can be taken into consideration for publishing.


The Editorial Board strictly accepts unpublished manuscripts and manuscripts that are not in consideration before another journal.


Submitted papers should not exceed more than 32 text cards (1 text card equals 1800 characters with spaces, which includes footnotes) i.e. 60.000 characters including summary, keywords and bibliography. The paper should have bibliography, summary in English and in the original language of the paper. If the paper is written in language other than Croatian, author is obliged to provide official confirmation that the text is proof – read (after the paper has been accepted for publishing).

Together with the title of the paper, the authors should submit their name, family name and title, name and address of the institution as well as their e-mail address.


The summary should encompass no more than 200 words and should inform about the purpose of the paper, methodology, the most important results and conclusion. The summary should state the name and family name of the author, name of the institution, title of the article, no more than five key words and a denotation "Summary".


The text must be written in the font Times New Roman, size 12, spacing 1,5. Footnotes should be quoted with continuous numbering at the bottom of the text as they appear in the text. They must be written in the font Times New Roman, size 10, line spacing single. Pages of the paper should be numbered.


The paper which is submitted for publication in the Collected Papers of the Faculty of Law University of Rijeka must be written and structured according to the Chicago Manual of Style (CMS).


All papers will be subjected to an anonymous review procedure. Due to paper anonymity, which is ensured by removing the author's name, it is unacceptable to submit a paper written in a way revealing the author's identity by citations of his own previous written works.


The Editorial Board holds the right to editorially adjust the paper to the journal's propositions and to language-edit written works in Croatian language according to the standards of the Croatian standard language.


Book reviews, comments and surveys are not subjected to review. They should not exceed three typed text cards. Authors are expected to sign them before submitting.
