# Supplementary material for: Peer Review in Law Journals
Source: Front Res Metr Anal. 2021 Dec 8;6:787768. doi: 10.3389/frma.2021.787768 (PMC8692876; doi:10.3389/frma.2021.787768)
Supplement: Supplementary file 3 [file DataSheet2.ZIP › DOCUMENT - 1330-5476.RTF]

       NOVOSTI
 
  Časopis "Pravo u gospodarstvu" 
Èasopis za gospodarsko-pravnu teoriju i praksu 
ÈASOPIS je va¾an i koristan alat svim pravnicima i onima koji se tako osjeæaju!
Redovito izlazi od 1. listopada 1962., kada je bio prvo glasilo te vrste u ondanjoj dr¾avi. U poèetku  prvenstveno glasnik pravnika u gospodarstvu, vremenom je postao nezaobilazno struèno tivo za sve pravnike, u gospodarstvu i/ili vezane uz gospodarstvo, kao i one u sudovima, upravi. Specijalistièke kaznenopravne teme u èasopisu rijetke su, osim onih od opæeg znaèaja.
Savez veæ preko pola stoljeæa, od 1962, kao i Èasopis, redovito organizira proljetno savjetovanje u Opatiji  SUSRET PRAVNIKA. 
 

Upute autorima 
  
Èasopis Pravo u gospodarstvu objavljuje radove iz podruèja pravnih znanosti i struke. Urednitvo prima ponajprije neobjavljene radove. Autori zadr¾avaju autorska prava za èlanke objavljene u èasopisu, a èasopisu daju pravo objavljivanja, kako u tiskanom, tako i u elektronièkom obliku. Radove prihvaæene ili objavljene u Èasopisu autor smije objaviti u drugoj publikaciji uz suglasnost Urednitva, te naznaku prethodne objave u Èasopisu.
Radovi se dostavljaju u Microsoft Word formatu elektronièkom potom. Uz naslov rada treba navesti ime i prezime autora, struèni/znanstveni stupanj i mjesto autorova zaposlenja (koje se na zahtjev mo¾e u objavi izostaviti). Uz recenziju, radovi se razvrstavaju u sljedeæe kategorije:  
1) izvorni znanstveni rad - odlikuje se izvornoæu zakljuèaka, ili iznosi prethodno neobjavljene izvorne rezultate znanstveno koncipiranog i provedenog istra¾ivanja;  
2) pregledni znanstveni rad - sadr¾i temeljit i obuhvatan kritièki pregled problematike, bez znaèajnije izvornosti rezultata;
3) prethodno znanstveno priopæenje - rad koji sadr¾i prve rezultate istra¾ivanja u tijeku, koji poradi aktualnosti zahtijevaju brzo objavljivanje, bez razine obuhvatnosti i utemeljenosti znanstvenog rada, te
4) struèni rad - rad koji sadr¾i znanja i iskustva relevantna za odreðenu struku, ali nema obilje¾ja znanstvenosti.
  
Preporuèeni sinopsis od najvie deset redaka, popis (do pet) kljuènih rijeèi.
Uz tekst se navode biljeke kojima se dopunjuje tekst te daju cjeloviti podaci o koritenoj literaturi, koja se ne objavljuje zasebno.
Prikazi knjiga, osvrti i ocjene ne podlije¾u recenziji, a u pravilu ne dulji od osam kartica.
Urednitvo pridr¾ava pravo prilagodbe rada opæim pravilima ureðivanja èasopisa i standardu hrvatskog jezika. Rukopisi se ne vraæaju.
 

Urednikova rijeè uz PUG 4/21 
Cijenjeni pretplatnici i svi èitatelji,

Proljeæe nam protjeèe u nestrpljivom ièekivanju znaèajnijih najava 'starog normalnog'. To se odrazilo i na organizaciji i pripremi naega ovogodinjeg Susreta pravnika, posljednjeg pred jubilarno ezdeseto Susretanje, pa i na neto ranijoj publikaciji broja Èasopisa koji sadr¾i (i) prispjela priopæenja uz 59. Susret. 
U broj je unesen i prilog najavljen u programu Susreta kao dodatak referatu s ovrnopravnom temom.
Uz organizacijske 'teme i dileme', velik je teret u pripremi dviju publikacija  Zbornika Susreta i ovog broja Èasopisa  i na Sveuèilinoj tiskari, s obzirom na last minute prispijevanje nekih priloga. No, ni seizmièke ni infekcijske smetnje ne uspijevaju nas zaustaviti u ostvarivanju pothvata.
Nadamo se i uvjereni smo da æe sudionici ovoga 'proljetnog' Susreta, koji je pred nama  na samom poèetku kalendarskog ljeta, ali i ostali èitatelji pripremljenih publikacija, naæi zanimljive i korisne materijale, najprije u Zborniku, a zatim i u tekstovima u Èasopisu. 
U realizaciji Susreta trudit æemo se da ne posrnemo i ne skljokamo se, da ne bismo doli na popis antologijskih nezgoda, koji je ipak rezerviran za velikane.

Do susreta na SUSRETU, ovako ili onako.
Va Urednik.
 
 
 
Skinite narud¾benicu za pretplatu na èasopis "Pravo u gospodarstvu".
Ako na raèunalu nemate instaliran Acrobat Reader, mo¾ete ga preuzeti sa ovih stranica.
 

Sadržaji brojeva časopisa od 2000. godine 
2000 / 12000 / 22000 / 32000 / 42000 / 52000 / 62001 / 12001 / 22001 / 32001 / 42001 / 52001 / 62002 / 12002 / 22002 / 32002 / 42002 / 52002 / 62003 / 12003 / 22003 / 32003 / 42003 / 52003 / 62004 / 12004 / 22004 / 32004 / 42004 / 52004 / 62005 / 12005 / 22005 / 32005 / 42005 / 52005 / 62006 / 12006 / 22006 / 32006 / 42006 / 52006 / 62007 / 12007 / 22007 / 32007 / 42007 / 52007 / 62008 / 12008 / 22008 / 32008 / 42008 / 52008 / 062009 / 012009 / 022009 / 032009 / 042009 / 052009 / 062010 / 012010 / 022010 / 032010 / 042010 / 052010 / 062011 / 012011 / 022011 / 032011 / 042011 / 052011 / 062012 / 012012 / 022012 / 032012 / 042012 / 052012 / 062013 / 012013 / 022013 / 032013 / 042013 / 052013 / 062014 / 012014 / 022014 / 032014 / 042014 / 052014 / 062015 / 012015 / 022015 / 032015 / 042015 / 052015 / 062016 / 012016 / 022016 / 032016 / 042016 / 052016 / 062017 / 012017 / 022017 / 032017 / 042017 / 052017 / 062018 / 012018 / 022018 / 032018 / 042018 / 052018 / 062019 / 012019 / 022019 / 032019 / 04 2019 / 052019 / 062020 / 012020 / 022020 / 032020 / 042020 / 052020 / 062021 / 012021 / 022021 / 032021 / 04	
 
·	Sa¾etci èasopisa: 
o	2008 / 6o	 
o	2009 / 1o	 
Impressum
 
Izdavač:
Hrvatski savez udruga pravnika u gospodarstvu
MBS 3283585 Zagreb, Križanićeva 16/IV, tel/fax: (01) 4614 890
Glavni urednik:
mr. sc. Miljenko Giunio, Zagreb / Zavala
Urednici:
prof. dr. sc. Marko Baretić, Zagreb
dipl. pravnik Ivica Crnić, Zagreb
prof. dr. sc. Hrvoje Markovinoviæ
prof. dr. sc. Petar Miladin, Zagreb
- iz inozemstva (dopisni):
prof. dr. sc. Tomislav Borić (Pravni fakultet Sveučilita u Grazu),
prof. dr. sc. ime Ivanjko (Pravni fakultet Sveučilita u Mariboru)
Nakladničko Vijeće:
Akademik Jaka Barbić / Zagreb
prof. dr. sc. Petar Klarić / Zagreb
prof. dr. sc. Mihajlo Dika / Zagreb
prof. dr. sc. Dragan Bolanča / Split
prof. dr. sc. Jozo Čizmić / Split
prof. dr. sc. Edita Čulinović-Herc / Rijeka
prof. dr. sc. Damir Klasiček / Osijek
dr. sc. Srđan imac / Zagreb
Prijevod sažetka na engleski:
Sandra Obuljen, dipl. iur.
Žiro-račun:
2360000-1101452394 kod Zagrebačke Banke d.d., Zagreb
IBAN:
HR3523600001101452394
OIB:
94225628059 
Časopis izlazi 6 puta godinje
Pretplata:
700,00 Kn + PDV, za inozemstvo 1.400,00 Kn
Tisak:
 Sveučilina tiskara d.o.o., Zagreb, Trg Republike Hrvatske 14.
 
© Hrvatski savez udruga pravnika u gospodarstvu | web by: seemplee	
