# Supplementary material for: Peer Review in Law Journals
Source: Front Res Metr Anal. 2021 Dec 8;6:787768. doi: 10.3389/frma.2021.787768 (PMC8692876; doi:10.3389/frma.2021.787768)
Supplement: Supplementary file 3 [file DataSheet2.ZIP › DOCUMENT - 1331-1182.RTF]

Instructions for authors

Refereeing policy and submission of papers

All received papers will be subject to a double-blind peer refereeing process. A minimum of two independent referees review every received paper. The referees are Croatian and international scholars well established within their fields. The reviews are carried out independently. Participants in the refereeing process are not paid. The journal keeps a record of all referees.

The paper must be accompanied with a 100-150 words abstract, as well as with 4-5 keywords. CIRR generally accepts papers up to 10 thousand words with the preferred length of 5 to 8 thousand words. Papers from the field of political science, especially international relations, as well as related areas such as law, sociology and economics, shall be considered for publication.

Language and layout

Papers should be submitted in English. It is preferred that the papers are written consistently in either British or US English. All accepted papers will go through a language editing process, but the authors are advised to make the text as correct as possible before submission. The text should be black, justified and written in Times New Roman, 12 pt font and 1,5 line spacing, margins 2,5 cm. Tables should be numbered consecutively and each table should also include a title and a source. It is requested that authors save and send their papers in simpler versions of Microsoft Word in order to avoid difficulties when opening the file.

Figures and Tables

Figures and tables may be uploaded either embedded in the article or as separate files.

Footnotes

Footnotes should be given in numerical order at the bottom of each page. Each footnote should be clearly indicated at the appropriate place in the text with a superscripted Arabic number.

References

Information about references should be within the text in brackets (author surname, year of publishing and page number in case of quoting). For instance:

(Lijphart 1979: 500)

Bibliography

The bibliography should be in alphabetical order, given at the end of the paper. It should be typed in Harvard style. Examples are as follows:

Journal article

Koinova, M., 2009. Diaspora and democratization in the post-communist world. Communist and Post-Communist Studies, 42(1): 41-64.

Book

Hoffman, J., 2004. Citizenship Beyond the State. London: Sage.

Book with an editor

Danaher, P. ed., 1998. Beyond the Ferris Wheel. Rockhampton: CQU Press.

Book with more than one author

Barker, R., Kirk, J. and Munday, R.J., 1988. Narrative analysis. 3rd ed. Bloomington: Indiana University Press.

Books with an anonymous or unknown author

The University Encyclopedia, 1985. London: Roydon.

Book chapter

Robertson, R., 2000. Globalization as a Problem. In: Lechner, F. J. and Boli, J., eds. The Globalization Reader. Oxford: Blackwell.

Web sources

Eriksen, J-M. and Stjernfelt, F., 2009. Culturalism: Culture as Political Ideology, Eurozine, [online]1 June. Available at: http://www.eurozine.com/articles/2009-01-09-eriksenstjernfelt-en.html [accessed 25 October 2011].

Dates should be in the following forms:

12 December 1985 2001–2010

the 1980s

Originality

Submitted papers are assumed to be the original work of the author(s). Papers published in other journals and papers submitted for publication elsewhere will not be accepted.

The processing of papers and paper rejection

The Editors of CIRR read all received papers. If the paper is in accordance with the journals thematic scope and adheres to required academic standards it is checked for plagiarism. At least two anonymous reviewers read and comment each selected paper. We expect the author to seriously consider the reviewers comments and recommendations for revisions. All selected papers pass through a procedure of language editing. Prior to the paper being published, the author signs a license to publish agreement.

A paper can be rejected a) on the decision of the Editors b) in case of negative reviews and c) if the author fails to incorporate the suggestions of the reviewers and Editors deemed to be essential for the attainment of quality necessary for the publishing of the paper.

Book reviews

CIRR will publish reviews of recently published books which are within the scope of the journal. Book reviews should generally be 1,000 to 1,500 words in length. Please contact the editorial team with a proposal to review a particular book.
