# Supplementary material for: Peer Review in Law Journals
Source: Front Res Metr Anal. 2021 Dec 8;6:787768. doi: 10.3389/frma.2021.787768 (PMC8692876; doi:10.3389/frma.2021.787768)
Supplement: Supplementary file 3 [file DataSheet2.ZIP › DOCUMENT - 1331-5978.RTF]

ČASOPIS JAVNI BILJEŽNIK
IZDAVAČ: HRVATSKA JAVNOBILJEŽNIČKA KOMORA

UPUTE RECENZENTIMA

Poštovani, zahvaljujemo Vam unaprijed na recenziji priloženoga članka i sugestijama koje će, ukoliko je potrebno, pomoći autoru da njegov rad bude što kvalitetniji.

Molimo Vas da u Vašoj recenziji priloženog članka pokušate prije svega odgovoriti

1. Je li sadržaj članka primjeren časopisu Javni bilježnik?
2. Odgovara li naslov sadržaju članka?
3. Donosi li članak nova saznanja o građi o kojoj je riječ?
4. Je li članak primjereno strukturiran?

Recenzija bi trebala biti utemeljena na pažljivoj analizi članka i vašoj dobroj informiranosti o temi i relevantnoj literaturi. Molimo Vas da u recenziji također ukažete na eventualne nedostatke u tekstu i iznesete sugestije o nadopunama ili izmjenama. 
Autorski rad koji je u prilogu valja sagledati temeljito i nepristrano te nastojati dati savjete autoru u vezi poboljšanja sadržaja teksta, a uredništvu savjet u svezi s tehničkim i sadržajnim kontekstom članka te u vezi kategorizacije članka.
Ako smatrate da članak treba objaviti, molimo Vas da ga kategorizirate pridržavajući se Standarda Ministarstva znanosti, obrazovanja i sporta RH.
Napominjemo da autor/autori neće biti upoznati s imenima recenzenata od strane uredništva, a i sami recenzenti moraju voditi računa o povjerljivosti i svojem pravu na anonimnost. Ovaj rad se upućuju na najmanje dvije recenzije. U slučaju zahtjevnosti teme članka ili većeg nepodudaranja u ocjeni dvaju recenzenata, rad se upućuje na treću recenziju.
Cijenimo Vaš trud i molimo Vas da recenziju pošaljete najkasnije do ___________
Ako zbog iznenadnih obveza ili drugih okolnosti niste u mogućnosti napisati recenziju, molimo Vas da članak vratite redakciji što prije kako bi se mogao osigurati drugi recenzent.

Recenzent dobiva primjerak tiskanog broja časopisa u kojem je surađivao.

Sažetak recenzije
(molimo vas da obilježite odabrano, a vaše obrazloženje priložite u nastavku)

Naslov članka:

Recenzent:

Datum predaje na recenziju:

Molimo Vas da obilježite (podcrtate ili obilježite bojom):

Prikladnost za objavljivanje	DA	NE	

Vaša preporuka:
Prihvatiti	Odbiti	Napraviti manje izmjene	Napraviti veće izmjene prema mojim uputama	

Kategorija:
Izvorni znanstveni rad	Prethodno priopćenje	Pregledni rad	Stručni članak	
Tekst recenzije (mišljenje recenzenta - na posebnoj stranici s potpisom - ne više od 3000 znakova):

Datum


Napomena: Vaše napomene autoru možete ispisati u vidu balončića u margini članka koji recenzirate da autor može ispraviti naznačeno (vidi više u wordu - Review/New Comment (Pregled/Novi komentari).

PODACI O RECENZENTU:

1)	Datum recenzije: 
2)	Podaci o recenzentu
a.	Ime i prezime: 
b.	Broj znanstvenika u Upisniku znanstvenika:
c.	Ustanova zaposlenja:
d.	Elektronička adresa:
e.	Kratki životopis s popisom relevantnih radova, a koji recenzenta kvalificiraju za recenziju rukopisa:
Napomene:

-	Ukoliko recenzent želi honorar za recenziju, potrebno je dostaviti sljedeće podatke: 

Ime i prezime:
OIB:
Adresa stanovanja:
IBAN žiroračuna i banka u kojoj se vodi: 
Recenzent je: a) zaposlena osoba b) u mirovini
II. stup mirovinskog osiguranja: a) DA     b) NE
