# Supplementary material for: Peer Review in Law Journals
Source: Front Res Metr Anal. 2021 Dec 8;6:787768. doi: 10.3389/frma.2021.787768 (PMC8692876; doi:10.3389/frma.2021.787768)
Supplement: Supplementary file 3 [file DataSheet2.ZIP › DOCUMENT - 1331-5978_1.doc.RTF]

ČASOPIS JAVNI BILJEŽNIK 
UPUTE AUTORIMA 

Javni bilježnik je časopis Hrvatske javnobilježničke komore i Hrvatske javnobilježničke akademije koji donosi stručno-znanstvene radove s područja pravne znanosti, posebno javnog bilježništva.
Vaš članak na hrvatskom ili engleskom jeziku možete poslati na e-mail adresu Uredništva (hjk@hjk.hr). Autori uz članak trebaju na adresu Uredništva poslati popunjen i potpisan autorski obrazac.
Uređivanje časopisa Javni bilježnik kao i načini rješavanja sporova temelje se na Standardu uredničkoga rada i standardu COPE koje preporuča Ministarstvo znanosti, obrazovanja i sporta Republike Hrvatske.
Rukopis je potrebno predati kao word dokument, naslovljen imenom i prezimenom autora. Treba izbjegavati uređivačke zahvate, različite stilove i fontove, formatiranje teksta, uporaba tipke “Tab” za uvlačenje odlomka. Tipku“Enter” koristit za prijelaz u novi red i samo pri odvajanju poglavlja te tipku za razmak samo za razmak između riječi. Lokalne nazive, istaknute sintagme i sl. pisati u kurzivu (italic). Fotografije i druge priloge poslati kao privitak u dobroj rezoluciji. Fotografije i drugi prilozi moraju zadovoljiti uvjete tiska: dimenzije približno moraju odgovarati predviđenim dimenzijama u otisnutom članku; rezolucija fotografija mora biti najmanje 300 dpi, a crteža najmanje 600 dpi. Opseg članka neka ne prelazi 40.000 znakova (uključivši razmake, bilješke i reference) i samo uz konzultaciju s Uredništvom može biti većeg opsega. 
Po predaji rukopisa autor pristaje na recenzentski postupak. Uredništvo odlučuje kojim će recenzentima (najmanje dva) pristigli rad biti poslan na ocjenjivanje. U slučaju različitih ocjena rukopis se šalje na dodatnu ocjenu. Nakon ocjenjivanja rada autor je dužan unijeti promjene u tekstu prema priloženim recenzijama i uputama urednika.
Rukopisi (izvornici) koji nisu uređeni prema mjerilima Uredništva časopisa i kojima nedostaju pojedini prilozi, bit će vraćeni autorima na doradu. Nakon tiska časopisa tekst rukopisa i eventualni prilozi ne vraćaju se autorima. Nakon izlaska iz tiska svaki autor dobiva po jedan primjerak časopisa. 

RASPORED ELEMENATA U ČLANKU
1. Naslov članka.
2. Ime i prezime autora i akademski stupanj (titula), naziv i adresa ustanove u kojoj radi te e-mail adresa.
3. Sažetak (Abstrakt) na hrvatskom i engleskom jeziku (do 100, a ne više od 150 riječi).
4. Ključne riječi na hrvatskom i engleskom jeziku (8 do 10 riječi).
5. Tekst članka. 
	5a) Citiranje priloga u tekstu (slike, karte, tablice i sl.) Npr.: (sl. 1), (karta 1), (tab. 1), (T. 1), (kat. br. 1). Ove priloge potrebno je naznačiti gdje autor želi smjestiti u članku te tu oznaku istaknuti bojom.
	5b) Citiranje literature u tekstu. Časopis prati harvardski sustav navođenja bibliografskih izvora. Citate označiti navodnicima; dulje citate (više od 30 riječi) izdvojiti u zaseban odlomak i označiti navodnicima. Bilješke (footnotes) uvrštavati slijedom teksta, označavati ih arapskim brojkama i koristiti samo za objašnjenja. Reference uklopiti u tekst (ne kao bilješke) u skraćenom obliku: (Obad, 2003).
6. Popis literature i izvora.
Literatura se navodi abecednim redom; djela bez autora ili urednika uvrstiti prema naslovu djela; više djela istoga autora navesti kronološki. U popisu literature treba izbjegavati kraćenje naslova i naziva časopisa. 
Upute što pisati kurzivom (italic) pogledati na stranici: http://pravopis.hr/pravilo/bibliografske-jedinice/87/.
7) Popis priloga.

KATEGORIJE RADOVA U ČASOPISU

Izvorni znanstveni rad je članak čiji znanstveni doprinos može biti istraživačke i/ili teorijske naravi. Prvi sadrži neobjavljene rezultate izvornih znanstvenih istraživanja u cjelovitom obliku odnosno uključuje metodološku, analitičku i interpretativnu razinu teksta te znanstvenu argumentiranost i vjerodostojnost. Drugi sadrži sustavne kritičke preglede i meta-analize pri čemu se izvornost doprinosa određuje s obzirom na fokus, širinu, argumentiranost rasprave i odnos prema suvremenoj obrađenosti teme.

Prethodno priopćenje je znanstveni članak koji sadrži neobjavljene preliminarne rezultate znanstvenog istraživanja koje je u tijeku ili teorijski postavljen problem i okosnice argumentacije, ali bez cjelovite razrade.

Pregledni rad je znanstveni članak koji sadrži sažet prikaz stanja i tendencija razvoja jednog znanstvenog područja, teorijskog problema ili istraživačke teme.

Stručni rad donosi korisne priloge iz područja struke kao što je dokumentacija građe (bez teorijske, metodološke i analitičke obrade), korištenje već objavljenih rezultata znanstvenoga istraživanja s fokusom na primjenu u praksi ili na njihovo širenje (obrazovna svrha) ili sažet i kritičan pregled odabrane aktualne teme. Stručni radovi svojom razumljivošću moraju biti dostupni širokom, ne usko specijaliziranom, čitateljskom profilu.
