# Supplementary material for: Peer Review in Law Journals
Source: Front Res Metr Anal. 2021 Dec 8;6:787768. doi: 10.3389/frma.2021.787768 (PMC8692876; doi:10.3389/frma.2021.787768)
Supplement: Supplementary file 3 [file DataSheet2.ZIP › DOCUMENT - 1331-8004.RTF]

University of Rijeka
FACULTY OF ECONOMICS AND BUSINESS
Ivana Flipovića 4, 51000 Rijeka, Croatia

PROCEEDINGS OF RIJEKA
FACULTY OF ECONOMICS
JOURNAL OF ECONOMICS

EDITORIAL BOARD
Phone: + 385 (0)51 355 182, Fax: + 385 (0)51 212-268
E-mail: zbornik@efri.hr  https://www.efri.uniri.hr/


GUIDELINES TO REVIEWERS – REVIEWER EVALUATION FORM

You are kindly requested to fill in the form which helps evaluate the suitability of the paper for publishing in the Proceedings of Rijeka Faculty of Economics – Journal of Economics and Business (Zbornik radova Ekonomskog Fakulteta u Rijeci – Časopis za ekonomsku teoriju i praksu).

On a scale of 1 to 5, where 5 represents an absolute reason to accept the paper and 1 an absolute reason to reject the paper, please rate the article in each of the following areas according to its originality, relevance of research tasks, methodology, validity of results, conclusions and overall quality and suitability of the article.

THE TITLE OF THE ARTICLE:

(consider each of the following aspects of the article and rate by using circle or bold options from 1 to 5):
	tend to reject-----	tend to accept	
1	The article's title reflects the content and purpose of the article	1	2	3	4	5	
2	The abstract is concise and relevant (up to 150-200 words)	1	2	3	4	5	
3	The key words provide adequate index entry for the article	1	2	3	4	5	
	(up to 5 words)						
4	The introduction contains a clearly stated objectives	1	2	3	4	5	
5	A brief overview of general knowledge about the problem at	1	2	3	4	5	
	the beginning of the investigation gradually introduces the						
	main issue of the article.						
6	A  clear-cut  methodology  of  the  research  is  concisely  and	1	2	3	4	5	
	systematically listed and defined.						
7	An extensive overview of the issue (supported by up-to-date	1	2	3	4	5	
	references   and   ISI   citations)   provides   evidence   and						
	counterevidence of the author's own findings and research						
	results						
8	The article is original and presents an important and suitable	1	2	3	4	5	
	contribution to economic theory and practice						
9	The research results are valid with respect to the relevance of	1	2	3	4	5	
	the methodology applied, conclusions and recommendations						
10	Conclusions  illustrate  the  research  results,  findings  and	1	2	3	4	5	
	recommendations   showing   what   is   new   and   giving						
	suggestions for future research						

TEXT EVALUATION AND SUITABILITY (consider each of the following aspects of the article and rate by using circle or bold options from 1 to 5):

11	The article is well-organized and conforms to the format of the	1	2	3	4	5	
	Journal						
12	The article's length is appropriate (16-20 A4 size pages)	1	2	3	4	5	
13	The article makes an appropriate use of graphs, diagrams and	1	2	3	4	5	
	tables						
14	The  references  used  are  up-to  date  and  the  format  of  the	1	2	3	4	5	
	citations is in Harvard style						
15	The article is written in standard language, free of spelling and	1	2	3	4	5	
	grammar  mistakes,  interesting  and  relevant  for  local  and						
	international readers						


FINAL RECOMMENDATION (mark one of the chosen option):

?	Accepted

?	Conditionally accepted

?	Rejected


If judged positively, please recommend the appropriate classification of the article:


?	Original scientific paper is a scientific paper that includes new results based on the research. The information given in the article can be verified by: a) Reproducing experiments and obtain the same/similar results or with tolerable experimental mistakes as estimated by the author himself; b) Repeating the author's observations and judge his analyses; c) Checking the author's analyses and deduction on which the author's analyses are based.

?	Preliminary communication is an article that includes at least one or more pieces of scientific information, but does not include the necessary details to check the scientific cognition.

?	Conference paper is an article that deals with the author's presentation at a conference.

?	Review article is an article that analyzes a special scientific problem already dealt with in published scientific works, but his approach is original and new.

?	Professional paper is an article that deals with specific problems in some professional areas.

GENERAL COMMENTS AND REVIEWER'S SUGGESTIONS FOR IMPROVEMENT OF
THE ARTICLE


REVIEWER' S DATA:

First and second name of the reviewer: ___________________________________________________

Signature:


Date: _____________________________________
