# Supplementary material for: Peer Review in Law Journals
Source: Front Res Metr Anal. 2021 Dec 8;6:787768. doi: 10.3389/frma.2021.787768 (PMC8692876; doi:10.3389/frma.2021.787768)
Supplement: Supplementary file 3 [file DataSheet2.ZIP › DOCUMENT - 1331-8004_1.RTF]

GUIDELINES FOR AUTHORS

– UPUTE AUTORIMA


Guidelines for authors • Upute autorima	725		
Zb. rad. Ekon. fak. Rij. • 2020 • vol. 38 • no. 2 • 723-744			

Authors are kindly requested to read carefully the Guidelines amended with detailed methodological instructions


GUIDELINES FOR AUTHORS

Zbornik radova Ekonomskog fakulteta u Rijeci: časopis za ekonomsku teoriju i praksu/Proceedings of Rijeka Faculty of Economics: Journal of Economics and
Business is an international peer reviewed journal open to scientists from all over the world in different fields of economic theory, economic policy and related research. As a rule, papers are published in English. However, the Journal may publish articles in other world languages.

Editorial procedure

The Editorial Board receives all manuscripts. After reviewing and accepting the manuscripts, Editor-in-Chief subjects them to the members of the Editorial Board for the assessment process, and in case of the specific content of the text, to external experts as well. After evaluation, Editor-in-Chief proposes Editorial decisions for further action including: (a) which manuscript, if necessary, should be returned to the author with suggestions, comments and recommendations for improvement, primarily methodological presentation of research materials, (b) which manuscripts could be sent to peer-review process, (c) which manuscripts should be rejected because of the reasons as follows:

•• the subject matter does not meet the required scientific level,

•• the article with a similar topic has already been published by the same author,

•• the subject matter does not meet the criteria of the Journal, especially if:

– the content is beyond the concept of scientific publishing orientation of the Journal (distinguished by the relevant databases), and
– does not meet international scientific and methodological standards that the Journal must comply with.

If an article is not accepted, the Editorial Board sends a notification to the author, but the manuscript is not returned.

If the manuscript is improved adequately, it is sent to two reviewers for extramural review.

If the manuscript is considered for publishing, the author will receive the Authorship Statement (Copyright Assignment Form), which should be filled in, signed and returned to the editor. In this way the authors confirm the originality of the article and validity of authorship.

In order to avoid withdrawing the paper until it is published or rejected, by signing the Authorship Statement, the authors assert compliance with the review process.


Book reviews, reviews on doctoral dissertations, as well as reviews on international conferences and seminars are not submitted to extramural reviews. They are accepted or rejected by the Editor and co-editors.

Review process

All scientific articles submitted for publication in Zbornik radova Ekonomskog fakulteta u Rijeci: časopis za ekonomsku teoriju i praksu/Proceedings of Rijeka Faculty of Economics: Journal of Economics and Business are double-blind peer review by two academics appointed by the Editorial board: one from Croatia and one from abroad. Reviewers stay anonymous for the authors and so do the authors for the reviewers. The reviewers are asked to apply highest international standards in their assessment of the submitted work. Reviewers receive an article and a Reviewer Evaluation Form with instructions how to fill it in. If the article does not fulfill the primary criteria of originality and relevance to the subject, it should not be accepted.

The categories in which articles, if judged positively, might be classified are:

Original scientific paper is a scientific paper that includes new results based on the research. The information given in the article can be verified by:

`)	reproducing methodological procedure and obtain the same/similar results or with tolerable degree of mistakes as estimated by the author himself, or

`)	repeating the author's observations and judge his analyses, or

`)	checking the author's analyses and deduction on which the author's analyses are based.

Preliminary communication is an article that includes at least one or more pieces of scientific information, but does not include the necessary details to check the scientific cognition.

Conference paper is an article that deals with the author's presentation at a conference, and has not been previously published as a full text.

Review article is an article that analyzes a special scientific problem already dealt with in published scientific works, but his approach is original and new.

Professional paper is an article that deals with specific problems in some professional areas.

After receiving the reviews, the Editorial Board will give clear general instructions to the author for further work on the manuscript. The comments and suggestions made by the reviewers should be addressed and closely followed by the author prior to final recommendation on acceptance or rejection made by the Editorial Board.

Only manuscripts with two positive reviews are published.

After completion of peer review and amendment of the text of the accepted articles by the authors and editors, Editor-in-Chief, proposes the sequence of accepted papers in the table of contents.


Guidelines for authors • Upute autorima	727		
Zb. rad. Ekon. fak. Rij. • 2020 • vol. 38 • no. 2 • 723-744			

Criteria for the order are:

0()	categorization according to the degree of scientific excellence,

0()	in case of two different levels of categorization of a particular article by reviewers, Editor-in-Chief proposes a decision to the Editorial Board, and as a rule, in favour of the author, i.e. a higher category,

0()	appreciation of topical issues, authors and common interest of the Journal taking into account the competitiveness at the same level of scientific excellence (category).

In order to increase the quality and number of papers published, the Editorial Board strives to fasten the publishing process by informing the authors on the status of their papers within ten working days of the submission. Provisional deadline for finishing the reviewing process, technical revisions and online publication of the paper shall last no longer than two months. Moreover, as of 2018, the forthcoming papers that will be included within the regular biannual issues are initially published on our Journal's website.

Organization of the manuscript

The manuscript should include the research aim and tasks, with detailed methodology, the research objective, achieved results and findings, conclusions and a list of references.

The authors of the manuscript should conform to the format and documentation requirements that are given below:

The separate page should contain: the article title, the author's full name, academic affiliation (title, institution, scientific field), phone, fax, e-mail address and personal­ website. If there are more authors than one, full names, respective affiliations and addresses of co-authors should be clearly stated. Authors'academic affiliation should be:

– in the language of the article,

– in Croatian language,

– in English if the original language of the article is other than English.

The first page should contain: the article title, abstract, JEL classification and key words in the language of the article. At the end of the article all data should be also:

– in Croatian language,

– in English if the original language of the article is other than English.

Main body of the text should contain: introduction, headings, footnotes, references in the text, tables, figures, conclusions and references.

Technical requirements

The manuscript submitted for publication should be in Microsoft Office Word (Ver. 95+), with maximum length up to 8,000 words in length (16-20 A4 size pages), printed in font style Times New Roman (12 point), single-spaced, justified and


728	Guidelines for authors • Upute autorima		
	Zb. rad. Ekon. fak. Rij. • 2020 • vol. 38 • no. 2 • 723-744		

without any special styling. Should a word, a phrase or a sentence be highlighted, italic font style can be used and never bold. Paragraphs are divided by double spacing and all margins are at 2.5 cm. In case the paper exceeds the normal length, the Editors' consent for its publication is needed.

JEL classification should be suggested by the authors themselves according to the classification available on the Journal of Economic Literature website: http://www. aeaweb.org/journal/jel_class_system.html.

Footnotes could be used only as an additional explanatory section of the text and should be numbered consecutively in Arabic numerals.

Section headings (from Introduction to Conclusions) must be short, clearly defined and bear Arabic numerals. If there are subsection headings they are outline numbered (e.g. 1; 1.1.; 2.; 2.1.; 2.1.1. etc.)

All tables and figures should bear Arabic numerals and must have captions. Tables set in MS Word may be included in the text.
Note: If MS Excel or other programs are used for tables, figures or illustrations, make sure to enclose them as a separate file on disk, separately from the text.

Before submission of the manuscript, the authors of the manuscript are advised to conform to the format and documentation requirements.

Text organization and style

Authors should apply scientific methodology in presenting the contents of their papers complying with the standards of scientific publications ("Harvard style"). This implies the procedure as follows:

(1) Title and the content of the paper:

The title is the most important summary of a scientific article, which reflects the scope of investigation and the type of study. Therefore, the title should not contain words such as "analysis", "methods" and similar.

The content of the paper consists of:

	Abstract – below the title

	Key words

	JEL classification.

It is followed by the main body of the paper divided into sections. The section headings are as follows:

	Introduction

	Literature review

	Methodology/method/model/conception of analysis (the third section)

	Empirical data (documentation background) and analysis (the fourth section)

	Results and discussion (the fifth section)

	Conclusions (the sixth section).


Guidelines for authors • Upute autorima	729		
Zb. rad. Ekon. fak. Rij. • 2020 • vol. 38 • no. 2 • 723-744			

0()	The content of some parts of the material presented: a. Abstract – up to 100-250 words must contain:

	purpose and research objective,

	methodology/method/model/conception of analysis,

	main findings and results of research (analysis),

	the underlined conclusion of research.

The abstract should not be written in paragraphs!

`.	Key words should disclose the essence of the article (up to 5 key words).

`.	JEL classification – the author should classify the subject matter of the article according to the code of The Journal of Economic Literature (JEL).
`.	Introduction – defines the problem and the subject matter of the research referring to recent bibliography and findings. However, these can more specifically be dealt with in the second section Literature review. The last part of the introduction is reserved for setting the hypothesis of the research that will be later on analyzed at the beginning of the conclusions. Finally, Introduction ends up by giving clues of the organization of the text.

`.	Literature review – precedes a research section providing readers with a cutting-edge context of the referential literature dealing with crucial points of current knowledge based on the relevant results of the current research. Literature review should be a synthesis of previous research, justifying the theoretical and empirical contributions of the respective paper, a not a simple listing of previous scientific contributions.

`.	Methodology/method/model/conception of analysis – usually in the third section of the paper, methodology/method/model/conception of the analysis should be transparently presented and pointed out in case of the research results being subjected to re-testing by interested researchers (it is one of the fundamental principles of the scientific methodology).
`.	Empirical data and analysis – contain documentation background and the results of the empirical analysis. The data sample shall be elaborated and the obtained results shall be explained based on statistical and econometric features, and their economic meaning.

`.	Results and discussion – explain the results, especially their economic significance and messages. In this section, the author(s) need to elaborate how their results and conclusions contribute to the scientific field and provide practical implications and recommendations.

`.	Conclusions – is not supposed to be a summary! Conclusions are the author's original thoughts and evaluation of the obtained results including the items as follows:

	Explanation of the working hypothesis – proved or not proved.

	Assessment of the results of research/analysis with the focus on what can be classified as a new contribution to economic science.

	Attention drawn to research limitations and problems.


	Guidelines to future research.

	Assessment of institutional-systemic implications of the results obtained by the research (suggestions and recommendations for direction or changes of economic system, economic and financial policy, development policy, instruments, measurements or similar).

It is recommended not to write conclusion in paragraphs.

0()	References should include only the titles (sources) that have been referred to and quoted in the paper.

TABLES should be included in the text in order to present the exact values of the data that cannot be summarized in a few sentences in the text. Each column heading for numerical data should include the unit of measurement applied to all data under the heading. Large numbers can be expressed in smaller units with appropriate column headings (in thousands, millions, etc), and logical presentation of data using table grid option in MS Word for table lines (both vertical and horizontal). Each table should be self-explanatory, bearing Arabic numerals (e.g. Table 1, Table 2, etc.) with an adequate title (clearly suggesting the contents) and the source of the data should be stated below the table, if other than author's.

FIGURES (GRAPHS, DIAGRAMS, ILLUSTRATIONS) should also be included in the text. They should be numbered in sequence with Arabic numerals, followed by the figure title, and the legend to the figure that contains all the necessary explanations of symbols and findings. The source of the data presented in the figure should be stated below the figure if other than author's.

Note. The text should not simply repeat the data contained in tables and figures, i.e. the text and the data in tables and figures should be related in the text by means of reference marks.

REFERENCES. The ISI citations should be followed by all authors of Zbornik radova Ekonomskog fakulteta u Rijeci: časopis za ekonomsku teoriju i praksu/ Proceedings of Rijeka Faculty of Economics: Journal of Economics and Business

(please, refer to http://www.isinet.com) and references to other publications must be in Harvard style. At each point in the text that refers to a particular document, insert the author's surname and publication year in brackets: (Rowley, 1996) or (Cutler and Williams, 1986), or in the case of more than two, the first author (from the title page) followed by "et al." (Matlock et al., 1986). If the author's name is repeated no ibid is used but his surname is repeated. If the author's name occurs naturally in the text, the year follows in the brackets: The work of Stevens (2001) was concerned with what they teach at Harvard Business School. In case of direct quotations the page numbers should be added, e.g. (Jones, 1995: 122–123).

At the end of the article a list of references is organized alphabetically as follows:

•  Books: Surname, Initials (year) Title, Place of publication: Publisher. See example:

Callicott, J. B. (1994) Earth's Insights: A Survey of Ecological Ethics from the Mediterranean Basin to the Australian Outback, Berkeley: University of California Press.


Guidelines for authors • Upute autorima	731		
Zb. rad. Ekon. fak. Rij. • 2020 • vol. 38 • no. 2 • 723-744			

If there are two or three authors you put down their surnames followed by initials:

Riddersträle, J., Nordström, K. (2004) Karaoke Capitalism Management for Mankind,

Harlow: Pearson Education Ltd.

If there are multiple authors (four or more) the first author's surname (from the title page ) is followed by et al.:
Norton, M. B. et al. (1981) A People and a Nation – A History of the United States,

Boston: Houghton Mifflin Company.

• Journals: Surname, Initials (year) "Title", Journal, Volume, Number, pages. See example:

Kostelich, E. (1995) "Symphony in Chaos", New Scientists, Vol. 146, No. 1972, pp. 36–39.

Fox, S. (1994) "Empowerment as a Catalyst for Change: An Example from the Food Industry", Supply Chain Management, Vol. 2, No. 3, pp. 29–33.

If there are multiple authors (four or more), the first author's surname (from the title page ) is followed by et al. See example:
Di Noia, C. et al. (1999) "Should Banking Supervision and Monetary Policy Tasks be Given to Different Agencies?", International Finance, Vol. 2, No. 3, pp. 285–361.
If there are multiple works by the same author published in the same year, the "a, b, c" is used after the year. See example:
Quah, D. T. (1993a) "Empirical Cross-section Dynamics in Economic Growth", European Economic Review, Vol. 37, No. 2–3, pp. 426–434.
---------- (1993b) "Galton's Fallacy and Tests of the Convergence Hypothesis",

Scandinavian Journal of Economics, 95, Vol. 95, No. 4, pp. 427–443.

---------- (1994) "Exploiting cross Section Variation for Unit Root Inference in Dynamic Data", Economics Letters, Vol. 44, No. 1–2, pp. 9–19.
---------- (1996a) "Empirics for Economic Growth and Convergence", European Economic Review, Vol. 40, No. 6, pp. 951–958.

---------- (1996b) "Regional Convergence Clusters across Europe", European Economic Review, Vol. 40, No. 6, pp. 951–958.

The author should provide Digital Object Identifier (DOI) for each reference that can be found whether it exists at CrossRef http://www.crossref.org/ and DOI appears in the form such as https://doi.org/10.5468/ogs.2016.59.1.1.

DOI is inserted by the author at the end of references as shown in the example as follows:
Hall, J.K., Daneke, G.A., Lenox, M.J. (2010) "Sustainable Development and Entrepreneurship: Past Contributions and Future directions", Journal of Business Venturing, Vol. 25, No. 5, pp. 439–448, https://doi.org/10.1016/j.jbusvent.2010. 01.002.

• Internet sources: Author's/editor's surname (year), "Title of the article", Title of the journal [type of medium], date of publication, volume number, pagination or online equivalent, <availability statement> [date of accession if necessary]:


732	Guidelines for authors • Upute autorima		
	Zb. rad. Ekon. fak. Rij. • 2020 • vol. 38 • no. 2 • 723-744		

Martin, C.L. (1998) "Relationship Marketing: a High-Involvement Product Attribute Approach", Journal of Product and Brand Management [Internet], Vol. 7, No. 1, pp. 6–26. Available at: <http://www.apmforum.com/emerald/marketing-research-asia.htm> [Accessed: October 3, 2002]

• Chapter/section from a book of collected writings: Author of the chapter/ section (year of publication) "Title of the Chapter/section". In Author/editor of collected work, Title of collected works, Place of publishing: Publisher. Example:

Porter, M.A. (1993) "The modification of method in researching postgraduate education". In Burges, R.G. ed., The research process in educational settings: ten case studies, London: Falmer.

• Conference papers from conference proceedings: Author of the conference paper (year of publication) "Title of the conference paper". In Title of conference proceedings. Place of publication: Publisher, pagination of section referred to:

Fedchak, E. & Duvall, L. (1996) "An engineering approach to electronic publishing". In Proceedings of the International Workshop on Multimedia Software Development, 25–26 March, Berlin, Los Alimos, Ca: IEEE Comput. Soc. Press,
`.	80–88.

	Theses and dissertations: Author's name (year) Title of doctoral dissertation, the name of the awarding institution:

Whitehead, S.M. (1996) Public and private men: masculinities at work in education management, PhD thesis, Leeds Metropolitain University.

• Official publications: Title of publication/organisation/institution (year) Title, Place of publishing: Publisher. Example:

Department of the Environment (1986) Landfilling wastes, London: HMSO (Waste management paper, 26).

Guidelines for other publications

The Journal reserves the main printing space for scientific articles accepted from scientists all over the world. However, the other part is devoted to reviews of scientific achievements, which are classified by the editorial board as follows:

• Book review. A brief overview of the book is written in a clear and concise manner evaluating the structure, style and scientific achievements of a particular book. It starts with the title of the book, and the main data: the author's name, academic affiliation, title of the book, subtitle (if any), year of publishing, publisher, volume (including number of pages), type of publication (hardcover or paperback), language, ISBN and the author's contact address (e-mail address). If there are more authors than one, full names, respective affiliations and addresses of co-authors should be clearly stated. At the end of the text it is written "Reviewed by" stating the reviewer's name, academic title and affiliation. In addition to the book review, the copy of the cover page of the book is submitted.


Guidelines for authors • Upute autorima	733		
Zb. rad. Ekon. fak. Rij. • 2020 • vol. 38 • no. 2 • 723-744			

	Review on Doctoral Dissertations. It starts with the following data: the name of the author of PhD dissertation, author's affiliation (institution he or she works for), the title of the PhD dissertation, the names of the members of the committee in charge, their affiliation, the date when the PhD dissertation was defended including the name of the awarding institution, and in which field of science has PhD been granted. The review evaluates the structure, style, research methodology and results. It analyzes theoretical and practical contribution to a particular scientific field, and implications for further research. At the end of the review, there is the reviewer's name, academic title and affiliation.

	Reviews on International Conferences and Seminars. It starts with the following data: title of the conference (seminar), organizer(s), date of the conference (seminar), venue, language, the name and e-mail address of the contact person, conference/seminar websites, and how, when and where conference material will be published and can be obtained (i.e. selected and reviewed conference papers). The review should provide a clear and comprehensive overview of the main objectives of the conference, mentioning the keynote speaker(s), participants' panel discussion on scientific achievements, research findings and suggestions for further research and pressing questions in need of answer. The reviewer's name is stated at the end of the text with his or her academic title and affiliation.

	In Memoriam. It is a short text (not longer than 1 A4 page) written in memory of a scientist or special contributor and his works. The author's name is stated at the end of the text with his or her affiliation.

	Letters to the Editor. Special section is available for comments, opinions and suggestions by readers, authors and other contributors.

Other important notes

If the author of the manuscript does not conform to the primary format and documentation requirements that are given in the instructions, editors reserve the right to reject the article, or adapt it to comply with the Journal standards, providing other acceptance criteria are fulfilled.

Therefore, avoid complex formatting; the text will be styled according to the Journal design specifications.

The editorial board makes the final decision on acceptance criteria and priority order in the table of contents.

The authors receive one copy of the journal in which their articles are published.

The author(s) should register via (https://orcid.org/signin) in order to obtain an ORCID identifier. The ORCID identifier is a researcher's unique and permanent identifier which allows for better visibility and interoperability of wide range of information systems.

Publisher does not charge "submission fee". If the paper is accepted for publication, the author receives notification on paying publishing fee (285 EUR) and should pay it prior to the publication of the paper. The author is obliged to cover bank charges.


734	Guidelines for authors • Upute autorima		
	Zb. rad. Ekon. fak. Rij. • 2020 • vol. 38 • no. 2 • 723-744		

Proofreading

Authors are responsible for ensuring that their manuscripts are accurately typed before final submission. One set of proof will be sent to authors, if requested, before the final publication, which must be returned promptly. At this stage, only misprints will be corrected.

Copyright

An article submitted to the journal should be authentic and original contribution of the author and should have neither been published before nor be concurrently submitted to any other journal as to avoid double publication.

Once the article has been accepted for publishing, the author commits him/herself not to publish the same article elsewhere without the Editorial Board's permission. In the event that the Editorial Board gave permission for publication in another journal, it should be stated that the article has previously been published in the journal Zbornik radova Ekonomskog fakulteta u Rijeci: časopis za ekonomsku teoriju i praksu/ Proceedings of Rijeka Faculty of Economics: Journal of Economics and Business.

Each manuscript, in order to avoid plagiarism, is checked by using CrossCheck Service.

Authors submitting articles for publication warrant that their paper is not an infringement of any existing copyright and will indemnify the publisher against any breach of such warranty. For ease of dissemination of scientific contributions and to ensure ethical principles of use, once accepted for publishing, papers and contributions become the legal copyright of the publisher unless otherwise agreed.

Submission of the manuscript

Electronic submission of the manuscript should be accompanied by the author's cover letter containing: the article title, the author's full name, academic affiliation (title, institution, scientific field), phone, fax, e-mail address and personal website­. If there are more authors than one, full names, respective affiliations and addresses of co-authors should be clearly stated. Authors'academic affiliation should be:

– in the language of the article,

– in Croatian language,

– in English if the original language of the article is other than English.

The address is: zbornik@efri.hr.

More detailed information on the Zbornik radova Ekonomskog fakulteta u Rijeci: časopis za ekonomsku teoriju i praksu/Proceedings of Rijeka Faculty of Economics: Journal of Economics and Business can be obtained on the website of the Faculty of Economics University of Rijeka: http://www.efri.uniri.hr/en/proceedings.

EDITORIAL BOARD


Guidelines for authors • Upute autorima	735		
Zb. rad. Ekon. fak. Rij. • 2020 • vol. 38 • no. 2 • 723-744			

Uredništvo skreće pozornost autorima da pažljivo pročitaju upute koje su dopunjene detaljnom metodologijom organizacije teksta

UPUTE AUTORIMA

Zbornik radova Ekonomskog fakulteta u Rijeci: časopis za ekonomsku teoriju i praksu/Proceedings of Rijeka Faculty of Economics: Journal of Economics and

Business međunarodno je recenziran časopis, otvoren za suradnju znanstvenicima iz cijelog svijeta iz različitih područja ekonomske teorije i prakse. Tekstovi se objavljuju, u pravilu, na engleskom jeziku. Međutim, časopis može objavljivati tekstove i na ostalim svjetskim jezicima.

Politika uređivanja

Uredništvo zaprima sve rukopise. Glavni i odgovorni urednik tekstove, nakon pregleda i prihvaćanja, upućuje u postupak prosudbe ("assessment process") članovima Uredništva, ali i ekspertnim stručnjacima izvan Uredništva u slučaju specifičnog sadržaja teksta. Poslije prosudbe, glavni i odgovorni urednik predlaže Uredništvu odluke za daljnji postupak i to: (a) koje se rukopise, u slučaju potrebe, vraća autoru sa sugestijama, preporukama i primjedbama radi poboljšanja, prije svega, metodološke prezentacije građe istraživanja; (b) koje rukopise uputiti u postupak recenzija ("reviewing process"); (c) koje rukopise odbiti, budući da:

•• tema ne zadovoljava zahtijevanu znanstvenu razinu;

•• autor je članak sa sličnom temom već objavio;

•• tema ne ispunjava kriterije časopisa, osobito ako je:

– sadržaj izvan koncepcije znanstveno-publicističke orijentacije časopisa (uvažene od relevantnih baza referiranja) i
– ne ispunjava svjetske znanstveno-metodološke standarde kojih se časopis mora pridržavati.

Ukoliko članak nije prihvaćen Uredništvo autoru šalje obavijest, ali rukopis se ne vraća.
Ukoliko je autor usvojio primjedbe i sugestije iz postupka prosudbe i poboljšao tekst prema zahtjevu navedenog postupka, rukopis se šalje u postupak recenziranja.
U tom slučaju autoru se šalje formular "Izjava o autorskim­ pravima" koji treba ispuniti, potpisati i vratiti Uredništvu. Svojim potpisom autor potvrđuje izvornost svoga članka i svoje autorstvo.
Da bi se izbjeglo autorovo odustajanje od objave članka u tijeku recenzentskog postupka, autor se obvezuje svojim potpisom u "Izjavi o autorskim pravima" da prihvaća recenzentski postupak. Nakon toga slijedi odluka o odbijanju ili prihvaćanju članka.


736	Guidelines for authors • Upute autorima		
	Zb. rad. Ekon. fak. Rij. • 2020 • vol. 38 • no. 2 • 723-744		

Prikazi knjiga, doktorskih disertacija, međunarodnih konferencija i drugih znan­ stvenih skupova ne podliježu recenziji. Uredništvo odabire i uređuje prikaze koji su relevantni za objavljivanje u časopisu sukladno koncepciji uređivačke politike.

Postupak recenziranja

Svi znanstveni članci obvezno se recenziraju. Za svaki članak predviđena su dva recenzenta, jedan iz inozemstva i jedan iz Hrvatske, a oba su anonimna. Imena autora također su za recenzente anonimna. Recenzenti pišu recenziju prema dobivenim uputama i na propisanom formularu. Ukoliko članak, prema stajalištu recenzenta,­ ne udovoljava međunarodnim standardima i kriterijima časopisa, Uredništvo članak ne prihvaća. Ukoliko pak recenzent članak pozitivno ocijeni, može ga kategorizirati­ u jednu od kategorija vrsnoće:

Izvorni znanstveni članak (Original scientific paper) je originalno znanstveno djelo u kojem su izneseni novi rezultati fundamentalnih ili primijenjenih istraživanja. Informacije iznesene u članku potvrđuju da je moguće:

`)	reproducirati metodološki i računski postupak i dobiti rezultate s jednakom točnošću ili unutar granice stupnja slobode, kako to navodi autor; ili

`)	ponoviti autorova opažanja i prosuditi njegove analize; ili

`)	provjeriti točnost analiza i dedukcija na kojima se temelje autorovi nalazi.

Prethodno priopćenje (Preliminary communication). Taj znanstveni članak obavezno­ sadrži jednu ili više znanstvenih informacija, ali bez dovoljno pojedinosti koje bi omogućile čitatelju provjeru iznesenih znanstvenih spoznaja.

Izlaganje sa znanstvenog skupa (Conference paper). Može biti objavljeno samo kao cjeloviti članak koji je prethodno referiran na znanstvenom skupu, a u obliku cjelo­ vitog članka nije objavljeno u zborniku skupa.

Pregledni rad (Review article). Sadrži poseban problem o kojem je već publiciran znanstveni rad, ali mu se pristupa na nov način.

Stručni članak (Professional paper). Sadrži korisne priloge iz struke i za struku.

Nakon primljenih recenzija, Uredništvo analizira recenzije. Ukoliko je to potrebno, rad vraća autoru koji ga je dužan prilagoditi zahtjevima recenzenta. Rad se dostavlja autoru nakon primitka obje recenzije. Međutim, u slučajevima gdje se ne dovodi u pitanje kvaliteta i autoriziranost teksta, Uredništvo također može intervenirati. Nakon što autor ponovno dostavi rad, Uredništvo utvrđuje usklađenost članka s primjedbama recenzenata.

Objavljuju se samo kategorizirani radovi koji imaju dvije pozitivne recenzije.

Glavni i odgovorni urednik, nakon završetka postupka recenziranja te izmjena i dopuna tekstova od strane autora i Uredništva, predlaže redoslijed prihvaćenih članaka u sadržaju. Kriteriji redoslijeda jesu:
(1)  kategorizacija sukladno stupnju znanstvene vrsnoće;


Guidelines for authors • Upute autorima	737		
Zb. rad. Ekon. fak. Rij. • 2020 • vol. 38 • no. 2 • 723-744			

0()	u slučaju dva različita stupnja kategorizacije članka od strane recenzenata, glavni i odgovorni urednik predlaže odluku Uredništvu, u pravilu, u prilog autora, tj. višu kategoriju;
0()	u slučaju konkurentnosti radova iste razine znanstvene vrsnoće (kategorije) uvažava se aktualnost teme, autora i interes časopisa.

U okviru ciljeva povećanja kvalitete i broja objavljenih radova Uredništvo se obvezuje da će nastojati odgovoriti autorima o statusu poslanog rada u okviru od deset radnih dana. Također, od 2018. godine, objavljivat će se radovi u najavi na web stranici Časopisa koji će biti tiskani u redovnom polugodišnjem tiskanom izdanju. Okvirni rok završetka recenzentnog postupka, tehničkog uređivanja te online objave rada je dva mjeseca.

Sadržaj članka

Rad treba biti relevantan za međunarodnu znanstvenu i stručnu javnost s jasno naznačenim ciljevima i rezultatima istraživanja, zaključkom, referencama u tekstu i bibliografskim jedinicama na kraju rada. Ideje u radu moraju biti originalne i trebaju značajno doprinositi razvoju predmeta istraživanja, a metodologija mora biti jasno opisana.

Autori u članku moraju posebnu pozornost obratiti na odgovarajuće strukturiranje teksta sukladno priznatim standardima znanstvene metodologije u ekonomskim istraživanjima, kako je navedeno:

Posebna stranica treba sadržavati: naslov članka, ime i prezime autora ili ako je više koautora za svakog ponaosob znanstveno zvanje, stručnu spremu, znanstveni interes, odnosno područje kojim se autor bavi, naziv i adresu institucije u kojoj je autor zaposlen, broj telefona, broj faksa, e-mail adresu i osobnu web stranicu. Svi navedeni podaci moraju biti napisani:

– na jeziku članka,

– na hrvatskom jeziku,

– na engleskom jeziku ako izvorni jezik članka nije engleski.

Prva stranica članka treba sadržavati: naslov članka, sažetak, JEL klasifikaciju i ključne riječi na jeziku članka.

Na kraju članka isti podaci daju se na:

– hrvatskom jeziku, te

– na engleskom jeziku ako izvorni jezik članka nije engleski.

Tekst članka mora početi uvodom, a sadrži još glavna poglavlja, fusnote, tablice, grafikone, slike, reference u tekstu, zaključak i popis korištene literature.

Tehničko uređivanje članka

Tekst rada piše se u programu Microsoft Office Word (inačica 95 i viša). Opseg rada smije iznositi do 8.000 riječi, što je oko 16 stranica A4 formata, a tekst je pisan vrstom slova Times New Roman (veličine 12 točaka), s jednostrukim razmakom,


738	Guidelines for authors • Upute autorima		
	Zb. rad. Ekon. fak. Rij. • 2020 • vol. 38 • no. 2 • 723-744		

poravnan s obje strane, pisan od početka reda (bez uvlačenja prvog retka pasusa), s marginama od 2,5 cm. Ukoliko je u tekstu potrebno posebno označiti neku riječ ili rečenicu koriste se pisana kosa slova (italic), nikako ne podebljana (bold). Za odva­ janje pasusa koristi se dvostruki razmak. Opseg rada može biti veći samo u dogovoru s glavnim i odgovornim urednikom.

JEL klasifikaciju predlaže autor u skladu s Journal of Economic Literature klasifika­ cijom koja je dostupna na web stranici: http://www.aeaweb.org/journal/jel_class_ system.html.

Fusnote se rabe samo za dodatna pojašnjenja osnovnoga teksta. One se ne koriste kao poziv na Literaturu. Označavaju se na dnu stranice, u kontinuitetu, kroz cijeli članak, arapskim brojevima počevši od 1.

Naslovi poglavlja (od Uvoda do Zaključka) moraju biti kratki i jasni, te redom numerirani arapskim jednocifrenim brojevima. Poglavlja mogu imati i podpo­glavlja koja se obavezno numeriraju s dvocifrenim odnosno trocifrenim brojevima. (primjer: 1; 1.1.; 2.; 2.1.; 2.1.1. itd.), ali ne više od toga.

Tablice, grafikoni i slike moraju imati, broj, naziv i izvor podataka. Numeriraju se u kontinuitetu arapskim brojevima (posebno grafikoni, posebno slike).

Važna napomena: Ukoliko tablica, grafikon ili slika sadržavaju posebne znakove te su rađeni u posebnom programu dostavljaju se u posebnom dokumentu s točno navedenim i označenim položajem na kojem dolaze u tekstu.

Članak mora zadovoljavati sve tehničke propozicije navedene u ovim uputama.

Stil i organizacija teksta

Autori se obvezno moraju pridržavati znanstvene metodologije prezentacije građe u pisanju tekstova koja je uobičajena u znanstvenim publikacijama ("Harvard style"). To zahtijeva sljedeći pristup:

(1) Naslov i organizacija prezentacije građe:

Naslov je najvažniji sažetak rada koji mora održavati sadržaj i svrhu rada. Ne smije biti "opisan" niti sadržavati riječi poput "analiza" ili "metoda", i sl. Građu se raspo­ ređuje u dijelove kao što su:

	Sažetak (Abstract) – ispod naslova

	Ključne riječi

	JEL klasifikacija.

Iza toga slijedi glavni dio rada podijeljen u odlomke:

	Uvod

	Pregled literature

	Metodologija/metoda/model/koncepcija analize (treće poglavlje)

	Empirijski podaci (dokumentacijska podloga) i analiza (četvrto poglavlje)

	Rezultati i diskusija (peto poglavlje)

	Zaključci (šesto poglavlje).


Guidelines for authors • Upute autorima	739		
Zb. rad. Ekon. fak. Rij. • 2020 • vol. 38 • no. 2 • 723-744			

0()	Sadržaj pojedinih dijelova prezentirane građe:

a. Sažetak – ispisuje se u 100–250 riječi, a obvezno treba sadržavati:

	utvrđeni cilj istraživanja,

	metodu/model/koncepciju analize,

	glavni rezultat istraživanja (analize),

	temeljni zaključak istraživanja.

Sažetak se ne smije pisati u odlomcima!

`.	Ključne riječi – moraju odražavati suštinu sadržaja rada, a navodi se do pet takvih riječi.
`.	JEL klasifikacija – autor svoju temu mora razvrstati sukladno kodu časopisa

The Journal of Economic Literature (JEL).

`.	Uvod – sadrži definiranje problema i predmeta istraživanja s pozivom na recentnu literaturu odnosno rezultate istraživanja. Taj se dio može istaknuti i u posebnom, tj. 2., poglavlju kao Literature review. Pri kraju uvodnog dijela treba utvrditi radnu pretpostavku (hipotezu) istraživanja o kojoj se treba očitovati (kasnije) na početku poglavlja Zaključak. Uvod treba završiti s naznakom organizacije teksta.

`.	Pregled literature – prethodi istraživačkom dijelu, a pruža čitateljima pregled referentne literature s ključnim točkama dosadašnjih spoznaja temeljenih na relevantnim rezultatima aktualnih istraživanja. Pregled literature ne smije biti taksativno navođenje prethodnog znanstvenog doprinosa, već autori trebaju izvršiti sintezu dosadašnjih istraživanja kako bi dokazali opravdanost teorijskog i empirijskog doprinosa vlastitog rada.

`.	Metodologija/Metoda/Model/Koncepcija – obično se prezentira u trećem poglavlju; metoda/model/koncepcija analize mora biti transparentno istaknuta radi eventualnog ponavljanja testiranja rezultata od strane zainteresiranih istraživača (to je jedno od temeljnih pravila znanstvene metodologije).

`.	Empirijski podaci i analiza – sadržavaju dokumentacijsku podlogu i rezultate empirijske analize. Potrebno je opisati i prikazati uzorak podataka korišten u analizi te prezentirati i objasniti statistička te ekonometrijska obilježja dobivenih rezutata uz tumačenje njihova ekonomskog sadržaja.
`.	Rezultati i rasprava – autor objašnjava rezultate, osobito njihovo ekonomsko značenje i poruke. U ovom dijelu očekuje se argumentacija znanstvenog doprinosa, povezivanje rezultata rada s rezutatima te zaključcima dosadašnjih empirijskih istraživanja te preporuke za promjene javnih i drugih politika.

`.	Zaključci – treba imati u vidu da taj dio teksta nije i ne smije biti sažetak! Zaključci su autorovo originalno mišljenje (ocjena) o dobivenim rezultatima i obvezno sadrže:
	očitovanje o polaznoj hipotezi – je li ili nije dokazana;

	ocjenu rezultata istraživanja/analize, novine, te koji je doprinos znanosti;

	osvrt na ograničenja i probleme u istraživanju;


740	Guidelines for authors • Upute autorima		
	Zb. rad. Ekon. fak. Rij. • 2020 • vol. 38 • no. 2 • 723-744		

	smjernice za buduća istraživanja;

	utvrđivanje institucionalno-sustavnih implikacija dobivenih rezultata istraživanja­ (kao npr. prijedlozi za promjene u ekonomskom sustavu, ekonomsko­-financijskoj i razvojnoj politici, instrumenti, mjere i sl.).

Preporuka je da se Zaključak ne piše u odlomcima.

(3) Literatura – navesti samo one naslove (izvore) koji su korišteni u tekstu!

TABLICE dolaze unutar teksta. Svi podaci u tablici stavljaju se u redove i kolone odvojene jednostrukim tankim linijama. Svaka kolona sadrži naziv i uključuje numeričku jedinicu koja se odnosi na cijelu kolonu. Tako se za višecifrene brojeve rabe jedan ili dva broja, a u nazivu za određenu kolonu označuju se numeričke jedinice u tisućama, milijunima i sl. Tablice se numeriraju u kontinuitetu arapskim brojevima (Tablica 1, Tablica 2, itd.), a pored broja i naziva, moraju imati i izvor podataka.

Mole se autori da se pridržavaju sljedećih pravila:

	iza godine nikad ne dolazi točka;

	tisuće, milijuni i sl. odvajaju se zarezom: 2,000; 250,000; 3,555,284 i sl.; mili­ jarde se označavaju s bn (billion); decimalni brojevi odvajaju se točkom: 2.32;

0.35 i sl.

GRAFIKONI I SLIKE dolaze unutar samog teksta. Moraju imati broj, naziv i izvor podataka. Numerira ih se u kontinuitetu arapskim brojevima (posebno grafikoni, posebno slike). Izvori podataka navode se ispod grafikona­ odnosno slika.

Napomena. U tekstu se ne prepričavaju i ne ponavljaju rezultati koji su navedeni u tablicama i grafikonima, već se rabe referentne oznake koje upućuju na podatke u tablicama ili grafikonima.

REFERENCE U TEKSTU. Citirane dijelove navodi se u tekstu, a ne u bilješkama.

Stavlja ih se u zagrade i sadrže prezime autora i godinu izdanja npr. (Babić, 2003), a u slučaju citata navodi se još i stranica (Babić, 2003: 150), ili ako se radi o dvojici autora: (Babić i Courty, 2004), ili ako je više od dva autora navodi se prvi i piše: (Babić i suradnici, 2003). Svaka referenca navodi se kao i prvi put. Ne koristi se ibid i sl. Ukoliko se autor spominje u tekstu, u zagradi se navodi samo godina: The work of Stevens (2001) was concerned with what they teach at Harvard Business School. Svaka referenca mora se navesti u dijelu Literatura na kraju članka.

LITERATURA obuhvaća sve korištene izvore i potpune podatke o djelima koja se spominju u referencama u tekstu. Popis literature piše se bez broja poglavlja i dolazi na kraju rada (poslije Zaključka). Literatura se ne numerira. Uređuje se abecednim­ redom autora te kronološki za radove istog autora. Preporuča se autorima kao literaturu što više koristiti časopise referirane od strane ISI (Institute of Science

Information).


Guidelines for authors • Upute autorima	741		
Zb. rad. Ekon. fak. Rij. • 2020 • vol. 38 • no. 2 • 723-744			

Literatura se citira prema primjerima za knjige, časopise i ostale izvore:

•  Knjige: Prezime, Inicijali (godina) Naslov, Mjesto izdavanja: Ime izdavača.

Primjer:

Mohr, L. B. (1996) Impact analysis for program evaluation, 2nd ed., London: Sage.

Ukoliko su dva ili tri autora, redom navesti njihova prezimena i inicijale (godinu)

Naslov, Mjesto izdavanja: Ime izdavača. Primjer:

Riddersträle, J., Nordström, K. (2004) Karaoke Capitalism Management for Mankind, Harlow: Pearson Education Ltd.

Perišin, I., Šokman. A., Lovrinović, I. (2001) Monetarna politika, Pula: Sveučilište u Rijeci, Fakultet ekonomije i turizma "Dr. Mijo Mirković".

Ukoliko su četiri ili više autora, navodi se prezime prvog autora nakon čega slijedi et al. Primjer:

Norton, M. B. et al. (1981) A People and a Nation – A History of the United States,

Boston: Houghton Mifflin Company.

• Časopisi: Prezime, Inicijali (godina) "Naslov članka", Naziv časopisa u kojem je objavljen, volumen, svezak, broj, stranice. Primjer:

Fox, S. (1994) "Empowerment as a Catalyst for Change: An Example from the Food Industry", Supply Chain Management, Vol. 2, No. 3, pp. 29–33.
Ukoliko je više autora (četiri ili više), navodi se prezime prvog autora nakon čega slijedi et al. Primjer:

Di Noia, C. et al. (1999) "Should Banking Supervision and Monetary Policy Tasks be Given to Different Agencies?", International Finance, Vol. 2, No. 3, pp. 285–361.

Ukoliko je više radova istog autora objavljenih iste godine, uz godinu se rabe oznake "a, b, c":

Quah, D. T. (1993a) "Empirical Cross-section Dynamics in Economic Growth", European Economic Review, Vol. 37, No. 2–3, pp. 426–434.
---------- (1993b) "Galton's Fallacy and Tests of the Convergence Hypothesis",

Scandinavian Journal of Economics, 95, Vol. 95, No. 4, pp. 427–443.

---------- (1994) "Exploiting cross Section Variation for Unit Root Inference in Dynamic Data", Economics Letters, Vol. 44, No. 1–2, pp. 9–19.
---------- (1996a) "Empirics for Economic Growth and Convergence", European Economic Review, Vol. 40, No. 6, pp. 951–958.

---------- (1996b) "Regional Convergence Clusters across Europe", European Economic Review, Vol. 40, No. 6, pp. 951–958.

Autor za svaku referencu treba navesti Digital Object Identifier (DOI), ukoliko postoji. DOI je dostupan na adresi CrossRef-a http://www.crossref.org/ u obliku https://doi.org/10.5468/ogs.2016.59.1.1.

DOI autor upisuje na kraju reference prema primjeru:


742	Guidelines for authors • Upute autorima		
	Zb. rad. Ekon. fak. Rij. • 2020 • vol. 38 • no. 2 • 723-744		

Hall, J. K., Daneke, G. A. Lenox, M. J. (2010) "Sustainable Development and Entrepreneurship: Past Contributions and Future directions", Journal of Business Venturing, Vol. 25, No. 5, pp. 439–448, https://doi.org/10.1016/j.jbusvent. 2010.01.002.

• Izvori preuzeti s Internet stranica: Prezime autora/urednika, Inicijali imena (godina)­ "Naslov članka", Naslov Časopisa, datum publikacije, godište, broj, stranice, Internet adresa [datum pristupa].

Martin, C.L. (1998) "Relationship Marketing: a High-Involvement Product Attribute Approach, Journal of Product and Brand management, Vol. 7, No. 1, pp. 6–26, //http:www.apmforum.com/emerald/marketing-research-asia.htm [pristupljeno:

0.	10. 2002]

	Knjige sabranih dijela: Autor poglavlja/odjeljka (godina) "Naslov poglavlja/ odjeljka''. U Ime izdavača ili autora sabranih djela, Naslov sabranih djela, Mjesto izdavanja:­ Izdavač. Primjer:

Silobrčić, V. (2000) "Znanstvena proizvodnost i kriteriji vrednovanja znanstvenika­ u

Hrvatskoj''. U Sunko, U.D. (ur.) Znanost u Hrvatskoj na pragu trećeg tisućljeća,

Zagreb: HAZU.

• Radovi u Zborniku skupa: Autor (godina izdanja) "Naslov članka". U Naslov zbornika. Mjesto izdanja: Izdavač, stranice. Primjer:

Fedchak, E. & Duvall, L. (1996) "An engineering approach to electronic publishing". In Proceedings of the International Workshop on Multimedia Software Development,

25-26 March, Berlin, Los Alimos, Ca: IEEE Comput. Soc. Press, pp. 80–88.

• Teze i disertacije: navodi se Ime autora (godina) Naslov disertacije, Institucija gdje je doktorska disertacija obranjena. Primjer:

Whitehead, S.M. (1996) Public and private men: masculinities at work in edu­cation management, doktorska disertacija, Leeds Metropolitain University.

•  Službene publikacije: Naziv publikacije/organizacije/ustanove (godina) Naslov,

Mjesto­ izdavanja: Izdavač. Primjer:

Department of the Environment (1986) Landfilling wastes, London: HMSO (Waste management paper, 26)

Ostali prilozi u Časopisu

Ostali prilozi dostavljaju se na isti način kao i članci. Ne recenziraju se, a Urednički odbor ih ocjenjuje i razvrstava u sljedeće vrste priloga:

• Prikazi knjiga. Kritička recenzija pisana jasnim i konciznim stilom u kojoj se procjenjuje struktura, stil i znanstvena dostignuća knjige. U naslovu Prikaza navodi se naslov knjige. Ispod toga osnovni podaci o autoru knjige (titula, ustanova u kojoj je autor zaposlen), naslov i podnaslov rada (ukoliko postoji), godina izdavanja, izdavač, broj strana, vrsta izdanja, jezik pisanja, ISBN, e-mail autora knjige te naslovnica knjige. Autor Prikaza potpisuje se na kraju. Uz ime autora prikaza navodi se i ustanova u kojoj je zaposlen.


Guidelines for authors • Upute autorima	743		
Zb. rad. Ekon. fak. Rij. • 2020 • vol. 38 • no. 2 • 723-744			

	Prikazi doktorskih disertacija. U naslovu Prikaza navodi se autor disertacije. Ispod toga osnovni podaci o njemu (titula, ustanova u kojoj je zaposlen), naslov i podnaslov rada (ukoliko postoji) te članovi komisije za obranu doktorske disertacije. Dalje se navodi datum, godina te ustanova i mjesto obrane. U kritičkom prikazu opisuje se struktura, stil i vrednuju metodologija i rezultati istraživanja. Analiziraju se teoretski i praktični doprinosi u određenom znanstvenom području. Autor prikaza potpisuje se na kraju. Uz njegovo navodi se i ustanova u kojoj je zaposlen.

	Prikazi konferencije ili drugih skupova. Pišu se kao kritičke recenzije. U naslovu prikaza navode se: Naziv konferencije, organizator, datum održavanja, mjesto održavanja, jezik konferencije, kontakt osoba, e-mail, web stranica, podatak o materijalu s konferencije. Prikaz daje jasan i koncizan pregled glavnih ciljeva konferencije, imena glavnih izlagača te diskusije sudionika o znanstvenim dostignućima, rezultatima­ istraživanja i prijedlozima za daljnja istraživanja o ključnim pitanjima. Autor prikaza potpisuje se na kraju. Uz njegovo ime navodi se i ustanova u kojoj je zaposlen.

	In Memoriam. Ovaj prilog piše se na jednoj strani. Autor prikaza potpisuje se na kraju. Uz njegovo ime navodi se i ustanova u kojoj je zaposlen.

	Pisma Uredniku. Za čitatelje i autore izdvojen je zaseban prostor za komentare, sugestije, diskusije i mišljenja.

Ostale važne napomene Uredništva

Uredništvo pridržava pravo da tekstove koji ne odgovaraju kriterijima uputa vrati autoru, odnosno da radove u potpunosti prilagodi propozicijama Zbornika i standardima hrvatskog književnog jezika (u dijelu Sažetak), odnosno stranog jezika.

U pogledu ostalih tehničkih elemenata uređivanja tekstova za autore ne postoje posebni zahtjevi. Uredništvo sve članke ujednačuje.

Konačnu odluku o objavljivanju članaka kao i redoslijed članaka, određuje Uredništvo Zbornika.

Autor dobiva jedan primjerak Zbornika u kojem je njegov rad objavljen.

Preporučamo autorima da se registriraju (https://orcid.org/signin) i pribave ORCID identifikator. ORCID identifikator je jedinstveni i trajni identifikator istraživača i suradnika čije korištenje omogućava bolju vidljivost autora i interoperabilnost

širokog kruga informacijskih sustava.

Izdavač ne naplaćuje pristojbu za prijem članka. Ukoliko je rad autora prihvaćen, obveza je autora platiti pristojbu za objavljivanje (285 EUR) i to prije objavljivanja rada. Obavijest o načinu plaćanja autor dobiva nakon prihvaćanja rada. Autor je dužan snositi bankarske troškove.


744	Guidelines for authors • Upute autorima		
	Zb. rad. Ekon. fak. Rij. • 2020 • vol. 38 • no. 2 • 723-744		

Korektura

Rad autora mora biti pisan standardnim jezikom i bez pravopisnih i gramatičkih pogrešaka. Autor dobiva probni otisak rada na korekturu. Taj postupak treba obaviti­ u najkraćem roku i Uredništvu vratiti ispravljeni tekst. Ispravljati se mogu samo tiskarske pogreške.

Autorska prava

Članak poslan u naš časopis mora biti autentičan i izvorni doprinos autora i nikad prije objavljen niti smije istovremeno biti poslan u neki drugi časopis da bi se izbjeglo dvostruko objavljivanje.

Jednom prihvaćeni članak za objavljivanje obvezuje autora da isti članak ne smije objaviti drugdje bez dozvole Uredništva časopisa koje je članak prihvatilo. U slučaju da je Uredništvo dalo dozvolu za objavljivanje u drugom časopisu, treba navesti da je članak prethodno objavljen u časopisu Zbornik radova Ekonomskog fakulteta u Rijeci: časopis za ekonomsku teoriju i praksu/Proceedings of Rijeka Faculty of Economics: Journal of Economics and Business.

Svaki rukopis, radi izbjegavanja plagijarizma, provjerava se koristeći CrossCheck Service.

Autori jamče da objavljivanje njihovog članka ne predstavlja kršenje autorskih prava i da će obeštetiti izdavača ukoliko dođe do kršenja toga jamstva. U cilju širenja znanstvenih doprinosa i etičkih načela korištenja, prihvaćanjem radova za objavljivanje, izdavač postaje nositelj autorskih prava ukoliko u sporazumu nije navedeno drukčije.

Dostavljanje radova

Radovi se dostavljaju elektronskom poštom uz dopis koji sadrži: naslov članka, ime i prezime autora ili, ako je više koautora, za svakog ponaosob znanstveno zvanje, stručnu spremu, znanstveni interes, odnosno područje kojim se autor bavi, naziv i adresu institucije u kojoj je autor zaposlen, broj telefona, broj faksa, e-mail adresu i osobnu web stranicu. Svi navedeni podaci moraju biti napisani:

–  na jeziku članka,

–  na hrvatskom jeziku, te

–  na engleskom jeziku ako izvorni jezik članka nije engleski.

Adresa za dostavu radova je: zbornik@efri.hr

Detaljnije informacije o Zborniku radova Ekonomskog fakulteta u Rijeci: časopis za ekonomsku teoriju i praksu/Proceedings of Rijeka Faculty of Economics: Journal of Economics and Business nalaze se na web stranicama Ekonomskog fakulteta: http://www.efri.uniri.hr/hr/zbornik-radova.

UREDNIŠTVO
