# Supplementary material for: Peer Review in Law Journals
Source: Front Res Metr Anal. 2021 Dec 8;6:787768. doi: 10.3389/frma.2021.787768 (PMC8692876; doi:10.3389/frma.2021.787768)
Supplement: Supplementary file 3 [file DataSheet2.ZIP › DOCUMENT - 1331-9914.RTF]

Etički kodeks uredništva časopisa Poredbeno pomorsko pravo 

Časopis Poredbeno pomorsko pravo = Comparative Maritime Law je značajna publikacija u području pomorskoga prava i međunarodnog prava mora u Hrvatskoj i šire. Objavljuje recenzirane članke, stručne prijevode, prikaze sudske i arbitražne prakse, prikaze knjiga i povremeno ostale stručne i znanstvene osvrte i informacije. 

Nakladnik je Hrvatska akademija znanosti i umjetnosti – Jadranski zavod. 

Standardima etičkog postupanja pri objavljivanju radova obuhvaćene su sve strane: glavni i odgovorni urednik i uredništvo časopisa, recenzenti i autori. 

Naš Etički kodeks je sastavljen u skladu sa Smjernicama za kodeks ponašanja i najbolju praksu urednika časopisa COPE (2011.), Smjernicama o načelima transparentnosti i najboljoj praksi u znanstvenom publiciranju COPE (2015.) i Pravilnikom o izdavačkoj djelatnosti Hrvatske akademije znanosti i umjetnosti (2007.). 

1. DUŽNOSTI I OBVEZE UREDNIKA I UREDNIŠTVA 
Nakladnik imenuje glavnog i odgovornog urednika koji saziva uredništvo kao tijelo sastavljeno od članova s primjerenom kvalifikacijom, koji mogu aktivno pridonijeti razvoju i dobrom upravljanju časopisom. 
Glavni urednik je odgovoran za odlučivanje o temama i vrstama članaka koji će biti objavljeni u časopisu. Odluke donosi temeljem kriterija kvalitete i značaja članka za znanstvenike i čitatelje. Uz glavnog urednika, odgovornost ima i njegov zamjenik kada vrši njegovu funkciju. 
Rukopisi se ocjenjuju prema njihovom intelektualnom sadržaju bez obzira na rasu, spol, spolnu orijentaciju, vjerska uvjerenja, nacionalnu pripadnost, državljanstvo ili politička uvjerenja autora. 
Uvažava se sloboda izraza, poštuje cjelovitost i integritet znanstvenih ideja, uz poštivanje propisa koji se odnose na klevetu, povredu autorskog prava i plagiranje. U slučaju da dođe do objavljivanja pogrešnih, netočnih ili dvosmislenih tvrdnji, uredništvo će uz dužnu pažnju osigurati pravovremenu objavu ispravka. Glavni i odgovorni urednik se savjetuje s članovima uredništva i recenzentima koje imenuje Razred za društvene znanosti Hrvatske akademije znanosti i umjetnosti. Uredništvo ne smije otkriti informacije o zaprimljenim rukopisima nikome osim autoru, recenzentima, potencijalnim recenzentima, ostalim uređivačkim savjetnicima i izdavaču, i to na prikladan način. Uredništvo ne smije iskoristiti neobjavljeni materijal sadržan u zaprimljenom rukopisu, osim uz poseban pisani pristanak autora. Glavni i odgovorni urednik odgovara autorima za održavanje integriteta i tajnosti autorskog rada tijekom procjene rada za publiciranje.. Glavni i odgovorni urednik ne smije imati osobne, novčane ili druge odnose koji bi ga doveli u sukob interesa u pogledu njegovih uredničkih odluka. Zadaci i dužnosti članova uredničkih tijela: podržavanje i promoviranje časopisa, predrecenziranje predanih radova za objavu u časopisu, traženje najboljih autora i radova, pisanje uvodnika, prisustvovanje i doprinos zajedničkim sastancima. Glavni i odgovorni urednik je dužan konzultirati se s članovima uredništva najmanje jednom godišnje radi savjetovanja o vođenju časopisa i eventualnim promjenama i poboljšanjima u politici časopisa, uzimajući u obzir nove stručne i znanstvene kriterije o uređivanju i izdavanju časopisa. Uredništvo treba nastojati udovoljiti potrebama čitatelja i autora, planirati budućnost svojega časopisa i održavati visoki standard kvalitete radova. Postupak imenovanja recenzenata i objavljivanja pojedinih priloga u časopisu Poredbeno pomorsko pravo propisan je Pravilnikom o izdavačkoj djelatnosti Hrvatske akademije znanosti i umjetnosti kojeg se Uredništvo dužno pridržavati. 

2. DUŽNOSTI I OBVEZE RECENZENATA 
Recenzenti pomažu glavnom i odgovornom uredniku u donošenju uredničke odluke te posredstvom komunikacije urednika s autorom mogu pomoći autoru u poboljšanju sadržaja članka. Svaki izabrani recenzent, koji se smatra kvalificiranim za reviziju podnesenog rukopisa, treba se odazvati na poziv u razumnom roku i u dogovoreno vrijeme poslati recenziju prihvaćenoga rukopisa. Ako zna da to neće biti moguće, treba o tome obavijestiti uredništvo i isključiti se iz postupka recenziranja. Recenzent treba obavijestiti Uredništvo u slučajevima za koje bi mogao postojati sukob interesa (primjerice zaposlenje na istoj ustrojbenoj jedinici ustanove, osobne veze ili financijski interesi, ili bilo koja druga okolnost zbog koje se može dovesti u pitanje nepristranost recenzenta). O mogućem sukobu interesa tada odlučuje glavni i odgovorni urednik. Sa zaprimljenim rukopisima recenzenti moraju postupati kao s povjerljivim dokumentima. Rukopisi se ne smiju pokazivati niti se o njima smije raspravljati s drugim osobama, osim uz dozvolu uredništva, te ih se ni u kojem slučaju ne smije upotrijebiti za osobnu korist. Svi znanstveni i stručni članci imaju anonimnu, dvojnu istorazinsku recenziju, u skladu s Pravilnikom o izdavačkoj djelatnosti Hrvatske akademije znanosti i umjetnosti. Recenzenti ne smiju kontaktirati s autorima direktno, već posredstvom uredništva časopisa. Postupak recenziranja treba provoditi objektivno. Recenzenti trebaju izraziti svoje mišljenje jasno i argumentirano i po potrebi dodati konstruktivne primjedbe kako bi pomogli autorima poboljšati radove. Recenzent treba upozoriti Uredništvo na svaku značajniju sličnost ili preklapanje teksta predmetnog rukopisa i objavljenih članaka koji su mu poznati. 

3. DUŽNOSTI I OBVEZE AUTORA Autor pojedinog priloga za časopis može biti jedna ili više osoba koje su značajno doprinijele oblikovanju i interpretaciji glavnih ideja rada i svih rezultata istraživanja. Autori trebaju iznositi točne podatke, objektivno raspraviti problem istraživanja i ukazati na značaj predmeta istraživanja. Članak treba sadržavati dovoljno detalja i korištene izvore, kako bi ostali znanstvenici mogli provjeriti iznesene tvrdnje i rezultate istraživanja. Namjerno prikazivanje netočnih podataka predstavlja neetično postupanje i nije prihvatljivo. Autori moraju sve korištene podatke po potrebi dati na uvid za potrebe recenziranja i čuvati ih u razumnom razdoblju nakon objave članka. Autorima treba biti omogućena sloboda izražavanja, a oni moraju osigurati izvornost sadržaja članka. Ako su u izradi članka koristili radove i/ili sadržaj članaka drugih autora, moraju ih primjereno citirati. Autor ne bi smio objaviti rukopis, koji se odnosi na isto istraživanje, istovremeno u više od jednog časopisa. Može postojati izuzetak, uz odobrenje uredništva i uz napomenu gdje je rad izvorno objavljen. Autori moraju biti upoznati sa standardima objavljivanja časopisa, licencom i otvorenim pristupom, te urediti i dostaviti svoj rukopis u skladu s Uputama za autore, a o svemu se mogu informirati na web stranicama časopisa: http://hrcak.srce.hr/poredbeno-pomorsko-pravo, http://jadranski-zavod.hazu.hr/. 


Molimo recenzente da svoje kritičke primjedbe na rad obrazlože i da prilikom ocjene rada po najstrožim kriterijima ocijene:
0)	znanstvenu vrijednost rada;
0)	metodološki pristup;
0)	originalnost rada.

Kategorizacija rada:	1.	Izvorni znanstveni članak	
ISPUNJAVA RECENZENT	2.	Prethodno priopćenje	
	3.	Pregledni članak	
	4.	Izlaganje sa znanstvenog skupa	
	5.	Stručni članak	
			
Prijedlog recenzenta da se rad	1.	Da se rad objavi u sadašnjem obliku	
preporučuje za objavljivanje:	2.	Da se rad objavi uz doradu	
ISPUNJAVA RECENZENT	3.	Da se rad nakon dorade ponovno dostavi recenzentu	
	4.	Da se rad ne prihvati za objavljivanje	
			

Komentar recenzenta
ISPUNJAVA RECENZENT

3
