# Supplementary material for: Peer Review in Law Journals
Source: Front Res Metr Anal. 2021 Dec 8;6:787768. doi: 10.3389/frma.2021.787768 (PMC8692876; doi:10.3389/frma.2021.787768)
Supplement: Supplementary file 3 [file DataSheet2.ZIP › DOCUMENT - 1332-0718.RTF]

Scientific Journal of Maritime Research - POMORSTVO	           	Sveučilište u Rijeci, Pomorski fakultet 
University of Rijeka, Faculty of Maritime Studies 

REVIEWER ASSESSMENT FORM
	

Reviewer: Click or tap here to enter text.

Author(s): Click or tap here to enter text.

Title: Click or tap here to enter text.

Information about the manuscript:

Number of pages: Click or tap here to enter text.	Number of figures: Click or tap here to enter text.        Number of sections: Click or tap here to enter text.	

Number of tables: Click or tap here to enter text.    Number of references: Click or tap here to enter text.

Reference list: Click or tap here to enter text.

The topic has been raised:        a) often ☐     b) rarely ☐     c) never  ☐

The presented arguments contain evidence of current practice and/or research  Choose an item.

Theoretical assumptions, models, and formulas are explicitly discussed.		 Choose an item. 

The author has an axe to the grind. The manuscript offers a new, original, and significant insight, and is clear to the intended audience. The manuscript makes a significant (practical, useful, plausible) contribution to the field. 											Choose an item.

The manuscript reflects sound scholarship and research design with appropriate, correctly interpreted references to other authors and works.  						Choose an item.

The presented major research findings are clearly presented and can be tested. 
											Choose an item.

The specialized terminology is usefully defined and correctly applied.	 	Choose an item.

The manuscript appeals to the general interests of the Scientific Journal of Maritime Research.
											Choose an item.

The content of the manuscript is accessible to the broad readership of the Scientific Journal of Maritime Research, not only to the specialists in the area addressed.			Choose an item.

The manuscript is well-structured, the title and section headings reflect the content and logically follow.
											Choose an item.

The conclusion is well-grounded and logically drawn.				Choose an item.

The summary reflects the main constructs, problems, and findings.		Choose an item.

Does the manuscript contain indicators of plagiarism or un-cited sources?	a) Yes ☐     b) No ☐

Scientific Journal of Maritime Research - POMORSTVO


The methodology is:
`)	explicit and clear			☐
`)	implicit and satisfactory		☐
`)	modest and substantially flawed	☐

The manuscript has a clearly stated purpose and point of view. The objectives are reached:
`)	completely   ☐
`)	partly   ☐

Strengths and weaknesses (suggestions for improvements):
Click or tap here to enter text.


Manuscript category:
`)	original scientific paper   ☐				
`)	preliminary communication   ☐
`)	review article   ☐
`)	technical paper    ☐

Assessment: The manuscript is:
`)	recommended for publication   ☐
`)	substantially flawed – needs improvement   ☐
`)	has many deficiencies, inadequate for publication   ☐

Reviewers Conflict of Interest Statement
A “conflict of interest” refers to any current professional, financial, commercial, legal or other interest which could: 
?	significantly impair the individual’s objectivity in carrying out his or her duties and responsibilities regarding the reviewer's process; 
?	create an unfair advantage for any person or organization;
?	having a personal relationship (family or close friends) with the author(s).
For this policy, circumstances that could lead a reasonable person to question an individual’s objectivity, or whether an unfair advantage has been created, constitute a potential conflict of interest. 
It is not considered a Conflict of Interest if the reviewers have worked together with the authors in a collaborative project (e.g. IAMU projects, Croatian science foundation projects, university projects, national scientific research projects, international scientific projects) or if they have co-organized an event (e.g PC chair/co-chairs/session chairs…).

Place:   Click or tap here to enter text.
Date:    Click or tap to enter a date.

							
									Reviewer's signature

							  _______________________________________


Scientific Journal of Maritime Research - Pomorstvo	
	
tel: ++385 51 338 411	         Studentska 2, 51000 Rijeka, Croatia	e-mail: casopis@pfri.hr                      http://www.pfri.hr/pomorstvo/	
