# Supplementary material for: Peer Review in Law Journals
Source: Front Res Metr Anal. 2021 Dec 8;6:787768. doi: 10.3389/frma.2021.787768 (PMC8692876; doi:10.3389/frma.2021.787768)
Supplement: Supplementary file 3 [file DataSheet2.ZIP › DOCUMENT - 1332-0718_1.RTF]

Scientific Journal of Maritime Research –

POMORSTVO


INSTRUCTIONS TO REWIEVERS


Paper reviewers of the journal Pomorstvo are prominent scientists.

Reviewers submit their opinion and evaluation of the paper. The deadline for submission of reviews is fifteen days. The reviewer may request an extension of this deadline for another fifteen days.

The review process is uniquely secret and confidential. The authors of the paper do not know the names of the reviewers (single-blind "review").

Upon receipt of the paper, it is addressed to the Editorial Board and / or the Editor-in-Chief, who decides on the admissibility of the paper. If the paper meets the criteria of the Journal, it will be sent to reviewers. Each paper will be reviewed by two or three reviewers.

The work can be accepted or rejected. Categorization of papers: original scientific paper, preliminary communication, review paper, and professional paper. Professional papers are not published in the Journal.

Each paper should have at least one positive review. In case of two negative reviews the author will be notified of non-acceptance of the paper. In case the paper has one negative review, the paper will be referred for additional review.

Proposals and suggestions of reviewers will be forwarded to the correspondent who is obliged to send the corrected work or comment on the remarks and suggestions of the reviewers. The authors are not familiar with the names of the reviewers.

In the case when the paper is differently categorized by the reviewers, the final decision on the categorization of the paper is made by the editor-in-chief.

COPE Ethical Guidelines for Peer Reviewers
