# Supplementary material for: Peer Review in Law Journals
Source: Front Res Metr Anal. 2021 Dec 8;6:787768. doi: 10.3389/frma.2021.787768 (PMC8692876; doi:10.3389/frma.2021.787768)
Supplement: Supplementary file 3 [file DataSheet2.ZIP › DOCUMENT - 1332-8670.RTF]

Pravni portal - ingbiro.com 
ingbiro.com 
·	PROPISI 
o	Jednostavni pretraživačo	 
o	Složeni pretraživačo	 
o	Zadnje NNo	 
o	Mjesečni pregled propisao	 
o	Područja pravao	 
o	Pojmovno kazaloo	 
o	Urednički pročišćeni tekstovio	 
o	Propisi EU - PRETRAŽIVAČo	 
o	SL EU - posebno izdanje 2013.o	 
o	EUR-Lex - PRETRAŽIVAČo	 
·	SUDSKA PRAKSA 
o	Jednostavni pretraživačo	 
o	Složeni pretraživačo	 
o	Zadnje odlukeo	 
o	Područja pravao	 
o	Nomenklaturno kazaloo	 
o	Odluke EU sudovao	 
o	Arhivao	 
·	STRUČNI ČLANCI 
o	Jednostavni pretraživačo	 
o	Složeni pretraživačo	 
o	Ovogodišnji e-HPRo	 
o	Zadnji brojo	 
o	Područja pravao	 
o	Pojmovno kazaloo	 
o	Autorio	 
o	Arhivao	 
o	Zbornici - PRETRAŽIVAČo	 
o	Zbornici po godinamao	 
o	Upute autorimao	 
·	UGOVORI 
o	Obrasci PUo	 
o	Obrasci - radno pravoo	 
o	Obrasci - obvezno pravoo	 
o	Obrasci - stečajno pravoo	 
·	MIŠLJENJA 
o	Mišljenja MRMSo	 
o	Mišljenja MGPOo	 
o	Mišljenja MGPUo	 
o	Mišljenja PUo	 
o	Mišljenja AZOPo	 
o	Mišljenja AZTNo	 
o	Mišljenja HAKOMo	 
o	Mišljenja HANFAo	 
o	Mišljenja CUo	 
o	Mišljenja HOKo	 
o	Odluke DKOMo	 
o	Tumačenja kolektivnih ugovorao	 
o	Q/A - PRETRAŽIVAČo	 
o	Q/A - područja pravao	 
·	KALKULATORI 
o	Izračun sudskih pristojbio	 
o	Izračun plaćao	 
o	Izračun ugovora, honorara, naknadao	 
o	Izračun zakonskih zateznih kamatao	 
5 kategorija sadržaja 
Interaktivno su povezani. 
 Više  

Fer cijena. Za godinu dana. 
Neograničeno pretraživanje. Bez skrivenih troškova. 
 Pretplatite se  

Fleksibilni smo. 
Pretplatite se na puni paket. Ili samo na pojedinačne sadržaje. 
 Pretplatite se  

Zakonodavstvo na dodir prsta 
 
Najveći portal pročišćenih i povezanih pravnih informacija: od važećih propisa i sudskih odluka RH i EU, pročišćenih tekstova zakona do stručno-znanstvenih članaka i korisnih pravnih sadržaja. ...više 
Kako koristiti Pravni portal ingbiro.com    
Postanite korisnik 
 
Najbolji način da se uvjerite u prednost korištenja više od 1 600 000 provjerenih, ažuriranih i povezanih pravnih sadržaja jest da sami probate koristiti Pravni portal ingbiro.com. Zatražite probnu lozinku! 
Postanite korisnik Pravnog portala ingbiro.com
Prijava 
  **

Upute autorima
 
- za objavljivanje radova u časopisu Hrvatska pravna revija (HPR)
HRVATSKA PRAVNA REVIJA je časopis u kojem znanstvenici i afirmirani praktičari mogu iznijeti svoje zamisli, kritike, iskustva i prijedloge o raznim pitanjima primjene prava. 
U časopisu se objavljuju radovi koji se recenziraju i oni koji ne podliježu recenzentskom postupku. Recenzirani radovi kategoriziraju se u ove kategorije:
·	izvorni (originalni) znanstveni članak - original scientific paper 
·	prethodno priopćenje - preliminary communication 
·	pregledni znanstveni članak - review 
·	izlaganje (referat) sa znanstvenog skupa - conference paper 
·	stručni članak - professional paper. 
Izvorni znanstveni članak sadrži dosad još neobjavljene rezultate izvornih istraživanja. Prethodno priopćenje sadrži nove rezultate znanstvenih istraživanja, koji zahtijevaju brzo objavljivanje. Pregledni znanstveni članak mora biti originalan, sažet i kritički prikaz jednog područja ili njegova dijela u kojem autor i sam aktivno sudjeluje. Mora biti istaknuta uloga autorova izvornog doprinosa u tom području s obzirom na već publicirane radove, kao i pregled tih radova. Izlaganje sa znanstvenog skupa, prethodno referirano na takvom skupu, mora biti objavljeno u obliku cjelovitog članka, i to samo ako nije prije toga objavljeno u zborniku skupa. Stručni članak sadrži korisne prijedloge iz određene struke i ne mora sadržavati izvorna istraživanja. 
Konačnu odluku o kategorizaciji pojedinog rada donosi Uredništvo. 
Pri dostavi radova za objavu u časopisu Hrvatska pravna revija molimo autore da se pridržavaju sljedećih pravila:
0.	Rad ne smije biti već objavljen ili u tom cilju upućen drugom časopisu; 
0.	Rad se dostavlja e-mailom na adresu glavnog urednika (abijelic@ingbiro.hr0.	), odnosno na adresu ingbiro@ingbiro.hr 0.	, a iznimno i na CD-u; 
0.	Opseg rada ne smije prelaziti osam kartica (oko 12000 znakova). Rad koji prelazi zadani opseg neće se objavljivati, osim prema posebnoj odluci Uredništva; 
0.	Naslov mora biti koncizan i istovremeno informativan; 
0.	U lijevom kutu naslovne stranice rukopisa treba stajati ime i prezime autora, akademski i stručni naziv odnosno akademski stupanj autora te naziv i adresa ustanove u kojoj radi; 
0.	Rad treba biti pripremljen u što je moguće jednostavnijoj formi, pri čemu je potrebno izbjegavati posebne formate, poravnavanja, uvlake, tabove, prazne redove, nepotrebne razmake, automatska nabrajanja, podnožja, zaglavlja i slično; 
0.	 Propise koji se navode u tekstu autori su dužni označiti punim nazivom propisa i službenoga glasila u kojem je objavljen, sa svim daljnjim izmjenama i dopunama te naznakom kako će se propis navoditi u daljnjem tekstu, u punom nazivu ili skraćenom obliku. Jednom navedeni brojevi službenoga glasila više se ne navode, osim ako je riječ o izmjenama i dopunama koje se posebno komentiraju. 
0.	Ako su u radu uporabljene kratice i simboli, uz rukopis je potrebno priložiti objašnjenje o njihovu značenju; 
0.	Tablice moraju biti pregledno i korektno priređene te opisane (broj, naslov, legenda); 
0.	Svi radovi osim prikaza moraju biti opremljeni sažetkom s maksimalno 150 riječi te ključnim riječima. Sažetkom, pisanim u trećem licu, na skraćen način predstavlja se sadržaj odnosno bitne odrednice članka; 
0.	Bilješke (fusnote) se koriste radi objašnjenja, komentara ili referenci teksta, a objavljuju se na dnu stranice; 
0.	Na kraju teksta abecednim redom navodi se literatura korištena u radu na sljedeći način: 
·	Knjige: 
- Bolanča, D., Delač, D., Frković, S., Jug, J., Kundih, B., Nakić, J., Seršić, V., Vukmanović, D., Pomorsko dobro, Inženjerski biro d.d., Zagreb, 2005., 
- Ćurković, M., Ugovor o osiguranju - komentar odredaba Zakona o obveznim odnosima, Inženjerski biro d.d., Zagreb, 2017. 
·	Časopisi: 
- Barbić, J., Pregled odredaba Zakona o trgovačkim društvima o monističkom ustroju organa dioničkog društva, Hrvatska pravna revija, 12/2007, str. 44, 
- Miladin, P., Markovinović, H., Pljenidba kreditnih zahtjeva po bankarskim računima, Pravo u gospodarstvu, 3/2007, str. 3. Literatura koja je već u cijelosti navedena u bilješkama, neće se ponovno navoditi u popisu literature; 
0.	Radovi se ne objavljuju prema redoslijedu pristizanja na adresu nakladnika, već, prije svega, ovisno o aktualnosti teme koju obrađuju, o čemu odluku donosi Uredništvo. O objavljivanju radova koji se ne objave u roku od šest mjeseci od zaprimanja, Uredništvo će se posebno dogovoriti s autorom. 
 

Kontakt 
 
Heinzelova 4a, 10000 Zagreb
HRVATSKA 
Telefon: (+385) 1 46 00 888 
Fax: (+385) 1 46 50 366 
Email: Prodaja
Newsletter: prijava / odjava
Inženjerski biro d.o.o. 
 
·	O nama·	 
·	O pravnom portalu·	 
·	Zatražite probni račun·	 
·	Pretplata·	 
·	Uvjeti korištenja·	 
·	Politika korištenja kolačića·	 
·	Politika zaštite privatnosti·	 
·	Korisničke upute·	 
Zašto izabrati nas? 
 
Zato što smo mi iskoristili mogućnosti interneta, ali nismo upali u zamku masovnosti neprovjerenih i nevažećih informacija - naš rad na portalu i njegov razvoj koncipira se na pomnoj profesionalnoj organizaciji, sistematizaciji i selekciji odabranih pravnih informacija. 
·	Postavite pitanje·	 
·	·	·	·	·	© Inženjerski biro d.o.o.
Na internetskim stranicama Pravnog portala ingbiro.com koriste se tehnički (nužni) kolačići (cookies), ne možemo ih isključiti te za korištenje takve vrste kolačića ne trebamo Vašu privolu. Svoj preglednik možete postaviti da blokira te kolačiće ili pošalje upozorenje o njima, ali u tom slučaju neki dijelovi stranice neće raditi. Pravni portal ingbiro.com funkcionira optimalno samo ako je omogućeno korištenje kolačića. 
Više o kolačićima možete saznati u Politici korištenja kolačića. 
Ako imate pitanja o zaštiti Vaše privatnosti, posjetite Politiku zaštite privatnosti. 
Razumijem 
