# Supplementary material for: Peer Review in Law Journals
Source: Front Res Metr Anal. 2021 Dec 8;6:787768. doi: 10.3389/frma.2021.787768 (PMC8692876; doi:10.3389/frma.2021.787768)
Supplement: Supplementary file 3 [file DataSheet2.ZIP › DOCUMENT - 1333-2546.RTF]

 
 
Open Journal Systems 
Pomoć 
Korisnik 
Korisničko ime		
Lozinka		
 Zapamti me	
	

Jezik / Language 
Odaberi jezik 
Veličina znakova 

Sadržaj časopisa 
Pretraživanje 
 	
##plugins.block.navigation.searchScope## 
	
	


Pregled 
·	Po broju časopisa·	 
·	Po autoru·	 
·	Po naslovu·	 
·	Naslovna·	 
·	O časopisu·	 
·	Prijava·	 
·	Registracija·	 
·	Pretraživanje·	 
·	Trenutni broj·	 
·	Arhiva·	 
Naslovna > O časopisu > Uređivačka politika 
Uređivačka politika
 
·	» Fokus i područje djelovanja·	 
·	» Rubrike·	 
·	» Recenzijski postupak·	 
·	» Učestalost izdavanja·	 
·	» Otvoreni pristup·	 
·	» Indeksiranje i referiranje·	 
·	» Cijena i pretplata·	 
Fokus i područje djelovanja
 
Migracije i etničnost aktualna su i afirmirana područja suvremenih društvenih istraživanja. Nove pojave i procesi u svijetu – globalizacija, sve veća prostorna pokretljivost ljudi, delokalizacija i relokalizacija kulture – razlog su sve većeg znanstvenoga zanimanja za pitanja migracija, etničnosti i identiteta. Istraživačima koji se bave tom tematikom časopis Migracijske i etničke teme otvara na svojima stranicama mogućnost razmjene ideja, javnih debata i interdisciplinarnoga dijaloga. 
Znanstveni časopis Migracijske i etničke teme prvenstveno objavljuje znanstvene radove o migracijama, problematici vezanoj uz migracije i demografske promjene te radove o različitim aspektima etničnosti i identiteta u međunarodnom, nacionalnom i međunacionalnom kontekstu. Prilozi donose teorijska dostignuća te empirijske analize i studije u širokom rasponu disciplinarnih pristupa. Kao internacionalan, časopis uistinu pokriva svojim interesom cijeli svijet; radovi se  objavljuju na hrvatskom i engleskom jeziku, a po posebnoj odluci uredništva i na drugim jezicima. Međunarodni karakter časopisa definiran je kako tematikom članaka tako i međunarodnim sastavom autora, recenzenata, uredništva i izdavačkoga savjeta.

U časopisu se objavljuju u prvome redu znanstveni radovi, ali i drugi prilozi od znanstvenoga i stručnoga interesa: eseji, stručni radovi, osvrti, prikazi i priopćenja o znanstvenim skupovima.
 
Zaprimanje i objavljivanje radova se ne naplaćuje.
 
Rubrike
 
ČLANCI
 
Uredništvo prima prethodno neobjavljene radove na hrvatskom ili engleskom jeziku. Rad zajedno s popisom literature i svim prilozima ne bi smio biti opsežniji od 57.600 znakova. Uz radove potrebno je priložiti bilješku o autoru (50–100 riječi) i sažetak (opsega od oko 250 riječi) na jeziku kojim je tekst pi­san i engleskom jeziku. Sa­žetak treba sadržavati opći prikaz teme, metodski pristup, glavne rezultate i zaključak. Iza sažetaka treba navesti ključne riječi (do 7) na istim jezicima.
Otvoreno za prijave	Katalogizirano	Recenzijski postupak	
OSVRTI
 
Recenziraju se domaća i strana djela ne starija od tri godine. Na početku teksta se navode svi podaci prikazanog djela dok se autor potpisuje na kraju rada. Poželjan opseg osvrta iznosi do 36.000 znakova.
Otvoreno za prijave	Katalogizirano	Recenzijski postupak	
PRIKAZI
 
Časopis prihvaća prikaze hrvatskih i stranih djela ne starijih od tri godine. Poželjan opseg prikaza iznosi od 5.400 do 9.000 tisuća znakova.
Otvoreno za prijave	Katalogizirano	Recenzijski postupak	
SKUPOVI
 
Časopis prihvaća prikaze hrvatskih i stranih znanstvenih skupova ne starijih od godinu dana. Poželjan opseg prikaza iznosi od 5.400 do 9.000 tisuća znakova.
Otvoreno za prijave	Katalogizirano	Recenzijski postupak	
PRIJEVOD
 
Časopis povremeno prevodi na hrvatski jezik posebno značajne ili zanimljive radove renomiranih inozemnih autora izvorno objavljenih na stranom jeziku.
Otvoreno za prijave	Katalogizirano	Recenzijski postupak	
BIBLIOGRAFIJA
 
Otvoreno za prijave	Katalogizirano	Recenzijski postupak	
 
Recenzijski postupak
 
Svi radovi poslani Migracijskim i etničkim temama prolaze kroz recenzijski postupak u kojemu dva anonimna recenzenta procjenjuju rad (dvostruko slijepa recenzija). Uredništvo će autore u primjerenom roku izvijestiti o rezultatima recenzijskog i uredničkog postupka. Uredništvo pridržava pravo prilagodbe rada uredničkim propozicijama i standardima hrvatskoga književnog jezika.

 
 
Učestalost izdavanja
 
Znanstveni časopis Migracijske i etničke teme izlazio je tromjesečno od 1985.–2009. (do 2000. pod nazivom Migracijske teme: časopis za istraživanje migracija i narodnosti). Od 2010. do 2020. izlazio je u tri broja godišnje, a od 2021. u dva broja godišnje.
 
Otvoreni pristup
 
Časopis omogućava otvoren pristup svojem cjelokupnom sadržaju. Institut za migracije i narodnosti kao izdavač podržava Hrvatsku deklaraciju o otvorenom pristupu.
 
Indeksiranje i referiranje
 
Tekstovi objavljeni u časopisu referiraju se u: 
CEEAS – Central & Eastern European Academic Source (EBSCO, Ipswich, USA)
CEEOL – Central and Eastern European Online Library (Frankfurt am Main, Germany)
DOAJ – Directory of Open Access Journals (Lund University, Sweden)
ERIH PLUS – European Reference Index for the Humanities and the Social Sciences (Bergen, Norway)
International Bibliography of the Social Sciences (London, UK)
Linguistics and Language Behaviour Abstracts (San Diego, USA)
Political Science Complete (EBSCO, Ipswich, USA)
SocINDEX with Full Text (EBSCO, Ipswich, USA)
Sociological Abstracts (San Diego, USA)
Social Services Abstracts (ProQuest, Ann Arbor, USA)
Worldwide Political Science Abstracts (San Diego, USA)
 
Cijena i pretplata
 
Godišnja pretplata: za pojedince 50 kn, za ustanove 100 kn, za inozemstvo 60 € (zračnom poštom 70 €).
 


Migracijske i etničke teme / Migration and Ethnic Themes. ISSN 1333-2546; E-ISSN 1848-9184; DOI: 10.11567/met 
