# Supplementary material for: Peer Review in Law Journals
Source: Front Res Metr Anal. 2021 Dec 8;6:787768. doi: 10.3389/frma.2021.787768 (PMC8692876; doi:10.3389/frma.2021.787768)
Supplement: Supplementary file 3 [file DataSheet2.ZIP › DOCUMENT - 1333-2546_1.RTF]

﻿ 389 

GUIDELINES FOR CONTRIBUTORS 

The  Migracijske etničke    Migration Ethnic  ) publishes  scientific articles on migration, migration-related issues and demographic changes, together with articles on different aspects of ethnicity and identity in any international, national or cross-national context. Contributions reflect the theoretical advances and empirical analyses and studies within a broad range of disciplinary approaches. 
The journal publishes also other contributions of scientific and professional in -terest: essays, professional papers, book reviews, notes on scientific meetings, etc. 
Papers are published in Croatian and English, and the editorial board may also decide to publish some of them in other languages. 
All articles have undergone rigorous peer review, based on initial editor screen -ing and double-blind refereeing by a minimum of two anonymous review ers. 
The journal does not have article submission and article processing charges. 

Submission requirements 
Papers are to be submitted electronically using the  Open Journal System  acces -sible on the web page of the journal:  https://ojs.imin.hr/index.php/met/index . On the first page of the paper the author should write only his or her name, surname, and the name and address of the institution in which he or she is employed. It is also necessary to give an e-mail address, fax or telephone number. 
It is preferable that the length of articles be 28,800–57,600 characters, including spaces. Book reviews, as well as notes, reports on scientific meetings and other sub -jects of interest to the journal, should be 5400–9000 characters. 
Along with the paper a note on the author/authors (50–100 words) should be submitted and a summary (around 250 words) in the language of the paper and in  
English. When submitting article in non-English language, the author should addi -tionaly provide an extended summary (around 1500 words). The summary should contain the general presentation of the subject, the methods implemented, the main results and the conclusion(s). After the summaries, key words (up to seven) should be added in the same languages. 
Graphic material (tables, illustrations, graphs, etc.) should be submitted in black and white technique and positioned within the text in their appropriate places. All illustrations, graphs and diagrams should be submitted additionally (in an adequate resolution) in their original electronic format (.jpg, .gif, .xls, etc.). If such material is taken from other sources, the author is obliged to secure copyright permission. 

Publication ethic 
The journal is committed to promoting the highest ethical publication practices and to maintaining the integrity of the scientific record. It is not a member of the  
Committee on Publication Ethics − COPE , but it follows COPE’s  Code of   Conduct whose policies detail what is to be expected of the key participants in the publish -ing process: authors, reviewers, editors and publishers. Clear guidelines for con390

The journal publishes exclusively unpublished papers. By submitting a manuscript the authors confirm that it is the result of their own original work for which they accept scientific and ethical responsibility (especially with respect to plagia -rism, forgery of data, multiple reporting or publishing identical research results, abuse of authorship, or any other form of academic misdemeanour). Additionally, by submitting a manuscript the authors confirm that the same manuscript has not been published or submitted for publishing elsewhere. 

Citation and references 
References the should made the manner:  
1986), or (Berger and Luckmann, 1966: 91), etc. Abbreviations such as “ibid.”, “op.  
cit.” and similar should be avoided, if possible. All references should be cited in Roman script. 
References should be listed at the end of the paper in alphabetical order as follows: 

Book 
Delanty, G. and O’Mahony, P. (2002).  Nationalism and Social Theory: Modernity and the Recalcitrance of the Nation . London − Thousand Oaks − New Delhi: SAGE  
Publications. 

Article or chapter in an edited book  
Damon, (1982). Entwicklung sozialen des in:  
W. Edelstein and M. Keller (eds).  Perspektivität und Interpretation . Frankfurt am  
Main: Suhrkamp, 110–145. 

Article in a periodical 
Podgorelec, S. and Bara, M. (2014). Žensko iskustvo migracija i starenja – pogled s otokâ,  Migracijske i etničke teme , 30 (3): 379–404, doi:  https://doi.org/10.11567/ met.30.3.5 
Web source 
Cassarino, (2009). Mobility Expression a  
Compromise,   Migration Institute Migration Source,  
September http://www.migrationinformation.org/Feature/display. 
cfm?ID=741 (28 November 2010). 

Corporate author 
UNESCO (2006).  UNESCO Guidelines on Intercultural Education . Paris. 

If more references from one author are referred from the same year, letters are added to the date – e.g. (1991a), (1991b), etc. 

Copyright notice 
Copyright for articles published in this journal is retained by the authors, with first publication rights granted to the journal (this applies to both print and elec -tronic issue). By virtue of their appearance in this open access journal, articles are free to use, with correct citation, in educational and other non-commercial settings (in accordance with CC-BY-NC Licence). 
