# Supplementary material for: Peer Review in Law Journals
Source: Front Res Metr Anal. 2021 Dec 8;6:787768. doi: 10.3389/frma.2021.787768 (PMC8692876; doi:10.3389/frma.2021.787768)
Supplement: Supplementary file 3 [file DataSheet2.ZIP › DOCUMENT - 1575-6548_1.RTF]

Focus and Scope
The Spanish Journal of Political Science (RECP) welcomes articles, research notes, review articles and book reviews from authors affiliated to Spanish academic institutions and from other parts of the world, both in Spanish and English.
The RECP advocates a plurality of epistemological perspectives and encourages authors to present articles from distinct theoretical angles or methodologies to explain relevant empirical political phenomena. The evaluation and selection of articles, research notes and review articles for publication is based on criteria of quality, originality, relevance and methodological consistency. And it will be undertaken by specialists who are independent from the Journal, in line with the ‘double-blind’ methodology; a process that maintains the anonymity of both the authors and the evaluators. 
The range of studies and research covered by the RECP are:
`.	Political Change
`.	Political Behaviour
`.	Political Communication
`.	Political Economy
`.	Gender Studies
`.	Government Studies
`.	Electoral Studies
`.	International Studies
`.	Regional Studies
`.	Public Management
`.	Governance
`.	Political Ideologies
`.	Political Thought
`.	Comparative Politics
`.	Foreign Policy
`.	European Politics
`.	Latin American Politics
`.	Local Politics
`.	Public Policy
`.	Political Regimes
`.	Political Systems
`.	Security and Defense
`.	Political Sociology
`.	Political Theory
Publication Frequency
The Spanish Journal of Political Science (RECP) is published three times a year, in March, July and November, by the Spanish Association of Political Science and Public Administration (AECPA).
Open Access Policy
This journal provides immediate open access to its content on the principle that making research freely available to the public supports a greater global exchange of knowledge. There are no processing charges.
