# Supplementary material for: Peer Review in Law Journals
Source: Front Res Metr Anal. 2021 Dec 8;6:787768. doi: 10.3389/frma.2021.787768 (PMC8692876; doi:10.3389/frma.2021.787768)
Supplement: Supplementary file 3 [file DataSheet2.ZIP › DOCUMENT - 1576-4729_1.RTF]

About the Journal
"Historia Constitucional" is an electronic journal co-edited by the Centro de Estudios Políticos y Constitucionales of Madrid (an institution forming part of the Spain's Ministry of the Presidency), and the Seminario de Historia Constitucional "Martínez Marina" (University of Oviedo, Spain). The journal was initially supported by RedIris, an institution belonging to the Spanish Consejo Superior de Investigaciones Científicas (the main national institution for scientific research).
"Historia Constitucional" has renewed the historical and legal studies, not only because it is a journal is exclusively devoted to the constitutional history (which is very unusual indeed), but also because it is an open-access electronic journal. In fact, this is the first Electronic Journal dealing with constitutional history, not only in Spain but also in Europe and surely all around the world.

"Historia Constitucional", founded in 2000, is a yearly journal whose original submissions are dedicated to the constitutional history of any country and the related Public Law, Legal History, Political Thought, Modern and Contemporary History and Political Science.

"Historia Constitucional"has a rigorous standard regarding the acceptance of articles for publication. The journal approves summisions in Spanish, English, Portuguese, German, French and Italian on constitutional history. All material is submitted anonymously to referees selected among specialists in that particular field.
"Historia Constitucional" will have regular sections (“Studies” and “Reviews”), but it also include, when possible, a monographic section. It also include interviews to constitutional historians, information about the activity of Centers specialized in teaching or researching constitutional history, and news about Congresses or scientific meetings.

"Historia Constitucional" has a great diffusion. The journal is daily viewed by 1200 users, and it has been included in some of the most important citation indexes and data bases, as   FECYT-RECYT, Scopus, Latindex, RESH, DICE, DIALNET, INTUTE, DOAJ, Google Scholar, SOCOLAR, vLex, In-recs, e-revistas, Regesta-Imperii, EBSCO Publishing, Hein Online, Elsevier,Zeitschriftendatenbank, Ulrichs, Miar, Open Science Directory, Sudoc, Dulcinea, Emerging Source Citation Index and ÍnDICE CSIC.
Joaquín Varela Suanzes-Carpegna (1954-2018)
Founder of the Journal
