# Supplementary material for: Peer Review in Law Journals
Source: Front Res Metr Anal. 2021 Dec 8;6:787768. doi: 10.3389/frma.2021.787768 (PMC8692876; doi:10.3389/frma.2021.787768)
Supplement: Supplementary file 3 [file DataSheet2.ZIP › DOCUMENT - 1577-4430_1.RTF]

Normas de publicación
Instrucciones para los autores
Descargar normas de edición
Descargar hoja de evaluación
CIRIEC-España. Revista Jurídica de Economía Social y Cooperativa es una revista jurídica de periodicidad semestral, cuyo campo de estudio es la Economía Social y las empresas y entidades que la conforman, principalmente cooperativas, sociedades laborales, mutualidades, fundaciones y asociaciones.
El Consejo de Redacción de la revista CIRIEC-España. Revista Jurídica de Economía Social y Cooperativa examinará todos los artículos relacionados con el mencionado objeto de estudio que le sean remitidos.
Los trabajos deberán ser inéditos y no estar presentados para su publicación en ningún otro medio. Se supone que todos los autores han dado su aprobación para que el manuscrito se presente a la revista.
Los originales serán sometidos al criterio de evaluadores externos anónimos (doble referee). Serán criterios de selección el nivel científico y la contribución de los mismos al intercambio de información entre el ámbito investigador y el de los profesionales de las administraciones públicas y de las empresas de la economía social.
Los trabajos podrán ser aceptados, sujetos a revisiones menores o mayores, o rechazados. La decisión editorial será comunicada a los autores, indicando las razones para la aceptación, revisión o rechazo del manuscrito.
Los autores de los manuscritos aprobados para su publicación deberán ceder el copyright del artículo y autorizar a la Revista para publicar el artículo en su página web y a incluirlo en diversas bases de datos científicas, conforme a la legalidad vigente, conservando el derecho a autoarchivo.
Página web
http://ciriec-revistajuridica.es/
Dirección postal
CIRIEC-España, Revista Jurídica de Economía Social y Cooperativa
Campus Els Tarongers
Facultad de Economía, despacho 2P21
46022 Valencia
Envío de originales
Los autores deben registrarse y subir su trabajo al sistema de gestión electrónica de CIRIEC-España, revista jurídica de economía social y cooperativa. Esto debe hacerse a través del enlace:
https://ojs.uv.es/index.php/juridicaciriec/author/submit/1
Además deben de remitir una copia de su artículo a ammb@uv.es
Los autores deben, asimismo, remitir un escrito a la revista asegurando que el texto enviado es enteramente original y propiedad de los autores, y que no se encuentra en proceso de evaluación en otra revista.
Cómo citar artículos de esta revista
Ejemplo:
FAJARDO GARCÍA, I.G.: “La masa activa y pasiva en el concurso de cooperativas”, CIRIEC-España, Revista Jurídica de Economía Social y Cooperativa, nº 16, noviembre, 2005, págs. 9-11. DOI: xxx
FAJARDO GARCÍA, I.G. (2005): “La masa activa y pasiva en el concurso de cooperativas”, CIRIEC-España, Revista Jurídica de Economía Social y Cooperativa, nº 16, págs. 9-11. DOI: xxx
