# Supplementary material for: Peer Review in Law Journals
Source: Front Res Metr Anal. 2021 Dec 8;6:787768. doi: 10.3389/frma.2021.787768 (PMC8692876; doi:10.3389/frma.2021.787768)
Supplement: Supplementary file 3 [file DataSheet2.ZIP › DOCUMENT - 1578-3138_1.RTF]

Normas editoriales
Los autores deberán atenerse a las siguientes normas editoriales:
Sólo se publicarán trabajos originales e inéditos y en español. Como criterio general, la extensión de los trabajos no deberá exceder de 35 páginas (200 KB) tratándose de artículos; 15 páginas (85 KB) en comentarios jurisprudenciales y legislativos, y 10 páginas (50 KB) en el caso de reseñas bibliográficas.
Cada trabajo deberá ir acompañado con un resumen en español y en inglés de no más de 200 palabras cada uno y de 5 palabras clave en ambos idiomas, así como la traducción del título a ese idioma. Todo material será sometido a evaluación por pares ciegos para su publicación. El resultado del mismo se comunicará dentro de los 15 días siguientes a la fecha de reunión del Consejo Editorial.
Los trabajos deberán ser enviados a la siguiente dirección:
Profesor Dr. Ángel Espiniella Menéndez
Área de Derecho Internacional privado
Departamento de Derecho privado y empresa
Facultad de Derecho, Universidad de Oviedo
Edfcio. de Dptos. Jurídicos 
Avda. de El Cristo s/n 
33006. Oviedo. Asturias (España)
Teléfono: (34) 985 104 8 73
espiniell@uniovi.es; espiniell@gmail.com
Garantizar una evaluación por pares anónima
Para asegurar la integridad de la evaluación por pares anónima para el envío a la revista, se debe intentar que los autores/as y los revisores/as desconozcan sus identidades entre ellos. Esto implica que los autores/as, editores/as y revisores/as (los cuales suben documentos como parte de su revisión) comprueben si los siguientes pasos se han seguido cuidadosamente en cuanto al texto y las propiedades del archivo:
`.	Los autores/as del documento han eliminado sus nombres del texto, con “Autor/a” y el año que se usa en las referencias y en las notas a pie de página, en vez de el nombre de los autores/as, el título del artículo, etc.
`.	En los documentos de Microsoft Office, las identificación del autor/a también debe eliminarse de las propiedades del archivo.
Para Microsoft 2003 y versiones previas, y versiones de Word de Macintosh:
()	Seleccione en el menú Archivo: Guardar como > Herramientas (o Opciones en Mac) > Seguridad > Eliminar información personal en las propiedades del archivo al guardar > Guardar.
Para MacIntosh Word 2008 (y futuras versiones)
()	En el menú Archivo seleccione “Propiedades”.
()	En la pestaña Resumen elimine la información identificativa de todos los campos.
()	Guarde el Archivo.
Para Microsoft 2007 (Windows):
()	Haga clic en el botón de Office en la esquina superior izquierda de la aplicación Office
()	Seleccione “Preparar” en el menú Opciones.
()	Seleccione “Propiedades” para el menú Opciones de “Preparar”.
()	Elimine toda la información de los campos de propiedades del documento que aparecen debajo de menú principal de opciones.
()	Guarde el documento y cierre la sección de campos de propiedades del documento.
Para Microsoft 2010 (Windows):
()	En el menú Archivo seleccione “Preparar para compartir”.
()	Haga clic en el icono “Comprobación de problemas”.
()	Haga clic en el icono “Inspeccionar documento”.
()	Desmarque todas las casillas excepto “Propiedades del documento e información personal”.
()	Ejecute el inspector de documento, el cual realizará una búsqueda en las propiedades del documento e indicará si algún campo de propiedades del documento contiene alguna información.
()	Si el inspector de documento encuentra información se lo notificará y le dará la opción de “Eliminar todo”, en la cual tendrá que hacer clic para eliminar todas las propiedades del documento y la información personal.
`.	Para archivos PDF:
()	En los PDFs, los nombres de los autores/as también deben ser eliminados de las propiedades del documento que se encuentran debajo de Archivo en el menú principal de Adobe Acrobat.
NORMAS DE ESTILO PARA EL ANUARIO
   El modelo de citas del Anuario Español de Derecho Internacional Privado no es caprichos o. Es el que se utiliza en Naciones Unidas para los documentos oficiales en lengua francesa o española y es el mismo que se sigue en el  para los estudios en lengua francesa del Recueil des Cours de l’Académie de Droit International de La Haye.
    No sigue el modelo americano, ni el alemán, ni el italiano.
    Se ruega a los autores que consulten algún volumen anterior, o algún estudio contenido en la presente página web, para familiarizarse con el modelo que se sigue.
     Algunas indicaciones pueden ser de utilidad
INDICACIONES GENERALES
1º) No se utilizan en ningún caso textos en MAYÚSCULAS, VERSALES o NEGRITAS (salvo para los títulos de los estudios).
2º) En el texto las cursivas deben reservarse exclusivamente para idiomas extranjeros y clásicos.
3º) Si se quiere enfatizar algo introducir comillas.
ESQUEMA Y SUMARIO
 El índice de los artículos deberá respetar el siguiente esquema
I. Las acciones reivindicatorias contra la mujer casada 
1. Acciones dirigidas las patrimonio
`.	A) Eficacia punitiva
De manera el sumario sea como, por ejemplo, el siguiente
     SUMARIO: I. Planteamiento. II. El Derecho procesal europeo ante las situaciones vinculadas a terceros Estados: 1. Crítica a la “asimetría” de las normas europeas: A) Admisibilidad de foros estatales no adecuados. B) Alteración de las normas europeas. 2. ¿Revisión de la “asimetría” a la luz del reconocimiento mutuo?: A) Correlación entre la competencia y el reconocimiento. B) Reflejo de las normas “regionales” en ordenamientos de terceros Estados. III. Los acuerdos con terceros Estados en materia procesal: 1. Crítica a la disparidad de competencias para participar en acuerdos: A) La competencia exclusiva de la Unión y la competencia “independiente” de los Estados. B) La autorización de la Unión a los Estados. 2. ¿Revisión de la disparidad a la luz del reconocimiento mutuo?: A) La competencia conjunta. B) Una competencia acorde con el carácter transversal del reconocimiento mutuo. IV. Conclusiones.
 AUTORES
Los autores se citan con la inicial del nombre y después el apellido y en ningún caso llevan versalitas o mayúsculas
 B. Ubertazzi  no   UBERTAZZI, B. o   Ubertazzi, B.
CITAS
0.	 A) Citas de remisión
Para las citas de remisión indicar vid.
Para los textos que se citan de manera literal indicar: cf.
Las páginas deberán citarse de la primera a la última. el empleo de “y siguientes” hace sospechar que la cita no está confrontada pero si se acude a este recurso y indicar únicamente “ss” y no “y siguientes” o términos similares
Si un artículo de revista o un capítulo de un libro ya está citado en su integridad  indicar: loc. cit.
Si el autor está varias veces citado indicar
B. Ubertazzi, “Las acciones contra la mujer casada…”, loc. cit.,
Si un libro se repite indicar op. cit.
Si el autor está varias veces citado indicar
B. Ubertazzi, El arresto del quebrado…, op. cit.,
0.	 B) Citas de libros
El orden es autor, libro, lugar de publicación, editorial, fecha y páginas. Se separan con comas y no se introducen puntos o dos puntos
B. Ubertazzi, El arresto del quebrado en el Derecho rumano, Madrid, Tecnos, 2011, pp. 234-267.
0.	C) Citas de estudios en revistas o en obras colectivas
 Los artículos de revistas no llevan cursiva, sino que se insertar entre comillas “Las acciones contra la mujer casada en el Derecho internacional privado rumano”
A continuación va el texto de la revista  o de la obra colectiva en cursiva, el volumen, el año (y no al revés) y las páginas
Las Revistas se citan el cursiva. Por favor comprobar el texto del Anuario para uniformizar las citas: las más frecuentes son
