# Supplementary material for: Peer Review in Law Journals
Source: Front Res Metr Anal. 2021 Dec 8;6:787768. doi: 10.3389/frma.2021.787768 (PMC8692876; doi:10.3389/frma.2021.787768)
Supplement: Supplementary file 3 [file DataSheet2.ZIP › DOCUMENT - 1578-3138_2.RTF]

Guía de buenas prácticas
Guía de buenas prácticas para la publicación
La presente Guía de buenas prácticas pretende constituir un código de conducta dirigido a las partes implicadas en la gestión y publicación de los resultados científicos en el Anuario Español de Derecho Internacional privado: equipo editorial, autores y revisores de los trabajos.
1. Equipo editorial del Anuario Español de Derecho internacional privado
El Consejo de Redacción del Anuario, junto con la Dirección y la Secretaría, son los responsables de los contenidos publicados, por lo que deben asegurar su calidad científica, evitar las malas prácticas en la publicación de los resultados de las investigaciones y gestionar la edición de los trabajos recibidos en un tiempo razonable. Dicha responsabilidad implica observar los siguientes principios:
1.1 Imparcialidad
El Consejo de Redacción debe ser imparcial al gestionar los trabajos propuestos para su publicación y ha de respetar la independencia intelectual de las autoras y autores, a quienes se debe reconocer el derecho a réplica en caso de haber sido evaluados negativamente. No se deben excluir los trabajos que presentan resultados negativos de una investigación.
1.2 Confidencialidad
Las personas que componen el Consejo de Redacción tienen la obligación de guardar confidencialidad sobre los textos recibidos y su contenido hasta que hayan sido aceptados para su publicación. Solo entonces se puede difundir su título y autoría. Asimismo, ningún miembro del Consejo de Redacción puede usar para sus propias investigaciones datos, argumentos o interpretaciones contenidos en trabajos inéditos, salvo consentimiento expreso por escrito de quienes lo hayan realizado.
1.3 Revisión de los trabajos
El Consejo de Redacción ha de asegurar que los trabajos de investigación publicados han sido evaluados por, al menos, dos especialistas en la materia, y que dicho proceso de revisión ha sido justo e imparcial. El método empleado en la revisión por pares que mejor se adapta a la comunidad científica a la que se dirige la revista es el procedimiento de doble ciego (anonimato de quienes han realizado el trabajo y la evaluación). Cuando una de las dos evaluaciones sea negativa, se solicitará un tercer informe. El Consejo de Redacción debe sopesar el rechazo de un autor o autora a ser evaluado por determinados especialistas (en caso de que la evaluación no sea anónima), si las razones presentadas se consideran razonables. El Consejo de Redacción no tiene la obligación de prescindir de tales especialistas si considera que sus opiniones son fundamentales para la correcta evaluación del trabajo. Las personas que sometan un trabajo a evaluación podrán proponer los nombres de hasta tres especialistas para la evaluación de su trabajo. El Consejo de Redacción se reserva la decisión de aceptar o no esta propuesta, no estando obligado a comunicar dicha decisión. El Consejo de Redacción debe hacer hincapié en que en el proceso de evaluación se vigilen la originalidad de los trabajos y detecten el plagio y las publicaciones redundantes, así como los datos falsificados o manipulados. Además, se han de indicar claramente las secciones de la revista cuyos contenidos están sujetos a revisión por pares. El Consejo de Redacción debe valorar y agradecer la contribución de quienes hayan colaborado en las evaluaciones de los trabajos remitidos a la revista. Asimismo, ha de promover que las autoridades académicas reconozcan las actividades de revisión por pares como parte del proceso científico y debe prescindir de quienes realizan evaluaciones de baja calidad, incorrectas, irrespetuosas o entregadas fuera de los plazos establecidos.
1.4 Aceptación o rechazo de manuscritos
La responsabilidad de aceptar o rechazar un trabajo para su publicación recae en el Consejo de Redacción, que se deberá basar para ello en los informes recibidos sobre el mismo. Dichos informes deberán basar su dictamen sobre la calidad de los trabajos en su relevancia, originalidad y claridad de exposición. El Consejo de Redacción puede rechazar directamente los trabajos recibidos, sin recurrir a un proceso de consulta externa , si los consideran inapropiados para la revista por carecer del exigible nivel de calidad, por falta de adecuación a los objetivos científicos de la revista, o por presentar evidencias de fraude científico.
1.5 Desautorización y noticia de irregularidad
El Consejo de Redacción se reserva el derecho de desautorizar aquellos trabajos ya publicados de los que posteriormente se determine su falta de fiabilidad como resultado tanto de errores involuntarios como de fraudes o malas prácticas científicas: fabricación, manipulación o copia de datos, plagio de textos y publicación redundante o duplicada, omisión de referencias a las fuentes consultadas, utilización de contenidos sin permiso o sin justificación, etc. El objetivo que guía la desautorización es corregir la producción científica ya publicada, asegurando su integridad. El conflicto de duplicidad, causado por la publicación simultánea de un artículo en dos revistas, ha de resolverse determinando la fecha de recepción del trabajo en cada una de ellas. Si solo una parte del artículo contiene algún error, este se puede rectificar posteriormente por medio de una nota editorial o una fe de erratas. En caso de conflicto, la revista solicitará al autor o autores las explicaciones y pruebas pertinentes para aclararlo, y tomará una decisión final basada en éstas. La revista publicará obligatoriamente, en sus versiones impresa y electrónica, la noticia sobre la desautorización de un determinado texto y en ella se deben mencionar las razones para tal medida, a fin de distinguir la mala práctica del error involuntario. La revista notificará asimismo la desautorización a los responsables de la institución del autor o autores del artículo. La decisión de desautorizar un texto debe adoptarse lo antes posible, con el objeto de que dicho trabajo erróneo no sea citado en su campo de investigación. Los artículos desautorizados se conservarán en la edición electrónica de las revistas, advirtiendo de forma clara e inequívoca de que se trata de un artículo desautorizado, para distinguirlo de otras correcciones o comentarios. En la edición impresa se hará constar la desautorización con la mayor brevedad por medio de una editorial o comunicación, en los mismos términos en que se haya hecho en la versión electrónica. Como paso previo a la desautorización definitiva, la revista podrá emitir una noticia de irregularidad, aportando la información necesaria en los mismos términos que en el caso de una desautorización. La noticia de irregularidad se mantendrá el tiempo mínimo necesario, y concluirá con su retirada o con la desautorización formal del artículo.
1.6 Aplicación de las normas del Consejo de Redacción
La persona que desempeña la Dirección de la revista es responsable de que se apliquen correctamente las normas que regulan el funcionamiento del Consejo de Redacción y ha de garantizar que sus miembros las conocen. Estas son: promocionar y representar a la revista en los distintos foros; sugerir y apoyar posibles mejoras; recabar las colaboraciones de especialistas de referencia en la materia; revisar, en una primera evaluación, los trabajos que se reciben; escribir para la revista editoriales, revisiones, comentarios, noticias, recensiones, etc.; asistir a las reuniones del Consejo de Redacción.
1.7 Normas de Autoría
Las normas de presentación de originales de cada revista (referentes a la extensión del resumen y del artículo, la preparación de las imágenes, el sistema para las referencias bibliográficas, etc.) deberán ser públicas.
1.8 Conflicto de intereses
El conflicto de intereses surge cuando un trabajo recibido en la revista está firmado por una persona que forma parte del Consejo de Redacción, por quien tiene relación personal o profesional directa, o está estrechamente relacionado con la investigación pasada o presente de quien lo integre. Quien está afectado por cualquiera de estos casos debe abstenerse de intervenir en el proceso de evaluación del artículo propuesto.
2. Sobre la autoría de los artículos en el Anuario
2.1 Normas de publicación
Los textos presentados para su publicación han de ser el fruto de una investigación original e inédita. Han de incluir los datos obtenidos y utilizados, así como una discusión objetiva de sus resultados. Se ha de aportar la información suficiente para que cualquier especialista pueda repetir las investigaciones realizadas y confirmar o refutar las interpretaciones defendidas en el trabajo. Las autoras y autores deberán mencionar adecuadamente la procedencia de las ideas o frases literales tomadas de otros trabajos ya publicados de la forma que se indique en la normativa de la revista. Cuando se incluyan imágenes como parte de la investigación, se deberá explicar adecuadamente cómo se crearon u obtuvieron, siempre y cuando resulte necesario para su comprensión. En caso de emplear material gráfico (figuras, fotos, mapas, etc.) reproducido parcialmente en otras publicaciones, los autores deberán citar su procedencia, aportando los permisos de reproducción pertinentes si fuera necesario. Se debe evitar la fragmentación innecesaria de los artículos. Si se trata de un trabajo muy extenso, se puede publicar en varias partes, de manera que cada una desarrolle un aspecto determinado del estudio general. Se deben publicar los diferentes trabajos relacionados en la misma revista para facilitar su interpretación por parte de los lectores.
2.2 Originalidad y plagio
Los autores deben asegurar que los datos y resultados expuestos en el trabajo son originales y no han sido copiados, inventados, distorsionados o manipulados. El plagio en todas sus formas, la publicación múltiple o redundante, así como la invención o manipulación de datos constituyen faltas graves de ética y se consideran fraudes científicos. Los autores no enviarán al Anuario originales que previamente estén sometidos a consideración en otra revista, ni enviarán ese original a otra revista en tanto no reciba notificación de su rechazo o lo retire voluntariamente. Sin embargo, es admisible publicar un trabajo que amplíe otro ya aparecido como nota breve, comunicación o resumen en las actas de un congreso, siempre que se cite adecuadamente el texto sobre el que se basa y que las modificaciones supongan una modificación sustancial de lo ya publicado. También son aceptables las publicaciones secundarias si se dirigen a lectores totalmente diferentes; por ejemplo, si el artículo se publica en diferentes idiomas o si hay una versión para especialistas frente a otra dirigida al público en general. Se deberán especificar estas circunstancias y se citará apropiadamente la publicación original.
2.3 Autoría del trabajo
Quien figure como responsable del artículo ante la revista, en el caso de autoría múltiple, debe garantizar el reconocimiento de quienes hayan contribuido significativamente en la concepción, planificación, diseño, ejecución, obtención de datos, interpretación y discusión de los resultados del trabajo; en todo caso todas las personas que lo firman comparten la responsabilidad del trabajo presentado. Asimismo, quien actúa como persona de contacto debe asegurar que quienes firman el trabajo han revisado y aprobado la versión final del trabajo y dan su visto bueno para su posible publicación. La autoría de contacto debe asegurar que no se ha omitido ninguna de las firmas responsables del trabajo y que satisface así los mencionados criterios de coautoría, con lo que se evita la autoría ficticia o regalada, que constituye una mala práctica científica. Asimismo, deben reconocerse en una nota del artículo, a modo de agradecimiento, la contribución de otras colaboraciones que no figuren como firmantes ni sean responsables de la versión final del trabajo. Si la revista o los firmantes del artículo lo solicitan, en la versión publicada se describirá de forma escueta la aportación individual de cada integrante del grupo firmante al trabajo colectivo.
2.4 Fuentes de información
En el texto del trabajo se deberán reconocer las publicaciones que hayan influido en la investigación, por lo que se debe identificar y citar en la bibliografía las fuentes originales en las que se basa la información contenida en su trabajo. No ha de incluir, no obstante, citas irrelevantes para su trabajo o referidas a ejemplos parecidos, y no ha de abusar de las menciones a investigaciones ya asentadas en el corpus del conocimiento científico. El autor o la autora no debe utilizar la información obtenida privadamente a través de conversaciones, correspondencia o a partir de algún debate con colegas en la materia, a no ser que cuente con permiso explícito, por escrito, de su fuente de información y dicha información se haya recibido en un contexto de asesoramiento científico.
2.5 Errores significativos en trabajos publicados
Cuando un autor o autora descubre un error grave en su trabajo tiene la obligación de comunicarlo a la revista lo antes posible, para modificar su artículo, retirarlo, retractarse o publicar una corrección o fe de erratas. Si el posible error es detectado por cualquiera de los miembros del Comité de Redacción, la autora o autor está obligado a demostrar que su trabajo es correcto. El proceso de resolución de estos conflictos es el descrito en el apartado 1.5.
2.6 Conflicto de intereses
Al texto del artículo se deberá acompañar una declaración, en la que conste la existencia de cualquier vínculo comercial, financiero o personal que pueda afectar a los resultados y las conclusiones de su trabajo. Asimismo, se deben indicar obligatoriamente todas las fuentes de financiación concedidas para el estudio. Esta información figurará en la versión publicada del artículo.
3. La evaluación de trabajos en el Anuario
Las personas que participan en la evaluación desempeñan un papel esencial en el proceso que garantiza la calidad de la publicación. Asisten a los órganos de la revista en la toma de las decisiones editoriales y ayudan a la mejora de los artículos.
3.1 Confidencialidad
Quien realice una evaluación debe considerar el trabajo que ha de revisar como un documento confidencial hasta su publicación, tanto en el transcurso del proceso de revisión como después de este. En ningún caso debe difundir ni usar la información, detalles, argumentos o interpretaciones contenidos en el texto objeto de revisión para su propio beneficio o el de otras personas, ni para perjudicar a terceras personas. Únicamente en casos especiales puede recabar el asesoramiento de otros especialistas en la materia, circunstancia de la que debe informar a la Dirección de la revista.
3.2 Objetividad
Quien realice una evaluación debe juzgar objetivamente la calidad del trabajo completo, es decir, incluyendo la información sobre la que se fundamenta la hipótesis de trabajo, los datos teóricos y experimentales y su interpretación, sin descuidar la presentación y redacción del texto. Debe concretar sus críticas, y ser objetivo y constructivo en sus comentarios. Ha de argumentar adecuadamente sus juicios, sin adoptar posturas hostiles y respetando la independencia intelectual de quien haya elaborado el trabajo. Quien realice una evaluación debe advertir a la Dirección de cualquier similitud sustancial entre el trabajo sometido a evaluación y otro artículo ya publicado o en proceso de evaluación en otra revista (publicación redundante o duplicada). Igualmente, ha de llamar la atención sobre textos o datos plagiados, falsificados, inventados o manipulados.
3.3 Prontitud de respuesta
Quien realice una evaluación debe actuar con celeridad y ha de entregar su informe en el tiempo acordado, por lo que notificará a la Dirección los posibles retrasos. Asimismo, deberá comunicar a la Dirección lo antes posible si no se considera capaz de juzgar el trabajo encargado o en caso de que no pueda cumplir su tarea en el plazo acordado.
3.4 Reconocimiento de las fuentes de información
Quien realice una evaluación debe comprobar que son citados los trabajos relevantes ya publicados sobre el tema. Con ese objetivo revisará la bibliografía recogida en el texto, sugiriendo la eliminación de referencias superfluas o redundantes, o la incorporación de otras no citadas.
3.5 Conflicto de intereses
Quien realice una evaluación debe rechazar la revisión de un trabajo cuando mantenga una relación profesional o personal con cualquiera de las personas que hayan intervenido en su autoría que pueda afectar a su juicio sobre dicho trabajo. Pueden surgir igualmente conflictos de intereses cuando el trabajo por evaluar está estrechamente relacionado con el que está desarrollando en ese momento o con el que ya ha publicado. En estos casos, ante la duda, debe renunciar a la tarea encomendada y devolver el trabajo a la Revista, señalando los motivos para tal decisión.
4. Repositorios institucionales
Se autoriza a los autores la publicación del pre-print de su contribución al Anuario en repositorios institucionales o web personal del autor, en versión Word o pdf propio, una vez hayan sido aceptados los originales por el Consejo de redacción del Anuario tras el proceso de evaluación. El autor deberá incluir una nota al inicio de su trabajo conforme a la cual su contribución ha sido aceptada por el Anuario, en el número correspondiente, en prensa. El autor podrá auto-archivar en Repositorios de carácter académico o en su web personal la versión ya publicada por el Anuario transcurridos 12 meses desde su publicación.
