# Supplementary material for: Peer Review in Law Journals
Source: Front Res Metr Anal. 2021 Dec 8;6:787768. doi: 10.3389/frma.2021.787768 (PMC8692876; doi:10.3389/frma.2021.787768)
Supplement: Supplementary file 3 [file DataSheet2.ZIP › DOCUMENT - 1590-749X.RTF]

REGOLAMENTO DI AUTODISCIPLINA DI RASSEGNA TRIBUTARIA
La pubblicazione dei contributi della sezione delle sezioni “Dottrina”, “Profili istituzionali” e “Giurisprudenza” è subordinata a due livelli di valutazione da parte della Direzione della Rassegna e del Comitato per la Valutazione.
Il Comitato per la Valutazione è formato da almeno 12 membri, individuati dalla Direzione fra professori ordinari di ruolo o fuori ruolo, indicati in un elenco periodicamente aggiornato. Il numero dei revisori può̀ essere aumentato fino a 36.
I contributi devono essere previamente inviati alla Redazione di Rassegna che provvede a trasmetterli ai Direttori anche in forma digitale.
Il contributo approvato collegialmente dalla Direzione è sottoposto, in forma anonima, al giudizio di un membro del Comitato per la Valutazione designato a rotazione sulla base delle specifiche competenze in relazione all’argomento del contributo. Il giudizio è comunicato entro 15 giorni. La valutazione può̀ essere positiva, negativa o subordinata a ulteriori interventi da parte degli autori. In quest’ultimo caso, l’autore è tempestivamente informato delle indicazioni formulate e degli interventi suggeriti e provvede all’ulteriore elaborazione; la Direzione, a sua volta, valutata la significatività̀ dell’ulteriore elaborazione, decide in ordine alla pubblicazione.
Nel Colophon di ciascun numero di Rassegna sono indicati i membri del Comitato per la Valutazione. Nell’indice di ogni numero di Rassegna, i contributi pubblicati a seguito di una valutazione positiva sono contrassegnati da un asterisco.
Ferma restando comunque l’approvazione collegiale della Direzione, possono essere pubblicati contributi che non siano stati sottoposti al giudizio del Comitato per la Valutazione entro il limite del 40%.
