# Supplementary material for: Peer Review in Law Journals
Source: Front Res Metr Anal. 2021 Dec 8;6:787768. doi: 10.3389/frma.2021.787768 (PMC8692876; doi:10.3389/frma.2021.787768)
Supplement: Supplementary file 3 [file DataSheet2.ZIP › DOCUMENT - 1696-9634_1.RTF]

1
INSTRUCCIONES A LOS AUTORES. REVISTAS GENERALES


1. EVALUACIÓN DE LOS ORIGINALES

Todos los estudios recibidos por la Revista General de Derecho Europeo, con independencia de que obedezcan a una petición de la propia revista o se hayan remitido espontáneamente por el autor/a, serán objeto de evaluación externa anónima por dos revisores que tengan la condición de pares, recurriéndose a una tercera opinión en caso de clara discrepancia.

Los autores serán en todo caso informados de forma motivada del resultado final de la referida evaluación con indicación expresa de los argumentos a favor o en contra del trabajo evaluado. En caso de que la evaluación resultara positiva con reparos, será exigible al autor del manuscrito la introducción de las mejoras correspondientes en el plazo que en cada caso se establezca por la dirección de la revista.

Para una mayor transparencia del proceso de selección de originales la revista publicará al final de cada año una relación de las personas que han ejercido labores de evaluación. Igualmente se hará pública la amplia lista de evaluadores que con carácter ordinario emplea la revista con independencia de que, dependiendo de la concreta materia de cada artículo o nota, pueda solicitarse también la emisión de un informe de evaluación por expertos ajenos a la citada lista de manera que pueda quedar preservada en todos los casos la exigencia de que la evaluación sea realizada por personas de la cualificación requerida.
Con todo, se garantizará plenamente el anonimato de los informes emitidos en cada caso particular y no se permitirá en ningún caso que los autores sugieran nombres de posibles revisores.

2. CUESTIONES RELATIVAS AL ENVÍO DE ORIGINALES

Los trabajos deben presentarse en Microsoft Word.

Las contribuciones podrán enviarse por correo electrónico a la siguiente dirección secretario_RGDE@iustel.com, del Secretario de la Revista General de Derecho Europeo D. Asier Garrido Muñoz.

Adicionalmente se mantiene abierta la siguiente dirección de http://www.iustel.com: Revistas@iustel.com.


Princesa, 29, 2. º 28008 Madrid > T 915 488 281 > F 915 478 645 > iustel@iustel.com > www.iustel.com

2


Los archivos deben nombrarse con los apellidos del autor del trabajo, seguido de un punto y de la abreviatura de la sección correspondiente de la Revista. Las abreviaturas de cada sección son las siguientes:

a)	ed. :	Editorial	
b)	est.:	Estudios	
c)	jur.:	Comentarios de jurisprudencia	
d)	leg.:	Comentarios de legislación	
e)	obs.:	Observatorio de actualidad europea	
f)	bib.:	Bibliografía	

3. CUESTIONES RELATIVAS A LA EDICIÓN DE LOS TRABAJOS

Podrán remitirse a cada una de las Revistas Generales, para su publicación, todos aquellos artículos relacionados con la materia propia de cada una ellas.

En el caso de la Revista General de Derecho Europeo, los trabajos podrán estar escritos en castellano o en inglés (excepcionalmente, podrían tomarse en consideración trabajos redactados en francés, italiano, alemán o portugués).

En todos los trabajos, sea cual sea la lengua en la que se escriban, se habrá de indicar en castellano y en inglés tanto el título del trabajo como el sumario y el resumen o abstract que lo acompañe.

El tipo de letra será Arial 10 con interlineado sencillo para el texto y Arial 9 para las notas a pie de página. El formato de página ha de ser estándar, con los márgenes superior e inferior de 2'5 cm. y los márgenes izquierdo y derecho de 3 cm. El texto estará justificado y puede contener hipervínculos a páginas web.
Los documentos deberán encabezarse con el título del trabajo (que, en la medida de lo posible, habrá de ser breve, preciso y reflejar de manera adecuada el contenido del análisis doctrinal del trabajo), el nombre del autor/a y su cargo académico o actividad que desempeña, así como la referencia a la Universidad o Institución a la que se encuentra adscrito/a. Asimismo debe aportarse su e-mail de contacto. A falta de esta dirección de correo electrónico PORTALDERECHO S.A. facilitará la siguiente: revistas@iustel.com.

El título deberá ir centrado, en letra mayúscula y en negrita.

El nombre y apellidos del autor/a irán en letra mayúscula y su cargo en letra minúscula. Ambos en letra redonda, sin negrita y centrado.

Los párrafos irán sin sangría en la primera línea ni espaciado entre ellos. Los primeros epígrafes se presentarán en letra mayúscula, en numeración romana y en negrita; los primeros subepígrafes se presentarán en letra minúscula, en numeración arábiga y en negrita; a partir de aquí, los posibles siguientes subepígrafes irán en letra minúscula, en numeración arábiga y sin negrita en la secuencia: 1.1, 1.2, 1.3: 1.3.1, etc.


Princesa, 29, 2. º 28008 Madrid > T 915 488 281 > F 915 478 645 > iustel@iustel.com > www.iustel.com

3


La extensión de los trabajos será (de forma aproximada) la siguiente:

-	Estudios: un máximo de 30 páginas

-	Comentarios de jurisprudencia y legislación: entre 10 y 20 páginas

-	Recenciones: 2-3 páginas.


Tanto los estudios como los comentarios de jurisprudencia y de legislación deberán inlcuir al inicio un breve resumen (Abstract) de 6 a 10 líneas redactado en el idioma del artículo, en castellano y en inglés, acompañado de una serie de palabras claves o Key Words (no más de 5) en castellano y en inglés.
A falta del cumplimiento de cualquiera de estos criterios, se autoriza a PORTALDERECHO S.A. a realizar las adaptaciones editoriales necesarias.

Método de cita: Las citas de los trabajos deberán ir en notas a pie de página y no en notas al final. Tampoco se incluirá al final del texto una lista de bibliografía citada.

Las referencias legislativas o jurisprudenciales contendrán todos los datos necesarios para su adecuada localización, y se ajustarán a los estándares de citación en publicaciones jurídicas españolas o, en su caso, de los países a que correspondan las normas o sentencias citadas. En relación con estas últimas, se recomienda con carácter general la utilización del sistema ECLI (Identificador europeo de jurisprudencia), en particular para la cita de jurisprudencia del TJUE (tal y como la propia Institución establece: https://curia.europa.eu/jcms/jcms/P_126035/es/). Cuando se haga referencia a sitios de Internet, habrá que indicar expresamente, entre paréntesis, la fecha última en que fueron visitados o bien incluir una referencia general a este respecto en la primera nota a pie de página del trabajo.

Para las referencias bibliográficas se seguirá el siguiente método de cita:

-	artículos: autor/a en mayúsculas comenzando por los apellidos, título entre comillas y denominación de la revista en cursiva (ej. GARCÍA GARCÍA, A., "La cuestión prejudicial en el marco del sistema jurisdiccional de la UE", Revista General de Derecho Europeo, nº 1, 2003, pp. 1-20).

-	monografías: autor/a en mayúsculas comenzando por los apellidos y título en cursiva (ej. GARCÍA GARCÍA, A., Introducción al Derecho de la Unión Europea, Iustel, Madrid, 2012).

-	contribuciones en obras colectivas: autor/a en mayúsculas comenzando por los apellidos, título de la contribución entre comillas y título de la obra en cursiva (ej. GARCÍA GARCÍA, A., "Los Abogados Generales en el Tribunal de Justicia de la Unión


Princesa, 29, 2. º 28008 Madrid > T 915 488 281 > F 915 478 645 > iustel@iustel.com > www.iustel.com

4


Europea", en HERNÁNDEZ HERNÁNDEZ, B., El sistema jurisdiccional de la Unión Europea, Iustel, Madrid, 2012, pp. 120-150).

Las referencias bibliográficas dentro de una misma nota se ordenarán alfabéticamente por el apellido del autor. Se autoriza a PORTALDERECHO S.A. a ajustar las referencias bibliográficas aportadas por los autores a las propias de la Editorial.

Por lo que se refiere a las abreviaturas más habituales, se utilizarán las siguientes: "art."; "párr." y "párrs."; "p." y "pp."; "núm." o "Núm."; "apdo."; "véase". Se emplearán siempre las comillas españolas " " y dentro de los textos no se utilizará negrita ni subrayado. Para resaltar alguna palabra o pasaje del texto se utilizará únicamente la cursiva.

4. CUESTIONES RELATIVAS A LA PUBLICACIÓN DE LOS TRABAJOS

En relación con los derechos de autor, los autores pueden utilizar sus derechos para publicar sus trabajos en cualquier otra publicación, siempre en soporte papel (y no en soporte electrónico), con el único requisito de comunicarlo con antelación a la Dirección de la Revista y de reconocer la previa aparición del trabajo en esta última, incluyendo el nombre y el dominio en la red de la RGDE (http://www.iustel.com).

Los Consejos rectores de cada una de las Revistas Generales exigirán que todos los trabajos publicados sean originales y, por tanto, los autores se abstendrán de enviar a la RGDE textos que ya hubieran sido publicados bajo cualquier formato. No obstante y con carácter muy excepcional, los órganos rectores de la Revista podrían considerar de interés publicar un trabajo que ya hubiera sido difundido por otra publicación periódica de papel atendiendo a la especial relevancia o actualidad del tema.

Se autoriza a PORTALDERECHO S.A. a la publicación en formato papel de los trabajos remitidos por los autores.


Princesa, 29, 2. º 28008 Madrid > T 915 488 281 > F 915 478 645 > iustel@iustel.com > www.iustel.com
