# Supplementary material for: Peer Review in Law Journals
Source: Front Res Metr Anal. 2021 Dec 8;6:787768. doi: 10.3389/frma.2021.787768 (PMC8692876; doi:10.3389/frma.2021.787768)
Supplement: Supplementary file 3 [file DataSheet2.ZIP › DOCUMENT - 1696-9642_1.RTF]

INSTRUCCIONES A LOS AUTORES. REVISTAS GENERALES


1. CUESTIONES RELATIVAS AL ENVÍO DE ORIGINALES

Los trabajos deben presentarse en Microsoft Word.

Las contribuciones podrán enviarse por correo electrónico a siguiente dirección: revistas@iustel.com, con copia a soledad.ruiz@ua.es.

Los archivos deben nombrarse con el nombre y apellidos del autor o la autora del trabajo, o con palabras que aludan al título o contenido del archivo cuando no se trate de un trabajo doctrinal, seguido de un punto y de la abreviatura de la sección correspondiente de la Revista. Las abreviaturas de cada sección son las siguientes:
a)	est. :	Estudios	
b)	obs.:	Observatorio permanente de justicia civil y penal	
c)	leg.:	Comentarios y notas de legislación procesal española	
d)	cjur.:	Comentarios de jurisprudencia española seleccionada	
e)	jur.tc.:	Jurisprudencia española seleccionada. Tribunal Constitucional	
f)	jur.ts.:	Jurisprudencia española seleccionada. Tribunal Supremo	
g)	jur.ap.:	Jurisprudencia española seleccionada. Audiencia Provincial	
h)	rev.:	Revista de revistas	
i)	lib.:	Libros	
j)	alem.:	Derecho alemán	
k)	ita.:	Derecho italiano	
l)	anglo.:	Derecho angloamericano	
m)	latinm:	Derecho Latinoamericano	


Ejemplos:	Gimeno Sendra.est.doc


173-2005.jur.tc.doc

De cara a los números que se editen a partir de septiembre de 2021, la RGDPR aceptará trabajos para su evaluación y publicación, en su caso, en atención a las siguientes circunstancias:

`)	La publicación de Estudios, Comentarios y Recensiones, se supeditará a las siguientes pautas temporales:

Para publicar en el Nº DE ENERO: la fecha tope de recepción de trabajos será la del 1 de octubre del año inmediatamente anterior.


Princesa, 29, 2. º 28008 Madrid > T 91548 82 81 > F 915 489 482 > iustel@iustel.com > www.iustel.com

2


Para publicar en el Nº DE MAYO: la fecha tope para la recepción de trabajos será la del 1 de enero del año en curso.

Para publicar en Nº DE SEPTIEMBRE: la fecha tope para la recepción de trabajos será la de 1 de mayo del año en curso.

Los trabajos recibidos más allá de estas fechas no se archivarán para los siguientes números. Las personas interesadas deberán enviarlos de nuevo –si persiste el ánimo de publicar en la RGDPR- dentro del plazo de aceptación de publicaciones para el siguiente número, con las indicaciones en el "asunto" del email señaladas más arriba.

Igualmente, si el número estuviera completo al tiempo de recibir la solicitud de publicación, no se archivará el trabajo, emplazando a la persona a enviarlo de nuevo de cara al siguiente número.

`)	En el email de envío de trabajos deberá hacerse constar en el asunto el mes de publicación para el que se envía (enero, mayo o septiembre), el año y la categoría del trabajo (Estudio, Comentario de Jurisprudencia, Recensión).


0.	CUESTIONES RELATIVAS A LA EDICIÓN DE LOS TRABAJOS

Podrán remitirse a cada una de las Revistas Generales, para su publicación, todos aquellos artículos relacionados con la materia propia de cada una ellas.

Los trabajos podrán estar escritos en castellano, inglés, francés, italiano, alemán o portugués.

En todos los trabajos doctrinales, sea cual sea la lengua en la que se escriban, se habrá de indicar en castellano y en inglés, el título del trabajo, el sumario, el resumen y las palabras clave.

El tipo de letra será Arial 10 con interlineado sencillo. El texto puede contener hipervínculos a páginas web y notas al pie.

Los documentos deberán encabezarse con el título del trabajo (que debe ser breve y reflejar el contenido del análisis doctrinal en su totalidad con el fin de que, con su inclusión en el sumario, éste quede claro, conciso y concreto), el autor o autora y su cargo académico, o actividad que desempeña, así como, en su caso, la Universidad a la que pertenece, o la Institución en la que desempeña su actividad. Así mismo, debe aportarse un e-mail de contacto. A falta de esta dirección de correo electrónico PORTALDERECHO S.A. facilitará la siguiente: revistas@iustel.com.
El título deberá ir centrado, en letra mayúscula y en negrita.

El nombre y apellidos del autor o autora del trabajo irán en letra mayúscula y su cargo en letra minúscula. Ambos en letra redonda, sin negrita y centrado.


Princesa, 29, 2. º 28008 Madrid > T 91548 82 81 > F 915 489 482 > iustel@iustel.com > www.iustel.com

3


Cada uno de los epígrafes en los que se divida el trabajo irán centrados. Los primeros epígrafes se presentarán en letra mayúscula, en numeración romana, centrados y en negrita; los primeros subepígrafes se presentarán en letra minúscula, en numeración arábiga, centrados y en negrita; a partir de aquí, los posibles siguientes subepígrafes irán en letra minúscula, en numeración arábiga, centrados y sin negrita en la secuencia: 1.1, 1.2, 1.3: 1.3.1, etc.

La extensión de los trabajos será (de forma aproximada) la siguiente:

-	Estudios: 30 páginas

-	Comentarios: 10-20 páginas

-	Notas: 2-5 páginas

-	Recensiones y comentarios de libros: 5-10 páginas

-	Notas de libros: 2 páginas


A falta del cumplimiento de cualquiera de estos criterios, se autoriza a PORTALDERECHO S.A. a realizar las actualizaciones editoriales necesarias.

Citas: Las citas de los trabajos deberán ir en notas a pie de página y no en notas al final. Las referencias bibliográficas, legislativas o jurisprudenciales contendrán todos los datos necesarios para su adecuada localización, y se ajustarán a los estándares de citación en publicaciones jurídicas españolas o, en su caso, de los países a que correspondan las normas o sentencias citadas. Cuando se haga referencia a sitios de Internet, habrá que indicar expresamente, entre paréntesis, la fecha última en que fueron visitados.

Se autoriza a PORTALDERECHO S.A. a ajustar las referencias bibliográficas aportadas por los autores o las autoras a las propias de la Editorial.

Desde la Revista General de Derecho Procesal se recomienda el uso de un lenguaje inclusivo en todo tipo de trabajo presentado.

3. CUESTIONES RELATIVAS A LA PUBLICACIÓN DE LOS TRABAJOS

En relación con los derechos de autoría, los trabajos se publicados se podrán volver a publicar en otra publicación, siempre que esta sea en soporte papel (y no en soporte electrónico), con el único requisito de reconocer la previa aparición en la Revista General correspondiente, incluyendo el nombre y el dominio en la red de la Revista (http://www.iustel.com).

Los Consejos rectores de cada una de las Revistas Generales exigirán que los trabajos sean originales, si bien siempre cabrán excepciones si por la importancia o actualidad del tema, los equipos consideran de interés publicar un trabajo en la Revista General una vez difundido por otra publicación periódica de papel.


Princesa, 29, 2. º 28008 Madrid > T 91548 82 81 > F 915 489 482 > iustel@iustel.com > www.iustel.com

4


Se autoriza a PORTALDERECHO S.A. a la publicación en formato papel de los trabajos remitidos por los autores y autoras.

EVALUACIÓN: Recibidos los originales, la Directora de Edición, la Responsable de Edición IUSTEL o la Secretaria de la Revista General realizará acuse de recibo al autor y enviará el original a dos expertos, para su evaluación, de forma anónima y siguiendo el sistema del doble ciego, con la finalidad de que emitan una valoración sobre el mismo de conformidad con el formulario establecido al efecto y que se les remite junto con el trabajo a evaluar.

La Directora de Edición notificará al autor o autora el resultado del proceso de evaluación externa que puede ser:

-	FAVORABLE.

-	FAVORABLE CONDICIONADO (con indicaciones de modificación).

-	DESFAVORABLE.


Si uno de los informes fuese favorable y otro desfavorable, se remitirá el trabajo a una tercera persona evaluadora. De ser positiva esta tercera evaluación, el trabajo será finalmente publicado en la revista.


Princesa, 29, 2. º 28008 Madrid > T 91548 82 81 > F 915 489 482 > iustel@iustel.com > www.iustel.com
