# Supplementary material for: Peer Review in Law Journals
Source: Front Res Metr Anal. 2021 Dec 8;6:787768. doi: 10.3389/frma.2021.787768 (PMC8692876; doi:10.3389/frma.2021.787768)
Supplement: Supplementary file 3 [file DataSheet2.ZIP › DOCUMENT - 1696-9650_1.RTF]

1
INSTRUCCIONES A LOS AUTORES. REVISTAS GENERALES


1. CUESTIONES RELATIVAS AL ENVÍO DE ORIGINALES

Los trabajos deben presentarse en Microsoft Word.

Las contribuciones podrán enviarse por correo electrónico a la siguiente dirección de http://www.iustel.com: revistas@iustel.com. La Editorial remitirá las mismas al Subdirector (D. Juan Francisco Mestre Delgado) y al Secretario (D. Tomás Cano Campos) de la Revista General de Derecho Administrativo.
Adicionalmente se mantiene abierta la siguiente dirección de http://www.iustel.com: cac@iustel.com.

Será asimismo opcional el envío de originales por medio de diskette a la dirección postal que indique el Subdirector / Secretario de la Revista General de Derecho Administrativo o a la siguiente dirección: C/ Princesa, n.º 29, 2.º dcha, 28008, Madrid.

Los archivos deben nombrarse con los apellidos del autor del trabajo, seguido de un punto y de la abreviatura de la sección correspondiente de la Revista. Las abreviaturas de cada sección son las siguientes:

a)	est. :	Estudios	
b)	cjur.:	Comentarios de jurisprudencia	
c)	crjur.:	Crónica de jurisprudencia	
d)	leg.:	Comentarios y Notas de legislación	
e)	ag.:	Resoluciones de las Agencias reguladoras	
f)	lib.:	Libros	
g)	rev.:	Revista de revistas	
h)	consul.:	Administración consultiva	
Ejemplos:	Muñoz Machado.est.doc	
		Mestre Delgado.cjur.doc	

2. CUESTIONES RELATIVAS A LA EDICIÓN DE LOS TRABAJOS

Podrán remitirse a cada una de las Revistas Generales, para su publicación, todos aquellos artículos relacionados con la materia propia de cada una ellas.

Los trabajos podrán estar escritos en castellano, inglés, francés, italiano, alemán o portugués.


Princesa, 29, 2. º 28008 Madrid > T 91548 82 81 > F 915 478 645 > iustel@iustel.com > www.iustel.com

2


En todos los trabajos, sea cual sea la lengua en la que se escriban, se habrá de indicar en castellano y en inglés, el título del trabajo, el sumario, el resumen y palabras clave.

El tipo de letra será Arial 10 con interlineado sencillo. El texto puede contener hipervínculos a páginas web y notas al pie.

Los documentos deberán encabezarse con el título del trabajo (que debe ser breve y reflejar el contenido del análisis doctrinal en su totalidad con el fin de que, con su inclusión en el sumario, éste quede claro, conciso y concreto), el autor y su cargo académico, o actividad que desempeña, así como, en su caso, la Universidad a la que pertenece, o la Institución en la que desempeña su actividad. Asimismo debe aportarse su e-mail de contacto. A falta de esta dirección de correo electrónico PORTALDERECHO S.A. facilitará la siguiente: revistas@iustel.com.

El título deberá ir centrado, en letra mayúscula y en negrita.

El nombre y apellidos del autor irán en letra mayúscula y su cargo en letra minúscula. Ambos en letra redonda, sin negrita y centrado.

Cada uno de los epígrafes en los que se divida el trabajo irán centrados. Los primeros epígrafes se presentarán en letra mayúscula, en numeración romana, centrados y en negrita; los primeros subepígrafes se presentarán en letra minúscula, en numeración arábiga, centrados y en negrita; a partir de aquí, los posibles siguientes subepígrafes irán en letra minúscula, en numeración arábiga, centrados y sin negrita en la secuencia: 1.1, 1.2, 1.3: 1.3.1, etc.

La extensión de los trabajos será (de forma aproximada) la siguiente:

-	Estudios: 30 páginas

-	Comentarios: 10-20 páginas

-	Notas: 2-5 páginas

-	Recensiones y comentarios de libros: 5-10 páginas

-	Notas de libros: 2 páginas

Los artículos de las secciones doctrinales y cuando la extensión de los mismos lo aconseje deberán contener un sumario y un breve resumen de 6 a 10 líneas redactado en el idioma del artículo, en castellano y en inglés.

Estos mismos artículos deberán acompañarse de las PALABRAS CLAVE (no más de 5), en el idioma original, en castellano y en inglés.

A falta del cumplimiento de cualquiera de estos criterios, se autoriza a PORTALDERECHO S.A. a realizar las actualizaciones editoriales necesarias.
Citas: Las citas de los trabajos deberán ir en notas a pie de página y no en notas al final. Las referencias bibliográficas, legislativas o jurisprudenciales contendrán todos los datos necesarios para su adecuada localización, y se ajustarán a los estándares de


Princesa, 29, 2. º 28008 Madrid > T 91548 82 81 > F 915 478 645 > iustel@iustel.com > www.iustel.com

3


citación en publicaciones jurídicas españolas o, en su caso, de los países a que correspondan las normas o sentencias citadas. Cuando se haga referencia a sitios de Internet, habrá que indicar expresamente, entre paréntesis, la fecha última en que fueron visitados.
Se autoriza a PORTALDERECHO S.A. a ajustar las referencias bibliográficas aportadas por los autores a las propias de la Editorial.

3. CUESTIONES RELATIVAS A LA PUBLICACIÓN DE LOS TRABAJOS

En relación con los derechos de autor, los autores pueden utilizar sus derechos para publicar sus trabajos en cualquier otra publicación, siempre en soporte papel (y no en soporte electrónico), con el único requisito de reconocer la previa aparición en la Revista General correspondiente, incluyendo el nombre y el dominio en la red de la Revista (http://www.iustel.com).

Los Consejos rectores de cada una de las Revistas Generales exigirán que los trabajos sean originales, si bien siempre cabrán excepciones si por la importancia o actualidad del tema, los equipos consideran de interés publicar un trabajo en la Revista General una vez difundido por otra publicación periódica de papel.

Se autoriza a PORTALDERECHO S.A. a la publicación en formato papel de los trabajos remitidos por los autores.
EVALUACIÓN: Recibido el artículo, el Subdirector / Secretario de la Revista General realizará acuse de recibo al autor y enviará el original a dos miembros del Consejo de Redacción o Equipo de Redacción, según la materia sobre la que verse el trabajo, para que emitan una primera valoración en un plazo máximo de dos semanas.

Pasada esta primera criba, se remite el artículo a dos evaluadores externos para que, de forma anónima y siguiendo el sistema del doble ciego, emitan una valoración sobre el mismo de conformidad con el formulario establecido al efecto y que se les remite junto con el trabajo a evaluar.
El Subdirector / Secretario notificará al autor el resultado del proceso de evaluación externa que puede ser:

-	Totalmente favorable.

-	Favorable, con indicaciones de modificación.

-	Desfavorable.

Si uno de los informes fuese favorable y otro desfavorable, el Subdirector / Secretario remitirá el trabajo a un tercer evaluador. De ser positiva esta tercera evaluación, el trabajo será finalmente publicado en la revista.

Los Consejos rectores de las Revistas Generales de http://www.iustel.com constituyen equipos independientes de valoración.


Princesa, 29, 2. º 28008 Madrid > T 91548 82 81 > F 915 478 645 > iustel@iustel.com > www.iustel.com
