# Supplementary material for: Peer Review in Law Journals
Source: Front Res Metr Anal. 2021 Dec 8;6:787768. doi: 10.3389/frma.2021.787768 (PMC8692876; doi:10.3389/frma.2021.787768)
Supplement: Supplementary file 3 [file DataSheet2.ZIP › DOCUMENT - 1698-1189_1.RTF]

1


INSTRUCCIONES A LOS AUTORES. REVISTAS GENERALES


1. CUESTIONES RELATIVAS AL ENVÍO DE ORIGINALES

Los trabajos deben presentarse en Microsoft Word.

Las contribuciones podrán enviarse por correo electrónico a siguiente dirección de http://www.iustel.com: revistas@iustel.com. La Editorial remitirá las mismas a la Secretaria de la Revista General de Derecho Penal (D.ª Carmen Gómez Rivero).

Adicionalmente se mantiene abierta la siguiente dirección de http://www.iustel.com: cac@iustel.com.

Será asimismo opcional el envío de originales por medio de diskette a la dirección postal que indique la Secretaria de la Revista General de Derecho Penal o a la siguiente dirección: C/ Princesa, n.º 29, 2.º dcha, 28008, Madrid.

Los archivos deben nombrarse con los apellidos del autor del trabajo, o con palabras que aludan al título o contenido del archivo cuando no se trate de un trabajo doctrinal, seguido de un punto y de la abreviatura de la sección correspondiente de la Revista. Las abreviaturas de cada sección son las siguientes:
a)	est. :	Estudios

`)	jur.euro.sel.:   Jurisprudencia. Reseñas jurisprudenciales. Europea. Selección

`)	jur.euro.com.:  Jurisprudencia. Reseñas jurisprudenciales. Europea. Comentario

d)	jur.tc.:	Jurisprudencia. Reseñas jurisprudenciales. TC	
e)	jur.ts.esp:	Jurisprudencia. Reseñas jurisprudenciales. TS. Especial	
f)	jur.ts.gen.:	Jurisprudencia. Reseñas jurisprudenciales. TS. General	
g)	jur.acu.:	Jurisprudencia. Acuerdos de Sala	
h)	jur.est.:	Jurisprudencia. Estudios	
i)	jur.cir.:	Jurisprudencia. Circulares e Instrucciones	

`)	leg.españa:    Legislación. España

k)	leg.euro.:	Legislación. Derecho penal europeo e internacional	
l)	crim.:	Notas criminológicas	
m)	pen.:	Derecho penitenciario	
n)	comp.:	Apuntes de Derecho comparado	
o)	ag.:	Agenda	
p)	mem.:	Memoria de actividades académicas	
q)	inmem.:	In memoriam	
r)	enl.:	Enlaces de interés	


Princesa, 29, 2. º 28008 Madrid > T 91548 82 81 > F 915 489 482 > iustel@iustel.com > www.iustel.com

2


`)	entrev.:Entrevistas

Ejemplos:	Berdugo Gómez de la Torre.est.doc 4-2005.jur.cir.doc

2. CUESTIONES RELATIVAS A LA EDICIÓN DE LOS TRABAJOS

Podrán remitirse a cada una de las Revistas Generales, para su publicación, todos aquellos artículos relacionados con la materia propia de cada una ellas.

Los trabajos podrán estar escritos en castellano, inglés, francés, italiano, alemán o portugués.

En todos los trabajos doctrinales, sea cual sea la lengua en la que se escriban, se habrá de indicar en castellano y en inglés, el título del trabajo, el sumario, el resumen y palabras clave.

El tipo de letra será Arial 10 con interlineado sencillo. El texto puede contener hipervínculos a páginas web y notas al pie.

Los documentos deberán encabezarse con el título del trabajo (que debe ser breve y reflejar el contenido del análisis doctrinal en su totalidad con el fin de que, con su inclusión en el sumario, éste quede claro, conciso y concreto), el autor y su cargo académico, o actividad que desempeña, así como, en su caso, la Universidad a la que pertenece, o la Institución en la que desempeña su actividad. Asimismo debe aportarse su e-mail de contacto. A falta de esta dirección de correo electrónico PORTALDERECHO S.A. facilitará la siguiente: revistas@iustel.com.

El título deberá ir centrado, en letra mayúscula y en negrita.

El nombre y apellidos del autor irán en letra mayúscula y su cargo en letra minúscula. Ambos en letra redonda, sin negrita y centrado.

Cada uno de los epígrafes en los que se divida el trabajo irán centrados. Los primeros epígrafes se presentarán en letra mayúscula, en numeración romana, centrados y en negrita; los primeros subepígrafes se presentarán en letra minúscula, en numeración arábiga, centrados y en negrita; a partir de aquí, los posibles siguientes subepígrafes irán en letra minúscula, en numeración arábiga, centrados y sin negrita en la secuencia: 1.1, 1.2, 1.3: 1.3.1, etc.

La extensión de los trabajos será (de forma aproximada) la siguiente:

-	Estudios: 30 páginas

-	Comentarios: 10-20 páginas

-	Notas: 2-5 páginas

-	Recensiones y comentarios de libros: 5-10 páginas

-	Notas de libros: 2 páginas


Princesa, 29, 2. º 28008 Madrid > T 91548 82 81 > F 915 489 482 > iustel@iustel.com > www.iustel.com

3


Los artículos de las secciones doctrinales y cuando la extensión de los mismos lo aconseje deberán contener un sumario y un breve resumen de 6 a 10 líneas redactado en el idioma del artículo, en castellano y en inglés.

Estos mismos artículos deberán acompañarse de las PALABRAS CLAVE (no más de 5), en el idioma original, en castellano y en inglés.

A falta del cumplimiento de cualquiera de estos criterios, se autoriza a PORTALDERECHO S.A. a realizar las actualizaciones editoriales necesarias.
Citas: Las citas de los trabajos deberán ir en notas a pie de página y no en notas al final. Las referencias bibliográficas, legislativas o jurisprudenciales contendrán todos los datos necesarios para su adecuada localización, y se ajustarán a los estándares de citación en publicaciones jurídicas españolas o, en su caso, de los países a que correspondan las normas o sentencias citadas. Cuando se haga referencia a sitios de Internet, habrá que indicar expresamente, entre paréntesis, la fecha última en que fueron visitados.

Se autoriza a PORTALDERECHO S.A. a ajustar las referencias bibliográficas aportadas por los autores a las propias de la Editorial.

3. CUESTIONES RELATIVAS A LA PUBLICACIÓN DE LOS TRABAJOS

En relación con los derechos de autor, los autores pueden utilizar sus derechos para publicar sus trabajos en cualquier otra publicación, siempre en soporte papel (y no en soporte electrónico), con el único requisito de reconocer la previa aparición en la Revista General correspondiente, incluyendo el nombre y el dominio en la red de la Revista (http://www.iustel.com).
Los Consejos rectores de cada una de las Revistas Generales exigirán que los trabajos sean originales, si bien siempre cabrán excepciones si por la importancia o actualidad del tema, los equipos consideran de interés publicar un trabajo en la Revista General una vez difundido por otra publicación periódica de papel.
Se autoriza a PORTALDERECHO S.A. a la publicación en formato papel de los trabajos remitidos por los autores.

EVALUACIÓN: Recibidos los originales, la Secretaria de la Revista General realizará acuse de recibo al autor, los trabajos se someterán a la evaluación de al menos dos árbitros externos, siguiendo el sistema de evaluación doble ciego. Los autores recibirán información del eventual rechazo de sus trabajos, de las reformas requeridas para la aceptación definitiva o de dicha aceptación.

Si uno de los informes fuese favorable y otro desfavorable, la Secretaria remitirá el trabajo a una tercera persona para que realice la calificación definitiva.


Princesa, 29, 2. º 28008 Madrid > T 91548 82 81 > F 915 489 482 > iustel@iustel.com > www.iustel.com

4


Los Consejos rectores de las Revistas Generales de http://www.iustel.com constituyen equipos independientes de valoración.


Princesa, 29, 2. º 28008 Madrid > T 91548 82 81 > F 915 489 482 > iustel@iustel.com > www.iustel.com
