# Supplementary material for: Peer Review in Law Journals
Source: Front Res Metr Anal. 2021 Dec 8;6:787768. doi: 10.3389/frma.2021.787768 (PMC8692876; doi:10.3389/frma.2021.787768)
Supplement: Supplementary file 3 [file DataSheet2.ZIP › DOCUMENT - 1699-3950_1.RTF]

 About the Journal 
Focus and Scope
Relaciones Internacionales is a journal of the Universidad Autónoma of Madrid (Spain) which is published electronically every four months. Its objective is the promotion of the study and the academic debates that surround the complex international reality, and to do so from an interdisciplinary perspective. It shows special interest in those theoretical approaches that, from the discipline of International Relations, emphasize a need to develop a “situated” account of the histories, in plural, of international relations, past and contemporary, as well as those theoretical approaches that advocate analysing among other things: the role of ideas, discourses, and identities in the configuration of international structures of power.
From the creation of the journal in 2005, within the framework of the Grupo de Estudios Internacionales (GERI-UAM), the main objective has been to extend and disseminate the specialist academic international relations literature –especially the one developed in the theory of international relations – in the professional Spanish speaking academic setting. Moreover, it is to bring this literature –for the most part, Anglo-Saxon- to a growing Spanish speaking community of almost 500 million people globally (of which Spanish is the official language in more than twenty countries around the world). Thus, the internationalization of the discipline is sought by reaching out to this enormous linguistic community in their mother tongue, and it is for this reason that the journal has been published since its inception in Spanish.
This orientation has contributed to the publication having been constituted as a reference in the specialist international relations literature in Spanish, being awarded in 2019 with the certification of FECYT (Spanish Foundation of Science and Technology), which distinguishes Relaciones Internacionales as one of the most significant journals in the Spanish academic panorama. Thus, throughout the last decade, the growing research relevance and academic significance that the publication has acquired, mainly in the Ibero American context, has been recognized. In addition, in recent years, publications of global relevance for the English speaking literature are also being disseminated.
Both through the articles and in the other sections, the journal offers original and unpublished contributions from researchers all over the globe, as well as unpublished translations of classic international relations texts into Spanish. This helps to produce and spread the different approaches, tools and theoretical knowledge of international relations to this language. In this way, the publication enriches the reflection on the discipline in the Spanish speaking academic community. Moreover, it connects, internationally, the academic production in the field of international relations emanating both from the Anglo-Saxon and Spanish speaking worlds.
Relaciones Internacionales publishes three editions annually (February, June and October). Two of these are normally in monographic format, while the third is open, with a view to making space for all of the varied contributions which arrive regularly to the journal.

Peer Review Process
Relaciones Internacionales admits the presentation of unpublished and original articles, review-essays and reviews that deal with the field of International Relations. To send the manuscripts, it will be used the OJS system of the Journal's website (https://revistas.uam.es/relacionesinternacionales/), which allows online monitoring of all the processes. 
Papers, reviews and review essays send to Relaciones Internacionales will first undergo a process of internal review by the Editorial Team and Board. Once assessed, they will be discussed at a meeting of the Editorial Team:
?	for articles and review essays, the Editorial Team will decide on the appropriateness of submitting manuscripts to the external double-blind peer review process, which will determine their value for publication; 
`.	for reviews, the Editorial Team will decide on their publication. 
Referees may suggest modifications to the author or even refuse publication if they consider it does not satisfy minimum quality requirements or edition and style rules of the journal. Referees may: refuse publication, accept publication conditioned to major corrections, accept publication conditioned to minor corrections, or accept direct publication. Possibilities are:
0.	Double rejection: the manuscript will not be published and the author will be informed.
0.	One rejection and one acceptance with major corrections: a third evaluation is requested. If this third evaluation recommends rejection, the manuscript will not be published and the author will be informed. Otherwise, a third evaluation decision will replace the rejected publication evaluation.
?	Double acceptance with major corrections/acceptance with major corrections and acceptance with minor corrections: to be published, the author should accept and implement in his paper/review changes suggested by reviewers. The paper/review will be then sent again to the referees for their consideration and final decision. If one of the referees considers again that the paper/review needs major changes, the issue's coordinators will decide whether publishing the paper or not. Otherwise, the manuscript will be sent back to the author to introduce the latest minor changes and then will go through the editing process for his publication.
0.	Double acceptation with minor changes: the manuscript will be published, but the paper/review will be sent to the author to make needed changes. Once returned, the manuscript will go through the editing process for his publication.
?	Double acceptation: the manuscript will be published and the author will be informed. The manuscript will go through the editing process for his publication.
?	Discrepancies between peers (an acceptance and a rejection): a third evaluation is requested. If this third evaluation recommends rejection, the manuscript will not be published and the author will be informed. If the third evaluation requests major/minor changes or accepts the publication of the article, the first rejection of the evaluation will be replaced by the third one and the situation outlined in the previous situations will be addressed.
External double-blind peer review process estimated resolution time:
?	Papers: 3-6 months.
?	Review essays: 2-3 months.
?	Reviews: 1-2 months.
Review-essays will be of a maximum of three books and the books reviewed must not be older than two years old.
The editing requirements demanded by Relaciones Internacionales are reflected in the "Style Manual" and the "Article template" of the Journal, available on our website.
The journal also has the following extraordinary sections:
`.	Guest author: Articles written by specialists on the specific subject of the issue will be included in this section, without the need to pass the evaluation process. These are articles that do not meet the writing requirements (length, originality, etc.) but are of interest to the journal due to their authorship.
`.	Excerpts: One of the main objectives with which the project of Relaciones Internacionales began was to translate into Spanish those texts considered classic by specialists, to provide tools to the Spanish-speaking academic community that enrich reflection on international relations. This section is intended for this purpose.
`.	Dialogues: It is a space in which the theory of international relations leaves the margins of the academy, to see the social actors who also generate reflection in their work. It has usually an interview format.
To know in detail the editing and evaluation requirements required for the acceptance of articles, please read the "Style Manual" and the "Evaluation Manual". If you need more information, do not hesitate to contact us by email.
Finally, you can access our evaluation form by clicking here.

Publication Frequency
Relaciones Internacionales is published every four months at once. No new content is added between issues.

Open Access Policy
This journal provides free and instant access to all content. It firmly believes that allowing free public access to academic investigation supports the open exchange of knowledge.

The published contents are under a Creative Commons Attribution-NonCommercial-NoDerivative 4.0 International license. Thus it allows reproduction, distribution and public presentation with the requirement that the author of the text and the source is properly cited in a note on the first page of the article, as demonstrated by the citation recommendation appearing in each article. Content is not for commercial use nor for derivative works. The rights of the articles published belong to the authors or the publishing companies involved.


Publication Ethics
Read the Ethics Statement of Relaciones Internacionales.

Digital Object Identifier (DOI)
From 2017 (No. 34), the Relaciones Internacionales Journal adopted the use of the digital object identifier (DOI) 10.15366/relacionesinternacionales. This identifier is assigned to sections articles, fragments,  and those snapshot of society's publications approved for their quality by the Publications Service of the Autonomous University of Madrid.

Anti-plagiarism
To fight against plagiarism and to guarantee that every article is original content, submissions are precessed through Turnitin software.
Those articles that the editorial team corroborates that are not original or that do not comply with any of the points of the ethical code will be removed from the website. Similarly, if the authors find any type of error, they will communicate it to the editorial team and it will be corrected as soon as possible.

Reviewer Board
Reviewer Board 2017 (Issues 34, 35 & 36)
Pablo AGUIRRE CARMONA
Steinar ANDRESEN
Rafael de ASÍS 
Maria Caterina la BARBERA
Paulo BOTTA
Elisa BREY
Pilar BUENO
Francesco CARUSO
Carlos de CASTRO
Nicolás COMINI
Alessandra CORRADO
Oriol COSTA
Fred DÉCOSSE
Ekaterina DOMORENOK
Rosa M. EGEA FERNÁNDEZ
Celia ESTRADA
Ornela Romina FABANI
Romain FILHOL
Matías FRANCHINI
Fernando FRANCO
Elena GADEA
Jorge GARCÍA ARIAS
Rosa GILES CARNERO
Eloy GÓMEZ PELLÓN
Cristina GORTÁZAR
Mercedes GUINEA LLORENTE
Ari JERREMS
Pedro LIMÓN
Elena de LUIS ROMERO
José LUOS COLOMER 
Gracia MORENO AMADOR
Juana MORENO NIETO
María Fernanda MOSCOSO
Lau ØFJORD BLAXEKJÆR
Alejandro OLAYO
Karlos PÉREZ DE ARMIÑO
Carmen PÉREZ GONZÁLEZ
Antonio RAMÍREZ MELGAREJO
Silvia ROQUE
André SILVA 
Daniel SORANDO
Carlos SORIA RODRÍGUEZ
Ewa K. STRZELECKA
Verónica TRPIN
Jose YÁNEZ DEL POZO

Reviewer Board 2018 (Issues 37, 38 & 39)
Jaime ABEDRAPO ROJAS
Diego BARRÍA TRAVERSO
María BECERRA
Elisa BREY
José BRICEÑO RUIZ
Diego BUFFA
Jaume CAMPS
Carlos CAÑETE
Eduardo Andres CARREÑO
Cristian CORREA
Carlos R. FERNÁNDEZ LIESA
Ramón FOGEL
Javier FRANZÉ MUDANÓ
Lorenzo GABRIELLI
Rita GIACALONE
Clarisa GIACCAGLIA
Felipe GÓMEZ
Irene GONZÁLEZ
Carlos GONZÁLEZ VILLA
Marta IÑIGUEZ DE HEREDIA
Mbuyi KABUNDA
Misael Arturo LÓPEZ ZAPICO
Ana MARLENY
Paz MILET GARCIA
Yoan MOLINERO
José A. MORANDÉ LAVÍN
Jose Luis NEILA
Cristian OVANDO SANTANA
Lorena OYARZUN SERRANO
Luisina PERELMITER
Gisela PEREYRA DOVAL
Adrián PIVA
Jerónimo RÍOS SIERRA
Jorge RIQUELME RIVERA
Pablo RIVAS PARDO
Daniel RODRÍGUEZ SUÁREZ
Héctor ROMERO RAMOS
Carlos SANZ DÍAZ
Consuelo SILVA
Jose Ángel SOTILLO
Rocío VELASCO DE CASTRO
Mercedes di VIRGILIO
Juan WAHREN
Iker ZIRION

Reviewer Board 2019 (Issues 40, 41 & 42)
Cassio ALBERNAZ
Carlos Hugo ANGARITA-CALLE
Khadija ARFAOUI
Gennaro AVALLONE
Miguel Ángel BENEDICTO
Sheida BESOZZI
Susana BORRÀS
Paulo BOTTA
Elisa BREY
María del Pilar BUENO RUBIAL
Diego BUFFA
Odilon CALDEIRA NETO
José Miguel CALVILLO CISNEROS
Fernando CAMACHO
Alicia CAMPOS
Diego CHECA HIDALGO
Gloria CLAUDIO QUIROGA
Alejandro COLÁS
Gilberto CONDE
Paolo COSSARINI
Mariela CUADRO
José Luis DADER GARCÍA
Fernando DELAGE CARRETERO
Thierry DESRUES
Marina DIAZ
Tanja DRAMAC
Rafael DUARTE VILLA
Albert FARRÉ 
Yves FAURÉ
Lorenzo FELTRIN
Carmen Beatriz FERNÁNDEZ
Paola GANDOLFI
Rosa GILES
Julián GONZÁLEZ SCANDIZZI
Ignacio GUTIÉRREZ DE TERÁN GÓMEZ-BENITA
Ari JERREMS
Mbuyi KABUNDA
Mario LÓPEZ AREU
Marta LUCEÑO
Juan Antonio MACÍAS AMORETTI
Emerson MAIONE
Inmaculada MARRERO ROCHA
Guadalupe MARTÍNEZ FUENTES
Óscar MATEOS
Laura MIJARES MOLINA
Valentine M. MOGHADAM
Ricardo MOLERO
Gracia MORENO AMADOR
Susana MURILLO
Carlos MURILLO ZAMORA
Alberto PRIEGO MORENO
Jordi QUERO ARIAS
Jorge RAMOS TOLOSA
Florentino RODAO GARCÍA
Mónica SALOMÓN GONZÁLEZ
Marcelo SANTOS
Keith SMITH
Kilian SPANDLER
Marta TAWIL KURI
Beatriz TOMÉ
Mayra VALCARCEL
Clara della VALLE
Luciano ZACCARA
 
Reviewer Board 2020 (Issues 43, 44 & 45)
Gracia ABAD QUINTANAL
Manuel AHEDO
Sonia ALDA MEJÍAS
José Antonio ALDREY VÁZQUEZ
Luís ANDUEZA
Marianna Restum ANTONIO DE ALBUQUERQUE
Pablo ARCONADA LEDESMA
Ana Elisa ASTUDILLO SALAZAR
Gennaro AVALLONE
Bruno AYLLÓN
María Jesús BELTRÁN MUÑOZ
Marta BIAGI
Andrea BIANCULLI
Elisa BREY
José BRICEÑO
Raúl BRINGAS NOSSTI
Leiza BRUMAT
Diego BUFFA
Alicia CAMPOS
Eduardo CARREÑO
María Francisca CASADO CLARO
Emilio CERDÁ TENA
Alicia CHICHARRO LÁZARO
Gloria CLAUDIO QUIROGA
Sergio CORTES
Isabel COTILLAS FERNÁNDEZ
Teresa CUNHA
Humberto José DA ROCHA
Raquel María DE ALMEIDA ROCHA
Leandro DEL MORAL ITUARTE
Fernando DELAGE CARRETERO
Camilo Andrés DEVIA GARZÓN
Marina DÍAZ
Luís DOMÍNGUEZ CASTRO
Flavio Américo DOS REIS
André DUVENHAGE
Cayetano ESPEJO MARÍN
José Antonio ESQUIVEL
Cecilia ESTRADA
Guillem FARRES
Susana FERREIRA
Ruth FERRERO TURRIÓN
Sebastián FLORES DÍAZ
Carlos Antonio FLORES PÉREZ
Gustavo FONDEVILA
Carolina GALINDO HERNÁNDEZ
María GARCÍA
Javier Ignacio GARCÍA GONZÁLEZ
Miguel Ángel GARCÍA RUBIO
Marcial GARCÍA SUÁREZ
Luciana GHIOTTO
Clarisa GIACCAGLIA
Lianeth GOYAS
Nuría HERNÁNDEZ GARCÍA
Jaime HOOGESTEGER VAN DIJK
Jorge Daniel IVARS
Daniel JACOBO-MARÍN
Mbuyi KABUNDA BADI
Christopher KIESSLING
Robin LARSIMONT
María LOIS
Leandro MANINI WILLIAMS
Santiago MANUEL ÁLVAREZ CARREÑO
Karen Isabel MANZANO ITURRA
Liber MARTÍN
Sara MARTÍN GUTIÉRREZ
Javier MARTÍN-UCEDA
Andrés MARTÍNEZ MOSCOSO
Mayra MEDINA
Ángel MENÉNDEZ REXACH
Ricardo MOLERO
Ainhoa MONTOYA
Natalia MORAES MENA
Gracia MORENO
Alfredo Leandro OCON
Chiara OLIVIERI
Borja PALADINI ADELL
Jaime PASTOR VERDÚ
Ferran PÉREZ MENA
Oriol PUIG
Antonio RAMÍREZ MELGAREJO
Luís RODRÍGUEZ CALLES
Josep María ROYO
Leticia SALDI
Elena SCHUCK
Gema SERÓN AIRES
Jordi SERRANO-MUÑOZ
Salvo TORRE
Astrid ULLOA
Yetzy URIMAR VILLARROEL
Beatriz Eugenia VALLEJO
Carmen VIDAL
Silvia ZAMBRANO NOLES
