# Supplementary material for: Peer Review in Law Journals
Source: Front Res Metr Anal. 2021 Dec 8;6:787768. doi: 10.3389/frma.2021.787768 (PMC8692876; doi:10.3389/frma.2021.787768)
Supplement: Supplementary file 3 [file DataSheet2.ZIP › DOCUMENT - 1699-8154_1.RTF]

About the Journal
Focus and Scope
Publication Frequency
Open Access Policy
Archiving Policy
Statement on publication ethics and misconduct
Publication Fees
Advertisement policy
Facts and Figures
 
Focus and Scope
IDP. Journal of Internet, Law and Politics is an e-journal produced by the UOC's Faculty of Law and Political Science. It aims to communicate and disseminate research, analysis and investigation into the challenges and questions faced by law and political science due to information and communication technologies. The papers published are based on the results of original research.
The subjects covered by the journalare, generally speaking, those that spark reflection and discussion on the scope and form of the changes brought on by information and communication technologies – and the internet in particular – in the fields of law, politics, criminology, and public administration. The journal's articles look at the effects that technology has on fundamental rights, data protection, intellectual property and freedom of expression on the internet, cyber-crime, electronic administration, new forms of participation in politics or e-commerce, among other subjects. And they do so both from the point of view of theoretical reflection and by presenting specific experiences in some of these fields.
Each issue of IDP has a section with articles (sometimes organized as a dossier or special issue), a specific section on the UOC Faculty of Law and Political Science's academic and research activities, and a section on the new developments in legislation and jurisprudence.
 
Submission and publication languages
IDP’s primary publishing language is Spanish.
All papers published in IDP, whether submitted in Catalan, Spanish or English, are edited and revised for style and format. At the discretion of the editorial board, some papers may also be translated to Catalan, Spanish or English. Likewise, the title, abstract and keywords of all articles and reviews are published in Catalan, Spanish and English.
 
Publication Frequency
IDP. Internet, Law and Politics Journal publishes its full issue semiannual, in March and in October, although the publication of the articles is on-line first as of March 2019.
 
Open Access Policy
This journal provides immediate open access to its content on the principle that making research freely available to the public supports a greater global exchange of knowledge.
Authors may deposit their works in open-access repositories, whether before peer-review (as pre-prints) or after (as post-prints).
This journal does not ask for neither submission charges nor article processing charges (APC).
 
Archiving Policy
The archiving policy and long-term preservation of articles published in the journal IDP is provided by RACO (CSUC) and Internet Archive.
The preservation by LOCKSS is under consideration.
 
Statement on publication ethics and misconduct
Authorship | Peer review
The Universitat Oberta de Catalunya (Open University of Catalonia, UOC) is committed to upholding the highest standards of quality and integrity in the publication of scientific articles, in accordance with the guidelines set out in its own Code of Ethics (in Catalan), the Code of Conduct and Best Practice Guidelines for Journal Editors (Committee on Publication Ethics, COPE), and CSE's White Paper on Promoting Integrity in Scientific Journal Publications (Council of Science Editors, CSE). The UOC aims to satisfy the needs of authors and readers alike, ensuring the quality of articles published in its journals, protecting and respecting all rights pertaining to the content of articles, and respecting the integrity of all submissions and published work.
The Editorial Board of IDP undertakes to publish all corrections, clarifications, retractions and apologies as and when they are required. As part of its commitment to best practice, IDP makes publicly available the evaluation system for submitted articles and the criteria applied in the external peer review process. IDP regularly updates these criteria, which are intended to ensure the scientific relevance, originality, clarity and pertinence of published articles.
IDP maintains full confidentiality throughout the evaluation process, protecting the anonymity of authors and external reviewers, the reviewed content, the reviewers' report and any other communication issued by the editorial, advisory and scientific boards, as required. Equally, it applies the strictest standards of confidentiality to any clarifications, claims or complaints that an author may wish to refer to any of the journal's boards or to the external reviewers.
IDP undertakes to respect the integrity of all published work. As such, XXXXXX will be particularly vigilant in identifying and sanctioning cases of plagiarism. Any manuscript that is found to plagiarize published work will be removed from the journal or barred from publication, as the case may be. The journal will act as swiftly as possible in all such cases. In agreeing to the terms of the journal, authors undertake to ensure that the articles they submit and all of the associated materials contain only original work and that they do not infringe on the rights of third parties. In the case of shared authorship, a clear statement must be made to the effect that all authors have agreed to the content of the manuscript and that the work has not been published previously in any other form.
 
Authorship
The authors of articles submitted for publication must ensure that the material they submit to IDP is original work and that it does not contain fragments of work published either by themselves or by other authors. In submitting a manuscript, the authors must also guarantee the accuracy of the data presented therein, which must not have been altered to verify the experimental hypothesis or hypotheses put forward.
Authors must ensure that the materials consulted during the preparation of their article are the most recent and relevant in the field with which the research is concerned and that they have given due consideration to all current schools of thought on the subject matter.
Authors must clearly identify all those individuals who have made a significant scientific contribution to the conceptual design and planning of the study, the interpretation of the results and the writing of the article. The list of authors must be ordered hierarchically to reflect the degree of responsibility of each author and their respective roles in the study.
All authors accept responsibility for the content of the manuscript.
 
Peer review
Reviewers undertake to produce a critical, sincere, constructive and impartial evaluation of submissions and to complete their review in the shortest time possible, to ensure that the deadlines of the evaluation process are met.
Reviewers are only assigned to a manuscript if they have the necessary expertise in the relevant field and are not affected by any conflicts of interest.
The reviewers will submit a full and thorough report, complete with the necessary references, in compliance with the terms of the evaluation process and any applicable public standards, particularly when rejecting a submission. In addition, reviewers must notify the Editorial Board of any part of the manuscript that has already been published or is under consideration for publication in another journal.
Reviewers must ensure that they have no conflicts of interest with regard to the research presented in the article, the author/s, and the sources of funding for the project.
Once the Editorial Board has verified that the article conforms to the standards on content and style indicated in the editorial criteria, it will send the article to two anonymous experts, not affiliated to the authors' home institution, for a double blind review.
The reviewers' evaluation of the article will focus on its interest to the scientific community, the novelty of its contribution to existing knowledge of the subject matter, the accuracy of the relationships it establishes with other work, the critical judgement displayed, the bibliographic references used, the quality of writing and presentation of the manuscript, and other standard considerations. Where necessary, recommendations will be made as to how the manuscript can or should be improved.
The Editor of IDP will examine the reviewers' report and notify the author/s of the outcome (fit for publication without changes; fit for publication with minor corrections; fit for publication with major corrections; not fit for publication) by sending an email to the address from which the manuscript was submitted. The reviewers' comments and suggestions will be provided for consideration by the first author.
If the manuscript has been deemed fit for publication with minor or major corrections, the authors must submit a revised version which addresses the external reviewers' comments and suggestions. The authors may also attach a rebuttal letter for the Editorial Board in which they explain the specific changes made to the original submission.
The Editorial Board will determine whether the revised manuscript is fit for publication on the basis of the changes made and the degree to which they successfully address the reviewers' comments and suggestions. The Editor will then notify the authors of the final decision.
 
Publication Fees
Since the publication costs for IDP are covered by the editing university (UOC) internal budget, authors do not need to pay an article-processing charge (APC) and no waivers are necessary.
 
Advertisement policy
The journal only displays advertisements that are of relevance to its scope and will be of interest to the readership (e.g. upcoming conferences). All advertising space is provided free of charge and the editor and publisher have the right to decline or withdraw adverts at any point.
 
