# Supplementary material for: Peer Review in Law Journals
Source: Front Res Metr Anal. 2021 Dec 8;6:787768. doi: 10.3389/frma.2021.787768 (PMC8692876; doi:10.3389/frma.2021.787768)
Supplement: Supplementary file 3 [file DataSheet2.ZIP › DOCUMENT - 1825-0173.RTF]

INSTRUCTIONS FOR AUTHORS

Diritto&Questioni Pubbliche is published twice a year, in June and December. The journal publishes articles in Italian, English, French and Spanish.

All publication proposals should be sent to: direzione@dirittoequestionipubbliche.org.


The articles are first evaluated by the journal's editors-in-chief and, if they are of interest to the journal, are then sent to an external specialist (or two if deemed necessary) for evaluation using the double-blind peer review method. The final decision on publication will take into account, but not be bound by, the peer review evaluation. The result of the peer review process is normally communicated to the authors within 45 days of receipt of the proposal. The issues in which the article can be published will also be indicated in the reply.

The double-blind peer review does not apply for book reviews and the replies of the author in the

Discussion section.


Authors are further expected to conform to the editorial rules below.


EDITORIAL RULES

1. FORMAT


Each issue is divided into the following sections: Monographic Section, Studies, Discussions, Reviews, Ratio Decidendi.

The length of the articles should not exceed:

-	for the Monongraphic Section, Studies, Ration Decidendi sections, 80.000 characters (spaces included) in the body text;

-	for the Discussions section, 40.000 characters (spaces included) in the body of the text (replies to critics by an author whose work is the center of the discussion can exceed this limit);

-	for the Review section, 15.000 characters (spaces included).

Font

Contributions should be written using the following font and font size: Times New Roman, 12 (10 for notes).

Sections

Contributions should be divided into sections which will be numbered and titled according to the following system: 1. First section; 2. Second section; 2.1. Sub-section; and so on.


The paragraph title should be separated from the preceding text by TWO lines; the text of the paragraph should be separated from the title by ONE line; the title of the subparagraph should be separated from the text of the preceding paragraph by ONE line.


Untitled subparagraphs are permitted; however, their text should be equally separated from the text of the preceding paragraph by ONE line.

Body of text

Quotations longer than three lines should be written using low quotation marks, Times New Roman font, font size 11, and 0,5 return.


2. ABSTRACT, KEYWORDS, DATA

All articles should be accompanied by:

0()	an English title

0()	an Abstract (max. 500 characters, including spaces) and keywords (max. five) in English

0()	Author's data: authors should indicate a valid email address, their qualification and their academic, or other institutional affiliation.


Articles written in Italian, Spanish or French should be also accompanied by Abstract and keywords in Italian, Spanish or French, respectively.

Abstracts and keywords are not required for Reviews.


3. REFERENCES TO FOOTNOTES

References to footnotes should be placed at the top of the corresponding word. They must precede the full stop, comma, colon and semicolon; they should follow the question mark, parentheses, hyphen and quotation marks.


4. QUOTATION MARKS

Low quotation marks («…») should be used when quoting a text from another source (the full stop must always be placed outside of the quotation marks, even if they already contain question/exclamation mark, or ellipsis.

For any other purpose, high quotation marks should be used ("…"), particularly for quotations included in texts already quoted with low quotation marks.

Titles of works reported in the text should be written in italic, and must not be included within quotation marks.


5. QUOTATIONS

As already indicated, quotations must be included within low quotation marks. Any intervention by the author to the quoted text must be indicated using squared parentheses. In case of omissions, ellipsis must be included within squared parentheses.


Indications outside of the quoted text should be put within normal parentheses. Eg. «quod erat demonstrandum» (Italics mine).

6. REFERENCES


a. Literature (monographs and articles)

References to literature can be indicated either in the main text or by using footnotes. For a high numbers of references, or for particularly long ones, notes are preferred.

The author-year method should be adopted, possibly followed by the comma, and by the quoted page, based on the following models:

RAZ 1975. RAZ 1975, 121.

RAZ 1975, 121 and 123. (e for Italian, y for Spanish, et for French) RAZ 1975, 121-128.

RAZ 1975, 121 f. (when referring to only one following page)
RAZ 1975, 121 ff. (when referring to more than one following pages).

(For articles in Italian, Spanish or French use s. and ss. rather than f. and ff.)

When the original's publication year does not correspond to the quoted version and the author retains it necessary to indicate the former in the text, she may add it in square brackets like this:

HART 1991 [1961]

To indicate chapters: ch./chs. for English, cap./capp. for Italian. To indicate paragraphs: sec. for English, par. for Italian.

To indicate notes: nt. for all languages.

In case of two co-authors or editors, both their surnames must be indicated, following the order of the quoted edition, separated by a comma.

Eg. ALCHOURRÓN, BULYGIN 1971.

In case of three or more co-authors or editors, only the first author's name must be indicated, in the same order as the quoted edition, followed by "et al.".

Eg. VERSCHUUREN  et al. 1971.

In case more works of the same author published in the same year are quoted, these should be labelled with a,b,c.. Eg:

RAZ   1975a.

RAZ 1975b.

Corresponding letters should be reported in the bibliography as well.


If two authors with the same surname are quoted, initials should be included as appropriate to distinguish them. Eg:

ROSS  A. 1980.

ROSS D. 1997.


b. Judgements and legal documents

Judgements or legal documents should not be included in the bibliography. Footnotes should therefore contain all required elements for identification of the text. Although there are no specific criteria for footnotes in this case, authors are required to use a coherent style.

Where a note refers to the same judgement or document as the previous note, Ibid. (written in italic) should be used, followed by a comma and page number if it differs between the notes.

If, however, a note refers to a judgement or document already quoted in a different note than the one immediately precedent, it will be enough to use an abbreviated citation followed by the cit. (in Roman type).

For all other cases, the same rules as those concerning the general literature (6.1) must be applied.


7. BIBLIOGRAPHY

Articles must be accompanied by a bibliography of quoted tests, which should be indicated as

References (Riferimenti bibliografici for Italian, Referencias bibliograficas for Spanish). References should respect the following criteria.

a. Monographs

()	Only one author:

RAZ J. 1975. Practical Reason and Norms, Oxford University Press.

(When the author has more than one name, initials must not be separated. Es. HART H.L.A.)


()	When referring to editors (ed.) and (eds.) should be used. In the case of only one editor:

HILPINEN R. (ed.) 1971. Deontic Logic: Introductory and Sistematic Reading, Riedel.

In the case of two or more editors, all of them should be indicated in the order adopted by the referenced edition, separating names with commas:

GIANFORMAGGIO L., LECALDANO E. (eds.) 1986. Etica e diritto. Le vie della giustificazione razionale, Laterza.


VERSHUUREN B., WILD R., MCNEELY J.A., OVIEDO G. (eds.) 2010. Sacred Natural Sites. Conserving Nature & Culture, Earthscan.


()	When the author wishes to add further information, such as references to the original edition, the translator, and the like, these should be indicated in parentheses at the end of the reference. Example:

FOUCAULT M. 1977. Discipline and Punish. The Birth of Prison, Pantheon (or. ed. Surveiller et punir. Naissance de la prison, Paris, Gallimard, 1975, tr. by A. Sheridan).


b. Journal articles

The complete journal title should be indicated in low quotation marks, followed by the volume, year and initial page. Eg.:

COLEMAN J.L. 1988. The Structure of Tort Law, in «Yale Law Journal», 97, 1988, 1233 ff.

For all other cases, the same indications as those provided for monographs should apply.

`.	Articles in volume

()	If an article is part of a volume by the same author:

FEINBERG J. 1980. The Idea of a Free Man, in ID., Rights, Justice, and the Bounds of Liberty, Princeton University Press, 3 ff.

()	If an article is part of either a volume by different authors or of an encyclopedia:

MACCALLUM  G.C. 1972. Negative and Positive Liberty, in LASLETT P., RUNCIMAN  W.G., SKINNER

Q. (eds.), Philosophy, Politics and Society. Fourth Series, Blackwell, 174 ff.


d. Web pages

If the quoted contribution is found on a web page, the corresponding bibliography should indicate the web page and the day in which it was accessed, as follows:

For English: Available at: http… (accessed….)

For Italian: Disponibile in: http… (consultato il….)

For Spanish: DIsponible en: http… (consultado el….)


`.	Special cases

()	If more works of the same author, edited on the same year, are quoted, they should be indicated by the letters a,b,c…Eg.:

RAZ J. 1975a. Practical Reason and Norms, Oxford University Press.

Corresponding letters should be reported in the references as well. Eg.: RAZ 1975a.


()	A literary work in the process of being published with a defined collocation must be referenced normally, followed by "forthcoming".

Eg. SMITH P. 2018. Law and Ontology, in «Legal Theory 2019», forthcoming.


()	In case of an unpublished manuscript, the corresponding footnote should indicate the name of the author and the abbreviation (mns.). Example:

FERDINAND  (mns.)

For the extended bibliographic reference, the following model should be adopted:

FERDINAND P. (mns.). Rules and Rulers, unpublished manuscript.


f. Judgements and legal documents

No final bibliography is expected for judgements and normative documents. The complete reference should be indicated within a footnote, based on the above-mentioned criteria.

8. INTERNAL CROSS REFERENCES

Internal cross references should be made indicating either the number of paragraph or of the note, as follows:

For English:

(see above/below, 2.1) (see above/below, nt.16)

For Italian:

(vedi supra/infra, 2.1) (vedi supra/infra, nt.16)


9. REVIEWS

Book reviews should not have original titles. Instead, they should be entitled as follows:

Review of [complete information about the work (author, title, subtitle where applicable, edition, series, year of publication, editor of Italian edition in case of translation, etc.).


Example: Review of F. Urbina, Against Proportionality and balancing, Cambridge University Press, 2017.


10. PROOFREADING

Proofs will be sent to the corresponding author by email for correction of typographical errors only. Authors are expected to return the revised manuscript within ONE WEEK at the latest.
