# Supplementary material for: Peer Review in Law Journals
Source: Front Res Metr Anal. 2021 Dec 8;6:787768. doi: 10.3389/frma.2021.787768 (PMC8692876; doi:10.3389/frma.2021.787768)
Supplement: Supplementary file 3 [file DataSheet2.ZIP › DOCUMENT - 1825-5167.RTF]

HOW TO SUBMIT A MANUSCRIPT TO E&P


Etica & Politica / Ethics & Politics accepts for publication original and previously unpublished manuscripts. Articles already published elsewhere will not be considered.


Submissions should be sent to: manuscriptsubmission.ep@gmail.com


Manuscripts can be submitted in one of the following languages: Italian, English, French, German, Portuguese, Spanish.

Authors for whom one of the accepted languages for publication is not their first language must have their manuscript edited by a native speaker expert and provide a declaration confirming that their manuscript was edited for proper language, grammar, punctuation, spelling, and style.

All citation formats are accepted as far as consistently used within the manuscript. Although E&P does not set a word limit for manuscripts, we strongly

recommend authors to not exceed 12000 words.


All submissions must be sent in a two separate Word.doc files format:

0)	a manuscript entailing following information (first page): article's title; author's name, affiliation (Department, University) and email address; abstract (max. 200 words) in English (only); keywords (max. 5) in English (only);
0)	an "anonymized" manuscript, so to facilitate our double blind-review process.


All manuscripts are evaluated, first, by the Editors in-chief and/or members of the Advisory Board for "desk acceptance" /"desk rejection". In case of acceptance, manuscripts

are sent to anonymous external referees complying with a policy of double-blind review.


We always seek to review manuscripts within two months upon receival, notifying authors of the acceptance, rejection or need for a revision and resubmission (R&R) of the paper. However, due to the high amount of submissions we receive, authors should consider that the review process might take longer.
