# Supplementary material for: Peer Review in Law Journals
Source: Front Res Metr Anal. 2021 Dec 8;6:787768. doi: 10.3389/frma.2021.787768 (PMC8692876; doi:10.3389/frma.2021.787768)
Supplement: Supplementary file 3 [file DataSheet2.ZIP › DOCUMENT - 1826-8269.RTF]

Jura Gentium

Centro di filosofia del diritto internazionale e della politica globale
℅ Dipartimento di Scienze Giuridiche Università degli Studi di Firenze

via delle Pandette, 35 50127 Firenze (Italy) C.F. 9413491081

E-mail info@juragentium.org Web-site: www.juragentium.org


PEER REVIEW


The Journal Jura Gentium sends on the submitted articles to external referees according to the following criteria:

0.	Preliminary evaluation (formal and substantial) by the Editorial Board (rea - dings, interviews, interventions in forum and/or scientific discussions, introductions, afterwords, won't be sent on to referees). The preliminary evaluation aims at ascertaining that the proposals meets basic criteria in terms of the focal interests of the Journal, editorial line, adequacy from a scientific point of view.

0.	Identification of two external referees on the basis of specific skills, to whom the Editorial Board sends the anonymized contributions following a double blind review. The referees are professors, lecturers, scholars, experts active in Universities and in Research Institutes in Italy and abroad. In case of monographic issues, edited by scholars who are not members of the Editorial Board, the contribution can be sent on to one external referee instead of two, being the external editor the second one.

0.	In case the external referees disagree on the acceptance of the paper, a third referee can be asked to review the paper.

0.	In case the referees judged that the paper is acceptable after modification and/or integrations, the article will be sent on to the author.

0.	After this mentioned phase, the article will be sent again to the external referees for the final acceptance or the final rejection. The Editorial Board may check the compliance of the paper with referees' requests, in case the required interventions by the author involve a minor revision.

0.	The full process must not require more than six months.

0.	Every four years the Journal publishes the list of the referees of the previous three years.

0.	The Journal will save all the filled out peer-reviewed forms.

0.	The Journal uses a peer-review model that meets the criteria described above.
