# Supplementary material for: Peer Review in Law Journals
Source: Front Res Metr Anal. 2021 Dec 8;6:787768. doi: 10.3389/frma.2021.787768 (PMC8692876; doi:10.3389/frma.2021.787768)
Supplement: Supplementary file 3 [file DataSheet2.ZIP › DOCUMENT - 1827-7942.RTF]

SUBMISSION

Authors interested in publishing their articles are kindly requested to send them via e-mail with attached files (Word format) to the following address: ombretta.fumagalli@unicatt.it.

Papers submitted to «JUS» will be subjected to 'double-blind peer review'.


GUIDELINES
Articles must be written in Italian, English, French, Spanish or Deutsch.

Articles should contain the following informations:

?	Title / Author(s)

?	Author's Footnote

The Author's footnote should contain the institutional affiliation with complete address and email address.

?	Abstracts and Key Words

A brief abstract (not exceeding 150 words) should be included before the main text of the paper along with up to five key words or phrases, separated by commas, which describe the subject matter covered

?	Article Text

?	References


ARTICLE TEXT

Headings

The main body of the article should be divided by appropriate numbered section and subsection headings.

Abbreviations should be defined at first mention and used consistently thereafter.

The article should not exceed 25 pages (3000 characters per page).

References

Bibliographic references should include only those works cited in the text. Accuracy of citation is the author's responsibility.

Examples:

Reference to a journal publication:


1

H. Muller, De formis iuridicis corresponsabilitatis in Ecclesia, in Periodica de re morali canonica liturgica 69 (1980), p. 303 ss.

M. Bianchi, Il giurisdizionalismo, in Stato, Chiese e pluralismo confessionale, Rivista telematica (www.statoechiese.it) 2012, n. 20

O. Fumagalli Carulli, Dietro il volto di Agostino Gemelli, in L'Osservatore Romano, 6 giugno 2009

Reference to a book:

O. Fumagalli Carulli, Il governo universale della Chiesa e i diritti della persona, Milano, 2003, p. 125 ss.

Van Lierde – A. Giraud, Le Sénat de l'Eglise. Le Sacré Collège, Paris, 1963

G. Alberigo (a cura di), Storia dei concili ecumenici, Brescia 1990

Web references

As a minimum, the full URL should be given and the date when the reference was last accessed. Any further information, if known (DOI, author names, dates, reference to a source publication, etc.), should also be given.


Editorial style

In order to avoid common problems of style:

1. use quotation marks

a) when a standard term is used in a nonstandard way: 'nnnnnn' b) to indicate the beginning and ending of a direct quotation: "nnnnnn";

2. italics may be used only sparingly for words in foreign languages, as well as to introduce and emphasize important terms.

PROOFS

Page proofs will be sent to the author for correction of typographical errors only. In the case of two or more authors please indicate to whom the proofs should be sent.

OFFPRINTS

One copy of the issue in which their article appears will be sent free of charge to each named contributor as well as the PDF file of the article.

Additional  copies  may  be  purchased  at	authors'  expenses  at  proof  stage.	
If  you  need  further  information,  please	contact  Vita  e  Pensiero  editorial	
office (redazione.vp@unicatt.it).		


2
