# Supplementary material for: Peer Review in Law Journals
Source: Front Res Metr Anal. 2021 Dec 8;6:787768. doi: 10.3389/frma.2021.787768 (PMC8692876; doi:10.3389/frma.2021.787768)
Supplement: Supplementary file 3 [file DataSheet2.ZIP › DOCUMENT - 1846-3010.RTF]

264	PILAR - Croatian Journal of Social Sciences and Humanities / Year IV (2009), Issue 7(1)-8(2)		
				


Instructions to Contributors

Articles published in the Pilar Journal will be protected by copyright, but the Journal reserves the right to first publication. Manuscripts which are accepted for publishing (or have already been published in the Journal) could be published only by permission of the editor, but only if their publication in the Journal is specifically mentioned.

Manuscripts should be submitted on a computer disc in the Microsoft Word or RTF formats and printed in two copies with double line spacing. The following should be provided: the title of the article, the author's name and surname, the author's title, the name and address of the employing institution, home address as well as the e-mail address.

Manuscripts are subject to double anonymous reviewing. They are categorised into the following:

0.	original scientific paper. The work is characterised by original conclusions or it reveals the previously unpublished original results of scientifically conceived and conducted research;

0.	review article. The work contains a thorough and inclusive critical review on a certain topic, but without significantly original results;
0.	preliminary communication. The work contains the first results of research which is being done. Due to their topicality the results require immediate publishing, but without any level of foundedness (required for the scientific paper);

0.	scientific work. The work contains knowledge and experience relevant to a cer-

tain profession, but it has no characteristics of the scientific text.

The recommended length of work should vary from 16 to 24 printed pages. With each manuscript the following should be enclosed: an abstract which should not exceed ten lines, a list of not more than five key words, and a summary, varying between 15 and 20 lines. Book reviews, essays and reviews are not subject to reviewing and should not exceed 150 lines.

The editorial board reserves the right to adapt the manuscript to the journal's standards and to the standard Croatian language, or to the corresponding foreign language if the international issue is in question.

Manuscripts will not be returned.

Texts should contain notes which provide additional information and complete the bibliography, as it is not published separately. The notes should include the author's name and surname, the title of the work, the publisher's name, the place and year of publication and, as a rule, the reference page.
