# Supplementary material for: Peer Review in Law Journals
Source: Front Res Metr Anal. 2021 Dec 8;6:787768. doi: 10.3389/frma.2021.787768 (PMC8692876; doi:10.3389/frma.2021.787768)
Supplement: Supplementary file 3 [file DataSheet2.ZIP › DOCUMENT - 1846-5412.RTF]

GUIDELINES FOR REVIEWERS FOR THE ANNUAL OF SOCIAL WORK

LJETOPIS

SOCIJALNOG RADA

Thank you for deciding to review an article for the Annual of Social Work. Your review is of great importance to us because it ensures the quality of papers and helps to maintain the journal standards in the area of social work. As a part of your review you should provide the author(s) with appropriate and constructive feedback and in your assessment of the text you should use neutral language. In order to provide you with all the necessary information, we have prepared for you the guidelines for reviewers of the Annual of Social Work with instructions which should make your reviewing task easier.


By accepting the reviewing task, you estimate that you have the necessary experience and competence to review the works in the foreseen period of time, you agree to the confidentiality of the reviewing process and commit to providing an objective review of the submitted paper emphasizing its weaknesses and strengths, so that the author could finalize the work according to the given remarks and recommendations.


In case of any conflict of interest regarding the work we send you for review (for example, if on the basis of the methodology of research you identify that you have a business relationship with the author, if you have already reviewed the work for another journal, if you are a part of the project on the basis of which the author has written the paper etc.), please notify the editorial board.

We would also like to ask you to notify the editorial board in case of suspicion of plagiarism.


In the Annual of Social Work every article accepted for review is submitted to a double-blind peer review. After the review, the editorial board will notify the authors of the results of the reviewers' assessment. In case the opinions of the reviewers are not coordinated, the editorial board may send the paper for a third blind review.The Annual of Social Work publishes articles in the Croatian and English language. Publishing in the Annual of Social Work is free for the authors.


Editorial recommendation

It is possible that the e-mail server identifies e-mails generated by the online journal system (Open Journal System) as spam. To ensure that you securely receive e-mails from Annual of social work, add the journal e-mail address to the list of secure contacts on the server you are using or regularly browse the spam mailbox.


2

LJETOPIS

SOCIJALNOG RADA

1.  THEMATIC AREA OF THE JOURNAL

The Annual of Social Work problematizes current insights about the theory and methodology, scientific research and the practice of social work, as well as the insights from other scientific areas related to social work which are important for understanding social issues and conducting social interventions more effectively. Alongside scientific and professional articles, the journal also publishes translations of selected texts which are of special significance for understanding contemporary social work, as well as reviews of conferences, books and journals from the field of social work and other humanities and social sciences disciplines important for social work.


0.	STANDARDS OF CLASSIFICATION OF SCIENTIFIC AND PROFESSIONAL ARTICLES

The reviewing process includes recommendations for the classification of the reviewed manuscript. Articles in the Annual of Social Work can be classified as scientific or professional.


Original scientific article			
Preliminary communication	Scientific articles		
Review article			
Professional article	Professional articles		
Practical experience			
			


0)	Original scientific article

Original scientific article contains original theoretical discussions and results of new research. This includes discussions about the fundamental theoretical issues in social work and social work practice, as well as a wide range of applied research (e.g. exploratory, evaluation and action research). The important qualities of original scientific articles are a clearly defined and consistently represented methodology, as well as a critical approach to findings and possible weaknesses of the conducted research. Qualitative and quantitative approaches to research are of equal value.


3

LJETOPIS

SOCIJALNOG RADA

0)	Preliminary communication

Preliminary communication is also a scientific article which presents results of an ongoing research, with the first results being issued to the public because of their relevance. This article contains new scientific data and has a clear methodological approach, but the results are still not considered final and cannot be verified.


0)	Review article

Review article comprises a comprehensive outline of the state and development trends of certain areas of social work theory and social work practice as well as of methodological approaches, the implementation of certain theoretical approaches and the analysis of practice. The article also contains the critical stance of the author as well as a review of relevant literature on the issues in question.


0)	Professional article

Professional article is primarily focused on the improvement of practice and, with the help of knowledge on the relevant theoretical approaches and sources, primarily deals with practical experiences of the author and issues related to practice.

Practical experiences are short reviews of the work of experts based on practical work which contain at least 1500 words. Practical experiences are expert contributions which can deal with some of the following topics:

?	the experience of working with clients through the implementation of different work methods and interventions,

?	organizational structure and working conditions in an organization/institution in the social welfare system (and beyond),

?	the challenges in the jurisdiction of a certain institution/organisation when working with a client – examples from practice (regulatory changes in the social welfare system, review of the enforcement of changes and amendments to the laws etc.),

?	description of cooperation of institutions inside and outside of the system as well as experiences gained from cooperation of different professional teams,

?	presentation of project activities and their results in the organisations/ institutions within the social welfare system (and beyond),

4

LJETOPIS

SOCIJALNOG RADA

?	experiences from professional trainings, conferences, seminars, round tables and the possibilities of their implementation in practice.


0.	REVIEWER'S RECOMMENDATION

As a part of the reviewing task in the Annual of Social Work, the reviewer can make one of the following recommendations:

?	Accept without revision: The article fully meets the standards of the journal. The reviewer can point to minor technical and content-related alterations.

?	Accept with suggested revisions: The article is suitable for publication in the Annual of Social Work. The recommended alterations should be made before publishing. The reviewer can ask for another review after implementation of revisions. Also, the editorial board can ask the reviewer to compare the revised version with the reviewer's suggestions.

?	Revise and return for review: The article is potentially suitable for the Annual of Social Work, but the reviewer thinks that the article requires major revisions and suggests another review after revision.

?	Suggest for publishing elsewhere: The reviewer believes that the article does not suit the thematic area of the journal and that it would be more suitable for publication in another journal.

?	The article is not suitable for publication: The text does not fit the standards of the Annual of Social Work and is not recommended for publishing.


0.	GUIDELINES FOR REVIEW

During the review process, please pay attention to and answer the following questions which refer to the standards and the quality of the article that is being reviewed:

0.	Originality – is the paper an original contribution or has it already been published? Does it constitute a significant scientific or technical contribution to the field? According to your knowledge, is there a significant overlap with previously published results?

0.	Importance for the reader – is the paper interesting and relevant for the reader (possibly specify categories of readership)?

0.	Is the topic of the paper appropriate for the journal?


5

LJETOPIS

SOCIJALNOG RADA

0.	Does the title reflect the major points of the paper?

0.	Does the abstract reflect the content of the paper? Does it contain appropriate keywords?

0.	Does the paper have an appropriate structure and is the writing clear?

0.	Are there references to relevant sources? Is something omitted?

0.	If the work contains figures and tables, do they contribute to the understanding of the paper or are they redundant?


FOR EMPIRICAL RESEARCH ARTICLES

0.	Are the purpose and the aims of research and hypothesis / research questions adequately stated?

0.	Is the methodology of research described clearly?

0.	Is the chosen research methodology suitable for examining the identified issues / providing answers to research questions?

0.	Has the research taken into account the accepted ethical standards related to research in social work and other helping professions?

0.	Are the research results presented and explained clearly? Are the results credible?

0.	Is the interpretation of the results adequate, is it based on the given results and are there any comparisons with similar research/results?


During the review, please pay attention to the adherence to the technical instructions for the submission of the contribution to the journal which can be found on the Journal web page ljsr.pravo.unizg.hr .


REMARK

When reviewing qualitative research please use:

0.	Ajduković, M. (2014). Kako izvještavati o kvalitativnim istraživanjima? Smjernice za istraživače, mentore i recenzente. Ljetopis socijalnog rada, 21 (3), 345-366.

0.	Tong, A., Sainsbury, P. & Craig, J. (2007). Consolidated criteria for reporting qualitative research (COREQ). International Journal for Quality in Health Care, 19 (6), 349-357.


6

LJETOPIS

SOCIJALNOG RADA
