# Supplementary material for: Peer Review in Law Journals
Source: Front Res Metr Anal. 2021 Dec 8;6:787768. doi: 10.3389/frma.2021.787768 (PMC8692876; doi:10.3389/frma.2021.787768)
Supplement: Supplementary file 3 [file DataSheet2.ZIP › DOCUMENT - 1847-7615.RTF]

Instructions to authors

Croatian Academy of Legal Sciences Yearbook (hereinafter: CALS Yearbook or Yearbook) primarily publishes unpublished papers regarding the field of legal sciences, and secondly interdisciplinary papers (which are connected to the field of legal sciences). Authors reserve copyrights over the papers published in the Yearbook, but they give Yearbook the right of first publication. Papers which are accepted for publication or papers already published in the Yearbook the author may publish in other publications only with the permission of the editorial board, but, even in that case, only with clear indication of their publication in the CALS Yearbook. 

Papers should be submitted to the editorial board in electronic form (CD or e-mail:   godisnjak-apzh@pravst.hr). The editorial board accepts only unpublished manuscripts. Along with the title of the paper, it is necessary to specify name, surname and title of the author, name and address of the institution or home address, and e-mail address.  Paper which is submitted for publication in the CALS Yearbook has to be adjusted in style with instructions to authors. Recommended size of the paper is typically up to one and a half author's sheets. Papers are to be written in Croatian or in one of the world languages. They should include an abstract (up to 300 words) and key words (up to five). 

All papers are subject to anonymous reviewing procedure (double blind peer-review) with two reviewers, and for the sake of anonymization of the manuscript, it is not acceptable for the author to write a paper in a way that it is possible to find out his/her identity (for example, from the way he/she refers to his/her own papers) or to indicate his/her name in the text. In addition to aforementioned, the editorial board reserves the right to editorially adjust the paper to the requirements of the journal and to edit papers written in Croatian language (according to the standards of Standard Croatian language). 

Before submitting the paper please: 
0)	check whether the text is in compliance with “technical instructions to authors”, as specified further on in these instructions;
0)	submit written statement (deliver to the editorial board of the Yearbook  and on CD or to e-mail: 	godisnjak-apzh@pravo.hr) according to “requirements for text submission”.


Requirements for text submission 
As the only or the first author of the paper which I apply for publication in Croatian Academy of Legal Sciences Yearbook (hereinafter: CALS Yearbook) I state that: 
?	the proposed paper is entirely the result of my own research work or the work of the research team which I represent as the true author of this text; 
?	I (in case I am the first author of the paper), informed all other authors about the procedure and ethical requirements for publishing our paper in the CALS Yearbook;
?	the work and reasoning of other papers used in the text, are clearly indicated and marked in the text, or as sources in footnotes, as well as in the reference list; 
?	the text is not published or sent for publishing in this form or in the form that uses the majority of this text (more than 50%) in some other journal, book or other publication. 


BIBLIOGRAPHY /REFERENCE LIST  (font: 12)

0.	Surname, initial of the name; Surname, initial of the name (year). Title of the book or paper in the journal. Name of the journal volume of the journal: issue, no. of pg. from-to or place of book publication.  (For on-line materials the following addition is indicated:) Available at: http://www.... (date of last access (not the words, only the date) -09.11.2011).
0.	Example of book: Derenčinović, D.; Getoš, A.-M. (2008). Introduction to Criminology with Basics of Criminal Law. Zagreb.
0.	Example of paper in journal: Kregar, J. (1997). Emergence of corruption. Croatian annual of criminal law and practice 4:1, 23-43.
0.	Example of on-line article: Keen, E. (2000). Fighting Corruption through Education. Budapest. Available at: http://www.hrea.org/pubs/keen2000.pdf (09.11.2011). (font 10)
