# Supplementary material for: Peer Review in Law Journals
Source: Front Res Metr Anal. 2021 Dec 8;6:787768. doi: 10.3389/frma.2021.787768 (PMC8692876; doi:10.3389/frma.2021.787768)
Supplement: Supplementary file 3 [file DataSheet2.ZIP › DOCUMENT - 1847-7615_1.RTF]

Instructions to reviewers 

Reviewers have to fill out the review form (“Reviewer's form”) and give their explanation of the reviewed paper and proposed categorization, as well as state their personal information in the form “Information about reviewer” (forms are located further in the document). 

Reviewers also have to check whether the submitted paper is in compliance with “Instructions to authors” as well as “Technical instructions to authors”, and, if not, remark on that include in their review. 

Reasons for exemption of a reviewer 
In case there is one of the reasons for a conflict of interest, for example, if the manuscript is a part of an academic paper (seminar paper, diploma thesis, master’s thesis, doctoral thesis etc.) which was made under the reviewer 's mentorship, if the reviewer knows the author of the paper, or the reviewer is related to the author, as well as any other reason which would be a reason for exemption in the administrative procedure, please inform the editorial board of the journal, or a reviewer can independently state their decision to be exempted from reviewing the submitted text.  

INFORMATION ABOUT REVIEWER


Name and surname		

OIB

		
Value Added taxpayer (encircle)	yes   -   no	

Address
		

Name of the bank and giro account number

		


CROATIAN ACADEMY OF LEGAL SCIENCES YEARBOOK

REVIEWER'S FORM


0.	Reviewer:

 
0.	Title of the paper:


0.	Proposal on classification of the reviewed paper (specify only one classification mark – on the back or on a separate sheet attach the explanation of the review): 

Original scientific paper 
Scientific review
Preliminary communication
Professional paper


0.	Recommendation for publishing (specify only one recommendation) : 


Accept without alterations
Accept with minor alterations (annotate in the manuscript)
Accept after proposed major alterations
Reject
