# Supplementary material for: Peer Review in Law Journals
Source: Front Res Metr Anal. 2021 Dec 8;6:787768. doi: 10.3389/frma.2021.787768 (PMC8692876; doi:10.3389/frma.2021.787768)
Supplement: Supplementary file 3 [file DataSheet2.ZIP › DOCUMENT - 1848-0357.RTF]

THE REVIEW PROCESS AND INSTRUCTIONS FOR REVIEWERS 
 

Papers undergoing categorisation in the journal Croatian and Comparative Public Administration are submitted to two double-blind reviews. At least one review needs to be written by a reviewer from outside Croatia.
Reviewers are renowned international and Croatian scholars and university professors. The reviewer must have at least a PhD in the relevant field. In case the paper deals with a country-specific topic, a reviewer from that country is chosen.
The paper is sent to the reviewers without any data that could indicate the author(s) of the paper. In addition, the reviewers receive a review form. 
The reviewers are under obligation to protect the anonymity of the review process and not to disclose any information of the authors that might come to their knowledge.
The reviewers shall give their assessment of the paper based on their professional knowledge and respecting the rules of professional ethics. The reviewers shall examine in particular the topic of the paper, its scholarly and practical contribution, the methodology implemented, and the results obtained. The reviewers shall also give their assessment of the technical part of the paper, especially regarding the accuracy of citations, the reference list, and the use of the appropriate bibliography. The reviewers are under obligation to report any suspicion of the existence of plagiarism. 
Once the review is written, the reviewers shall recommend the appropriate categorisation of the paper from the following:
?	original scientific paper – the paper contains original research conducted by the author and/or the author’s original conclusions, as well as represents a contribution to a specific area of science;
?	review scientific paper – the paper offers a synthesis and a review of the situation in a particular area or of a particular topic, it contains an appropriate review of the scientific literature, but it does not contain the results of original research conducted by the author or original ideas;
?	preliminary scientific report – the paper contains initial results of the research conducted by the author, but it does not make an elaborate contribution to a specific area of science;
Apart from categorisation, the reviewers shall give one of the following recommendations:
?	Publish without any changes
?	Publish with changes stated in the review
?	Reject the paper

The reviewers’ comments are delivered to the author without the reviewers’ names (anonymously). In case the reviewers recommend any changes, the author is expected to amend and/or update the paper respecting the instructions of the reviewers.
The Editorial Board, based on the papers themselves and upon the recommendations of the Editor-in-Chief, the Assistant Editors, and the Executive Editor, shall make the final decision on the categorisation of the papers. The meetings of the Editorial Board are held four times a year, prior to the publication of every issue. If the first review recommends that the paper be categorised as a professional paper, and the Editor-in-Chief, the Assistant Editors, and the Executive Editor agree with the proposed categorisation, the paper shall not be reviewed further. In the case of conflicting reviews, the paper shall undergo a third and, if necessary, a fourth review.
The reviewers are expected to deliver their reviews by e-mail in two weeks’ time. 
