# Supplementary material for: Peer Review in Law Journals
Source: Front Res Metr Anal. 2021 Dec 8;6:787768. doi: 10.3389/frma.2021.787768 (PMC8692876; doi:10.3389/frma.2021.787768)
Supplement: Supplementary file 3 [file DataSheet2.ZIP › DOCUMENT - 1848-6118.RTF]

      	
 	
Početna stranica 
Abecedni popis časopisa  

Časopisi po područjima 
o	Prirodne znanostio	 

§	Prirodne znanosti (all)§	 
§	Matematika§	 
§	Fizika§	 
§	Geologija§	 
§	Kemija§	 
§	Biologija§	 
§	Geofizika§	 
§	Interdisciplinarne prirodne znanosti§	 

o	Tehničke znanostio	 

§	Tehničke znanosti (all)§	 
§	Arhitektura i urbanizam§	 
§	Brodogradnja§	 
§	Elektrotehnika§	 
§	Geodezija§	 
§	Građevinarstvo§	 
§	Grafička tehnologija§	 
§	Kemijsko inženjerstvo§	 
§	Metalurgija§	 
§	Računarstvo§	 
§	Rudarstvo, nafta i geološko inženjerstvo§	 
§	Strojarstvo§	 
§	Tehnologija prometa i transport§	 
§	Tekstilna tehnologija§	 
§	Zrakoplovstvo, raketna i svemirska tehnika§	 
§	Temeljne tehničke znanosti§	 
§	Interdisciplinarne tehničke znanosti§	 

o	Biomedicina i zdravstvoo	 

§	Biomedicina i zdravstvo (all)§	 
§	Temeljne medicinske znanosti§	 
§	Kliničke medicinske znanosti§	 
§	Javno zdravstvo i zdravstvena zaštita§	 
§	Veterinarska medicina§	 
§	Dentalna medicina§	 
§	Farmacija§	 

o	Biotehničke znanostio	 

§	Biotehničke znanosti (all)§	 
§	Poljoprivreda (agronomija)§	 
§	Šumarstvo§	 
§	Drvna tehnologija§	 
§	Biotehnologija§	 
§	Prehrambena tehnologija§	 
§	Nutricionizam§	 
§	Interdisciplinarne biotehničke znanosti§	 

o	Društvene znanostio	 

§	Društvene znanosti (all)§	 
§	Ekonomija§	 
§	Pravo§	 
§	Politologija§	 
§	Informacijske i komunikacijske znanosti§	 
§	Sociologija§	 
§	Psihologija§	 
§	Pedagogija§	 
§	Edukacijsko-rehabilitacijske znanosti§	 
§	Logopedija§	 
§	Kineziologija§	 
§	Demografija§	 
§	Socijalne djelatnosti§	 
§	Sigurnosne i obrambene znanosti§	 
§	Interdisciplinarne društvene znanosti§	 

o	Humanističke znanostio	 

§	Humanističke znanosti (all)§	 
§	Filozofija§	 
§	Teologija§	 
§	Filologija§	 
§	Povijest§	 
§	Povijest umjetnosti§	 
§	Znanost o umjetnosti§	 
§	Arheologija§	 
§	Etnologija i antropologija§	 
§	Religijske znanosti (interdisciplinarno polje)§	 
§	Interdisciplinarne humanističke znanosti§	 

o	Umjetničko područjeo	 

§	Umjetničko područje (all)§	 
§	Kazališna umjetnost (scenske i medijske umjetnosti)§	 
§	Filmska umjetnost (filmske, elektroničke i medijske umjetnosti pokretnih slika)§	 
§	Glazbena umjetnost§	 
§	Likovne umjetnosti§	 
§	Primijenjena umjetnost§	 
§	Plesna umjetnost i umjetnost pokreta§	 
§	Dizajn§	 
§	Književnost§	 
§	Interdisciplinarno umjetničko polje§	 

o	Interdisciplinarna područja znanostio	 

§	Interdisciplinarna područja znanosti (all)§	 
§	Kognitivna znanost (prirodne, tehničke, biomedicina i zdravstvo, društvene i humanističke znanosti)§	 
§	Geografija§	 
§	Integrativna bioetika (prirodne, tehničke, biomedicina i zdravstvo, biotehničke, društvene, humanističke znanosti)§	 
§	Kroatologija§	 
§	Obrazovne znanosti (psihologija odgoja i obrazovanja, sociologija obrazovanja, politologija obrazovanja, ekonomika obrazovanja, antropologija obrazovanja, neuroznanost i rano učenje, pedagoške discipline)§	 
§	Rodni studiji§	 
§	Biotehnologija u biomedicini (prirodno područje, biomedicina i zdravstvo, biotehničko područje)§	 
§	Projektni menadžment§	 

o	Interdisciplinarna područja umjetnostio	 

§	Interdisciplinarna područja umjetnosti (all)§	 


Uredništva 
Posjećenost časopisa 
Izjava o otvorenom pristupu 
Statusi časopisa 
Kriteriji uvrštavanja časopisa 
Prijava novog časopisa 

Autori 
Prijava radova 
ORCID identifikator 

Politike i razmjena 
Politike korištenja 
Interoperabilnost 


 
 
 
 
 
 


	Zagrebačka pravna revija
   


  


Status u Hrčku:aktivan ISSN 1848-6118 (Online)  Kontakt:Email:Url:http://revija.pravo.unizg.hrIzdavač:Pravni fakultet Sveučilišta u Zagrebu Trg Republike Hrvatske 14, Zagreb http://zbornik.pravo.hr

Recenzija: vanjske recenzije, pretežito tuzemna, dvostruka, samo znanstveni i stručni radovi, dvostruko slijepa Prva godina izlaženja: 2012
Učestalost izlaženja (godišnje): 3

Područja pokrivanja: Društvene znanosti; Pravo; Uključen u Hrčak: 22. 10. 2014.
Arhiva


2020   Vol. 9  No. 2     Vol. 9  No. 1   2019   Vol. 8  No. 3     Vol. 8  No. 2     Vol. 8  No. 1   2018   Vol. 7  No. 3     Vol. 7  No. 2     Vol. 7  No. 1   2017   Vol. 6  No. 3     Vol. 6  No. 2     Vol. 6  No. 1   2016   Vol. 5  No. 3     Vol. 5  No. 2     Vol. 5  No. 1   2015   Vol. 4  No. 3     Vol. 4  No. 2     Vol. 4  No. 1   2014   Vol. 3  No. 3     Vol. 3  No. 2     Vol. 3  No. 1   2013   Vol. 2  No. 2     Vol. 2  No. 1   2012   Vol. 1  No. 1   Posjeta: 169.466 *  	     
Kontakt 

Pretraživanje članaka


Napredno pretraživanje
Upute za pretraživanje


Moj profil
Registracija novih korisnika
Promjena načina autorizacije	
Izjava o pristupačnosti  Politika privatnosti  Kontakt 
Srce 	
