# Supplementary material for: Peer Review in Law Journals
Source: Front Res Metr Anal. 2021 Dec 8;6:787768. doi: 10.3389/frma.2021.787768 (PMC8692876; doi:10.3389/frma.2021.787768)
Supplement: Supplementary file 3 [file DataSheet2.ZIP › DOCUMENT - 1849-241X.RTF]

11. 07. 2021.	FIP - Financije i pravo

FIP - Financije i pravo


Status u Hrčku: aktivan

ISSN 1849-241X (Tisak)

ISSN 1849-4803 (Online)

Kontakt:	dr.sc.Vedran Ceranić

vceranic@effectus.com.hr

Email:	vceranic@effectus.com.hr

Izdavač:	EFFECTUS poduzetnički studiji - visoko učilište

Trg J.F.Kennedy 2, 10000 Zagreb

https://effectus.com.hr/visoko/

referada@effectus.com.hr Upute za autore (127 KB)


Recenzija: vanjske recenzije, podjednako tuzemna i inozemna, dvostruka, samo znanstveni i stručni radovi, dvostruko slijepa

Prva godina izlaženja: 2013

Učestalost izlaženja (godišnje): 2

Područja pokrivanja: Društvene znanosti; Ekonomija; Pravo;

Uključen u Hrčak: 13. 1. 2015.


https://hrcak.srce.hr/financije-i-pravo	1/1
