# Supplementary material for: Peer Review in Law Journals
Source: Front Res Metr Anal. 2021 Dec 8;6:787768. doi: 10.3389/frma.2021.787768 (PMC8692876; doi:10.3389/frma.2021.787768)
Supplement: Supplementary file 3 [file DataSheet2.ZIP › DOCUMENT - 1849-3734.RTF]

Journal for International and European Law, Economics and Market Integrations

PAPER REVIEW FORM


Zagreb, 2014.


I. Introduction


The Journal editorial board is interested in publishing wide range of papers from the field of European and international law, cross border business, international trade and economy and market integrations.

All the papers submitted for publishing must be written in compliance with the Journals Instructions for the authors and are subject to double-blinded review. Only part IV. - The Review of this form will be presented to the author thus not revealing the reviewers identity. The papers shall be classified as (1) original scientific paper, (2) preliminary communication, (3) review article, or (4) professional paper.


II. Guidelines for the reviewers


In reviewing please pay attention to the following elements:

0()	It is important to consider and conclude if the paper makes a valuable contribution to existing knowledge and literature in terms of theory development, new data, new methodology etc.

0()	Is the paper written and submitted according to Journals Instructions for the authors.

0()	Is the paper written and presented up to publishable standards of the Journal in terms of: whether any concepts have been defined adequately, whether the paper is well structured with coherent argumentation and well integrated, clarity of any statistical data, tables and diagrams, whether the title reflects the contents of the paper accurately, if any part of the paper should be cut out, restructured or further developed.

0()	Appropriateness of referencing in terms of giving adequate credit to other contributors in the field, major omissions and whether the references are complete and written correctly.

0()	Reviewers proposal to the course of action may be to: accept the paper as presented, repeat the review process after changes recommended by reviewer, reject the paper.


`.	Classification Guidelines


0()	An original scientific paper presents the results of original research that were not previously published in either full or preliminary form.

0()	A preliminary communication contains at least one new scientific fact or result that requires immediate publication, but it should not contain enough details to test the described results, as is the case with original scientific articles.

0()	A review article contains an overall review of recent and current research in a specific area. Papers in this category are surveys in nature that should contain critical references and evaluations. The references must be complete enough to permit a good insight into the subject-matter.

0()	A professional paper does not have to be based on original research, but it should contribute to the application of well-known research results and present theoretical conceptions.


IV. Reviewers personal data


NAME:				
TITLE:				
ORGANIZATION:				
ADDRESS:				
				
PHONE:		E-MAIL:		
Hereby  I  state  that  submitted  review  is  done	SIGNATURE	
objectively and indifferently and is solely based			
on professional, scientific and ethical standards.			

V. The Review


A. GENERAL DATA ON PAPER

Title:___________________________________________________________________________
________________________________________________________________________________
________________________________________________________________________________

No. Of Pages:_______________________ No. Of References:______________________________


B. GENERAL EVALUATION OF THE PAPER

		Poor	Below	Average	Good	Excellent		
			Average					
								
	Theoretical / Conceptual							
	Framework							
								
	Statement Of The Problem							
								
	Significance Of Research							
	Literature Review							
								
	Methodology							
	Quality Of Data Or Findings							
								
	Results And Conclusion							
	Readability And Writing Style							


C. ORIGINALITY OF THE PAPER AND ITS CONTRIBUTION TO THE FIELD

	None	Trivial	Modest	Important	Very significant	
						
Contribution to the field						
						


Other titles covering the same issues:

________________________________________________________________________________

________________________________________________________________________________

________________________________________________________________________________

________________________________________________________________________________

________________________________________________________________________________

________________________________________________________________________________

________________________________________________________________________________

________________________________________________________________________________

D. OVERALL REVIEWERS COMMENTS AND IMPRESIONS

________________________________________________________________________________

________________________________________________________________________________

________________________________________________________________________________

________________________________________________________________________________

________________________________________________________________________________

________________________________________________________________________________

________________________________________________________________________________

________________________________________________________________________________

________________________________________________________________________________

________________________________________________________________________________

________________________________________________________________________________

________________________________________________________________________________


E. OVERALL EVALUATION

	Reject	Accept with major Revision	Accept with minor revision	Accept	
		and further review	without further review	unconditionally	
					
Evaluation					
					


Reviewers recommendations on improvements and revision (if any):

________________________________________________________________________________

________________________________________________________________________________

________________________________________________________________________________

________________________________________________________________________________

________________________________________________________________________________

________________________________________________________________________________

________________________________________________________________________________

________________________________________________________________________________


F. CLASIFICATION OF THE PAPER

If the paper is accepted unconditionally or is accepted with minor revision without further review under the term that author has revised the paper according to the Reviewers recommendations on improvements and revision (which is evaluated by the chief editor) the paper should be classified as:

Original scientific paper		
Preliminary communication		
Review article		
Professional paper		
