# Supplementary material for: Peer Review in Law Journals
Source: Front Res Metr Anal. 2021 Dec 8;6:787768. doi: 10.3389/frma.2021.787768 (PMC8692876; doi:10.3389/frma.2021.787768)
Supplement: Supplementary file 3 [file DataSheet2.ZIP › DOCUMENT - 1970-5476.RTF]

About the Journal
Focus and scope
The Theory and Critics of Social Regulation (TCrs) journal aims to develop a research on philosophy of law and in general on critical theory in the social sciences and philosophy. Hereafter are the most important research topics of the journal: hermeneutics, epistemology and legal aesthetics, “law and literature”, rhetoric and legal arguments, “law and humanities” and “critical studies”, bioethics and new technologies.
The research program is based on the necessity to critically analyze the social bond institutive processes. The hypothesis is that the study of these processes requires the elaboration of a general theory of law and institutions that should be able to surmount the nationalistic paradigm, on the basis of critical remarks on current problems, such as: symbolic forms and social bond; European identity; post-national legitimacy; forms of governance and procedural turn; crisis of regulation processes and new models of subjective identity.
 
Peer review
The review has a double blind referee. The criteria by which referees are selected depends on referee’s thematic competence and the paper topics. Specific referee criteria can be found within Director Bruno Montanari’s paper within the 2013 number. 
 
Publication Frequency
Theory and Critics of Social Regulation (TCrs) is biannual.
Authors have to submit articles for the Essays (Miscellaneous), and Notes sections to the journal at any time. They will be published once the referee’s process is over, in the provisional graphic form sent by the author in the ‘Latest articles’ section, and then inserted in the Essays and Notes sections in the Issue following their publication.
 
Privacy
The names and email addresses entered in this journal site will be used exclusively for the stated purposes of this journal and will not be made available for any other purpose or to any other party.
 
