# Supplementary material for: Peer Review in Law Journals
Source: Front Res Metr Anal. 2021 Dec 8;6:787768. doi: 10.3389/frma.2021.787768 (PMC8692876; doi:10.3389/frma.2021.787768)
Supplement: Supplementary file 3 [file DataSheet2.ZIP › DOCUMENT - 1971-8543.RTF]

Valutazione
Valutazione
I parametri di valutazione delle pubblicazioni scientifiche e della ricerca rendono necessaria la pratica della “revisione dei pari” o “referaggio” per conservare la classificazione in fascia “A”. Per ragioni di trasparenza si riportano di seguito le modalità attuative.
Tipologia - È stata prescelta la via del referaggio anonimo e doppiamente cieco: l'Autore non conosce chi sarà il Valutatore e questi non conosce chi sia l’Autore. L’Autore invierà il contributo alla Redazione in due versioni, la prima identificabile, destinata alla pubblicazione, e la seconda, destinata al Valutatore, anonima, ossia priva di qualsivoglia riferimento - nel testo, nelle note e anche nei metadati - che possa fare risalire in via diretta o anche indiretta all’Autore.
Consenso - L'Autore,  con l'invio di un articolo alla Redazione, esprime il consenso a sottoporre il testo al referaggio di un esperto del settore scientifico disciplinare, o di settori affini, scelto dalla Direzione in appositi elenchi.
Regole ed eccezioni - Tutti i contributi inviati dagli Autori sono sottoposti alla valutazione dei pari, e porteranno in calce alla prima pagina l'annotazione "Contributo sottoposto a valutazione".
Il Direttore, o il Comitato scientifico a maggioranza, può decidere di non sottoporre a valutazione i contributi, d'interesse per gli studiosi del settore scientifico-disciplinare Jus 11: a) di Autori (stranieri e italiani) di riconosciuto prestigio accademico, che ricoprano cariche di rilievo politico-istituzionale in organismi nazionali, comunitari e internazionali anche confessionali (a pie' di pagina l'annotazione "Contributo non sottoposto a valutazione"); b) di docenti collocati fuori ruolo (a pie' di pagina l'annotazione "Contributo non sottoposto a valutazione"); c) già editi in altre riviste o in lavori collettanei, di cui si chieda la pubblicazione con il permesso dell’Autore e dell’editore della pubblicazione (a pie' di pagina l'annotazione "Contributo pubblicato per la cortesia dell'Autore e dell'Editore"); d) le relazioni, le presentazioni e gli interventi a Congressi, Convegni, Tavole rotonde organizzate dall’ADEC, o da altre associazioni di docenti, o comunque di rilevanza nazionale o internazionale, per i quali non sia di fatto possibile rispettare la regola dell'anonimato dell’Autore (a pie' di pagina l'annotazione "Contributo non sottoposto a valutazione. Relazione tenuta al Congresso ...").
Nella Sezione "A chiare lettere", gli editoriali e i contributi destinati alle sotto-rubriche "Confronti" e "Transizioni", non sono soggetti a valutazione.
Il Direttore, sentito se del caso il Comitato di direzione, potrà rifiutare la pubblicazione di contributi palesemente compilativi e/o privi dei necessari requisiti di scientificità, originalità, pertinenza senza sottoporli alla  valutazione previa.
Pertinenza - La Direzione, prima ancora di procedere alla valutazione del contributo,  ne accerterà la pertinenza all’ambito del settore scientifico-disciplinare Jus 11 (studi relativi: alla disciplina giuridica del fenomeno religioso, anche nella prospettiva comparatistica, sia all’interno dell’ordinamento statuale, sia negli ordinamenti confessionali; alla storia del diritto canonico; alla storia e ai sistemi dei rapporti tra gli Stati e le Chiese; al diritto comparato delle religioni; ai profili di rilevanza giuridica dei fenomeni di pluralismo etico e religioso). In caso di pertinenza del contributo ad altri settori scientifici, la Direzione valuterà se il tema trattato possa comunque essere d'interesse per gli studiosi del settore scientifico-disciplinare Jus 11; il contributo potrà essere sottoposto alla valutazione di esperti di altre discipline scelti di volta in volta.
Criteri - La valutazione dei pari non potrà essere influenzata dalle convinzioni personali, dagli indirizzi teorici o dalle appartenenze di scuola dell’Autore, e verterà soltanto sui seguenti cinque parametri: 1) originalità dell’impianto della ricerca e dei risultati conseguiti; 2) conoscenza critica della dottrina e della giurisprudenza relative al tema trattato; 3) appropriatezza delle basi metodologiche; 4) coerenza interna formale (tra titolo, sommario, e abstract) e sostanziale (rispetto alla posizione teorica dell’Autore); 5) chiarezza e correttezza formale dell'esposizione.
Doveri e compiti del Valutatore – L’esperto cui sarà affidata la valutazione di uno scritto: - osserverà scrupolosamente i criteri sopra indicati; - rinuncerà al compito affidatogli se avrà riconosciuto l'Autore dello scritto; - tratterà il testo da valutare come confidenziale fino a che non sia pubblicato, e distruggerà tutte le copie elettroniche e a stampa degli articoli ancora in bozza e le sue stesse relazioni una volta ricevuta la conferma dalla Redazione che la relazione è stata ricevuta; - terrà in debito conto l’anzianità d’impegno nella ricerca e la qualifica accademica dell’Autore, che conoscerà in forma anonima (dottorando, dottore di ricerca, assegnista, ricercatore, associato, ordinario, magistrato, ecc.); - non rivelerà ad altri quali scritti ha giudicato, e non diffonderà tali scritti neanche in parte; - assegnerà per ognuno dei cinque parametri prefissati un punteggio variabile dal minimo di 1 al massimo di 5, utilizzando un’apposita scheda da restituire compilata al Direttore, a uso esclusivo e riservato del medesimo; - formulerà un sintetico giudizio, in forma libera, in ordine a originalità, accuratezza metodologica, e forma dello scritto, giudicando con obiettività, prudenza e rispetto.
Esiti - Gli esiti della valutazione dello scritto possono essere: a) non pubblicabile; b) non pubblicabile se non rivisto, indicando motivatamente in cosa; c) pubblicabile dopo qualche modifica/integrazione, da specificare nel dettaglio; d) pubblicabile (salvo eventualmente il lavoro di editing per il rispetto dei criteri redazionali). Tranne che in quest’ultimo caso, l’esito è comunicato all’Autore a cura della Direzione, nel rispetto dell’anonimato del Valutatore. Un giudizio favorevole alla pubblicazione presuppone che la qualità complessiva del contributo risulti “buona” (vale a dire un punteggio complessivo non inferiore a 15, senza nessun “uno” e con non più di due “due”).
Revisione dei testi - Qualora il Valutatore incaricato di esaminare un articolo evidenzi la necessità/opportunità di modifiche o integrazioni dello scritto, l’Autore ne sarà puntualmente informato, nel rispetto dell’anonimato del Valutatore. L’Autore, quindi, evidenzierà nel testo le variazioni apportate sulla base delle indicazioni ricevute, utilizzando l’evidenziatore o un diverso colore dei caratteri, in modo da agevolare il Valutatore nel confronto della nuova versione con la precedente.
Riservatezza - I Valutatori e i componenti della Direzione, del Comitato scientifico e della Redazione s'impegnano al rispetto scrupoloso della riservatezza sul contenuto della scheda e del giudizio espresso, da osservare anche dopo l’eventuale pubblicazione dello scritto.
Valutatori – I Valutatori sono individuati tra gli studiosi - fuori ruolo e in ruolo, italiani e stranieri, esperti del settore scientifico-disciplinare Jus 11 o che hanno dato a esso specifici contributi , pur appartenendo ad altri settori, -  disponibili a esaminare in tempi brevi (di regola entro due settimane) il contributo sottoposto alla loro valutazione, e che accettino espressamente i criteri e le modalità previste per l’espletamento del loro compito. Nella home page di questa Rivista è riportato il loro elenco; al termine di ogni anno sarà riportato anche l’elenco di quanti tra essi sono stati richiesti di assolvere il compito della valutazione.
Vincolatività - Sulla scorta dei dati della scheda e del giudizio sintetico del Valutatore il Direttore decide se pubblicare lo scritto, se chiederne all'Autore la revisione o se respingerlo. In casi eccezionali, la valutazione di non pubblicabilità potrà non essere vincolante, sempre che il Direttore e almeno due componenti del Comitato scientifico non la ritengano adeguatamente motivata e decidano, pertanto, di sottoporre il contributo all'esame di un diverso Valutatore, il cui giudizio sarà in ogni caso vincolante.  
