# Supplementary material for: Peer Review in Law Journals
Source: Front Res Metr Anal. 2021 Dec 8;6:787768. doi: 10.3389/frma.2021.787768 (PMC8692876; doi:10.3389/frma.2021.787768)
Supplement: Supplementary file 3 [file DataSheet2.ZIP › DOCUMENT - 1973-3593.RTF]

NOTA PER GLI AUTORI

La Rivista di Diritto Alimentare è aperta a giuristi, economisti, imprenditori, funzionari pubblici, policy makers e giornalisti, interessati a condividerne l'impostazione e le finalità.

Sollecitiamo tutti coloro che sono interessati ad inviare, per posta elettronica, i loro lavori a:

Rivista di Diritto Alimentare Redazione

Monica Minelli

Via Ciro Menotti, 4 00195 ROMA
e-mail: redazione@aida-ifla.it

L'invio non comporta il pagamento di alcuna fee. I lavori inviati vengono sottoposti al giudizio di due referees anonimi scelti tra gli esperti dei temi trattati. Qualora siano giudicati positivamente dai referees, i lavori verranno pubblicati nei numeri successivi della rivista. In ogni caso gli articoli ed i commenti pubblicati impegnano esclusivamente la responsabilità degli autori.

I lavori devono essere in formato doc di WORD (interlinea singola, Arial 12 per il testo ed Arial 9 per le note).

Gli "Interventi" devono essere di dimensione contenuta possibilmente entro i 20 mila caratteri (note e spazi inclusi) mentre i "Commenti" devono essere di dimensione compresa tra i 7 mila e 15 mila caratteri (note e spazi inclusi). Devono riportare il titolo del contributo, il nome dell'autore (o autori), l'affiliazione, ed essere suddivisi in paragrafi titolati (orientativamente: un sottotitolo ogni 4-5 mila caratteri). I titoli possono essere ritoccati per esigenze editoriali in fase di chiusura della rivista.

Le note devono essere di dimensioni contenute e vanno limitate allo stretto indispensabile, privilegiando i riferimenti normativi e giurisprudenziali.
La bibliografia deve essere quella essenziale.
E' utile che si indichino i siti internet di riferimento per approfondimenti o i links ai documenti citati (questi possono essere inseriti in calce al documento).

I riferimenti bibliografici diversi dai siti internet vanno inseriti come negli esempi qui di seguito forniti:

-	G. Sgarbanti, Commento all'art. 4, in La sicurezza alimentare nell'Unione europea (commento articolo per articolo al reg. 178/2003), a cura dell'IDAIC, in
NLCC, 2003, 188.
-	A. Jannarelli, Il diritto dell'agricoltura nell'era della globalizzazione, 2^ ed., Bari, 2003.

Ciascun lavoro deve essere accompagnato da un breve abstract in lingua inglese, e dall'indicazione di tre parole chiave per la classificazione, e può rinviare a lavori di approfondimento e di maggiore dimensione in corso di pubblicazione su riviste cartacee.

Nel rispetto della pluralità di voci e di opinioni accolte nella Rivista, gli articoli ed i commenti pubblicati impegnano esclusivamente la responsabilità degli autori.
