# Supplementary material for: Peer Review in Law Journals
Source: Front Res Metr Anal. 2021 Dec 8;6:787768. doi: 10.3389/frma.2021.787768 (PMC8692876; doi:10.3389/frma.2021.787768)
Supplement: Supplementary file 3 [file DataSheet2.ZIP › DOCUMENT - 1988-5091_1.RTF]

1


INSTRUCCIONES A LOS AUTORES – INSTRUCTION FOR

CONTRIBUTORS

1. CUESTIONES RELATIVAS A LA PRESENTACIÓN DE LOS ARCHIVOS


Los trabajos deben presentarse en Microsoft Word.


Las contribuciones podrán enviarse por correo electrónico a las siguientes direcciones 1) de IUSTEL (www.iustel.com): revistas@iustel.com;

2) del secretario de la Revista, Matteo Nicolini: matteo.nicolini@univr.it


En caso de trasmisión del trabajo a la Editorial, esta lo remitirá al Secretario (Prof. Dr. D Matteo Nicolini) de la Revista General de Derecho Público Comparado.


Los archivos deben necesariamente nombrarse con los apellidos del autor del trabajo, seguido de un punto y de la abreviatura de la sección correspondiente de la Revista.

Las abreviaturas de cada sección son las siguientes:

`)	edit: Editorial

`)	est.: Estudios

`)	cdoc: Comentarios de doctrina

`)	cleg.: Comentarios de legislación

`)	cjur.: Comentarios de jurisprudencia

`)	cron: Crónicas de actualidad

`)	rec.: Recensiones


Ejemplos:

Mortati.est.doc

Mestre Delgado.cjur.doc


En todos los trabajos, sea cual sea la lengua en la que se escriban, se habrá de indicar:


1)  El título del trabajo, en castellano y en inglés;


Princesa, 29, 2. º 28008 Madrid > T 91548 82 81 > F 915 489 482 > iustel@iustel.com > www.iustel.com

2


0)	Nombre y Apellidos del Autor;

0)	Cargo académico, o actividad que desempeña, así como, en su caso, la Universidad a la que pertenece, o la Institución en la que desempeña su actividad;

0)	Correo electrónico;

0)	El sumario, en el idioma en que está escrito, en castellano y en inglés;

0)	Las palabras clave, en el idioma en que está escrito, en castellano y en inglés;

0)	El resumen/abstract (máximo 10 líneas) en el idioma en que está escrito, en castellano y en inglés;

0)	e-mail de contacto.


-	El título deberá ir centrado, en letra mayúscula y en negrita.

-	El nombre y apellidos del autor irán en letra mayúscula y su cargo en letra minúscula. Ambos en letra redonda, sin negrita y centrado.

-	Cada uno de los epígrafes en los que se divida el trabajo irán centrados. Los primeros epígrafes se presentarán en letra mayúscula, en numeración romana, centrados y en negrita; los primeros subepígrafes se presentarán en letra minúscula, en numeración arábiga, centrados y en negrita; a partir de aquí, los posibles siguientes subepígrafes irán en letra minúscula, en numeración arábiga, centrados y sin negrita en la secuencia: 1.1, 1.2, 1.3: 1.3.1, etc.


Al faltar de uno solo de los datos indicados, el comité de redacción suspenderá el proceso de evaluación y remitirá el artículo al autor para que lo integre.


Princesa, 29, 2. º 28008 Madrid > T 91548 82 81 > F 915 489 482 > iustel@iustel.com > www.iustel.com

3


2. CUESTIONES RELATIVAS A LA EDICIÓN DE LOS TRABAJOS


Podrán remitirse a la Revista General, para su publicación, todos aquellos artículos/ensayos/recensiones/comentarios relacionados con la materia y método propios de ella: Derecho comparado; Derecho comparado general; Derecho público comparado; Derecho privado comparado; Cuestiones metodológicas; Cuestiones conectadas al aprendizaje y a la enseñanza del Derecho comparado; Estudios interdisciplinarios de Derecho comparado (Derecho y Literatura, Derecho y Lengua, Derecho y Geografía, Derecho y Religión etc.).

Los trabajos podrán estar escritos en castellano, inglés, francés, italiano, alemán o portugués.

NORMAS DE REDACCIÓN


FORMATO ARCHIVO:

Layout:

–   Superior 2,5 cm;

–   Inferior 2,5 cm;

–   Derecha 2,5 cm;

–   Izquierda 2,5 cm.


EXTENSIÓN DE LOS TRABAJOS (de forma aproximada):

-	Estudios: 20-35 páginas

-	Comentarios de doctrina, legislación o jurisprudencia: 10-15 páginas

-	Crónicas de actualidad: 3-5 páginas


SUMARIO, PALABRAS CLAVE Y UN BREVE RESUMEN redactado en el idioma del artículo, en castellano y en inglés.

Resumen: máximo 10 líneas resumen redactado en el idioma del artículo, en castellano y en inglés.

Palabras clave (no más de 5), en el idioma original, en castellano y en inglés.


TEXTO: ARIAL 10

Párrafo:

Alineado: JUSTIFICADO


Princesa, 29, 2. º 28008 Madrid > T 91548 82 81 > F 915 489 482 > iustel@iustel.com > www.iustel.com

4


Sangría: O CM Espacio: O PTOS

Especial: 1º LINEA – 0, 5 (salvo los títulos de los epígrafes que no llevan) Interlineado: 1.5 (salvo abstract/resumen, palabras clave y sumario que llevan

sencilla)


NOTAS A PIE:

no se incluirá ningún epígrafe de bibliografía, sino que esta quedará reflejada en las notas a pie

TEXTO: ARIAL 9

Párrafo:

Alineado: JUSTIFICADO

Sangría: O CM

Espacio: O PTOS antes; 6 PTOS después

Especial: 1º LINEA – 0, 5

Interlineado: sencillo


EPÍGRAFES:

MAYUSCULA Y NEGRITA: I. … – II. … – III. – IV. … Subepígrafes:

minúscula y negrita: I.1. … – I.2. … – I.3. …


Sub-subepígrafes:

minúscula, sin negrita: I.1.1. … – I.1.2.


Ejemplo:

I.	EPÍGRAFE

I.1.	Subepígrafe

I.1.1. Subsubepigrafe Citas:

Las transcripciones de parte de textos, sentencias, etc. deberán ir entre "…."; la cita dentro de la cita entre '…'.

Si en el texto del artículo la citación directa sobresalga tres líneas irá separada del texto y con sangría izquierda de 1 cm


Princesa, 29, 2. º 28008 Madrid > T 91548 82 81 > F 915 489 482 > iustel@iustel.com > www.iustel.com

5


Ejemplo:


... And then we read:


But in my experience, it's the other way around. I don't know of any absolute acquittals, but I do know of many times when a judge has been influenced […]. I must admit it, I have never witnessed a single case of true absolution […] So. Not a single acquittal," said K., as if talking to himself and his hopes. "That confirms the impression I already have of the court. So, there's no point in it from this side either. They could replace the whole court with a single hangman. […] The courts don't make their final conclusions public, not even the judges are allowed to know about them, so that all we know about these earlier cases are just legends. (Kafka, "The Trial", cit., p. 126).

While the atmosphere of "The Trial" is punitive, and therefore ….


Las citas de los trabajos deberán ir en notas a pie de página y no en notas al final.

Las referencias bibliográficas, legislativas o jurisprudenciales contendrán todos los datos necesarios para su adecuada localización, y se ajustarán a los estándares de citación en publicaciones jurídicas españolas o, en su caso, de los países a que correspondan las normas o sentencias citadas. Cuando se haga referencia a sitios de Internet, habrá que indicar expresamente, entre paréntesis, la fecha última en que fueron visitados.

Artículos

-	M. Iglesias Berlanga, C. Quesada Alcalá, "La autodeterminación y el reconocimiento de Estados, Crónica de hechos internacionales", Revista Electrónica de Estudios Internacionales, Vol. (o nº 16), n. 2, 2008, pp. 6-8.

Citaciones sucesivas:

-	M. Iglesias Berlanga, C. Quesada Alcalá, "La autodeterminación", cit., pp. 6-8.


Libros:

-	E. Álvarez Conde, Derecho Constitucional, Tecnos, Madrid, 2013, p. 23. Citaciones sucesivas:

-	E. Álvarez Conde, Derecho Constitucional, cit., p. 23.


Capítulos De Libro:


Princesa, 29, 2. º 28008 Madrid > T 91548 82 81 > F 915 489 482 > iustel@iustel.com > www.iustel.com

6


-	Moya Luckett, "Image and Nation in 1990s British Cinema," in British Cinema of the 90s, ed. Robert Murphy (London: British Film Institute, 2000), p. 98.

Citaciones sucesivas:

-	Moya Luckett, "Image and Nation", cit., p. 98.


No se utilizen ibid. or op. cit.


Páginas web:

Citación entera

-	http://nwscholasticpress.org/2012/09/30/follow-these-simple-techniques-to-write-the-perfect-caption-every-time-to-intrigue-inform-readers-2/ (acceso 12.11. 2015).

-	Yansong Feng and Mirella Lapata, "How Many Words Is A Picture Worth? Automatic Caption Generation for News Images," in Proceedings of the 48th Annual Meeting of the Association for Computational Linguistics, eds. T. Huang et al. (Uppsala: ACL, 2010), 1241 publicado en http://www.aclweb.org/anthology/P10-1126 (acceso 10.03.2016).

Legislación:

La primera citación será de acuerdo con los criterios aplicados por el ordenamiento en que se ha adoptado la ley, el reglamento, etc.

Las citaciones sucesivas podrán realizarse según los criterios siguientes:

-	Mediante acrónimo, en los ordenamientos en que éste se utiliza, como en España (LOTC, EA, etc.);

-	Indicando número y año: ley no. 2 de 2017;

-	Utilizando el así dicho Short Title: Scotland Act, etc.


Jurisprudencia:

La primera citación será de acuerdo con los criterios aplicados por el ordenamiento en que se ha adoptado la ley, el reglamento, etc.; en alternativa:

Nombre del Tribunal, fecha, número, año, partes.


Los Autores que quieren proponer un artículo para la evaluación podrán enviar el artículo así como lo han escrito, y adecuar el mismo a los estándares de la Revista al haber sido el artículo evaluado positivamente.


A falta de cumplimiento de cualquiera de estos criterios, el artículo será devuelto al autor/a la autora para que realice las actualizaciones editoriales necesarias.


Princesa, 29, 2. º 28008 Madrid > T 91548 82 81 > F 915 489 482 > iustel@iustel.com > www.iustel.com

7


3. CUESTIONES RELATIVAS A LA PUBLICACIÓN DE LOS TRABAJOS


En relación con los derechos de autor, los autores pueden utilizar sus derechos para publicar sus trabajos en cualquier otra publicación, siempre en soporte papel (y no en soporte electrónico), con el único requisito de reconocer la previa aparición en la Revista General correspondiente, incluyendo el nombre y el dominio en la red de la Revista (http://www.iustel.com).


El Consejo rector de la Revista General de Derecho Público Comparado exigirá que los trabajos sean originales, si bien siempre cabrán excepciones:

-	por la importancia o actualidad del tema;

-	si el artículo estará publicado en revista y libros de difusión territorialmente limitadas;

-	si el artículo estará publicado en un idioma diferente del utilizado por publicar en la Revista;

en estos casos, los equipos consideran de interés publicar un trabajo en la Revista General una vez difundido por otra publicación periódica de papel.


Se autoriza a PORTALDERECHO S.A. a la publicación en formato papel de los trabajos remitidos por los autores.


Princesa, 29, 2. º 28008 Madrid > T 91548 82 81 > F 915 489 482 > iustel@iustel.com > www.iustel.com

8


4. EVALUACIÓN


Recibidos los originales, el Comité editorial de la Revista General de Derecho Público Comparado realizará acuse de recibo al autor/a la autora.


El Comité evaluará si los artículos/ensayos/recensiones/comentarios enviados sean adecuados y coherentes con la materia y método propios de ella, esto es: Derecho comparado; Derecho comparado general; Derecho público comparado; Derecho privado comparado; Cuestiones metodológicas; Cuestiones conectadas al aprendizaje y a la enseñanza del Derecho comparado; Estudios interdisciplinarios de Derecho comparado (Derecho y Literatura, Derecho y Lengua, Derecho y Geografía, etc.).

Los artículos/ensayos/recensiones/comentarios de Derecho doméstico relativos a síngulos Países (por ejemplo, un ensayo sobre Colombia escrito por un peruano o por un español sobre Francia) podrán ser publicados en la medida en que conste la relevancia del artículo para la comparación jurídica. Dicha relevancia deberá ser indicada en el artículo.


En caso de que el texto no se estime adecuado al contenido de la Revista, será devuelto al autor/a la autora.


Si, por el contrario, los artículos/ensayos/recensiones/comentarios se estimen adecuados a los criterios editoriales serán enviados anonimizados por el Secretario de la Revista a dos evaluadores, que sean miembros del listado de evaluadores, según la materia sobre la que verse el referido trabajo para que emitan la nota de calificación que les merece.


La Dirección de la Revista según la materia sobre la calificación que les merece.


podrá designar también evaluadores externos al listado , que verse el referido trabajo para que emitan la nota de

El Secretario notificará al autor el resultado de la nota de calificación que puede ser:

-	Totalmente favorable.

-	Favorable, con indicaciones de modificación.

-	Desfavorable.


Princesa, 29, 2. º 28008 Madrid > T 91548 82 81 > F 915 489 482 > iustel@iustel.com > www.iustel.com

9


Si uno de los informes fuese favorable y otro desfavorable, el Comité podrá remitir el trabajo a una tercera persona para que realice la calificación definitiva.


Los Consejos rectores de las Revistas Generales de http://www.iustel.com constituyen equipos independientes de valoración.


Princesa, 29, 2. º 28008 Madrid > T 91548 82 81 > F 915 489 482 > iustel@iustel.com > www.iustel.com
