# Supplementary material for: Peer Review in Law Journals
Source: Front Res Metr Anal. 2021 Dec 8;6:787768. doi: 10.3389/frma.2021.787768 (PMC8692876; doi:10.3389/frma.2021.787768)
Supplement: Supplementary file 3 [file DataSheet2.ZIP › DOCUMENT - 1989-4570_1.RTF]

Editorial Policies
`.	Focus and Scope
`.	Section Policies
`.	Peer Review Process
`.	Plagiarism control
`.	Ethics and malpractice statement
`.	Abstracting and Indexing
Focus and Scope
Cuadernos de Derecho Internacional is a journal which publishes research papers on private international law, uniform law and comparative private law biannually (March and October).
CDT is edited by  Department Private International Law –Universidad Carlos III
 
Section Policies
Descarga CDT
 Open Submissions	 Indexed	 Peer Reviewed	
Presentación
 Open Submissions	 Indexed	 Peer Reviewed	
Estudios
 Open Submissions	 Indexed	 Peer Reviewed	
Varia
 Open Submissions	 Indexed	 Peer Reviewed	
Congresos
 Open Submissions	 Indexed	 Peer Reviewed	
Recensiones
 Open Submissions	 Indexed	 Peer Reviewed	
 
Peer Review Process
Peer evaluation process. The publication of the papers will be decided by the Direction of the Journal, with the participation of the Editorial Board and being advised by the Scientific Committee, based on an external, independent and anonymous evaluation (peer 2 peer review). The paper may be "accepted", "accepted with modifications" or "rejected". Depending on the nature of the paper, it will be included in the "Studies" section or in the "Varia" section.
 
Plagiarism control
Cuadernos de Derecho Transnacional in order to guarantee the academic integrity of the publication uses  <<Feedback Studio>> , an application that allows reviewing documents by detecting incorrect citations or plagiarism.
 
Ethics and malpractice statement
1. General duties of the editorial team that make up CDT
i) The Direction and the editorial management of CDT as well as the Advisory Committee, the Scientific Committee and the Evaluation Committee undertake to be up to date with all the contents published in the journal.
ii) Strive to meet the needs of readers and authors.
iii) To commit to the constant improvement of the review.
iv) To ensure the quality of the published contents.
v) To put science and ethical commitments before economic benefits
vi) To retract, clarify, correct or apologize in relation to the content of the publications when necessary
2. Ethical commitments of the CDT editorial team with readers
i) Inform readers about who founded the review, who are the directors and are part of its editorial team.
ii) Ensure that all contents have been reviewed by experts in the field.
iii) Publish some clear, simple and concise rules for authors so that anyone can know the steps to follow to publish in the review.
iv) Commit to promoting scientific references in the works, maintaining clear guidelines for authors in this regard.
v) Show clarity regarding the ownership of published works.
3. Ethical commitments of the CDT editorial team with the authors
i) That the reasons that lead to accept, accept with modifications or reject a paper for publication only rely on scientific reasons based on the quality and originality of it.
ii) Except for serious errors in relation to the evaluation process, decisions about whether a job is accepted or denied will not be modified.
iii) The evaluation process followed to accept a work will be published on the review's website.
iv) The publication of  clear, simple and concise rules  for authors so that anyone can know the steps to follow to publish in the review.
4. Commitments of the CDT editorial team with the reviewers
i) The commitment to provide the reviewers with clear instructions on the evaluation process and on the obligations they have with the journal in relation to their review task.
ii) The commitment to require reviewers to manifest any personal interest that may cause problems of neutrality in the review process.
iii) The commitment to maintain the anonymity of the review.
 
Abstracting and Indexing
- ÍnDICEs-CSIC
- HeinOnline
- ERIH Plus
- MIAR 2018 (Matriz de Información para el Análisis de Revistas).
- Catálogo LATINDEX.
- Dialnet (Universidad de La Rioja)
- Category B –Law  CARHUS Plus+ 2018
