# Supplementary material for: Peer Review in Law Journals
Source: Front Res Metr Anal. 2021 Dec 8;6:787768. doi: 10.3389/frma.2021.787768 (PMC8692876; doi:10.3389/frma.2021.787768)
Supplement: Supplementary file 3 [file DataSheet2.ZIP › DOCUMENT - 1989-8975_1.RTF]

Reviewer guidelines
COMPETING INTEREST
REALA uses the "double blind" peer review evaluation system, so that neither authors nor reviewers know the identity of the other party.
In the event that the author is identified, although their names have been removed from the manuscript, the reviewers must declare any conflict of interest and reject the publisher's invitation to evaluate a manuscript.
A conflict of interest may occur as a result of:
`.	Animosity towards the authors;
`.	Academic or family closeness:
`.	They belong to the same university, department, research group, professional network, research project, etc.
`.	Have published articles with the author.
`.	any other type of connection or conflict / professional proximity.
On the other hand, if you suspect that an article is a substantial copy of another work, the evaluator must inform the editors. Also, publishers will be informed if there is a suspicion that the results of the article are false or fraudulent.
REVIEW GUIDELINES
Reviewers will consider following criteria to accept or reject manuscript evaluation:
They will have knowledge and experience in the topic discussed. They should only perform their function if they consider themselves competent in the matter they are proposed to evaluate.
They will have availability. They undertake to respect the time limits and to follow the journal’s guidelines in their work. In case of acceptance, they must comply with the deadlines established for review end (4 weeks from the receipt of the manuscript). In case of refusal, they will communicate to Secretary as soon as possible.
They will assume a commitment of confidentiality so that they may not, throughout the process, to disclose the content of the article to a third party. Consultations with another expert should be previously consulted with the editor. The only authorized interlocutors for any issue related to the evaluation process are the director and editors of the journal.
Indications for review
Once the review has been accepted, the reviewers shall carry out an objective, technical and constructive review of the article or book review.The task of the reviewers in the evaluation process includes:
0.	Check that the article meets with the guidelines of the journal’s review policy.
0.	Fill out the peer review form created by the journal’s editor, adding comments to the author for the manuscript review: necessary suggestions, improvements or reforms to enable the article to be published.
0.	Add confidential comments to editors: any other consideration in relation to the quality and acceptability of the manuscript, or any comment that you do not wish to pass on to the author.
The review will be carried out by fill in the article’s evaluation form/report and concludes with its submission through the journal’s website to the editors by issuing the corresponding recommendation. This form addresses the following aspects:
Formal aspects
The reviewers will make sure that the documents:
?	Respect the maximum extent of the article.
?	Include title (spa/en), biographical note, summary (spa/en), keywords (spa/en) and table of contents.
?	Respect the meaning of the IMRyD structure.
?	Incorporate the figures and tables into their position in the document when they exist.
?	Incorporate the footnotes.
?	Includes bibliographic references, complete and updated.
?	They correctly use the citation system proposed by the journal, a variation of the ASA (American Sociological Association) system, listed in the journal’s guidelines, and include the DOI identifier of those references that have it, as well as the hyperlinks to the external directions.
Aspects related to content
The reviewers will evaluate the content of the article according to the aspects related in the article’s evaluation form, that can be seen in detail on this link and includes:
PRESENTATION, CLARITY AND READABILITY
`.	Is the approach to the content of the work clear?
`.	Is the presentation structure adequate?
`.	Are the ideas well organized?
`.	Is the wording adequate?
RELEVANCE AND SCIENTIFIC INTEREST
?	Is the work current?
?	Is it of interest to the scientific community?
?	Does it present innovative elements?
THEORETICAL CONTRIBUTION
?	Does it present relevant elements from a theoretical point of view?
?	Are the well founded?
?	Are elements of the state of the discipline reviewed?
?	Is it a purely descriptive work?
?	In this last case (purely descriptive work), does the subject justify it?
METHODOLOGICAL QUALITY
0.	Are the method used and the treatment given to the subject adequate?
SOURCES
0.	Is the bibliography used by the author adequate and updated?
0.	Is the jurisprudence used by the autor adequate and updated?
0.	What other sources could the author have used?
TITLE, ABSTRACT AND KEYWORDS
?	They are appropriate?
?	How could they be reformulated?
COMMENTS FOR THE  MANUSCRIPT REVIEW
Indicate all those suggestions, improvements or reforms necessary for the article to be published.
CONFIDENTIAL COMMENTS
Indicate to the editors any other consideration regarding the quality and acceptability of the manuscript, or any comments that you do not wish to be communicated to the author.
EVALUATOR'S DECISION
Once the article has been evaluated, the reviewer must choose an option from the Peer review Form:
?	PUBLISHABLE: It must be published as is or with the small modifications indicated in the report.
?	PUBLICABLE AFTER YOUR REVIEW. It can be published in the report.
?	NOT PUBLISHABLE. The manuscript should not be published.
The reviewer must also select a recommendation from the proposals in the “Recommendation” section.
?	Accept this paper.
?	Publishable with modifications.
?	Foward for review.
?	Forward to another publication.
?	Not publishable.
?	See comments.
