# Supplementary material for: Peer Review in Law Journals
Source: Front Res Metr Anal. 2021 Dec 8;6:787768. doi: 10.3389/frma.2021.787768 (PMC8692876; doi:10.3389/frma.2021.787768)
Supplement: Supplementary file 3 [file DataSheet2.ZIP › DOCUMENT - 1989-8975_2.RTF]

About the Journal
Focus and Scope
Peer Review Process
All received articles are subjected to an initial review to rule out those which are not suitable in terms of the areas addressed by REALA.
The journal’s review policy is based in the double-blind evaluation method, so that neither authors nor reviewers know the identity of the other party.
The maximum time for review an article is six months.
Reviewers are chosen from recognized professionals in the field addressed by the articles, coming from academia and the private sector, with both national and international profiles.
1. REALA publishes academic works of rigorous theoretical and empirical research, with multidisciplinary nature, particularly in those social sciences that contribute to understanding the subject matter of the journal: law, administrative sciences, political science, public management, sociology and economy.
2. The format and layout errors, the non compliance with the journal’s rules or the incorrect spelling and syntax may be cause for rejection of the manuscript without being evaluated.
3. The review will be by peer-reviewed by the «double-blind» evaluation method. All the works sent to REALA will be reviewed according to strict criteria of scientific quality.
4. Once received a text that complies with all the formal requirements, the author(s) will receive a confirmation of receipt and the evluation process will start.
5. In a first stage, the Editorial Board of REALA will revise the overall quality and the thematic adjustment of the work. By doing so, those manuscripts which quality is notoriously low or not implementing any contribution to the journal’s thematic areas may be directly rejected without undergoing an external review. For this first review, the Editorial Board may require the assessment by members of the Advisory Board, if deemed necessary.
6. The articles which pass this first filter will be sent to two external reviewers, who will be specialist in the field or in line of research concerned, linking the article review form. When the assessments are discrepant or for any other reason deemed necessary, the Editorial Board could send the text to a third reviewer.
7. In view of the reports of the reviewers, the Editorial Board may adopt one of the following decisions  that will be communicated to the author(s):
`.	Publishable as it is (or with minor revisions).
`.	Publishable after revision. In this case, publication will depend on the realization by the author(s) of all the changes required by the editors. The deadline for these modifications is one month, and the author(s) will attach a cover letter explaining the changes and how they satisfy the requirements of the Editorial Board. As one of these changes, the journal may propose the conversion of a text submitted as an article into a research note/bibliographical note, or vice versa.
`.	Not publishable.
8. If a text is accepted for publication, the galleys should be reviewed by the author(s) within Three days.
9. REALA will publish annually the list of all persons who have made anonymous evaluations.
10. REALA may make public, once they are verified, the following bad scientific practices: plagiarism, data falsification or fabrication, misapppropriation of authorship and double publication.
