# Supplementary material for: Peer Review in Law Journals
Source: Front Res Metr Anal. 2021 Dec 8;6:787768. doi: 10.3389/frma.2021.787768 (PMC8692876; doi:10.3389/frma.2021.787768)
Supplement: Supplementary file 3 [file DataSheet2.ZIP › DOCUMENT - 2038-5633.RTF]

_________OSSERVATORIO SULLE FONTI__________


Regolamento per la valutazione scientifica dei contributi proposti alla Rivista


1. Proposta di pubblicazione dei contributi

La proposta di pubblicazione sulla rivista Osservatorio sulle fonti (di seguito la "Rivista") è trasmessa alla Redazione (indirizzo di posta elettronica reda-zione@osservatoriosullefonti.it) da uno dei membri della Direzione, ovvero direttamente dall'Autore.

La proposta deve contenere:

`)	L'indicazione della qualifica accademica o professionale e dei recapiti dell'Autore;

`)	La Sezione della Rivista nella quale si richiede la pubblicazione (Saggi, Note e commenti, Speciali);

`)	Un abstract nella lingua inglese;

`)	Un titolo del contributo in formato ridotto (massimo 50 caratteri);

`)	La dichiarazione che il contributo è esclusiva opera dell'Autore e che lo stesso non abbia già pubblicato lo scritto in altra sede. Nel caso in cui lo scritto sia destinato alla pubblicazione in volume tale destinazione, se già conosciuta, dev'essere indicata in nota. L'Au-tore è altresì tenuto a dichiarare se il contributo abbia ricevuto una valutazione negativa da parte di altra rivista.

Il contributo ricevuto è trasmesso tempestivamente alla Direzione che ef-fettua una prima valutazione editoriale in ordine alla generale adeguatezza tematica del contributo proposto, con particolare riferimento all'imposta-zione e ai temi trattati nella Rivista.


2. Formazione dell'elenco dei valutatori

La Direzione approva un elenco di valutatori esterni agli Organi della Rivi-sta, composto da studiosi italiani e stranieri particolarmente qualificati nel settore dello studio delle fonti del diritto, la cui inclusione nell'elenco può essere proposta da ciascun componente della Direzione.


© Osservatoriosullefonti.it


2


L'elenco dei valutatori è custodito e aggiornato dalla Direzione.


3. Valutazione dei contributi

La Direzione garantisce l'anonimato dell'intera procedura di valutazione, secondo lo schema della single blind peer review.

Qualora una proposta di pubblicazione sia preliminarmente accettata, la Direzione invia il contributo fatto pervenire dall'Autore ad un valutatore tratto dall'elenco di cui all'articolo 2.

Nella individuazione dei valutatori per ciascun contributo, la Direzione se-leziona coloro che presentano una maggiore competenza sul tema del con-tributo, rispettando un criterio di rotazione.

Il contributo è trasmesso ai valutatori in forma pienamente anonimizzata, senza l'indicazione del nominativo dell'Autore, e omettendo eventuali rife-rimenti anche bibliografici che ne possano determinare la riconoscibilità. L'Autore non viene in alcun modo messo a conoscenza del nominativo del valutatore cui il contributo è stato trasmesso.

La Direzione trasmette al valutatore la relativa scheda su cui esprimere il giudizio e indica allo stesso un termine entro il quale esprimersi. Nel caso in cui il giudizio non sia espresso nei tempi assegnati la Direzione può pro-cedere alla sostituzione dal valutatore.

L'Autore riceve il rapporto del valutatore mediante la trasmissione della scheda contenente il parere reso, con eventuali suggerimenti per la revisione.

Nel caso in cui fosse richiesto il riesame di decisioni inerenti alla valuta-zione, la decisione spetta alla Direzione.

La Redazione facilita i rapporti tra il Direttore e gli Autori, svolge attività di sostegno tecnico nella procedura di valutazione (tra cui la tenuta dell'ar-chivio contenente le schede di valutazione) ed è preposta alla pubblicazione dei contributi, nel pieno rispetto dei criteri editorali, nonché alla fascicola-zione dei numeri.


4. Giudizio dei valutatori

I valutatori, una volta ricevuto il contributo, sono tenuti a esprimere un


© Osservatoriosullefonti.it


3


parere in ordine alla pubblicazione sulla Rivista attraverso l'utilizzo della scheda di valutazione.

Il giudizio ha carattere discorsivo e sintetico ed è formulato con riferimento ai seguenti parametri:

`)	la pertinenza del contributo ai temi trattati dalla Rivista;

`)	la novità/originalità del contributo;

`)	la metodologia seguita dall'autore;

`)	la chiarezza e lo stile dell'esposizione.

Il giudizio può contenere anche indicazioni confidenziali per la Direzione, che non vengono trasmesse all'autore.

Il giudizio può concludersi con:

`)	indicazione di dignità di pubblicazione;

`)	raccomandazione di non procedere alla pubblicazione;

`)	richieste di revisione del contributo cui è subordinato il giudizio di di-gnità di pubblicazione

Nel caso sub c), la Direzione inoltra immediatamente all'Autore, sempre in forma anonima, le richieste di revisione pervenute, invitandolo a adeguar-visi. La Direzione verifica l'adeguamento del contributo alle richieste di revisione, eventualmente inviandolo di nuovo – se necessario e soprattutto in relazione alla quantità e alla ampiezza delle revisioni richieste – al valutatore che le ha formulate.


5.- Contributi esentati dalla peer review

Nel caso di contributi di Autori di particolare autorevolezza la Direzione può decidere di non ricorrere al procedimento di valutazione previsto dagli articoli precedenti. Tale circostanza, debitamente motivata, è indicata in una nota riportata nella prima pagina del contributo, conformemente a quanto stabilito dalla regolamentazione adottata dall'ANVUR.

I contributi appartenenti alla sezione Rubriche o diversamente ospitati dalla Rivista non sono sottoposti alla procedura di valutazione e sono col-locati al di fuori del relativo fascicolo (cd. "fuori numero"). La medesima eccezione vale, ad esempio, per contributi quali gli interventi a forum e/o discussioni scientifiche, gli editoriali o legati ad argomenti di particolare di attualità.


© Osservatoriosullefonti.it


4


La scelta circa la pubblicazione di tali contributi è rimessa alla Direzione della Rivista.

7. Tempi di pubblicazione dei contributi sottoposti a valutazione

In considerazione della rigorosa procedura di valutazione indicata, che se-gue le indicazioni richieste per le riviste di fascia A, la pubblicazione dei contributi sottoposti a valutazione richiede un tempo complessivo stimabile in non meno di un mese dall'invio del contributo da parte dell'Autore.

Sul sito della Rivista sono pubblicati i termini di scadenza per la consegna dei contributi, elaborati sulla base di tempistiche che consentano di rispet-tare la periodicità quadrimestrale della stessa.


8. Entrata in vigore e applicazione

Tale regolamento entra in vigore a partire dal 1° gennaio 2020 e si applica a partire dai contributi destinati al numero 1/2020 della Rivista.


© Osservatoriosullefonti.it
