# Supplementary material for: Peer Review in Law Journals
Source: Front Res Metr Anal. 2021 Dec 8;6:787768. doi: 10.3389/frma.2021.787768 (PMC8692876; doi:10.3389/frma.2021.787768)
Supplement: Supplementary file 3 [file DataSheet2.ZIP › DOCUMENT - 2217-561X.RTF]

      	
 	
Početna stranica 
Abecedni popis časopisa  

Časopisi po područjima 
o	Prirodne znanostio	 

§	Prirodne znanosti (all)§	 
§	Matematika§	 
§	Fizika§	 
§	Geologija§	 
§	Kemija§	 
§	Biologija§	 
§	Geofizika§	 
§	Interdisciplinarne prirodne znanosti§	 

o	Tehničke znanostio	 

§	Tehničke znanosti (all)§	 
§	Arhitektura i urbanizam§	 
§	Brodogradnja§	 
§	Elektrotehnika§	 
§	Geodezija§	 
§	Građevinarstvo§	 
§	Grafička tehnologija§	 
§	Kemijsko inženjerstvo§	 
§	Metalurgija§	 
§	Računarstvo§	 
§	Rudarstvo, nafta i geološko inženjerstvo§	 
§	Strojarstvo§	 
§	Tehnologija prometa i transport§	 
§	Tekstilna tehnologija§	 
§	Zrakoplovstvo, raketna i svemirska tehnika§	 
§	Temeljne tehničke znanosti§	 
§	Interdisciplinarne tehničke znanosti§	 

o	Biomedicina i zdravstvoo	 

§	Biomedicina i zdravstvo (all)§	 
§	Temeljne medicinske znanosti§	 
§	Kliničke medicinske znanosti§	 
§	Javno zdravstvo i zdravstvena zaštita§	 
§	Veterinarska medicina§	 
§	Dentalna medicina§	 
§	Farmacija§	 

o	Biotehničke znanostio	 

§	Biotehničke znanosti (all)§	 
§	Poljoprivreda (agronomija)§	 
§	Šumarstvo§	 
§	Drvna tehnologija§	 
§	Biotehnologija§	 
§	Prehrambena tehnologija§	 
§	Nutricionizam§	 
§	Interdisciplinarne biotehničke znanosti§	 

o	Društvene znanostio	 

§	Društvene znanosti (all)§	 
§	Ekonomija§	 
§	Pravo§	 
§	Politologija§	 
§	Informacijske i komunikacijske znanosti§	 
§	Sociologija§	 
§	Psihologija§	 
§	Pedagogija§	 
§	Edukacijsko-rehabilitacijske znanosti§	 
§	Logopedija§	 
§	Kineziologija§	 
§	Demografija§	 
§	Socijalne djelatnosti§	 
§	Sigurnosne i obrambene znanosti§	 
§	Interdisciplinarne društvene znanosti§	 

o	Humanističke znanostio	 

§	Humanističke znanosti (all)§	 
§	Filozofija§	 
§	Teologija§	 
§	Filologija§	 
§	Povijest§	 
§	Povijest umjetnosti§	 
§	Znanost o umjetnosti§	 
§	Arheologija§	 
§	Etnologija i antropologija§	 
§	Religijske znanosti (interdisciplinarno polje)§	 
§	Interdisciplinarne humanističke znanosti§	 

o	Umjetničko područjeo	 

§	Umjetničko područje (all)§	 
§	Kazališna umjetnost (scenske i medijske umjetnosti)§	 
§	Filmska umjetnost (filmske, elektroničke i medijske umjetnosti pokretnih slika)§	 
§	Glazbena umjetnost§	 
§	Likovne umjetnosti§	 
§	Primijenjena umjetnost§	 
§	Plesna umjetnost i umjetnost pokreta§	 
§	Dizajn§	 
§	Književnost§	 
§	Interdisciplinarno umjetničko polje§	 

o	Interdisciplinarna područja znanostio	 

§	Interdisciplinarna područja znanosti (all)§	 
§	Kognitivna znanost (prirodne, tehničke, biomedicina i zdravstvo, društvene i humanističke znanosti)§	 
§	Geografija§	 
§	Integrativna bioetika (prirodne, tehničke, biomedicina i zdravstvo, biotehničke, društvene, humanističke znanosti)§	 
§	Kroatologija§	 
§	Obrazovne znanosti (psihologija odgoja i obrazovanja, sociologija obrazovanja, politologija obrazovanja, ekonomika obrazovanja, antropologija obrazovanja, neuroznanost i rano učenje, pedagoške discipline)§	 
§	Rodni studiji§	 
§	Biotehnologija u biomedicini (prirodno područje, biomedicina i zdravstvo, biotehničko područje)§	 
§	Projektni menadžment§	 

o	Interdisciplinarna područja umjetnostio	 

§	Interdisciplinarna područja umjetnosti (all)§	 


Uredništva 
Posjećenost časopisa 
Izjava o otvorenom pristupu 
Statusi časopisa 
Kriteriji uvrštavanja časopisa 
Prijava novog časopisa 

Autori 
Prijava radova 
ORCID identifikator 

Politike i razmjena 
Politike korištenja 
Interoperabilnost 


 
 
 
 
 
 


	Političke perspektive : časopis za istraživanje politike
   


  


 Status u Hrčku:aktivan ISSN 2217-561X (Tisak)  ISSN 2335-027X (Online)  UDK:303.1https://doi.org/10.20901/ppKontakt:Fakultet političkih znanosti, Lepušićeva 6, 10000 Zagreb, Hrvatska
Tel.: +385 1 464 2016
Fax.: +385 1 465 5316
e-mail: perspektive@fpzg.hr
Fakultet političkih nauka, Jove Ilića 165, 11040 Beograd, Srbija
Tel.: +381 11 3092999
e-mail: perspektive@fpn.bg.ac.rsUrl:https://www.fpzg.unizg.hr/izdavastvo/casopisi/politicke_perspektiveIzdavač:Fakultet političkih znanosti HR-10000 Zagreb, Lepušićeva 6 http://www.fpzg.unizg.hrUpute za autore (69 KB) Impressum (89 KB) Urednički odbor (283 KB) 
Političke su perspektive regionalni časopis koji su 2011. pokrenuli Fakultet političkih znanosti iz Zagreba, Fakultet političkih nauka iz Beograda i Udruženje za političke nauke Srbije. Časopis nije disciplinarno ograničen mada mu je fokus na političkoj znanosti. Ideja je da se naglasi stručno i akademsko istraživanje politike, tako da su u časopisu dobrodošli i tekstovi bliskih disciplina (prava, sociologije, filozofije, ekonomije itd.) kojima takav stav nije stran.

Političke perspektive izlaze tri puta godišnje i objavljuju recenzirane tekstove na engleskom, hrvatskom i srpskom jeziku. 

Rukopisi se šalju putem mrežne platforme za uređivanje časopisa Open Journal System (OJS) na: 
https://hrcak.srce.hr/ojs/index.php/politicke-perspektive

Sve pristigle rukopise evaluira uredništvo, te ih zatim šalje na dvostruko slijepe recenzije

Časopis Političke perspektive ne naplaćuje autorima obradu ili objavljivanje rada. Časopis nema naknade za obradu članaka (APC), naknade za prijem rukopisa, naknadu za recenziranje ili bilo koje druge naknade potrebne za dovršetak postupka objavljivanja.

Recenzija: vanjske recenzije, podjednako tuzemna i inozemna, dvostruka, samo znanstveni i stručni radovi, dvostruko slijepa Prva godina izlaženja: 2011
Učestalost izlaženja (godišnje): 3

Područja pokrivanja: Ekonomija; Pravo; Politologija; Sociologija; Filozofija; Uključen u Hrčak: 25. 8. 2015.

Prava korištenja: Političke perspektive su časopis s otvorenim pristupom. Sva izdanja časopisa Političke perspektive dostupna su u otvorenom pristupu, a sav sadržaj časopisa mogu besplatno koristiti pojedinci ili institucionalni korisnici. Svi korisnici smiju čitati, spremati, fotokopirati, tiskati, distribuirati, pretraživati ​​i dijeliti dijelove ili cjelovite tekstove članaka, recenzija, recenzija knjiga i drugog sadržaja bez traženja pristanka ili dopuštenja od strane autora ili izdavača sve dok na odgovarajući način citiraju izvor. 


Arhiva


2021   Vol. 11  No. 1   2020   Vol. 10  No. 2-3     Vol. 10  No. 1   2019   Vol. 9  No. 3     Vol. 9  No. 2     Vol. 9  No. 1   2018   Vol. 8  No. 3     Vol. 8  No. 1-2   2017   Vol. 7  No. 3     Vol. 7  No. 1-2   2016   Vol. 6  No. 3     Vol. 6  No. 1-2   2015   Vol. 5  No. 3     Vol. 5  No. 2     Vol. 5  No. 1   2014   Vol. 4  No. 3     Vol. 4  No. 2     Vol. 4  No. 1   2013   Vol. 3  No. 3     Vol. 3  No. 2     Vol. 3  No. 1   2012   Vol. 2  No. 3     Vol. 2  No. 2     Vol. 2  No. 1   2011   Vol. 1  No. 3     Vol. 1  No. 2     Vol. 1  No. 1   Posjeta: 126.354 *  	     
Kontakt 

Pretraživanje članaka


Napredno pretraživanje
Upute za pretraživanje


Moj profil
Registracija novih korisnika
Promjena načina autorizacije	
Izjava o pristupačnosti  Politika privatnosti  Kontakt 
Srce 	
