# Supplementary material for: Peer Review in Law Journals
Source: Front Res Metr Anal. 2021 Dec 8;6:787768. doi: 10.3389/frma.2021.787768 (PMC8692876; doi:10.3389/frma.2021.787768)
Supplement: Supplementary file 3 [file DataSheet2.ZIP › DOCUMENT - 2239-8570.RTF]

CODICE ETICO
0.	Doveri della Rivista
1.1. Il Direttore della Rivista, il Comitato di Direzione e i membri del Comitato di Redazione sono i soli responsabili della decisione di pubblicare gli articoli sottoposti alla Rivista stessa. Nelle loro decisioni, essi sono tenuti a rispettare le strategie e l’impostazione editoriale della Rivista. Essi sono inoltre vincolati dalle disposizioni di legge vigenti in materia di diffamazione, violazione del copyright e plagio.
1.2. Nell’assumere le proprie decisioni, la Direzione si avvale del supporto del Comitato di Direzione e del Comitato di Redazione.
1.3. I membri del Comitato di Direzione Comitato di Redazione e del Comitato dei Revisori sono tenuti a valutare i manoscritti per il loro contenuto scientifico, senza distinzione di razza, sesso, orientamento sessuale, credo religioso, origine etnica, cittadinanza, di orientamento scientifico, accademico o politico degli autori.
1.4. Il Direttore della Rivista, il Comitato di Direzione e i membri del Comitato di Redazione sono tenuti a non divulgare alcuna informazione sui manoscritti inviati a nessun altro soggetto diverso dagli autori, revisori e potenziali revisori.
1.5. Il materiale inedito contenuto nei manoscritti sottoposti alla Rivista non può essere usato dal Direttore della Rivista, dal Comitato di Direzione e dai membri del Comitato di Redazione per proprie ricerche senza il consenso dell’autore.
1.6. Se il Direttore della Rivista, il Comitato di Direzione o i membri del Comitato di Redazione rileva o riceve segnalazioni in merito ad errori o imprecisioni, conflitto di interessi o plagio in un articolo pubblicato, ne darà tempestiva comunicazione all’autore e all’editore ed intraprenderà le azioni necessarie per chiarire la questione e, in caso di necessità, ritirerà l’articolo o pubblicherà una ritrattazione.
1.7. La peer review, c.d. revisione paritaria, è finalizzata a garantire il livello qualitativo della Rivista. A tal fine i contributi pervenuti per la pubblicazione nella Rivista vengono trasmessi dalla Direzione a revisori, scelti tra i componenti del Comitato dei revisori, tenendo conto delle competenze specifiche. I revisori operano nel rispetto del codice etico della Rivista e dei doveri ivi indicati.
Il contributo viene inviato al Revisore – il cui nome viene mantenuto riservato – dalla Segreteria di Redazione e senza alcuna indicazione del nome dell’Autore.
Il Revisore trasmette alla Segreteria di redazione, nel rispetto dei tempi indicati, una succinta valutazione, adeguatamente motivata, del lavoro indicando la opportunità della pubblicazione. 
Qualora il giudizio sia di meritevolezza della pubblicazione, il Revisore può indicare, in prospettiva migliorativa, ulteriori interventi. La documentazione relativa alla procedura di revisione svolta per ciascun contributo è conservata dalla Segreteria di redazione della Rivista.
I contributi destinati alle rubriche web sono oggetto di valutazione da parte della Direzione e\o di un membro del Comitato di Redazione e\o del Comitato di Direzione.
 
`.	Doveri dei revisori
2.1. I membri del ‘Comitato di revisori’ assistono il Direttore della Rivista, il Comitato di Direzione e i membri del Comitato di Redazione nelle decisioni editoriali e possono eventualmente indicare all’autore correzioni e accorgimenti atti a migliorare il manoscritto.
2.2. Il revisore selezionato che non si senta qualificato alla revisione del manoscritto assegnato o che sappia di non essere in grado di eseguire la revisione nei tempi richiesti, deve notificare la sua decisione alla Segreteria di redazione e al Direttore, rinunciando a partecipare al processo di revisione.
2.3. I manoscritti ricevuti per la revisione devono essere trattati come documenti riservati. Essi non devono essere mostrati o discussi con chiunque non sia previamente autorizzato dal Direttore.
2.4. La revisione deve essere condotta con obiettività. Non è ammesso criticare o offendere personalmente un autore. I referee devono esprimere le proprie opinioni in modo chiaro e con il supporto di argomentazioni documentate. Ogni dichiarazione, osservazione o argomentazione riportata deve preferibilmente essere accompagnata da una corrispondente citazione.
2.5. Il revisore deve richiamare l’attenzione della Segreteria di redazione e del Comitato di Direzione qualora ravvisi una somiglianza sostanziale o una sovrapposizione tra il manoscritto in esame e qualunque altro documento pubblicato di cui è a conoscenza. Le informazioni o idee ottenute tramite la revisione dei manoscritti devono essere mantenute riservate e non utilizzate per vantaggio personale.
2.6. I revisori non devono accettare manoscritti qualora versino in posizione di conflitto di interessi derivante da rapporti di concorrenza, di collaborazione, o altro tipo di collegamento con gli autori, aziende o enti che abbiano relazione con l’oggetto del manoscritto.
 
`.	Doveri degli autori
3.1 Gli autori devono garantire che le loro opere siano del tutto originali e, qualora siano utilizzati il lavoro e/o le parole di altri autori, che queste siano opportunamente parafrasate o citate letteralmente. In ogni caso, il riferimento al lavoro di altri autori deve essere sempre indicato.
3.2 Gli autori hanno l’obbligo di citare tutte le pubblicazioni che hanno avuto influenza nel determinare la natura del lavoro proposto. Gli autori di articoli basati su ricerca originale devono presentare un resoconto accurato del lavoro svolto, nonché una discussione obiettiva del suo significato. I dati relativi devono essere rappresentati con precisione nel manoscritto.
3.3 I manoscritti devono contenere sufficienti dettagli e riferimenti per permettere ad altri la riproduzione della ricerca svolta. Dichiarazioni fraudolente o volontariamente inesatte costituiscono un comportamento non etico e sono inaccettabili.
3.4 I manoscritti proposti non devono essere stati pubblicati come materiale protetto da copyright in altre riviste. I manoscritti in fase di revisione dalla rivista non devono essere sottoposti ad altre riviste ai fini di pubblicazione. Inviando un manoscritto, l’autore/gli autori concordano sul fatto che, se il manoscritto è accettato per la pubblicazione, tutti i diritti di sfruttamento economico, senza limiti di spazio e con tutte le modalità e le tecnologie attualmente esistenti e/o in futuro sviluppate, saranno trasferiti alla Direzione della Rivista.
3.5 La paternità letteraria del manoscritto è limitata a coloro che hanno dato un contributo significativo per l’ideazione, la progettazione, l’esecuzione o l’interpretazione dello studio. Tutti coloro che hanno dato un contributo significativo devono essere elencati come co-autori. Qualora vi siano altri soggetti che hanno partecipato ad aspetti sostanziali del progetto di ricerca, devono essere riconosciuti ed elencati come contributori nei ringraziamenti. L’autore di riferimento deve garantire che tutti i relativi coautori siano inclusi nel manoscritto, che abbiano visto e approvato la versione definitiva dello stesso e che siano d’accordo sulla presentazione per la pubblicazione.
3.6 Gli autori devono indicare nel manoscritto conflitti finanziari o altre tipologie di conflitto di interessi che possono influenzare i risultati o l’interpretazione del manoscritto. Tutte le fonti di sostegno finanziario al progetto devono essere indicate.
3.7 Qualora un autore riscontri errori significativi o inesattezze nel manoscritto pubblicato ha il dovere di comunicarlo tempestivamente al Comitato di Direzione o all’Editore e cooperare con gli stessi al fine di ritrattare o correggere il manoscritto.
