# Supplementary material for: Peer Review in Law Journals
Source: Front Res Metr Anal. 2021 Dec 8;6:787768. doi: 10.3389/frma.2021.787768 (PMC8692876; doi:10.3389/frma.2021.787768)
Supplement: Supplementary file 3 [file DataSheet2.ZIP › DOCUMENT - 2240-7618.RTF]

Ogni contributo proposto per la pubblicazione è preliminarmente esaminato dalla direzione, che verifica l’attinenza con i temi trattati dalla rivista e il rispetto dei requisiti minimi della pubblicazione.
In caso di esito positivo di questa prima valutazione, la direzione invia il contributo in forma anonima a due revisori, individuati secondo criteri di rotazione tra i membri dell’Editorial Advisory Board in relazione alla rispettiva competenza per materia e alle conoscenze linguistiche. I revisori ricevono una scheda di valutazione, da consegnare compilata alla direzione entro il termine da essa indicato. Nel caso di tardiva o mancata consegna della scheda, la direzione si riserva la facoltà di scegliere un nuovo revisore.
La direzione comunica all’autore l’esito della valutazione, garantendo l’anonimato dei revisori. Se entrambe le valutazioni sono positive, il contributo è pubblicato. Se una o entrambe le valutazioni raccomandano modifiche, il contributo è pubblicato previa revisione dell’autore, in base ai commenti ricevuti, e verifica del loro accoglimento da parte della direzione. Il contributo non è pubblicato se uno o entrambi i revisori esprimono parere negativo alla pubblicazione.
La direzione si riserva la facoltà di pubblicare, in casi eccezionali, contributi non previamente sottoposti alla procedura di peer review. Di ciò è data notizia nella prima pagina del contributo, con indicazione delle ragioni relative.
