# Supplementary material for: Peer Review in Law Journals
Source: Front Res Metr Anal. 2021 Dec 8;6:787768. doi: 10.3389/frma.2021.787768 (PMC8692876; doi:10.3389/frma.2021.787768)
Supplement: Supplementary file 3 [file DataSheet2.ZIP › DOCUMENT - 2240-9823.RTF]

Regolamento per l’invio e la valutazione dei contributi
REGOLAMENTO PER L’INVIO E LA VALUTAZIONE DEI CONTRIBUTI
Il presente regolamento è adottato in conformità dei principi del COPE’s Code of conduct for Journal Editors e disciplina le modalità di redazione, invio e valutazione dei contributi scientifici destinati alla pubblicazione all’interno della rivista Dirittifondamentali.it
ART. 1 SOTTOPOSIZIONE DEI CONTRIBUTI E DATI NECESSARI
La proposta di pubblicazione sulla rivista dirittifondamentali.it avviene attraverso la trasmissione del contributo, redatto secondo i criteri editoriali della rivista, all’indirizzo info@dirittifondamentali.it.
Nella mail è necessario indicare: a) nome e cognome; b) titolo accademico; c) inquadramento universitario; d) istituzione (Università o altra istituzione scientifica) di appartenenza; e) dichiarazione che il contributo sia esclusiva opera dell’Autore e che lo scritto non sia già stato pubblicato o non sia in corso di pubblicazione su altra rivista telematica. In alternativa, se lo scritto è destinato alla pubblicazione in volume o in altra rivista cartacea, indicare in nota tale destinazione; f) abstract in lingua italiana ed in lingua inglese.
ART. 2 VALUTAZIONE PRELIMINARE
Per garantire uno standard elevato della qualità scientifica dei contributi pubblicati, questi ultimi sono sottoposti ad un controllo preliminare da parte del Direttore Scientifico e del Comitato di Direzione. Ciò al fine esclusivo di verificare prima facie l’attinenza del contributo inviato ai profili tematici della Rivista e la sussistenza dei requisiti minimi formali per la pubblicazione.
Le risultanze di tale attività, se negative, sono comunicate all’Autore e ne viene conservata traccia a cura della Segreteria di redazione.
ART. 3 REFERAGGIO
I saggi e gli articoli di ricerca proposti per la pubblicazione che hanno superato la valutazione preliminare sono sottoposti a referaggio cieco svolto da esperti anonimi dell’Accademia, i cui esiti sono formalizzati in apposita scheda che resterà nell’archivio della rivista, a cura della segreteria di redazione.
Viene adottato in via preferenziale lo schema della double blind peer review.
Se uno solo dei valutatori si esprime negativamente rispetto alla pubblicazione, il contributo non è avviato alla pubblicazione.
Se i valutatori si esprimono a favore della pubblicazione, senza suggerire modifiche, il contributo è avviato alla pubblicazione.
Se uno o entrambi i valutatori subordinano la pubblicazione a delle modifiche, i rilievi vengono trasmessi all’Autore il quale, sopo gli adattamenti, potrà nuovamente inviare il contributo alla rivista.
Solo in via residuale e a discrezione dei condirettori può essere utilizzato il sistema di valutazione cd. open peer review: è garantita la forma anonima della valutazione, ma i soggetti chiamati a effettuare la valutazione potranno conoscere l’identità dell’autore del contributo sottoposto a valutazione.
L’esito del referaggio verrà comunicato all’autore. Se positivo, l’Autore disporrà di ulteriori cinque giorni dalla comunicazione per far pervenire alla redazione della rivista la versione definitiva del contributo, corredata del suddetto abstract.
Una volta pubblicato il lavoro non sarà suscettibile di modificazioni.
In ogni caso, il numero massimo di contributi scientifici pubblicati sulla rivista nel corso dell’anno solare non può essere di regola superiore a tre, fatta eccezione per quelli che, stante comunque l’esito positivo del referaggio, risultano prodotti da Studiosi aventi la qualifica di Professori Ordinari di materie giuridiche, di Atenei nazionali od esteri, in servizio o a riposo, nonché per i contributi resi nel corso di eventi scientifici, di cui la Rivista pubblica gli atti.
ART. 4 CONTRIBUTI DI AUTORI PARTICOLARMENTE AUTOREVOLI
A discrezione del Direttore scientifico e del Comitato di direzione, i contributi di Professori universitari Emeriti o di personalità che ricoprono anche cariche accademiche e/o istituzionali, di stretta attinenza con l’attività di ricerca scientifica nel settore giuridico (es.: Presidenti autorità amministrative indipendenti, giudici costituzionali, etc.), possono essere pubblicati senza referaggio. 
Di tale procedura è data indicazione in nota.
ART. 5 CRITERI EDITORIALI 
L’Autore deve predisporre un sommario nel caso in cui il contributo risulti diviso in paragrafi. I titoli dei paragrafi vanno inseriti anche nel corpo del testo.
Le note sono solo a piè di pagina e con numerazione progressiva. Non vanno inseriti ritorni a capo tra una nota e l’altra.
Nelle citazioni bibliografiche gli Autori sono indicati con il nome puntato e il cognome per esteso in “MAIUSCOLO”. 
I volumi sono così indicati: Autore, Titolo in corsivo, casa editrice e/o città, anno di pubblicazione, eventuali pagine. 
Gli articoli tratti da riviste scientifiche sono così indicati: Autore, Titolo articolo in corsivo, in denominazione rivista in corsivo, anno, numero, eventuale parte, eventuale pagina iniziale.
Le riviste on-line dotate di codice ISSN sono citate senza la necessità di indicare l’indirizzo web. Per i contributi editi su riviste on-line non dotate di codice ISSN e per gli altri contributi tratti da siti internet, va riportato l’indirizzo web in corsivo.
Il contributo scientifico non può superare, di regola, il numero massimo di 70.000 battute, spazi inclusi, salvo deroghe specifiche, proposte dal referee, per contributi di particolare pregio scientifico.
ART. 6 TEMPI DI PUBBLICAZIONE
In considerazione dell’esigenza di sottoporre il contributo a referaggio cieco, i termini di pubblicazione possono estendersi, di regola, fino a sessanta giorni.
ART. 7 DISPOSIZIONE FINALE
Per tutto quanto non previsto dal presente regolamento si rinvia al Regolamento dell’ANVUR recante Criteri di classificazione delle riviste ai fini dell’Abilitazione scientifica nazionale e al suo Documento di accompagnamento.
