# Supplementary material for: Peer Review in Law Journals
Source: Front Res Metr Anal. 2021 Dec 8;6:787768. doi: 10.3389/frma.2021.787768 (PMC8692876; doi:10.3389/frma.2021.787768)
Supplement: Supplementary file 3 [file DataSheet2.ZIP › DOCUMENT - 2279-7238.RTF]

NOTE PER GLI AUTORI


DESTINAZIONE EDITORIALE

La Direzione della Rivista Nomos accetta solamente i saggi e le note che per forma e contenuto risultino coerenti con l'impostazione e con le tematiche generalmente trattate nella Rivista.


CRITERI REDAZIONALI

I contributi che pervengono alla redazione di Nomos devono rispettare i criteri di editing della rivista.


LINGUA DI PUBBLICAZIONE

Nomos accetta contributi in italiano, inglese, francese e spagnolo. Ogni contributo, in ogni caso, deve essere corredato da un abstract in lingua inglese, ovvero in italiano (se il contributo è in inglese).


VALUTAZIONE DEI REFEREE

I contributi pervenuti sono sottoposti alla valutazione di due referee.

Per preservare l'integrità del processo di revisione, ai referee non viene rivelato il nome dell'autore, e quest'ultimo non conosce l'identità dei referee.

I saggi vengono pubblicati solo se valutati positivamente. L'esito del referaggio può comportare:

`)	l'accettazione integrale del testo

`)	l'accettazione del testo con proposte migliorative non sostanziali

`)	l'accettazione del testo subordinata a modifiche sostanziali

`)	la scelta di non procedere alla pubblicazione.

I contributi non pubblicati vengono restituiti in ogni caso agli autori.

Le relazioni tenute nell'ambito di Convegni che vengano pubblicate nella sezione Saggi della Rivista possono essere esentate dalla procedura di revisione e ciò verrà in ogni caso adeguatamente segnalato.


SCELTA DEI REFEREE

I referee sono scelti tra i giuspubblicisti italiani e stranieri, su proposta del Comitato scientifico/di redazione.

I criteri che guidano la scelta dei referee cui inoltrare le proposte editoriali sono

`)	la competenza del referee in ordine agli argomenti trattati

`)	la gestione confidenziale del referaggio, con obbligo di distruggere il materiale elettronico al momento della pubblicazione del contributo o della sua definitiva restituzione all'autore
`)	la disponibilità a produrre una relazione circostanziata, in formato elettronico, circa l'originalità del contributo, la metodologia seguita dall'autore, la chiarezza e lo stile dell'esposizione, l'interesse generale per le tematiche affrontate, i meriti e le eventuali migliorie da apportare al testo ai fini della pubblicazione, e a formulare il giudizio in termini anche critici, ma comunque costruttivi.

`)	l'impegno a riferire, e dimostrare, tempestivamente alla Redazione eventuali fenomeni di plagio, in maniera che possano essere presi gli opportuni provvedimenti

`)	l'impegno a rispettare le scadenze convenute per il referaggio, e a fornire comunque un feed-back agli autori non appena possibile.


PROPOSTE DI RECENSIONE

Nomos accetta in ogni momento proposte di letture critiche e recensioni. I volumi che trattano di argomenti coerenti con i temi della rivista per i quali si auspichi una recensione possono essere inviati in ogni momento all'indirizzo:

NOMOS. LE ATTUALITÀ DEL DIRITTO

AL DIRETTORE DELLA BIBLIOTECA DEL DIPARTIMENTO DI SCIENZE POLITICHE E ALLA C.A. DI PAOLA PICIACCHIA
UNIVERSITÀ DEGLI STUDI DI ROMA "LA SAPIENZA" P.ZZALE ALDO MORO, 5
00185 ROMA


Cronache costituzionali dall'estero	Nomos 1-2012
