# Supplementary material for: Peer Review in Law Journals
Source: Front Res Metr Anal. 2021 Dec 8;6:787768. doi: 10.3389/frma.2021.787768 (PMC8692876; doi:10.3389/frma.2021.787768)
Supplement: Supplementary file 3 [file DataSheet2.ZIP › DOCUMENT - 2279-7416.RTF]

How to submit an article to the journal

To submit an article to the journal for publication, it is necessary to fill out the form and upload the Word file.

Texts may be written in Italian, French, English, Spanish and German.

All articles will be assessed through a peer review system.

Each article will be anonymously assigned, for evaluation, to two specialists in the subject matter.

The evaluation of the specialists will be communicated to the author within two months from the date of receipt of the text. In the event of disagreement regarding assessment of the text, by the two reviewers selected, the article will be given to a third reviewer.

The following assessment will be given to the pieces analyzed: - positive (the text will be published) - positive with suggestions for various corrections (the text will be published if corrected) - negative (the text will not be published).

A fee will be required for publication costs: a) articles up to 25 pages € 30; b) articles up to 50 pages € 60; c) articles up to 75 pages € 90. The fee should be payed on the bank account Associazione Culturale "Historia et ius" - Unicredit Agenzia Roma "La Sapienza" - p.le Aldo Moro 5 – 00185 Roma - IBAN: IT.37.E.02008.05227.000103716429.

In drafting the article, the authors must follow the criteria listed here.
