# Supplementary material for: Peer Review in Law Journals
Source: Front Res Metr Anal. 2021 Dec 8;6:787768. doi: 10.3389/frma.2021.787768 (PMC8692876; doi:10.3389/frma.2021.787768)
Supplement: Supplementary file 3 [file DataSheet2.ZIP › DOCUMENT - 2279-9737.RTF]

NORME PER LA VALUTAZIONE E LA PUBBLICAZIONE E CODICE ETICO
NORME PER LA VALUTAZIONE E PUBBLICAZIONE
 
La Rivista di Diritto Bancario seleziona i contributi oggetto di pubblicazione sulla base delle norme seguenti.
I contributi proposti alla Rivista per la pubblicazione vengono assegnati dal sistema informatico a due valutatori, sorteggiati all’interno di un elenco di ordinari, associati e ricercatori in materie giuridiche, estratti da una lista periodicamente soggetta a rinnovamento.
I contributi sono anonimizzati prima dell’invio ai valutatori. Le schede di valutazione sono inviate agli autori previa anonimizzazione.
Qualora uno o entrambi i valutatori esprimano un parere favorevole alla pubblicazione subordinato all’introduzione di modifiche, aggiunte o correzioni, la direzione esecutiva si riserva di negare la pubblicazione dell'articolo. Nel caso in cui la direzione esecutiva decida per la pubblicazione, il comitato editoriale verifica che l’autore abbia apportato le modifiche richieste.
Qualora entrambi i valutatori esprimano parere negativo alla pubblicazione, il contributo viene rifiutato. Qualora solo uno dei valutatori esprima parere negativo alla pubblicazione, il contributo è sottoposto alla direzione esecutiva, la quale assume la decisione finale in ordine alla pubblicazione.
 
CODICE ETICO
La Rivista di Diritto Bancario è una rivista scientifica che si ispira alle migliori prassi internazionali in materia di valutazione e pubblicazione dei contributi. Il presente codice etico si ispira alle Guidelines on good publication practice del Committee on Publication Ethics (COPE).
 
1. Norme per la valutazione e la pubblicazione
La Rivista di Diritto Bancario seleziona i contributi oggetto di pubblicazione sulla base delle "norme per la valutazione e pubblicazione" sopra indicate.
 
2. Doveri dei membri della Direzione
La Direzione è responsabile della definizione e dello sviluppo della linea editoriale della Rivista, nonché del rispetto delle regole in materia di selezione e pubblicazione dei contributi, così come di ogni altra disposizione di legge in materia.
I membri della Direzione si impegnano a svolgere la propria attività in maniera diligente, professionale, obiettiva e non discriminatoria. Essi dichiarano i conflitti di interesse che li riguardano e agiscono in maniera da impedire che provochino conseguenze dannose alla Rivista o a terzi.
 
3. Doveri degli Autori
Gli Autori che sottopongono un contributo alla Rivista devono rispettare le norme di cui all’art. 1, le quali sono altresì pubblicate sul sito internet della Rivista.
Gli Autori si impegnano a proporre alla Rivista soltanto lavori di cui hanno la paternità e che sono originali in ogni propria parte, con chiara e distinta evidenza delle citazioni di lavori altrui.
I contributi proposti alla valutazione devono essere inediti e non sottoposti ad altre Riviste per la pubblicazione, nè possono in seguito essere sottoposti ad altre Riviste per la pubblicazione.
Nel caso di lavori redatti a più mani, è fornita distinta evidenza dei paragrafi attribuiti a ciascuno degli autori. L’attribuzione di paternità deve rispettare l’effettivo contributo fornito alla redazione del contributo.
Gli Autori che presentano un contributo alla valutazione devono comunicare alla Rivista l'esistenza di conflitti di interessi in relazione alla materia trattata.
 
4. Doveri dei Revisori
I Revisori sono coinvolti nelle decisioni in ordine agli articoli sottoposti alla Rivista.
Tutti i contributi pervenuti ai Revisori sono da reputarsi riservati. Pertanto, essi non possono essere divulgati a terzi.
L’attività di Revisione è gratuita. Essa deve essere svolta con diligenza e imparzialità.
Tutte le informazioni riservate o le indicazioni ottenute durante il processo di peer-review devono essere considerate confidenziali e non possono essere usate per altre finalità. I Revisori rifiutano contributi per i quali sussiste un conflitto di interessi.
