# Supplementary material for: Peer Review in Law Journals
Source: Front Res Metr Anal. 2021 Dec 8;6:787768. doi: 10.3389/frma.2021.787768 (PMC8692876; doi:10.3389/frma.2021.787768)
Supplement: Supplementary file 3 [file DataSheet2.ZIP › DOCUMENT - 2281-5147.RTF]

Focus and Scope

Opinio Juris in Comparatione is an electronic full open access journal devoted to “Studies in Comparative and National Law”. It aims at enhancing the dialogue among all legal traditions in a broad sense e.g.:
`.	in a cultural sense in order to increase legal knowledge and to promote circulation of (and competition between) models and legal transplants;
`.	in a more policy perspective, in order to harmonize, when possible/feasible, substantive (or procedural) civil law or private international law rules having regard to the different ‘legal formants’.
Its international character, the fully anonymous peer review process and its openness to publication in several languages are among its challenging features. Finally, it offers an on-line discussion arena where comparative law experts may debate, similar to that experienced by the classic comparative law journals.
Yet, Opinio Juris will not desert exploring national and european law. The aim of diffusing contributions on national law as well, is to expand access to foreign legal materials and ideas to those who do not already have access to the traditional avenues (such as journals in the language of the explored legal system).
Section Policies
ARTICLES
Opinio Juris in Comparatione will give preference to articles under 25,000 words in length including text and footnotes.
Open Submissions Indexed Peer Reviewed
ESSAYS
A piece will be considered an essay if it is 8.000 words or less in length, and its primary purpose is to advance an idea, to summarize a development, or to initiate or engage in discussion.
Open Submissions Indexed Peer Reviewed
CASE NOTES
A case note will not exceed 7.000 words and will focus on an important decision
Open Submissions Indexed Peer Reviewed
SELECTED CONFERENCE PROCEEDINGS
Open Submissions Indexed Peer Reviewed
NEWS AND BOOK REVIEWS
overview of conferences, new books, etc.
Open Submissions Indexed Peer Reviewed
Peer Review Process
The quality control system, aimed at ensuring a serious and anonymous evaluation of the works submitted to the peer-reviewed sections (Articles, Essays, Case notes), is structured in two stages.
`.	The Editorial staff, coordinated by the Editorial Executive Board, provides a first review to examine the consistency of the submission with the Series, the quality of the arguments and of the writing, the internal consistency concerning citation, quotation and paragraph numbering and the proficiency level of the language in which the contribution is submitted.
`.	Then, contributions accepted for review will be sent to up to three independent international reviewers suggested by the Editorial Board (according to the subject matter of the submitted work).
The Editorial Board functions in a flexible way enlisting scholars from individual countries (larger countries would require more than one) who will contribute in various ways to the life of the Journal. Besides suggesting peer reviewers, the Editorial Board will be asked, among other things, to solicit scientific contributions, contribute to the direction of the Journal and its expansion and promote special issues on dedicated topics.
The selected international reviewers will be asked to carry out the anonymous (double-blind) peer-review process by completing a review form that will include the following open questions:
0.	Is the article/essay/ embedded in the scholarly debate, including sufficient awareness of this debate and due consideration of previous works in the subject? If the answer is YES, please try to explain what is its proper originality with regard to content, scope and approach. If the answer is NOT, please try to outline the gaps that could be worked on.
0.	What is the relevance of the contribution in terms of capability to bring “added value” to the current body of knowledge?
0.	Do you recommend the publication of the article? If the answer is YES, would you like to add any comment? If the answer is NOT, what could be changed in order that the contribution can be accepted for publication in our Journal?
0.	Please list the most important strengths of this paper that the author should not lose in the process of revision.
0.	Please confirm the absence of any conflict of interest
The evaluation form is then immediately sent to the author.
Open Access Policy
This journal provides immediate open access to its content on the principle that making research freely available without charge to the public supports a greater global exchange of knowledge.
As a matter of fact, the Open Access movement has triggered a profound and lasting change in the way knowledge is circulated and diffused.
In taking advantage of digital technology evolution and the Internet, the aim is to create a new framework where research results can be more easily accessible to a wider audience at a lower cost.
In the last ten years the Open Access movement has gained worldwide support in the scholarly debate leading to a number of important initiatives that have outlined the main features allowing us to improve access to and the impact of scholarly publications. The vigor of the movement can be highlighted by simply mentioning just some of the most important initiatives: the Budapest Open Access Initiative, 2002; the Bethesda Statement on Open Access Publishing, 2003; the Berlin Declaration on Open Access to knowledge in the Science and Humanities, 2003, endorsed in Italy by the Messina Declaration in 2004.
Furthermore the European Commission has made open access to scientific publications a general principle of Horizon 2020 “The EU Framework Programme for Research and Innovation” for 2014-2020.
Willing to follow what has been called the “Golden Road” to Open Access, Opinio Juris in Comparatione is committed to developing a clear open access policy.
All the contributions published in this Journal will be licensed under a Creative Commons Attribution 3.0 License, which means that readers are allowed to freely read, download, print and share all the works with an acknowledgement of the work’s authorship and initial publication in this journal.
Authors retain copyright and grant the journal right of first publication with the work simultaneously licensed under the above-mentioned Creative Commons Attribution 3.0 License.
Code of Ethics
Ethical Code Of “Opinio Juris in Comparatione”
Opinio Juris in Comparationeis a doubleblind peer-reviewed Open Access scientific journal. Its ethical code takes inspiration from the main national and international ethical Guidelines in Scholarly Publishing (such as, among others, the Principles of Transparency and Best Practice in Scholarly Publishingproviding by the
Committee on Publication Ethics, COPE, http://publicationethics.org/resources/guidelines)
Publisher responsibilities
The Publisher must provide the Journal with adequate resources and the guidance of experts (for example, as far as legal and graphic advice are concerned) in order to carry out his/her role in a professional way and to improve the quality of the Journal.
The Publisher will define in a written agreement his/her relationship with the owner of the Journal and/or with the Editor, according to the best practices of COPE.
The relationship among the Editor in chief, the Editorial Board and the Publisher should be firmly based on the principle of the publishing independence.
The Publisher is committed to developing an open access policy in scientific publishing.
Editors responsibilities
The Editor in Chief and the Editorial Board of Opinio Juris in Comparatione are in charge of the decision of publishing the articles submitted to the Journal. In their decisions, they have to follow the policy and the mission of the Journal.
The Editorial staff, coordinated by the Executive Board, provides a first review to examine the consistency of the submission with the Series, the quality of the arguments and of the writing, the internal consistency concerning citation, quotation and paragraph numbering and the proficiency level of the language in which the contribution is submitted. Then, contributions accepted for review will be sent to up to three independent international reviewers suggested by the Editorial Board (according to the subject matter of the submitted work).The selected international reviewers will be asked to carry out the anonymous (double-blind) peer-review process by completing a review form. The evaluation form is then immediately sent to the author.
The acceptance of the manuscripts is subordinated to the implementation of possible revisions required and to the definitive decision of the Editorial Board.
The Editor in Chief and the Editorial Board are in charge of evaluating the manuscripts on the basis of their scientific content, without discrimination based on race, sex, gender identity, creed, ethnic origin, citizenship, or scientific, academic and political position of the Authors.
If the Editorial Board notices or receives notifications on mistakes or inaccuracies, conflict of interest, plagiarism or other misconducts in a published article, it will make a prompt communication to the Author and the Publisher and will undertake the necessary actions to clear up the matter in an impartial way; moreover, if necessary, it will withdraw the article or will publish a recantation.
Authors responsibilities
The Authors – in submitting an article to the Journal – are obliged to follow the Authors Guidelines provided by the Journal.
The Authors must guarantee the originality of their respective work. The sources have to been duly quoted.
The authorship of the work has to be correctly attributed; moreover, all those who gave an essential and meaningful contribution to the conceiving, organization, accomplishment and revision of the research the article is based on, have to be indicated as Co-Authors or indicated in the acknowledgement according to the respective roles.
All the Authors are obliged to declare unequivocally that there is no conflict of interest which could have influenced the results obtained or the interpretations suggested. Moreover, the Authors must indicate any financing agency of the research or the project the article derives from.
When an Author notices a mistake or an inaccuracy in his/her article, he/she must make a prompt communication to the Editors, giving them all the information required to make the due adjustments.
The researches must be carried out according to ethical rules, with express reference to the Helsinki declaration.
Authors guarantee that the manuscript is freely available and lawful. Authors release the publisher and the Editors from any liability, damage or claim from third parties.The Editors and the Publisher of Opinio Juris in Comparatione are not responsible for the quality, correctness and/or completeness of the information provided by the published articles. Subject to intent and gross negligence, liability of the editors is excluded.
Reviewers responsibilities
By means of the peer-review procedure, the reviewers give assistance to the Editorial Board in taking decisions on the articles submitted. They can, moreover, suggest to the Authors some adjustments or expedients aimed to improve their contribution.
If they don’t feel up to the task they are in charge of, or if they know to be unable to read the works in a timely manner, they are obliged to make a prompt communication to the Editorial Board.
Each work to be read has to be considered confidential; therefore, the works must not be discussed with third parties without the explicit authorization of the Editor in chief. It is strictly forbidden for the reviewers to use the submitted manuscript before the eventual publication or to usurp the scientific authorship of the manuscripts.
The peer review must be carried out from an objective point of view. In this spirit the reviewers must not provide false or unbalanced evaluations in order to take an undue advantage on their own behalf or for others. The reviewers are obliged to state grounds for their evaluation.
The reviewers should report any similarity or overlapping of the work received with other works, as well as any other suspect of misconduct.
Every reserved information or instruction obtained during the peer-review process must be regarded as confidential and cannot be used for other purposes. Despite the anonymous process of evaluation, the reviewers are obliged not to accept articles for which there is a potential conflict of interest due to previous contributions or competition with the Author and/or with his/her institution.
