# Supplementary material for: Peer Review in Law Journals
Source: Front Res Metr Anal. 2021 Dec 8;6:787768. doi: 10.3389/frma.2021.787768 (PMC8692876; doi:10.3389/frma.2021.787768)
Supplement: Supplementary file 3 [file DataSheet2.ZIP › DOCUMENT - 2282-667X.RTF]

Procedura di Revisione

Procedimento di valutazione dei lavori proposti per la pubblicazione
I lavori che aspirano alla pubblicazione vengono inviati, per posta elettronica ed in formato Word, al seguente indirizzo: redazione@orizzontideldirittocommerciale.it e da qui inoltrati all’indirizzo dei componenti della Direzione scientifica.
La Direzione scientifica, espletata una prima delibazione del singolo lavoro, lo avvia al referaggio o revisione scientifica tra pari (peer review), dopo aver espunto dal testo ogni elemento idoneo a consentire l’identificazione dell’autore o dell’autrice.
Per ciascun lavoro (salve le eccezioni che verranno illustrate infra), la revisione scientifica viene affidata, con il metodo del “doppio cieco”, ad uno studioso (referee) esterno alla Direzione scientifica ed alla Redazione della Rivista, e scelto sulla base della riconosciuta esperienza e competenza rispetto ai temi trattati nel lavoro stesso, nonché sulla base dell’assenza di conflitti d’interessi, da un lato, e di legami particolari (di parentela o affinità, di stretta amicizia, di colleganza nel medesimo Dipartimento o nella medesima Scuola accademica) con l’autore o con l’autrice, dall’altro.
Al referee viene richiesto di esprimere, entro il termine massimo di tre settimane, la propria valutazione positiva ovvero negativa sul punto se il lavoro sottoposto al suo esame sia maturo per la  pubblicazione nella Rivista, formulando e motivando un breve giudizio scritto, che viene inviato alla Direzione scientifica, la quale ne cura l’inoltro, in maniera anonima, all’autore/autrice dell’articolo.
Nel caso in cui la valutazione del referee risulti negativa, e comunque in ogni caso in cui si reputi opportuno un riscontro della prima valutazione, la Direzione scientifica della Rivista può decidere collegialmente di interpellare, con le medesime modalità del primo, un secondo referee.
Le schede contenenti le valutazioni espresse dai referees vengono archiviate e conservate, anche in formato elettronico, a cura della Direzione della Rivista.
In linea con le previsioni del Regolamento dell’ANVUR, non sono di regola sottoposti a revisione scientifica (peer review): (a) i lavori pubblicati, a firma della Direzione o di uno o più condirettori, nella rubrica “Editoriale”, trattandosi di lavori specificamente rivolti alla rappresentazione di una determinata linea editoriale della Rivista ovvero al lancio di una determinata proposta culturale, il cui merito non è perciò assoggettabile ad una valutazione esterna; (b) gli articoli scaturiti dalla partecipazione a “I seminari della Rivista ODC”; (c) le rassegne di casi giurisprudenziali, destinate alla rubrica “Giurisprudenza delle Corti superiori”, in quanto aventi carattere eminentemente informativo; (d) le schede di lettura o le recensioni pubblicate sotto la rubrica “Letture”.
In casi eccezionali, la Direzione può assumere direttamente la responsabilità della pubblicazione di singoli contributi senza sottoporli a previa revisione scientifica, segnalando la circostanza e le relative motivazioni in una nota nella prima pagina del contributo.
