# Supplementary material for: Peer Review in Law Journals
Source: Front Res Metr Anal. 2021 Dec 8;6:787768. doi: 10.3389/frma.2021.787768 (PMC8692876; doi:10.3389/frma.2021.787768)
Supplement: Supplementary file 3 [file DataSheet2.ZIP › DOCUMENT - 2284-3531.RTF]

Codice etico
Codice etico e pratiche scorrette inerenti la pubblicazione di lavori scientifici
Il presente codice etico si basa sulle COPE’s Best Practice Guidelines for Journal Editors.
1. Decisione di pubblicazione e doveri del direttore responsabile
La pubblicazione dei risultati e degli esiti della ricerca scientifica è un procedimento complesso e multiforme, che impone a tutti gli attori coinvolti (editore, membri dei boards, referees, autori) rigore, accuratezza ed estrema attenzione.
Il direttore responsabile promuove l'adozione delle best practices internazionali e ne verifica l’applicazione; richiede pubblicazioni originali – redatte nel rispetto del diritto d’autore, conformemente alla licenza adottata, e non sottoposte, allo stesso tempo, a valutazione presso altre riviste – che garantiscano alti standards nella qualità editoriale dei contributi.
Inoltre, il direttore stabilisce e controlla l’utilizzo della peer review come metodo di selezione degli articoli, promuove l’indipendenza della ricerca, condanna senza riserve la violazione del copyright ed ogni pratica di plagio, richiede e promuove contributi originali basati su dati rigorosi, impegnandosi a pubblicare le correzioni di eventuali errori negli articoli pubblicati.
Infine, Il direttore responsabile contribuisce alla riflessione sull'evoluzione dell'editoria accademica, dialoga con ministero dell’Istruzione, dell’Università e della Ricerca, Anvur, Cun e Crui, si confronta con ricercatori e bibliotecari sui temi legati alla diffusione e valorizzazione della  ricerca, con particolare riferimento alla dimensione dell’open access.
2. Doveri degli organi editoriali
La direzione della rivista garantisce la correttezza e l’analiticità dei meccanismi di valutazione utilizzati per esaminare, accettare o respingere gli articoli sottoposti dagli autori interessati ed in particolare vigila metodicamente sul processo di peer review e sulla garanzia del pieno anonimato dei revisori rispetto allo specifico articolo in esame; si impegna, inoltre, ad evitare ogni forma di conflitto di interesse e di discriminazione per motivi di genere, orientamento sessuale o religioso, convinzioni politiche, provenienza geografica.
La direzione della rivista avverte senza indugio l’editore ed il direttore responsabile in caso riscontri, negli articoli in esame, episodi di plagio e potenziali violazioni del diritto d’autore.
Inoltre, qualora venissero accertati gravi errori, situazioni di conflitto di interessi o casi di plagio in un articolo già pubblicato, informa l’editore ed il direttore responsabile e, se appropriato, appronta un erratum o invita a ritirare l’articolo, dandone adeguata e completa pubblicità nelle forme più indicate.
Il direttore responsabile esercita sul contenuto della rivista il controllo necessario ad impedire che nella pubblicazione siano perpetrate condotte passibili di sanzione penale.
Il comitato di redazione è sottoposto alle politiche editoriali della rivista e vincolato al rispetto delle disposizioni di legge vigenti in materia di diffamazione, violazione del copyright e plagio.
Il comitato di redazione è vincolato a non divulgare alcuna informazione sui contributi scientifici inviati ai fini della pubblicazione se non agli autori stessi o a chi sia stato preventivamente ed appositamente autorizzato.
Il materiale inedito contenuto nei contributi sottoposti alla rivista non può essere usato dai membri del comitato di redazione per proprie ricerche senza il consenso dell’autore prestato espressamente e per iscritto.
3. Doveri dei revisori
Il revisore assiste e coadiuva la direzione della rivista nel processo di valutazione degli articoli rispettando i tempi previsti.
La revisione deve essere condotta con metodo obiettivo ed analitico, tramite argomentazioni chiare, precise e documentate.
Il revisore non deve accettare l’esame manoscritti rispetto ai quali si trovi in istato di conflitto di interesse derivante da rapporti di concorrenza, collaborazione, o altro tipo di collegamento con autori, imprese od enti connessi in qualche modo con l’oggetto del contributo presentato.
Il revisore è tenuto ad identificare la presenza di materiale bibliografico rilevante per il contributo da valutare ma non citato dall’autore, e ad indicare agli autori eventuali miglioramenti ed integrazioni utili alla pubblicazione dei contenuti presentati.
Gli articoli presi in esame per la valutazione ai fini dell’eventuale pubblicazione devono essere trattati come documenti riservati. Essi non devono essere mostrati o discussi con chiunque non sia stato preventivamente autorizzato da parte del comitato di redazione.
4. Doveri degli autori
L’autore si impegna a garantire che il contributo sottoposto a valutazione sia originale, inedito e non sottoposto contemporaneamente ad altre riviste ai fini della pubblicazione, e deve accettare le modalità di valutazione e selezione degli articoli ed in particolare il prescritto processo di peer review. Su autorizzazione della Direzione il contributo, dopo essere stato pubblicato nella rivista potrà essere destinato ad altra rivista cartacea per successiva pubblicazione, citandone la fonte.
Qualora l’articolo sia  stato accettato, l’autore riconosce all’editore il diritto alla pubblicazione, autorizzando tutte le modalità di utilizzo previste nella licenza editoriale adottata dalla rivista.
Tutte le eventuali fonti di sostegno economico finanziario al progetto devono essere espressamente indicate.
L’autore ha l’obbligo di citare correttamente e puntualmente le fonti dei contenuti riportati e di ottenere le autorizzazioni necessarie alla pubblicazione di immagini, tabelle o altri contenuti già editi secondo quanto disposto dalla legge sul diritto d’autore. Dichiarazioni fraudolente o volontariamente inesatte costituiscono un comportamento non contrario ai principi del presente codice e sono inaccettabili. Comportano, altresì, conseguenze in merito alla pubblicazione.
L’autore garantisce la veridicità dei dati contenuti nell’elaborato e l’obiettività delle proprie interpretazioni. I dati relativi devono essere riportati con esattezza e in maniera dettagliata per permettere ad altri di replicare l’indagine ed effettuare le controverifiche del caso.
Devono essere inclusi nel contributo presentato, e figurare come autori, tutti coloro che hanno effettivamente partecipato alla stesura del testo, visto e approvato la versione definitiva dello stesso e sono concordano sulla relativa pubblicazione.
Qualora vi siano altri soggetti che abbiano fornito contributi sostanziali relativi a parti rilevanti dell’articolo, devono essere riconosciuti ed appositamente elencati in qualità contributori.
Qualora l’autore riscontri errori significativi o inesattezze nel contributo pubblicato ha il dovere di comunicarlo tempestivamente al comitato di redazione e fornire gli opportuni errata corrige, perché si provveda senza indugio ai necessari emendamenti.
