# Supplementary material for: Peer Review in Law Journals
Source: Front Res Metr Anal. 2021 Dec 8;6:787768. doi: 10.3389/frma.2021.787768 (PMC8692876; doi:10.3389/frma.2021.787768)
Supplement: Supplementary file 3 [file DataSheet2.ZIP › DOCUMENT - 2284-4503.RTF]

 
 
Language 

Journal Content Search 
	
	
	


Browse 
·	By Issue·	 
·	By Author·	 
·	By Title·	 
User 
Username		
Password		
 Remember me	
	
Register 	

Font Size 

·	Home·	 
·	About·	 
·	Archives·	 
·	Biodiritto.org·	 
Home > About the Journal > Editorial Policies 
Editorial Policies
 
·	» Focus and Scope·	 
·	» Section Policies·	 
·	» Peer Review Process·	 
·	» Publication Frequency·	 
·	» Open Access Policy·	 
·	» Referees·	 
·	» Publication ethics and publication malpractice·	 
·	» Cookie Policy·	 
Focus and Scope
 
BioLaw Journal - Rivista di BioDiritto is a quarterly, peer reviewed, free online law journal focusing on the relationships between law and life sciences under a comparative perspective. According to its interdisciplinary nature, the Journal hosts contributions in the fields of law, life sciences and bioethics.
The Journal presents articles, commentaries and book reviews which provide an innovative and original source of reference for academics, lawyers, legal and medical practitioners, law students, and anyone interested in national, European and international biolaw.
BioLaw Journal is indexed by: DoGi-Dottrina Giuridica, Essper, Catalogo italiano dei periodici (ACNP), Google Scholar, Directory of Open Access Scholarly Resources (ROAD), ERIH plus, Open Academic Journals Index, Emerging Sources Citation Index (Clarivate Analytics), Scopus, Journalseek.
 
Section Policies
 
Front Cover
 
Open Submissions	Indexed	Peer Reviewed	
Editorial
 
Open Submissions	Indexed	Peer Reviewed	
Interview
 
Open Submissions	Indexed	Peer Reviewed	
Forum
 
Open Submissions	Indexed	Peer Reviewed	
Call for papers
 
Open Submissions	Indexed	Peer Reviewed	
Focus on
 
Open Submissions	Indexed	Peer Reviewed	
Essays
 
Open Submissions	Indexed	Peer Reviewed	
Perspectives
 
Open Submissions	Indexed	Peer Reviewed	
Commentaries
 
Open Submissions	Indexed	Peer Reviewed	
Artifical Intelligence and Law
 
Open Submissions	Indexed	Peer Reviewed	
CONCLUSIONS
 
Open Submissions	Indexed	Peer Reviewed	
Troubling historical roots and contemporary challenges
 
Open Submissions	Indexed	Peer Reviewed	
Views into the future
 
Open Submissions	Indexed	Peer Reviewed	
 
Peer Review Process
 
All papers submitted to the Journal are subject to the approval of the Steering Committee; in particular, papers submitted to some of the Journal’s sections (Call for papers, Essays and Commentaries) are subject to double blind peer review. Papers that are outside the scope of the Journal or are otherwise judged to be unsuitable by the Steering Committee will be rejected without peer review. Appropriate papers are sent to at least two independent referees for evaluation. Referees advice on the originality and merit of the papers. The Steering Committee decides on publication.
EDITORIAL: written by the member of the Steering Committee or of the Editorial Board that edited the issue.
FORUM/FOCUS ON: collections of invited contributions demanded by the Steering Committee. Invites are addressed to scholars of authority or to selected experts in the relevant subject matter.
CALL FOR PAPERS: papers collected after a public Call for papers and selected by the Steering Committee in compliance with the following criteria:
- Papers are sent to the three members of the Steering Committee upon anonymization;
- autonomous evaluation by the three members of the Steering Committee;
- papers that received three positive evaluations are published; papers with just one positive evaluation are not published; papers which received two positive evaluations are subject to a second review.
ESSAYS: papers subject to double blind peer review (in exceptional cases: invited contributions by scholars of authority and/or experts in the relevant subject matter).
PERSPECTIVES: papers subject to double blind peer review (in exceptional cases: invited contributions by scholars of authority and/or experts in the relevant subject matter).
COMMENTARIES: papers subject to double blind peer review (in exceptional cases: invited contributions by scholars of authority and/or experts in the relevant subject matter).
 
Publication Frequency
 
Journal items will be published collectively, as part of an issue with a frequency of four issues per year.
 
Open Access Policy
 
This journal provides immediate open access to its content on the principle that making research freely available to the public supports a greater global exchange of knowledge.
  

This work is licensed under a Creative Commons Attribution-NonCommercial-NoDerivatives 4.0 International License.
 
Referees
 
List of BioLaw Journal's referees (updated to issue 3/2020): 
Fulvia Abbondante, Pia Acconci, Ugo Adamo, Stefano Agosta, Anna Alberti, Antonino Alì, Salvatore Amato, Daniele Amoroso, Denise Amran, Adriana Apostoli, Marco Arlorio, Marco Azzalini, Gianni Baldini, Maurizio Balistreri, Benedetta Barbisan, Camillo Barbisan, Giuseppina Barcellona, Luisella Battaglia, Francesco Belvisi, rPaolo Benciolini, Carla Bernasconi, Marta Bertolini, Malaika Bianchi, Daniela Bifulco, Andrea Boggio, Carlo Bona, Enrico Bonadio, Sergio Bonini, Sara Bonomelli, Andrea Bonomi, Silvia Borelli, Carlo Botrugno, Barbara Bottalico, Giuditta Brunelli, Maria Esmeralda Bucalo, Claudio Buccelli, Simona Cacace, Mia Caielli, Eugenio Caliceti, Erminia Camassa, Stefano Canestrari, Benedetta Cappiello, Elena Carpanelli, Corrado Caruso, Carlos Maria Romeo Casabona, Maria Casado, Roberto Caso, Stefano Catalano, Giulia Cavaliere, Elisa Cavasino, Eleonora Ceccherini, Alfonso Celotto, Lorenzo Chieffi, Daniele Coduti, Lucilla Conte, Giuseppe Contissa, Cristina Coppola, Federica Coppola, Alessandra Cordiano, Lucia Corso, Fulvio Cortese, Matteo Cosulich, Alessia Ottavia Cozzi, Laila Craighero, Rosaria Cristiano, Marco Croce, Christian Crocetta, Entela Cukani, Cristiano Cupelli, Giacomo D’Amico, Marilisa D’Amico, Luigi D’Andrea, Patrizio Ivo D’Andrea, Francesca Danesi, Marco Dani, Andrea de Bertolini, Maria Vita De Giorgi, Inigo de Miguel Beriain, Stefano Delsignore, Michela Denti, Roberto Dias, Michele Di Bari, Elena Di Carpegna Brivio, Giovanni Di Cosimo, Caterina Di Costanzo,  Angela Di Gregorio, Maurizio Di Masi, Gian Paolo Dolso, Rossana Ducato, Aitziber Emaldi Cirion, Mariachiara Errigo, Laura Fabiano, Fernanda Faini, Elena Falletti, Gianluca Famiglietti, Carla Faralli, Rosanna Fattibene, Elena Ferioli, Matteo Ferrari, Margherita Fiorentini, Gianluigi Fioriglio, Thomaz Fiterman Tedesco, Massimo Foglia, Gabriele Fornasari, Lorena Forni, Antonio Fortino, Aldo Frignani, Tommaso Edoardo Frosini, Enrico Furlan, Simone Gabbi, Ilmira Galimova, Marco Galletti, Matteo Galletti, Daniele Gallo, Lucia Galvagni, Giuseppe Gennari, Simone Gianello, Francesca Giardina, Orsetta Giolo, Federica Giovanella, Maribel Gonzalez Pascual, Luca Gori, Giorgio Grasso, Paolo Guarda,  Andrea Guazzarotti, Stefano Guizzi, Stefanie Hennette Vauchez, Daniele Gallo, Luigi Gaudino, Luca Ghidoni, Federica Grandi, Maria Pia Iadicicco, Paola Iamiceli, Carlo Iannello, Antonio Iannuzzi, Karl Kob, Elisabetta Lamarque, Juan Alberto Lecaros, Benedetta Liberali, Andrea Lollini, Laura Lorello, Anna Lorenzetti, Nicola Lucchi, Nicola Lucifero, Marco Macchia, Enrico Maestri, Raffaele Mafrellotti , Veronica Manca, Michela Manetti, Barbara Marchetti, Francesca Marin, Davide Marino, Valeria Marzocco, Michele Massa, , Ilenia Massa Pinto, Anna Mastromarino, Giuditta Matucci, Paola Mazzina, Davide Mazzon, Antonia Menghini, Davide Menozzi, Franca Meola, Michele Miravalle, Viviana Molaschi, Monia Montorsi, Donatella Morana, Piero Morino, Edmondo Mostacci, Annamaria Nico, Matteo Orlando, Luciano Orsi, Stefano Osella, Fabio Pacini, Laura Palazzani, Monica Palmirani, Elisabetta Palermo Fabris, Davide Paris,  Baldassarre Pastore, Fulvio Pastore, Teresa Pasquino, Andrea Patroni Griffi, Ilja Pavone, Marco Pelissero, Irene Pellizzone, Simone Penasa, Andrea Perin, Giulia Perrone, Roberto Perrone, Marco Pertile, Barbara Pezzini, Mariassunta Piccinni, Cinzia Piciocchi, Lucia Pilati, Alessandra Pioggia, Roberto Pinardi, Cesare Pitea, Federico G. Pizzetti, Marco Plutino, Oreste Pollicino, Annamaria Poggi, Francesca Poggi, Barbara Poggio, Ludovica Poli, Simone Pollo, Salvatore Prisco, Roberto Puccella, Andrea Pugiotto, Edoardo Raffiotta, Chiara Ragni, Giada Ragone, Francesca Rescigno, Fernando Rey Martinez, Giovanni Maria Riccio, Nicola Riva, Ilaria Rivera, Daniele Rodriguez, Francesca Romanin, Roberto Romboli, Laura Ronchetti, Monica Rosini, Emanuele Rossi, Stefano Rossi, Andrea Rovagnati, Antonio Ruggeri, Ilenia Ruggiu, Marco Ruotolo, Carmela Salazar, Barbara Salvatore, Amedeo Santosuosso, Lucia Scaffardi, Simone Scagliarini, Antonio Scalera, Roberto Scarciglia, Stefania Scarponi, Angelo Schillaci, Alexander Schuster, Davide Servetti, Giuseppe Settanni, Caterina Sganga, Paula Siverino Bavio, Giorgio Sobrino, Giovanni Sogari, Paolo Sommaggio, Domenico Sorace, Andrea Spagnolo, Angioletta Sperti, Andrea Stazi, Elettra Stradella, Stefania Stefanelli, Davide Strazzari, Kolis Summerer, Rui Tavares Lanceiro, Diletta Tega, Alessandro Torre, Paola Torretta, Sunita Tripathy, Chiara Tripodina, Lara Trucco, Alberto Turco, Elena Urso, Veronica Valenti, Giuseppe Alessandro Veltri, Maria Carmela Venuti, Paolo Veronesi, Nicoletta Vettori, Simone Vezzani, Corrado Viafora, Benedetta Vimercati, Teresa Violante, Lorenza Violini, Francesco Visioli, Alessandra Viviani, Matteo WIlker, Maria Zanichelli, Nereo Zamperetti, Giovanni Ziccardi, Silvia Zullo.
 
Publication ethics and publication malpractice
 
BioLaw Journal – Rivista di BioDiritto is committed to the respect of ethical values of scientific research, to promote the circulation of science in line with the principles of transparency in contributions’ selection and of Open Access standards.
Authors, editors and referees are required to be aware of and agree upon the following principles, based on the Code of Conduct and Best Practice Guidelines for Journal Editors del COPE – Committee On Publication Ethics.
 
Steering Committee and Editorial Boards
Decisions on publication
The Steering Committee conducts a preliminary screening on submitted papers, to check their adherence to the focus and scope of the Journal and the respect of minimum requirements for publication. In the case of a positive outcome of the preliminary screening, the Steering Committee chooses two referees among the experts in the scientific area; as to invited contributions, the Steering Committee decides on publication upon evaluation of the contribution.
Fair Play
The Steering Committee and the Editorial Boards at any time evaluate manuscripts for their scientific content, granting the pluralism of ideas.
Confidentiality
The Steering Committee and the Editorial Boards must not disclose any information about a submitted manuscript to anyone other than the corresponding author, reviewers and the publisher.
Disclosure and conflicts of interest
Unpublished materials disclosed in a submitted manuscript must not be used in an editor’s own research without the express written consent of the author.
Referees
Manuscripts submitted to BioLaw Journal – Rivista di BioDiritto are sent, after their anonymization, to two reviewers, selected by the Steering Committee among the experts of the relevant scientific discipline. The peer-review procedure contributes to the raising of the quality level of the Journal, it grants the publication of manuscripts that received a positive evaluation by two experts and may also assist the author in improving the paper.
Promptness
Reviewers are required to respect the deadline for the submission of their opinion, in order to help the Editorial Boards in respecting the timing of publication.
Any selected referee who feels unqualified to review the research reported in a manuscript or knows that its prompt review will be impossible should notify the Associate Editors in a timely manner, to permit a prompt substation and an adequate evaluation of submitted manuscripts.
Confidentiality
Any manuscripts received for review must be treated as confidential documents.
Standards of Objectivity
Reviews should be conducted objectively and should exclusively be based on the contents of the contribution. Referees should express their views clearly with supporting arguments.
Disclosure and Conflict of Interest
Information or ideas obtained through peer review must be kept confidential and not used for personal advantage. Reviewers should not consider manuscripts in which they have conflicts of interest and in this case should promptly inform the Associate Editors.
Authors
Originality and Plagiarism
BioLaw Journal considers for publication original and unpublished works. Upon submission, authors shall declare that their manuscript is completely original and that they cited all text and sources they used. The Steering Committee will eventually consider the submission of essays to be published in paper book series or already published on foreign reviews, which are not freely accessible in Italy. Articles or essays already published on online Reviews or websites will not be considered for publication.
Acknowledgement of sources
Proper acknowledgement of the work of others must always be given. Authors should cite publications that have been influential in determining the nature of the reported work and should cite them in adherence to the Journal’s editorial guidelines.
Authorship of the paper
Authorship should be properly assigned; all those who have made significant contributions to the conception, design, execution, or interpretation of the reported study should be listed as co-authors.
Disclosure and Conflicts of Interest
All authors should disclose in their manuscript any financial or other substantive conflicts of interest that might be construed to influence the results or interpretation of their manuscript. All sources of financial support for the project should be disclosed.
Fundamental errors in published works
When an author discovers a significant error or inaccuracy in his/her own published work, it is the author’s obligation to promptly notify the Associate Editors and cooperate with them to retract or correct the paper.
 
Cookie Policy
 
You are viewing BioLaw Journal – Rivista di BioDiritto. By using the website of the journal you agree that we may store cookies in your browser.
If you don't consent to this, but still wish to use the website, you may deactivate cookies from journals.edizioniseed.it in your browser settings.
You will still be able to access all the published articles with cookies disabled, but you won't be able to perform tasks that requires you to log into the Open Journals System
What are cookies?
Cookies are small textfiles that are stored locally on your computer. The use of cookies is a standard technology and is used by the majority of websites.
Most web browsers such as Google Chrome, Opera, Mozilla Firefox, Safari or Internet Explorer are set to automatically accept cookies. But you may choose to change these settings in your browser.
If your browser has disabled cookies, some websites might not function optimally.
What kind of cookies does journals.ediziniseed.it use Open Journal Systems
BioLaw Journal – Rivista di BioDiritto runs on the Open Journal Systems platform as known as OJS. The OJS platform sets a cookie called OJSSID:
Cookie name: OJSSID
Standard expiration time: End of browser session or 30 days
Description: Contains a machine-generated session-id for the OJS-platform that will keep track of your browsing session and log-in to the OJS-webpage. This cookie is normally deleted at the end of the browsing session.
If you check "remember me" when you log in, you may quit your browser and open it at a later time, and you will still be logged in. In that case this cookie will expire after 30 days since your last visit or until you log out and close your browser.
This cookie is necessary in order to log into the OJS platform.
You will still be able to access all articles on the OJS platform with cookies disabled, but you will not be able to perform any tasks that requires the user to be logged in.
Google Analytics
We are using the 3rd party Google Analytics in order to analyze the traffic on our website. Google Analytics stores the following cookies on your computer:
Cookie name: __utma
Standard expiration time: 2 years from set/update
Description: Used to distinguish users and sessions. The cookie is created when the javascript library executes and no existing __utma cookies exists. The cookie is updated every time data is sent to Google Analytics.
You may opt out from being tracked by Google Analytics, read more about this at Google's webpage about this topic: Google Analytics Opt-out Browser Add-on. Read more about the cookies used by Google Analytics.
 
ISSN 2284-4503
Editor University of Trento
Registered by the Tribunal of Trento (act n. 6 11 April 2014)
Cookie Policy
 
