# Supplementary material for: Peer Review in Law Journals
Source: Front Res Metr Anal. 2021 Dec 8;6:787768. doi: 10.3389/frma.2021.787768 (PMC8692876; doi:10.3389/frma.2021.787768)
Supplement: Supplementary file 3 [file DataSheet2.ZIP › DOCUMENT - 2384-9479.RTF]

Valutazione scientifica
Linee operative per la revisione dei contributi pervenuti alla Rivista
La peer review, c.d. revisione paritaria, è finalizzata a garantire il livello qualitativo della Rivista.
A tal fine i contributi pervenuti per la pubblicazione nella Rivista vengono trasmessi dalla Direzione a due revisori, scelti tra Professori di prima e seconda fascia – o a queste abilitati – di Diritto penale e di Diritto processuale penale, italiani e stranieri, tenendo conto delle competenze specifiche.
I due revisori sono selezionati, l’uno, tra i membri del Comitato scientifico per la valutazione e, l’altro, tra i membri del Comitato di Direzione, del Comitato scientifico o del Comitato editoriale. I revisori operano nel rispetto del codice etico della Rivista e dei doveri ivi indicati.
Il contributo viene inviato ai Revisori – il cui nome viene mantenuto riservato dalla Direzione – senza alcuna indicazione del nome dell’Autore.
I Revisori trasmettono alla Direzione, nel rispetto dei tempi indicati, una succinta valutazione secondo un modello (c.d. Scheda di valutazione), adeguatamente motivata, del lavoro indicando la meritevolezza di pubblicazione. 
Qualora il giudizio sia di meritevolezza della pubblicazione, il Revisore può indicare, in prospettiva migliorativa, ulteriori interventi.
In caso di discordanza di giudizio, è il Direttore a valutare se pubblicare il contributo.
Sono oggetto di revisione i contributi destinati alle rubriche contenute nella Rivista web e nella Rivista a stampa. 
I contributi destinati alla rubriche web Osservatorio Corte di cassazione, Il Merito, Osservatorio Corte costituzionale, Osservatorio sulla responsabilità penale degli enti, Dal Parlamento, Dall’Europa, Dalla Comunità internazionale, Dossier e Laboratorio di scrittura sono oggetto di valutazione da parte di un membro del Comitato editoriale – composto da professori e ricercatori universitari, magistrati e avvocati di chiara fama – che opera secondo quanto previsto per il Comitato scientifico per la valutazione, il Comitato di Direzione e il Comitato scientifico.
La documentazione relativa alla procedura di revisione svolta per ciascun contributo è conservata dalla Direzione della Rivista.

Protocollo UPI per la definizione dei criteri di scientificità
Pisa University press ha aderito al protocollo d'intesa tra le Case Editrici aderenti al Coordinamento delle University Press Italiane per la definizione dei criteri di scientificità delle pubblicazioni di alta divulgazione.
Il Protocollo del Coordinamento University Press Italiane intende formulare una proposta operativa che possa definire adeguati criteri di scientificità e individuare concrete procedure di attribuzione dello status di "pubblicazione scientifica" a quei prodotti della ricerca che potranno essere presentati per le diverse istanze di valutazioni (NdV, VQR, ASN, progetti nazionali e europei).
All’interno del processo di valutazione scientifica si terrà conto dell’importanza scientifica dell’argomento, della novità e originalità del testo, delle competenze dell’autore, della coerenza e rigore di applicazione metodologica, della leggibilità della forma in funzione dei criteri di analisi scientifica. 
