# Supplementary material for: Peer Review in Law Journals
Source: Front Res Metr Anal. 2021 Dec 8;6:787768. doi: 10.3389/frma.2021.787768 (PMC8692876; doi:10.3389/frma.2021.787768)
Supplement: Supplementary file 3 [file DataSheet2.ZIP › DOCUMENT - 2421-2156.RTF]

Manuscripts should be sent through Scholastica or ExpressO, or by emailing to the editors in-chief (info@theitalianlawjournal.it).
                                       
Alternatively, we also accept submissions at the following address: ItaLJ Editors, Dipartimento di Scienze Politiche Jean Monnet, Università degli Studi della Campania 'Luigi Vanvitelli', Viale Ellittico 31, 81100 Caserta, Italia.
The Italian Law Journal requests that contributors comply with the following standards.
1. Originality
The Italian Law Journal accepts for publication original and previously unpublished manuscripts. Articles already published elsewhere will not be considered.
2. English-Language Editing
Authors for whom English is not their first language must have their manuscript professionally edited by a native legal English expert and provide a certificate confirming that their manuscript was edited for proper English language, grammar, punctuation, spelling, and style. The use of an English-language editing service does not guarantee acceptance or preference for publication.
3. Citation Format
Please use footnotes rather than endnotes. Footnotes should conform to the House Style of the Journal.
4. Cover Letter and Abstract
All submissions must include, in addition to a copy of the manuscript:
a) a  separate cover letter,  to facilitate our anonymous review process, which should feature the following: the title of the manuscript, an e-mail address, a mailing address, a daytime telephone number.
b) a short abstract  on a separate page.
5. Double-blind Peer Review
All manuscripts are evaluated, first, both by the Editors in chief and one or more members of the Advisory Board. Afterwards, manuscripts are sent to anonymous external referees. The review process will take into account, especially, the compliance of the contents with issues and aims which characterize and inspire the Journal. For manuscripts which are sent by non-English mother-tongue scholars we do monitor the linguistic level. We always seek to review manuscripts within two months after receiving them, notifying author of the acceptance, rejection or need for revision of the manuscript; but it might take longer to reach a decision when submission volumes are high.
6. Open Access Policy and Publishing Ethics
The Italian Law Journal is an open access journal. By submitting a manuscript, the authors retain the rights to the published material. Users can use, reuse, and build upon the material published in The Italian Law Journal, provided that they cite the original source.
Publications in the Italian Law Journal are licensed under the Creative Commons Attribution 3.0 Unported License. The Italian Law Journal conforms to the 'Code of Conduct and Best Practice Guidelines for Journal Editors' developed by the Committee on Publication Ethics (COPE). 
The Publisher and the Journal do not support and are not responsible for any plagiarism concerning any articles. Plagiarized articles will be rejected outright. If any plagiarized article is published by mistake, it will be deleted from our website and contents. Authors are fully responsible for plagiarism and are instructed to take care of plagiarism while submitting their manuscripts. Authors are advised not to submit plagiarized articles.
Thank you for considering submitting your piece to The Italian Law Journal.
-----------------------------------------------------

 
           Publication Ethics and Publication Malpractice Statement
 
 
ItaLJ House Style
SECTION I - Technical  and  Stylistic  Requirements
Article 1: Sections of the journal
The  Italian  Law  Journal  consists  of  the  following  sections:  a) Italian Legal Culture: History and Project; b) Essays; Italian-European Lexicon; c) Hard Cases; d) Book Review; e) Malebolge: Thoughts & Polemics; f) Constitutional Court Watch; g) Corporate and Financial Markets Law.
Article 2: Keywords and abstracts
The author’s name should appear under the title and should be asterisked, with the author’s designation just above the notes.
A list of 5-6 keywords (eg main topics, legislation and case law cited) should precede all contributions.
A short abstract not exceeding 150 words should also precede all essays. Both abstracts and key words will bepublished online.
Article 3: Headings
Headings should be clear and brief.
Please do not number headings, but code them in the margin to indicate the level of importance as follows:
First level – I., II., III., IV., etc
Second level – 1., 2., 3., 4., etc
Third level – a), b), c), d), etc
Fourth level – aa), bb), cc), dd), etc
Please use capital letters only to begin each sentence and proper nouns.
Article 4: Cross references
Cross references in an article should be reduced to a minimum. English terms (eg above/below) should be preferred to Latin (eg ante/post, supra/infra). In particular, please do not use op. cit., loc. cit., ibidem, etc; and use “n 10 above” rather than “op. cit. n 10”.
     ItaLJ House Style 2017
 
