# Supplementary material for: Peer Review in Law Journals
Source: Front Res Metr Anal. 2021 Dec 8;6:787768. doi: 10.3389/frma.2021.787768 (PMC8692876; doi:10.3389/frma.2021.787768)
Supplement: Supplementary file 3 [file DataSheet2.ZIP › DOCUMENT - 2421-2563.RTF]

Regole per la revisione

I contributi, inviati alla Rivista per la pubblicazione, sono soggetti a revisione tra pari a doppio cieco (double blind). È, quindi, garantito l'anonimato dei valutatori e dei valutati.
Vengono sottoposti a revisione tutti i contributi costituenti Articoli e saggi, inseriti in una delle voci tematiche. 
Il Comitato dei revisori è costituito, esclusivamente, da professori ordinari dell'area privatistica, indicati in un apposito elenco pubblicato. 
La revisione è affidata a due membri del Comitato dei revisori, scelti a rotazione dai curatori in base alle indicazioni di settore fatte da ciascun componente. 
Il referee è tenuto a compilare la scheda di valutazione. È garantita la piena autonomia dei revisori rispetto alla Direzione della Rivista.
Soltanto in casi eccezionali, i Curatori assumono, con adeguata motivazione, la responsabilità della pubblicazione.
