# Supplementary material for: Peer Review in Law Journals
Source: Front Res Metr Anal. 2021 Dec 8;6:787768. doi: 10.3389/frma.2021.787768 (PMC8692876; doi:10.3389/frma.2021.787768)
Supplement: Supplementary file 3 [file DataSheet2.ZIP › DOCUMENT - 2459-6531.RTF]

      	
 	
Home 
Journals alphabetically  

Journals by scientific areas 
o	Natural Scienceso	 

§	Natural Sciences (all)§	 
§	Mathematics§	 
§	Physics§	 
§	Geology§	 
§	Chemistry§	 
§	Biology§	 
§	Geophysics§	 
§	Interdisciplinary Natural Sciences§	 

o	Technical Scienceso	 

§	Technical Sciences (all)§	 
§	Architecture and Urbanism§	 
§	Shipbuilding§	 
§	Electrical Engineering§	 
§	Geodesy§	 
§	Civil Engineering§	 
§	Graphic Technology§	 
§	Chemical Engineering§	 
§	Metallurgy§	 
§	Computing§	 
§	Mining, Petroleum and Geology Engineering§	 
§	Mechanical Engineering§	 
§	Traffic and Transport Technology§	 
§	Textile Technology§	 
§	Aviation, Rocket and Space Technology§	 
§	Basic Technical Sciences§	 
§	Interdisciplinary Technical Sciences§	 

o	Biomedicine and Healthcareo	 

§	Biomedicine and Healthcare (all)§	 
§	Basic Medical Sciences§	 
§	Clinical Medical Sciences§	 
§	Public Health and Health Care§	 
§	Veterinary Medicine§	 
§	Dental Medicine§	 
§	Pharmacy§	 

o	Biotechnical Scienceso	 

§	Biotechnical Sciences (all)§	 
§	Agronomy§	 
§	Forestry§	 
§	Wood Technology§	 
§	Biotechnology§	 
§	Food Technology§	 
§	Nutrition§	 
§	Interdisciplinary Technical Sciences§	 

o	Social Scienceso	 

§	Social Sciences (all)§	 
§	Economics§	 
§	Law§	 
§	Political Science§	 
§	Information and Communication Sciences§	 
§	Sociology§	 
§	Psychology§	 
§	Pedagogy§	 
§	Education/Rehabilitation Sciences§	 
§	Speech Therapy§	 
§	Kinesiology§	 
§	Demography§	 
§	Social Activities§	 
§	Security and Defense Science§	 
§	Interdisciplinary Social Sciences§	 

o	Humanistic Scienceso	 

§	Humanistic Sciences (all)§	 
§	Philosophy§	 
§	Theology§	 
§	Philology§	 
§	History§	 
§	History of Art§	 
§	Art Sciences§	 
§	Archeology§	 
§	Ethnology and Anthropology§	 
§	Religious Studies (Interdisciplinary Area)§	 
§	Interdisciplinary Humanistic Studies§	 

o	Field of Arto	 

§	Field of Art (all)§	 
§	Theater Arts (Performing and Media Arts)§	 
§	Film Art (Film, Electronic and Media Art of Moving Pictures)§	 
§	Art of Music§	 
§	Fine Arts§	 
§	Applied Art§	 
§	Art of Dance and Art of Movement§	 
§	Design§	 
§	Literature§	 
§	Interdisciplinary Fields of Art§	 

o	Interdisciplinary Areas of Knowledgeo	 

§	Interdisciplinary Areas of Knowledge (all)§	 
§	Cognitive Science (Natural, Technical, Biomedical and Healthcare, Social and Humanistic Sciences)§	 
§	Geography§	 
§	Integrative Bioethics (Natural, Technical, Biomedical and Healthcare, Social and Humanistic Sciences)§	 
§	Croatian Studies§	 
§	Educational Sciences (Child and Educational Psychology, Sociology of Education, Political Science of Education, Economics of Education, Anthropology of Education, Neurosciences and Early Learning, Educational Disciplines)§	 
§	Gender Studies§	 
§	Biotechnology in Biomedicine (natural science, biomedicine and healthcare, bioethics area§	 
§	Project Management§	 

o	Interdisciplinary Fields of Arto	 

§	Interdisciplinary Fields of Art (all)§	 


Journal editors 
Visit statistics 
Jorunal status 

Authors 
Paper submission 
ORCID iD 

Policies & exchange 
Policies 
Interoperability 


 
 
 
 
 
 


	Croatian Annual of Criminal Sciences and Practice
   


  


 Status in HRČAK:active ISSN 2459-6531 (Print)  ISSN 2670-9996 (Online)  UDK:343Contact:Hrvatsko udruženja za kaznene znanosti i praksu, Trg Republike Hrvatske 14, 10000 ZagrebEmail:Url:https://www.pravo.unizg.hr/hljkppPublisher:Croatian Association of Criminal Law and Practice Trg maršala Tita 14Impressum (87 KB) 

Peer review: peer review, national peer review, double, all papers, double blind review First year of publication: 2016
Frequency (annually): 2

Scientific disciplines and subdisciplines: Law; Date added to HRČAK: 22 August 2017
Preceding journal: Hrvatski ljetopis za kazneno pravo i praksu
Archive


2020   Vol. 27  No. 2     Vol. 27  No. 1   2019   Vol. 26  No. 2     Vol. 26  No. 1   2018   Vol. 25  No. 2     Vol. 25  No. 1   2017   Vol. 24  No. 2     Vol. 24  No. 1   2016   Vol. 23  No. 2     Vol. 23  No. 1   Visits: 102.675 *  	     
Contact 

Article search


Advanced search
Search instructions


My profile
Register
Authorization type change	
Accessibility statement  Privacy policy  Contact 
Srce 	
