# Supplementary material for: Peer Review in Law Journals
Source: Front Res Metr Anal. 2021 Dec 8;6:787768. doi: 10.3389/frma.2021.787768 (PMC8692876; doi:10.3389/frma.2021.787768)
Supplement: Supplementary file 3 [file DataSheet2.ZIP › DOCUMENT - 2459-6639.RTF]

11. 07. 2021.	Forum za sigurnosne studije

Forum za sigurnosne studije


Status u aktivan Hrčku:

ISSN 2459-6639 (Tisak)

Kontakt:	Centar za međunarodne i sigurnosne studije Fakultet političkih znanosti

Lepušićeva 6

10 000 Zagreb

Tel. + 385 (0)1 4642 000

Fax. + 385 (0)1 4655 316

E-mail: godisnjak.foss@gmail.com ruzica.jakesevic@fpzg.hr

Email:	godisnjak.foss@gmail.com

Url:	http://www.fpzg.unizg.hr/izdavastvo/casopisi/foss

Izdavač:	Fakultet političkih znanosti - Centar za međunarodne i sigurnosne studije

Lepušićeva 6, 10 000 Zagreb http://www.fpzg.unizg.hr foss@gmail.com

Upute za autore (357 KB) Impressum (40 KB)

Forum za sigurnosne studije/Forum for Security Studies (FoSS) je znanstveni časopis čiji je izdavač Fakultet političkih znanosti Sveučilišta u Zagrebu – Centar za međunarodne i sigurnosne studije (CeMSS), koji izlazi jednom godišnje.

Prvi broj časopisa objavljen je 2017. godine. U časopisu Forum za sigurnosne studije objavljuju se znanstveni radovi, rezultati znanstvenih istraživanja, studije, prikazi i recenzije knjiga u znanstvenom polju političkih znanosti, sa osobitim naglaskom na sigurnosne studije. Ova godišnja publikacija otvorena je za i znanstvene i stručne doprinose iz svih ostalih politoloških poddisciplina, kao i za radove srodnih polja u području društvenih znanosti (posebice sigurnosne i obrambene znanosti). Svrha časopisa je sustavno objavljivanje radova koji se bave teorijskim promišljanjem pojma i sadržaja sigurnosti, sigurnosnim pitanjima na nacionalnoj, regionalnoj i globalnoj razini, razvojem sigurnosnih politika i sustava, te dinamikom sigurnosnih pojava i procesa u svijetu.

Pri odabiru i objavi radova u Forumu za sigurnosne studije Uredništvo se vodi najvišim standardima za ovu vrstu publikacija, kako u pogledu aktualnosti tema, tako i u pogledu pridržavanja pravila anonimnog i neovisnog recenzentskog postupka.

Tekstovi u časopisu objavljuju se na hrvatskom ili engleskom jeziku.

Rukopisi radova šalju se elektroničkom poštom na adresu godisnjak.foss@gmail.com ili na adresu glavne urednice ruzica.jakesevic@fpzg.hr

Recenzija: vanjske recenzije, podjednako tuzemna i inozemna, dvostruka, samo znanstveni i stručni radovi, dvostruko slijepa

Prva godina izlaženja: 2017

Učestalost izlaženja (godišnje): 1

Područja pokrivanja: Pravo; Politologija; Sociologija; Demografija; Sigurnosne i obrambene znanosti; Interdisciplinarne društvene znanosti;

Uključen u Hrčak: 16. 10. 2017.

Prava korištenja: Časopis Forum za sigurnosne studije jest časopis s otvorenim pristupom (open access journal) što znači da je cjelokupan sadržaj svakog broja dostupan online u elektroničkoj verziji svim zainteresiranim čitateljicama, čitateljima i institucijama bez naplate. Čitatelji (korisnici) imaju pravo čitati, spremiti sadržaj, kopirati ga, prosljeđivati, pretraživati, koristiti poveznice na tekst te ga koristiti u sve druge svrhe sukladno zakonu i akademskim pravilima, a da pritom prethodno ne trebaju tražiti suglasnost izdavača ili autora. Ovo je u skladu s BOAI definicijom otvorenog pristupa. Autorice i autori članaka objavljenih u Forumu za sigurnosne studije zadržavaju autorsko pravo, uključujući i pravo na copyright. Forum za sigurnosne studije prihvaća uvjete licence Creative Commons CC BY-NC-ND.


https://hrcak.srce.hr/forum-za-sigurnosne-studije	1/1
