# Supplementary material for: Peer Review in Law Journals
Source: Front Res Metr Anal. 2021 Dec 8;6:787768. doi: 10.3389/frma.2021.787768 (PMC8692876; doi:10.3389/frma.2021.787768)
Supplement: Supplementary file 3 [file DataSheet2.ZIP › DOCUMENT - 2459-8860.RTF]

01..	Confidentiality
The editor and the editorial staff must not disclose any information about a submitted manuscript to anyone other than the corresponding author, reviewers, potential reviewers, other editorial advisers, and the publisher, as appropriate.
01..	Disclosure and conflicts of interest
`)	Unpublished materials disclosed in a submitted manuscript must not be used in the editor’s own research without the written consent of the author.
`)	Privileged information or ideas obtained through peer review must be kept confidential and not used for personal advantage.
`)	The editor should recuse her/himself from considering manuscripts in which he/she has conflicts of interest resulting from competitive, collaborative, or other relationships or connections with any of the authors, companies, or institutions associated with that particular article.
`)	The editor should require all contributors to disclose relevant competing interests and publish corrections if competing interests are revealed after publication. If needed, other appropriate actions should be taken, such as retraction of the publication.
01..	Involvement and cooperation in investigations
The editor, in conjunction with the publisher, will take adequate steps when ethical complaints concerning a submitted manuscript or published article have been presented. Such measures will generally include contacting the authors of the manuscript or published article and giving due consideration to the respective complaint or claims made. They may also include further communications with the relevant institutions and research bodies. In cases in which a complaint is upheld, the publisher will issue a correction, retraction, a notice of removal, or other note, as may be relevant. Every reported act of unethical publishing behaviour must be looked into, even if it is revealed years after publication.
2. The editorial board members
The publisher will insist on a highly competent editorial board whose members can actively contribute to the development and good management of the journal.
The functions and duties of the editorial board members are:
	supporting and promoting the journal,
	seeking out the best academic contributions and actively encouraging submissions of the manuscripts,
	accepting commissions to pre-review and review submissions, write editorials, reviews and commentaries on articles in their specialist area.
The editor is obliged to:
	consult the editorial board members at least once a year to gauge their opinions about the running of the journal,

	inform them of any changes to the policies of the journal and identify future challenges.
03..	Authorship of the article
Authorship should be limited to those who have made a significant contribution to the conception, design, execution and interpretation of the reported study. All those who have made significant contributions should be listed as co-authors. Where there are others who have participated in certain aspects of the research project, they should be acknowledged or listed as contributors.
It is the responsibility of the corresponding author to ensure that the list of authors is accurate and complete, as well as to make sure that all co-authors have approved the final version of the article and have agreed to its submission for publication.
03..	Disclosure and conflicts of interest
When submitting the manuscript, all authors should disclose any financial or other substantive conflict of interest that might be construed as influencing the results or interpretation of their manuscript. All sources of financial support for the project should be disclosed.
Examples of potential conflicts of interest, which should be disclosed, include employment, consultancies, stock ownership, honoraria, paid expert testimony, patent applications/registrations, and grants or other funding.
03..	Fundamental errors in published works
When the authors discover a significant error or inaccuracy in their own published work, it is their obligation promptly to notify the editor or the publisher and cooperate with the editor on this issue. If the editor or the publisher learns from a third party that a published work contains a significant error, it is the obligation of the authors promptly to correct/retract the article or provide evidence of the correctness of the original article.
4. The REVIEWERS
04..	Contribution to editorial decisions
Peer review assists the editor in making editorial decisions and through the editorial communications with the authors may assist in improving the article. Peer review is an essential component of formal scholarly communication, and lies at the heart of the scientific method. The journal shares the view of many that all scholars who wish to contribute to publications have an obligation to do a fair share of reviewing.
04..	Promptness
Any selected referee who feels unqualified to review the research reported in a manuscript, or knows that a prompt review will be impossible, should notify the editor and excuse themselves from the review process.
04..	Confidentiality
Any manuscripts received for review must be treated as confidential documents. They must not be shown to or discussed with others except as authorized by the editor.
