# Supplementary material for: Peer Review in Law Journals
Source: Front Res Metr Anal. 2021 Dec 8;6:787768. doi: 10.3389/frma.2021.787768 (PMC8692876; doi:10.3389/frma.2021.787768)
Supplement: Supplementary file 3 [file DataSheet2.ZIP › DOCUMENT - 2531-6710.RTF]

About the Journal 
Focus e ambito
La rivista telematica SeD si propone di sviluppare il dibattito nei campi della teoria generale del diritto, la giurisprudenza e la sociologia del diritto.
 Sono accetti articoli, note a sentenza, commenti, atti di convegno e recensioni.
La rivista non prevede pagamento né per l'elaborazione né per la sottomissione degli articoli.
Il Comitato di redazione, prima ancora di procedere alla valutazione del contributo,  ne accerterà la pertinenza all’ambito del settore scientifico-disciplinare IUS20 o SPS12.  la Direzione valuterà se il tema trattato possa comunque essere d'interesse per gli studiosi dei settori sopra indicati.
Il Direttore, sentito il Comitato di redazione, potrà rifiutare la pubblicazione di contributi palesemente compilativi e/o privi dei necessari requisiti di scientificità, originalità, pertinenza.
Processo di Peer Review
I parametri di valutazione delle pubblicazioni scientifiche e della ricerca rendono necessaria la pratica della “revisione dei pari” o “referaggio” per ottenere o conservare la classificazione in fascia “A”. Per ragioni di trasparenza si riportano di seguito le modalità attuative.
Il referaggio è anonimo e doppiamente cieco, non saranno prese in considerazione contributi privi di consenso a sottoporre il testo al referaggio di un esperto del settore scientifico disciplinare, o di settori affini, scelto dalla Direzione in appositi elenchi. La direzione di riserva la facoltà di non sottoporre a referaggio: i contributi di: autori di riconosciuto prestigio accademico, di docenti collocati fuori ruolo le relazioni, le presentazioni e gli interventi a Congressi, Convegni, Tavole rotonde per i quali non sia di fatto possibile rispettare la regola dell'anonimato dell’Autore, solo in tal caso il saggio conterrà in calce l'annotazione "Saggio non soggetto a valutazione").
La valutazione sarà attuata dai Referees mediante compilazione di un questionario  (su originalità, coerenza e correttezza formale) con un giudizio sintetico finale sulla pubblicabilità del saggio.
Gli esiti della valutazione dello scritto possono essere: (a) non pubblicabile; (b) pubblicabile dopo qualche modifica/integrazione, da specificare nel dettaglio; (c) pubblicabile (salvo eventualmente il lavoro di editing per il rispetto dei criteri redazionali). Tranne che in quest’ultimo caso l’esito è comunicato all’Autore a cura della Direzione, nel rispetto dell’anonimato del Referee. 
Open Access Policy
Questa rivista fornisce accesso aperto ai suoi contenuti, ritendendo che rendere le ricerche disponibili liberamente al pubblico migliori lo scambio della conoscenza a livello globale.
Archiviazione digitale
L'Università degli Studi di Milano ha un accordo di archiviazione con le Biblioteche Nazionali Centrali di Firenze e Roma nell'ambito del progetto nazionale Magazzini Digitali.
La rivista ha attivato il servizio PKP PN (Preservation Network) per conservare i contenuti digitali attraverso il loro trasferimento al progetto LOCKSS (Lots of Copies Keep Stuff Safe).
 
Misure antiplagio
Gli editor hanno il compito di agire tempestivamente in caso di errori e comportamenti scorretti, accertati o presunti, sia nei confronti dei contributi pubblicati che di quelli ancora da pubblicare. In caso di errori, pubblicazione fraudolenta o plagio, negli articoli pubblicati o durante il processo di pubblicazione, verranno prese le misure appropriate, seguendo le raccomandazioni e le linee guida di di COPE. Le correzioni avverranno con il dovuto risalto, inclusa la pubblicazione di un erratum (errori nel processo di pubblicazione), rettifica (errori degli autori) o, nei casi più gravi, il ritiro del lavoro interessato. I documenti ritirati verranno conservati online e saranno contrassegnati in modo ben visibile come ritrattazione in tutte le versioni online, incluso il PDF, a garanzia di trasparenza verso i lettori.              
Regole di Correttezza e divieto di plagio
Come rivista soggetta a referaggio anonimo e doppiamente cieco, tanto autori quanto il Comitato scientifico i revisori e l’Editore debbono attenersi a regole di comportamento ispirate al principio di correttezza.
 1 Sulla Pubblicazione
 1.1 La decisione in merito è affidata al Direttore in base alle indicazioni del Comitato Scientifico, anche in relazione alla necessità di rispettare il diritto di autore, garantire l’originalità dei contributi ed evitare il plagio o la diffamazione, secondo quanto indicato in seguito.
1.2 La scelta di cui al punto precedente viene assunta senza alcun riguardo a distinzione di lingua, sesso, religione, origine etnica o razziale, opinioni politiche, filosofiche o sindacali.
 2 Sulla Segretezza
 2.1 Tutte le informazioni relative ai manoscritti sono segrete, e sono poste a conoscenza esclusivamente degli autori, revisori, del direttore, del Comitato Scientifico e dell’Editore.
2.2 Coloro ce vengono a conoscenza del contenuto dei manoscritti non ne debbono fare uso se non nei limiti in cui ciò sia consentito dal loro ruolo nel processo editoriale o dall’autore stesso.
 3 Sul processo di revisione
 3.1 I revisori accettano l’incarico solo se verificano di essere qualificati per effettuare la revisione, se non sono in conflitto di interessi e possano realizzare l’attività entro il tempo concordato.
3.2 Ogni informazione relativa ai documenti oggetto di revisione è confidenziale e non può essere divulgata senza autorizzazione del direttore.
 3.3 L’attività dei revisori deve ispirarsi ai principi di imparzialità ed oggettività esprimendo in termini chiari e appropriati i propri giudizi
 3.4 E’ compito dei revisori segnalare al Direttore eventuali citazioni occulte o altre ipotesi di coincidenza tra gli argomenti trattati da altri autori.
  4 Sulla posizione degli Autori
 4.1 Gli autori mantengono i diritti d’autore ed ogni altro diritto di utilizzazione economica sui manoscritti pubblicati.
 4.2 Gli autori devono assicurare l’originalità dei manoscritti e nel caso siano utilizzati parti di soggetti terzi che questi siano correttamente citati.
4.3 Gli autori devono astenersi dal sottoporre a valutazione articoli già pubblicati o accettati per la pubblicazione su altre riviste
4.4 Nel caso di più autori del medesimo manoscritto essi avranno cura di identificare le parti attribuite a ciascuno di essi.
4.5 Gli autori devono segnalare ogni potenziale confitto di interessi, economico o di altro tipo che coinvolga il manoscritto in questione.
4.6 Gli Autori devono collaborare per la segnalazione e l’eliminazione degli errori di stampa con la direzione del giornale.
Frequenza di pubblicazione
La pubblicazione esce due volte l’anno riunendo i contributi in fascicoli semestrali. I singoli articoli, note a sentenza, commenti, atti di convegno sono pubblicati man mano che il processo di referaggio è concluso e vanno a comporre i due fascicoli annuali. 
Articles and submissions processing charges (APC)
Società e Diritti non richiede alcun contributo per la pubblicazione (APC).
Codice etico
“SED Rivista Società e Diritti” è una rivista scientifica peer-reviewed che si ispira al codice etico delle pubblicazioni elaborato da COPE, Committee on Publication Ethics, Best Practice Guidelines for Journal Editors. (link a url http://publicationethics.org/resources/guidelines)
 Doveri e compiti del Direttore e del Comitato di Direzione
Il Direttore e il Comitato di Redazione sono i soli responsabili della decisione di pubblicare gli articoli sottoposti alla rivista stessa. Nelle loro decisioni, essi sono tenuti a rispettare le linee di indirizzo della rivista.
Il Direttore è nominato in seno al comitato di direzione e dura in carica 7 anni, salvo dimissione anticipata. Il Comitato di direzione eviterà intromissioni o influenze esterne per garantire la correttezza e scientificità delle scelte editoriali.
Il Comitato di direzione si compone dei redattori della rivista in numero non inferiore a tre.
 
Doveri dei Redattori
Gli articoli scelti verranno sottoposti alla valutazione di uno o più revisori e la loro accettazione è subordinata all’esecuzione di eventuali modifiche richieste e al parere conclusivo del Comitato di Redazione.
La Direzione e il Comitato di Redazione sono tenuti a valutare i manoscritti per il loro contenuto scientifico, senza distinzione di razza, sesso, orientamento sessuale, credo religioso, origine etnica, cittadinanza, di orientamento scientifico, accademico o politico degli autori.
Se il Comitato di Redazione rileva o riceve segnalazioni in merito a errori o imprecisioni, conflitto di interessi o plagio in un articolo pubblicato, ne darà tempestiva comunicazione all’autore e all’editore e intraprenderà le azioni necessarie per chiarire la questione e, in caso di necessità, ritirerà l’articolo o pubblicherà una ritrattazione.
 
Doveri degli Autori
Gli autori, nel proporre un articolo alla rivista, devono attenersi alle Norme per gli autori consultabili sul sito internet della rivista.
Gli autori sono tenuti ad assicurare di avere redatto un lavoro originale in ogni sua parte e di avere debitamente citato tutti i testi utilizzati. Qualora siano utilizzati il lavoro e/o le parole di altri autori, queste devono essere opportunamente parafrasate o letteralmente citate.
Va correttamente attribuita la paternità dell’opera e vanno indicati come coautori tutti coloro che abbiano dato un contributo significativo all'ideazione, all’organizzazione, alla realizzazione e alla rielaborazione della ricerca che è alla base dell’articolo.
Tutti gli autori sono tenuti a dichiarare esplicitamente che non sussistono conflitti di interessi che potrebbero aver condizionato i risultati conseguiti o le interpretazioni proposte. Gli autori devono inoltre indicare gli eventuali enti finanziatori della ricerca e/o del progetto dal quale scaturisce l’articolo.
I manoscritti in fase di valutazione non devono essere sottoposti ad altre riviste ai fini di pubblicazione.
Quando un autore individua in un suo articolo un errore o un’inesattezza rilevante, è tenuto a informare tempestivamente la Redazione e a fornirle tutte le informazioni necessarie per indicare le doverose correzioni del caso.
I protocolli di studio dei lavori originali, qualora ve ne saia l’obbligo giuridico, saranno preventivamente autorizzati dai comitati etici di riferimento degli Autori e le ricerche devono essere condotte secondo norme etiche con specifico richiamo alla dichiarazione di Helsinki.
 
Doveri dei Revisori
Attraverso la procedura del peer-review i revisori assistono il Comitato di Redazione nell’assumere decisioni sugli articoli proposti, e inoltre possono suggerire all’autore correzioni e accorgimenti tesi a migliorare il proprio contributo.
Qualora non si sentano adeguati al compito proposto o sappiano di non potere procedere alla lettura dei lavori nei tempi richiesti sono tenuti a comunicarlo tempestivamente al Comitato di Redazione.
Ogni testo assegnato in lettura deve essere considerato riservato; pertanto tali testi non devono essere discussi con altre persone senza l’esplicita autorizzazione della Direzione.
La revisione deve essere effettuata in modo oggettivo. I revisori sono tenuti a motivare adeguatamente i giudizi espressi.
I revisori s’impegnano a segnalare al Comitato di Redazione eventuali somiglianze o sovrapposizioni del testo ricevuto con altre opere a loro note.
Tutte le informazioni riservate o le indicazioni ottenute durante il processo di peer-review devono essere considerate confidenziali e non possono essere usate per altre finalità. I revisori sono tenuti a non accettare in lettura articoli per i quali sussiste un conflitto di interessi dovuto a precedenti rapporti di collaborazione o di concorrenza con l’autore e/o con la sua istituzione di appartenenza.
 
Doveri dell’Editore
Qualora la proprietà decida di affidare la pubblicazione ad un impresa editrice stabilirà mediante contratto che l’editore si impegni a fornire  alla rivista risorse adeguate nonché la guida di esperti (p. e. per la consulenza grafica, legale ecc.), così da svolgere il proprio ruolo in modo professionale e aumentare la qualità del giornale.
L’editore dovrebbe avere un contratto scritto che definisca il suo rapporto con il proprietario della rivista e/o con la Direzione.
I termini di detto contratto devono essere in linea con il Codice di condotta per editori di riviste scientifiche messo a punto da COPE.
Il rapporto tra Direzione, Comitato di Redazione ed editore deve basarsi saldamente sul principio di indipendenza editoriale.
Sponsors
La rivista non gode di sponsorizzazioni
