# Supplementary material for: Peer Review in Law Journals
Source: Front Res Metr Anal. 2021 Dec 8;6:787768. doi: 10.3389/frma.2021.787768 (PMC8692876; doi:10.3389/frma.2021.787768)
Supplement: Supplementary file 3 [file DataSheet2.ZIP › DOCUMENT - 2584-3613.RTF]

PARAGRAF god. 2, br. 1/2018.

289

INSTRUCTIONS FOR AUTHORS

"Paragraf" is an annual journal containing the best student papers dealing with legal and social issues. It contains papers of the students of both the Faculty of Law Osijek and other faculties in the Republic of Croatia. The Editorial Board may also approve of the publication of a paper from a foreign faculty. The periodical is published by the Faculty of Law Osijek. Papers submitted for publication must deal with legal, economic, sociological or other social topics.

Papers submitted for publication must not have been published in any other professional or scientific publication. Every author signs the Authorship Statement (the form may be downloaded from the journal website). Having signed the Statement, the author/ authoress guarantees that the submitted paper is his/her original manuscript, that it does not violate any ethical rules or copyrights, and that it strictly follows the rules and methodology of scientific work regarding the citation of other researcher work results. All submitted papers undergo authentication and anti-plagiarism tests based on available and verified software.

When sending their papers to the Editorial Board, authors should also attach a recommendation letter from a university professor for the publication of the paper. The recommendation form can be downloaded from the journal website.

Papers are sent to the Editorial Board in electronic form (format.doc) using the following E-mail address: paragraf@pravos.hr. The recommendation letter and one printed copy of the paper are sent to the following address:

"Paragraf"

Josip Juraj Strossmayer University of Osijek, Faculty of Law Osijek Stjepana Radića 13

31 000 Osijek Republic of Croatia

The author/ authoress retains copyrights for the published paper, but gives the journal the right of first publication. The author/authoress has the permission to publish papers accepted for publication or already published in "Paragraf" in other publications but with the remark that the paper was already published in the "Paragraf" journal. By submitting the paper for publication, authors/authoresses agree to the publication of an electronic version of the paper.

All papers (with the exception of reviews, critical overviews, conference reports etc.) must undergo a review procedure, and only positively reviewed papers are published. The papers are categorised as follows: original scientific paper, preliminary communication, review article, and professional paper. A final decision about paper categorisation is made by the Editorial Board primarily on the basis of the reviewers' opinion. The Edi-

PARAGRAF god. 2, br. 1/2018.

290

torial Board reserves the right to adjust the paper to general editing rules for journals and the rules of the standard Croatian language.

Papers submitted for publication must meet the following technical requirements?

0.	Papers submitted for publication may be written in Croatian or in English.

0.	A maximum of three persons may be accepted as authors of one paper.

0.	The title of the paper must be followed by the name and family name of the author/ authors, name of the faculty, current year of study, and E-mail address.

0.	The paper must contain an abstract which must not exceed 250 words. The abstract must be followed by (up to five) key words.

0.	The title of the paper and the abstract with the key words must also be submitted in English.

0.	The main part of the paper must not exceed 32 pages (57,600 characters, including blank spaces and footnotes).

0.	Character style, character size, spacing, and alignment must be set as follows:

Paper title – Times New Roman, uppercase letters, character size 14, spacing 1.5, centred;
Subtitles of the paper – Times New Roman, character size 12, spacing 1.0, left alignment;
Information on authors – Times New Roman, character size 12, spacing 1.0, left alignment;
Abstract, key words, body text and the list of references – Times New Roman, character size 12, spacing 1.0, justified alignment;
Footnotes – Times New Roman, character size 10, spacing 1.0, justified alignment.

0.	The title and all subtitles should be bolded.

0.	Latin expressions and abbreviations (ibid., op. cit.) as well as the words from other foreign languages are always italicised.

0.	At the end of the paper, the authors must provide a list of references that have been consulted which should be classified into the following groups: books, articles, sources of law, Internet sources, and other sources; within each group bibliographical notes are listed alphabetically. References on the list must be written according to citation rules.

0.	The authors/authoresses are obliged to observe the citation rules throughout their paper; the first citation contains a full description of the bibliographic unit.

Books – Last name of the author, first name of the author, title, publisher, publishing place, year of publication, number of the page referred to, e.g.

PARAGRAF god. 2, br. 1/2018.

291

	Andrassy, Juraj; Bakotić, Božidar; Seršić, Maja; Vukas, Budislav, Međunarod-no pravo 1, Školska knjiga, Zagreb, 2010, p. 78.

Articles – Last name of the author, first name of the author, title of the article, name of the periodical, volume, number and year of publication, number of the page referred to, (in the list of references pages must be specified from – to), e.g.

	Tucak, Ivana, Pravni odnosi in rem i pravni odnosi in personam, Pravni vjesnik, Vol. 27, No. 2, 2011, p. 9.

In the list of references:

	Tucak, Ivana, Pravni odnosi in rem i pravni odnosi in personam, Pravni vjesnik, Vol. 27, No. 2, 2011, pp. 7–23.

Sources of law – name of the source, official gazette, number of the gazette in which the source of law was published, e.g.:

	Strategy for Sustainable Development of the Republic of Croatia (Strategija održivog razvitka Republike Hrvatske), Official Gazette, No. 30/2009

	United Nations Convention on the Law of the Sea, Official Gazette, International Agreements, No. 9/2000

	Regulation (EU) No 1177/2010 of the European Parliament and of the Council of 24 November 2010 concerning the rights of passengers when travelling by sea and inland waterway and amending Regulation (EC) No 2006/2004, OJ L 334, 17. 12. 2010, pp. 1–16.

Internet sources – title, website title, the last access date

	Prava putnika u autobusnom prijevozu, http://europa.eu/youreurope/citizens/travel/passenger-rights/bus-and-coach/index-hr.htm, accessed 5 Oc-tober, 2015.
