# Supplementary material for: Peer Review in Law Journals
Source: Front Res Metr Anal. 2021 Dec 8;6:787768. doi: 10.3389/frma.2021.787768 (PMC8692876; doi:10.3389/frma.2021.787768)
Supplement: Supplementary file 3 [file DataSheet2.ZIP › DOCUMENT - 2584-5055.RTF]

Upute autorima


Zbornik Sveučilišta Libertas objavljuje znanstvene i stručne članke iz svih znan-stvenih područja koja se mogu studirati na Libertas međunarodnom sveučilištu: po-slovne ekonomije sa svim svojim područjima, znanosti o međunarodnim odnosima i diplomaciji, socijalne gerontologije, poslovne sigurnosti, menadžmentu sporta te dramske i scenske umjetnosti.

Članci podliježu recenziji (dva anonimna i nezavisna recenzenta) i kategoriziraju se na prijedlog recenzenata u sljedeće kategorije:

0.	Izvorni znanstveni rad (originalno znanstveno djelo u kojem su izneseni novi rezultati fundamentalnih ili primijenjenih istraživanja. Tu spadaju nove, još ne-poznate znanstvene činjenice, spoznaje i teorije koje predstavljaju doprinos zna-nosti, a djelo je napisano tako da bilo koji kvalificirani istraživač na temelju da-nih informacija može uz ista takva istraživanja doći do istih rezultata, zapažanja, proračuna ili teorijskih izvoda kao i autor).
0.	Prethodno priopćenje (znanstveno djelo koje sadrži znanstvene spoznaje ili re-zultate znanstvenih istraživanja čiji karakter zahtijeva objavljivanje. Ovaj znan-stveni članak obvezno sadrži jednu ili više znanstvenih informacija, ali bez do-voljno pojedinosti koje bi čitatelju omogućile provjeru iznesenih znanstvenih spoznaja, rezultata istraživanja i znanstvenih informacija kako je to moguće kod originalnog znanstvenog članka).
0.	Pregledni članak (znanstveno djelo koje sadrži cjelovit prikaz o određenom pi-tanju ili problemu istraživanja o kojemu je već objavljena znanstvena informaci-ja odnosno rezultat znanstvenog istraživanja ili znanstvenih spoznaja. Ne mora sadržavati originalne – nove rezultate istraživanja već se može temeljiti na već objavljenim rezultatima istraživanja, iako može sadržavati i autorove originalne i nove rezultate istraživanja, koji obično ne predstavljaju bitan element predme-ta istraživanja).

0.	Izlaganje sa znanstvenog skupa (ako su već recenzirani pismenim recenzija-ma na znanstvenom skupu, ubrajaju se u znanstvene članke – znanstvena djela. U suprotnom slučaju radi se o stručnom članku. Konferencijsko priopćenje –

381

Zbornik sveučilišta Libertas, 1-2, 2017.


znanstveno predavanje može biti izvorni znanstveni članak, prethodno priopće-nje, pregledni članak ili stručni članak).

0.	Stručni rad (djelo u kojem se iznose korisne informacije i spoznaje do kojih se u pravilu došlo razvojnim istraživanjem, a ne fundamentalnim i primijenjenim istraživanjima. Takav članak ne sadrži originalne rezultate istraživanja nego se u njemu iznose već poznate spoznaje radi primjene u teoriji i praksi i širenja već poznatih spoznaja, stavova i teorija za potrebe znanstvene teorije i gospodarske prakse).

Prijevodi značajnih radova na hrvatski jezik, koji po svom sadržaju mogu po-buditi interes znanstvene i stručne javnosti, također se objavljuju u Zborniku. Opseg prijevoda ne smije biti veći od 20 kartica teksta (jedna kartica - 1800 znakova).

Prikazi knjiga i ostalih publikacija trebaju biti napisani kao kritičke recenzije, a opseg prikaza ne bi trebao prelaziti 5 kartica teksta.

Preporučuje se nastavnicima Libertas međunarodnog sveučilišta da svoje rado-ve za Zbornik prijavljuju i u koautorstvu sa studentima (u pravilu sa zadnje godine preddiplomskog ili sa specijalističkog diplomskog studija). Ovo je nastavnicima važno zbog njihova izbora u viša nastavna zvanja.

Radovi poslani Uredništvu zbornika moraju zadovoljiti sljedeće:

	rukopis ne smije biti već objavljen ili poslan drugom izdavaču na objavljivanje;

	opseg rada ne smije biti manji od 12 kartica teksta (21000 znakova) niti veći od 24 kartice teksta (43.000 znakova ), uključujući sažetak, fusnote i literaturu;

	zaglavlje članka treba sadržavati jasan, koncizan i informativan naslov članka, ime i prezime autora, titulu i zvanje, te naziv i adresu ustanove u kojoj je autor zaposlen;

	ispod naslova rada treba napisati sažetak na hrvatskom jeziku (do 150 riječi), a u kojem se u trećem licu na skraćen način opisuje sadržaj članka i rezultati istra-živanja, te istaknuti četiri do osam ključnih riječi;

	sažetak i ključne riječi trebaju biti prevedeni na engleski jezik i priloženi ruko-pisu
	grafički prikazi, slike i sl. predaju se (za sada) isključivo u crno-bijeloj tehnici;

	korištenu literaturu treba popisati abecednim redoslijedom prema prezimenu autora i kronološkim redom za radove istog autora, a podaci o navedenom djelu u popisu literature moraju sadržavati izdavača, mjesto i godinu izdanja;

	autori se trebaju pridržavati propisane metodologije za izradu znanstvenih i stručnih radova, kako je to opisano u odgovarajućim knjigama koje se bave tom problematikom,

	sve ostalo radi se prema pravilima o pisanju stručnih i znanstvenih radova te ko-rištenju znanstvene metodologije u skladu s propisima struke.

382


Upute autorima


Ispravno navođenje referenci u tekstu

Citiranje i parafraziranje u tekstu mora biti konzistentno provedeno i u skladu s pravilima. Obvezno je korištenje APA stila pri navođenju izvora. Eventualno odstu-panje od APA stila moguće je jedino u dogovoru s uredništvom.

Izvore treba navoditi u tekstu (kao tekstnote), a ne u bilješkama (fusnote). Re-ferenca se stavlja u zagrade i sadrži prezime autora, godinu izdanja, te, ako je riječ o citatu, broj stranice; na primjer:

——  (Kasapović, 2015) ili (Kasapović, 2015: 122)

——  Ako rad ima dva autora, treba navesti oba, na primjer:

——  (Andrijanić i Pavlović, 2016).

——  U slučaju zajedničkog rada tri ili više autora može se koristiti sljedeći oblik:

——  (Merkaš i dr. 2016).

Sve reference u tekstu navode se kao i prvi put, odnosno ne koriste se oblici po-put „ibid.", „op. cit." i slično. Autorima se sugerira da bilješke rezerviraju isključivo za komentiranje ili dopunu rečenog u tekstu, a ne za navođenje izvora.

Izrada popisa literature prema APA stilu – primjeri:

——  Knjiga – jedan autor:

Zandi, Mark (2010). Financijski šok. Zagreb: ZŠEM.

——  Knjiga – više autora:

Andrijanić, Ivo i Pavlović, Duško (2016). Međunarodno poslovanje. Zagreb: Plejada

——  Rad iz zbornika:

Zgurić, Borna (2015) „Povijest i politika Egipta", u: Bliski istok: Povijest i politika, ur. Mirjana Kasapović, 45-72, Zagreb: Fakultet političkih znanosti.

——  Članak u časopisu:

Boban, Davor (2014) „Izborne reforme u Ukrajini", Politička misao, 51 (2), 34-51.

——  Neobjavljena doktorska disertacija:

Prezime, X. (Godina). Naslov disertacije. (Neobjavljena doktorska disertacija). Naziv institucije, Lokacija.

——  Zakoni:

Zakon o lokalnoj i područnoj (regionalnoj) samoupravi. Narodne novine, 33/2001, 129/2005.

——  Članak u novinama (autor nije potpisan):

„Naslov članka", (Datum izdavanja). Naziv novina, str. xx. Ostali primjeri mogu se vidjeti na http://www.apastyle.org/


383


Zbornik sveučilišta Libertas, 1-2, 2017.


Prijavljivanje, prihvaćanje ili odbijanje prispjelih radova:

	Uvjet za objavljivanje članka u Zborniku je da članak bude prihvaćen od strane Uredništva Zbornika Sveučilišta Libertas te prođe recenzentski postupak kod dvaju anonimnih recenzenata koje je odredilo uredništvo
	Uredništvo prima prijave isključivo do datuma kojeg je odredilo kao zadnji dan zaprimanja prijedloga tema članaka za objavu. Radovi se šalju na e-mail adresu urednika Zbornika: vfilipovic@libertas.hr
	Na temelju izvješća recenzenata Uredništvo Zbornika donosi odluku o prihva-ćanju prispjelih radova za objavljivanje ili o njihovu odbijanju za objavljivanje s obrazloženjem. Konačna odluka Uredništva o objavljivanju članka ovisi o pozi-tivnom mišljenju recenzenta. U slučaju neslaganja oba recenzenta o podobnosti članka za objavu – konačnu odluku o objavljivanju donosi Uredništvo Zbornika. Kategorizacija rada ovisi o ocjeni recenzenta.

	Prihvaćanjem kategoriziranih članaka za objavljivanje obvezuje se autor da isti članak ne objavljuje na drugom mjestu i bez dopuštenja Uredništva Zbornika.

	Nakon objavljivanja rada autor dobiva jedan primjerak Zbornika u kojem je nje-gov rad objavljen.

	Za sve eventualne nejasnoće potencijalni se autori mogu javiti glavnom uredni-ku Zbornika doc.dr.sc. Vladimiru Filipoviću na e-mail: vfilipovic@libertas.hr

U Zagrebu, 3. svibnja 2016.	Za uredništvo

Zbornika sveučilišta Libertas

doc. dr. sc. Vladimir Filipović, glavni urednik


384
