# Supplementary material for: Peer Review in Law Journals
Source: Front Res Metr Anal. 2021 Dec 8;6:787768. doi: 10.3389/frma.2021.787768 (PMC8692876; doi:10.3389/frma.2021.787768)
Supplement: Supplementary file 3 [file DataSheet2.ZIP › DOCUMENT - 2671-2113.RTF]

UPUTE ZA PISANJE SAŽETKA ZA PRIJAVU RADA


	sažetak treba imati 250 do 300 riječi s izdvojenih 5 do 6 ključnih riječi

	sažetak se piše u Microsoft Word za Windowse

	font: Times New Roman, veličina 12, prored 1.5

	margine (gore, dole, lijevo, desno): 2,5 cm

	jezik sažetka:

·	prilozi hrvatskoga govornoga područja: hrvatski jezik

·	prilozi engleskoga govornoga područja: engleski jezik

·	prilozi njemačkoga govornoga područja: njemački jezik i engleski jezik

Sažetak treba sadržavati naslov rada, kratak opis teme i svrhe rada, opis pristupa obradi teme, popis glavnih pitanja koja će biti obrađena u cjelovitom radu.

Sažetak treba sadržavati podatke o autoru: ime i prezime autora sažetka, titule, puni službeni naziv institucije u kojoj je autor zaposlen, adresu i podatke za kontakt (tel., mob., e-mail adresa)

Sažetak poslati na huo-casopis@huo.hr.

Radovi u časopisu Hrvatski časopis za OSIGURANJE (u daljnjem tekstu: časopis) objavljuju se isključivo na hrvatskom jeziku. Radovi pisani drugim jezicima biti će prevedeni odnosno usklađeni s hrvatskim jezičnim standardima.


UPUTE ZA PISANJE CJELOVITOG RADA


Radovi za objavu u časopisu moraju biti napisani prema uputama u nastavku i šalju se Uredništvu časopisa na e-mail adresu: huo-casopis@huo.hr. Nakon primitka rada Uredništvo i glavni i odgovorni urednik odlučuju zadovoljava li pristigli rad kriterije za objavu.

Kategorizacija radova koji se objavljuju u časopisu

·	Izvorni znanstveni rad je članak čiji znanstveni doprinos može biti istraživačke i/ili teorijske naravi. Prvi sadrži neobjavljene rezultate izvornih znanstvenih istraživanja u cjelovitom obliku odnosno uključuje metodološku, analitičku i interpretativnu razinu teksta te znanstvenu argumentiranost i vjerodostojnost. Drugi sadrži sustavne kritičke preglede i meta-analize pri čemu se izvornost doprinosa određuje s obzirom na fokus, širinu, argumentiranost rasprave i odnos prema suvremenoj obrađenosti teme.

1

Hrvatski časopis za OSIGURANJE


·	Prethodno priopćenje je znanstveni članak koji sadrži neobjavljene preliminarne rezultate znanstvenog istraživanja koje je u tijeku ili teorijski postavljen problem i okosnice argumentacije, ali bez cjelovite razrade.

·	Pregledni rad je znanstveni članak koji sadrži sažet prikaz stanja i tendencija razvoja jednog znanstvenog područja, teorijskog problema ili istraživačke teme.

·	Izlaganje sa znanstvenog skupa može biti objavljeno samo kao cjelovit rad koji je prethodno referiran na znanstvenom skupu, i to ako u obliku cjelovitog članka nije objavljen u zborniku skupa.

·	Stručni rad donosi korisne priloge iz područja struke kao što je dokumentacija građe (bez teorijske, metodološke i analitičke obrade), korištenje već objavljenih rezultata znanstvenoga istraživanja s fokusom na primjenu u praksi ili na njihovo širenje (obrazovna svrha) ili sažet i kritičan pregled odabrane aktualne teme. Stručni radovi svojom razumljivošću moraju biti dostupni širokom, ne usko specijaliziranom, čitateljskom profilu.

Kategorizaciju rada predlažu autori. Konačnu odluku o kategorizaciji, uzimajući u obzir prijedlog autora i ocjene recenzenata, donosi glavni i odgovorni urednik odnosno u slučaju dvojbi glavni urednik i još najmanje dvojica članova Uredničkog vijeća.

Postupak recenziranja

Ako glavni i odgovorni urednik zaključi da rad zadovoljava kriterije za objavu u časopisu, Uredništvo pokreće postupak recenziranja. Uredništvo samostalno odlučuje o objavi rada bez obzira na recenzije.

Recenzenti svoje recenzije dostavljaju Uredništvu na posebnom obrascu koje im Uredništvo dostavlja prilikom slanja molbe za recenziranjem.

Rukopis recenziraju najmanje dva nepristrana recenzenta, a recenzija je jednostruko slijepa. Recenzije se dostavljaju Uredništvu na adresu e-pošte: huo-casopis@huo.hr.

Prema potrebi, nakon ocjene recenzenata i glavnog i odgovornog urednika, Uredništvo može rad vratiti autoru s obrazloženjem i zahtjevima za doradu i ispravak rada. Ispravljene i/ili dorađene radove autori su obvezni vratiti Uredništvu u roku od osam dana od primitka rukopisa sa navedenim zahtjevima.


Prava izdavača u vezi s radovima

Autori su suglasni da njihovi radovi budu postavljeni na mrežnu stranicu časopisa, odnosno na mrežnu stranicu Hrvatskog ureda za osiguranje. Autori potpisuju ugovor s Hrvatskim uredom za osiguranje kao izdavačem (dalje izdavač). Objavljeni radovi honoriraju se prema odluci izdavača. Časopis i izdavač zadržavaju i sva ostala prava u odnosu na rad i objavu rada, osim ako nije drugačije dogovoreno s autorom.


2

Hrvatski časopis za OSIGURANJE


Tehničke osobine radova

Rad treba imati od 10 do 15 stranica. Pod „stranicom" se misli na veličinu A4 (21 x 29.7 cm; portret orijentacija). Svaki rad treba sadržavati naslov, sažetak na hrvatskom (100 – 300 riječi), ključnih riječi (do 5), uvoda, glavnog dijela rada, zaključka, popisa navedenih izvora i korištene literature, sažetka na engleskom jeziku i ključnih riječi na engleskom (do 5). Izuzetno mogu se prihvatiti i opsežniji radovi.

Autori su suglasni da prijevod sažetka na engleski jezik ako ne bude dostavljen, izvrši Uredništvo.

Tehničko oblikovanje rada:

·	format: A4

·	margine: 2,5 cm

·	font: Times New Roman

·	na početku rada u gornji lijevi ugao napisati ime i prezime i titulu te kao fusnotu prezimena/titule, upisati ime, prezime i titulu, naziv institucije, e-mail adresu autora. Slijedi prazan red, zatim naslov.

·	NASLOV: velika tiskana slova, veličina fonta 14, podebljano (bold), centrirano.

·	glavni dio rada (uključuje i sažetak uklopljen u rad): Times New Roman 12, prored 1.5.

·	sažetak i ključne riječi pisati u kurzivu (italic)

·	obostrano poravnanje

·	prored 1.5 za cijeli rukopis, osim za sažetak i ključne riječi

·	Naslove poglavlja i potpoglavlja ne uvlačiti.

·	U tablicama: Times New Roman 10, prored 1.0., obostrano poravnanje

·	sve stranice je potrebno numerirati

·	riječi na stranom jeziku navode se u kurzivu (italic)

·	naslovi poglavlja i potpoglavlja se numeriraju (preporuka je da se naslovi poglavlja numeriraju brojevima 1, 2, 3…; naslovi druge razine 1.1., 1.2.,…; naslovi treće razine 1.1.1., 1.1.2., … i ne više od toga.).

·	Ako su u radu korištene kratice i simboli, uz rukopis je potrebno priložiti objašnjenje o njihovu značenju.

·	Bilješke (fusnote): Times New Roman 10. Poravnanje na obje strane. Bilješke (fusnote) se koriste isključivo radi objašnjenja, dopune ili komentara, a objavljuju se na dnu stranice

Tablice, grafovi i ilustracije trebaju biti crno-bijele (izbjegavati sjenčanje i sl., budući da takvi efekti umanjuju preglednost), uglavljene u tekst rada, ali ne „zaključane", numerirane i imati svoj naslov i opis (npr. Tablica 1. Obrazovna struktura uzorka).

Sve grafike trebaju biti visoke kvalitete. Potrebno je voditi računa o autorstvu svih materijala u radu..

Uredništvo pridržava pravo da rad redakcijski prilagodi propozicijama časopisa.

Sažetak rada treba sadržavati opći prikaz teme rada iz kojeg je vidljiva njegova svrha, metodologiju rada, rezultate istraživanja ako je bilo provedeno i zaključak.

Ključne riječi sadrže pojmove i izraze važne za brzu identifikaciju i klasifikaciju sadržaja rada. Treba voditi računa da to budu relevantni pojmovi za obrađenu temu te da ne budu opće i preširoke, ali ni preuske naravi opisani s previše riječi.


3

Hrvatski časopis za OSIGURANJE


Navođenje referenci unutar teksta

Citirane i parafrazirane izvore navodi se u tekstu, a ne u bilješkama. Stavlja ih se u zagrade uz navođenje prezimena autora i godine izdanja, npr. (Horvatić, 2003), a u slučaju citata navodi se još i strana (Horvatić, 2003:150). Svaka bilješka navodi se kao i prvi put. Ako se radi o dvojici autora: (Horvatić i Perić, 2012). Ako je više od 2 autora navesti prvog i pisati: (Horvatić i suradnici, 2003) ili (Horvatić i sur., 2003) ili (Horvatić et al., 2003). Svaka referenca mora se navesti u popisu literature.


Navedeni izvori i korištena literatura

Literatura se ne numerira. Uređuje se abecednim redom autora te kronološki za radove istog autora, a ukoliko je više radova istog autora objavljenih iste godine, uz godinu se rabe oznake "a,b,c" (npr., 2011a, 2011b itd.).

Literatura se citira prema primjerima za knjige, časopise i ostale izvore (nije ih potrebno sortirati, već navesti prema abecedi).


PRIMJERI ZA:

Knjige

Prezime, I. (godina), Naslov, Mjesto izdavanja, Ime izdavača

Horvatić, V. (2003), Kako napisati znanstveni rad, Zagreb, Naklada Perić

Ukoliko su dva ili tri autora, redom navesti njihova prezimena i inicijale, a ukoliko je četiri ili više autora, navodi se prezime prvog autora nakon čega slijedi: et al.


Časopisi

Prezime, I. (godina), "Naslov članka", Naziv časopisa, broj sveska, volumena ili godišta (broj u tekućem godištu), raspon stranica

Ukoliko je više autora (četiri ili više), navodi se prezime prvog autora nakon čega slijedi: et al.

Lovrinčević, Ž. et al. (2005). „Kako optimalno regionalizirati Hrvatsku?", Ekonomski pregled, 56 (12), 1109-1160


4

Hrvatski časopis za OSIGURANJE


Izvori preuzeti s Internet stranica

0.	Prezime i inicijali imena autora (ako je poznat).

0.	Potpuni naslov članka (djela) – stranice.

0.	Točan izvor - internet adresa izvora

0.	Datum preuzimanja sadržaja s interneta (podjećeno DATUM)


Sustav zaštite posjetitelja u EU. http://huo.hr/hrv/sustav-zastite-posjetitelja-u-eu/108/ (posjećeno 01.07.2013.)


Radovi u zborniku, poglavlje ili članak u knjizi

Prezime, I. (godina), "Naslov i podnaslov poglavlja ili članka u knjizi ili rada u zborniku", U: Naslov knjige/zbornika, prezime i ime urednika knjige/zbornika (ur.), mjesto izdavanja, izdavač, raspon stranica


Keglević, A. (2014), „Zajednički referentni okvir i europsko ugovorno pravo osiguranja", U: Zbornik s međunarodne znanstveno-stručne konferencije Dani hrvatskog osiguranja 2014., Sanja Ćorić et al. (ur.), Zagreb, HGK/HUO, 9-22


Propisi

Zakon o radu. NN 93/14


Uredništvo


5

Hrvatski časopis za OSIGURANJE
